# Synthetic Biology of Plants and Microbes for Agriculture, Environment, and Future Applications

**DOI:** 10.1021/acs.chemrev.4c00687

**Published:** 2026-01-28

**Authors:** Phillip Clauer, Angelina X. Nou, Tyler Toth, Qiguo Yu, Yonatan Chemla, Alice Boo, Kwan Yoon, Christopher Voigt

**Affiliations:** 1 Synthetic Biology Center, Department of Biological Engineering, 2167 Massachusetts Institute of Technology, Cambridge, Massachusetts 02139, United States

## Abstract

Agriculture is under pressure to provide food for a growing population and the feedstock required to drive the bioeconomy. Methods to breed and genetically modify plants are inadequate to keep pace. When engineering crops, traits are painstakingly introduced into plants one-at-a-time, combine unpredictably, and are continuously expressed. Synthetic biology is changing these paradigms with new genome construction tools, computer aided design (CAD), and artificial intelligence (AI). “Smart plants” contain circuits that respond to environmental change, alter morphology, or respond to threats. Further, the plant and associated microbes (fungi, bacteria, archaea) are now being viewed by genetic engineers as a holistic system. Historically, plant health has been enhanced by many natural and laboratory-evolved soil microbes marketed to enhance growth or provide nutrients, or pest/stress resistance. Synthetic biology has expanded the number of species that can be engineered, increased the complexity of engineered functions, controlled environmental release, and can assemble stable consortia. New CAD tools will manage genetic engineering projects spanning multiple plant genomes (nucleus, chloroplast, mitochondrion) and the thousands of genomes of associated bacteria/fungi. This review covers advanced genetic engineering techniques to drive the next agricultural revolution, as well as push plant engineering into new realms for manufacturing, infrastructure, sensing, and remediation.

## Graphical abstract

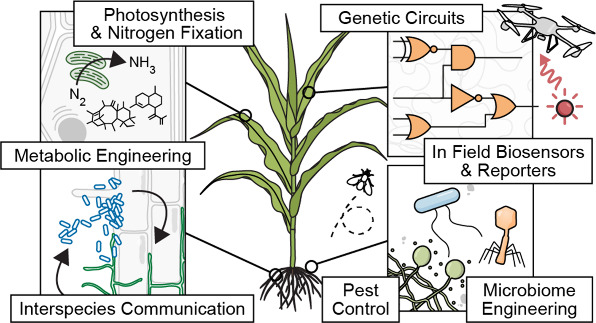

## 1 Introduction

Traditional plant breeding and precision farming are not keeping pace with the demands of global population growth.^1^ In addition to food, the growing bio-economy places increased pressure on agricultural products as feedstock for energy, chemicals, materials, and medicines that were previously derived from nonrenewable sources.^2^ As of 2018, engineered plants were grown on 500 million acres (1.9 million km^2^), benefiting about 2 billion people.^3^ Higher yields need to be obtained from less land, without destroying the environment. The United Nations projects that by 2050, crop yields must increase by 50% compared to 2013, but the reality is that productivity is stagnating.^4,5^ Over the last 30 years, genetic engineering has improved yields while lowering carbon emissions and has reduced the impact of uncertainties such as pests and weather. However, combining these functions in a single plant (“trait stacking”) has been difficult and it remains a guessing game as to which combination of seeds and traits should be planted each year. In many cases, the widespread use of engineered crops and microbes has been slowed by costly regulatory hurdles, international disparity in rules, and the lack of public acceptance.^6^ As a result, the ancient blights of agriculture remain; 60% of the yield losses in major crops are due to abiotic stress (drought, heat, salt, etc.) and 30% are due to pathogens and pests.^7,8^ Moreover, it is estimated that climate change could reduce yields by 10% or more by the 2050s.^9^

Synthetic Biology encompasses tools for the design and genetic construction of living organisms.^10^ While developed with model species, such as *E. coli* and *S. cerevisiae*, the technologies are now being applied to species of agricultural relevance, from plants and soil microbes to insects, animals, and viruses.^11–13^ Crop genomes are more easily designed to carry multi-gene traits at precise locations in their chromosomes, thus simplifying trait stacking. Synthetic regulatory networks (“genetic circuits”) reduce the load of traits on cells by turning them on only when needed, for instance, responding to a pest attack by releasing a pulse of insecticide. Plants grow slowly, making it difficult to build and evaluate complex designs. This challenge could be addressed by advances in computer aided design (CAD) to improve the likelihood of success and automated prototyping pipelines that could evaluate thousands of designs in parallel using cell-free or protoplast systems. Machine learning (ML), combined with -omics and rapid prototyping, enables the mapping of these results to performance in a crop plant in the field. This review looks at the emerging application of these concepts to crop plants and soil microbes. These advances extrapolate to a future where agricultural solutions involve interlocked consortia encompassing engineered plants, microbes, and insects, all working in concert to maximize yields and fend off threats (Figure 1).

**Figure 1:**
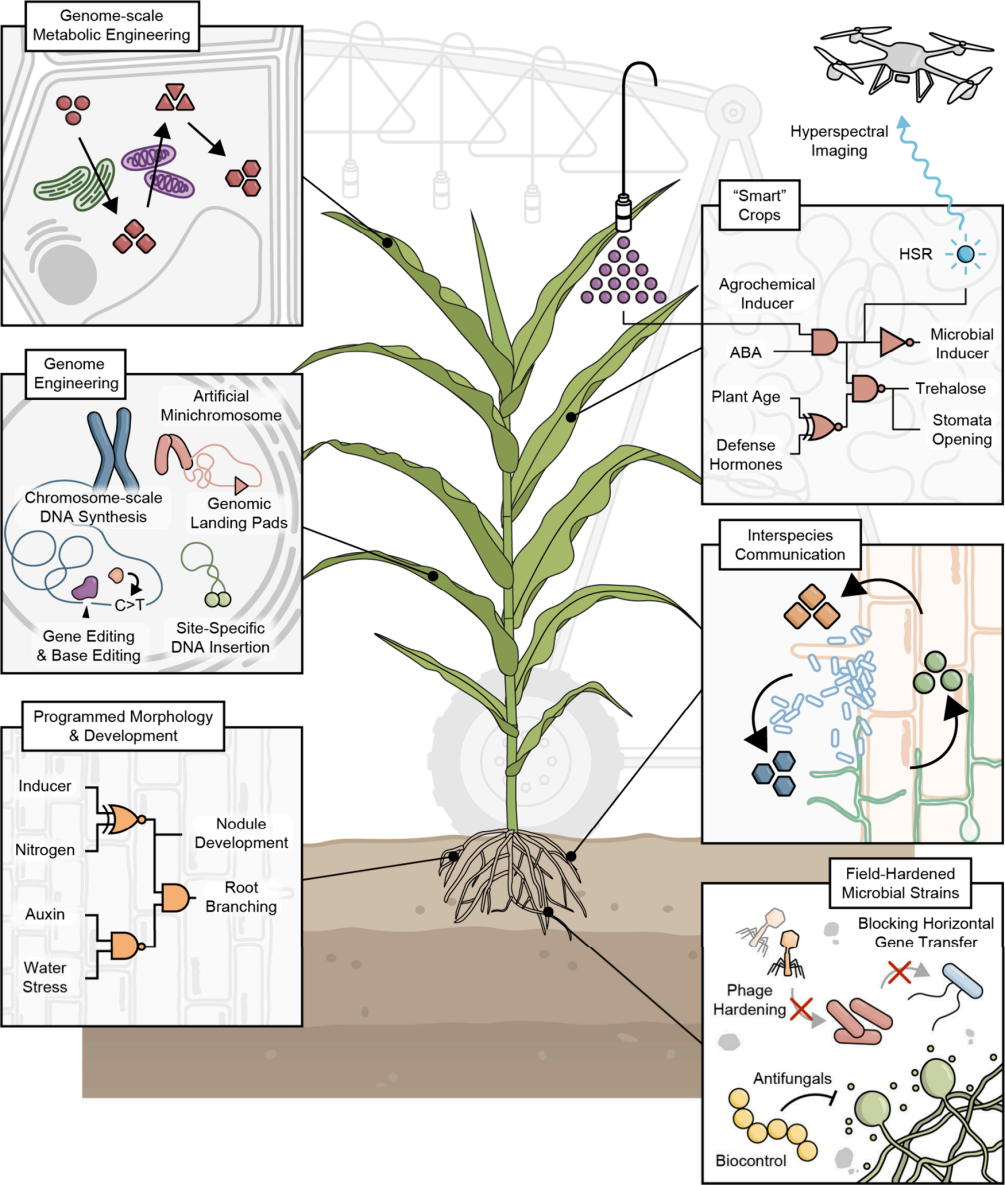
**Overview of advanced agricultural genetic engineering covered in this review**. (**Top Left**) Metabolic engineering (Section 2.2.2) to improve nutrition, bioproduction, and photosynthetic efficiency (Section 2.2.2). Enzyme genes can be introduced into chromosomes or organelle genomes (Section 2.1.4). (**Top Right**) “Smart” crops will respond dynamically to environmental stress and disease using synthetic genetic circuits (Section 2.3). The sensors can respond to agrochemicals (Section 2.3.4) and circuits produce reporters (HSR: hyperspectral reporter) to be imaged by unmanned aerial vehicles (UAVs). (**Center Left**) New genome engineering methods (Section 2.1) enable the modification of the native genome (*e.g.*, to redirect flux through a pathway or knockout a function). The use of DNA synthesis (Section 2.1.5) enables the construction of large and more complex genetic designs that can be more reliably inserted into landing pads (Section 2.1.2.2) and artificial chromosomes (Section 2.1.2.3). (**Center Right**) Signaling pathways between microbes and plants will allow the passage of information across kingdoms (Section 3.4.2). For example, the plant could signal to the bacteria a need to produce antimicrobials or insecticides (Section 3.1). The bacteria could signal to the plant environmental conditions or the presence of nutrients in the soil (Section 3.4.1). (**Bottom Left**) Genetic circuits in the plant could respond during growth to environmental changes and alter the plant morphology, such as changing the root length or grain yield (Section 2.3.6). (**Bottom Right**) These engineered crops will communicate with microbes (Section 3.4.2) designed to perform growth-enhancing functions through microbial pathway engineering (Section 3.3). The construction of consortia could make them more resilient to environmental perturbations (Section 3.5.7). Genetically modified microbes could have biocontainment mechanisms that prevent DNA transfer (Section 3.5). The consortia will include engineered nonmodel soil bacteria (Section 3.1), fungi (Section 3.7.2), and archaea (Section 3.7.1).

This review covers the application of the principles of Synthetic Biology to agriculture and paints a picture as to where the technologies are headed in the near and far future. The first section describes the impact of genome editing, early collections of genetic parts and the construction of the first genetic sensors and circuits toward the creation of “smart plants” that can respond to their environment. Then, the engineering of other species of relevance are described, from soil bacteria to phage and fungi and how these could be connected into integrated systems. Throughout, we cover the advances from other areas that are not yet being applied to plant synthetic biology, but likely will be soon, from whole chromosome synthesis and replacement to opportunities for advanced computer aided design (CAD) and artificial intelligence (AI). We also lay out grand challenges, where we relax the constraints of current technical feasibility, to envision the impact of a future of highly engineered agricultural systems. Even these visions are timid compared to what will be possible when we have full design control over the 100 gigabases of DNA^14,15^ encoding the complete agricultural system, not just the genome of the crop, but all the bacteria and fungi and supporting insects, all of which could be encoded in a single treated seed. Finally, we discuss the remaining challenges, technology gaps, and promising future directions of genetic engineering applied to agriculture.

## 2 Plant Design and Construction

### 2.1 Engineering Plant Genomes

Precision genome editing is a needle-in-the-haystack problem, requiring the targeting of cleavage to a specific basepair in a large genome. This remarkable feat is now possible using DNA-binding domains where the targeted sequence can be programmed. Many variants of these methods can knockout genes, make specific base pair changes, or drop payloads of synthetic DNA into the genome. These payloads can be delivered by plant viruses or *Agrobacterium*, either as a laboratory technique or to edit plants in the field. Instead of simply editing a natural genome, it has been a dream to build a complete genome from scratch using only chemistry. While not yet applied to plants, entire eukaryote chromosomes have been built using *de novo* DNA synthesis and assembly, which can be used to radically reorganize native genetics or make systematic changes throughout the chromosomes.

#### 2.1.1 Editing Chromosomes

Genome editing tools delete, modify, or insert DNA at a precise location in the genome.^16,17^ DNA recognition and cleavage can be performed by meganucleases, zinc-finger nucleases (ZFNs), transcription activator-like effector nucleases (TALENs), or CRISPR/Cas systems, all of which have been shown to work in plants.^18–21^ Meganucleases, ZFNs, and TALENs have domains that can be reprogrammed to theoretically target any desired DNA sequence. In practice, they all have limitations. Meganucleases and ZFNs are particularly difficult to design, with only a handful of examples of their use in crop plants.^17,18,22–26^ TALENs are more easily designed by combining domains, each of which contributes modularly to the DNA sequence that is recognized.^27,28^ CRISPR/Cas9, however, relies on an easily programmed guide RNA (gRNA) to target a desired DNA sequence; thus, it has rapidly become the method of choice for engineering plants.^21,29,30^ In a survey of genome edited crops to 2020, 161 traits have been introduced using Cas9 and 20 using TALENs.^31^

Despite its success and widespread use, Cas9 is still limited by its large size, off-target effects, and the requirement of a protospacer adjacent motif (PAM) next to the target site.^32,33^ In eukaryotes, editing efficiency is dependent on chromosome access and remodeling,^34^ and it has been observed in plants that gRNA can lead to inhibition even in the absence of Cas9.^35^ Using engineered or alternative CRISPR variants can address some of these limitations. For example, CRISPR/Cas12a (Cpf1) has lower off-target activity than Cas9.^36,37^ CRISPR/Cas12a was used to edit the promoters of two *csLOB1* genes in Duncan grapefruit to increase its resistance to citrus canker, a feat not possible with CRISPR/Cas9 due to its limited specificity.^38^ CRISPR systems with relaxed or alternative PAM requirements have been developed through rational engineering or directed evolution, thus allowing for more positions in the genome to be targeted.^39–41^ For example, the engineered variant Cas9-NG is active at NGN PAM sites and has been used in *Arabidopsis*, tobacco, rice, *Brassica*, and tomatoes.^42–46^ The PAM-independent SpRY variant showed activity at nearly all sequences tested in rice protoplasts, although at varying efficiencies.^47^ The “miniature” Cas12f and CasΦ are less than half the size of Cas9, thus simplifying delivery into the cell. Two versions of CasΦ function in plants.^48–52^

Following a nuclease-mediated double-stranded break (DSB), a DNA repair template can introduce specific mutations or payloads to the genome through homologous recombination (HR). However, the rate of homologous recombination in plants is low and so cleaved DNA is primarily repaired through nonhomologous end joining (NHEJ).^58^ This results in small insertion or deletions (indels) of nucleotides at the site of cleavage, often disabling the targeted gene. There are many examples of crops being improved using gene knockouts performed through gene editing. Prior to genome editing technology, screening campaigns identified many such examples, but the techniques required time and labor-intensive mutagenesis or the insertion of recombinant genes onto the genome.

These crops have been treated by the USDA as GMOs, which increases development cost and time. For example, the Flavr Savr tomato achieved extended storage life through the suppression of the polygalacturonase (*PG*) gene by RNAi.^59^ The same modification can be recapitulated by CRISPR/Cas to generate a tomato *PG* mutant.^60^ Genome editing techniques that leave no foreign DNA behind simplify the process of obtaining regulatory approval.^10^ The first genome-edited crop to enter the US food supply was a high-oleic soy with improved oil stability, developed by knocking out two fatty acid desaturase genes (*FAD2-1A* and *FAD2-1B*) with TALENs (Figure 2A).^53^ This crop entered the market in 2019 after the USDA decided not to regulate it and other crops that do not contain foreign DNA.^61,62^ In 2016, the first CRISPR-edited food product to gain this status was an anti-browning white mushroom made by knocking out polyphenol oxidase (*PPO*).^63^ This crop was followed by a waxy corn, high-oil-content camelina, and drought tolerant soybean.^62^ Recently, the first CRISPR-edited aminobutyric acid (GABA)-rich tomato entered the market in Japan.^62,64^

**Figure 2:**
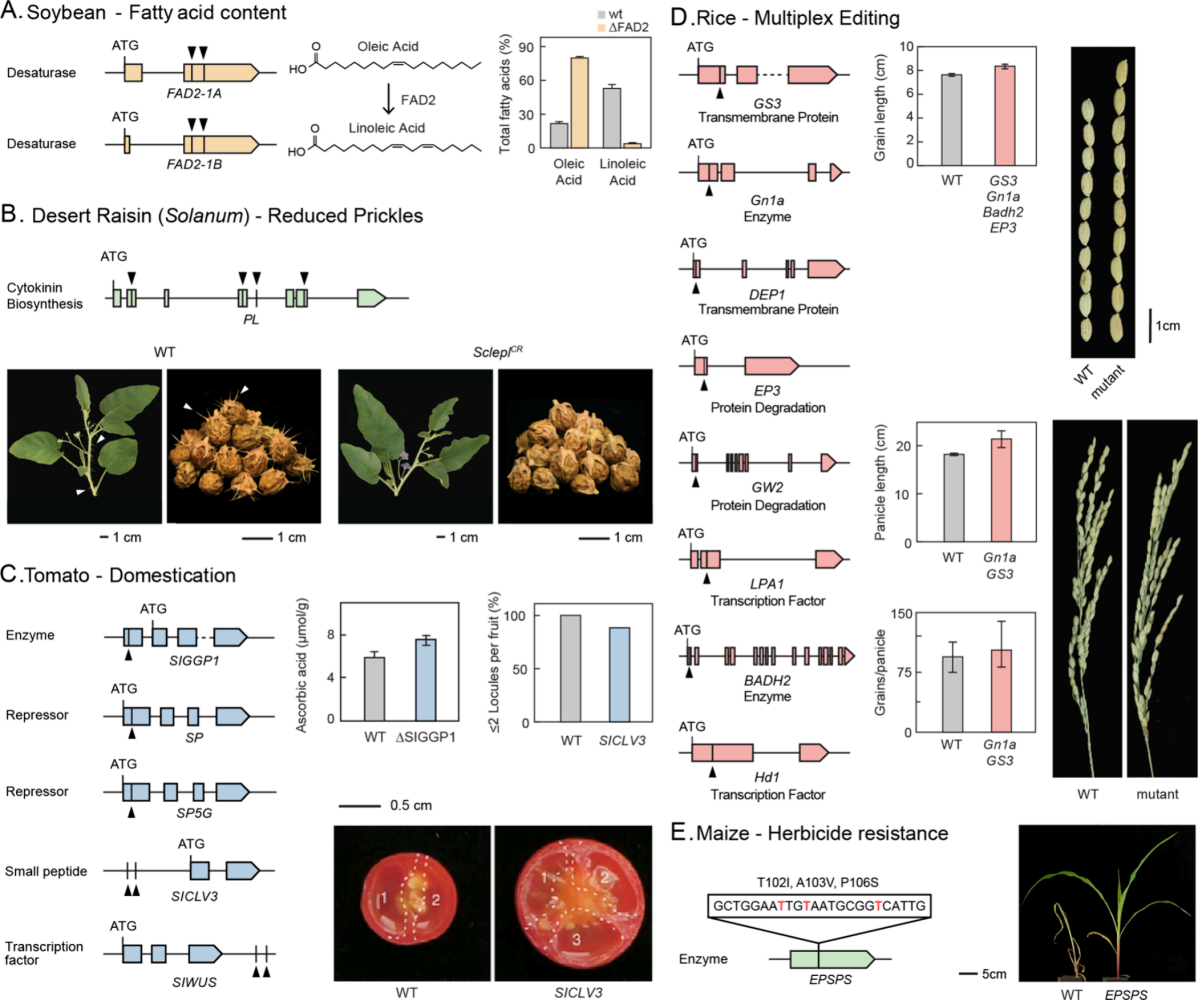
**Improving agronomic traits through gene editing. A**. Increasing soybean oleic acid content.^53^ The disruption of *FAD2-1A*/*FAD2-1B* by TALENs decreases the conversion of oleic acid to linoleic acid. **B**. Improving *Solanum* harvestability by editing genes involved in prickle development.^54^ The cytokinin biosynthesis gene pricklesss (*PL*) was targeted by four gRNAs in *Solanum cleistogamum* to develop mutant plants (*SclepI* ^*CR*^) devoid of thorny prickles on stems and fruit (white arrows) without leading to unwanted pleiotropic effects. WT: wild-type. Image of plants and fruits reproduced with permission from ref 54. Copyright 2024, Science. **C**. Tomato relative domestication using multiplexed targeting of genes using CRISPR/Cas9.^55^ *Solanum pimpinellifolium* is a stress- and pest-resistant relative of domesticated tomatoes. A set of domestication traits were made simultaneously. The triangles mark the locations of the knockouts. Edits targeted to the upstream open reading frame (μORF) of the vitamin C biosynthetic enzyme *SlGGP1* led to lines with higher foliar vitamin C (ascorbic acid) content. The *SlCLV3* gene is associated with fruit locule number (number of separated seed compartments within a single fruit), which is associated with fruit size. The dotted lines illustrate locules in a wild-type and edited fruit. Plants with edited *SlCLV3* alleles had a lower frequency of fruit with 2 locules or fewer. Image of tomato reproduced with permission from ref 55. Copyright 2018, Nature Biotechnology. **D**. Multiplexed editing of Nipponbare rice simultaneously targets multiple trait loci associated with improved yield.^56^ Eight genes were targeted, five implicated in high yield (*GS3*, *Gn1a*, *DEP1*, *EP3*, *GW2*) that affect grain length, width, weight, and number. The others affect plant architecture (*LPA1*), rice fragrance (*BADH2*), and photoperiod (*Hd1*). WT: wild-type. One line containing a combination of edits to rice QTLs produced grains with a larger average length (“mutant”, top). A second line produced larger panicles, the branching structure where grains develop, with a higher density of grain (“mutant”, bottom). Images of rice reproduced with permission from ref 56. Copyright 2017, Science China Life Sciences. **E**. Herbicide resistance through prime editing.^57^ The 5-enolpyruvylshikimate-3-phosphate synthase (EPSPS) gene in maize was targeted with a SpCas9 nickase-based prime editor and single prime-editing guide RNA, while expressing a dominant negative MutL homolog (MLH1dn) to inhibit mismatch repair. Prime edited T1 mutants (*EPSPS*) contained three single base pair substitutions (red letters) in the gene to generate glyphosate-resistant maize (WT: wild-type). Maize seedlings at the V4 stage were sprayed with 10 mM glyphosate. Images of maize plants reproduced with permission from ref 57. Copyright 2023, Journal of Integrative Plant Biology.

##### 2.1.1.1 Multiplexed Genome Editing

Multiple genome sites can be simultaneously targeted for editing using multiplexed methods. In a remarkable example of parallel editing, 107 of the 109 members of the caffeic acid *O*-methyltransferase (*COMT*) gene family were mutated using TALENs in sugarcane to improve bioethanol production from lignocellulosic biomass.^65^ The simplicity of CRISPR/Cas targeting makes it possible to affect many genes at once by simultaneously expressing multiple gRNAs. These approaches include expressing gRNAs as individual transcripts from nonrepetitive RNA polymerase III promoters and terminators (*e.g.*, the U6 promoter)^66,67^ or as a single polycistronic transcript processed into separate gRNAs using the intrinsic RNA processing machinery from CRISPR endonucleases (Cpf1),^68^ CRISPR-derived ribonucleases (Csy4),^69^ self-cleaving ribozymes,^70^ or endogenous tRNA-processing systems.^71^

Multiplexed gRNAs have been used to improve lycopene content in tomato fruits (6 gRNAs),^72^ decrease the glycoalkaloids in potato tubers (9 gRNAs)^73^ and eliminate the glycan in tobacco (14 gRNAs).^74^ In the genus *Solanum*, which contains eggplant, tomato, and ornamental roses, up to 5 gRNAs were targeted to the cytokinin biosynthesis gene prickleless (*PL*) to generate plants lacking thorny projections called prickles (Figure 2B).^54^ In addition to making null mutations to genes responsible for specific traits, gene editing can be used to generate genetic diversity in regions that contribute to complex quantitative traits (yield, fruit size, plant architecture, etc.). In tomato, variation in fruit size was obtained by targeting 8 gRNAs to the noncoding region upstream of *CLAVATA3*, which is involved in the feedback circuit controlling meristem proliferation and seed number.^75^

Multiplexed CRISPR/Cas methods can simultaneously target many genes implicated in separate traits to accelerate crop improvement. In tomato, multiple genes were targeted that are involved in synchronized fruit ripening, day-length insensitivity, fruit size, and vitamin C content (Figure 2C).^55^ In rice, multiple genes were simultaneously targeted to control rice yield, architecture, fragrance, and photoperiod (Figure 2D).^56^ In poplar, combinations of 21 lignin biosynthetic genes predicted by a metabolic model were targeted to improve wood pulp characteristics.^76^ In maize, this approach was scaled up to individually target 743 genes associated with agronomy and nutrition. Of these, 118 genes led to observable phenotypic changes including changes in plant size and morphology, flowering time, response to day length, and susceptibility to disease.^77^

A common barrier to genome editing in plants is the presence of unexpected paralogs which are functionally redundant to the target. This can prevent phenotype improvements since paralogs can compensate for the altered function of the edited gene. Single gRNAs can be designed to simultaneously target multiple paralogs within a gene family. For example, a library of 5,635 individual gRNAs, each targeting between 2-10 *Arabidopsis* transporter genes, was used to generate *Arabidopsis* lines.^78^ This led to the discovery of a family of tonoplast hormone transporters, which only became detectable by an altered phenotype when both paralogs were mutated simultaneously. In bread wheat, α-gliadins are a component of gluten and are encoded by a gene family of roughly 100 members; up to 35 genes were simultaneously edited by targeting conserved regions of α-gliadins with two sgRNAs, which reduced the gluten composition in the edited wheat grain.^79^

##### 2.1.1.2 Base Editors

Genome editing tools can also make single nucleotide replacements in the genome. Oligonucleotide-directed mutagenesis, an early technique used to make herbicide resistant crops, relied on double stranded DNA:RNA oligos containing the desired nucleotide change within the target sequence.^80^ This approach suffered from low efficiency.

Base-editors make single targeted replacements by use of a nuclease-deactivated Cas9 (dCas9) to position an enzyme capable of C-to-T (cytosine base editor) or A-to-G (adenine base editor) mutations.^81^ Various dual base editors function in plants that can introduce both C-to-T and A-to-G substitutions by fusing cytidine and adenosine deaminases to a Cas protein.^82^ Herbicide resistant rice, wheat, maize, cassava, and watermelon have been created by making mutants of the enzymes targeted by the herbicides.^83–89^ In strawberries, base editors were targeted to a regulatory region upstream of a transcription factor (TF) that controls sugar metabolism to increase the sugar content in fruit.^90^ Base-editing has also been used to substitute a single base pair in the *ALC* gene to create a nonsynonymous amino acid change that increased the shelf life of tomato.^91^

Prime editors can generate all 12 types of base conversions without the need for double-strand breaks or donor DNA templates.^92^ They consist of a prime editor (a fusion protein between Cas9 nickase and reverse transcriptase) and the prime-editing guide RNA (pegRNA). The pegRNA contains the primer binding site and the reverse transcriptase template. The desired edits in the reverse transcriptase template can be reverse transcribed and inserted into the target site. Herbicide resistant rice, wheat, maize, tomato, and *Arabidopsis* have also been made using prime editing (Figure 2E).^57,93–96^ Note that prime editing has been found to be much less efficient in dicots.^97^

##### 2.1.1.3 Targeted Random Mutagenesis

Directed evolution is a powerful tool to optimize biological systems, including individual proteins, pathways, and entire microbial genomes.^98–102^ Rounds of diversity creation (*e.g.*, mutagenesis) are followed by screening to iteratively improve a property-of-interest. The plant genome can be mutagenized using CRISPR machinery through imprecise double strand break repair. In rice, it has been applied to target a gene that confers resistance to the herbicide herboxidiene.^103^ Mutations were made to the entire length of the gene by tiling 119 gRNAs, followed by screening 15,000 transformed calli (undifferentiated masses of plant cells) on media containing herboxidiene to identify insensitive mutations.

Base editors can be used to perform targeted saturation mutagenesis.^104^ For this purpose, a base editor was developed to simultaneously perform C-to-T and A-to-G conversions by fusing a cytidine deaminase, adenosine deaminase, Cas9 nickase, and uracil DNA glycosylase inhibitor.^82,105^ The tool, called STEMEs, performed saturation mutagenesis of a rice gene to obtain herbicide resistance.

##### 2.1.1.4 Computer Aided Design (CAD) of Genome Editing

Software for genome editing aids the design of proteins (TALEs, ZFPs) or sgRNA (Cas9) to target specific chromosomal sites. TALEN Targetter 2.0 provides a web-based interface that identifies potential TALENs target sites within a user-provided DNA sequence and outputs the corresponding TALENs amino acid sequence.^28,106^ TALEN Targeter 2.0 can design TALENs for use in any organism with a published genome sequence and has been applied to crops, including rice,^107^ corn,^108^ and wheat.^109^ Custom TALENs designed to mutagenize fatty acid desaturase 2 (FAD2) in peanuts using TALEN Targeter (TALE-NT) led to a 2-fold increase in the oleic acid.^110^ Beyond genome editing, TALE-NT 2.0 has been used to predict the binding of TALE effectors from *Xanthomonas oryzae* in rice to explain the effect of sequence polymorphisms in resistance genes.^111^ ZZiFiT is an online tool that designs an amino acid sequence for a chimeric ZNF-N to target a user-specified DNA sequence.

CRISPR/Cas gRNA design software packages are available to design optimal gRNAs and predict on-target activity and avoid off-target events. Many popular gRNA design tools, such as CRISPOR,^112^ Cas-Designer,^113^ CRISPR-P,^114,115^ and PE-Designer^116^ have been applied to plant genomes.^45,117,118^ To introduce quantitative trait variation, the web-tool CRISPR-P was used to design an array of nine gRNAs that simultaneously targeted the promoter region of two maize CLE genes, which are associated with increase in kernel and ear size.^118^ The tools differ in their evaluation of gRNA efficiency and specificity, selection of reference genomes and CRISPR endonucleases, and ability to predict genome-wide off-target events. The accuracy of these tools have been validated for plant genome editing.^119,120^

#### 2.1.2 Chromosomal Insertion of Synthetic DNA

DNA payloads can be inserted into the genome; for example, to introduce a trait involving a recombinant enzyme or pathway. Previous methods inserted the payload at multiple random locations and the resulting plants would be screened for those with the desired phenotype and no discernable defects.^121–124^ This process complicates trait stacking because genes must be added serially and screened afresh leading to different insertion locations than the plants originally screened for individual traits. Due to this limitation, to our knowledge, the most traits put into a single commercial crop was seven in maize (one herbicide tolerance and six insect resistances).^125^

In contrast, providing a repair template along with the site-specific nucleases, payloads can be inserted at a specific location using NHEJ and HR. These insertions have been used to change the regulatory control of a gene; for example, maize drought tolerance was improved by replacing a native promoter with a stronger one to increase the expression of a negative regulator of ethylene responses.^126^

There are technical limitations on the size of a DNA payload that can be delivered to a plant cell. There have been examples of the delivery of large DNA sequences, for example, rice has been engineered to produce anthocyanin and astaxanthin by introducing ten (31 kb total) and four (15 kb) recombinant genes, respectively (Figure 3).^127,128^ Wheat resistant to a fungal pathogen was generated by introducing a 37 kb construct encoding five resistance genes into a single locus^129^ and two pathogen resistance gene clusters (20 kb) from *Arabidopsis* were introduced into soybean.^130^ The largest DNA fragment delivered into a plant nucleus using *Agrobacterium*-mediated transformation is 150 kb (a piece of human genomic DNA).^131^

**Figure 3:**
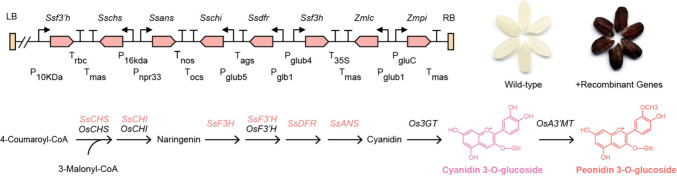
**Biofortifying rice through anthocyanin production**. A 31 kb construct containing native endosperm-specific promoters from rice and commonly used plant terminators was transformed using *Agrobacterium*.^127^ The construct also included two TFs from maize (ZmLc and ZmPl) known to upregulate anthocyanin biosynthesis genes along with six biosynthetic genes *ssF3′H* (flavonoid 3′-hydroxylase), *ssCHS* (chalcone synthase), *ssANS* (anthocyanidin synthase), *ssCHI* (chalcone isomerase), *ssDFR* (dihydroflavonol 4-reductase), and *ssF3H* (flavanone 3-hydroxylase) from the highly pigmented plant Coleus (*Solenostemon scutellarioides*) flanked by the Left (LB) and Right Border (RB) sequences from the *Agrobacterium* T-DNA. The anthocyanin biosynthetic pathway is depicted including the recombinant genes (red) and key endogenous genes (black). Images of rice grains reproduced with permission from ref 127. Copyright 2017, Molecular Plant.

A challenge with making large insertions is obtaining sufficient concentrations of template DNA inside the plant cell to trigger NHEJ or HR. This has been addressed by using the geminivirus replicon to amplify the template DNA within a cell, which increases HR efficiency in potato, tomato, wheat, and rice.^132–134^ Alternatively, chemically-modified double-stranded oligodeoxynucleotides can be used to facilitate HR. These nucleotides have been used to insert a 60 bp translation enhancer and a 2 kb promoter in rice and this approach could theoretically insert complete protein-coding genes.^87,135–137^

##### 2.1.2.1 Site-Specific Transposons

Some applications of gene editing in plants, such as insertion of a payload into a landing pad or an enhancer into a plant promoter (Section 2.2.1.1.1), require delivering a large genetic payload to a specific genomic site. Homologous recombination is often used to deliver DNA to bacterial, yeast, and mammalian genomes, but the efficiency of HR in plants is very low.^138^ DNA repair-based insertion methods can also be prone to small unwanted insertions or deletions.^139^ One alternative is to use site-specific transposase systems, which use a site-specific endonuclease and a high-efficiency transposase to specifically and efficiently deliver a DNA payload to a target genomic site. Most commonly, these systems use natural CRISPR-associated transposases from prokaryotes and to guide the DNA to a user-specified in bacterial or mammalian (including human) cells.^140–143^ However, these bacteria-derived programmable transposases have not been shown to be functional in plants.

Transposase-assisted target-site integration (TATSI) site-specifically inserts a DNA payload into the plant genome, demonstrated for *Arabidopsis* and soybean. A programmable RNA-guided endonuclease (Cas9 or Cas12) is co-expressed with hyperactive mutants of the rice *Pong* transposase (Figure 5A).^144^ A DNA payload is originally inserted within the *Pong* transposable element, which is then excised by the transposase. The DSB generated by the endonuclease at the target site is then used for integration. When *Arabidopsis* plants were transformed with vectors containing the TATSI system, > 35% of the progeny had site-specific insertions.

##### 2.1.2.2 Genomic Landing Pads

The insertion of a large DNA construct into the genome can disrupt native genes either directly or in noncoding regions by interfering with neighboring regulatory sequences. Another problem is that the function carried by the payload will be sensitive to the local chromosomal environment and background transcription. The insertion of “landing pads” into the chromosome first, into which the payload is later added, is one way to improve the reliability of the encoded function. Sometimes, insertion sites are included within the landing pad that are specific to the payload delivery method (phage integrase or transposase sites).^145^ Landing pads have been used for bacteria, yeast, and mammalian cells.^145–148^ In the maize genome, sets of landing pads were introduced into four regions of low gene density and high recombination frequency.^149^ Integration was performed using FLP recombinase and the associated *lox* recognition sequence in the landing pad. Integration into these sites was highly efficient. To speed up the development of multi-trait hybrids, landing pads were inserted at adjacent sites in different maize lines. When separate traits were individually integrated onto these landing pads, they could be stacked through chromosome crossover and genetic recombination after crossing the maize lines.

The first step of landing pad design is to identify a chromosomal location that can lead to high expression.^145^ Ideally, there is also limited variability due to environmental changes or tissue context. In other eukaryotes and bacteria, landing pad sites have been identified empirically using random transposon insertion to scan the genome with a reporter^150,151^ or using RNA-seq and machine learning (ML) to predict appropriate sites.^152^ These techniques would be difficult to use as-is in plants. However, an alternative method to find high-expression genomic regions is ATAC-seq (Assay for Transposase-Accessible Chromatin with high-throughput sequencing), which has been used in plants.^153–156^ This technique probes genome-wide DNA accessibility to identify highly-transcribed regions for recombinant gene insertion. Once a site is identified, it must be tested to ensure that the associated DNA does not affect host gene expression. This screen can be performed by measuring phenotypic or growth changes or, more thoroughly, by using RNA-seq in different tissue types, developmental stages, and environmental conditions to ensure that host gene expression is not impacted.^145,146,149,157^

Insulators can further isolate a landing pad from the genome (Section 2.2.1.3). Various approaches to insulation have been taken, including flanking it with terminators to block transcription into or out of the landing pad, factors to open up the chromatin, and insulators to protect against nucleosome changes.^158^ New classes of insulators are needed for plants to decouple expression from neighboring enhancer sequences and block expansion of DNA silencing from neighboring regions.^159^

##### 2.1.2.3 Artificial Chromosomes

Artificial chromosomes offer another means to isolate recombinant genes. In other eukaryotes, stable artificial chromosomes have been made by reintroducing centromere DNA to DNA containing genes or pathways-of-interest.^160–163^ This approach does not work in plants because their centromeres are based on epigenetic features and contain large arrays of repeats, sometimes as large as a megabase. As a result, placement of centromere sequences on an artificial chromosome does not necessarily generate the kinetochore required for spindle fibers attachment during cell division.^164,165^

An alternative approach is to build plant artificial minichromosomes through telomere-mediated truncation strategies originally developed in yeast.^166^ Minichromosomes are constructed by introducing arrays of telomeric sequences into the plant chromosomes (Figure 4A). Some of these integrations result in truncations at the site of the telomere insertion, presumably through the stabilization of one end of the T-DNA by the telomeric proteins. Originally performed in maize, stable artificial chromosomes have since been introduced to rice, barley, wheat, *Arabidopsis*, and *Brassica*.^167–177^ A preferred target for the creation of minichromosomes are B chromosomes, which are nonessential, small chromosomes found in maize and other plant species.^175^ Minichromosomes can also be generated from essential chromosomes in tetraploid plants, such as wheat, where the extra copy of the chromosome provides redundancy for any essential genes deleted by the truncation. In diploid plants such as maize, minichromosomes can be derived from essential A chromosomes by selecting for spontaneous polyploid events. A challenge to using minichromosomes is their failure to properly segregate during meiosis, resulting in erratic copy numbers during spore formation and low rates of transmission.^178,179^ Various pollen selection methods that require maintenance of minichromosomes for viability have been proposed.^166,180,181^ Artificial chromosomes can be detrimental to plant growth and their transfer between species is not always reliable.^182^

**Figure 4:**
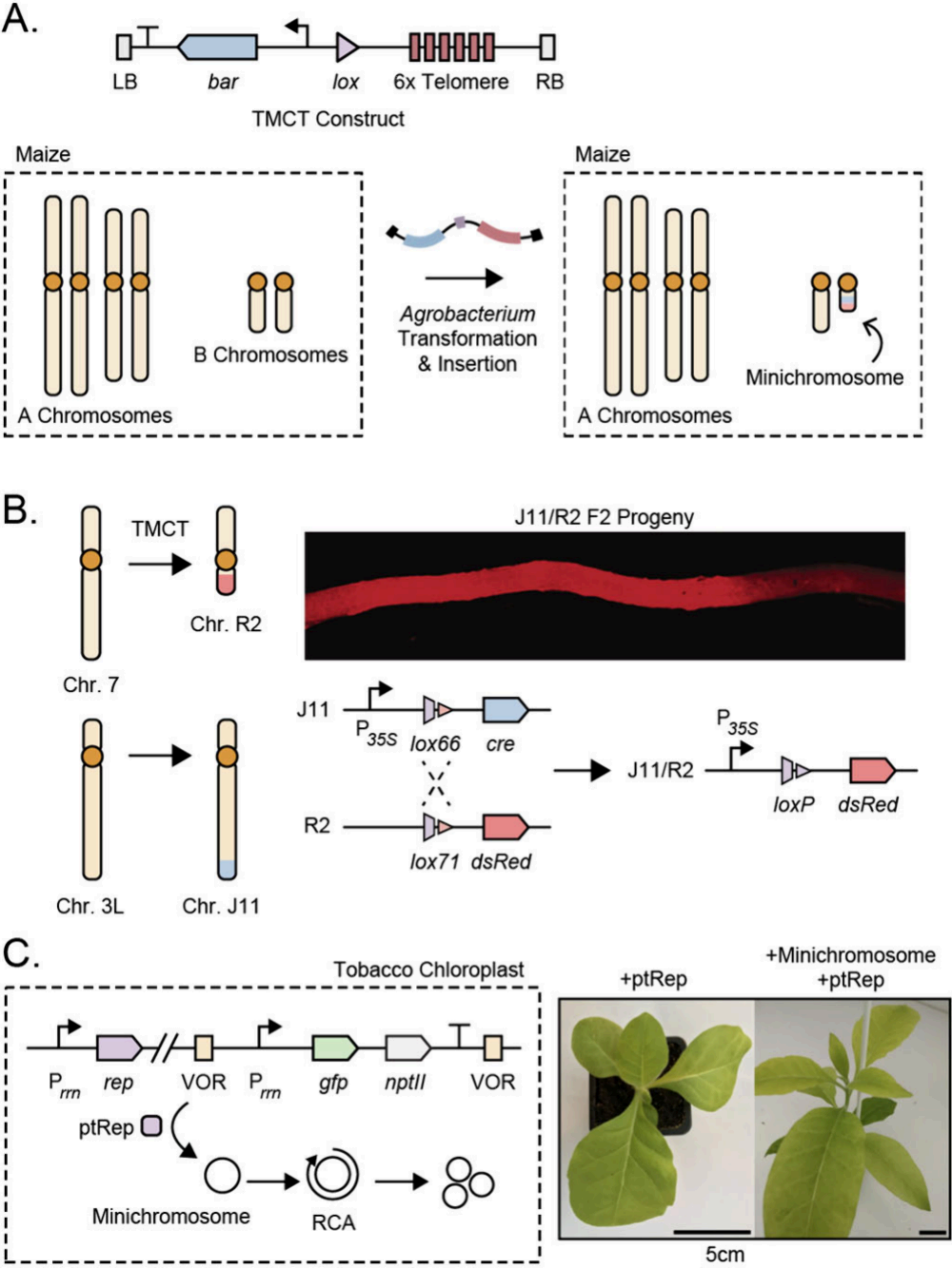
**Expressing transgenes from artificial plant minichromosomes**. **A**. Building plant minichromosomes using telomere-mediated chromosomal truncation (TMCT).^176^ Repeats of *Arabidopsis* telomere sequences are placed onto an *Agrobacterium* T-DNA construct containing a *bar* selection gene and transformed into maize cells. Site-specific recombination or insertion sites (*lox/FRT*) can be inserted to facilitate future genome engineering. A minichromosome is generated when the telomere-containing TMCT construct is inserted onto a B chromosome, leading to its truncation. **B**. Performing site-specific genome modifications in maize using artificial plant chromosomes.^175^ An artificial minichromosome (R2) was generated from chromosome 7 through TMCT and introduced a promoter-less fluorescent protein (*dsRed*) downstream of a *lox* recombination site. Separately, an artificial chromosome (J11) was generated from Chromosome 3L constitutively expressing Cre recombinase downstream of a *lox* recombinase site. When maize plants containing Chromosomes R2 and J11 were crossed, the two chromosomes site-specifically recombined to generate a functional construct (J11/R2). Maize roots of the J11/R2 cross progeny were visibly fluorescent (right image). Image of maize reproduced with permission from ref 175. Copyright 2007, National Academy of Sciences. **C**. Replicating minichromosomes derived from geminivirus in plastids.^183^ A construct flanked by viral origin of replication sequences (VOR) was inserted onto the plastid genome along with a separate transgene expressing the replication initiator protein (Rep) from beet curly top geminivirus. The Rep protein initiates replication at the VOR sequences, which results in a circular vector that undergoes rolling circle amplification (RCA) containing the sequence between the two VOR sequences. To help maintain and track the minichromosome, *gfp* and the antibiotic marker *nptII* were included on the minichromosome. Plants expressing Rep from the chloroplast (+ptREP) and maintaining the minichromosome (+minichromosome) only experienced minor bleaching in leaves and limited fitness burden. Image of tobacco plants reproduced with permission from ref 183. Copyright 2021, Nature Plants.

Artificial minichromosomes possess desirable properties for crop engineering. They could simplify the delivery and insertion of transgenes as well as provide integration sites insulated from the genetic context of an endogenous chromosome. For example, the Cre/lox recombination system was used to transfer a genetic payload from a minichromosome to an endogenous chromosome in both maize and *Brassica* (Figure 4B).^175,184^ Selection markers have also been excised from maize minichromosomes by crossing the engineered plants with Cre-recombinase expressing lines.^185^ The copy number of minichromosomes can also be altered by introducing a *trans*-acting region from native B chromosomes involved in causing nondisjunction (failure of chromosomes to separate properly during cell division), which could provide a means to vary the expression of recombinant genes they carry.^181^

#### 2.1.3 Gene Editing and Recombinant DNA Delivery to Mature Plants

Transformation methods have been developed that can engineer plant cells after the plant has grown. These methods are typically used as a research tool or to test a genetic system without having to stably engineer the germline. There are some situations where one might want to deliver genetic material to a crop after it has been planted. For example, genes conferring resistance to an insect pest could be delivered to the plant as the problem emerges. In the field, plant transformation could be performed by viruses or *Agrobacterium*. The plant genome could be permanently modified or genetic circuits used to eliminate the recombinant DNA after a proscribed period of time.

##### 2.1.3.1 Viral Delivery

DNA and RNA viruses have been engineered for the delivery of gene-editing reagents to plant cells.^186,187^ Geminiviridae is the largest known family of circular single-stranded DNA viruses in plants.^188^ Their genomes feature long and short intergenic regions that separate the regions required for replication machinery and systemic infection. Several viruses from this family were used to build geminiviral replicons (GVR) by maintaining the essential replication components and replacing the transmission components with the DNA cargo to be delivered.^189^ GVR vectors are commonly delivered into the plant cell with donor template molecules and used for gene editing through homology-directed repair. These vectors increased integration frequency up to 100-fold compared to Agrobacterium due to stronger expression of the site-specific nuclease and higher copy number of the donor template.^190,191^ They were used to make specific changes into the target sites in the genome across species, including tobacco, tomato, potato, cassava, wheat and rice.^133,191,192^ For example, WDV (Wheat Dwarf Virus) replicons were used to knock-in reporter genes in wheat and rice using Cas9.^134,136^

RNA viruses are usually smaller, with few genes required for the replication, movement, and regulation. Their best-known application is viral-induced gene silencing, an important reverse genetics tool to study gene function.^193,194^ Other applications of plant RNA viral vectors include the control of recombinant protein expression and the induction of RNAi to kill insects upon feeding.^195,196^ They are notable for their potential to facilitate gene editing in plants. Specifically, RNA viral vectors spread throughout the plant, including to germ cells.^197,198^ Edits performed in germ line cells can be transmitted directly to progeny of the infected plants, thereby eliminating the need for laborious tissue culture protocols to isolate and regenerate edited cells. Additionally, plants generally prevent viruses from being sexually transmitted, meaning the transgenic gene editing machinery would be absent in the progeny.^199^ However, RNA viral vectors are generally too small to deliver Cas9 to perform CRISPR-mediated edits.

First attempts to perform heritable gene edits using RNA viral vectors circumvented their size limitation by creating transgenic plants to express the Cas protein and using the viral vector to only express the gRNA.^200^ To this end, the Foxtail Mosaic Virus (FoMV)-derived vector was used to express the gRNA to edit plants engineered to express Cas9. Genes that were targeted encoded phytoene desaturase in tobacco, the carbonic anhydrase 2 in a grass, and a potassium transporter in maize.^201^ This strategy is limited by the inheritance of Cas9 by the editing progeny, which may later need to be removed. To overcome this limitation, the ultracompact RNA-guided TnpB nuclease (ISYmu1) was co-delivered with its guide RNA (ωRNA) to *Arabidopsis* using a single tobacco rattle virus (TRV) transcript (Figure 5B).^202^ Delivery of the TRV vector by *Agrobacterium* led to ISYmu1 targeting *AtPDS3* throughout the plant, including meristem and germline cells. Of 2318 seeds germinated from the TRV-infected plants, 41 seedlings contained biallelic mutations at *AtPDS3* and no seedlings tested had detectable TRV transcripts.

**Figure 5:**
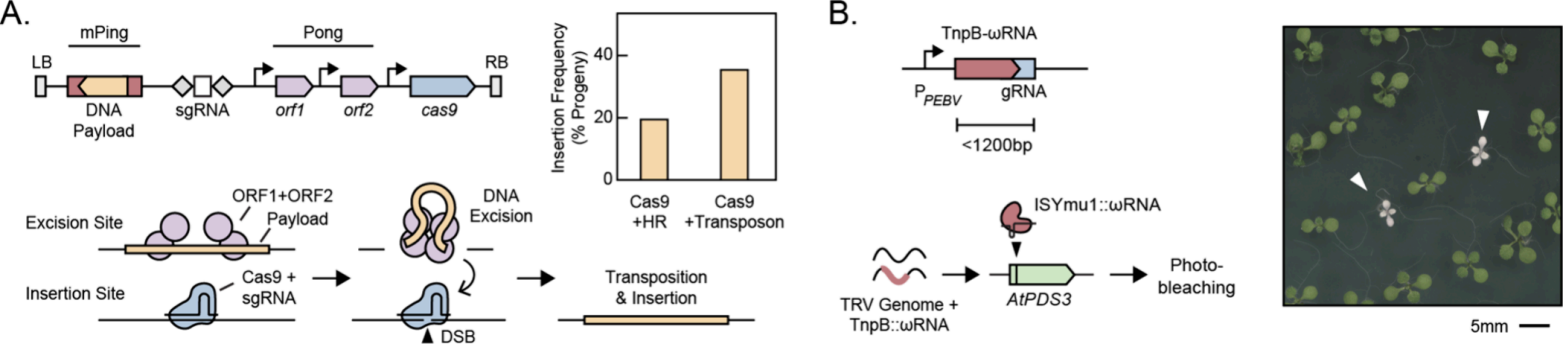
**Performing high-efficiency gene editing and insertion in plants**. **A**. Site-specific programmable targeted gene insertion in plants.^144^ The *Pong* transposase proteins, ORF1 and ORF2 (purple), facilitate the excision from a donor plasmid and insertion of their transposable element *mPing* (red) carrying DNA cargo (orange) at the site of a DSB generated by Cas9 on the plant genome. The CRISPR-associated transposase was functional when ORF2 was fused to Cas9 or expressed separately. The on-target insertion frequency of the CRISPR-associated transposase was higher than that of Cas9 and homologous recombination of a repair template (Cas9 + HR) but decreased when herbicide resistance genes were added as cargo or when ORF2 and Cas9 were expressed as a fusion protein; LB: *Agrobacterium* left border region; RB: *Agrobacterium* right border region. **B**. Heritable, markerless gene edits using a compact RNA-guided endonuclease to *Arabidopsis*.^207^ A compact nuclease (red) and guide RNA (blue) expressed from the pea early browning virus RNA-dependent RNA polymerase promoter (pPEBV) were delivered to tobacco on one of two TRV genomes via *Agrobacterium*-mediated transformation. The TnpB nuclease ISYmu1 was targeted to the *AtPDS3* gene, which inhibited carotene biosynthesis and led to photobleaching of chlorophyll (white). Image of *Arabidopsis* plants reproduced with permission from ref 202. Available under a CC-BY license. Copyright 2024, Nature Plants.

Another strategy is to use negative-sense RNA viruses, which possess a carrying capacity for larger DNA. A Barley Yellow Striate Mosaic Virus (BYSMV) vector can simultaneously deliver both gRNA and Cas9 into tobacco leaves via agroinfection.^203^ To increase the frequencies of heritable gene editing with RNA viral vectors, the Flowering Locus sequence or tRNA-like motif were added into the 3′ end of the gRNA.^198^ The motifs increase the RNA cell-to-cell mobility and therefore facilitate the modification of the germline chromosome to create heritable changes.^204^ This method was used to induce efficient multiplex heritable editing and base-editing in *Arabidopsis* expressing Cas9 or a dCas9-cytidine deaminase base editor.^205,206^

##### 2.1.3.2 Agrobacterium Delivery

*Agrobacterium*-mediated transformation harnesses the natural *Agrobacterium* infection machinery to deliver a ssDNA cargo (T-DNA) for random integration onto the plant nuclear genome. *Agrobacterium* is advantageous for its ease of engineering, high efficiency, broad host range, and ability to integrate very large cargos of >150 kb.^208^ *Agrobacterium* transformation efficiency and host range has been improved by introducing mutations to *vir* genes responsible for coordinating transformation, genes that break down negative inhibitors of *Agrobacterium*-plant interactions, or expressing effectors that suppress host immunity.^209–215^ Directed evolution of the transformation vector improved transformation through increasing plasmid copy number.^216^ To reduce concerns over the unwanted spread of recombinant DNA in the field, auxotrophy or counter selection markers have been introduced into *Agrobacterium* vectors.^217–219^ A cumic acid-inducible promoter was used to control *Agrobacterium* transformation by using it to express VirE2.^220^ Other species of non-pathogenic rhizobia are also capable of plant transformation, including *Sinorhizobium meliloti*,^221^ *Rhizobium etli*,^222^ *Ensifer adhaerens*,^223^ and *Ochrobactrum haywardense*.^224^

High expression levels of recombinant proteins are achievable using transient *Agrobacterium* transformation following a protocol of “magnifection”.^225,226^ It involves the delivery of T-DNA encoding an RNA viral vector carrying the gene-of-interest. After expression from the nuclear genome, viral proteins facilitate the amplification and cell-to-cell spread of the RNA replicon, leading to higher levels of the mRNA encoding the gene.^227^ These vectors can lead to yields exceeding 50% of total soluble protein. In the laboratory, there are several examples where *Agrobacterium* was used to deliver traits to a mature plant. In a drought-sensitive tomato line, a gene in the abscisic acid biosynthetic pathway was delivered by spraying *Agrobacterium* on leaves to transiently improve drought tolerance.^228^ Transforming orthologs of a regulator involved in flowering control using *Agrobacterium* also promoted or repressed flowering in tomato, peppers, and wheat.^228^ It has been proposed to spray *Agrobacterium* onto crops from airplanes, although doing so would invoke a high regulatory hurdle.^226^

#### 2.1.4 Editing the Plastid and Mitochondrial Genomes

Mitochondria and plastids are prokaryotic in origin and share similar genome organization and expression machinery with bacteria. As such, they have potential for expressing bacterial pathways that would be incompatible with nuclear expression due to differences in genetic organization and regulation.^229^ They also have enzyme co-factors, such as metal clusters, that are not present elsewhere in the cell; contain higher concentrations of some core metabolites; and have high (chloroplast) or low (mitochondria) oxygen tensions, which are desirable for the activities of some recombinant enzymes.^230^

##### 2.1.4.1 Plastid Genome Engineering

Plant leaves can have up to 100 chloroplasts per cell, each containing 50 copies of the plastid genome.^231^ The genome is relatively small, encoding about 100 genes, and can range from 19 to 217 kb across species, implying that it can carry more genes without over-burdening the cell.^232^ Very high levels of recombinant gene expression, more than 70% of the plant’s total soluble protein, can be achieved when it is expressed from the plastid genome.^233^ An additional benefit it that the maternal inheritance of the plastid genome mitigates environmental dispersion of recombinant genes through pollen. The plastid also lacks RNAi machinery, making it possible to express double-stranded RNA (dsRNA), which has been used to confer insect resistance.^234^ Non-green plastids, such as carotenoid-rich chromoplasts and starch-accumulating amyloplasts, do not perform photosynthesis and are less sensitive to recombinant protein accumulation, and thus may be more suitable for metabolic engineering.^235^

Despite their advantages, the difficulty of engineering plastids currently limits the scope of their manipulation for agriculture.^236^ Plastid genome engineering has only been successful in a limited number of agriculturally-relevant crops: tobacco, potato, tomato, lettuce, soybean, rapeseed, carrot, cabbage, sugar cane, sugar beet, eggplant, and cauliflower.^237–245^ A major bottleneck is the need to reach homoplasy, in which all plastids, and all genomes within each plastid, contain the recombinant gene.^246^ Getting to this point requires continuously propagating transplastomic lines under selection pressure using laborious and time-consuming tissue culture, selection, and regeneration procedures. Not all lines within a crop species may be amenable to this process. For example, plastid engineering in *Arabidopsis* can only be performed in transformation-competent lines deficient in a nuclear-encoded fatty acid biosynthesis enzyme, which sensitizes *Arabidopsis* to the selection agent spectinomycin by making cell proliferation dependent on translation of the plastid-encoded *accD* gene.^242,247^ Transplastomic rice has been reported, but the plants were not homoplasmic.^248,249^

Major cereal/monocot crops are recalcitrant to plastid transformation primarily due to the lack of sensitivity to the selection agent spectinomycin.^249^ In crops amenable to plastid transformation, spectinomycin naturally inhibits translation in the plastid, resulting in bleaching during tissue regeneration and arresting cellular growth. In these plants, selection is achieved through the recombinant expression of the bacterial spectinomycin resistance aminoglycoside 3′-adenyl transferase (*aadA*) gene.^250^ Another problem is that recombinant gene expression in the chloroplast often imparts a negative impact on plants by interfering with photosynthesis.^233,251^ Non-green plastids, such as chromoplasts in tomato fruits and amyloplasts in potato tubers, provide a less-explored location for carrying a recombinant function as expression in these organelles does not impact photosynthesis.

Chloroplast genome editing is possible through homologous recombination, as used in bacterial engineering, where sequence overlap between the payload and plastid directs integration to a specific site. While any genome position can be edited using homologous recombination, a common set of integration sites have been chosen for their high expression and limited impact on endogenous gene expression.^252^ CRISPR/Cas9 has been used for gene insertion in *Chlamydomonas reinhardtii* plastid genomes. However, there are not similar capabilities in higher plants, where the transcription and processing of gRNAs has proven difficult.^253^

To edit native genes and avoid the introduction of foreign selection markers, base editors have been developed to target the chloroplast genome. A cytidine deaminase domain (DddA) was fused to a TALE DNA-binding domain to carry out C-to-T conversions in the plastids of rice, *Arabidopsis*, lettuce, and rapeseed.^254–256^ Fusion of the DddA with the TadA deoxyadenosine deaminase derived from *Escherichia coli* induced targeted A-to-G editing in human mitochondria and *Arabidopsis* plastids.^257,258^ Homoplasmic base editing was accomplished by applying a selection pressure for specific mutations, such as for edits that confer resistance to a chemical-based selection.^256^ The strong expression of base editors during early embryonic growth in *Arabidopsis* led to near-homoplasmic editing (>99%) at certain nucleotide positions.^258^

Recombinant genes carried on episomally-replicating plasmids or minicircular chromosomes offer an alternative to genome editing that leaves the native plastid genome untouched. Circular vectors containing a short sequence called the tobacco native extrachromosomal element (*NICE1*) can be maintained extrachromosomally even without a chloroplast origin of replication.^259–261^ Another approach is to use the replication origin from marine dinoflagellate chloroplast genomes, which are composed of multiple small minicircles with reduced size (2-3 kb).^262,263^ Transformation of these mini-synplastomes into potato plastids demonstrated they could persist across multiple generations without antibiotic selection.^263^ Finally, ssDNA (single-stranded DNA) geminiviruses replicate by rolling circle amplification using host replication machinery, but require a replication initiation protein (Rep) to initiate replication at a viral origin of replication (VOR) (Figure 4C). DNA vectors containing VOR elements from beet curly top geminivirus (BCTV) were replicated as minichromosomes in tobacco chloroplasts when the Rep protein was either expressed in the nucleus and targeted to the chloroplast or expressed from the chloroplast genome.^183^ However, the maintenance of self-replicating plasmids when growing plants in the soil without antibiotic selection and their inheritance by the next generation via seeds remain challenging.

##### 2.1.4.2 Mitochondrial Genome Engineering

Mitochondria, the “powerhouse of the cell,” also contain independent genomes that evolved from prokaryotes. While mitochondria have been genetically engineered in other eukaryotes (yeast, algae, and mammalian cells^264–269^ ), plant mitochondria have only been transformed after isolation.^270,271^ Their small size and lack of selectable marker genes have been obstacles for the transformation of plant mitochondria. Instead of directly transforming mitochondria, gene editing nucleases can be expressed from the nuclear genome and directed into the mitochondria with signaling peptides.^272,273^ Nucleus-encoded TALENs fused to these mitochondrial signaling peptides have been used to knock out mitochondrial genes in rice and *Brassica* to cure cytoplasmic male sterility.^273^ With the same approach, *atp6-1* and *atp6-2*, encoding ATP synthase subunit 6 was also deleted in the *Arabidopsis* mitochondrial genome.^274^ In addition, the DddA-derived cytosine base editor has been targeted into the mitochondria of lettuce and rapeseed through protoplast transformation to edit mitochondrial genes.^256^ Pairs of TALENs were targeted to the tobacco mitochondrion to mutate a non-essential gene and homochondriomic mutants resulting from TALEN activity were isolated.^275^

#### 2.1.5 Toward De Novo Synthesis of Complete Plant Genomes

Entire genomes have been constructed using chemical DNA synthesis and used to replace the native genome in living cells. The sizes and complexities of the synthesized genomes have been growing: 5.4 kb phage (2003),^276^ 1.1 Mb bacteria (2010),^232^ and 12 Mb chromosomes of yeast (2022).^277,278^ Mammalian chromosomes are likely next and it seems inevitable that plant genomes will follow, possibly starting with whole plastid synthesis and simpler artificial chromosomes.^234^ Complete genome synthesis, where every base pair is specified and built using chemistry and molecular biology, offers the ability to radically redesign the genome. For example, genes can be reorganized to simplify engineering efforts, non-essential genes and regions can be removed (retroviruses, transposons, insertion elements), and proteins can be recoded to repurpose certain codons to incorporate non-natural amino acids or to prevent gene transfer and viral infection.^279–284^ These changes would simplify future modeling and engineering efforts.

Plastid genomes are relatively small, approximately 100 kb, making them approachable targets for *de novo* synthesis.^285^ Successful transformation of a synthetic plastid has only been achieved in *Chlamydomonas reinhardtii*, a single cell alga with a single chloroplast, with the intent of making multiple alterations to its photosynthetic apparatus.^286^ The entire 203 kb plastid genome, which includes several photosynthetic genes from red algae, was cloned, assembled in yeast, and transformed into the *C. reinhardtii* chloroplast, where it recombined with the native plastid genome. A tobacco minimal plastid genome, with all photosynthesis-related and other non-essential genes removed, has been designed, but not yet built or transformed.^287^ The mouse mitochondrion genome has been synthesized, but one from plants has not, and its replacement remains technically very challenging.^288^

Minimal plant genomes could be built through the iterative deletion of nonessential genes. This capability would aid the functional analysis of gene clusters and noncoding genetic elements and generate new agronomic traits through deletion of unwanted loci. The first minimal cell, the bacterium *Mycoplasma mycoides* JCVI-Syn3.0, has a 531 kb genome containing only 438 protein-coding genes and 35 RNA-coding genes was created by deleting about half of the parental genome.^232,283^ Viable minimal *Escherichia coli* and *Bacillus subtilis* strains have been constructed by removing about a third of their genomes (Section 3.2.1).^289–291^ One method to delete large regions of the chromosomes uses two pairs of nucleases (ZFNs, TALENs, or Cas9) to generate two double strand breaks that are then ligated via repair pathways. This method removed up to a Mb from the genomes of prokaryotes and mammalian cells (up to 65 Mb from neuroblastoma cell lines).^292^ This strategy was applied to rice by deleting two nonessential 170 and 245 kb regions that encode genes involved in diterpenoid synthesis.^293^ Another method is based on the repurposing of a prime editor to delete large chromosomal regions, by combining a pair of pegRNAs and the Cas9 nickase or nuclease.^294^ This method can precisely delete up to 10 kb in mammalian cell lines but have not yet been applied to plants.

Synthesizing complete plant chromosomes and replacing the native genomes of cells will be difficult due to their size and complex organization. Even in simpler model eukaryotes, the synthesis of entire chromosomes has taken years and a large multi-institutional international collaboration. The Synthetic Yeast Genome Project (Sc2.0) is an ongoing project to completely synthesize and assemble 16 yeast (*Saccharomyces cerevisiae*) chromosomes (12 Mb).^277,295^ While they have been synthesized individually, only 6.5 have been consolidated into a single strain to date.^278^ The assembly strategy involved the iterative replacement of 30-60 kb fragments assigned with one of two auxotrophic markers. Sc2.0’s genome is 8% smaller, does not contain retrotransposons, repeated sequences, redundant and nonessential genes, or many introns (pre-mRNA introns and all tRNA introns).^277^ Further, the tRNA genes were relocated to a specialized new chromosome encoding only tRNA.^296^ In a particularly radical re-engineering project, all *Saccharomyces cerevisae* chromosomes were fused to create a single 11.8 Mb chromosome, which surprisingly resulted in viable cells.^297^ When whole genome construction and simplification is possible in plants, the impact would be large. One can imagine massive genome reorganization to facilitate the insertion of large multi-gene designs, as well as improved designability, evolutionary stability, and viral resistance through genome-scale modifications (*e.g.*, changing codon usage across all genes).^6,284,298^

### 2.2 Plant Genetic System Design

Abstraction is a core principle of synthetic biology intended to aid complex design projects.^299^ Following this paradigm, genetic parts are the most basic units of DNA; they encompass a minimal function, such as a promoter. Large libraries of reliable genetic parts aid in precisely tuning expression levels, for example to balance flux through a metabolic pathway or facilitate the construction of large designs. Genetic devices are assemblies of parts that collectively perform a function, such as a sensor or metabolic pathway. Devices are assembled into a system, which encompasses the complete recombinant design introduced to the cell. Plant genetic engineering projects have not necessitated these abstractions as they have been relatively simple, with few parts and random integration, but as they get more complex and reliable over time, this principle will become more important in organizing designs.

This section describes libraries of genetic parts for plants. The parts are organized into those involved in transcriptional and translational processes, including insulators that isolate a synthetic construct from genetic context. In the simplest form, they can be used to maximize the expression of a protein in a plant; up to 5 g/kg biomass has been reported.^300^ These genetic engineering projects are simple enough where they can be organized “by hand.” However, more complex metabolic engineering and regulatory control requires combining many parts. As projects get larger, it will become more important to use CAD software, which has already become routine for simpler organisms. Computational tools for plant genetic part design are described in Section 2.5.1.1. It is expected that these tools will be needed as plant engineering efforts get more complex and require the balancing of many design constraints.

#### 2.2.1 Plant Genetic Parts

Compared to simpler organisms, defining a genetic part for a plant can be more complex. A part should be discrete, meaning it has a clear nucleotide beginning and end, and ideally has a single function, irrespective of genetic context. These are often not the case for natural regulatory parts in plants; for example, promoters can have enhancers many kilobases upstream, functions can overlap, and chromosomal effects and silencing are often position-dependent. Most of plant genetics will not conform to this idealized abstraction, but that is okay: the point of the exercise is to simplify and collate synthetic genetics to facilitate engineering and not to be descriptive of natural genetics. Further, part classes can be engineered to be modular and tunable by insulating against complicating phenomena that occur during transcription and translation.^301–303^ As such, it is valuable to build part libraries, develop methods to minimize context effects, and maintain them in public databases to facilitate widespread use. A notable example of this ongoing effort for plant engineering is OpenPlant (https://www.openplant.org/).^304^ GenoCAD and the GoldenBraid webdesign tool (GB3.0) manage the process of part selection, storage of characterization data, and the implement of assembly rules in creating plant expression cassettes.^305,306^

One reason that plant part libraries have been slow to develop compared to other organisms is due to the historical limitations of genome modifications. Because of the random nature of genome insertions, a single gene with one promoter and terminator could be transformed and it would insert into many chromosomal locations. These different locations and copy numbers lead to different levels of expression. The after-the-fact selection of a plant that has the desired phenotype is effectively a directed evolution experiment.^307,308^ However, genome editing facilitates single, defined integrations and landing pads improve reliability; albeit, not yet in most agriculturally-relevant crops. Replacing this process can actually be problematic, where the earlier random approach allowed for one to test many expression levels with one part (*e.g.*, a promoter) whereas a defined insertion site means that different parts must be screened. To this end, libraries of genetic parts can be collated by using bioinformatics to scan databases for lists of natural sequences (“part mining”), computational design, or randomizing a DNA scaffold and screening.^303,309–311^

Part characterization is also harder in plants because high-throughput experiments are difficult, there is variability in expression due to cell and tissue type, and chlorophyll interferes with the fluorescent signal from common reporters of expression.^312,313^ Part characterization has relied heavily on Agroinfiltration methods, where constructs are delivered by *Agrobacterium* and expressed transiently at high-copy number in the leaves of tobacco or other plants (Section 2.1.3). Agroinfiltration is preferred for being fast and scalable and it is feasible to characterize thousands of parts on the order of days. Multiple constructs can also be co-infiltrated, which enables the combinatorial characterization of parts such as promoters and transcription factors (TFs).^314^ Nevertheless, agroinfiltration has limitations, including heterogeneous results arising from inconsistent infiltration efficiency, variability in gene delivery, and expression requiring careful normalization, and potential gene silencing mechanisms in the plant. It also does not permit testing in the tissue of interest (*e.g.*, roots) or the plant species of interest. To reduce variability when evaluating the transcriptional activity of plant parts, a ratiometric dual *lux* system was developed that uses a second constitutive luciferase gene as an internal reference for normalization.^315^

The cost and time required to genetically engineer plants make it difficult to build hundreds of plant lines just to characterize a library of parts (*e.g.*, a set of constitutive promoters of different strengths). The slow throughput is in contrast to bacteria and yeasts, especially model species. For example, large part libraries have been screened using multiplexed DNA construction and high-throughput characterization strategies. An example is flow-seq, where a cell sorter is used to isolate parts from a large library by their activity, followed by deep sequencing to determine the parts.^309,316^ This approach was used to characterize over 12,000 *Escherichia coli* promoters and ribosome binding sites (RBSs) in a single experiment.

While these methods are not suitable for screening whole plants, protoplasts provide a promising way to scale-up the throughput and have been used to characterize over 100 synthetic promoters in *Arabidopsis* and sorghum.^317^ Protoplasts are individualized plant cells lacking a cell wall that are generated from a variety of plant tissues through enzymatic or mechanical means.^318^ Once isolated, protoplasts can be transformed by electroporation or PEG-mediated techniques and assayed either as individual protoplasts or undergo regeneration into entire plants. Protoplasts can also be sorted using fluorescence-activated cell sorting (FACS), although to our knowledge, this has not yet been applied to *in planta* part discovery.^319,320^ An alternative to protoplasts is cell culture suspensions, such as the tobacco BY-2 cell line, which are fast growing and easy to transform. A plate-based fluorescence screen of 19 plant promoters and 20 promoter-terminator combinations was performed using Tobacco BY-2 cell suspensions co-pelleted with *Agrobacterium* cultures.^321^

##### 2.2.1.1 Transcriptional Parts

In plants, transcription is regulated by many proteins that alter chromatin accessibility to recruit RNA polymerase II (RNAPII), control its production of a transcript, and determine when it dissociates from the DNA (Figure 6A).^332^ It is desirable to make the DNA sequences controlling these processes as modular as possible so that they can be easily combined in new ways. A complication is that natural eukaryotic transcription, including in plants, often is controlled by large and complex DNA regions that contain many poorly-defined motifs. This organization has made it historically difficult to make even simple parts; for example, a “constitutive” native promoter can change expression based on the tissue type and developmental stage. It also makes it more difficult to be quantitative in combining parts to achieve a precise level of expression. In bacteria, promoter strength has been defined as the RNAP flux emanating from it, which has enabled model-guided genetic circuit design.^333^ Using this paradigm to characterize a eukaryotic promoter is more complicated because of the impact of chromosome accessibility, promoter-terminator looping, pervasive transcription, and interactions with the nuclear pore complex.^147^ There are also many additional steps after mRNA transcription that can alter expression levels and complicate the assignment of a single strength to a promoter (5′-capping, export, splicing, 3′-polyadenylation, etc.).^334^ This section outlines the definition of transcription control motifs as modular parts that can be used to control transcription.

**Figure 6:**
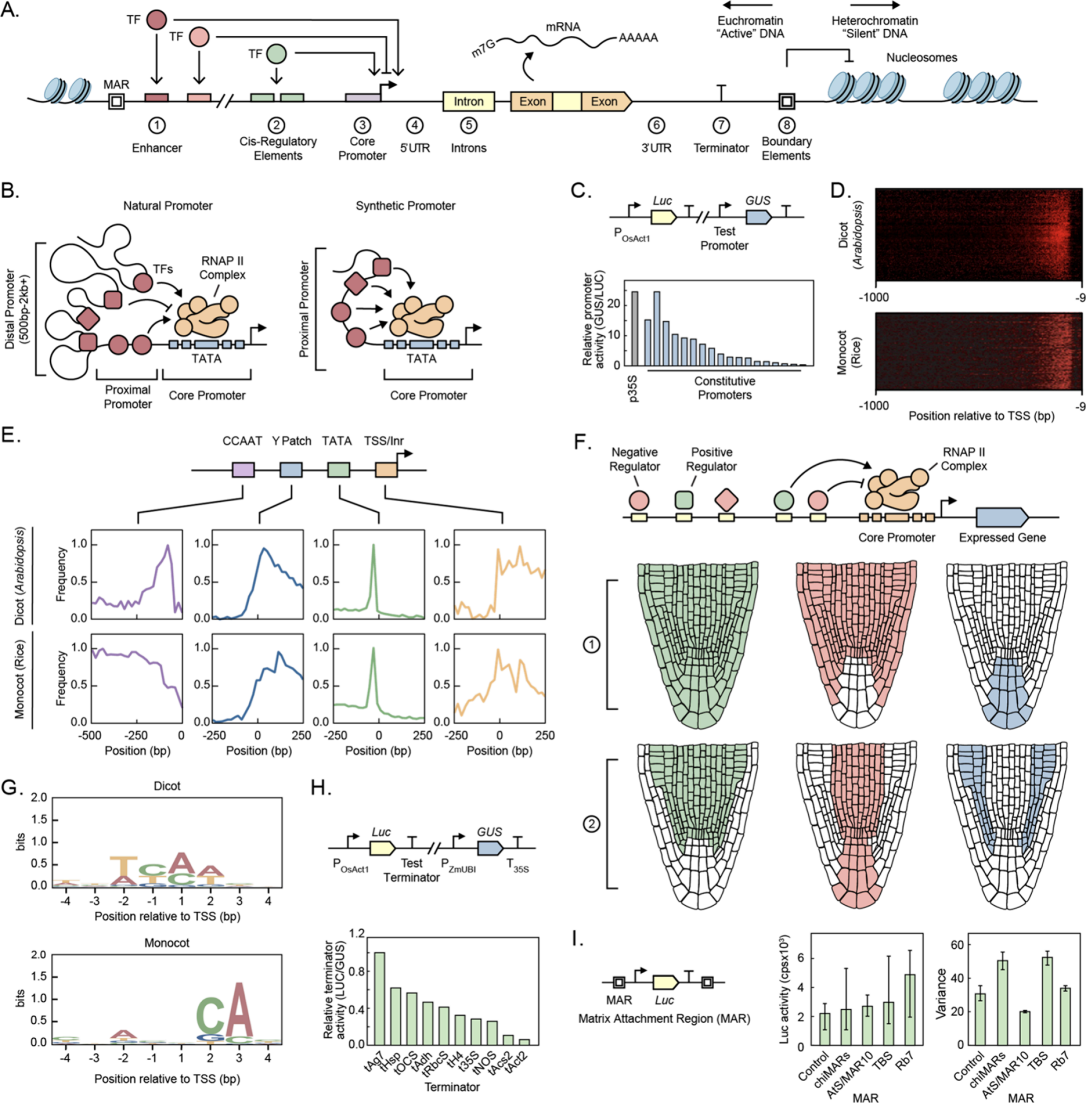
**Genetic parts characterized in plants**. **A**. Layout of genetic parts that control the expression of a plant gene. See the section text for part definitions: 1-5. Transcriptional Parts (Section 2.2.1.1), 4-6. Translational Parts (Section 2.2.1.2), 7-8. Insulators (Section 2.2.1.3). **B**. Schematic of natural and synthetic promoter architecture in regard to TF binding motifs.^322^ Natural plant promoters include a core region (blue) encompassing many core promoter elements (Y patch, CCAAT element, Inr element, CA element, and TATA-box). Binding and assembly of transcription machinery (yellow) to the core promoter are regulated by diverse TFs, which bind to CREs in a proximal region and a distal region located 2kb+ away from the TSS. Synthetic promoters were simplified to include fewer core promoter elements and user-defined CREs near the core promoter to achieve more desirable promoter properties. **C**. A library of promoters was characterized in *Nicotiana benthamiana* leaves.^323^ Strengths were reported relative to the rice actin promoter (pOsAct1). **D**. Location of upstream CREs in dicots^324^ and monocots.^325^ CRE motifs (red pixels) were predicted across many promoters (pixel rows). Maps of dicot and monocot CREs reproduced with permission from refs 324 and 325 , respectively. Map of dicot CREs^324^ available under a CC BY license. Copyright 2007, BMC Genomics and Copyright 2007, Nucleic Acids Research. **E**. Location of various core promoter elements in dicot (*Arabopdsis*) and monocot (rice) promoters.^326,327^ The frequency of predicted core promoter elements was determined by calculating the proportion of sequences containing each element within a 20-bp sliding window, normalized to the total number of analyzed sequences. Element positions are shown relative to the transcription start site (TSS). Figure adapted from ref 327. **F**. Generating tissue specific expression using transcriptional regulation.^328^ Tissue-specific expression of TFs determines gene expression in specific cell types; positive regulators (green) promote transcription, but the effect can be negated by the presence of negative regulators (red). **G**. TSS motifs.^329^ The web logo was generated from nucleotide frequencies calculated using 217 dicot and 70 monocot promoters. **H**. Terminator efficiency in barley roots.^330^ Strengths were quantified as the expression ratio of an upstream reporter (firefly luciferase; *luc*) and a downstream reporter (β-glucuronidase; *gus*). **I**. Impact of MARs on recombinant gene expression in Arabidopsis.^331^ MARs were shown to increase the expression of luciferase. The variability in luciferase activity between independent insertion events was also quantified to assay the MARs’ ability to insulate expression from genetic context. It was shown that the AtS.MAR10 sequence could reduce variability between separate, stable insertion events likely due to its ability to insulate the gene from epigenetic regulation from neighboring sequences.

###### 2.2.1.1.1 Plant Promoters

This section describes design of promoters for the production of coding mRNA to express proteins in plants. The minimal core promoter is the DNA sequence that lies directly upstream of the transcription start site (TSS) to which the pre-initiation complex, including RNAPII, binds.^335^ The core promoter typically contains sequence motifs that bind to different parts of the complex. Upstream of the core promoter, *cis*-regulatory elements (CREs) bind to TFs (Figure 6B) (TFs).^336^ CREs can make a promoter responsive to additional signals, including tissue-specific TFs (Section 2.3.3.a) or those involved in environmental stress (Section 4.1). Enhancers even further upstream alter the chromatin structure to change the probability that a promoter is on without increasing the expression level.^300^ Plant promoters can be large when the CRE and enhancer sequences are considered; rescuing knockouts can require the inclusion of 4-10 kb of promoter DNA.^337^ The binding of TFs to the CREs and enhancer region leads to looping and protein-protein interactions with the RNAPII complex (Figure 6B).^338^

Natural promoters sometimes appear modular, where the various motifs can be mixed-and-matched and sometimes they exhibit non-modularity. Deep learning algorithms have implied that the entire sequence of the promoter, and even the full transcription unit, is required to predict expression.^339^ However, promoters with very different arrangements of motifs can yield the same expression patterns, indicating a degree of modularity.^322^ This modularity can be used to design synthetic promoters (Section 2.2.1.1.2).

Natural plant promoters are often used for genetic engineering projects. The PlantProm database contains 576 native plant promoters with experimentally-verified TSSs across 86 species including rice, maize, soybean, grape, *Medicago*, and *Arabidopsis*.^329^ The largest collection of experimentally-validated natural plant promoters is Plant Proteome DataBase (ppdb) with promoter positioning for over 27,000 genes (as of 2021) for *Arabidopsis*, as well as rice, *Physcomitrella patens*, and poplar.^340^ Computational tools, such as TSSPlant, can be used to predict novel plant promoters using data from these large datasets.^341^

Natural plant promoters can be identified using microarrays or RNA-seq.^342,343^ Depending on how the data are processed, promoters can be identified that are constitutive, tissue-specific, or inducible. Using this approach, a library of 15 natural promoters was identified that spanned a 100-fold dynamic range.^344^ The strongest promoters in this library were similar in strength to commonly-used viral promoters (CaMV35S, see below). They showed the same rank order of activity when experimentally validated with a reporter in *Nicotiana benthamiana* (tobacco) and *Lactuca sativa* (lettuce). Other libraries of constitutive promoters have also be identified and characterized in tobacco, producing over a 300-fold range in expression.^345^

Most plant engineering projects on a few constitutive promoters to obtain high levels of expression. Because plant promoters are so large and complex with unknown regulatory inputs, these promoters tend to be sourced from viruses or bacteria (*e.g.*, Agrobacterium).^346^ As part of their virulence programs, they insert DNA into the plant genome containing a promoter controlling a gene encoding a virulence factor.

A notable example is the 343 bp Cauliflower Mosaic Virus 35S promoter (referred to as CaMV35S or 35S). Due to the fact that the 35S promoter provides high expression and has a very wide host range, it has become the *de facto* standard to which other plant promoters are compared.^347^ The 46bp CaMV35S minimal promoter and its variations can be found in over 60% of all transgenic crops grown worldwide.^347^ While the 35S promoter is considered constitutive, its activity has been shown to be dependent on tissue type and the plant’s physiological state. In *Arabidopsis*, CaMV35S-driven expression was up to 18-fold higher in root tissue than in stem tissue and up to 14-fold higher following heat stress.^300,348^ Interpreting results from the literature based on this promoter can be challenging as there is no standard and the same promoter name can be used to describe different DNA sequences.

Hybrid promoters have been built that vary the sequence of CaMV35S and related viral promoters. When then have about the same activity, they could be used together in a construct to avoid homologous recombination and silencing. Stronger promoters have also been identified, producing up to 5-fold increased expression over the already-strong CaMV35S.^349–354^

Other commonly-used promoters have been derived from plants or viruses that infect plants and can be tissue-specific (below) or stress-inducible (Section 4.1).^355^ A few of these promoters are as strong as the CaMV35S promoter. They had similar strengths when tested in *Brassica rapa* and *Nicotiana benthamiana*, indicating that at least a subset of plant genetic parts can be reliably used across species.^356^ However, there is ample evidence that promoters do not generate the same expression levels in different species. After comparing the strengths of 46 promoters across tobacco leaves, *Medicago truncatula* roots, *Lotus japonicus*, and barley, a core set of only seven was identified that showed functionality across the species and tissues tested (Figure 6C).^330^ Note that in genetic engineering, this core set could then be the focus and the misbehaving species-specific promoters removed from the design set. A high-throughput analysis of 8,000 *Arabidopsis*, 34,000 maize and 27,000 sorghum core promoters was performed by measuring transcript abundance in pooled libraries.^357^ The promoter strengths were only weakly correlated when expressed in tobacco leaves compared to maize protoplasts. The species variability of promoters has been reduced using principles from control theory, such as feedforward loops, but this strategy has not yet been applied to part design for plants.^358,359^

###### 2.2.1.1.2 Synthetic Promoter Design Principles

Synthetic promoters have been built from the ground-up using only well-defined sequence motifs. They have a number of advantages over using a natural promoter in a project. First, they are more specific because they only recruit the defined TFs and do not have unknown regulatory factors that are usually present in natural promoters.^322^ Thus, they generate less leaky expression from uncharacterized native TF binding.^337^ It also reduces their crosstalk with endogenous regulatory networks and synthetic genetic circuits consisting of multiple TFs.^360^ They also do not have unnecessary negative feedback and less metabolic/energy burden.^360^ In this section, the rules for building a core promoter are described, with the addition of upstream CREs in Section 2.2.1.1.7. Distal promoter regions containing enhancers are typically not part of synthetic promoters, but they may be used as insulators (Section 2.2.1.3).

The core promoter is defined as a region that has to be placed about ±50 bp around the TSS for transcription to initiate.^322,360^ More generally, the region from about -250 to the TSS has a strong effect on transcriptional activity (Figure 6E).^360,361^ Within this region, there are motifs that interact directly with the RNAPII and associated proteins (the transcription complex). In addition, the nucleotide composition impact activity.^357^ This effect is plant specific, with AT-rich promoters in tobacco leading to 4-fold higher activity whereas there was no impact on maize promoters. Note that the list below provides the approximate sequences and locations of these motifs; differences have been observed between species and across monocots and dicots.

- *TSS*.^325,327^ There is usually a single C or T upstream of the TSS and a single A or G downstream.
- *TATA box* (TATAWAW) with a consensus of TATAAA).^360^ This is the most important motif required for high expression.^327^ It interacts directly with the RNAPII transcriptional complex and is usually positioned 30 – 40 bp upstream of the TSS.^337,360^ The precise flexibility in the location of the TATA box is variable across plant species.^357^ For example, in Arabidopsis there is a single peak at about -30 whereas in maize, there is a second peak that also occurs at -50. Replacement of any of the A/T’s in the TATA box with a G results in sharply decreased activity.^357^
- *Downstream promoter element (DPE)* (RGWCGTG).^327^ Promoters that do not have TATA boxes can have a DPE instead. It has a similar function as the TATA box in recruiting RNAPII. They are typically located +30 from the TSS.
- *Inr (initiation) element* (PTCANTPP where the A is the +1 first bp transcribed).^322,327,357^ RNAPII cooperatively binds to the Inr and either the DPE or TATA box.^327^
- *Y patch* (CYTCYYCCYC).^322,325,327,362^ It is variable in its position from about -150 to -1.^327^ It is a common motif that is unique to plants: 18% of Arabidopsis and 50% of rice promoters contain Y patches.^327,362,363^ Promoters with Y patches are 15% stronger.^357^
- *GC box* (GRGCGS).^326^ It recruits TFs and typically is found about 100-300 bp upstream, but can occur much further away from the core. Multiple GC boxes often occur in a promoter.
- *CCAAT box*.^322,360^ It interacts with the RNAPII machinery by recruiting the nuclear TF-Y.^327^ Its binding to this position causes changes to the histones that can enhance or suppress expression. It is positioned -120 to -40 in dicots and -460 to -140 bp in monocots.^327^

These sequence motifs have been observed in the core, but they are not all required for activity. In fact, some natural promoters do not have any of them and yet transcription occurs.^357^ But adding more motifs increases the promoter strength.^327^ This effect was apparent when analyzing completely random nucleotide sequences that do not act as plant promoters. Placing motifs into these otherwise inert sequences was sufficient to turn them into promoters. The TATA box was the most important, followed by the Y patch, and then the Inr sequence. This effect has been shown in Arabidopsis, maize, and sorghum. Obtaining synergy between motifs requires their proper spacing as they can also interfere with each other.^322,364^ Promoter activity exhibits a sharp decrease if there is more than 50 bp between the first upstream motif and the TATA box.^356^

Promoter design would benefit from mathematical models with interpretable biophysical contributions of each motif and TF to the promoter activity. The closest to this ideal has been the application of ML to large datasets of natural transcriptomic data to extract the contributions of sequences to promoter activity.^360^ ML results can be difficult to interpret and thus make informed choices regarding the rules underlying promoter activity. However, it is still possible to apply these models to design improved promoters through a process of computational mutations and evaluation.^357^ By screening different upstream CREs and downstream minimal plant promoters, libraries consisting of hundreds to thousands of unique constitutive synthetic promoters with varying strengths have been created for *Arabidopsis*.^356,365,366^ Using these data, novel synthetic promoters were computationally designed, and their strength was predicted by comparing their sequences to an experimentally-characterized set of promoters.^356^

Libraries of promoters that produce different levels of gene expression are very useful for different applications of genetic engineering. Metabolic flux could be controlled through a pathway by systematically varying the expression levels of enzymes.^345^ Not allowing a toxic intermediate to accumulate in the cell reduces the growth impact. Even promoters of the same strength, but different sequences, can be helpful by allowing traits to be expressed consisting of multiple genes. The sequence variability avoids problems due to homologous recombination^300,367,368^ and silencing.^300^ These problems can be addressed by using promoters that have the same strength, but vary in sequence composition. One approach is to vary the sequences of the motifs or regions between them or sourcing different organisms, including from outside plants (*e.g.*, yeast).^360^ Constitutive promoters also provide the backbones for inserting CREs that allow for inducible- or condition-specific promoters to be built.

###### 2.2.1.1.3 Adding Cis-Regulatory Elements (CREs) to Control Expression Conditions

Regulatory inputs can be added to a promoter by including upstream CREs. Natural plant CREs are associated with heat shock, light, development, wounding, pathogen attack, sugar sensing, reactive oxygen species, and cold stress and combinations of them can be seen upstream of the TSS (Figure 7).^369–377^ Adding CREs to a synthetic promoter can be used to create a sensor or inducible system (Section 2.3.4). Regulatable promoters can be specific to a particular cell type, under a defined set of conditions, or to keep the expression of a toxic protein off until an appropriate growth stage is reached.

**Figure 7:**
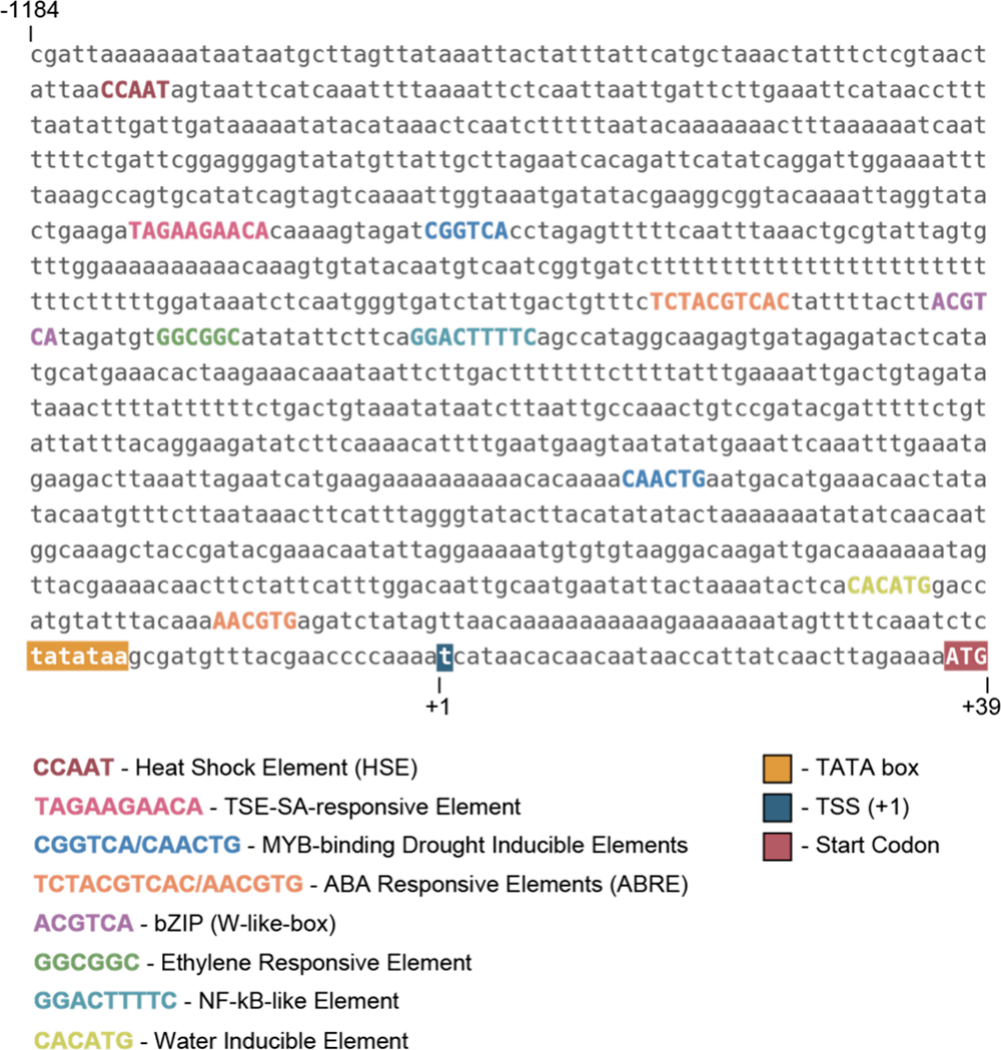
**CRE placement in a natural *Arabidopsi* *s*** **PR-1 plant promoter**. Stress-responsive gene expression is coordinated by many CREs found upstream of the gene (ATG start). Figure provided courtesy of Neal Stewart Jr.

CREs responsive to specific stimuli can be found in databases of published sequences, such as PLACE,^378^ TRANSFAC,^379^ or PlantCARE.^380^ Their activities are modular and the incorporation of these CREs into a synthetic promoter makes it responsive to different stimuli.^338^ However, some caution needs to be taken as CREs can have differently, sometimes opposite, functions in different species.^328^ For example, a motif was observed to be root-specific in tobacco but then was green tissue specific in rice.^381^ Table 1 provides examples of promoters whose CREs allow them to only turn on in a particular tissue type. In Section 4.1, Table 5 provides promoters whose cis-motifs cause them to respond to stresses.

**Table 1:**
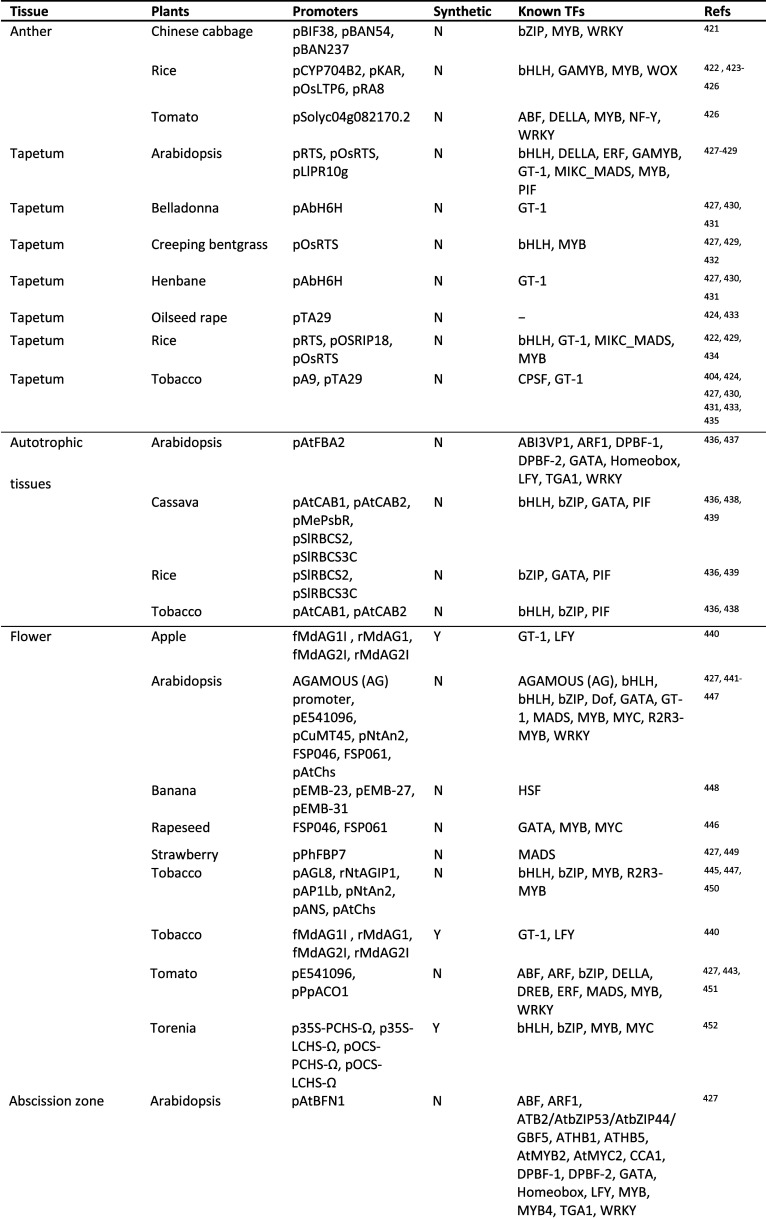

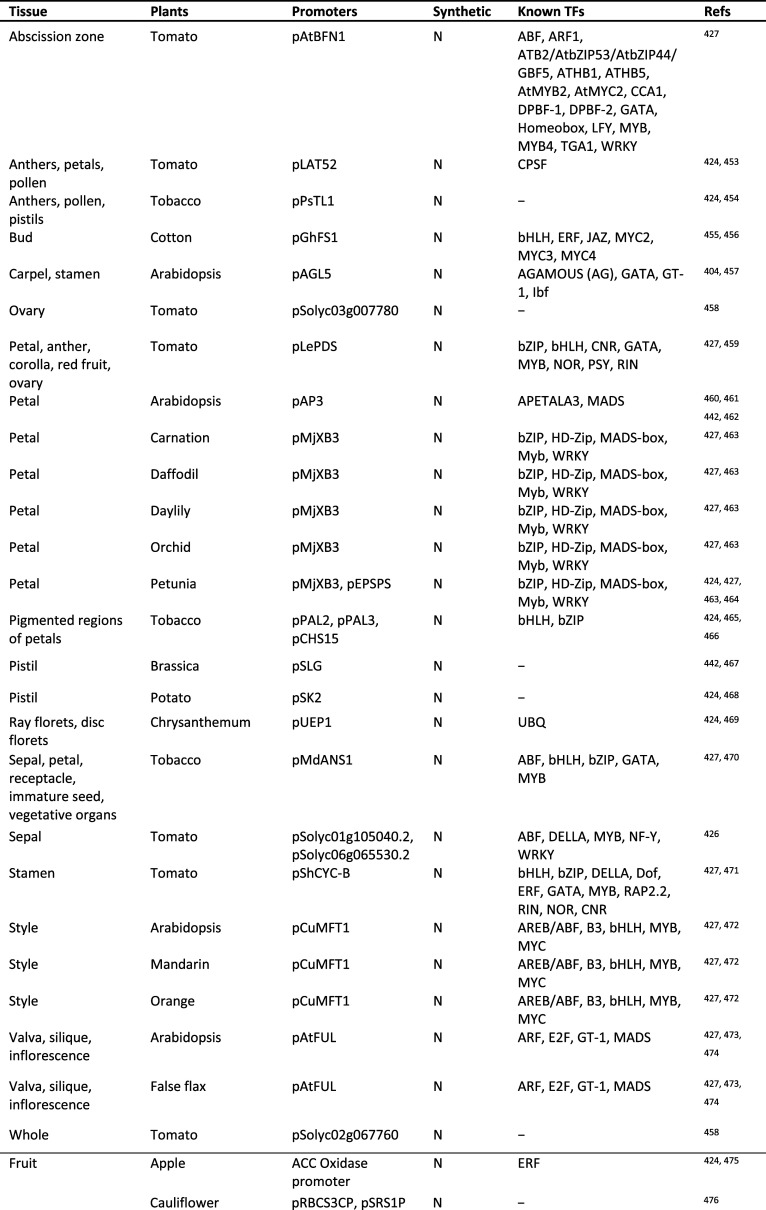

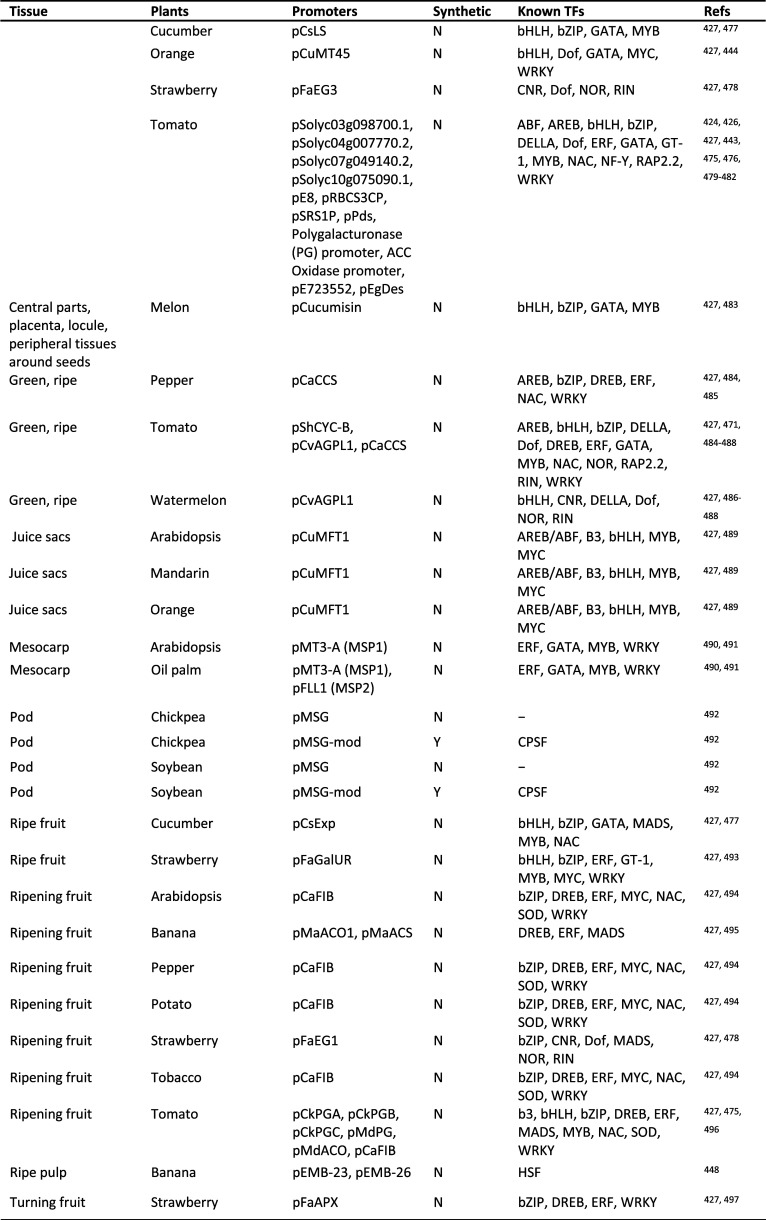

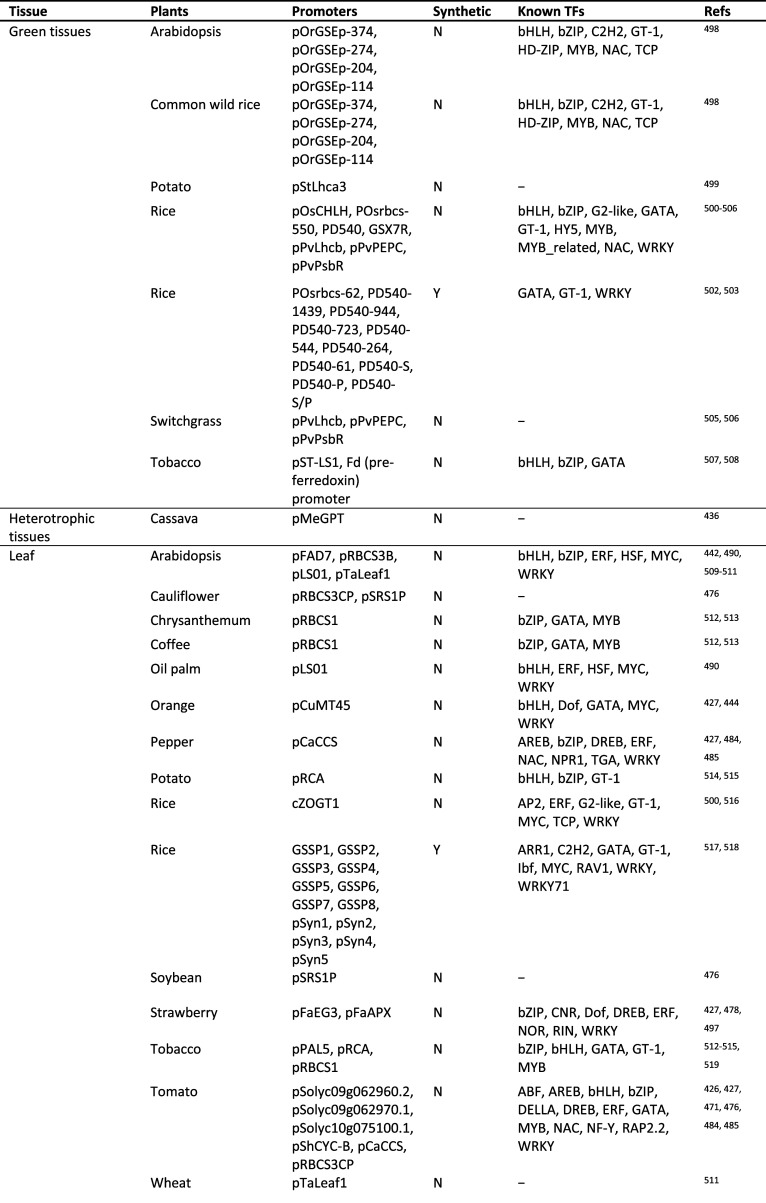

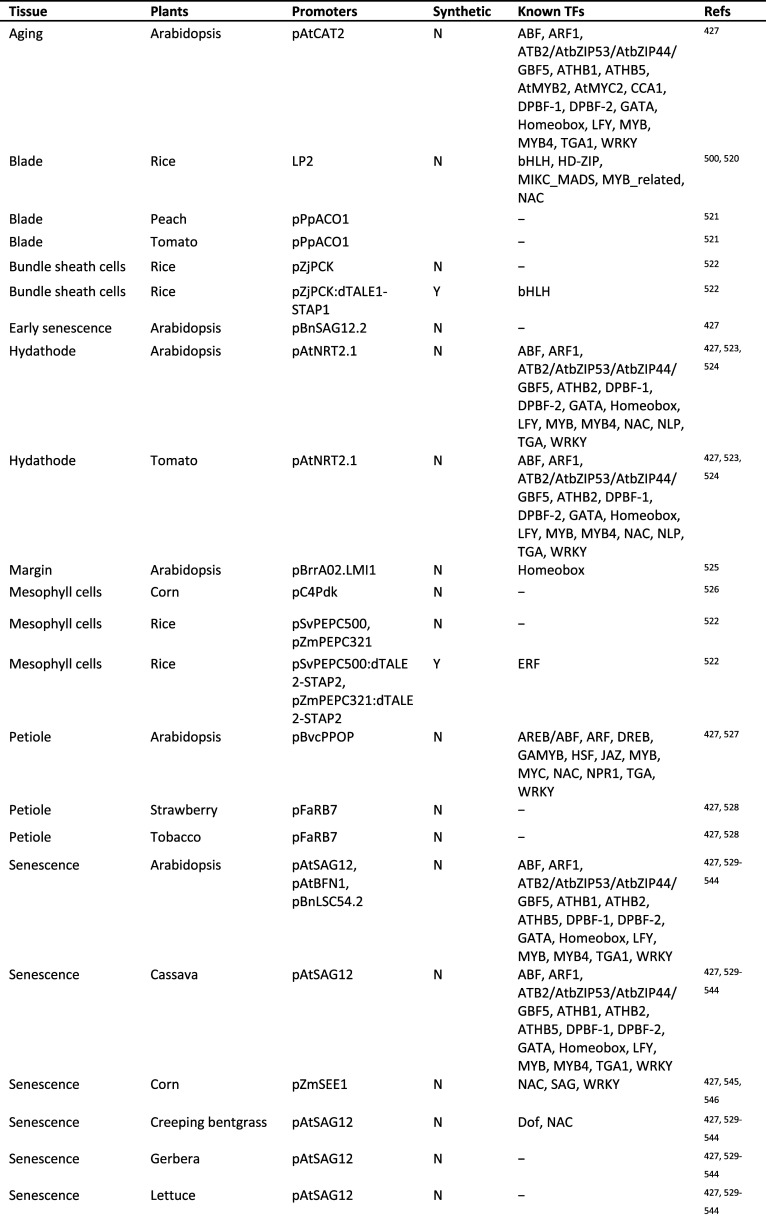

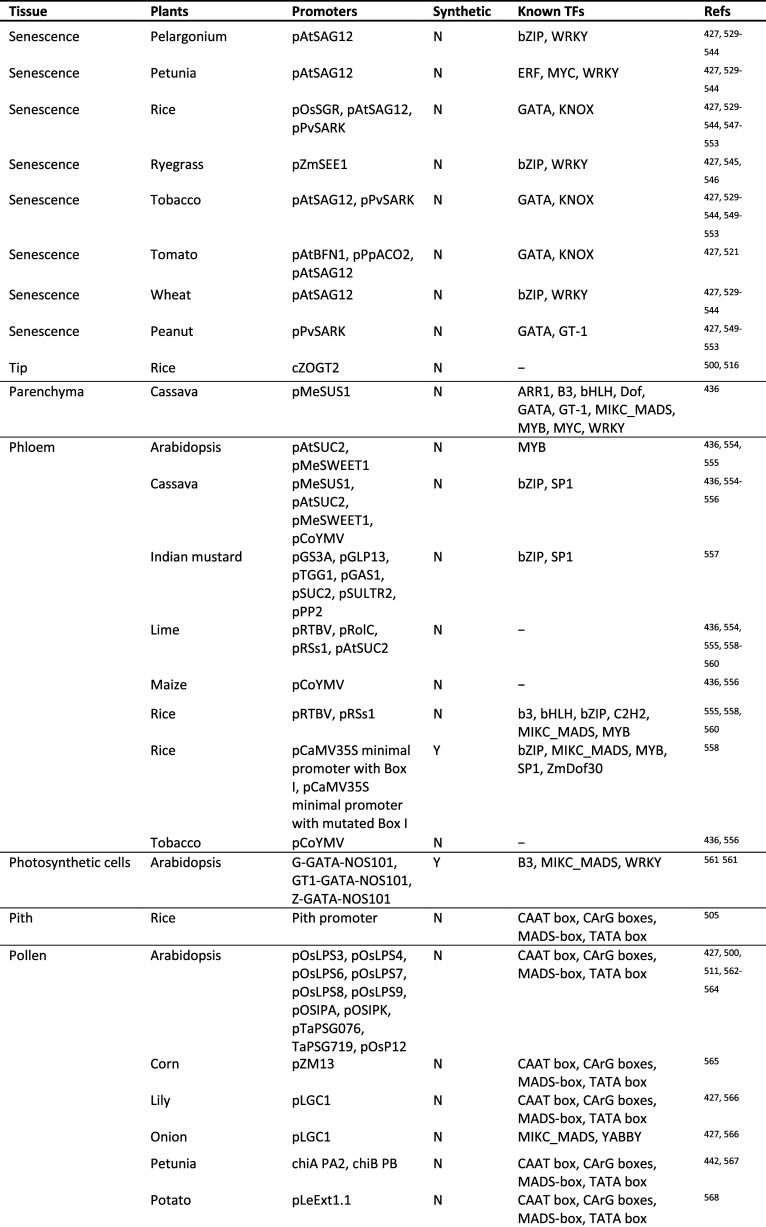

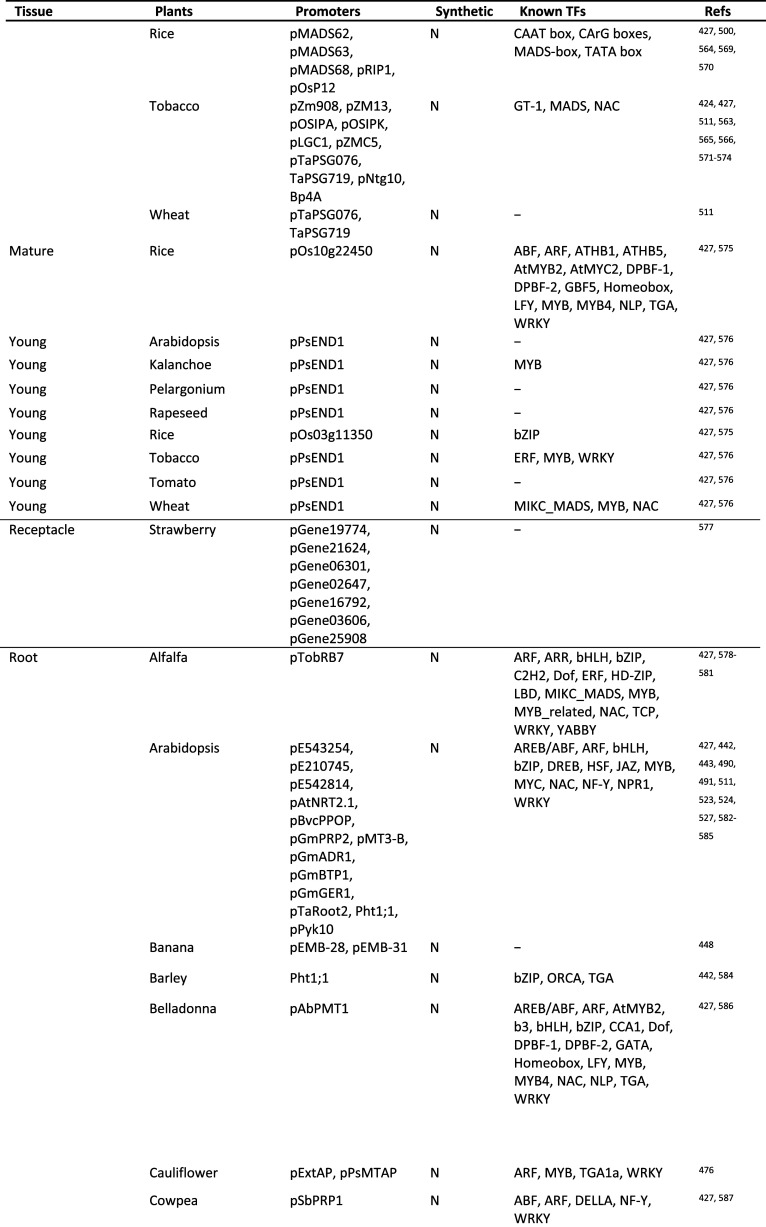

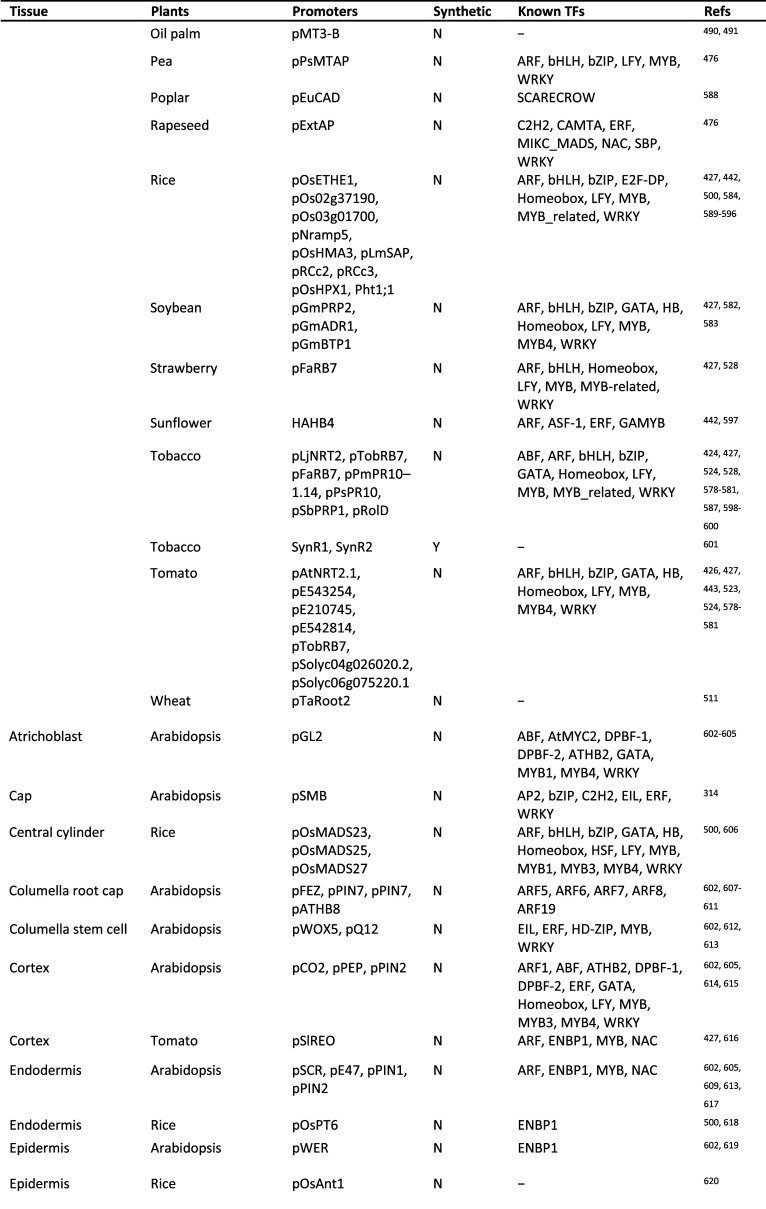

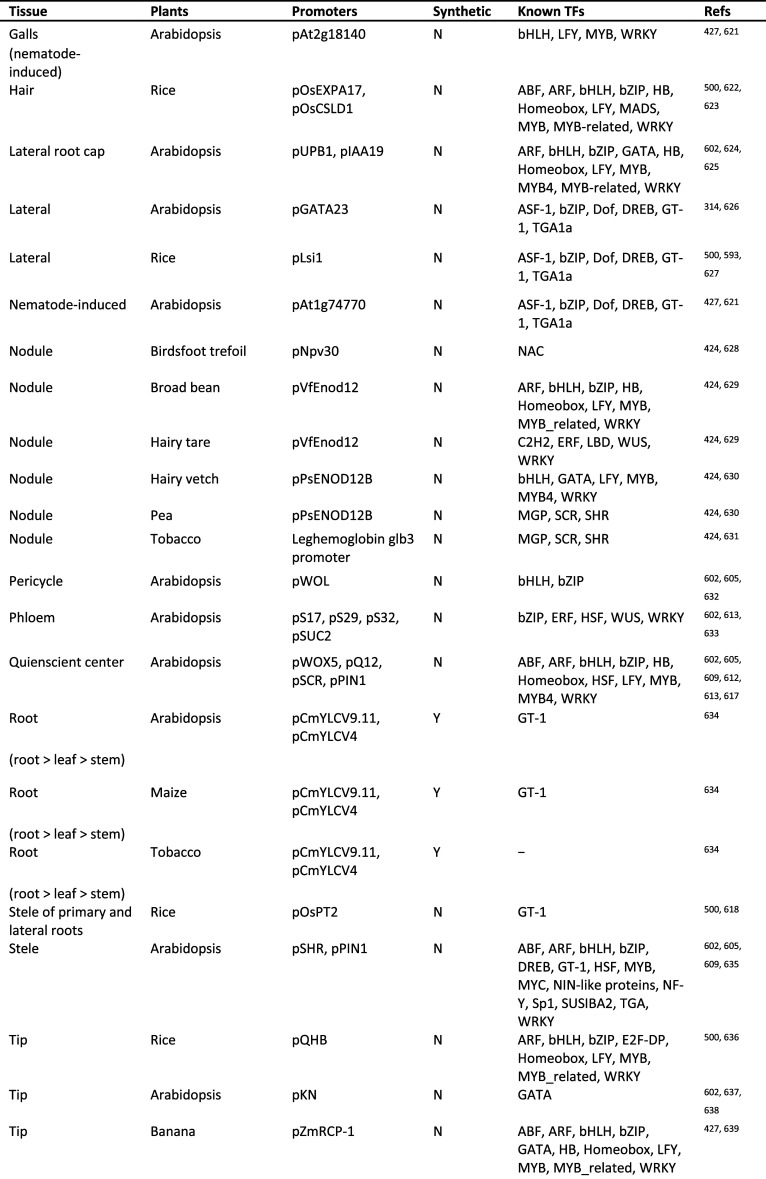

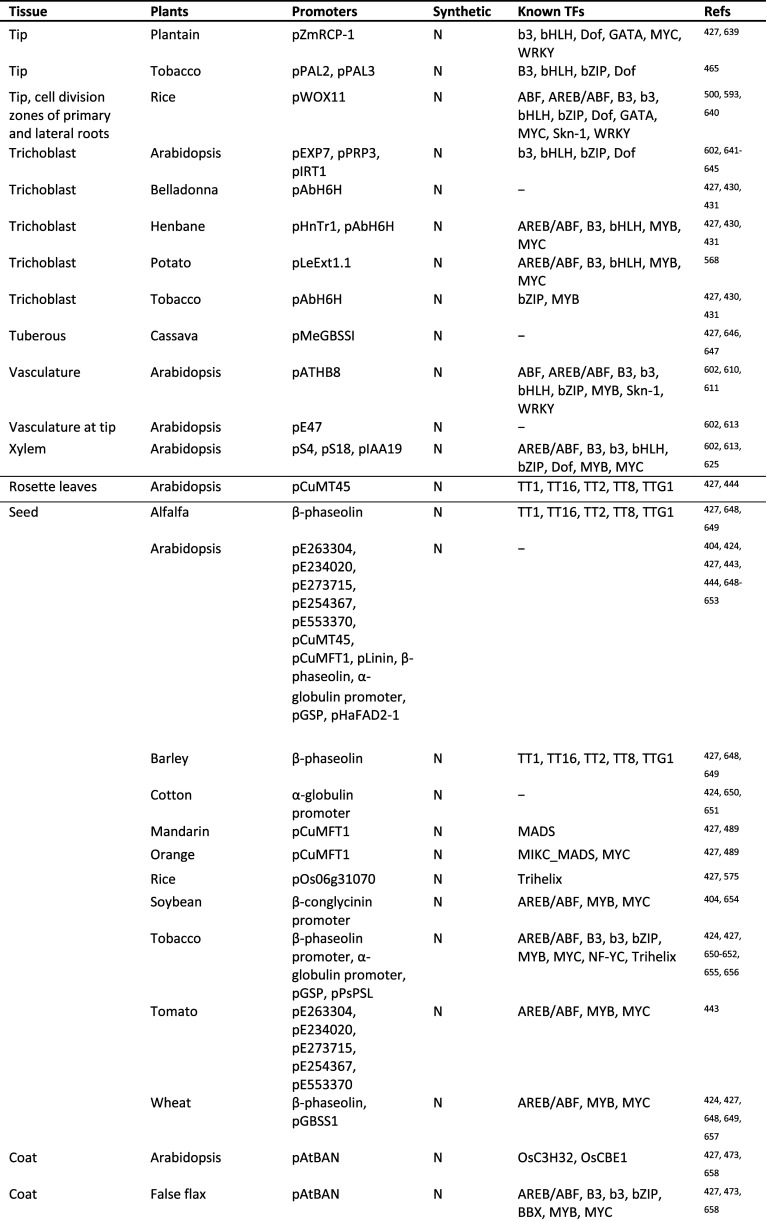

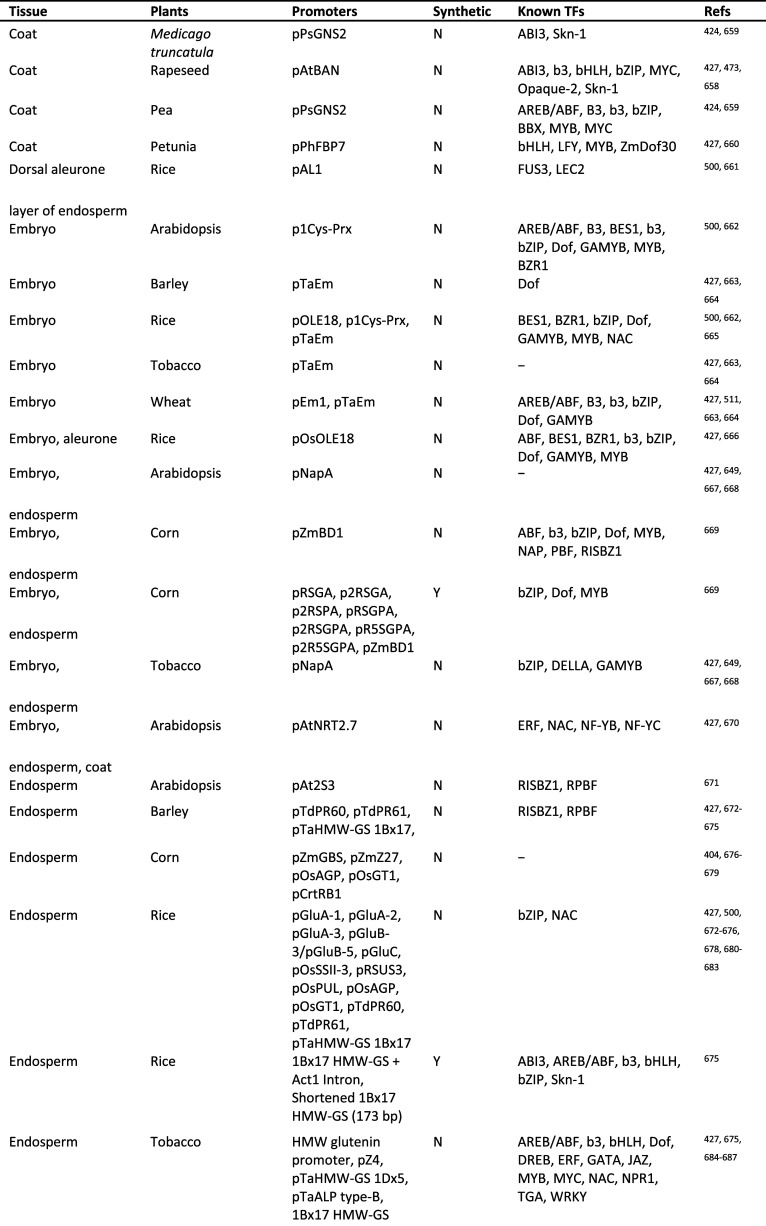

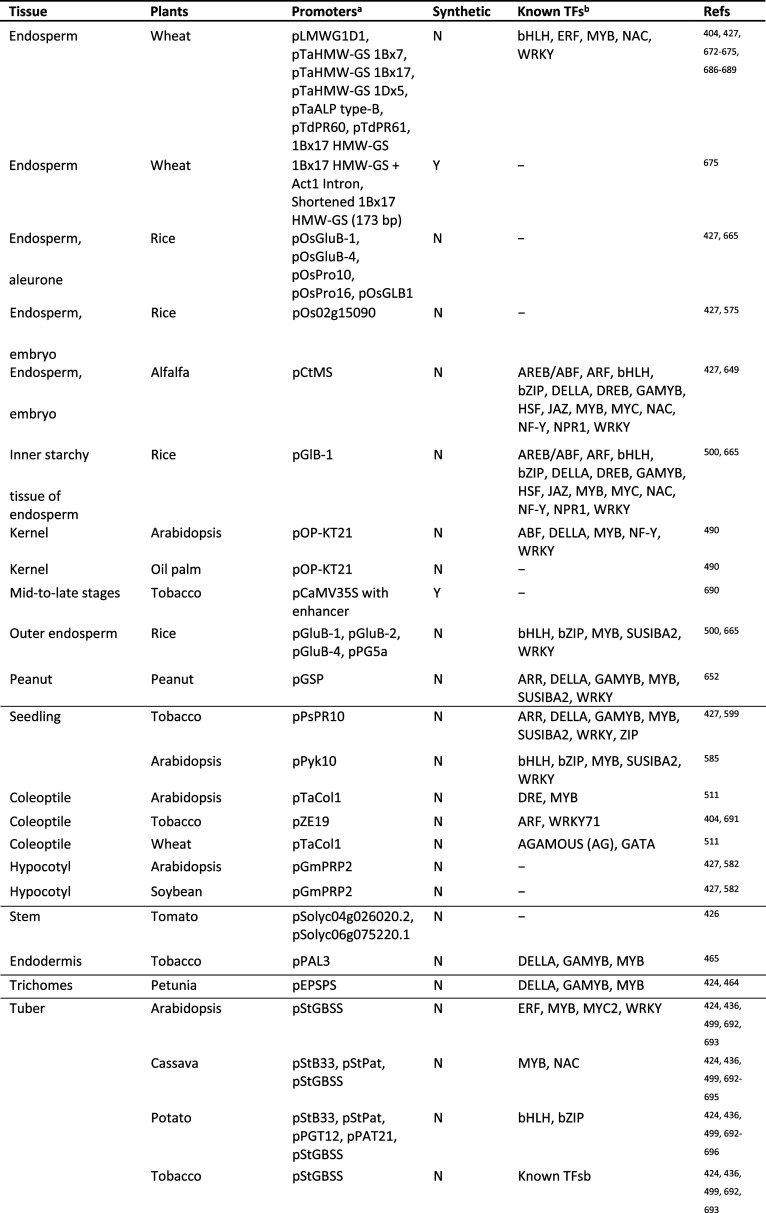

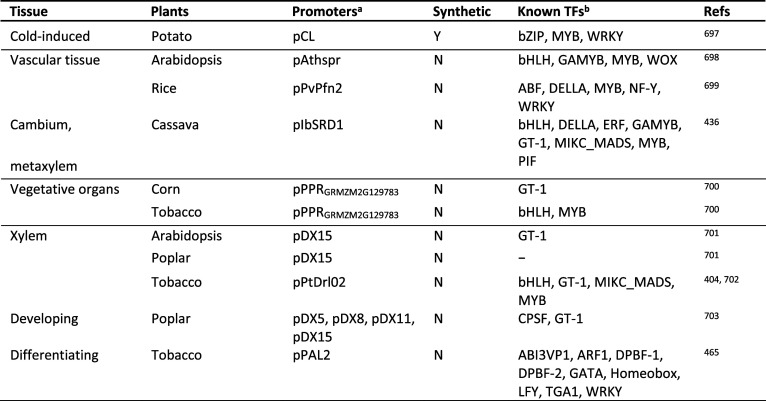
Tissue-Specific Promoters^421,422,423,424,425,426,427,428,429,430,431,432,433,434,435,436,437,438,439,440,441,442,443,444,445,446,447,448,449,450,451,452,453,454,455,456,457,458,459,460,461,462,463,464,465,466,467,468,469,470,471,472,473,474,475,476,477,478,479,480,481,482,483,484,485,486,487,488,489,490,491,492,493,494,495,496,497,498,499,500,501,502,503,504,505,506,507,508,509,510,511,512,513,514,515,516,517,518,519,520,521,522,523,524,525,526,527,528,529,530,531,532,533,534,535,536,537,538,539,540,541,542,543,544,545,546,547,548,549,550,551,552,553,554,555,556,557,558,559,560,561,562,563,564,565,566,567,568,569,570,571,572,573,574,575,576,577,578,579,580,581,582,583,584,585,586,587,588,589,590,591,592,593,594,595,596,597,598,599,600,601,602,603,604,605,606,607,608,609,610,611,612,613,614,615,616,617,618,619,620,621,622,623,624,625,626,627,628,629,630,631,632,633,634,635,636,637,638,639,640,641,642,643,644,645,646,647,648,649,650,651,652,653,654,655,656,657,658,659,660,661,662,663,664,665,666,667,668,669,670,671,672,673,674,675,676,677,678,679,680,681,682,683,684,685,686,687,688,689,690,691,692,693,694,695,696,697,698,699,700,701,702,703^

Motifs can also be identified by screening random sequence libraries or searching databases with bioinformatic tools.^382^ Identifying CREs in these datasets is like finding a needle-in-a-haystack because the motifs are only 4-10 bp and occur in a region that is 500-5000 bp upstream of the TSS.^346^ Plant CREs that respond to pathogens were found by combining approaches.^383^ First, putative CREs were found by searching the PathoPlant database (microarray data) for *Arabidopsis* genes up-regulated by pathogen-related stimuli. This search led to 79 putative CREs, each of which was used to build a promoter by placing four copies upstream of a minimal CaMV35S promoter. After screening, 25 were found to be active in parsley protoplasts and tobacco. Increasingly, annotating and characterizing CREs in genomic data is accomplished using machine learning (Figure 8).^384^ For example, sequences surrounding the TSS were used as inputs to train a classification model to predict the level of gene expression in transcriptional data.

**Figure 8:**
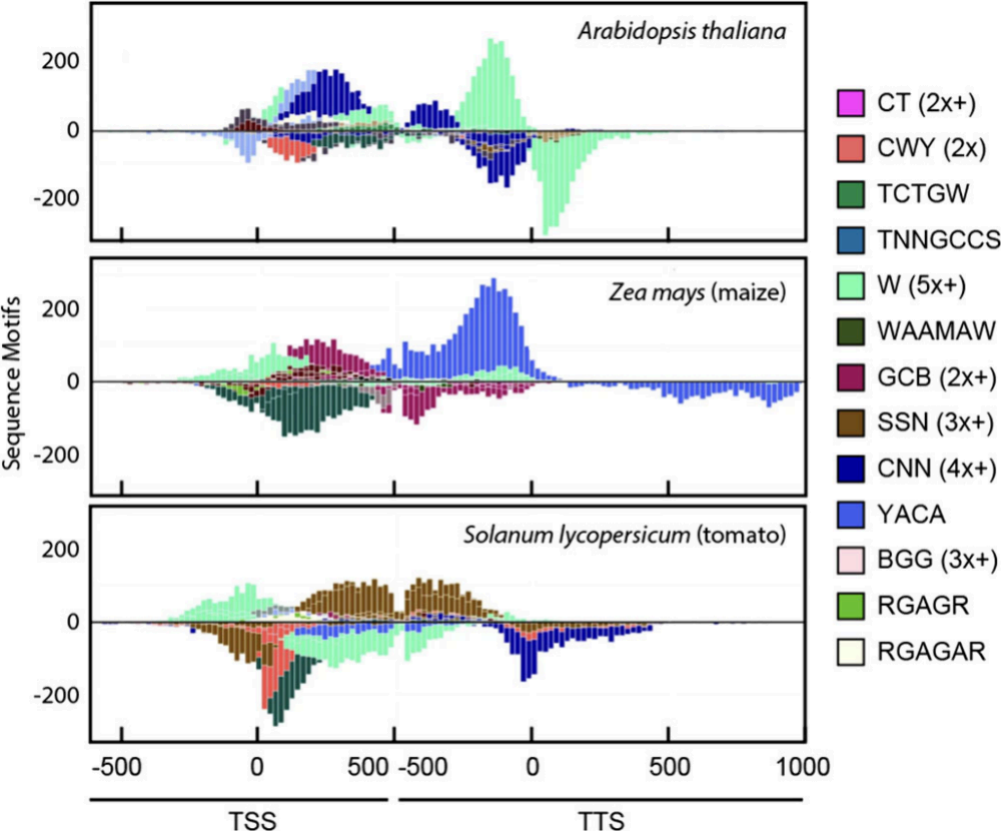
**Deep learning models to predict regulatory sequence motifs**.^384^ Sequences flanking the TSS and transcription termination sites (TTS) of genes in model plant species were used to generate input matrices to train a CNN to classify the expression of an adjacent gene as high or low expression. Through backpropagation, the importance of each nucleotide was scored, which was used as an input for the TF sequence motif algorithm to generate position weight matrices. The positional distribution of these sequence motifs was graphed relative to the TSS and TTS, where negative y-values represent occurrence in the reverse complementary orientation. Motif consensus sequences are displayed using the extended IUPAC nucleotide code, and minimum repeat length is displayed in parentheses. Image reproduced with permission from ref 384. Available under a CC BY license. Copyright 2024, Nature Communications.

There are rules for adding CREs to a core promoter scaffold. Often, CREs that bind to different TFs function independently and the spacing relative to the core does not matter.^338,356^ Even moving the motif one base pair at a time, thereby altering the rotation of the bound TF around the DNA relative to the bound RNAPII machinery, usually does not have an effect. To avoid steric clashing, CREs should be spaced between 15-20 bp apart from each other (the center of their sequences by about 50 bp).^338^ CREs have a bell-shaped distribution in the promoter region and are enriched between -500 and -250 (Figure 6D).^360^

Changing the number of CREs can modulate the activity of a synthetic promoter.^360^ Concatenating the number of CREs linearly increases that amount of expression from a promoter up to a point, after which it starts to decline.^385^ This effect could be due to the depletion of the TF pool.^346,386^ This has been observed in synthetic genetic circuits, where the increase in TF binding sites can act as a “sponge” that alters the activation threshold.^387,388^ The sharing of a resource leads to “retroactivity,” which has been studied computationally and experimentally in bacteria and yeast.^389,390^ One of the consequences of retroactivity is that promoters can impact each other indirectly by influencing the availability of the pools of cellular proteins involved in their activity. Combining multiple CREs that bind to different TFs upstream of the TATA box, rather than repeating the same CRE, has been observed to lead to higher expression.^356^

Increasing the number of CREs also increases basal activity when used for inducible or environment-specific control.^338^ This effect may have different consequences depending on the application.^338^ For example, in some cases the maximum inducibility is required and no background is tolerated; for instance, when expressing toxic proteins. In other cases, the background may not matter, but the highest possible expression needs to be achieved when induced.

The ability to insert multiple CREs into a synthetic promoter enables the implementation of logic operations that integrate many environmental and cellular signals.^391^ For example, the insertion of CREs that respond to salicylic acid, jasmonic acid, and ethylene created a promoter that responds to different combinations of these signals.^369^ Natural plant regulatory networks have interconnected webs of TFs that combinatorically impact different promoters, thereby implementing logical operations to enable the cells to perform computation for pathogen defense, metabolic needs, and development.^392^ This type of signal integration upstream region of a promoter has been more thoroughly studied for bacterial and yeast promoters.^339^

Note that using only a sequence motif to direct TF binding to promoter DNA is a simplification. In reality, it is also affected by the local region, DNA shape, and context of other binding events and it must scan a region of about 90-150 bp when finding the target. ML and protein-folding models (*e.g.*, AlphaFold) help to identify these factors and incorporate them into mathematical models of binding.^393^ More comprehensive models will aid the combination of CREs to integrate signals at longer synthetic promoters.

###### 2.2.1.1.4 Differences between Monocot and Dicot Promoters

In the literature, it is often noted that an important consideration for selecting a promoter is whether it was sourced from a monocot or dicot. Plants separated into monocots and dicots about 150 million years ago and have different genome organizations and promoter architectures. It has been observed that some promoters work in one branch but not the other, or they work in both but have very different levels of activity.^357,394–403^

Simple core promoters tend to work in both but can exhibit variable activity. Core promoter motifs such as CCAAT, Y-patch, TATA-box, and TSS/Inr are shared between both branches, but their relative abundances differ.^326,327,404^ The sequence motifs around the TSS also differ (Figure 6G).^329^ Viral promoters tend to function similarly in monocots and dicots. For instance, the CaMV35S promoter, the SCBV promoter, and the Milk vetch dwarf virus promoter have proven versatile across monocots (rice, wheat, etc.) and dicots (tobacco, Arabidopsis, etc.).^405–410^ However, they can also show differences in expression levels between plant species and within a species across tissues.^405,406,408,411^ Deletion analysis of the sugarcane bacilliform virus promoter revealed nuances in activity levels when used in different plant species.^412^ Note that these studies use natural promoters, which can be sensitive to TFs present in one branch and not the other. They also measure “promoter activity” using methods that group it with mRNA transport degradation and protein translation, which differ between monocots and dicots.^413^ Therefore, the degree to which a well-annotated constitutive core promoter generates different RNAPII fluxes between monocots and dicots remains unknown and it could be less variable than what is assumed.

Differences between CREs are an important design consideration between monocot and dicot promoters.^326,327,414–416^ CREs are frequently active in both, but enhance activity more strongly in one over the other. For example, some CREs (G-box, ABRE, STRE) are more abundant in the promoters of highly expressed genes in dicots, while others (Skn-1, GCN4, CAAT-box) are more frequent in monocots.^417^ Some are able to lead to high levels of expression in both; notably, the AG-box motif.^418^

A better understanding of these effects, shared motifs, and perhaps the integration of feedforward/feedback loops^358^ could lead to “universal” promoters that produce the same levels of activity across monocots and dicots. Toward this goal, promoter libraries have been built and tested in both branches. High levels of expression in both can be obtained by using strong enhancers (*e.g.*, the murine Emu) with efficient leaders (TMV omega prime).^419^ These promoters have been used for recombinant expression in dicots (tobacco, Arabidopsis) and monocots (rice, wheat, maize). Libraries of promoters have been tested across both branches: 6 promoters (rice, tobacco),^420^ 8 promoters (wheat, Guinea grass, wild carrot protoplasts)^419^ and a large set of 18,329 *Arabidopsis*/34,415 maize/27,094 sorghum promoters (maize and tobacco protoplasts).^357^

###### 2.2.1.1.5 Bidirectional Promoters

Bidirectional promoters contain two opposing core promoter sequences and simultaneously transcribe two genes oriented in opposite directions. Endogenous bidirectional promoters can be identified in genomes by looking for diverging gene pairs with correlated expression levels.^704–706^ They are prevalent in plants and libraries of putative bidirectional promoters have been collated in *Arabidopsis*, rice, and poplar.^707^ Synthetic bidirectional promoters have been constructed by fusing a CaMV35S promoter to viral and native plant promoters in an opposite orientation.^708^ In maize, a synthetic bidirectional promoter was used to stack four recombinant genes (two translationally-fused insect resistance genes in one direction and a fused antibiotic resistance gene with herbicide tolerance gene in the other).^709^ The use of the bidirectional promoter had reduced silencing compared to when each of the four genes was place under its own independent promoter. In rice, a native bidirectional promoter was used to co-express Cas9 and an sgRNA to improve editing efficiency.^710^

###### 2.2.1.1.6 RNAPIII Promoters for Noncoding RNA

Importantly, the transcription of non-coding RNA (siRNA, miRNA, gRNA, etc.) in plants requires different promoters than those described in Section 2.2.1.1.1.^711,712^ While technically possible, promoters that use RNAPIII are usually capped, polyadenylated, and the transcript ends can vary. Plants naturally use RNAPIII based promoters when transcribing RNA products that are not translated, such as tRNAs. Because these RNAs are not capped, they tend to be restricted to staying in the nucleus. For Cas9 gRNA, the natural U3 and U6 promoters are most commonly used. The U3 promoter control of gRNA was first demonstrated with Arabidopsis.^710^ The U6 promoter was used for *Sorghum biocolor* and has been extended to other plants, including cereals.^713^

RNAPIII promoters tend to be simpler than RNAPII promoters, with some shared and some different sequence motifs. The transcription initiation site nucleotide can vary. The TATA box is the same, located approximately 25-30 bp upstream of the TSS, and *in vitro* experiments suggest that this may be all the information needed for RNAPIII initiation.^714^ There is an upstream sequence element (USE) that is required for RNAPIII initiation.^715^ The USE is typically 50-70 bp upstream of the TSS and the consensus sequence is RTCCCACATCG.^712^ Monocots require an additional monocot-specific promoter element (MSP) upstream of the USE.^716^ When present, there are 1-3 MSPs with a consensus sequence of RGCCCR.^712^ Additional upstream enhancers can increase the strength of some promoters.^711^ Minimal RNAPII promoters, containing only the most well-defined promoter motifs, can be as small as 170 bp. These promoters have been shown to function in rice.^716^ These promoters are constitutive and expressed at in all tissues at all times.^70,71,712^

Their size, simplicity, and shared sequence motifs simplify the use of promoters across species. Therefore, the creation of libraries of promoters with defined strengths are more likely to perform as expected in a new species context.^66^ Libraries of RNAPIII promoters have been built where variants have different strengths. The wild-type U3 and U6 promoters were gleaned from a variety of plants (rice, grapes, soybean, etc.), mutated, and evaluated for activity.^716–724^ Across these efforts, 44 promoters have been characterized with a range of strengths.^711^ To date, the application of these promoters has been to optimize gRNA transcription for Cas9 in various plant species. The most common promoters used for this purpose are U3/U6 from Arabidopsis (dicot) and rice (monocot).^712^ When transcribing multiple gRNAs, placing each one under a different promoter avoids hairpin structure formation.^104^

###### 2.2.1.1.7 High-Throughput Promoter Evaluation Using Protoplasts

Protoplasts can be sorted using FACS to characterize synthetic TFs and genetic part libraries (Section 2.5.3.3).^725^ This approach will be valuable for characterizing libraries of genetic parts and synthetic regulation, where it is hard to evaluate every member independently in a full plant. For example, to vary the expression level of a recombinant gene, a set of promoters, 5′-UTRs, and 3′-UTRs were combinatorically assembled into 91 expression cassettes and transformed into tobacco leaves (Figure 9).^726^ The leaves were then harvested to generate protoplasts and obtain single-cell fluorescence measurements that showed a 200-fold range in expression between constructs. Combinations of promoters (CaMV35S, maize Adh1, and sugarcane Ubi1), introns (from maize Adh1 and sugarcane Ubi1), and enhancer elements (from CaMV35S and maize Adh1) were fused to the GUS reporter and introduced into protoplasts from sugarcane (monocot) and carrot (dicot). While certain promoter-intron-enhancer combinations were effective in both sugarcane and carrot protoplasts, others showed distinct preferences for one plant type over the other.^727^

**Figure 9:**
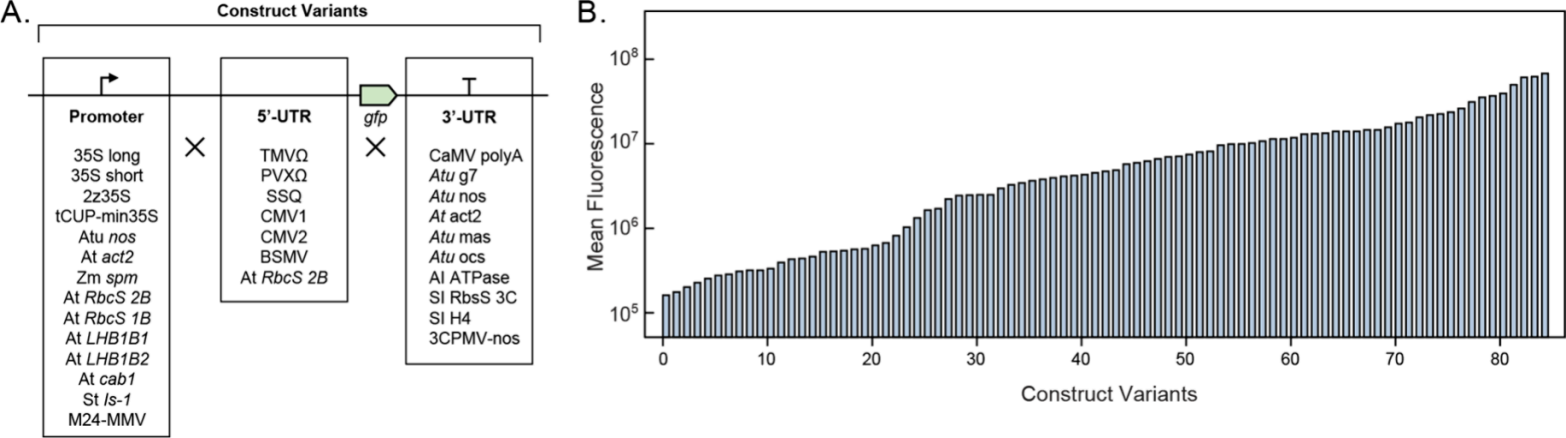
**High-throughput characterization of plant genetic parts using protoplasts**. **A**. Expression constructs encompassing a combination of native and synthetic genetic parts derived from plants and viruses were assembled through golden gate modular cloning.^726^ **B**. A combinatorial library of expression constructs was assembled using 14 plant promoters, 7 5′-UTRs, 11 3′-UTRs, and 5 promoter-5′-UTR fusions (not pictured).^726^ *N. benthamiana* leaves were transformed though *Agrobacterium*-mediated canopy infiltration followed by protoplast generation. Single-cell protoplast fluorescence was measured using flow cytometry.

###### 2.2.1.1.8 Toward Standardized Part Measurements

The use of parts in quantitative design algorithms requires that they be characterized in a way where the numbers are meaningful when shared between labs. “Arbitrary units” is the scourge of biology, where the numbers reported in one lab are not meaningful in another lab. This makes it impossible to combine lists of genetic parts from different sources without remeasuring them and design algorithms cannot select previously-characterized parts based on quantitative needs. This problem was first addressed in bacteria by defining a “reference promoter” to which the activity of other promoters can be compared.^333^ The promoter activities can then be reported in RPUs (relative promoter units).^158,728,729^ In *E. coli*, single molecule experiments were used to convert RPUs to absolute units of RNAP/s,^730^ but this conversion remains uncommon because of the technical difficulty.

In plants, the CaMV35S promoter is often the *de facto* standard to which other promoters are compared. This got more formally standardized as a specific 200 bp variant of the promoter that showed the highest activity.^731^ Carried on a plasmid, this promoter was defined as 1 RPU for plant genetic circuit design (Section 2.3). It will be difficult to use this reference between labs for stable plant line development until it becomes simpler to insert it into a defined location in the chromosome. The naming of CaMV35S promoters is also variable and the ideal length for a reference promoter is as short as possible, without being so short that it is influenced by the upstream genetic context.

Characterization of genetic parts is difficult in plants, making the precise definition of a reference promoter and its strength difficult. The measured fluorescence of a genetic part ignores sources of variability, such as the location or number of genomic integrations. To address this problem, a dual-luciferase system was developed to enable the measurement of the activity of a genetic part.^317^ The assay involves the expression of *Renilla* luciferase from a previously characterized promoter (a dexamethasone-inducible or 4-hydroxytamoxifen-promoter) and firefly luciferase from a synthetic promoter. The ratio of *Renilla* luciferase to firefly luciferase luminescence allows for comparisons of promoter activity between samples and experiments. Similar approaches have been taken for other combinations of two reporters.^731^

In addition to the promoter sequence and the reporter, a reference standard is also defined by the specific conditions under which activity is measured. To this end, the first standards for the measurement of promoter activity in plants have been proposed.^306^ The SE_001 standard is based on the Luc/Ren reporter system. It also defines the experiment in tobacco leaves. It also specifies that the transformation is performed by Agrobacterium-mediated transient expression and defines that number of integrations that must be achieved, as measured by a separate reporter. An internal reference promoter is defined and the standard specifies that it must be measured in the same experiment. However, unlike above, it defines the reference promoter as a weak natural promoter of the nopaline synthase gene and defines the activity of this reference as 1 RPU. Ultimately, it would require an effort in the scientific community to define the reference parts and conditions for their measurement.

###### 2.2.1.1.9 Plant 3′-Untranslated Regions (3′-UTRs)

In plants, transcript stability is strongly affected by the 3′-UTR.^732^ The 3′-UTR of the transcript is essential for its stability and export out of the nucleus so that the gene can be expressed.^733^ The region between the end of the open reading frame and the start of this tail is also important. The sequence in this region can stabilize the mRNA, enhance translation, and increase gene silencing.^404^ It can function independently of the promoter and 5′-UTR sequence. Adenine rich elements (AREs) often occur in this region and recruit proteins that degrade the transcript.^457^ The 3′-UTR region is also a target of miRNAs that can implement additional regulatory control by affecting transcript stability. ML can predict cleavage and polyadenylation sites in *Arabidopsis* 3′-UTRs mRNAs.^1378^

###### 2.2.1.1.10 Plant Terminators

Terminators interact with RNAPII to stop progression and adds termination signals to the mRNA that lead to its release.^327^ The transcript ends at a conserved AAUAAA (or similar) site. At this position, endonucleases cut the transcript. Then, a protein adds a poly(A) tail of 100-250 adenosines; longer tails provide more protection against degradation. It also facilitates the export of the transcript from the nucleus, and recruitment of ribosomes, so that the gene can be expressed. Note that RNAPIII promoters use different termination signals; typically, a poly-T sequence.^734^

In bacteria, terminator strengths are quantified as the fraction of RNAP that are blocked from progressing through the terminator sequence.^735,736^ Despite their importance, very few terminator libraries have been characterized for plants and there has not been work to design synthetic terminators. A library of 13 natural terminators was characterized in tobacco and shown to change expression by a few fold, but the underlying mechanism was unclear.^345^

In eukaryotes, the role of terminators is more complex, and their strength can be promoter-dependent.^345,737,738^ This effect could be due to gene looping, where DNA-binding proteins bend the DNA so that the promoter and terminator physically interact.^739–741^ Gene looping affects the variation of expression from a promoter through the reinitiation of transcription, where the RNAPII continues from the terminator to the promoter without dissociating from the DNA, as well as other mechanisms.^742–746^ High-resolution methods to probe chromatin organization and gene looping have been applied to *Arabidopsis*,^740,747,748^ maize,^749–751^ rice,^749,750,752^ cotton,^753^ and sorghum.^749^ The control of looping can lead to different expression levels. For example, regulators of gene looping control flowering in *Arabidopsis* by modulating FLOWERING LOCUS C (*FLC*) and fine-tune the expression of UNBRANCHED3 (*UB3*), which influences kernel row number in maize.^741,754^

Terminators differ in their ability to stabilize transcripts, an example being the HSP terminator which provides 3-fold increase in mRNA levels compared to the NOS terminator.^755^ In addition, strong terminators avoid the production of aberrant transcripts without polyadenylation and sRNAs, leading to gene silencing.^732^ The strength of terminators can be host-dependent.^756^ As a result, viral- or pathogen-derived terminators, such as the *Agrobacterium*-derived NOS and OSC terminators, are commonly used because they are highly efficient and have a broad host range.^757,758^ However, even for these terminators, there can be wide variation in expression levels across species. Ten terminators screened in *Medicago truncatula* and barley had no correlation in terminator strengths between the two species highlighting the importance of identifying appropriate terminators for optimal recombinant gene expression (Figure 6H).^330^

##### 2.2.1.2 Translational Parts

To produce protein, an mRNA transcript must be exported from the nucleus. The sequence and chemical form of the mRNA dictates the level of expression (Figure 10A). The 5′-UTR cap is usually required for translation in eukaryotes (Section 2.2.1.1.8). In addition, the region upstream of the translational start codon and 3′-UTR (Section 2.2.1.1.9) both play important roles regulating translation efficiency.^759^ Finally, the poly(A) tail at the 3′- end of the transcript recruits the protein complex that unfolds mRNA secondary structure and facilitates ribosomal assembly.^760^ Secondary structures throughout the mRNA can also block the progression of the ribosome.^761^ Binding sites for RNA-binding proteins (RBPs) and small RNAs can also modulate translation.^762,763^

**Figure 10:**
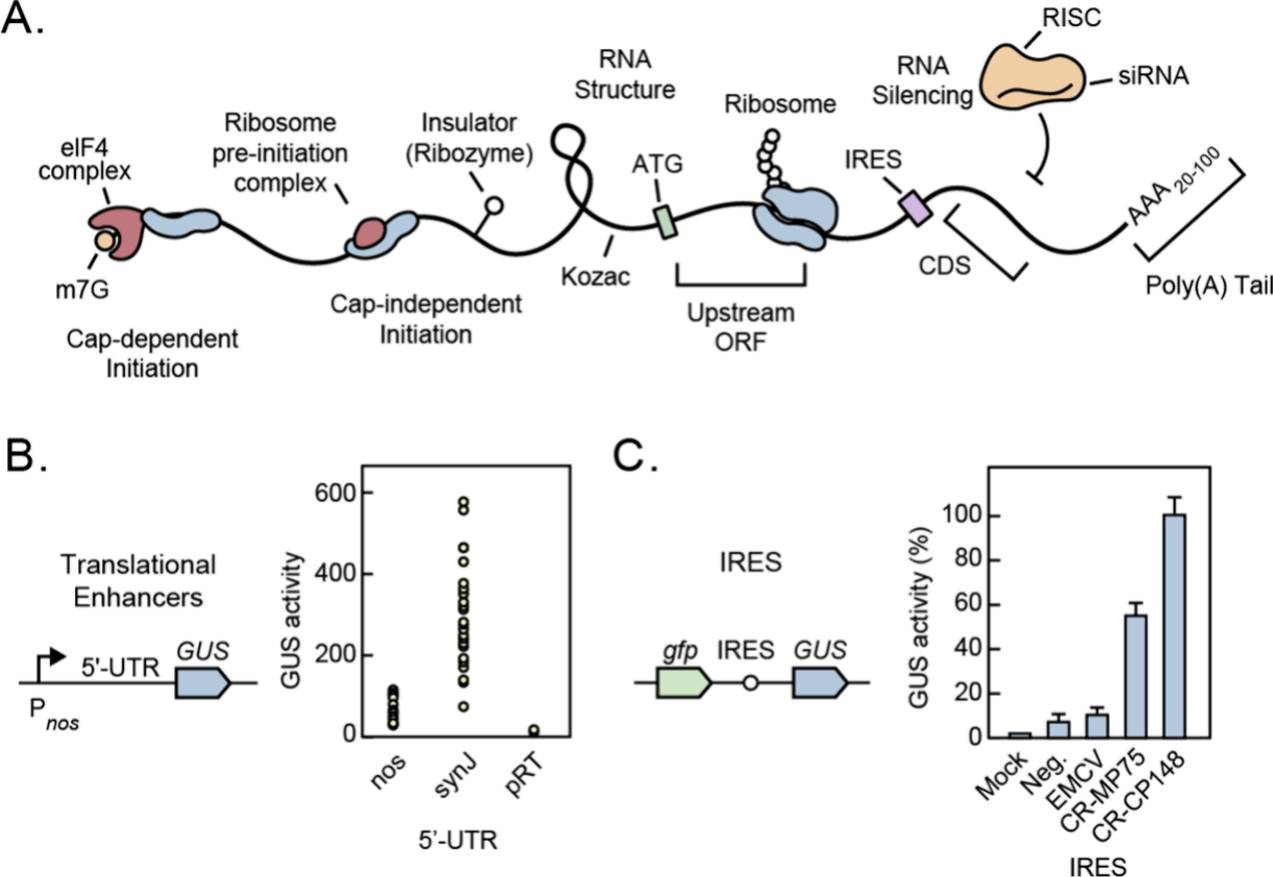
**Translational parts**. **A**. Schematic of translation regulation in plants. Translation is initiated when the eIF4 protein complex recognizes the modified guanine nucleotide cap, which begins ribosomal recruitment. In some cases, ribosomes can initiate translation in a cap-independent manner at specific sequences, such as internal ribosome entry sites. The ribosome pre-initiation complex scans the mRNA until reaching a start codon; the presence of Kozac sequences and uORFs can influence translation initiation and efficiency. Various components of the mRNA, such as secondary structure and the 3′-polyadenylated tail, influence mRNA stability and recruitment of proteins that regulate translation. RNA silencing inhibits gene expression by targeting mRNA for degradation. **B**. A synthetic translation enhancer (*synJ*) was used as a 5′-UTR to increase translation initiation of recombinant genes in cotton calli.^764^ Cotton calli were transformed with vectors encoding a *GUS* reporter expressed from the *Agrobacterium* nopaline synthase *(nos)* promoter upstream of one of three 5′-UTRs: the native *nos* 5′-UTR, synJ, or one from the plant expression vector (pRT) carrying a consensus kozac sequence. Each dot represents expression measurements from individual transformed calli. **C**. IRES translation initiation frequency.^765^ The initiation efficiency of various IRES sequences was measured by comparing *GUS* activity to *gfp* fluorescence when the candidate sequence was placed between the two genes and transfected into tobacco protoplasts. The candidate sequences were derived from known upstream IRES in the encephalomyocarditis virus (EMCV) or crucifer-infecting tobamovirus (CR) genomes. As a control, either no DNA (Mock) or a construct containing a 68-nt GCU-rich sequence (negative control; Neg.) was also transfected.

###### 2.2.1.2.1 Plant 5′-Untranslated Regions (5′-UTRs)

The 5′-UTR comes after the TSS and is transcribed as part of the mRNA containing the gene-of-interest. It is important in regulating prokaryotic expression, where it impacts the stability of the transcript and can block or expose the ribosome binding site (RBS) and ATG. In plants, the 5′-UTR also plays an important role in the expression of genes via a suite of mechanisms, including its processing into multiple isoforms.^337,766^ One role of the 5′-UTR is to be “capped,” where a 7-methylguanosine (m7G) is added when the nascent mRNA is only about 20–30 nucleotides long. The cap is a signal that protects against degradation, facilitates nuclear export, and recruits ribosomes for translation. It also participates in nonsense-mediated decay, where transcripts with premature stop codons are degraded to avoid expressing potentially harmful truncated proteins.^766^

The 5′-UTR also affects gene expression either by encoding sequence motifs that interact with RNAPII downstream of the TSS or through internal sequences that recruit or block ribosome binding. Some plants and plant viruses have translational enhancers encoded at the 5′-end of the transcript.^767–769^ The 5′- and 3′-UTR regions have not yet been fully exploited in controlling expression in plant engineering, but work with machine learning has begun to illuminate the importance of these regions.^384^

Plant 5′-UTRs can contain a small upstream open reading frame (uORF) before the main gene.^404^ The ribosome can start to translate the short uORF and then reinitiate translation of the main open reading frame through a process known as “leaky scanning”.^770,771^ The distance of the uORF to the main open reading frame and the sequence and length of the uORF all dictate the impact on expression.^772^ These sequences can be identified by computational analysis and mutated to remove them. This can be desirable in plant engineering because introns are usually used to increase expression, but the uORF could have the opposite effect.^404^ As a design strategy, uORFs can be placed in a transcript to modulate the translation of the downstream gene.^773^ uORFs could potentially also be used for other purposes. In *E. coli*, they were used to obtain a more reliable RBS strength.^301^ It was observed that their presence made the RBS controlling the main open reading frame less dependent on genetic context, making their pre-measured strengths more reliable.

A synthetic 5′-UTR for plants (78 bp) was constructed by fusing motifs. The downstream 5 bp from the TSS of the CaMV35S promoter was fused to a CT-rich region (an octamer combined with nine CAA repeats).^774^ The resulting 5′-UTR was found to be about 10-fold stronger than a commonly used one from an expression vector. Rational design and machine learning has also been applied to created libraries of 5′-UTRs that yield different strengths through the combination of motifs.^775,776^

Translational enhancers can be derived from many plant RNA viruses and are useful for obtaining high protein expression. Some that are commonly used, such as the tobacco mosaic virus (TMV) Ω enhancer, have been shown to recruit ribosomes without requiring a properly-capped and polyadenylated transcript.^777,778^ A 28 nt synthetic 5′-UTR containing repeats of sequence motifs present in the Ω enhancer was used to enhance gene expression in tobacco and cotton to higher levels than the native Ω enhancer, but it varied depending on the plant tissue (Figure 10B).^764^ As a rule, the strengths of viral enhancers are variable across plant species and match the host range of the source virus.^779,780^

Including an intron in the 5′-UTR has been found to improve recombinant gene expression in plants.^781^ The mechanism is unknown with hypotheses that encompass increased rates of transcription, pre-mRNA processing, mRNA export, mRNA stability, and translation.^782,783^ Some design rules have been discovered, such as the placement of the introns in the correct orientation within the transcribed sequence and close to the TSS.^784^ A few dozen introns have been used to modulate expression or restrict it to specific tissues.^785^ However, due to the lack of mechanistic understanding, the use of introns to quantitatively tune gene expression is difficult. The effect of an intron on expression also varies across plant species.^784^

Interactions between intronic sequences and proteins involved in transcription and mRNA processing differ between monocots and dicots.^413^ For example, an intron from the rice cytosolic superoxide dismutase gene (*sodCc2*) enhances gene expression in rice, wheat, and maize. However, it does not enhance expression in dicots, such as Arabidopsis and tobacco. Intron-mediated enhancement (IME) is hypothesized to be due to conserved motifs, which are required for efficient splicing and gene expression in monocots but not in dicots.^786^ The monocot (maize) pre-mRNAs were processed less efficiently compared to the dicot (pea) pre-mRNAs in the tobacco host.^413^ IME can increase mRNA accumulation by 10-fold, but it must be in the correct orientation and within 1 kb of the ATG of the gene.^300^ An algorithm was developed to predict whether an intron is likely to exhibit IME and several have been characterized as parts.^787–789^

The use of introns needs to be done with caution because they can lead to the silencing of recombinant genes.^300^ Hairpin structures within the intron are known to correlate with silencing.^790^ Before using an intron, it should be examined for such structures. In addition, the removal of sequences that could be splice sites has been shown to increases expression.

###### 2.2.1.2.2 Internal Ribosome Entry Sites (IRESs)

Native plant genes are most often expressed alone as monocistrons. That being said, ribosomes can also be recruited to an internal site in the transcript using an IRES.^759^ The use of IRESs allows multiple genes to be encoded as a polycistronic operon. This organization could aid the introduction of traits into the plant that require multiple genes without requiring a promoter being used for each gene. It also couples the genes transcriptionally and translationally, potentially allowing for the ratio between the proteins to be kept constant, which is sometimes important.

The use of an IRES also bypasses the need to cap the mRNA, thus enabling polycistronic expression in plants.^759^ Many plant RNA viruses naturally rely on IRESs to recruit ribosomes without the standard protein translation initiation machinery.^791^ The IRES from *Tobacco mosaic virus* promotes the translation of a second cistron to 30% the levels of the first cistron in tobacco (Figure 10C).^765^ Several other IRES’s from plant viruses have been shown to function in plants.^760^

##### 2.2.1.3 Insulators

Advanced genetic design requires that parts perform reliably when used in different contexts. The central assumption made when defining a DNA sequence as a “genetic part” is that its function is only determined by its sequence and not the neighboring sequences or genetic context (*i.e.*, different locations in the genome). However, this assumption is clearly not true for any cells, much less the complex genetics of plants. To this end, new classes of insulator parts have been developed with the intent of reducing context effects to increase part reliability.^301,302,358,359,792^

###### 2.2.1.3.1 Control of Chromosome Remodeling and Gene Silencing

Plant chromosomes have complex tertiary structure with protein factors and DNA modifications that control access of a gene to transcriptional machinery. As a result, there are regions of the chromosome that are associated with high or low expression of a recombinant gene. Several approaches have been taken to reduce these effects so that expression is dependent only on the construct being inserted and not local context effects. High-throughput open chromatin mapping (Hi-C and related techniques) can aid in identifying boundary elements.^793,794^ The native function of these sequences is to interact with DNA-binding proteins to insulate genes from heterochromatic silencing. The use of Hi-C to identify boundary elements has been used in *Arabidopsis*, rice, cotton, and maize.^752,795,796^

Boundary elements are believed to act in two ways. First, they could interfere with unwanted enhancer activity by physically separating enhancer binding sites from the core promoter. Second, they could protect a recombinant gene from position-dependent silencing by heterochromatin histone modifying enzymes that create binding sites for silencing factors.^797^ Matrix or scaffold attachment regions (MARs/SARs) are a well-studied class of boundary elements composed of A/T-rich sequences that facilitate nuclear matrix binding, resulting in the structural organization of the chromatin. MARs from tobacco, petunia, and *Arabidopsis*, as well as chicken, have been used to insulate recombinant genes in plants.^798^ In *Arabidopsis*, flanking a gene by MARs (*e.g.*, AtS and MAR10) increased its expression and reduced the expression level variation between transgenic lines (Figure 6I).^331^ By combining nuclear matrix protein binding sites and other MAR sequence motifs, small synthetic MARs were constructed that were functional in both *Arabidopsis* and rice.^799^ Databases of MARs/SARs binding sites are available.^800^

Genetic parts, including promoters and terminators, derived from viruses or pathogens can also lead to gene silencing.^801^ Gene silencing mediated by small RNAs (sRNAs) regulate gene expression in plants.^732^ They can result from double-stranded RNA (dsRNA) degradation by enzymes like DICER and the resulting sRNAs complex with ARGONAUTE proteins to repress target gene expression, including through mRNA degradation.^732^ Genetic parts can be included in the mRNA that are less susceptible or resist silencing. For example, strong terminators induce polyadenylation of transgene transcripts, which decreases silencing by preventing RNA-dependent RNA polymerases from producing siRNAs.^802,803^ Intron splicing blocks the silencing of highly expressed endogenous genes. Including Rubisco-derived intron sequences improves transgene expression by suppressing RNA silencing.^804,805^ However, endogenous introns differ in their capacity to increase gene expression, potentially due to differences in intron splicing efficiency.^806^ Another approach to suppress silencing is to express the P19 protein from *Cymbidium ringspot virus* to sequester sRNAs.^807^

###### 2.2.1.3.2 Blocking Ribosomal Read-through

Synthetic systems often differ from natural genetics due to how close multiple genes are encoded in a design. Even when each gene has its own promoter and terminator, they are inevitably imperfect and RNAPII from an upstream promoter will readthrough and synthesize transcripts with multiple genes. Bacterial ribozyme insulators (*e.g.*, RiboJ) were developed to decouple promoter strength from the 5′-UTR of the mRNA.^302,808^ These ribozymes have been moved to yeast to eliminate the translation of undesired upstream elements due to transcriptional read-through between neighboring cistrons.^147^ They could perform a similar function if used in plants.

##### 2.2.1.4 RNA Interference, Post-transcriptional Gene Silencing, and CRISPR-Mediated RNA Editing

Plants possess post-transcriptional gene silencing mechanisms that regulate or suppress gene expression. Genes are sequence-specifically targeted by sRNAs, including small interfering RNA (siRNA) and microRNA (miRNA), which are processed by and loaded onto protein complexes to coordinate mRNA degradation or translation repression. Many crop traits have been improved by introducing or manipulating sRNAs, including enhancement of plant disease and pest resistance, alternation of plant architecture, and removal of toxic compounds.^239,809–813^ The first GMO plant approved in the U.S. for human consumption was the “Flavr Savr” tomato, which was based on the expression of a siRNA that knocked down polygalacturonase, an enzyme that degrades pectin in the fruit wall making it more likely to be damaged during transport.^814^ Since then, many trait improvements derived from siRNA and miRNA have been applied to problems across agriculture.

RNA interference (RNAi) is a method to knockdown genes by specifically targeting their mRNA for degradation. The mechanism is derived from native defense pathways that target invasive nucleic acids, such as viruses and mobile genetic elements. RNAi is initiated by the transcription of a long double stranded RNA (dsRNA) or hairpin RNA (hpRNA), which are processed into many siRNAs comprised of 21 to 24-nucleotide duplexes by the enzyme Dicer.^815^ An RNA-inducing silencing complex (RISC) complex is formed by binding of the siRNA to the ARGONAUTE proteins, which complex with the complimentary mRNA to cause its cleavage and degradation.^816^ In some cases, RNAi can result in epigenetic modifications, such as DNA methylation, that further contribute to gene silencing.^817^

The programmability of RNAi makes it an effective means to disrupt native gene expression. Artificial siRNAs are generated in plants using a 100-800 bp inverted repeat of the target sequence to form a hpRNA.^818^ The cassette is often placed under the control of the strong CaMV35S promoter and the inclusion of an intron sequence can improve the knockdown of the target gene. An alternative RNAi strategy is virus-induced gene silencing (VIGS), which utilizes plant RNA viruses, such as tobacco mosaic virus (TMV) and potato virus X (PVX), that produce dsRNA during their life cycle.^819^ Delivery of the viral genome containing the 300-500 bp of the intended target transcript leads to the efficient knockdown of the target gene. The viral genome is typically delivered by *Agrobacterium* transformation.

RNAi has been used to control diverse phenotypes in crop plants, from improving crop traits (improved nutrition, seedless fruits, plant biomass regulation, flower coloration and scent, shelf-life enhancement, abiotic stress tolerance, etc.) to protecting against diseases (fungi, nematodes, pathogens, etc.).^820–823^ In one example, gentian flower color has also been modified with hairpin RNA that knocks down enzymes in anthocyanin and flavonoid pathways (Figure 11A).^824^ To make alfalfa more digestible to livestock, it was engineered to constitutively transcribe siRNA to knockdown a lignin biosynthesis enzyme in vascular tissue.^825–827^ Transgenic peanut plants expressing siRNAs that target a potent food allergen were found to be less likely to cause immune activation in peanut allergy patients (Figure 11B).^828^

**Figure 11:**
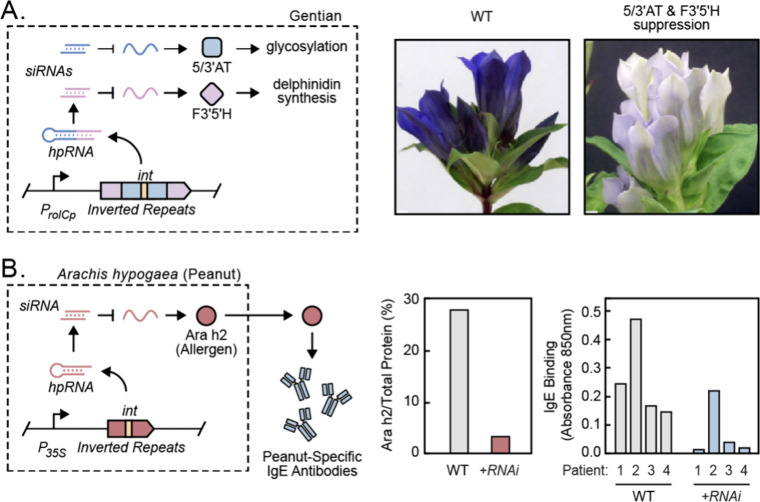
**Engineered phenotypes using RNAi**. **A**. Inducible altered flowering phenotypes in ornamental gentian (*Gentiana trifloral*).^824^ The proteins 5/3′AT and F3′5′H are both required to produce the pigment gentiodelphin. Expression of siRNAs as an inverted repeat and targeting 5/3′AT and F3′5′H produced altered flower color. The siRNAs were expressed from a strong promoter from *Agrobacterium rhizogenes* (*rolC* promoter). Images of gentian flowers reproduced with permission from ref 824. Copyright 2010, Journal of Plant Physiology. **B**. Alleviating peanut allergies by silencing allergens in peanuts using RNA interference.^828^ The protein Ara h 2 is the most immunodominant allergen in peanuts and causes human peanut allergies by binding to allergen specific IgE antibodies. A siRNA cassette constitutively expressing a 265bp inverted repeat of the Ara h 2 coding region separated by the *pdk* intron (*int*) decreased the relative concentration of Ara h 2 in peanut protein extract. Protein extract from the transgenic plants (+RNAi) caused less IgE binding than protein extract from wild-type peanuts (WT) in serum in four patients (1, 2, 3, and 4) with a known peanut allergy as measured by an ELISA assay.

miRNAs also play a role in targeted gene silencing by repressing translation and inducing mRNA degradation, although the details of their synthesis and function differ from siRNAs in RNAi. Notably, while the role of siRNAs is generally to cause the near-total suppression of virus or transposon-derived genes, miRNAs precisely fine-tune the expression of endogenous genes. Before interacting with DICER, AGO, and RISC proteins, miRNAs are synthesized from long precursor primary RNAs (pri-miRNA) that form hairpin-like structures required for their processing, which complicates the design of artificial miRNAs.^829^ Therefore, artificial miRNAs incorporate a target sequence within an existing endogenous miRNA such that the hairpin-like structure, as well as structural features such as bulges or mismatches, are maintained. Several software tools, including Web MicroRNA Designer, have been built to facilitate plant-specific miRNA design.^829–831^ The *Arabidopsis*-derived *MIR390a* is a common miRNA precursor used to express artificial miRNAs in many plant species.^832^

Applications of miRNA-based gene silencing resemble those using siRNA or RNAi and include increasing disease resistance by targeting virus suppressor genes.^833^ The degree of gene silencing caused by artificial miRNA can be quantitatively tuned by varying miRNA-target complementarity.^834^ miRNAs are less prone to off-target effects as they can be designed to avoid sequence similarity with nontarget genes and avoid the generation of secondary sRNAs.^835,836^ miRNAs are also less likely than siRNAs to spread systemically, making them the preferred route to perform tissue-specific gene silencing.^837^

CRISPR/Cas13 targets and cleaves ssRNA analogously to how CRISPR/Cas9 targets DNA (Section 2.1.1). As a post-transcriptional gene silencing method, CRISPR/Cas13 enables programmable gene expression control and viral suppression.^838^ Expression control was demonstrated in cotton by varying Cas13 activity or the crRNA sequence for a target gene.^839^ Virus-resistant Tobacco and *Arabidopsis* plants were made by expressing Cas13 and CRISPR RNAs (crRNAs) targeting the RNA genome of Turnip mosaic virus.^840,841^ Analogous to PAM sites in Cas9 systems, Cas13 variants have different preferences for the sequence of the binding site (protospacer flanking sites), thus increasing the targeting flexibility.^839,842^ Methods for targeting multiple RNAs and the use of Cas13 to base edit mRNA have been developed in mammalian cells,^843–845^ but not yet applied to plants. Note that sometimes crRNAs can also trigger post-transcriptional gene silencing in the absence of Cas13 by triggering endogenous RNAi machinery.^846^

##### 2.2.1.5 Protein Tags

Peptide tags or small protein domains can be fused to a protein to direct it to a destination in the cell, make its function dependent on other factors, or alter stability. Ideally, these tags act modularly, imparting their effect on whatever protein to which they have been attached. Tags can also be added to convert a DNA-binding protein to a regulator or sensor (Sections 2.3.2 and 2.3.3.b).

###### 2.2.1.5.1 Protein Localization

Peptide tags are used like “zip codes” to localize proteins to subcellular compartments, including the nucleus, chloroplast, or mitochondrion, or to target them for secretion and glycolsylation.^847,848^ Subcellular localization can be required for the proper activity of foreign proteins. For example, proteins that interact with genomic DNA, such as nucleases or TFs, need to localize to the nucleus and some metabolic enzymes require substrates only found in organelles (*e.g.*, the chloroplast or mitochondrion).

Targeting recombinant proteins to the endoplasmic reticulum (ER) may be required to ensure proper folding.^849^ Proteins encoding a signal peptide, composed of a positively charged N-terminal region, central hydrophobic core, and a peptidase cleavage site, are co-translationally translocated into the ER.^850,851^ A tetrapeptide H/KDEL (histidine/lysine, aspartic acid, glutamic acid, leucine) motif in the C-terminus is used to retain proteins in the ER, which is a strategy for increasing their accumulation and avoid glycosylation.^852,853^ Plants target proteins to vacuoles using sequence-specific and structural-dependent vacuolar sorting determinants (VSDs), this includes the NPIXL/NPIR sequence found at the N-terminus.^854^ In the absence of an ER retention or other localization signal, proteins in the ER are secreted into the apoplast (extracellular space).^849^

The nuclear import of proteins is required for gene regulation and the import mechanisms are often highjacked by pathogenetic viruses and bacteria. Proteins-of-interest can be localized to the nucleus using nuclear localization signals (NLSs) from these sources, such as the Simian Virus 40 (SV40).^855^ These NLSs have to be attached to any proteins that are expressed outside of the nucleus so that they can be imported and bind to DNA to impart their effect. Such factors include repressors/activators as part of genetic circuits or Cas9 and related genome editing machinery.

Proteins are targeted to chloroplasts by N-terminal transit peptides that commonly contain hydroxylated amino acids (Ser and Thr) and lack acidic ones (Asp and Glu), resulting in a net positive charge.^856^ For example, the Rubisco small subunit N-terminal peptide and N-terminal peptide of the F_1_-ATPase β subunit are used to transport proteins to the chloroplast or mitochondrion, respectively.^857–860^ While transit peptides lack strict sequence motifs, specific amino acids residues within the N-terminus have been shown determine specificity between the chloroplast and mitochondrion.^861^

Mitochondria-targeting signals also have common structural features, notably a positively-charged amphiphilic α-helix, that can be used to design new targeting signals.^862,863^ To target the mitochondrion, peptides gleaned from *S. cerevisiae* CoxIV and *Arabidopsis* F1-ATPase pFAγ have been shown to function in a transient tobacco leaf assay and *Arabidopsis* protoplasts.^864,865^

Proteins can be targeted for secretion from plant cells. This pathway begins by translation into the ER and onto the Golgi complex, before proteins are secreted into the extracellular space, apoplast, or vacuole.^866^ Proteins are co-translationally directed into the ER by an N-terminal signal sequence: a variable 12-30 residue motif containing a short positively charged N-terminus, hydrophobic core, and polar C-terminus.^867^ The tag is proteolytically cleaved upon import.^867^ Once targeted to the endomembrane system, proteins will be secreted extracellularly and glycosylated by N-linked oligosaccharides.

###### 2.2.1.5.2 Protease Sites

It can be hard to control the ratios between proteins in a plant cell due to the noise of transcription and translation.^868,869^ Alternatively, multiple proteins can be fused to create a single large protein that is transcribed as a gene. The proteins are separated by cleavable peptide sequences. Therefore, after being cleaved, the proteins will be at a fixed stoichiometry in the cell. For example, placing the self-cleaving 2A peptide from the foot-and-mouth disease virus between two proteins led to a constant expression ratio between them.^870^

A similar approach is to use site-specific proteases to release individual components from a large polyprotein. Potyviruses, including Tobacco Etch Potyvirus (TEV), are the largest group of plant-infecting RNA viruses and are abundant in proteases that function in plants. While these proteases are widely used for engineering other organisms,^871,872^ they have not seen similar adoption for plant engineering. It was shown in *Escherichia coli* that 18 nitrogen fixation proteins from *Klebsiella oxytoca* can be compressed into 5 polyproteins separated by TEV protease cleavage sites.^873^

Fusing proteins into a single open reading frame also reduces silencing because only a single promoter and terminator are necessary to express multiple proteins. This effect was shown in the construction of New Golden Rice, where two carotenoid biosynthetic proteins were placed under the control of a single rice globulin promoter, which led to less silencing.^874^

##### 2.2.1.6 Plastid/Mitochondrial Genetic Parts

Plastids are of prokaryotic origin and share similar machinery for transcription and translation. Thus, many genetic parts designed for bacteria, including *Escherichia coli*, will function in the plastid. Because of these similarities, *Escherichia coli* has sometimes been used to prototype genetic parts before using them in a plastid. However, there are also many differences, such as sensitivity to supercoiling, different mRNA processing enzymes, and Shine-Dalgarno (SD) sequences.^875–879^ These differences complicate the use of a surrogate bacterial host to quantify how parts will perform in the plastid.

Chloroplast transformation protocols require time and labor-intensive tissue culture methods to regenerate transformants under antibiotic selection. There are also currently no transient plastid expression assays that can be used to screen synthetic constructs without regenerating full plants. This limitation makes it more difficult to build large part libraries in the context they would be used. Thus, there have been efforts to characterize part libraries using alternative systems that are compatible with high-throughput characterization.

Protoplasts could be used for plastid/mitochondria part characterization. Polyethylene glycol (PEG) treatment of protoplasts can introduce DNA into chloroplasts.^880^ Part activity can be measured using a GUS reporter.^880^ Cell-free protein synthesis (CFPS) is another approach, where DNA containing the parts is added to lysed isolated chloroplasts (Section 2.4.3).^881^ Part strength is characterized by measuring transcript or protein levels. CFPS and related systems have been widely applied to study the natural mechanisms of chloroplast transcription and translation.^882–885^ They have also been used to characterize small libraries of genetic parts, albeit described in the literature as being done for the purpose of understanding chloroplast biology as opposed to genetic engineering.

Another problem in designing large, multi-gene systems for plastids is that the repeated use of the same part leads to homologous recombination, which deletes regions of the DNA.^886,887^ Recombination frequency is a function of the length of the repeated sequences.^888^ For bacteria, the size of the repeat where deletion occurs with high probability is between 20 and 30 bp, but can be as short as 14 bp.^889,890^ In plastids, the precise number is not known, but repeated sequences of 100 bp have been observed to lead to excision.^891,892^ Therefore, avoiding recombination requires part libraries of sufficient size such that there are variants of sufficient sequence diversity to avoid homologous recombination, even if the part functions are themselves identical.^886,893,894^

###### 2.2.1.6.1 Transcriptional Parts

There are two RNAPs that drive transcription: 1. a prokaryotic-like plastid-encoded RNAP (PEP) that is directed to promoters with a σ factor or 2. a phage RNAP-like nuclear-encoded polymerase (NEP) that is expressed from the nucleus and localized to the plastid.^295^ NEP is largely responsible for transcribing chloroplast housekeeping and replication genes, including the expression of the *rpoB* operon encoding PEP. PEP has a wider range of gene targets, is responsible for expressing photosynthetic genes and those controlled by light, and interacts with a number of regulators, including those imported from the genome. Sometimes promoters respond to both RNAPs. For example, P_atpB_ contains motifs recognized and either can be deleted to create a promoter that only responds to one.^895^

PEP promoters share similar -10 and -35 consensus sequences with “housekeeping” *E. coli* σ70 promoters. *Escherichia coli* constitutive promoters often have an additional TG upstream of the canonical -10 sequence (an “extended -10 motif”). PEP-based plastid promoters also use this motif to obtain high transcription levels or a high background, such as the often-used strong *psbA* promoter.^896^ The strongest promoters are derived from the *rrn* operon (P_psbA_ and P_rbcL_). While P_psbA_ is *de facto* used as a constitutive promoter, it also is light sensitive due to sequence motifs upstream of the -35 motif. They can be removed to make the promoter more constitutive, but this results in a decrease in transcription by 30-fold.^897^

NEP is related to phage RNAPs, and uses similar short promoters.^883^ The tobacco plastid *accD* and *rpoB* genes are both exclusively transcribed by NEP. The minimal length of P_rpoB_ is a short 15 bp.^883^ Within NEP-dependent promoters, there is a short 3 bp consensus sequence: (T or C)AT.

In plastids, there has not been much work to create part libraries for genetic engineering. However, it has been a common exercise to make variants of native promoters to study chloroplast transcription, which effectively leads to small promoter libraries that can be exploited by engineers.^898^ These libraries can be generated by mutation, truncation, or deletion and usually contain about a dozen variants. Libraries of the following promoters have been made: P_rrn_,^882,899^ P_accD_,^900^ P_trnM_,^901,902^ P_atpB_,^903^ P_psbA_,^904^ P_psbD_,^905^ and P_rpoB_.^481^ Fusions between P_trnM_, P_rbcL_, P_atpB_, and P_psbA_ have also been measured.^906^ Other less characterized promoters, such as P_atpI_, P_atpH_, and P_psbB_ can also be used to obtain different levels of gene transcription.^907,908^

In non-green plastids, the expression of most plastid genes, especially photosynthesis-related genes, is downregulated at both the transcriptional and translational level.^909,910^ Two exceptions are *accD*, involved in lipid metabolism, and *clpP*, involved in the protein turnover pathway, which are both highly expressed. Transgene expression in amyloplasts was achieved using the maize P_clpP_ promoter and tobacco P_accD_ promoters, although transcription levels were lower than the tobacco ribosomal RNA operon promoter P_rrn_.^910–912^ Amyloplasts are responsible for the synthesis and storage of starch granules, typically found in roots and tubers.

###### 2.2.1.6.2 Orthogonal Transcriptional Regulation

Phage polymerases and prokaryotic σ factors can be expressed from the plastid genome or from the nucleus and targeted to the plastid.^913–915^ RNAP derived from T7 phage has been targeted to the chloroplast, where it transcribes T7 RNAP promoters.^916^ In the nucleus, it has been put under the control of promoters that are constitutive, light-regulated, or salicylic acid-inducible.^917,918^ Libraries of T7 RNAP promoters whose strength has been measured in *Escherichia coli* are available to control gene expression across orders-of-magnitude.^919,920^

The multi-subunit PEP interacts with σ factors, like its bacterial counterpart. It will also interact with some σ factors from bacteria that direct it to new promoter sequences. A chimeric σ factor comprising the C-terminal DNA-recognition domain of the *Escherichia coli* heat-shock σ^32^ factor and the N-terminal domain of a plastid σ factor SIG1 was expressed in tobacco chloroplasts.^914^ The chimera activated the *Escherichia coli* P_groE_, which shares no sequence identity with a chloroplast PEP promoter.

###### 2.2.1.6.3 Translational Parts

Translation has been hypothesized to play a larger role in plastid gene expression than transcription.^921^ While translation in plastids is similar to prokaryotes, there are important differences. In *E. coli*, nearly all mRNAs have an RBS containing a Shine-Delgarno (SD) sequence 4-9 nucleotides upstream of the translational start codon to recruit the ribosome.^922,923^ However, in plastids, the location of the SD sequence is more variable, ranging from the 2-29 nucleotides upstream of the start codon.^884,924–926^ Additionally, almost half of the protein-coding genes in tobacco plastids do not harbor a SD or SD-like sequence, but instead require the absence of secondary structure around the start codon or a nucleus-encoded *trans*-acting factor to initiate translation.^877,926–930^ Bacteria are sometimes used as surrogates to screen RBSs, but this is imperfect and leads to quantitative differences.^931^ Eukaryotic algae, such as *Chlamydamonas*, have very different RBS sequences than both plants and bacteria and therefore are not suitable hosts for the characterization of translation parts.^932^

The 5′-UTR derived from a phage T7 gene (*T7g10*), which contains a strong RBS, is the most commonly-used means to obtain high expression in the plastid.^933^ Coupling it with a strong promoter can lead to 23% of total cellular protein in tobacco.^934^ This strength can be tuned 100-fold by varying the sequence of the 5′-UTR.^884,926,935–945^ A plastid CFPS was used to measure a library of 23 5′-UTRs from different plant species, *Escherichia coli* phage T7, and synthetic RBSs^946^ and a library of 104 designed RBS variants that spanned a 1300-fold range of expression.^881^

There are a number of caveats in using these references to glean genetic parts for engineering. Other proteins encoded in the nucleus and transported to the chloroplast also regulate the maturation of plastid transcripts through post-transcriptional RNA processing, intron splicing, editing, and turnover.^947,948^ These factors may be dependent on various plant conditions, including the day-night cycle, season, or biotic/abiotic stresses. Also, while there are ranked lists of RBSs within each study, there has not been systematic comparison of their activities under identical conditions. Many of the studies were performed *in vitro* with reconstituted cell-free systems and the ranking may change in vivo or under different growth or environmental conditions. Finally, the use of some of these 5′-UTRs, not necessarily the strongest ones, have been observed to lead to growth defects or other undesired phenotypes, possibly due to the sequestration of native proteins.^949,950^

###### 2.2.1.6.4 mRNA Processing and Stability

When an operon is transcribed in bacteria, all of the encoded genes can be translated from the transcript. While this is possible in a chloroplast as well, it is more common that the operon is first cleaved into a set of mRNAs that each encode one gene. This processing has been observed to be important for obtaining high levels of expression.^237,951–954^ However, this effect has not been found to be important all of the time. For example, the maize *psb* operon can be translated from both polycistronic and processed mRNA.^955,956^ GFP has been expressed from the second position of an operon without transcript processing.^957^ Its expression level was found to be a function of the RBS strength controlling the first gene, as is the case for bacteria.

To divide engineered operons in plastids, the 50 nt intercistronic expression element (IEE) has been used. Adding IEEs in front of each gene in an operon is proposed to mediate its processing into stable and translatable monocistronic mRNAs.^937,958^ The most frequently-used IEE is a 50-bp minimal sequence from the *psbB* operon in tobacco.^951^ IEEs have been used to express multiple genes involved in different pathways, including to vitamin E,^237^ artemisinic acid,^952^ and to build multi-subunit structures, such as Rubisco^953^ and the carboxysome.^954^

Compared to the 5′-UTRs, the 3′-UTRs are thought to play more minor roles in regulating translation efficiency within plastids. The *E. coli rrnB* operon 3′-UTR confers a 4-fold higher abundance of *gfp* transcripts than *rbcL* and *rpoA* 3′-UTRs, but results in similar amounts of GFP accumulation.^959^ Exchange of the 3′-UTR of *psbA* and *rbcL* with that from *rpl32* increased the mRNA levels only 3-fold but did not increase translation efficiency.^935^

###### 2.2.1.6.5 Mitochondrial Genetic Parts

The inability to engineer plant mitochondrial DNA has negated the development of genetic part libraries. However, some information can be gleaned from the understanding of their genetics that could aid part design in the future. The mitochondrial genome does not encode any endogenous RNAP. Rather, its transcription relies on one or more nucleus-encoded phage-type RNAPs.^960–962^ Promoters that respond to the plastid NEP and mitochondrial RNAP have similar sizes, sequences, and core motif.^883^ Additionally, mRNAs from algae mitochondria usually have no 5′-UTRs and short 3′-UTRs^963–965^ and mRNAs from plant and yeast mitochondria have long 5′-and 3′-UTRs but lack SD-like sequences to recruit the ribosome.^966^ Mitochondrial mRNA undergoes a wide array of post-transcriptional processing steps to form mature mRNA, such as intercistronic RNA processing, intron splicing, and RNA editing. This process requires the coordination of many nuclear proteins.^270,967^ These factors serve as the bottleneck to developing genetic parts to regulate transgene expression.

Mitochondrial transformation is only possible in two unicellular organisms, the yeast *Saccharomyces cerevisiae*, and green alga *Chlamydomonas reinhardtii*. Much less progress has been made toward characterizing genetic parts to regulate gene expression within the mitochondria. Only two recombinant genes, an antibiotic-resistance gene and *gfp*, have been integrated into the algae mitochondrial genome.^968,969^ Their transcription was controlled by a native upstream bidirectional promoter.

More genetic parts have been used to regulate recombinant protein expression in yeast mitochondria. The RBS derived from the mitochondrial *COX2* gene was used to control the expression of the *Barstar* selectable marker gene or reporters (*gfp* or *nanoluciferase*).^970^ The mitochondrial *COB* RBS was used to control the expression of a reporter.^971,972^ To relocate a nuclear-encoded respiratory complex III subunit to yeast mitochondria, the *COX1* RBS was used.^973^

#### 2.2.2 Plant Metabolic Engineering

Plants shuttle the carbon obtained from CO_2_ into many primary and secondary metabolite pathways. Using genetic engineering, their metabolism can be redirected to a metabolite that is either itself a desired chemical product or is a precursor to a recombinant pathway. Metabolic enzymes in plants are distributed across the cytosol and organelles, including mitochondria and chloroplasts. Some metabolic reactions are only performed in a specific location, such as glycolysis in the cytosol and the TCA cycle in mitochondria. Metabolic enzymes also differ in their requirements for ATP, reducing power, oxygen tension, and co-factors, thus requiring that they be localized to an appropriate place in the cell.

Plant metabolic engineering has improved the agronomic traits of crop plants, for example through improvements to growth by increasing CO_2_ fixation, nutritional content, or pest resistance. Beyond food, plants can be engineered to produce other commercial compounds, including pharmaceuticals, biologics, nutritional supplements, fragrances, materials, and dyes.^974–976^ Entire books have been written about plant metabolic engineering;^977^ here, we focus on the more complex designs that combine techniques from Synthetic Biology.

##### 2.2.2.1 Increasing Production by Deducing Natural Networks

Manipulating a metabolic pathway requires first mapping out the participating enzymes. Plant metabolic pathways are large, highly interconnected, and compartmentalized across organelles. The enzyme genes are often distributed across chromosomes, as well as the plastid and mitochondrial genome, and can be difficult to find using simple bioinformatics annotation tools. Further, genes are not always turned on, rather they are regulated based on developmental stage, cell and tissue type, and environmental conditions.

Omics- tools can aid the mapping of plant metabolic networks. Missing enzymes within a biosynthetic pathway can be identified by clustering genes with similar RNA-seq expression profiles.^978–981^ This approach was used to deduce the pathways to the high-value anti-cancer drugs etoposide (in mayapple) and vinblastine/vincristine (in *Catharanthus roseus*).^978,979^ This approach will not work if only bulk samples are evaluated and the genes for a pathway are only transcribed in specialized tissues. Single-cell transcriptomics was performed on the needle cells that produce taxol in English yew in order to identify missing components of a 17-gene pathway to the precursor baccatin III.^982^

When the pathway is involved in a stress response, it can be discovered by stressing the plants and measuring gene expression using RNA-seq and metabolomics. For example, the complete pathway for the *Arabidopsis* defense molecule 4-hydroxyindole-3-carbonyl nitrile was uncovered by profiling the responses to different pathogens.^983^ Similarly, the pathway to falcarindiol was deduced by challenging tomato leaves with immune elicitors.^984^ A 16-gene pathway converting tyrosine and phenylalanine to *N*-formyldemecolcine, a precursor for the anti-inflammatory drug colchicine, was identified from spatially and temporally resolved metabolomics and transcriptomics data from autumn crocus and flame lilies and expressed transiently in *Nicotiana benthamiana*. To identify the pathway responsible for biosynthesizing the potent vaccine adjuvant QS-21 derived from the bark of Chilean soapbark tree *Quillaja saponaria*, transcriptomics data was used to identify genes that were both highly expressed in primordia tissue and coexpressed with the enzyme for the first committed step of the 20-step pathway.^985^ Once the full pathway was elucidated, it was transferred to tobacco via transient transformation to produce QS-21.

ML can predict metabolic pathways. PlantiSMASH^986^ and PhytoClust^987^ use hidden Markov models of plant enzyme families to annotate plant biosynthetic gene clusters using genomic and, optionally, transcriptional co-expression data. The plant-specific tool mApLe uses a ML model trained on protein amino acid composition and physicochemical properties to predict pathways and differentiate those in primary or secondary metabolism.^988^ A ML model was also used to predict whether *Arabidopsis thaliana* enzymes were involved in primary or secondary metabolism with 90% accuracy.^989^

When the pathway to a product can be deduced, there are several strategies to increase the titer. If the plant contains the enzymes to produce a molecule-of-interest, but they are not highly expressed under normal growth conditions, then natural regulators can be overexpressed. Alternatively, a gene that is not active can be copied to another location in the genome and placed under the control of synthetic genetic parts. Some metabolite precursors could be bottlenecks and their pool increased by overexpressing rate-limiting enzymes. This strategy was taken to optimize the mevalonate pathway in rice to overproduce carotenoid precursors to increase nutrient content.^990^

Genes can also be knocked down or knocked out to reduce the loss of carbon flux into competing pathways. To direct flax (*Linum usitatissimum*) to produce desirable antioxidants and polyunsaturated fatty acids, a native enzyme was repressed to redirect flux in the shikimic acid pathway away from flavonoids to these products.^991^ Enzymes can be knocked out using genome editing (Section 2.1.1). The first commercialized genome-edited plant were soybeans with increased oleic oil content, marketed as Calyno.^53^ TALENs were used to disrupt two fatty acid desaturase genes that reduced the content of linoleic acid, which is not shelf-stable and degrades quickly in a fryer. Similarly, opium poppies were modified with Cas9 to reduce the production of benzylisoquinoline alkaloids, including morphine and codeine, and reroute flux to a novel alkaloid.^992^ The medicinal plant *Atropa belladonna* produces the anticholinergic drug hyoscyamine, as well as two bio-active derivatives, anisodamine and scopolamine. To increase hyoscyamine production and remove potentially unwanted side-products, the enzyme responsible for modifying hyoscyamine was knocked out in *A. belladonna* (Figure 12A).^993^ Deletion of betaine aldehyde dehydrogenase in rice led to an increase of the desirable aromatic molecule 2-acetyl-1-pyrroline and development of fragrance in grains.^994^

**Figure 12:**
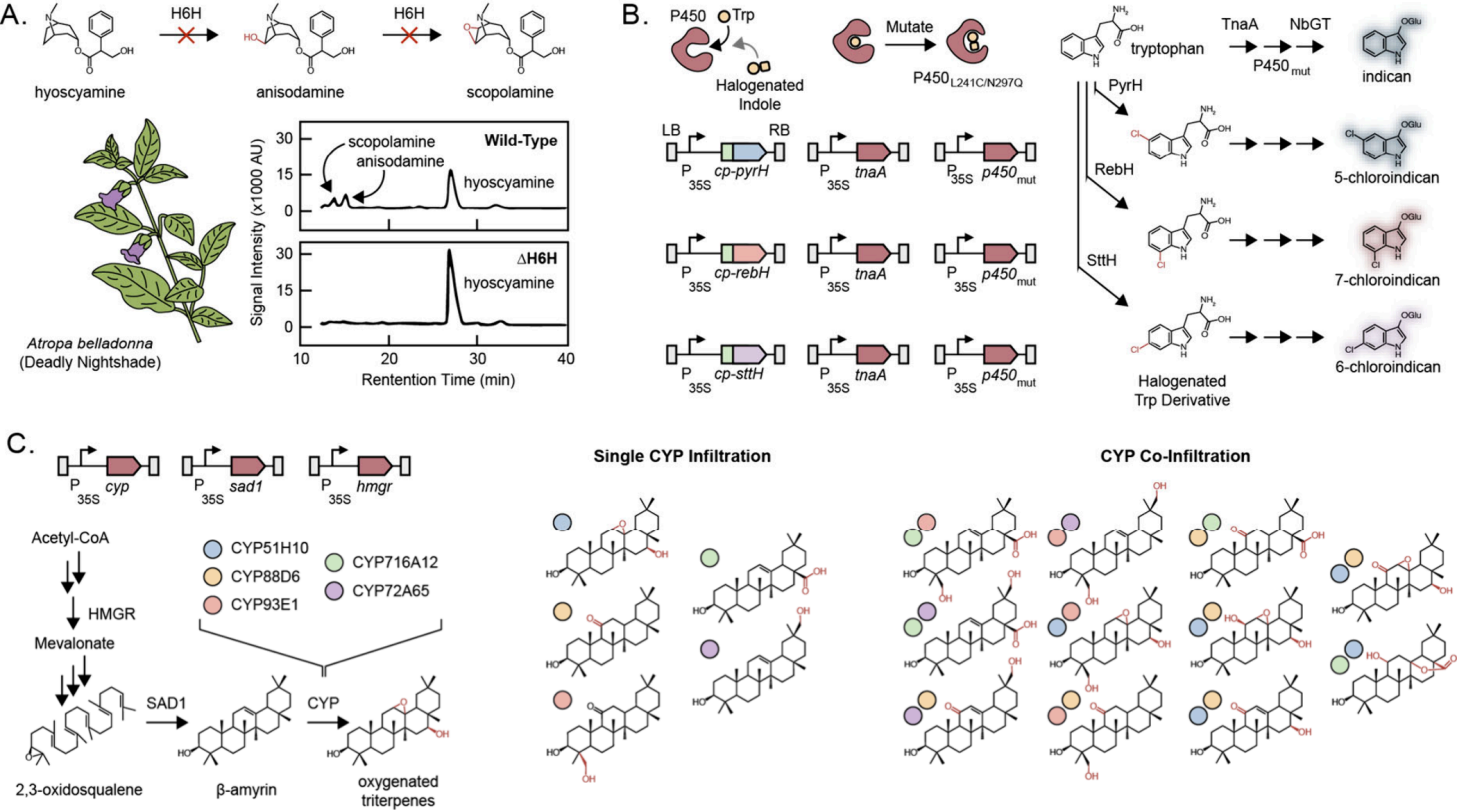
**Metabolic engineering in plants**. **A**. Redirecting carbon flux to increase production of the anticholinergic drug hyoscyamine.^993^ CRISPR-mediated disruption of the gene hyoscyamine 6β-hydroxylase (H6H) eliminated biosynthesis of the unwanted hyoscyamine derivatives anisodamine and scopolamine. Stable *A. belladonna* transformants were generated using *Agrobacterium*-mediated transformation, and alkaloid concentrations in *A. belladonna* roots were measured using HPLC in 25 cm tall plants. HPLC data reproduced with permission from ref 993. Available under a CC BY license. Copyright 2021, International Journal of Molecular Sciences. **B**. Producing new-to-nature halogenated indigo precursors in tobacco.^995^ The indigo precursor indican can be produced in tobacco by transiently expressing the bacterial tryptophanase, TnaA, and the human P450 2A6 monooxygenase along with the presence of a native tobacco enzyme glucosyltransferase, NbGT. Three microbial tryptophan halogenases (PyrH, RebH, SttH) chlorinate the indole moiety of tryptophan. The downstream wild-type P450 enzyme is unable to oxidize these chlorinated substrates, but a mutated version can accept the alternative substrates. Transient co-infiltration of tobacco leaves with combinations of a tryptophan halogenase, tryptophanase, and the mutated P450 enzyme produced precursors to three halogenated precursors that produce indigo dyes with altered colors. cp: chloroplast transit peptide sequence; LB: *Agrobacterium* left border region; RB: *Agrobacterium* right border region. **C**. Biosynthesis of new-to-nature triterpenes in tobacco.^996^ Overexpression of two enzymes, β-amyrin synthase (SAD1) and 3-hydroxy-3-methylglutaryl-CoA reductase (HMGR), in tobacco leaves increased the pool of the terpene scaffold β-amyrin, which can be tailored by cytochrome P450 (CYPs) to produce oxygenated triterpenes. Tobacco plants were co-infiltrated by *Agrobacterium* strains individually carrying Sad1, HMGR, and one of five different CYP plant species (oat, licorice, soybean, barrelclover). Different triterpenes were purified and identified through GCMS analysis of plants infiltrated with single or combinations of the CYPs. The enzymes responsible for producing a given molecule are indicated by adjacent colored circles, and chemical modifications are highlighted in red.

##### 2.2.2.2 Building Pathways to New Molecules

Plants can make new compounds by introducing enzymes from different organisms, either individually or assembling them into a synthetic pathway.^997^ These enzymes could modify a molecule naturally made by the plant to alter its function. For example, brassinin is an antifungal compound produced in many cruciferous crops. By expressing cytochrome P450 enzymes from different species, non-natural brassinin derivatives were produced that exhibited greater potency against pathogens-of-interest.^998^

Halogenation can increase the potency of some molecules and can be used as a reactive group for downstream chemical reactions.^999^ In tobacco, the antifungal potency of brassinin derivatives was increased through fluorination.^998^ Other examples are novel vibrant dyes (5-, 6-, and 7-chloro-indicans) that have been produced in tobacco by combining bacterial halogenases with a synthetic pathway comprising a bacterial tryptophanase, a human P450 mutated to tolerate halogenated substrates, and an endogenous glucosyltransferase (Figure 12B).^995^ A pathway to unnatural halogenated auxin indole acetic acid derivatives was assembled in *Nicotiana benthamiana* using bacterial halogenases, an *Arabidopsis thaliana* aminotransferase, and a *Pisum sativa* monooxygenase.^1000^

When designing a pathway to a new-to-nature compound, the artificial combination of enzymes from different sources often requires that they be promiscuous to new substrates. The more promiscuous they are, the more likely they can be “mixed-and-matched” to create large libraries of compounds. Terpenes are a great example of this capability. It is the largest class of natural products, many of which are produced by plants that have applications ranging from fragrances, fuels, pest repellants, and pharmaceuticals.^1001–1003^ Plants make high titers due to high concentrations of the precursor metabolites.^996,1004^ Diterpene scaffolds are made from the isoprene precursor by a pair of synthases. By combinatorically expressing 40 diterpene synthases sourced from ten plant species, 41 diterpenes were produced in tobacco that have not been observed in nature.^1005^ Additional chemical diversity is generated by the action of different modifying enzymes on the terpene scaffolds. By introducing combinations of five cytochrome P450s from different plant species into tobacco, over 40 oxygenated triterpenes, including several novel products, were produced (Figure 12C).^996^

Enzymes that are too specific to be used in an artificial pathway can be mutated to increase their substrate promiscuity. This approach improved the activities of enzymes on halogenated natural products. If halogenation occurs at an early pathway step, native enzymes may no longer work because their active site cannot accept the larger halogenated substrate. This problem occurred when halogenases were introduced into a medicinal plant (*Catharanthus roseus*) that produces monoterpene indole alkaloids, including the anticancer agents vinblastine and vincristine.^1006,1007^ A mutant had to be made to the downstream enzyme strictosidine synthase to accommodate halogenated substrates.

Sometimes the expression of heterologous enzymes leads to the modification of off-target metabolites, resulting in unexpected byproducts.^1008^ Such byproducts were observed when the marine bacterial β-carotene ketolase CrtW was expressed in rice and tobacco to produce the high-value carotenoid xanthin. In addition to the intended product, other carotenoids were formed because of the activity of CrtW on precursors outside of the xanthin biosynthetic pathway.^1009,1010^

##### 2.2.2.3 Interfacing with Chloroplast and Mitochondrial Metabolism

Chloroplasts and mitochondria are the “powerhouses” of the cell due to their role in generation of the energy carrier ATP. They are also central to the plant’s metabolism. Some metabolites are only produced in the chloroplast or accumulate there during photosynthesis, including some carbohydrates, essential amino acids, and lipids.^1011,1012^ In metabolic engineering projects, recombinant enzymes sometimes need to be targeted to chloroplasts to take advantage of their ATP and reducing power or if the precursor metabolite is only present there. Mitochondria operate the tricarboxylic acid (TCA) cycle and generate ATP through oxidative respiration. Chloroplasts have high O_2_ concentrations during the day when it is generated through photosynthesis, whereas mitochondria have a lower concentration where it is being consumed. This difference can be important to consider when expressing recombinant O_2_-sensitive enzymes. Finally, each organelle produces a suite of different co-factors, including metal clusters, that may be required by a recombinant enzyme.

###### 2.2.2.3.1 Chloroplast Metabolic Engineering

Because of their prokaryotic origin, it is potentially simpler to transfer operons or biosynthetic gene clusters from bacteria to plastids, as compared to the difficulty of moving multi-gene systems to the nuclear chromosomes. To make plants that produce light, the six-gene *lux* operon from *Photobacterium leiognathi* was expressed from the plastid genome.^1013^ Terpenoids can be produced using methyl-erythritol phosphate (MEP) precursors produced in the plastid.^1014^ The availability of MEP motivated the movement of entire prokaryotic biosynthetic gene clusters into the plastid genome to produce vitamin E, astaxanthin, artemisinic acid, and polyester polyhydroxybutyric acid (Figure 13A,B).^229,238,289,291,1015–1018^ Terpenoid titers increased when the MEP rate-limiting enzymes were overexpressed.^990,1019–1021^ In addition, the 6-gene cytosolic MEV pathway has been transferred to the tobacco plastid genome to increase terpene production.^1022^ Enzymes can be also expressed in the nuclear chromosomes and directed to the chloroplast by fusing them to transit peptides (Section 2.2.1.3). This fusion directed enzymes for the production of patchoulol, amorphadiene, and limonene from MEP in the chloroplast.^1014^

**Figure 13:**
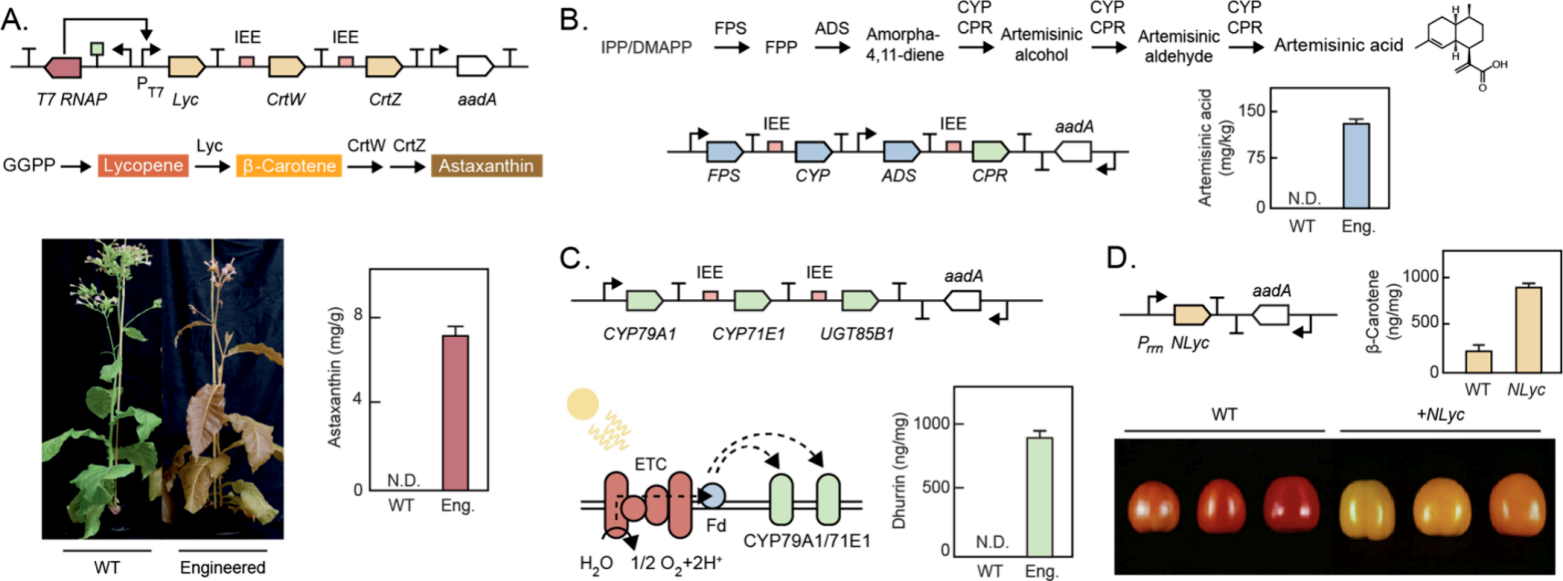
**Chloroplast metabolic engineering**. **A**. Tobacco engineered to produce pigments.^1018^ Engineered *Nicotiana tabacum* (Eng.) produces the antioxidant pigment astaxanthin after insertion of a synthetic operon expressing lycopene β-cyclase (Lyc) from daffodil and β-carotene ketolase and hydroxylase (CrtW and CrtZ) from the marine bacterium *Brevundimonas* sp. strain SD212. The operon contained IEEs (red square) for polycistronic expression and was expressed by T7 RNAP regulated by the theophylline riboswitch (green box). WT: wild-type. N.D.: no data. Images of tobacco plants reproduced with permission from ref 1018. Available under a CC BY license. Copyright 2022, Plant Physiology. **B**. Malaria drug production by tobacco.^952^ Transplastomic plants (Eng.) biosynthesize an artemisinin precursor through the introduction of a synthetic operon encoding the artemisinin acid pathway. IEEs (red half circle) were used to enable polycistronic expression. **C**. Insect repellent (dhurrin) production in tobacco.^1023^ Three genes from the dhurrin biosynthesis pathway in sorghum were expressed in engineered tobacco chloroplasts (Eng.). The P450 enzymes CYP79A1 and CYP71E1 were localized to thylakoid membrane. The P450s utilized electrons (dotted line) derived from the photosynthetic electron transport chain (red) mediated by ferredoxin (blue). The y-axes in the titer graphs are per kg of plant dry weight. **D**. Changing the color of tomatoes.^1032^ Expression of lycopene β-cyclase (*NLyc*) in tomato chromoplasts from a strong promoter (P_rrn_) converted lycopene into β-carotene. Transgenic tomato fruit producing β-carotene turns orange. Image of tomatoes reproduced with permission from ref 1032. Available under a CC BY license. Copyright 2009, Plant Physiology.

Many natural product pathways require enzymes whose redox chemistry needs a cellular source of electrons. Cytochrome P450 oxidases are examples, where electrons are obtained from a partner reductase that, in turn, usually obtains them from NADH or NADPH. They use this reducing power to introduce chemical moieties, such as hydroxyl groups, into the natural product. Plastids can redirect electrons from the photosynthetic electron transport chain to power recombinant enzymes, including cytochrome P450 oxidases. This electron source powered the synthesis of dhurrin, an insect repellent native to sorghum, that requires two cytochrome P450 oxidases and a UDP-glucosyltransferase (Figure 13C).^1023,1024^ In sorghum, the electrons are obtained from NADPH-dependent cytochrome P450 oxidoreductase, but this can be replaced by ferredoxin, an electron carrier in the photosynthetic electron transport chain.^1025^ The cytochrome P450 oxidases in the dhurrin pathway can be fused to ferredoxin and a tag that directs them to insert into the chloroplast thylakoid membrane.^1026,1027^

Terpene pharmaceuticals are often produced naturally by plants; however, either the titer is too low to be economical or the plant is not one compatible with agriculture. Therefore, these pathways have been moved into plants where metabolic engineering can be applied to increase titers. For example, the potent anti-cancer compound paclitaxel (Taxol) is naturally synthesized by Pacific yew tree (*Taxus brevifolia*) in the bark and needles, but is only produced in small quantities and puts pressure on a scarce natural resource. Therefore, the pathway was moved so that it can be produced by a crop plant or yeast.^1028,1029^ Its biosynthetic pathway is large, with at least 19 steps from the isoprenoid derivative GGPP, including nine cytochrome P450-catalyzed reactions.^1030,1031^ Due to the number of cytochrome P450 enzymes, it has been proposed that the chloroplast is the ideal compartment for synthesis.^1023^ To date, the most successful attempt to produce paclitaxel in a heterologous plant has involved targeting the enzymes that catalyze the first two reactions (taxadiene synthase and taxadiene-5α-hydroxylase) to the chloroplast and overexpressing DXS and GGPPS to overcome rate-limiting steps in precursor formation.^1020^

###### 2.2.2.3.2 Nongreen Chloroplast Metabolic Engineering

Recombinant gene expression in chloroplasts can lead to impaired photosynthetic activity and stunted growth due to resource competition.^1021,1023,1033^ These problems could be avoided by limiting expression to only occur in non-green chloroplasts.^235,1034^ These differentiated organelles have natural roles in metabolite production, including the sequestration of intermediate metabolites to avoid potential toxicity. For example, vanillin is produced in phenyloplasts where it is stored as a highly concentrated solid in vanilla pods.^1034–1036^ Amyloplasts occur in roots and store carotenoids and starch.^235^

Chromoplasts are differentiated chloroplasts that store high levels of carotenoids and this has been exploited in engineering projects. Enzymes can be targeted to the chromoplast by fusion with a transit peptide, as was done with phytoene synthase (CrtB) in tomato fruits to increase phytoene, lycopene, β-carotene and lutein production.^481^ Transplastomic expression of a daffodil lycopene β-cyclase was used to increase lycopene conversion to β-carotene in tomato fruits, driven by a strong chromoplast promoter (Figure 13D).^1032^ Further, methods to increase differentiation of chromoplasts have been developed. In some non-photosynthetic tissues (tomato fruit, potato tubers, etc.), overexpression of the cauliflower *OR* gene leads to greater production of chromoplasts.^1037–1039^ Chloroplast-to-chromoplast differentiation can be induced in leaf tissues with a burst of phytoene production (*e.g.*, through the expression of recombinant CrtB).^1040^

###### 2.2.2.3.3 Mitochondrial Metabolic Engineering

Mitochondria also have capabilities useful for metabolic engineering, including the sequestration of diverse metabolites, high reducing redox potential, and ATP availability. It remains intractable to transform plant mitochondria, but it is possible to direct enzymes there using transit tags (Section 2.2.1) or to edit the mitochondrial genome using base editors (Section 2.1.4). Here, we review some of the work in engineering yeast mitochondria for metabolic engineering projects that is likely to be transferrable to plants.

To utilize the yeast mitochondrial acetyl-CoA pool as a substrate to make different terpenes, enzymes from the MVA pathway and related terpene synthases, cyclases, and oxidases normally expressed in the cytoplasm were targeted to mitochondria.^1041,1042^ By increasing local enzyme concentration and limiting the loss of intermediates to competing pathways common to the cytoplasm, compartmentalization of the Ehrlich pathway into the yeast mitochondria increased isobutanol synthesis.^1043^ Overexpression of a bacterial lipoamidase in yeast mitochondria enabled *R*-lipic acid production by exploiting its pool of lipoate.^1044^

Due to oxidative respiration, the oxygen concentration of the mitochondrion is low. This feature can be exploited to host enzymes that are oxygen sensitive. One example is nitrogenase, the enzyme responsible for converting atmospheric nitrogen to ammonia, which is very sensitive to oxygen.^1045^ For this reason, nitrogenase proteins synthesized in the nucleus have been targeted to the mitochondrion of yeast using transport tags (Section 4.2).

##### 2.2.2.4 Synthetic Compartmentalization

Hijacking natural organelles, such as the plastid, for engineering purposes could impact the functions they need to perform for the plant. Further, they have complex structures and molecular compositions that are not fully characterized. Simpler synthetic compartments can be built inside the cell by expressing proteins or biosynthesizing lipids that self-assemble into a three-dimensional structure. Some of these approaches allow for the targeting of metabolites or proteins to the inside of the compartment. This approach can increase the concentration of a substrate, sequester toxic intermediates, or protect enzymes from inhibitors or non-optimal conditions, such as oxidative stress and pH fluctuations.

Some metabolic pathways in bacteria are compartmentalized in proteinaceous bacterial microcompartments that have pores through which substrate enter and products exit.^1046–1050^ The localization of enzymes to the compartment interior increases the concentration of the substrates and sequesters toxic intermediates.^1051^ Encapsulins are 25 - 40 nm microcompartments whose synthesis requires only one self-assembling capsid protein that has been moved from prokaryotes to yeast.^1052,1053^ They can increase enzyme stability by protecting cargo proteins from proteases in the cell. Enzymes have been assembled into large and porous aggregates by fusing them to protein-protein interaction domains.^1054,1055^ Doing so has the effect of increasing the local concentration of intermediates. This approach has been applied to increase the CO_2_ concentration for Rubisco (Section 2.2.3.b.).

Synthetic compartments have been used in plants to increase the titer of a metabolic product. Lipid droplets are subcellular compartments used to store lipids and other nonpolar molecules in plant cells and their formation is induced by membrane-bound proteins, such as oleosin.^1056^ Targeting an oleosin hydrophobic domain to tobacco chloroplasts resulted in the assembly of synthetic lipid droplets that accumulated biosynthesized terpenes.^1057^ When co-expressed with chloroplast-targeted squalene biosynthetic enzymes, the synthetic lipid droplets improved squalene titers by shielding the product from downstream pathways, preventing feedback inhibition of biosynthesis enzymes, and limiting toxicity. Essentially, this system performs an organic extraction inside of the plant.

##### 2.2.2.5 Directed Evolution

Directed evolution is a powerful means to alter properties-of-interest.^1058^ Iterative rounds of diversity creation and screening improve desired properties over generations. Directed evolution has been used to improve proteins, pathways, or even entire genomes (Section 3.2.6).^98,101,102^ Books have been written about the techniques and applications of directed evolution and here we just focus on some notable applications in plants.^98,101,102^

Directed evolution can be applied to improve the activity of a plant enzyme. For example, diacylglycerol acyltransferase 1 from oilseed rape was evolved for improved activity using mutagenesis and screening for triacylglycerol production.^1059^ Transient expression of the evolved gene in tobacco leaves led to elevated levels of triacylglycerol compared to leaves transformed with the native gene. Plant herbicide resistance can also be evolved using random mutagenesis and screening of individual genes, typically performed in yeast. Examples include the following: 1. durum wheat and barley glutathione transferases for butachlor resistance, 2. maize glutathione transferases for fluorodifen resistance, 3. rice and maize 4-hydroxyphenylpyruvate dioxygenases for pyrazoles resistance, and 4. glyphosate-resistance enzymes in maize and *Brassica*.^1060–1066^

When microbial enzymes or pathways are transferred to a plant, it can often require directed evolution to make them compatible. One example is the replacement of thiazole synthase because the plant version degrades following a single reaction cycle. In contrast, some microbial thiazole synthases can be recycled, but require the high substrate concentrations present in the microbe. To address this problem, a thiazole synthase from the anaerobic bacterium *Mucinivorans hirudinis* was evolved for aerobic activity and physiologically-relevant sulfide concentrations in yeast.^1067^ A second example is a bioluminescence pathway from the fungus *Neonothopanus nambi*, which was transferred to six different plants, including *Arabidopsis*, tobacco, poplar, *Petunia hybrida*, and *Chrysanthemum morifolium* (Figure 14).^1068^ The native *Neonothopanus nambi* luciferase and hispidin-3-hydroxylase had low activity outside of their native host. Thus, they were evolved using random mutagenesis to increase their stability and brightness. The evolved pathway produced visible luminescence in plants and is now commercially available as ornamental petunias.

**Figure 14:**
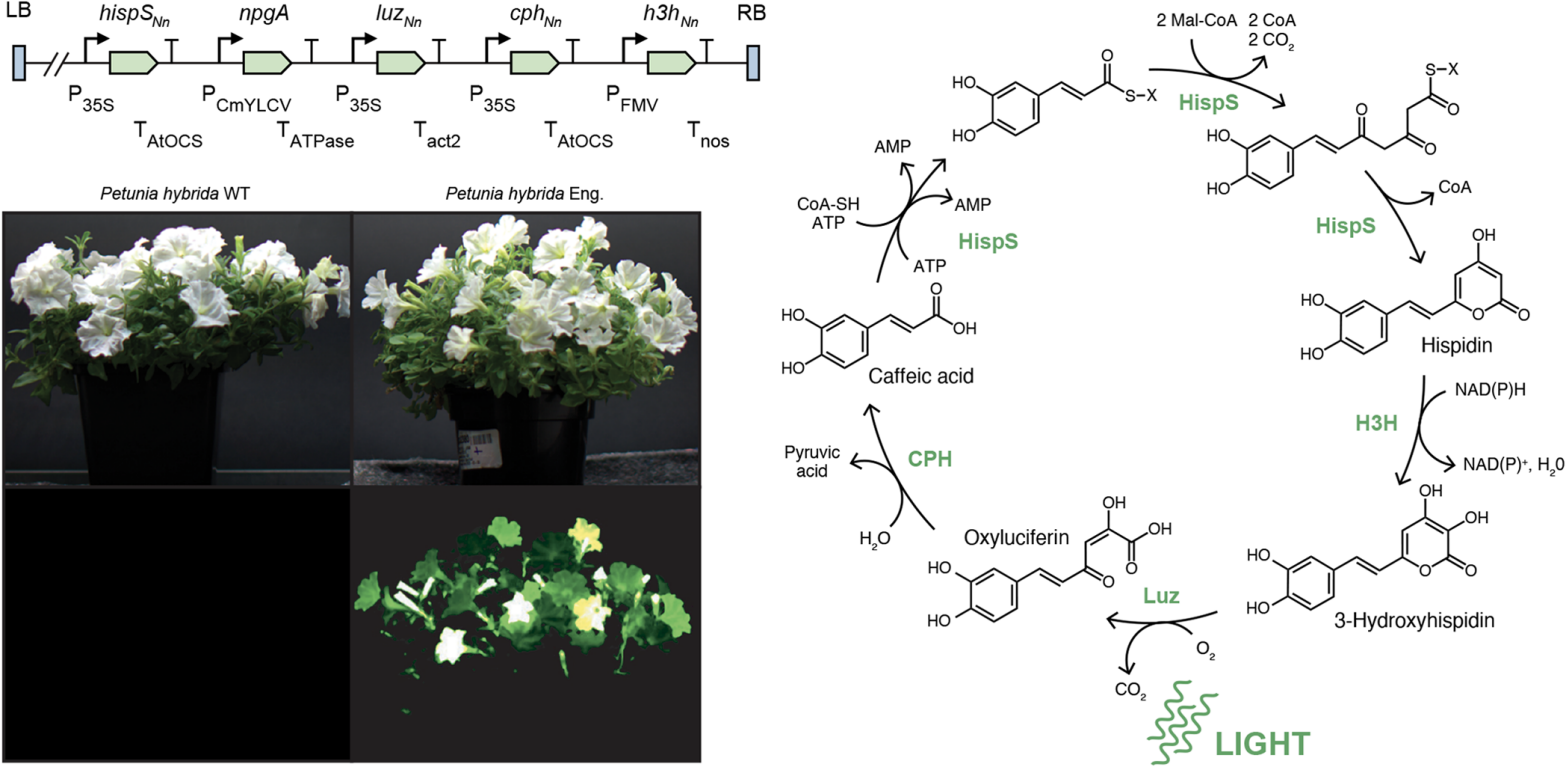
**Engineered plants that emit light**. A bioluminescence pathway from the fungus *Neonothopanus nambi* was evolved and then transferred to commercially and scientifically valuable plants.^1068^ The luciferase (*luz*) and hispidin-3-hydroxylase (*h3h*) genes required random mutagenesis and screening to improve heterologous expression, and the phosphopantetheinyl transferase (*npgA*) gene from *Aspergillus nidulans* was required for activation of the *N. nambi*-derived *HispS*. Ornamental petunias transformed with the evolved construct (Eng.) were visibly luminescent with the naked eye. LB: *Agrobacterium* left border region; RB: *Agrobacterium* right border region. Image of petunias and biosynthesis pathway reproduced with permission from ref 1068. Available under a CC BY license. Copyright 2024, Nature Methods.

Selections connect improved mutants to the survival of the host and can increase the number of mutants screened by orders of magnitude. Sometimes, the product of an enzymatic reaction is not easily connected to a screen or selection. Genetically-encoded sensors (Section 3.4.1) can be used to respond to the product and turn on a reporter or a gene required for survival.^1069–1071^ Continuous directed evolution strategies simultaneously perform diversification, selection, and amplification to increase throughput further.^100^ Phage-assisted continuous evolution (PACE) inserts the gene-to-be mutated into a bacteriophage genome lacking an essential replication gene.^1072^ A genetically-encoded sensor for the product can then be connected to replication.

Insecticide resistance has been improved using continuous evolution to increase potency or specificity for specific species.^1073,1074^ For example, Bt toxins with improved potency were evolved using PACE by making the expression of the gIII bacteriophage gene dependent upon the binding of Bt toxins to insect cadherin-like receptors (the insect target of Bt toxin).^1075^

Performing directed evolution *in planta* is good for screening for trait improvements that cannot be replicated *in vitro*, such as grain yield. *In planta* directed evolution has been carried out by targeting mutagenesis to a specific gene using CRISPR machinery (Section 2.1.1). To evolve herboxidiene-resistant spliceosome protein variants in rice, *SF3B1* was mutagenized by transforming a library of 119 sgRNAs that target all internal PAM sites to generate in-frame deletions or mutations.^103^ The library was transformed into embryonic calli and resistant transformants were selected through the addition of herboxidiene to the regeneration media. Multiplexed orthogonal base editor (MoBE) employs an sgRNA fused to a combination of RNA aptamers that recruit dual base editor proteins to perform site-specific base conversions on the plant genome.^1076^ This strategy was used to generate up to 27,294 combinations of edits in the rice acetyl-coenzyme A carboxylase gene to evolve haloxyfop resistance in transformed calli.

##### 2.2.2.6 Optimizing Photosynthetic Efficiency

The photosynthetic efficiency of a plant is calculated as the percent of light energy that is converted to chemical energy (glucose). The measured photosynthetic efficiency of crops is surprisingly low (1-3%).^1077^ Enhancing photosynthetic efficiency to increase agricultural productivity has been a longstanding goal of genetic engineering.^1078^ There are different strategies for how it could be achieved, from optimizing the components of native photosynthetic systems to constructing synthetic photorespiration bypass routes or entirely new carbon fixation processes that do not exist in nature.^1078–1081^ The maximum efficiency that could be achieved by a plant has been estimated to be 12%.^1082^ However, even a small change could have a big effect; it has been estimated that only a 0.1-fold increase in photosynthetic efficiency would increase crop yields by 50%.^1083^

###### 2.2.2.6.1 Conversion of a C3 to a C4 Plant

C3 and C4 plants are named according to the number of carbons contained in the first metabolic product after the fixation of CO_2_ by the enzyme Rubisco. In a C3 plant, Rubisco catalyzes the condensation of CO_2_ and ribulose 1,5-bisphosphate (RuBP) to produce glyceraldehyde-3-phosphate. Major C3 crops include tobacco, soybeans, and rice. The metabolic pathway for C4 plants (the Hatch-Slack pathway) is much more complicated. First, in mesophyll cells, CO_2_ is fixed by PEP carboxylase with phosphoenolpyruvate (PEP) to produce the 4-carbon oxaloacetic acid (OAA). These are metabolized to intermediates, which are imported into bundle sheath cells and decarboxylate to create high CO_2_ concentration around Rubisco. Rubisco produces phosphoglycerate (PGA), which diffuses back to the mesophyll cells. It is then reduced and diffuses back to the to the bundle sheath cells. Major C4 crops include corn, sugarcane, and switchgrass. The C3 plants are more ancient than C4 plants and tend to grow in cooler and wetter climates with moderate sunlight. C4 plants have a higher photosynthetic efficiency; while only 5% of global biomass, they fix 23% of the carbon.^1086–1088^ C4 plants are also more efficient at nitrogen utilization, water requirements, and are more productive at high temperature. Thus, the conversion of a C3 plant to a C4 plant has been a longstanding goal of plant genetic engineering, but it poses challenges due to the metabolic complexity and multiple compartments required for the latter.^1081,1089–1095^

A characteristic feature of C4 photosynthesis is the physical separation of carbon fixation and photosynthesis in mesophyll cells and bundle sheath cells, respectively (Figure 15A). This is achieved by cell type-specific expression of many enzymes, such as PEP carboxylase in mesophyll cells and Rubisco in bundle sheath cells, as well as morphological organization of these cells to facilitate carbon exchange referred to as the “Kranz anatomy”.^1089^ Achieving cell type-specific expression in C3 plants has been difficult. C4 photosynthesis genes experience complex regulation at the transcriptional, post-transcriptional, and translational levels and transferring their promoters into C3 plants usually results in loss of their cell-specific phenotype.^1096,1097^ Notable counter examples include mesophyll-specific expression of the maize PEP carboxylase promoter in rice and bundle sheath-specific expression of the *Flaveria* glycine decarboxylase complex P-subunit (GLDP) promoter in *Arabidopsis*.^394,1098^ Five C4 metabolism genes from maize, encoding the minimal set of enzymes required for a complete C4 cycle, were transferred to rice under control of four PEP carboxylase promoters from grass and the *Flaveria* (perennial herb) GLDP promoter (Figure 15B).^1084^ The enzymes were enriched in the anticipated cell types but displayed low levels of CO_2_ incorporation into C4 acids and no evidence of C4 decarboxylation, likely because they were shuttled to the citric acid cycle. A 276 bp CRE from *Arabidopsis* was shown to control bundle sheath cell expression and could be engineered for tunable expression.^1099^

**Figure 15:**
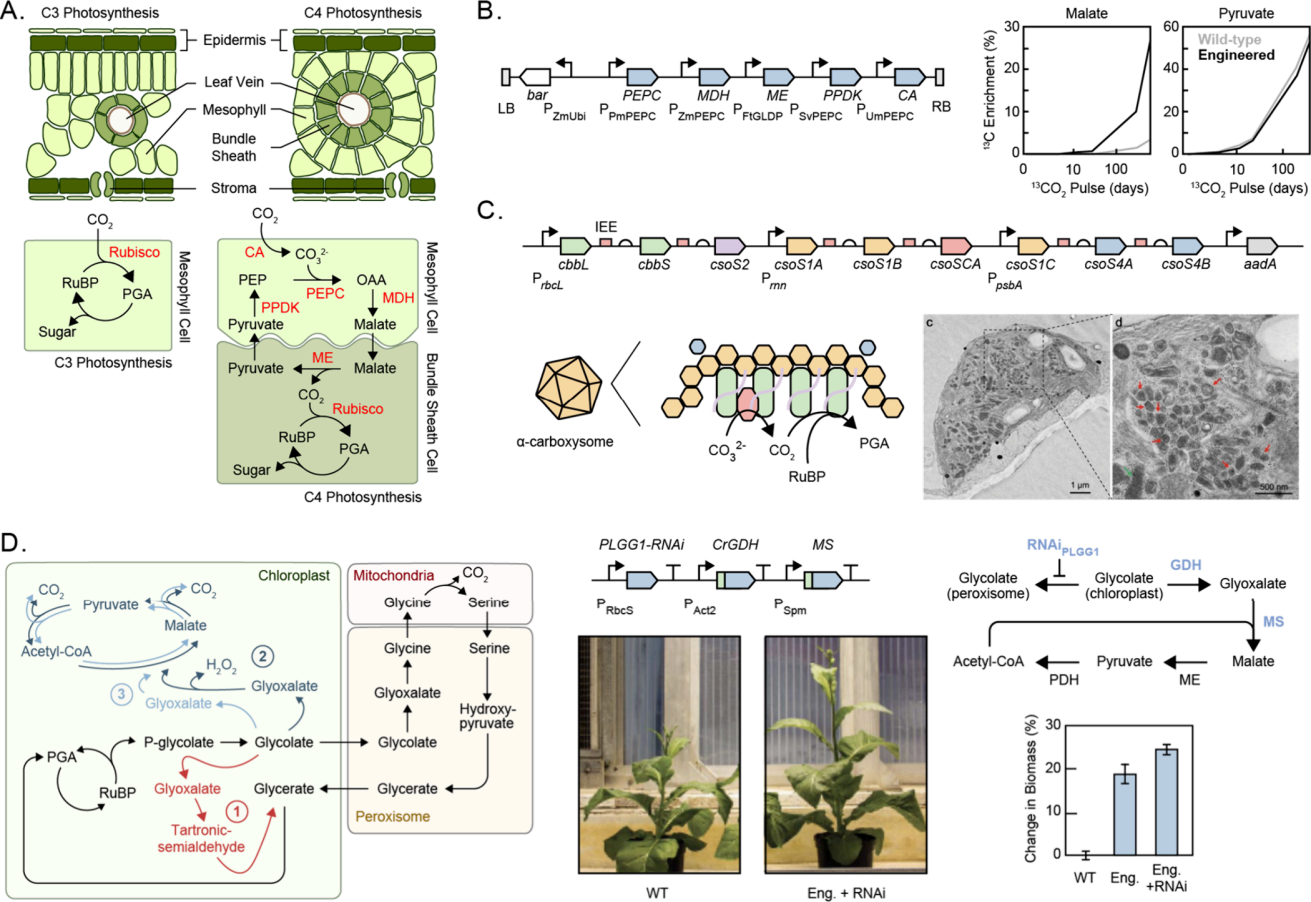
**Engineering Photosynthesis in Plants**. **A**. Schematic overview of C_3_ and C_4_ photosynthesis. Leaf anatomy differs between C_3_ and C_4_ plants, specifically C_4_ spatially separate carbon fixation in mesophyll cells and photosynthesis in bundle sheath cells through the characteristic “Kranz Anatomy.” Key enzymes in C_3_ and C_4_ photosynthesis are highlighted in red (see text for full enzyme names). **B**. Rice engineered to perform C_4_ metabolism.^1084^ The minimal set of genes required to perform C_4_ carbon cycling reactions were expressed in rice from mesophyll-specific (P_PEPC_) and bundle sheath cell-specific (P_GLDP_) (“Engin.”). Carbon labelling was used to trace metabolites and malate levels are increased while pyruvate remains the same, which is consistent with differences between C3 and C4 plants. **C**. Functional and structurally correct *Halothiobacillus neapolitanus* α-carboxysomes in tobacco plastids.^954^ The nine-gene cluster containing the minimal set of proteins for a functional α-carboxysome is shown (protein function is grouped by color). The cluster included *Halothiobacillus neapolitanus* Rubisco (CbbLS), a linker peptide (CsoS2), shell proteins (CsoS1A/B/C), shell vertex proteins (CsoS4A/B), and carbonic anhydrase (CsoSCA). Genes eithin operons were separated by IEEs (red squares) and RBSs (white half circles) to enable polycistronic expression. Images show TEM pictures of structurally accurate carboxysomes in chloroplast (red arrows). Image reproduced with permission from ref 954. Available under a CC BY license. Copyright 2023, Nature Communications. **D**. Engineering photorespiratory bypass routes in tobacco.^1085^ Three glycolate metabolism pathways were tested in tobacco (left). Pathway 1 involves a five-gene glycolate oxidation pathway from *Escherichia coli*. Pathway 2 contains three genes from bacteria and plants to convert glycolate to malate. Pathway 3 is similar to Pathway 2 but avoids hydrogen peroxide production in the first reaction. The best performing pathway (Pathway 3; “Eng.”) is shown on the right and includes a glycolate dehydrogenase (CrGDH) from *Chlamydomonas reinhardtii* and malate synthase (MS) from pumpkin targeted to the chloroplast using peptide tags (green boxes). Co-expressing dsRNA targeting the endogenous glycolate exporter (PLGG1) further increased biomass (Eng.+RNAi). Images of tobacco reproduced with permission from ref 1085. Available under a CC BY license. Copyright 2019, Science.

Another bottleneck is the relative lack of chloroplasts in C3 bundle sheath cells, which increase in quantity and size in C4 bundle sheath cells. Expressing the maize plastid biogenesis TF ZmGLK1 from either constitutive or cell type-specific promoters led to an increase in size and number of chloroplasts and mitochondria in rice bundle sheath cells.^1100^ Rare examples of single-cell C4 metabolism exist in nature that involve complex organization of organelles and metabolism that will likely be difficult to recapitulate in crops.^1101^

###### 2.2.2.6.2 Rubisco Engineering

Rubisco is the rate limiting step of photosynthesis because it has a slow turnover rate and can catalyze a reaction between RuBP and O_2_ instead of CO_2_, which leads to the release of three previously-fixed CO_2_’s, a process known as “photorespiration”.^1102^ A challenge with increasing its rate is that doing so often makes the enzyme more promiscuous, thereby reducing its efficiency because the increase also leads to more photorespiration.

Rubisco is composed of eight copies of the large subunit (RbcL) encoded in the plastid genome and eight copies of the small subunit (RbcS) encoded in the nuclear genome. Introduction of the RbcS from the C4 plant sorghum into rice increased the Rubisco catalytic turnover rate.^1103,1104^

Directed evolution could be used to improve the catalytic rate or selectivity against O_2_. Such experiments were performed using bacterial and algal Rubiscos. In *Escherichia coli*, a selection coupled Rubisco activity to cellular growth by engineering *Escherichia coli* to produce the substrate RuBP.^1105–1108^ In the absence of RuBisCO activity, toxic concentrations of RuBP accumulate and retard growth. Rubisco mutants were found with a 5-fold increase in specific activity compared to the wild-type enzyme.^1106^ A version of this selection assay was used to assay >99% of the single-amino acid mutants of Rubisco from *Rhodospirillum rubrum*, from which rare mutations were identified that improve CO_2_ affinity.^1109^ Another selection for Rubisco activity in *Escherichia coli* is based on deleting an enzyme required for glycolysis (glyceraldehyde-3-phosphate dehydrogenase), which is rescued by active Rubisco.^1110,1111^ This selection was used to find *Synechococcus* Rubisco mutants with improved stability.^1112^ Directed evolution of archaeal Rubisco, which serves a non-photosynthetic role in archaea, increased its catalytic rate and CO_2_ specificity.^1113^ It supported photoautotrophic growth in tobacco at elevated CO_2_ concentration. A historical challenge with evolving plant Rubiscos has been the inability to functionally express them in a recombinant model organism. This issue was overcome in *Escherichia coli* by co-expressing five chaperones with *Arabidopsis* Rubisco, but this has not yet been applied to evolve higher activity.^1114,1115^

The overexpression or transfer of proteins that aid Rubisco activation or assembly can also increase the photosynthetic rate of plants. Rubisco activase (Rca) is a chaperone that works by removing chemical moieties that block its active site.^1113^ Replacement of *Arabidopsis* Rubisco activase (Rca) with a thermostable version enhanced the photosynthetic rate at higher temperatures.^1116^ Rubisco accumulation factor 1 (RAF1) is present across photosynthetic microbes and plants and aids RbcL assembly.^1117^ Co-expression of *Arabidopsis* RbcL with RAF1 in tobacco leads to a 2-fold increase in photosynthetic CO_2_ assimilation rate and plant growth.^1118^ The abundance of Rubisco was also increased in maize by overexpressing its subunits and RAF1 in the nucleus and targeting them to the chloroplast, leading to a 15% increase in CO_2_ assimilation.^1119^ The proteins were expressed in the nucleus due to the inability to manipulate the maize chloroplast genome (Section 2.1.4).

###### 2.2.2.6.3 Increasing CO_2_ Concentration

One way that C4 plants improve the photosynthetic rate is by increasing the concentration of CO_2_ around Rubisco, which also improves selectivity against O_2_. In C4 plants, the biology is complicated, so other mechanisms have been moved from non-plants to plants. Cyanobacteria use carboxysomes, 100-400 nm protein microcompartments, to increase the concentration of CO_2_ around an encapsulated Rubisco. Carboxysomes resist CO_2_ efflux and carry the enzyme carbonic anhydrase, which can rapidly convert aqueous bicarbonate (HCO_3_^–^) into CO_2_, leading to a buildup of internal CO_2_. Expressing cyanobacterial Rubisco and an internal carboxysomal protein in tobacco plastids led to the formation of protein complexes capable of carbon fixation and mimicked early intermediates of carboxysome assembly.^953^ Only two shell proteins were required to be expressed in tobacco to self-assemble and encapsulate cyanobacterial Rubisco.^1120^ The transfer of nine carboxysome genes from *Halothiobacillus neapolitanus*, including shell proteins, Rubisco, and carbonic anhydrase, led to functional carboxysomes in tobacco plastids (Figure 15C).^954^ However, the engineered plants required elevated CO_2_ levels for photoautotrophic growth. To assemble fully functional carboxysomes in C3 plants, additional shell proteins, chaperons, bicarbonate transporters, will be necessary.

Eukaryotic algae use a pyrenoid organelle containing a transporter of HCO^3–^/CO_2_/CO_3_^2–^ to increase the concentration of CO_2_ around Rubisco.^1121–1128^ The pyrenoid has an intricate internal structure that forms due to phase separation of a liquid-like condensate. This structure allows a rich concentration of Rubisco, which moves dynamically within the pyrenoid. It serves to concentrate CO_2_ around the structure, which is further trapped by a thick starch sheath. It has been postulated that their transfer into land plants could increase photosynthetic efficiency. To this end, the protein responsible for the condensate from algae (*Chlamydomonas reinhardtii*) was moved with a plant-algal hybrid Rubisco into Arabidopsis chloroplasts, where is formed an organelle with similar properties.^1129^ Membranes span the internal structure of the pyrenoid that are important for its activity. Two algal proteins have been expressed in Arabidopsis and shown to form similar membrane structures.^1130^ These advances are key steps toward moving the entire functional pyrenoid to crops to improve photosynthetic efficiency.

###### 2.2.2.6.4 Metabolic Engineering

The CO_2_ fixation rate can also be improved by rebuilding photosynthetic carbon metabolism.^1081^ The natural photorespiration pathway recycles the Rubisco toxic byproduct glycolate back to the Calvin cycle, but at the expense of energy dissipation and net loss of fixed carbon and nitrogen in mitochondria. Shunt pathways that completely restrict glycolate oxidation in the plastid can increase the CO_2_ concentration around the Rubisco enzyme and thus enhance photosynthetic efficiency.^1131,1132^ Three alternative photorespiratory pathways composed of ten nucleus-encoded enzymes from *Escherichia coli*, plants, and green algae were directed to the tobacco plastid (Figure 15D).^1085^ This feat required balancing gene expression by selecting different promoters and the use of RNAi to downregulate the native glycolate exporter to limit flux into the native photorespiration recycling pathway. It led to a 20% increase in biomass in field trials. In rice, three nuclear-encoded rice enzymes (glycolate oxidase, oxalate oxidase, and catalase) were redirected to the chloroplast using a transit peptide to completely oxidize glycolate within the chloroplast.^1133^ This led to an increase in the photosynthesis efficiency as well as biomass and grain yield in the field.

Artificial CO_2_ fixation routes can be constructed by combining enzymes sourced from different organisms. More than twenty theoretically-feasible synthetic CO_2_ fixation routes have been proposed.^1134^ One route, referred to as the CETCH (crotonyl–coenzyme A (CoA)/ethylmalonyl-CoA/hydroxybutyryl-CoA) cycle was built by combining the enzymes *in vitro*.^1135^ The pathway encompassed 17 enzymes originating from nine organisms.^1135^ It included an enoyl-CoA carboxylases/reductase (ECR) with the highest carboxylase activity measured (37-fold higher than Rubisco).^1136^ The CETCH cycle enzymes, along with an additional *E. coli* glyoxylate/hydroxypyruvate reductase and spinach thylakoid membranes, were encapsulated in a water-in-oil droplet.^1137^ The resulting system can use light energy to convert CO_2_ to glycolate. The malonyl-CoA-Oxaloacetate-Glyoxylate (MOG) cycle mimics many of the steps naturally found in the C4 cycle and could require as few as four enzymes to implement.^1136^ However, it produces glyoxylate as a terminal product, which would need to be integrated into metabolism.

##### 2.2.2.7 Toward Hybrid Vigor: Engineering Apomixis

“Hybrid vigor” is when the offspring of genetically distinct parents can exhibit superior traits, such as improved yields or stress resistance, as compared to either parent. The challenge is that the beneficial traits seen in the initial hybrid generation are often lost in later generations due to genetic segregation. Therefore, the parents must be crossed each time to make new seed because the seed of the offspring will not have the desired traits. One strategy around this costly and time-intensive process is to harness apomixis, which is a form of asexual reproduction where seeds are produced clonally without fertilization. This mechanism results in genetically identical offspring with the parents. Several plant species naturally exhibit apomixis, notably grasses and fruit trees. If apomixis could be introduced into hybrid crops, it would allow for the indefinite propagation of hybrid seeds. Synthetic apomixis strategies mimic the process of natural apomixis and involve three discrete developmental steps: bypassing meiosis (*MiMe*), generating an embryo from the unfertilized egg cell (parthenogenesis), and autonomously developing an endosperm.^1138^

To avoid genetic recombination and chromosome reduction, female reproductive cells can be reprogrammed to undergo *Mitosis instead of Meiosis* (*MiMe*).^1139^ MiMe clonally generates diploid gametes genetically identical to their mother cells rather than genetically diverse haploid gametes. To achieve MiMe, proteins responsible for coordinating three critical steps in meiosis were inactivated. First, chromosome recombination was prevented by inactivating a member of the recombination initiation complex (*SPO1-1, PRD, or PAIR1*).^1140,1141^ Next, a meiosis-specific cohesion subunit protein (*REC8*) involved in sister chromatid migration was disabled, leading to mitosis-like chromatid separation.^1140^ Finally, a positive regulator of the second meiotic cell division (*OSD1, TAM, or TDM1*) was knocked out.^1139^

The diploid female gametophyte from the *MiMe* process must be “activated” to form a functional embryo. In most plants, this is mediated by proteins found exclusively in pollen-derived sperm cells.^1142^ During synthetic apomixis, the *MiME* female gamete must either fuse with a sperm cell and eliminate the unwanted male chromosomes or “activate” itself in the absence of a sperm cell through the process of parthenogenesis. Pollen produced by plants expressing an engineered variant of the centromere protein (*CENH3*) or possessing an inactivated phospholipase gene (*MTL/PLA1/NLD*) produce zygotes without a paternal genome.^1143–1146^ However, these plants generally suffer from low fertility. Instead, through parthenogenesis, the unfertilized egg cell can develop directly into a multicellular embryo by expressing proteins that regulate embryo formation. Parthenogenesis was obtained by expressing the parthenogenetic trigger protein *BABYBOOM1* (*BBM1*)^1147^ or the dandelion parthenogenetic *PAR* gene from egg cell-specific promoters.^1148,1149^ Finally, embryos require an endosperm for nutrients, which is normally formed after a sperm cell fertilizes a maternal diploid central cell. Many naturally apomictic plants still require fertilization of the central cell, which is undesirable to produce clonal seeds.^1142^ In *Arabidopsis*, mutating epigenetic regulators of auxin biosynthesis (*FIS-PRC2*) leads initiation, but not completion, of autonomous endosperm development.^1138^

Currently, synthetic apomixis has only been demonstrated in *Arabidopsis* ^1144^ and rice.^1145,1147,1148,1150,1151^ Crossing *Arabidopsis* plants carrying *MiMe* mutations with plants expressing *CENH3* variants produced >34% clonal seeds.^1144^ In rice, synthetic apomixis was achieved using a single construct, which simultaneously inactivated three *MiMe* genes (*PAIR1*, *REC8*, and *OSD1*) using CRISPR/Cas9 and expressed *BBM1* from an egg cell-specific promoter.^1150^ After three generations, >95% of seeds were clonal progeny.

### 2.3 Genetic Control of Pests

Pests remain a blight to agriculture, resulting in large losses.^1152,1153^ Weeds can reduce yields 50-90% in the U.S. without the use of herbicides and 5-10% with them. The most common trait added to crops is herbicide resistance. It allows the herbicide to be sprayed to kill weeds without the crop; most famously, a trait protects against glyphosate (Roundup) allowing it to be used without killing the crop. However, weed herbicide resistance is rapidly spreading. Insects are another blight that accounts for 10-25% of global losses annually. The fall armyworm alone is responsible for over $13 billion in destroyed crops (maize, rice, sorghum, wheat, and sugarcane), can migrate up to 500 km a night, and is rapidly evolving resistance against pesticides. Vertebrate pests are also a major problem; rodents can lead up to 20% of losses of stored grain.^1154^ Chemical treatments of plant, insect, and vertebrate pests are losing efficacy, while also contributing to animal/human health problems and ecological damage.

Genetic controls offer an alternate management strategy for pests over chemical or mechanical approaches. The pest, or microbes inhabiting it, are genetically engineered and released into the wild to induce a change in the population. It could be to cause a decline, pushing the pest to extinction, or to introduce a new function to sensitize it to traditional controls (*e.g.*, agrochemicals) or remove genes encoding human allergens. Enclosed laboratory experiments suggest that the concept can work, driving a trait through a population. However, the release of organisms into the open ecosystem will face regulatory and social hurdles. They also can take a long time to take effect – even decades – and the pests can evolve in the field and break the genetic control.

#### 2.3.1 Manipulating Insects through Their Microbiomes

Insect behavior and biology can be controlled by altering the microbes that inhibit them.^1159–1163^ Genetically tractable microbes stably colonize all insects, including those of agricultural importance, including bees, tsetse flies, leafhoppers, aphids, and western flower thrips. The microbial genomes can be modified to contain recombinant genes that are expressed in the context of the insect. For example, it could encode a dsRNA that enters the insect and knocks down insect genes through RNA interference (RNAi). Microbes producing dsRNA can be used to kill pests. For example, to kill western flower thrips (a crop pest), bacteria were isolated from the insect and engineered to constitutively express dsRNA targeting the insects’ alpha-tubulin, which kills the larvae.^1164,1165^ The engineered bacteria can be formulated as a spray. Aphids are a major burden to agriculture and a bacterium in its microbiome was engineered to include an inducible system for recombinant gene expression.^1166^

This approach can also be used to save beneficial insects that are facing population declines.^1167^ Bees essential for pollination of crops are suffering from colony collapse disorder. One cause is the Varroa mite, which feeds on bee “blood” and transmits damaging viruses. To address this blight, the honey bee gut symbiote *Snodgrassella alvi* was engineered to induce an RNAi response by targeting genes in the bee host, as well as sequences from a honey bee virus and the *Varroa* mite (Figure 16A).^1155,1168–1170^ Bees inoculated with *Snodgrassella alvi* expressing dsRNA targeting the insulin receptor InR1 experienced increased feeding behavior and physiological changes to sugar metabolism. The engineered symbiotes were also able to improve bee mortality when expressing dsRNA targeting genes specific to the Deformed Wing Virus or *Varroa* mite.

**Figure 16:**
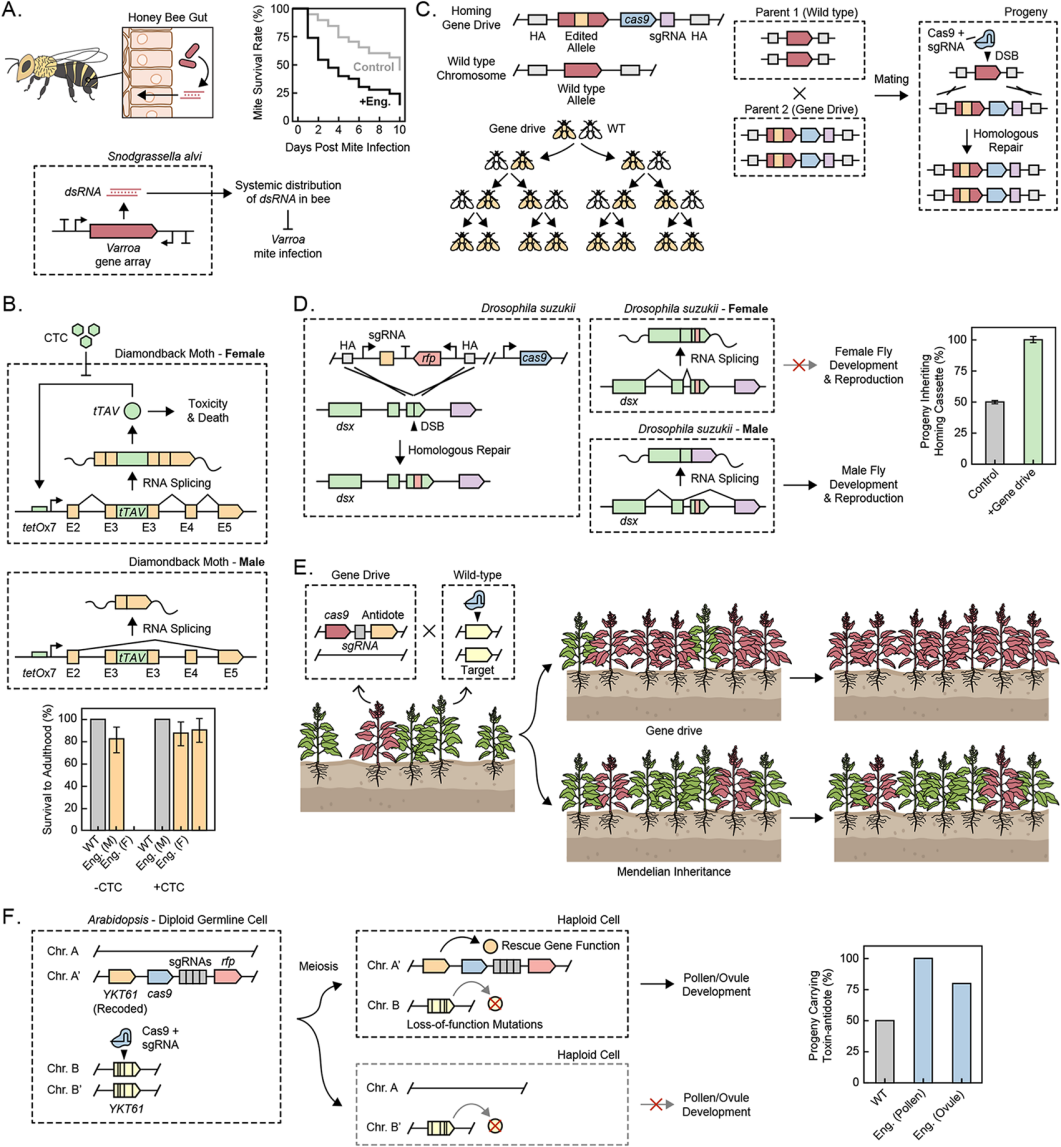
**Genetic approaches toward controlling agricultural pest populations**. **A**. Honeybee gut microbe engineered to target bee parasites.^1155^ The honeybee symbiotic gut bacterium, *Snodgrassella alvi*, was engineered to produce dsRNA to induce RNAi in parasitic mites by expressing an array of 14 concatenated sequences from essential genes in *Varroa* mites using flanking inverted constitutive promoters. Fewer *Varroa* mites survived in hives of honeybees inoculated with the engineered *S. alvi* (Eng.) than in hives inoculated with *S. alvi* expressing only *gfp* (Cont.). **B**. Inducible sex-specific lethality in diamondback moth (*Plutella xylostella*).^1156^ A tetracycline-repressible activator (tTAV) was inserted within the E3 exon in the doublesex (*dsx*) gene. Sex-specific RNA splicing causes the E3 exon and tTAV to be spliced out in males, resulting in normal function. In females, tTAV is maintained in the transcript after RNA splicing, which induces further *dsx*-*tTAV* expression caused by *tetO* sites upstream of the promoter. The resulting positive feedback loop causes high levels of tTAV expression, toxicity, and larvae death. Feeding larvae the tetracycline analog chlortetracycline (CTC) deactivates tTAV, preventing positive feedback. **C**. Schematic of a homing gene drive spreading a genetic edit through an insect population. A homing gene drive is composed of a site-specific endonuclease (Cas9-sgRNA) and rescue template carrying the intended edit (edited allele) and homologous sequences (homology arms; HA). When an individual carrying the gene drive mates with the wild-type population, the homing endonuclease creates a double-stranded break, which is repaired by the chromosome copy carrying the gene drive through homologous recombination and causes the spread of the gene drive to the wild-type locus. Theoretically, the homing gene drive is adopted by all progeny of a mating pair, resulting in it becoming increasingly prevalent in the population following each generation. **D**. A CRISPR-based homing gene drive for population suppression in the agricultural pest *Drosophila suzukii*.^1157^ A gene drive composed of a fluorescent marker, sgRNA, and homology arms was targeted to the sexual development gene *dsx*. The cas9 gene was expressed from a separate locus in the lab *D. suzukii* population to ensure biocontainment. Insertion of the gene drive within a female-specific *dsx* exon caused gene disruption, infertility, and development deformities in female offspring. Male flies splice out the gene drive, resulting in normal development and enabling the spread of the gene drive throughout the population. To quantify inheritance, female flies expressing only cas9 were crossed with either male flies carrying the gene drive (+gene drive) or flies lacking the gene drive (control). **E**. A schematic of using a gene drive to manage weeds. A field could be seeded with weeds carrying a gene drive, which would spread over multiple seasons to fix the introduced genetic allele in the population. **F**. A toxin-antidote gene drive in *Arabidopsis*.^1158^ A gene drive was constructed in *Arabidopsis* by expressing a toxin (Cas9 and array of sgRNAs) targeting the essential *Arabidopsis* gene *YKT61* and its antidote, a recoded *YKT61* gene unable to be targeted by Cas9. Expression of Cas9 and sgRNAs in germ line cells deactivates endogenous *YKT61* genes. Following meiosis, only pollen and ovule cells carrying the gene drive and expressing the recoded YKT61 gene are viable. WT *Arabidopsis* plants either were fertilized by pollen from T3 plants carrying the gene drive (Eng. (Pollen)) or were used to pollinate T3 plants carrying the gene drive (Eng. (Ovule)). A WT heterozygous allele is predicted to be transmitted to half of the progeny.

Bacteria expressing dsRNA can also be used to improve the resilience of native strains against bio-control treatments. For example, western corn rootworms can decimate corn fields and one way to treat this is through the use of insect-killing nematodes. However, nematode treatment can lead to rootworms accumulating and processing plant benzoxazinoids, leading to the suppression of the beneficial symbiont *Photorhabdus* spp. To overcome this problem, symbiotic *Photorhabdus* strains were evolved to have increased resistance against benzoxazinoid-defense systems.^1171^

#### 2.3.2 Genetic Control of Insects

The most common genetic approach to insect control is to release an engineered version of a particular sex that causes non-viable offspring when they mate with wild members of the opposite sex. The first approach was to induce sterility by irradiating the insects, most commonly males.^1172,1173^ The males are released and mate with wild females, leading to sterile offspring. This approach was first used in the 1950s in the United States to eradicate the screwworm fly. Subsequently, it has been used globally to suppress outbreaks of agricultural pests. It is expensive and requires infrastructure for the continuous release of irradiated insects and reinvasion from untreated areas is a problem. In addition, the random effects of irradiation are not predictable and can lead to males with lower fitness in the wild.

Several targeted genetic approaches have been taken to have the same effect, but in a more controlled way. Insects can be genetically modified to carry a “self-limiting” gene that causes offspring to die. Often, this trait is carried by males that, upon release, mate with wild females and the gene kills the female offspring. This approach was taken to suppress the Mediterranean fruit fly (*Ceratitis capitata*), which causes substantial global crop damage, affecting over 300 types of fruits, vegetables and nuts. Female-specific mRNA splicing was used to disrupt a transcript encoding a recombinant activator (TetR) in males, but not females.^1174^ When expressed in females, the activator can turn on genes that lead to lethality. TetR is inactivated by tetracycline and when this molecule was present in the diet, both males and females survive. A similar system was developed for the Diamondback Moth (*Plutella xylostella*) and Pink Bollworms (*Pectinophora gossypiella*) (Figure 16B).^1156^ Oxitec has been working to bring these engineered insects to market and has demonstrated effectiveness in closed settings and have entered field trials.^1175–1179^

#### 2.3.3 Synthetic Gene Drives

Classic or “Mendelian” genetics is taught in elementary school biology, where the offspring have a 50% chance of getting a trait from either parent. However, there are also natural selfish elements whose biochemistry drives them to be acquired by the offspring with higher probabilities.^1180–1182^ These systems have studied as natural phenomena and have been harnessed in synthetic systems.^1183^ Harnessing them to drive an arbitrary gene or mutation through a population has proven difficult because their biochemistry and complex regulatory pathways are difficult to control.

Synthetic gene drives fast forward the movement of a gene through a population as compared to what could occur due to natural evolution.^1184^ The rise of genome editing tools (Section 2.1.1) has made it easier to target specific alleles in the chromosome. The first synthetic gene drive was based on a homing endonuclease that induced a double strand break at a specific location in a chromosome of *Drosophila melanogaster* and then DNA repair via HDR catalyzed the transfer of the target gene to the site.^1185,1186^ The gene drive works by having the nuclease encoded with an attached gene in a chromosome that is inherited by the offspring. During reproduction, the gene is inherited as in Mendelian genetics, but then the nuclease inserts the gene into the other chromosome. Thus, its inheritance in the next generation is assured. The limiting problem was that meganucleases are difficult to reprogram to target arbitrary sites in the genome. The ability to target specific alleles was enabled by TALENs and Cas9. The resulting gene drives radically improved in effectiveness and flexibility.^1187,1188^ Since the original homing-based gene drive was developed, other designs have been developed to be spatially limiting or only progress for a defined period of time.^1153^

Gene drives that are based on targeted nucleases require that the gene is copied to the new site via HDR. The nuclease, usually Cas9, creates the double strand break at the target site. If HDR is available, the cell repairs the break using the gene drive cassette as a template. HDR is usually prevalent in germline cells during meiosis. HDR repairs the break using the gene drive DNA cassette as the template, leading to its copying onto both chromosomes and super-Medelian inheritance. However, if the DNA repair occurs by non-homologous end joining (NHEJ), then mutations are introduced (insertions or deletions) that can make the allele resistant to cleavage, preventing the further spread of the gene drive.^1189^

##### 2.3.3.1 Insect Gene Drives

The majority of animal gene drives are “homing-based drives” that have been shown to function in insects, including those of agricultural importance (Figure 16C).^1190^ The gene drive DNA consists of the gene-of-interest that confers the function that should be driven through the population. It also contains the gene for Cas9 and the gRNA to target it to the allele on the other chromosome. Instead of normal repair, the cell uses HDR to insert the gene drive into the new chromosome, including the Cas9 and gRNA genes. While this basic structure is usually adopted, there is variability in the gene (and function) that is targeted and spatial/temporal control over the expression of Cas9 (*e.g.*, limiting it to a sex or tissue type). The gene drive can be quite large, transferring >15kb of DNA.

The first insect homing-based drive using Cas9 was demonstrated in *Drosophila melanogaster*.^1190^ Subsequently, gene drives have been developed for agriculturally-relevant insects. Spotted Wing Drosophila (*Drosophila suzukii*) is an invasive pest that consumes cherries, berries, and grapes. A gene drive has been developed that targets a gene crucial for female development, thus rendering them sterile in a population (Figure 16D).^1157,1191^ Gene drives have also been developed for diamondback moths.^1192^ A homing-based gene drive was developed for the Mediterranean fruit fly that converts females to males, rather than killing them.^1193^

##### 2.3.3.2 Rodent Gene Drives

Mammalian invasive pests primarily comprise house mice (*Mus musculus*) and various species of rats. They lead to 5-15% annual losses in agricultural yield; in South Asia alone, the losses could feed 180 million people a year.^1194^ Means of managing rodents include pelleted poisons, which can have ecological effects by impacting unintended species that either consume the pellets or the poisoned animals. They can also be toxic to humans.

Gene drives have begun to be explored for rodents, but it is much more challenging in mammals than insects. The difficulty lies in the reliance of gene drives on HDR, whereas in mammals, NHEJ is dominant. Female embryonic stem cells and oocytes have higher HDR activity due to differences in cell cycle regulation compared to male-derived cells. Male spermatogonia continuously undergo mitosis to make new spermocytes; thus, NHEJ is the dominant DNA repair mechanism. Indeed, it was found that the components of a gene drive in *Mus musculus* only functioned properly when Cas9 expression was restricted to the female germline.^1195^ This design used a similar strategy as for insects except that the sgRNA targeting the gene to be copied was transcribed in one mouse, and Cas9 expressed constitutively (testing different tissue types and developmental timing), which were then crossed. This design led to super-mendelian inheritance with measured frequencies of up to 70%.

##### 2.3.3.3 Weed Control by Gene Drives

Invasive weeds could be controlled by gene drives.^1196,1197^ They could be used to drive a population to extinction. Alternatively, they could be applied to spread a gene that makes weeds susceptible to an herbicide or removes an herbicide resistance gene. There are limitations on using gene drives in plants. The NHEJ pathway is the preferred mode of DNA repair, requiring similar solutions to gene drives in mammals, and resistance is more likely to occur.^1196^ In addition, the gene drives to date work through the spread of pollen between members, but many weeds are self-pollinating.^1184^ Farmers would also have to add seeds at the beginning of a treatment, increasing the weed burden initially, and it could take decades to have an effect.^1152^ There are also significant regulatory and societal challenges in the release of self-propagating engineered functions into the wild, particularly if they have the potential of driving a species to extinction (*e.g.*, a weed in one context may be a food source in another).^1158^

Plant populations cannot be easily controlled by homing-based gene drives because they preferentially use repair pathways that lead to resistance.^1198^ Toxin-antidote gene drives are used instead (Figure 16E). These drives consist of a toxin that kills the cell. Cas9 can serve as a toxin by designing gRNAs to target an essential gene. Multiple gRNAs were used to increase the knockdown of the gene and reduce the probability of resistance arising through mutation. The drive also had an antidote that negates the effect of the toxin. When Cas9 is used, the antidote is just a second copy of the essential gene that is mutated at the sgRNA binding sites. The gene drive was placed under the control of a promoter that only turns on in the sexual organs in order to avoid unnecessary fitness burden.

Unlike homing-based drives, toxin-antidote based drives follow Mendelian inheritance where 50% of the gametes inherit the gene drive. However, while the toxin gene is not present in the offspring, they still suffer the consequences of the toxin. The Cas9 targeting the essential gene is still present as mRNA or protein in the ovule before fertilization. This causes the seeds without the gene drive to fail to germinate. Further, by having the drive target a sex-specific essential gene, either male or female seeds can be killed thereby driving the population to one sex, ultimately leading to extinction.

Two toxin-antidote gene drives have been developed for plants and demonstrated using *Arabidopsis thaliana*, which is a weed, albeit not an agricultural pest. They have similar basic structures. In one, *NPG1* is targeted by Cas9.^1198^ This gene is required for pollen (male) development, but is not expressed in ovules (female). Therefore, only when pollen is carrying the gene drive does it develop properly, causing all the fertilized seeds to carry it. In contrast, the second toxin-antidote gene drive uses a killing mechanism that targets early embryo development rather than pollen germination (Figure 16F).^1158^ Cas9 was designed to target *YKT61*, which is recessive lethal due to its involvement in vesicle membrane fusion. Its effects occur after fertilization.

Both approaches generate seeds where up to 99% contain the gene drive and were shown to be stable for generations. The first approach targeting pollen is theoretically better at driving a gene through the population, whereas the second approach is more powerful for controlling invasive weeds. Modeling indicates that the gene drive can spread through a population in as little as 10-30 generations, requiring 10-20 years to wipe out a local population.^1152^

### 2.4 Programming Plants to Respond to Their Environment

Plants continually survey their environment and turn genes on and off to respond to shifting conditions or as a defense against threats.^1199^ Growth and development also require changing the pattern of gene expression at different times for cells to differentiate, spurring the plant to sprout, grow new leaves, control root morphology, and flower.^1200^ Chemical communication signals are transmitted between organelles within plant cells, between cells and tissues, and exogenously with colonizing bacteria and mycorrhizal fungi as well as between entire plants.^11,1201^ This language is coordinated by a large regulatory network involving thousands of biochemical interactions that sense changes, process this information, and calculate the correct pattern of gene expression.

The regulatory networks of plants respond to changes over wide time scales. Within minutes, it reacts to microbial infections or tissue damage done by insects or animals.^1202,1203^ Within hours, it responds to shifts in soil conditions, such as nutrient availability, salinity, water content, and microbial signals, to shift carbon storage or remodel root morphology.^1204,1205^ Changes in temperature, humidity, and length-of-day lead to seasonal changes in gene expression.^1199^

This dynamic control of gene expression is in stark contrast to the synthetic regulatory control typically implemented in plant genetic engineering projects. Most often, recombinant genes are designed to be always on (constitutively expressed) or use natural tissue-specific or stress-responsive promoters (Tables 1 and 5).^1206,1207^ In many cases, it would be better to produce some traits only when they are needed, for example, turning on a cold resistance trait when the temperature is low.^1208^ This can be done by building synthetic sensors, encoded in the genome, that respond to environmental stimuli by turning a promoter on or off. The output of a sensor can be used to control a gene, or it can be connected to a synthetic genetic circuit to integrate signals or implement dynamic gene expression.^1208–1211^ The circuit output can be connected to the control of recombinant or native genes. Many such sensors and circuits have been developed in bacteria, yeast, and mammalian cells, but have been slower to be adopted in plants.^887,1212,1213^ One can imagine a future where plants are programmed to continuously survey their environment, process the information to determine which traits need to be activated, and implement the dynamics of a response.

#### 2.4.1 Mapping and Manipulating Natural Regulatory Networks

Natural plant regulatory networks are large and complex and are only beginning to be understood as a system.^1214^ For example, in *Arabidopsis* there are >1,700 TFs that collectively have 1.6 million interactions with genes, 14 enhancers that impact chromosome accessibility, 20 MAPKs, 100 miRNAs, and 1,000 peptides that target 600 receptors.^1215–1220^ These regulators have an enormous impact on native gene expression; for example, the central glucose regulator KIN11 affects over 1,000 genes.^1199,1205,1221^ Chromatin immunoprecipitation sequencing (CHIP-seq) was used to identify 2 million binding sites for 104 maize leaf TFs, showing an incredible median of 16,000 binding sites per TF.^1222^ DNA affinity purification sequencing (DAP-seq) also provides information regarding how TF binding to DNA is impacted by epigenetic modifications (*e.g.*, methylation), which affected 76% of TFs in *Arabidopsis*.^1223,1224^ Yeast one-hybrid screens can screen many proteins by fusing them to an activator peptide (VP16) (Section 2.3.3.1) and then building a “bait” promoter upstream of a reporter gene. They have been used to identify the TFs that bind to a promoter to map plant regulatory networks involved with cell wall differentiation, defense response, and cell reprograming.^1224–1227^

Computational approaches accelerate the mapping of natural regulatory networks. Bioinformatic methods have identified over 400 sequence motifs conserved in genes that are up- or down-regulated by pathogens in parsley and *Nicotiana benthamiana*.^383^ Using 63 plant species, regulatory networks have been mapped between 20,000 TFs and 20 million TF binding sites.^1228^ These networks can be further refined by inferring new regulatory interactions based on co-expression data.^1229^

#### 2.4.2 Perturbing Native TF Expression

A map of the regulatory network is a useful guide to design genetic changes to improve a trait-of-interest. Within native regulatory networks, there are motifs that perform signal processing, including logic operations, feedback loops, filtering, and memory.^1204,1230^ While these motifs encode functions that are similar to electronic circuits, their high interconnectedness with native regulation and non-modularity makes it difficult to harness them in engineering projects. Due to this complexity, currently most successful projects involve only simple up- or down- regulation of a small number of TFs. While experiments that worked are described in this section, targeted manipulations to even well-characterized pathways often lead to unexpected results.^1231^

For metabolic engineering applications, natural TFs can be overexpressed to turn on silent biosynthetic pathways or to increase product titers. For example, tomatoes were made purple by expressing natural TFs (Del and Ros1) from the fruit-specific *E8* promoter leading to high levels of anthocyanins.^1232^ Taste and aroma are the result of secondary metabolites that can be altered by changing the expression of native TFs. As a means to control apricot flavor, RNA-seq data from ripening was used to find sixteen TFs.^1233^

The control of native TFs can also change plant morphology. Overexpressing TFs has led to roots that grow deeper and produce more hairs, thereby improving nutrient uptake and drought resistance in rice, plumb, and *Arabidopsis*.^813,1234^ The reproductive stages of pears were controlled through two natural TFs (via targeted RNA silencing) to create an early flowering line for breeding.^1235^ In rice, controlling the expression of a TF (IPA1) allowed the balancing of yield and disease resistance.^1236,1237^

Plant TFs can be mutated or modified to achieve a desired effect. The yield of rice increased from the mutation of a repressor to be phytohormone-insensitive to divert resources from biomass to grain.^1238,1239^ Tomato shelf life was improved by removing the activator domain from the *NOR* gene, while maintaining its ability to bind to DNA, so that it could prevent other TFs from activating ripening genes.^1240^ Rice with reduced lignin content and improved digestibility was achieved by fusing repressor domains to the OsSWN2S TF involved in promoting cell wall thickening and xylose generation.^1241^

#### 2.4.3 Synthetic Regulatory Proteins

One can use synthetic TFs when gene expression needs to be controlled without interference from the complexity of native regulatory networks. They have two essential components: a sequence-specific DNA-binding domain and a functional domain to manipulate transcription.

##### 2.4.3.1 Protein Tags to Build Regulators

A DNA-binding domain can be turned into a repressor to block the transcription of a gene. One way to achieve repression is to fuse the protein to the C-terminal SRDX tag, which is only 12 amino acids and functional in plants.^1242–1244^ It has been added to ZFPs to improve salt tolerance in ryegrass,^1245^ TALEs in *Arabidopsis*,^1244^ and dCas9 in *Nicotiana benthamiana* leaves.^1246^ Additional repression domains have been identified that work in plants, including EAR and OFPx.^1247–1249^

DNA binding domains can also be turned into activators that recruit transcriptional machinery to turn on gene expression. A commonly-used activating domain is from the Herpes Simplex Virus VP16 protein that turns on transcription by interacting with chromatin remodelers and recruiting transcriptional machinery.^1244,1250,1251^ The C-terminal VP16 domain consisting of 11 amino acids has been fused to ZFPs, TALEs, and dCas9 to create synthetic transcriptional activators in a wide variety of plant species.^1252–1255^ There are several ways to increase expression. Fusing four tandem repeats of VP16 (VP64) improves activation.^1256^ Mutant VP16 sequences increase expression in tobacco.^1257^ Other activating domains originating from plant TFs have been identified based on sequence similarity with VP16.^1257^

There are additional protein tags that can modulate the activity of TFs. The transfer of TFs from other kingdoms requires that they be fused to a nuclear localization signal (NLS) to enter the nucleus after translation. The most common NLS is simian virus 40 (SV40) motif which works across a wide range of plant species.^1258^ To make TF expression short lived, a destabilizing domain can also be added, such residues 21-30 of the KIP-RELATED PROTEIN1 (KRP1) that has been shown to increase synthetic TF degradation in *Arabidopsis*.^1259^ This approach is analogous to the often-used bacterial “LVA” tags that can be added to regulators in bacteria to increase their degradation rates.^1260–1264^ These tags have been critical in the design of rapidly responding and dynamic genetic circuits in microbes.

Chromatin remodeling is a means for a regulator to control the access of transcriptional machinery to a gene. Synthetic regulators of chromatin remodeling have been developed in other eukaryotes but have not yet used in plants. In yeast, the recruitment of the chromatin remodeling repression domain Mxi1 by fusing it to dCas9 resulted in strong repression, targeted to a specific genomic locus through gRNA design.^1265^

##### 2.4.3.2 DNA-Binding Regulators for Plants

Regulatory proteins from outside the plant kingdom have been shown to function in plants. In tobacco, TetR:VP16 drives expression from a synthetic promoter containing *tet* operators and the TATA box.^1266^ Yeast TFs from different families (MADS, Homeobox, GATA, and bZIP, Gal4) also function as activators or repressors in *Arabidopsis* and tobacco, depending on the tag fused to them.^365,1267^

A library of prokaryotic TFs was transferred to tobacco and *Arabidopsis* to serve as transcriptional activators and repressors.^314^ Synthetic inducible promoters were built by fusing up to six repeats of the TF operator motif to the CaMV35S promoter. A VP16 or *Arabidopsis* ETHYLENE RESPONSE FACTOR 2 (ERF2AD) activating domain or SV40 nuclear localization signal was fused to the C-terminus of the TF. Decreasing the number of operator sites tuned down the ON state of the gate. To create a repressible promoter, a single copy of the operator was placed immediately downstream of the CaMV35S promoter. While expressing the TF fused to a NLS was sufficient for repression, the OFF state of some TFs could be further decreased by adding endogenous repressor domains. In *Agrobacterium* transient assays, the synthetic activator- and repressor-promoter pairs displayed up to 45-fold and 13-fold induction, respectively.

Genetic circuits are artificial regulatory networks that perform computational operations. They usually are built using multiple regulators. These regulators must be orthogonal to be used together in a circuit; in other words, not interfere with each other or to each other’s DNA motif. Orthogonal regulators based on dCas9 are simple to create by designing sets of unique gRNAs, an approach that works in plants.^1246,1255,1265,1268^ However, because the gRNAs share dCas9, this resource can be drawn down and cause the fold-repression to decrease (referred to as “retroactivity”).^389,390^ Alternatively, TALEs and ZFPs can also be designed to be orthogonal and not target sequences found in the plant genome.^1269–1272^ They have modular sequences designed to allow their DNA operator to be programmable. In practice new sequences do not always work and they can exhibit nonspecificity, so orthogonal sets must be screened.

Synthetic regulators can impact gene expression by controlling chromatin access. MARs are anchor points for the genome to the nuclear matrix (Section 2.2.1.3.1).^23,331,342,798,1273–1275^ Flanking a gene with MARs has been shown to upregulate its expression in *Arabidopsis*, potato, and rice.^342,798,1276^ In yeast, the control of chromatin access at specific loci has been achieved by fusing chromatin regulator (CR) proteins to ZFPs designed to flank the recombinant gene.^1277^ TFs can also be recruited to genome regions neighboring the recombinant gene to control it through DNA looping or bending.^332,1278^ While these approaches have not been shown to work in plants, they or analogous strategies with homologous proteins are likely to be able to implement similar expression control.

#### 2.4.4 Genetically-Encoded Sensors in Plants

A sensor responds to an environmental signal or cell state and controls a function (output). The output could be a reporter, program of gene expression, or cellular response. This section focuses on transcriptional sensors, where the output is a promoter. These sensors are simple to connect to recombinant genes directly or native genes through genomic insertions or the expression of a natural or synthetic TF.^1279^

Sensors can be based on natural regulators in the cell and implemented using native promoters known to be turned on under the desired conditions. They can also be entirely synthetic, where *de novo* regulators are computationally designed to bind to a desired small molecule. The number of sensors available for plant engineering has been growing, with examples that respond to environmental conditions (drought, high temperature, etc.), agrochemical inducers, phytohormones, cell-cell communication signals, and synthetic small molecules (agrochemicals, fentanyl, TNT, etc.).^1280,1281^ Most of the work performed to date involves moving a single sensor into one plant line as a proof-of-principle, but one can imagine a future where plants contain an array of synthetic sensors that provide a myriad of situational awareness.

##### 2.4.4.1 Condition-Responsive Plant Promoters and Transcription Factors

The simplest sensors are based on a native promoter that turns on or off under the desired conditions. This section focuses on the creation of promoters that respond to synthetic signals. Table 1 (Section 2.2.1.1.1) and Table 5 (Section 4.1) provide promoters, both natural and synthetic, that respond to tissue types and stresses, respectively. Natural promoters that respond to a signal can be used to create a sensor. For example, methanol regulates some biosynthetic genes in rice and this was used to develop a methanol-inducible system based on the natural *TDC1* promoter.^1282,1283^ This promoter was used to control tyrosine production by modulating the expression of tyrosine decarboxylase in transgenic rice.^1282^ Finally, a promoter that serves as a sensor can be built by incorporating CREs that respond to plant TFs into a minimal synthetic core promoter (Section 2.2.1.1.3).

The mechanism by which the promoter responds to the signal does not have to be well understood. Methods to scan the genome for the induction of gene expression (microarrays, RNA-seq, etc.) have been used to find promoters that turn on in response to a particular stimulus. This strategy has identified promoters that respond to nutrient deficiencies (phosphorus, sulfur, magnesium, etc.), heavy metal contamination (copper, cadmium, nickel, etc.), and stress (heat, cold, drought, etc.).^1284–1289^ Isolating transcripts upregulated in leaf tissue after harvest in alfalfa led to the identification of a post-harvest inducible and ABA-responsive promoter.^1290^

The problem with using a natural promoter is that it is subject to other input signals, some of which may be unknown.^1286^ Synthetic promoters avoid this problem by placing well-defined binding sites for native TFs into a scaffold promoter. The identification of the TF binding site and the placement of its corresponding CRE placement into a promoter are aided by computational tools and databases of plant regulators.^245,1075,1291–1296^ Promoter design is described in more detail in Section 2.2.1.1.3. This approach has been used to build promoters that respond to pathogens, stress, cell type, and developmental stages.^327,356^

Promoters can also be designed to bind synthetic TFs (ZFPs, TALEs, dCas9) that can be engineered to respond to signals. This simplifies the construction of a sensor because the operator for the DNA-binding domain is well-defined, making it easier to place into a promoter scaffold. This method was used to build phytohormone sensors that respond to jasmonic acid, gibberellic acid, and auxin (so-called HACRs “hormone activated dCas9-based repressors”) (Figure 17A).^1297^ Plant sensory domains were used that, upon binding their ligand, recruit proteolytic machinery to degrade the TF. By fusing this domain to dCas9, the ligand causes the fusion protein to degrade, leading to de-repression of a promoter.

**Figure 17:**
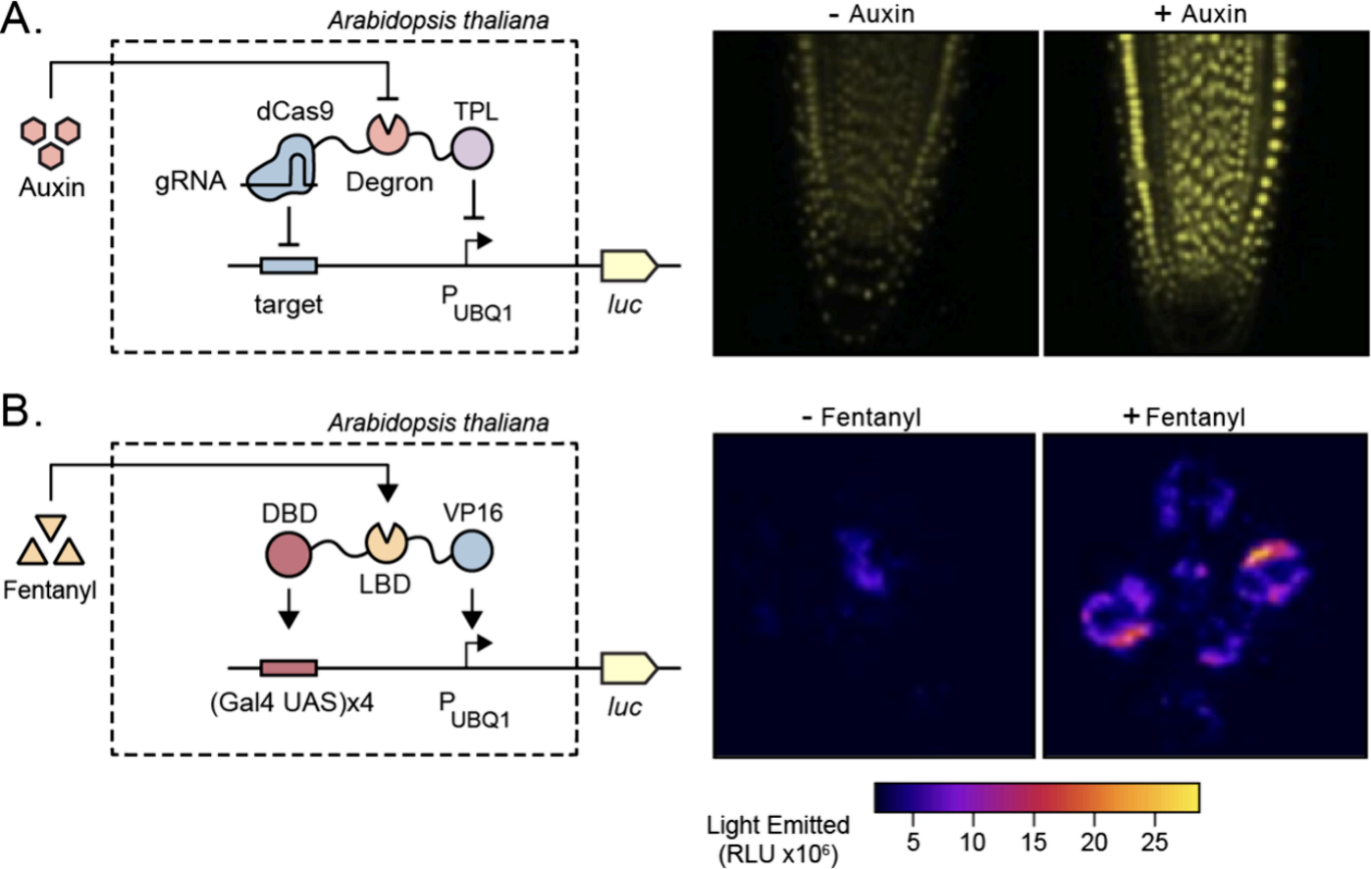
**Synthetic plant sensors**. **A**. An auxin-responsive sensor in *Arabidopsis*.^1297^ dCas9 represses the promoter through a fused TOPLESS repressor (TPL). When auxin binds the degron, the fusion protein degrades and the promoter is activated, leading to luciferase expression. *Arabidopsis* root cells displayed increased luciferase activity (yellow) upon the addition of 5 μM auxin. Image of root tips reproduced with permission from ref 1297. Available under a CC BY license. Copyright 2018, Elife. **B**. A fentanyl sensor based on computationally-designed ligand binding domains (LBDs).^1327^ The addition of fentanyl leads to an increase in protein stability. The DNA-binding domain (DBD) directs the fusion protein to Gal4 operators where VP16 activates the production of *luc*. The images show the induction of luciferase in *Arabidopsis*. Images reproduced with permission from ref 1327. Available under a CC BY license. Copyright 2017, Elife.

##### 2.4.4.2 Moving Sensors from Other Species to Plants

Regulators from other kingdoms, such as fungi or bacteria, are more likely to not interfere with native plant regulatory networks. They also offer many more options for signals that one may want to sense in a plant. The simplest mechanism is a single regulator that binds to its ligand and changes conformation to bind or unbind a DNA operator in a promoter.^1298^ They can be moved to plants by fusing them to a NLS and placing their operators in a plant minimal promoter. The binding event can activate or repress a promoter by fusing the regulator to VP16 or SRDX domains, respectively.^1244^ This approach led to antibiotic (anhydrotetracycline) and ethanol sensors using bacterial TetR-family repressors.^1266,1299,1300^ Many bacterial TetR homologues that respond to a myriad of ligands have been moved to yeast and mammalian cells and are likely to also work in plants.^1301–1305^ The so-called “XVE system” uses the DNA-binding domain of bacterial LexA, fused to VP16 and the regulatory region of the human estrogen receptor.^1306^ The resulting estradiol-activated regulator works in *Arabidopsis*, tobacco, and rice. Ligand-binding domains from other eukaryotes have been used to build plant sensors, including quinic acid, glucocorticoid, and estrogen sensors, sourced from mold, rats, and humans, respectively.^1307–1309^ The pC-HSL-responsive quorum sensor RpaR from *Rhodopseudomonas palustris* was fused to a NLS and VP16 activating domain to create a microbe-to-plant communication channel.^1310^

Plants could experience hypoxia as the result of floods or otherwise waterlogged conditions. An oxygen sensor was constructed using a TF that had been divided into two parts that need to come together for activity (split TF).^1259^ One TF half was fused to a human enzyme that responds to oxygen (hypoxia-inducible factor, HIF) and the other TF half was fused to the Gal4 binding domain from yeast fused to human pVHL (von Hippel-Lindau tumor suppressor). The PHD3 (hyman prolyl-hydroxylase) enzyme was co-expressed and hyroxylates the HIF domain, causing it to bind to pVHL and together they form a transcriptional activator.

Domains can also be attached to a protein to make them active under specific conditions. For example, proteins fused to a temperature sensitive dihydrofolate reductase (DHFR) mutant from yeast become unstable and are degraded at high temperatures.^1311^ To use it in plants, two mutations were made to shift the temperature range to that which is more consistent with plant growth.^1312^ In *Arabidopsis*, it was used to modulate native TFs involved in trichrome development and flowering time. Certain degradation domains can also be protected by or induced by the presence of a small molecule, but these systems have not been demonstrated in plants.^1313,1314^

Chemicals used in agriculture could be used to induce gene expression in the field. A sensor for the commercially-available insecticide methoxyfenozide was built by fusing the ligand-binding domain from spruce budworm ecdysone receptor (EcR), a yeast DNA-binding domain (Gal4 or LexA), and a VP16 activation domain.^1307–1309,1315^ However, the sensor had low sensitivity and high background expression. An improved “two-hybrid” sensor was developed using retinoid X receptor (RXR) from *Locust migratoria* that heterodimerzises with EcR upon the addition of methoxyfenozide.^1316^ The DNA-binding and activating domains are separated by expressing an EcR-Gal4 and RXR-VP16 fusion hybrid, ensuring DNA binding and transcription activation can only occur in the presence of methoxyfenozide. These sensors have been used in *Arabidopsis* and tobacco plants as well as corn and soybean protoplasts. Tebufenozide is an insecticide used to counter caterpillar pests. A sensor for it was constructed in tobacco by combining the ligand binding domain from a receptor from a caterpillar and the DNA-binding domain of the mammalian glucocorticoid receptor.^1317^

RNA can also serve as the sensing modality, implemented through the protection or cleavage of mRNA encoding the gene-of-interest. Ribozymes have an aptamer domain that binds to the ligand (aptamer) that controls its cleavage activity.^1318,1319^ Typically, the riboswitch is placed in the 3′-UTR of the mRNA and it controls transcript stability. The plant-derived TPP (thiamine pyrophosphate) riboswitch controls 3′-UTR splicing and can be moved to other genes to make them downregulated by TPP allowing for post-transcriptional regulation.^1320–1322^ Using high-throughput selections, aptamers have been identified that bind to many ligands, including small molecules and proteins.^1323,1324^ The frequently-used theophylline-responsive riboswitch has been used to control a nuclear gene in plants.^913,1320–1322,1325,1326^ RNA regulators can also be part of transcriptional sensor when their response can be converted to the activity of a promoter. One such method is to fuse it the mRNA encoding a TF whose translation leads to the regulation of a promoter.

##### 2.4.4.3 De Novo Sensor Design

Most sensors rely on an existing natural domain that responds to the signals. It is very difficult to create a protein that binds to a molecule or responds to a signal when there is no natural protein that does so already to some degree. However, as the structures of more plant receptors are being solved, this information can be used to guide mutations so that they bind to new ligands. Computational protein design or directed evolution can be used to alter the amino acids of a ligand binding pocket to make it accept a new ligand.^1328,1329^ The computational design of RNA aptamers remains out of reach, but powerful SELEX methods can be used to find sequences that bind to a target molecule.^1330^

Using the protein design software Rosetta, the binding pocket of a eukaryotic regulator was mutated to make variants that bind to the pharmaceuticals digoxin, fentanyl, or progesterone (Figure 17B).^1331–1333^ These variants function as sensors in *Arabidopsis*. The “bump-and-hole strategy” is a computational method to expand a binding pocket to bind to a larger molecule. It has been applied to design an auxin receptor to bind to other auxin-like molecules in plants.^1334^ Mutations can be made to make a protein stable only when bound to the target molecule. This approach was used to create a digoxin sensor in *Arabidopsis* by fusing a ligand-dependent stabilization domain to the Gal4 DNA-binding domain.^1332^

Signaling networks often use phosphorelays to integrate stimuli or transmit a signal to the nucleus. The simplest phosphorelay motifs in plants and bacteria are two-component systems, where a sensor histidine kinase phosphorylates a response regulator that binds to DNA.^1335^ A sensor for the explosive TNT (trinitrotoluene) was constructed in *Arabidopsis* by fusing a computationally-designed TNT-binding domain to the prokaryotic histidine kinase PhoR, which phosphorylates PhoB.^1336^ PhoB fused to VP16 activated a plant promoter and TNT concentrations as low as 10 pM could be detected. The response, however, was limited by cross reactions with endogenous histidine kinases.

##### 2.4.4.4 Tuning the Sensor Response Function

The output of a transcriptional sensor is a promoter that turns on or off in response to the signal. The change in a sensor’s output promoter activity as a function of the input is known as the “response function,” which can be represented mathematically.^887,1337^ Its shape determines how the sensor can be connected to a genetic circuit or used to control a change in phenotype. The activities of the output promoter in the OFF (basal level or “leakiness”) and ON (maximal) states are important in determining whether the promoter can be connected to a downstream circuit or used to induce gene expression across a threshold required for a phenotype. The ratio of these output levels is the dynamic range and the threshold is the level of input signal at the output half-maximum. The cooperativity determines the shape of the response, with low cooperatively leading to a graded response and high cooperativity leading to a step-like response. When engineering microbes, there are many ways to genetically change the response function of a sensor, including by computational design, the selection of genetic parts, or directed evolution (Section 3.6.1.4).^1338–1341^ In principle, these approaches are applicable to plants, but it is more challenging and longer process to do so. The inducer of a small molecule-responsive sensor may also have limited uptake rates or be cleared by plant metabolism, limiting its concentration in the plant cell. Therefore, the response characteristics of plant-based sensors tend to be less desirable than their microbial counterparts, even for well-characterized inducible systems.^1342,1343^

A common problem with sensors is that the OFF state is leaky, which can make them difficult to connect to circuits or cause growth defects when used to control toxic genes. One method to achieve a low OFF state is to insert the plant 5S rRNA mimic (P5SM) suicide exon within the output gene. The P5SM sequence contains a stop codon that prematurely terminates the translation of the output gene when included in the mature mRNA.^1344,1345^ Through a process called exon skipping, the expression of the ribosomal protein L5 induces efficient RNA splicing and excision of the P5SM exon, leading to translation of the complete mRNA.^1346^ To reduce the leakiness of the dexamethasone-inducible sensor, P5SM was placed in the mRNA of the output gene and both the output gene and L5 were placed under control of a Dex-inducible promoter.^1347^ This modification eliminated detectable leaky expression when the sensor was OFF.

#### 2.4.5 Computing in Planta with Synthetic Circuits

Cells can be programmed to perform computational operations using synthetic genetic circuits.^887^ They implement signal processing functions that can alter the output of a sensor, including noise filters, thresholding, inversion, or making the response more graded or switch-like. Integrating multiple sensors using logic operations increases the specificity of a response, ensuring that a gene only turns on in the correct combination of conditions. Circuits can also coordinate a multi-stage process during development or growth. They can implement dynamics, such as producing a pulse of activity or oscillations. Analog circuits perform complex mathematical operations, such as addition, with an efficient small number of regulators.^1348–1351^ Memory permanently records a transient signal. Collectively, these circuits enable the programming of “smart plants” to continuously surveil the environment, respond to or remember threats, and change gene expression when a farmer applies an inducing agrochemical in the field.

The bulk of genetic circuit design has been performed with prokaryotes and the eukaryotic work has focused on yeast and mammalian cells.^1279,1304,1352–1354^ There are several challenges when building circuits in plants. First, more complex circuits require precision in balancing the expression of multiple regulators. This has been difficult because of a lack of part libraries encompassing wide ranges of expression and the historical reliance on techniques that insert genes in random locations in the genome. Second, the slow growth of plants makes it difficult to rapidly evaluate designs (Section 2.5) and makes it difficult to use directed evolution methods *in planta* (Section 2.2.2.5). In contrast, microbial circuit design has required iterations of alternative designs and trial-and-error mutations to tune the circuit response. Finally, it is difficult to measure the output of a circuit due to interference with fluorescent reporters, tissue-specific expression, and the inability to use single cell methods (*e.g.*, flow cytometry). Despite these challenges, circuits are slowly being developed for plants. This capability would allow plants to be programmed to sense and respond to environmental stresses in the field and communicate with microbes in the soil. Further, genes could be turned on in specific tissues or stages of development to have a desired effect.^1355^

##### 2.4.5.1 Tuning Sensor Response Functions

Circuits can change the response of a sensor output promoter. They can amplify a signal, filter noise, create a biphasic response, or change the input threshold at which the sensor turns on. An amplifier has been built for plants. In *Arabidopsis*, a positive feedback loop was constructed by placing TALE-VP64 expression under the control of a promoter it induces.^1253^ This amplifier was connected to the anthocyanin biosynthesis regulator *PAP1*, which led to a 150-fold increase in *PAP1* mRNA.

Another signal amplifier, the so-called “Q-system,” has a minimal promoter that is controlled by the expression of a transcriptional activator and a repressor that is inhibited by quinic acid.^1307^ This system has been validated in soybean protoplasts and *Nicotiana benthamiana* leaves. Expressing the transcriptional activator can amplify a promoter output by 8-fold. Further, the level of amplification can be tuned by mutating or removing domains in the activator protein.

##### 2.4.5.2 Logic Operations

Logic is at the core of every computer. A logic circuit takes one or more inputs and produces an output following a defined operation. The inputs and outputs of digital circuits can be either a 1 (on) or 0 (off). An AND gate is an example; the output is only on when both inputs are on and is otherwise off. NAND and NOR gates are special because they can be used to build any computational operation. Thus, they are referred to as “universal” or “Boolean complete.”

Logic is common in natural regulatory networks. It is used to integrate signals to identify a cell type, improve the specificity of a response, or control multiple traits in response to different combinations of signals. Note that biological responses, including synthetic genetic circuits, have on/off states that are “fuzzy,” meaning they have intermediate values between 1 and 0 (*e.g.*, 0.9 and 0.2).

Natural promoters encode logic operations by combining CREs upstream of the core promoter (Section 2.2.1.1). Synthetic promoters can be designed that implement logic through the placement of CREs.^147,365,1356^ For example, if the binding of either of two TFs turns on the promoter, this acts as an OR gate. Different logic operations can be realized by changing the number and organization of the operator sites (CREs) in the promoter (Section 2.2.1.1.3). All 2-input logic operations were demonstrated in *Arabidopsis* using bacterial DNA binding proteins and the placement of their bacterial DNA operators as CRE (Figure 18).^314^ This work was demonstrated by transforming different combinations of repressors and measuring the response in plants (each plant is a different combination of inputs). Similarly, a 3-input 1-output logic gate was built by designing a promoter to respond to three synthetic TFs (two activators and one repressor).^365^

**Figure 18:**
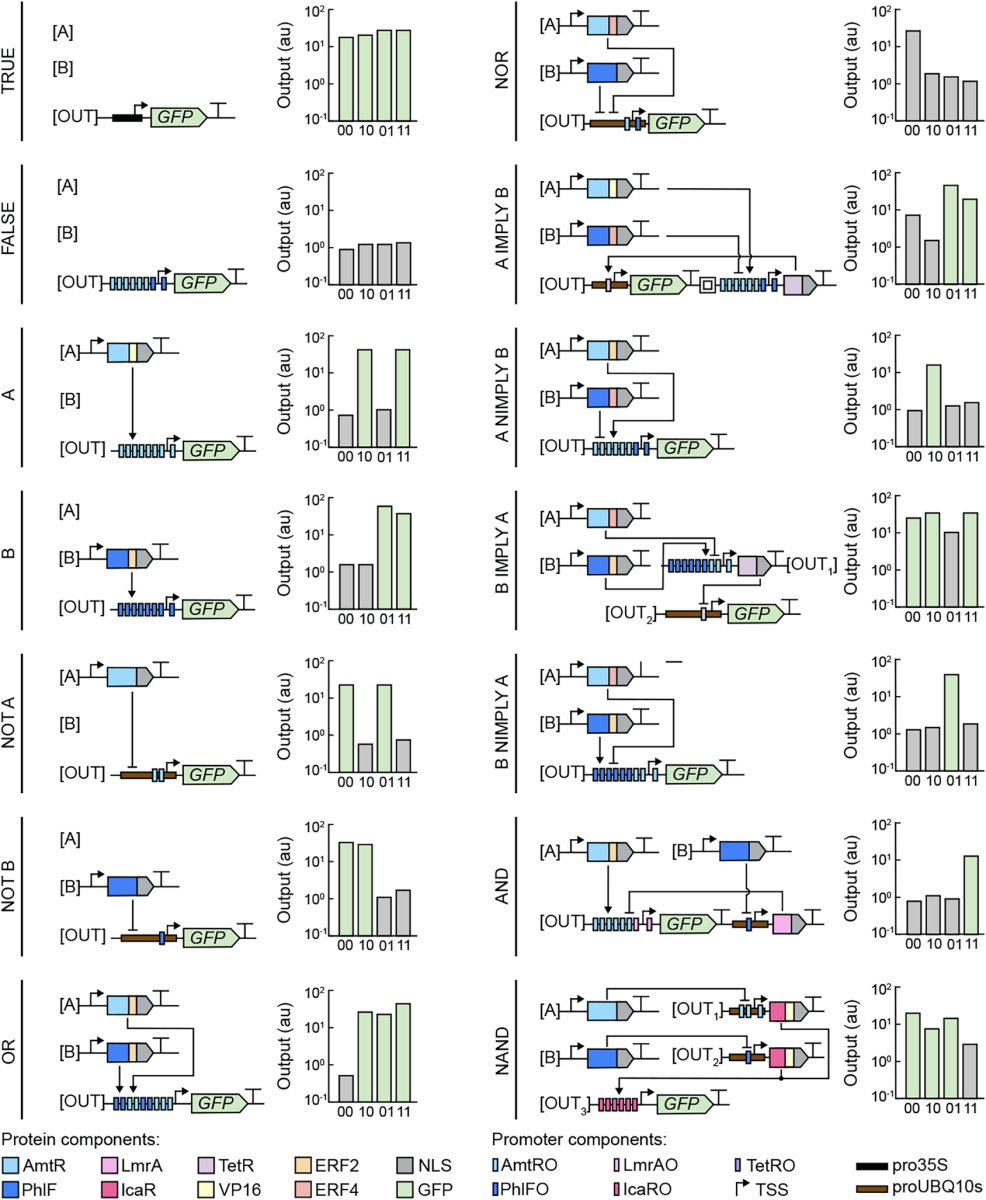
**Synthetic genetic logic in plants**. **A**. Logic gates measured through the transient transfection of *Nicotiana benthamiana* leaves.^314^ The expected output of each gate given inputs [A] and [B] is indicated by the color of the bar; green bars should be ON and gray bars should be OFF. The inputs of the circuit [A] and [B] represent transcriptional activators or repressors encoded on separate *Agrobacterium* plasmids co-infiltrated into tobacco leaves. The TRUTH values ‘00’, ‘01’, ‘10’, and ‘11’ indicate whether either or both inputs were delivered; ‘1’ means the input was present and ‘0’ means it was absent (in order: AB). Likewise, the circuit output [OUT] was encoded on an *Agrobacterium* plasmid, co-infiltrated into the tobacco leaf with the inputs. Circuit output was measured as a ratio of the *gfp* output to a constitutively-expressed *rfp* marker on the output plasmid.

Logic can be used in a single plant to dynamically respond to changes in conditions. Doing so requires the connection of the logic operation to upstream sensors (Section 2.4.4). The sensor output is a promoter, which can be used to drive the expression of a TF that then serves as the input to the promoter implementing the logic operation. For example, a DEX-inducible sensor was connected to a NOT gate, which was shown to invert the response (Figure 19A).^317^ The response was measured in *Arabidopsis* and sorghum protoplasts and the data used to parameterize a mathematical model to aid the connection of the sensor to the circuit. A larger set of NOT gates for plants was constructed using a set of six orthogonal bacterial repressors (Figure 19B).^731,1340^ These were characterized by connecting an auxin sensor (NAA, 1-naphthaleneacetic acid) output to drive the expression of a repressor. The data were obtained using protoplasts (Section 2.2.1.1.6) and used to parameterize a mathematical model that could predict how to connect (“layer”) the repressors to make all possible 2-input logic gates (Figure 19B). The model used promoter activities measured using a reference standard to convert to RPU (Section 2.2.1.1.7). Finally, it was shown that the data could be used to build a NAND gate in *Arabidopsis* plants that could dynamically integrate the NAA sensor with a cytokinin sensor (tZ) (Figure 19B). These experiments are the first step toward the development of genetic circuit design automation software, where the user specifies the desired circuit and the DNA sequence is generated (Section 3.6.1.4).^158,729,1357^ This tool is available for other eukarotyes, including yeast and mammalian cells.

**Figure 19:**
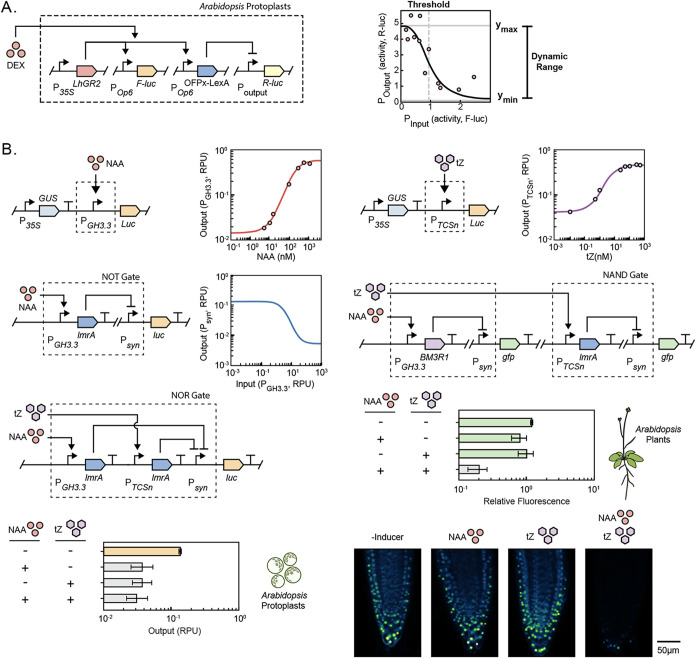
**Connecting sensors to logic gates**. **A**. Connection of a dexamethasone (DEX) sensor to a NOT gate.^317^ The sensor was used to drive the expression of the synthetic repressor (OFPx-LexA) and firefly luciferase (F-luc), which allowed for quantification repressor expression. The output of the synthetic promoter was quantified with *Renilla luciferase* (R-luc). The line shows the fit to the NOT gate response function: *y* = *y*_*min*_ + (*y*_*max*_ – *y*_*min*_)/[1 + (*x*/*K*)^n^], where *y* is the output promoter activity (with range *y*_*min*_ to *y*_*max*_), *x* is the sensor output promoter activity, *K* is the half-activation, and *n* is the cooperativity. The threshold, or the switchpoint of the sensor, is shown as a dashed line. The 4-hydroxytamoxifen (OHT) inducible promoter was also tested as an input (not shown). **B**. Building logic gates in plants. The endogenous auxin-inducible *Arabidopsis* promoter (pGH3.3) and synthetic cytokinin-responsive promoter (pTCSn) were characterized in *Arabidopsis* protoplasts. Promoter activity following addition of auxin (1-naphthaleneacetic acid, NAA) or cytokinin (trans-zeatin, tZ) was measured in relative promoter units (RPUs) by normalizing luciferase (*luc*) activity to a constitutive β-glucuronidase (*GUS*) control. NOT gates were built by placing prokaryotic repressors (*lmrA* or *BM3R1*) downstream of the inducible promoters, which leads to repression of a synthetic promoter (Psyn) upon NAA induction. Using these parts, a NOR logic gate was constructed; the activity of the gate was measured in *Arabidopsis* protoplasts using RPUs with the expected on states displayed in yellow and the off state in gray. A NAND gate was constructed controlling *gfp* expression and stably integrated into *Arabidopsis* plants. The activity of the logic gate was measured using GFP fluorescence relative to the maximum intensity measured in each line. Root tip fluorescence images reproduced with permission from ref 731. Available under a CC BY-NC-ND 4.0 license. Copyright 2025, Nature Communications.

Other regulatory interactions have been used to build layered gates. In *Arabidopsis* protoplasts, NOR gates were constructed using dCas9 to target sequences that repress an otherwise constitutive promoter.^1358^ These NOR gates were layered by having the output of an upstream gate be a gRNA that represses a promoter that is the input to a downstream gate. These circuits were used to integrate dexamethasone-inducible and heat-inducible promoters in *Arabidopsis* plants. In tobacco leaf infiltrations, an AND gate was built using TFs based on TALEs that had been split into two proteins that must both be present.^1359^ Logic gates have also been built by fusing computationally-designed heterodimerizing domains to either DNA-binding or a transcription-activation domains: when the correct pair bind, the complete TF is formed.^1360,1361^ In *Arabidopsis* plants, the system was used to express luciferase only when two hormone-responsive promoters were activated. Finally, sensors can control the expression of enzymes that produce ligands that then activate or repress TFs to impart genetic logic at a promoter.^1362^ For example, the bacterial TtgR is derepressed by flavonol-derived metabolites that are produced in plants by expressing the endogenous MYB12. By using MYB12-induced flavonol production as a circuit input, AND and NIMPLY logic operations were performed in *Nicotiana benthamiana* transient transfections.^1362^

A problem with this approach is that each layer has a delay, which can lead to transient incorrect outputs known as a “fault” in electronics.^1363^ A fault could lead to a pulse of gene expression when it is unwanted, during the transition between two circuit states that should both be off, expressing a pesticide gene even when there is no insect. Any given logic operation can be implemented with many different combinations of gates. Wiring diagrams can be selected to reduce the possibilities of faults by choosing a design with fewer layers.

##### 2.4.5.3 Memory

Memory records events. In the field, genetically-encoded memory could convert a transient into signal into a persistent change in gene expression. For example, a single treatment with an agrochemical to mature plants could be converted to a response that continues to the end of a growth season. Plants naturally have mechanisms of memory, for example exposure to a stress, but they involve epigenetic and chromosome changes that affect many native genes and are hard to control with genetic engineering.^815^

“Volatile memory” requires the constant usage of energy and resources to maintain the state. For example, bistable switches formed by positive feedback loops or cross-repression require the continuous expression of the TF(s), otherwise the memory state is lost.^1364–1366^ For a range in the input stimulus, bistable switches have two stable steady-states (on and off) and therefore exhibit hysteresis in the transition from on-to-off and off-to-on.^1367,1368^ Positive feedback loops have been built in plants (Section 2.3.4), but bistability was not experimentally shown. Cross-repression has been implemented in Arabidopsis using two repressors (the LexA and Gal4 DNA binding domains fused to either the EAR or OFPx repressor domain) that inhibit each other’s expression.^1249^ Each repressor was also expressed by a sensor (DEX or 4-hydroxytamoxifen). This circuit was shown to behave as a bistable switch in the plant.

“Permanent memory” does not require constant energy to maintain the state. In other words, the state would persist even after the plant has died. Enzymes that modify DNA act as a form of memory. For example, when an invertase flips a DNA sequence in the genome, each orientation corresponds to a different memory state.^1369,1370^ The state can be read out as a change in gene expression when the flip re-orients a promoter or terminator to turn expression on or off. Such a memory switch has been built in tobacco using a phage integrase to invert DNA containing a promoter (Figure 20A).^1371^ The integrase was placed under the control of an estradiol sensor and when plants are exposed transiently to this molecule, expression continued permanently.

**Figure 20:**
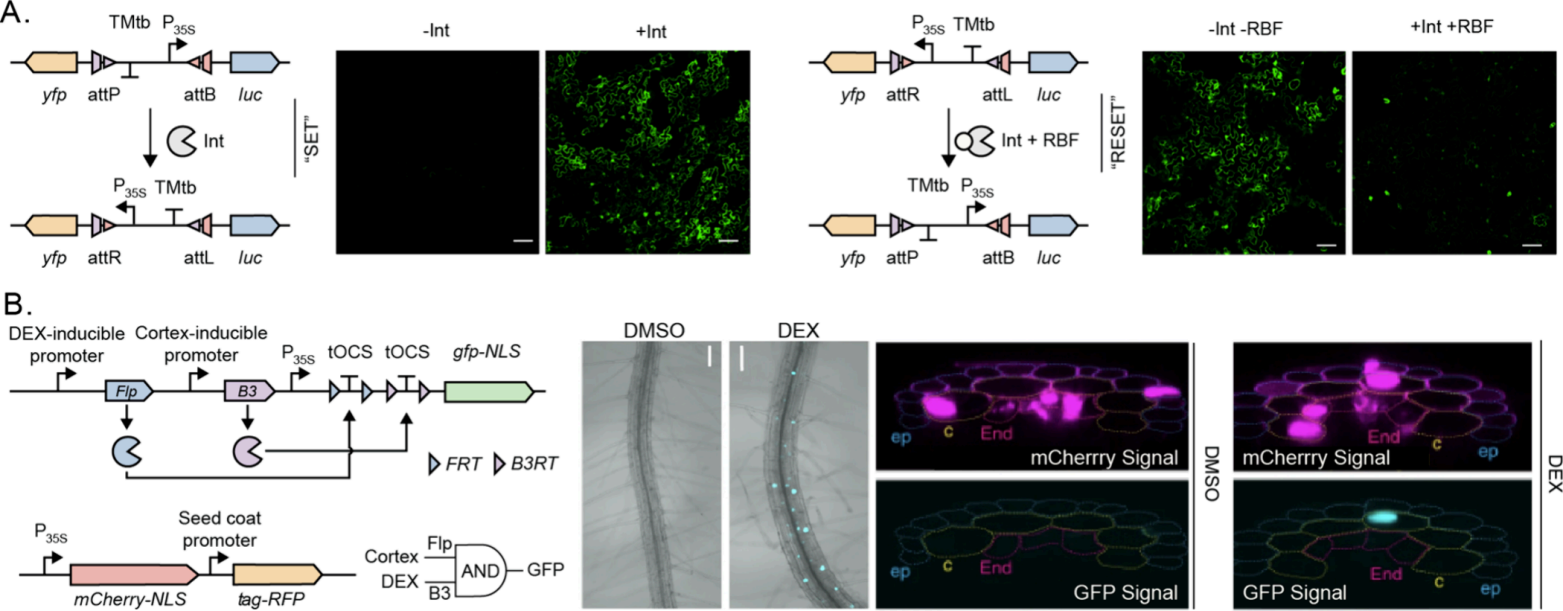
**Memory circuits in plants**. **A**. Reversible bistable circuit in tobacco.^1371^ The circuit was “SET” by the expression of a bacteriophage ϕC31 integrase (Int) turning ON the expression of a fluorescent reporter (YFP). The circuit was “RESET” when Int and recombination directionality factor (RDF) are co-expression in tobacco leaves, reversing the recombined orientation of the circuit. For microscopy experiments, T1 generation tobacco carrying the fluorescent reporter constructs was transiently infected with *Agrobacterium* carrying DNA cargo encoding the Int or Int+RBF proteins. Fluorescence images reproduced with permission from ref 1371. Available under a CC BY license. Copyright 2020, Nucleic Acids Research. **B**. Logic memory in *Arabidopsis*.^1377^ An AND gate was constructed in a stable transgenic *Arabidopsis* line that required activity from a cortex-specific promoter and a dexamethasone (DEX)-inducible promoter to express a fluorescent reporter localized to the nucleus. The construct also targeted mCherry to the nucleus and RFP to the cell membrane (Tag-RFP) as a control and to outline three root cell types: epidermis (ep; blue), cortex (c; yellow) and endodermis (end; pink). Root images reproduced with permission from ref 1377. Copyright 2022, Nature Biotechnology.

The presence of a particular combination of signals can also be recorded by memory that is implemented by logic operations where the underlying biochemical mechanism is permanent. While this implements a logic-like function, it is not true logic because it can only respond once and does not change output states.^887^ Here, we refer to this type of circuit as “memory logic.”

Memory logic can be implemented with recombinases.^1369,1372–1376^ FLP recombinase binds to two target sequences and excise the DNA between them (CRE recombinase does as well). They can impact gene expression several ways. They can remove a part, such as an upstream terminator, that turns genes on or off. Or they can permanently remove an entire gene. Two-input Boolean logic gates were made in *Arabidopsis* plants using two orthogonal recombinases (Flp and B3) and recombination sites flanking a strong terminator, promoter, or reporter gene (Figure 20B).^1377^ When a single upstream terminator (tOCS) was flanked by both Flp and B3 recombinase sites, either of the recombinases could activate expression, thus functioning as an OR gate. A different arrangement that included placing the recombinase sites outside of the promoter sequence, rather than terminator, led to a NAND gate.

Memory logic can also be implemented at the post-translational level. In tobacco, a transcriptional activator was fused to viral protein domains that suppress its function.^1378^ Cleavage sites were introduced into the fusion protein so that the suppressing domain is removed upon the expression of the protease. OR logic was implemented by placing two protease recognition sites for two separate proteases (TEV and TVMV) between the protein domains. To perform AND logic, two different suppressing domains were fused to the N and C-terminal of the activator to require two cleavage events for activation. The output was converted to pigment (anthocyanin) biosynthesis.

#### 2.4.6 Connecting Synthetic Circuits to Plant Phenotypes

While often characterized using a reporter gene, the outputs of sensors and circuits can be used to control plant phenotypes. The simplest approach is to express the gene(s) controlling the phenotype by placing them under the control of the output promoter of the circuit. Complex traits, that require the more subtle perturbation of multiple native genes on the genome, can be more difficult to control. For example, to alter progression through a developmental pathway or to up- or down-regulate enzymes to change fluxes through metabolism. To this end, there are technologies to connect a promoter activity to the control of expression of multiple genes at the transcriptional, translational, or post-translational levels.

##### 2.4.6.1 Expression of Native TFs

An output promoter can control the expression of a native TF sourced from the plant's regulatory network. In effect, this connects a synthetic circuit to natural circuits.^1379^ This connection can be implemented either by knocking out the native copy of the TF gene or connecting a second copy to the output promoter of the synthetic circuit. The latter approach allows the TF to be mutated to remove unwanted natural inputs. For example, the *gai* repressor was mutated to make it insensitive to the phytohormone gibberellin.^1238^ When the mutated Gai was expressed in rice, wheat, and maize, this induced a dwarf phenotype with increased grain yield.

There are several examples where a TF was placed under the control of a sensor to induce a phenotype at a desired time. Petunia growth can be stunted at a specified time using a dexamethasone sensor to control the expression of the mutant Gai-1 (Figure 21A).^1380^ Similarly, to time rice flowering to optimize yield or react to inclement weather, a repressor was constitutively expressed and its anti-repressor placed under the control of sensors that respond to the agrochemicals Routine and Orzyemate (Figure 21B).^1381^

**Figure 21:**
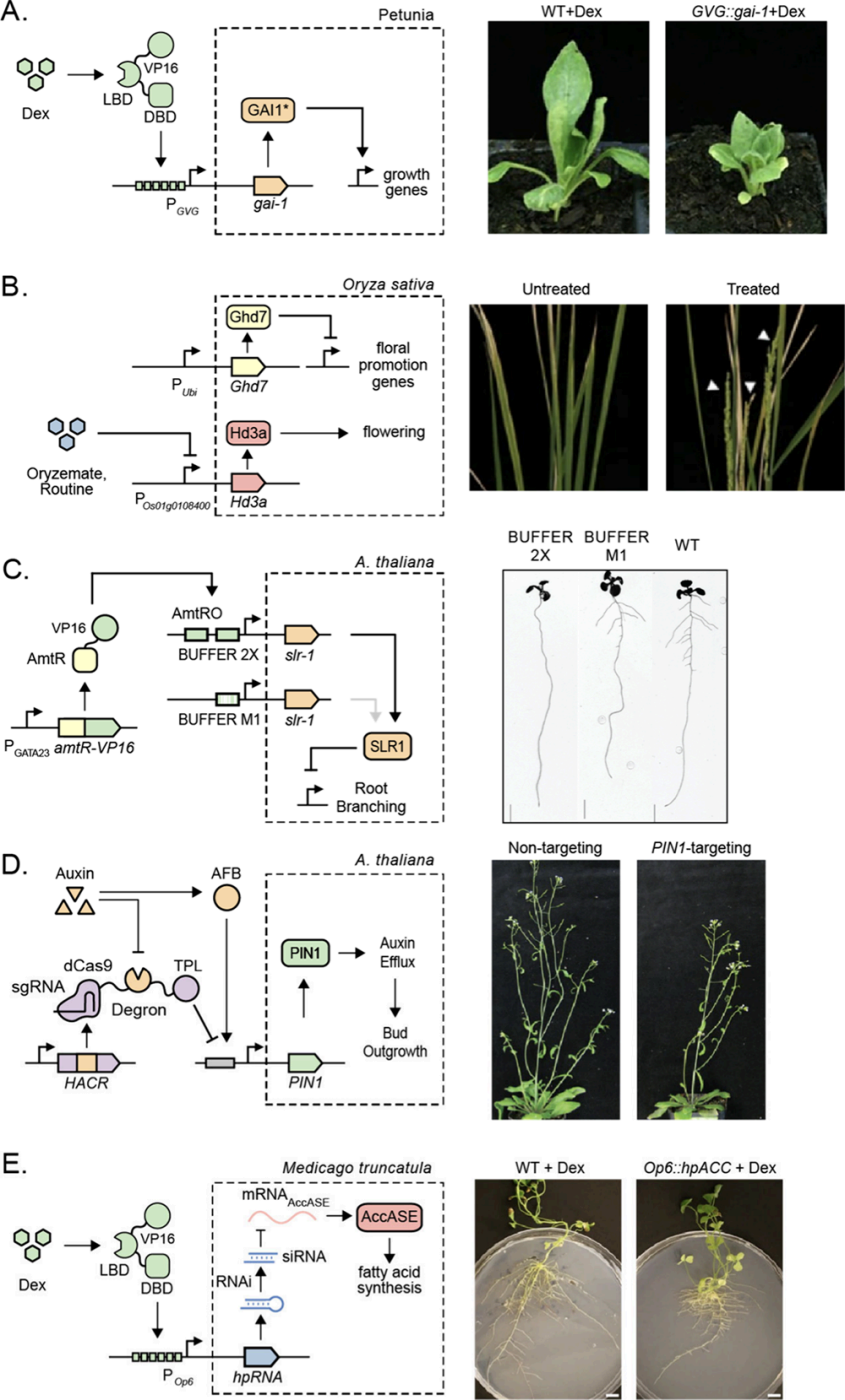
**Connecting a circuit output to control a plant phenotype**. For each case, the output of a sensor or genetic circuit controls the plant phenotype. **A**. Stunted petunias.^1380^ A dex-inducible system leads to the activation of P_GVG_ and the expression of the GAI1, which represses growth causing dwarfism. Mutant GAI1 (GAI1*) was introduced to be insensitive to other signaling pathways in the cell. Plants containing the circuit that were sprayed with 30 μM dex displayed stunted growth. LBD: ligand binding domain. DBD: DNA binding domain. Images of plants reproduced with permission from ref 1380. Available under a CC BY-NC-SA 3.0 license. Copyright 2014, Horticulture Research. **B**. Agrochemical induction of flowering of rice.^1381,1382^ A constitutive promoter P_Ubi_ was used to express Ghd7 and factors that inhibit spontaneous flowering. Routine or Oryzemate upregulates P_Os01g0108400_, which was connected to the TF Hd3a to induce flowering. Rice plants exposed to Routine (Treated) or not exposed to Routine (Untreated) are shown. White arrows indicate flowering spikelets indicating initiation of flowering. Images of plants reproduced with permission from ref 1381. Copyright 2017, Nature Plants. **C**. Control of root branching in *Arabidopsis*.^314^ P_GATA_ is a cell-type specific promoter for lateral root stem cells, which initiate root branching in *Arabidopsis*. Expression of a mutant of the root development regulator *SLR1* decreases root branching. Instead of directly connecting the promoter to this TF, a buffer gate is formed with the bacterial AmtR DNA-binding domain to tune the expression of *SLR1* and the frequency root branching. A BUFFER 2X gate contains two AmtR operator sites, leading to efficient recruitment of the AmtR transcriptional activator and high *SLR1* expression. Mutating AmtR operator sites creates a mutant (M1) BUFFER gate, which leads to weak AmtR binding and low *SLR1* expression. Representative 10-day old *Arabidopsis* plants with wild-type (WT) and engineered branching phenotypes (BUFFER 2X and BUFFER M1) are shown. Images of *Arabidopsis* reproduced with permission from ref 314. Copyright 2022, Science. **D**. Control of shoot architecture of Arabidopsis.^1297^ PIN1 expression leads to auxin efflux, which controls bud outgrowth and shoot formation. PIN1 is activated via the auxin signaling F-box (AFB) protein in the presence of auxin. To tune down the expression of PIN1 and reduce bud outgrowth, an HACR protein was used, which contains a dCas9 and TOPLESS repressor (TPL) fused by an auxin-inducible degron domain. In the absence of auxin, HACR repressor SIN1 was guided by an sgRNA. An *Arabidopsis* plant containing an SIN1-targeted HACR and a control plant lacking the SIN1 sgRNA (non-targeted) are shown. Auxin leads to the degradation of HACR, thus derepressing SIN1. HACR plants require a higher concentration of auxin than wild-type to express SIN1, leading to dampened SIN1-induced bud outgrowth. Images of plants reproduced with permission from ref 1297. Available under a CC BY license. Copyright 2018, Elife. **E**. Control of root development in *Medicago*.^1383^ The dex-inducible system drove the transcription of hpRNA that produced siRNAs targeting acetyl-CoA carboxylase (AccASE) mRNA. The strain displayed decreased root growth in media containing DEX. WT: wild-type. Images of *Medicago* roots reproduced with permission from ref 1383. Available under a CC BY license. Copyright 2016, Scientific Reports.

A delay can also be implemented with a BUFFER gate, which is single-input single-output logic where the input and output states are identical. The gate was built by having the output of the sensor or circuit control the expression of an intermediate activator, which then controls the final TF responsible for the phenotype. The delay was due to the additional time required to express the intermediate activator. A BUFFER gate can also tune the expression of an output by using the intermediate TF to amplify or suppress the input signal. In *Arabidopsis*, a BUFFER gate based on an AmtR-VP16 activator was used to control a mutant of the native TF IAA14. In lateral root cells, IAA14 is responsible for determining the degree of root branching. By modulating the intermediate activator strength by adjusting the number and strength of AmtR operator sites in a synthetic promoter upstream of the mutant IAA14, the degree of lateral root branching was controlled (Figure 21C).^314^

##### 2.4.6.2 Expression of Synthetic TFs

The output of a circuit can be connected to the transcriptional control of native genes by repurposing the DNA-targeting tools developed for genome editing (Section 2.1.1). By fusing them to activation or repression domains, TALEs, ZFPs, and dCas9 can repress native genes.^32,1384,1385^ In *Brassica*, ZFPs have been used to reduce the saturated fatty acid content of canola oil by elevating the expression of two canola β-ketoacyl-ACP synthases.^1386^ In *Arabidopsis*, dCas9 fused to various epigenetic modifiers targeting the floral regulator FLOWERING LOCUS T (FT) caused altered flowering time.^1387^

Plant morphology can also be controlled with synthetic circuits. Repressors based on dCas9 that respond to ligands can be targeted to specific regions of the genome (HACRs, Section 2.3.4.1). In Arabidopsis, a HACR responding to auxin was targeted to the promoter of an auxin transporter, thereby reducing the gain of a natural positive feedback loop.^1297^ This change reduces the strength by which auxin regulates its own abundance in the shoot, leading to a reduction in shoot branching (Figure 21D).

Simultaneously targeting multiple genes could redirect carbon flux, mediate complex phenotypes (*e.g.*, lignin biosynthesis), or coordinate stress responses. Multiple TALEs were used to simultaneously activate three *Arabidopsis* genes.^1388^ However, each gene targeted required the design of a different TALE. It is comparatively easy to direct dCas9 to multiple genes because of the simplicity of designing gRNA; in bacteria, up to 22 gRNAs have been used to regulate 13 genes simultaneously.^894^ Directing multiple gRNAs to a single promoter leads to a stronger activation or repression than relying on a single gRNA.^1246^ In rice protoplasts, this approach was used to simultaneously activate three genes.^1388^ The three genes were targeted using a multiplexed CRISPR system (CRISPR-Act2.0) that combined a gRNA scaffold with bacteriophage aptamers that recruit four VP64 activating domains to dCas9:VP64. This system activated three genes simultaneously in rice protoplasts.^1388^ Note that multiplexing a shared resource (*e.g.*, dCas9) to different genes could weaken their activation due to retroactivity (Section 2.3.1).^389,1389^

##### 2.4.6.3 Control of mRNA Degradation

Circuits can also control expression of native genes by expressing factors that target their mRNA. RNAi (Section 2.2.1.4) can be used as the output of a genetic sensor or circuit. To control root growth in *Medicago*, a dexamethasone sensor drove the expression of hpRNA that led to the suppression of a fatty acid synthesis gene that is critical for root survival (Figure 21E).^1383^ An ethanol sensor was used to control dsRNA to knockdown genes involved in chlorophyll biogenesis in tobacco plants.^1390^ A 17β-estradiol sensor was connected to a cre/lox memory switch to control RNAi that suppressed a GFP reporter in *Arabidopsis thaliana* and *Nicotiana benthamiana* plants.^1391^

Endonucleases can also be used to regulate expression at the mRNA level. The bacterial Csy4 endonuclease targets a 28 nt sequence that can be inserted into the 5′-UTR of a transcript. When this sequence is present, the induction of Cys4 degrades the mRNA. Csy4 and homologues thereof have been used to degrade mRNA in tobacco and rice.^134^ RNA-guided RNA-targeting endonucleases (*e.g.*, Cas13a) offer the ability to target any mRNA for degradation (Section 2.2.1.4). Cas13a can be directed to cleave viral RNA, thus providing engineerable viral resistance to the host plant.^1392,1393^ Alternatively, catalytically deactivated dCas13a fused the functional domains IF3 and Rbfox1 can be used to enhance translation or alter splice sites, respectively.^1394,1395^

##### 2.4.6.4 Epigenetic Modifications

A sensor or circuit can also drive epigenetic modifications to the plant chromosome. A DNA methyltransferase has been fused to different ZFPs to target various promoters for methylation, thus silencing downstream gene expression.^1396^ Upregulation of a promoter can also be induced in this way. In *Arabidopsis*, a human demethylase was fused to a ZFP to target it to a promoter to turn it on.^1397^ Epigenetic modifications can also alter access to and recognition of DNA motifs. In cassava, a methyltransferase domain fused to a ZFP targeted a promoter that *Xanthomonas* effectors bind to during infection, thus preventing gene activation and increasing bacterial resistance.^1398^ A wider range of DNA sequences can be targeted by fusing the methyltransferase to dCas9.^1399^ Multiple gRNAs can be tiled in the promoter to methylate a larger region of the DNA, thus having a larger effect.

An interesting aspect of epigenetic modifications is that they can result in a heritable phenotype (epialleles).^1396,1397,1399^ For example, a demethylase targeted to the promoter of the FLOWERING WAGENINGEN (FWA) gene in *Arabidopsis* caused up-regulation of FWA and a late-flowering phenotype.^252^ When the demethylase was segregated away in later generations, the FWA remained demethylated and the late-flowering phenotype was inherited despite the loss of the recombinant demethylase gene.

#### 2.4.7 Genetic Sensors and Circuits in Plastids

Plants have the capacity for large circuits, as evidenced by their complex native regulatory networks, but the ability to design synthetic circuits is far behind bacteria.^147,1403–1405^ Genetic circuit design is much more advanced in bacteria, where entire electronic circuits can be encoded; for example, adders, counters, or multiplexors (Section 3.4.3).^1351,1406,1407^ Even entire electronic chips, including one controlling the liquid crystal display (LCD) of a calculator, have been encoded in DNA and carried by a bacterium.^1408,1409^ Design automation software, such as Cello, aid in the design of DNA sequences encoding these circuits and simulation software can be used to predict performance in the cell (Section 3.6.1.4).^1023,1408^ Further, many more sensors have been developed for bacteria and a dozen or more can be carried by one cell.^1410^

As an alternative, the plastid may be a promising place to carry circuits because of its prokaryotic origin. Chloroplasts or differentiated non-green versions of them (Section 2.2.2.3.2) could be used as a sort of central processing unit (CPU) in the plant. One can imagine sensors in the plant nucleus communicating their output to the chloroplast, where the computation is performed. There are protein transport pathways by which tagged proteins produced in the cytoplasm are imported into a plastid. These have been used to direct T7 RNAP, proteins required for mRNA stability to the plastid.^917,918,1017^ The result of the calculation could be connected to recombinant gene expression from the chloroplast. However, to truly serve as the CPU, the chloroplast would have to be able to send the signal back to the nucleus, but no such signal has been identified or devised.

As an early step to this vision, genetic sensors built using *E. coli* parts have been moved to the plastid genome. An IPTG sensor was constructed in the tobacco plastid by placing the LacI operator into a constitutive plastid promoter.^1017^ A light sensor has been built based on cyanobacterial Orange Carotenoid Protein (OCP) domains that dimerize in blue light.^1411^ Each domain was fused to a σ and anti-σ pair that turn on a promoter when dimerized. A sensor based on a theophyllin riboswitch has been built by fusing it to mRNA encoding T7 RNAP.^913,1412^ When bound to theophylline, the RNAP is expressed leading to the 100-fold induction of a T7 RNAP promoter (Figure 22D). A heat sensor was built in tobacco plastids using a switch based on a repressor from bacteriophage λ.^1413^ A mutant of the repressor cI857 is heat sensitive and when it degrades, a promoter turns on. In tobacco plants, it turned on after 12 days of heat induction at 39°C.

**Figure 22:**
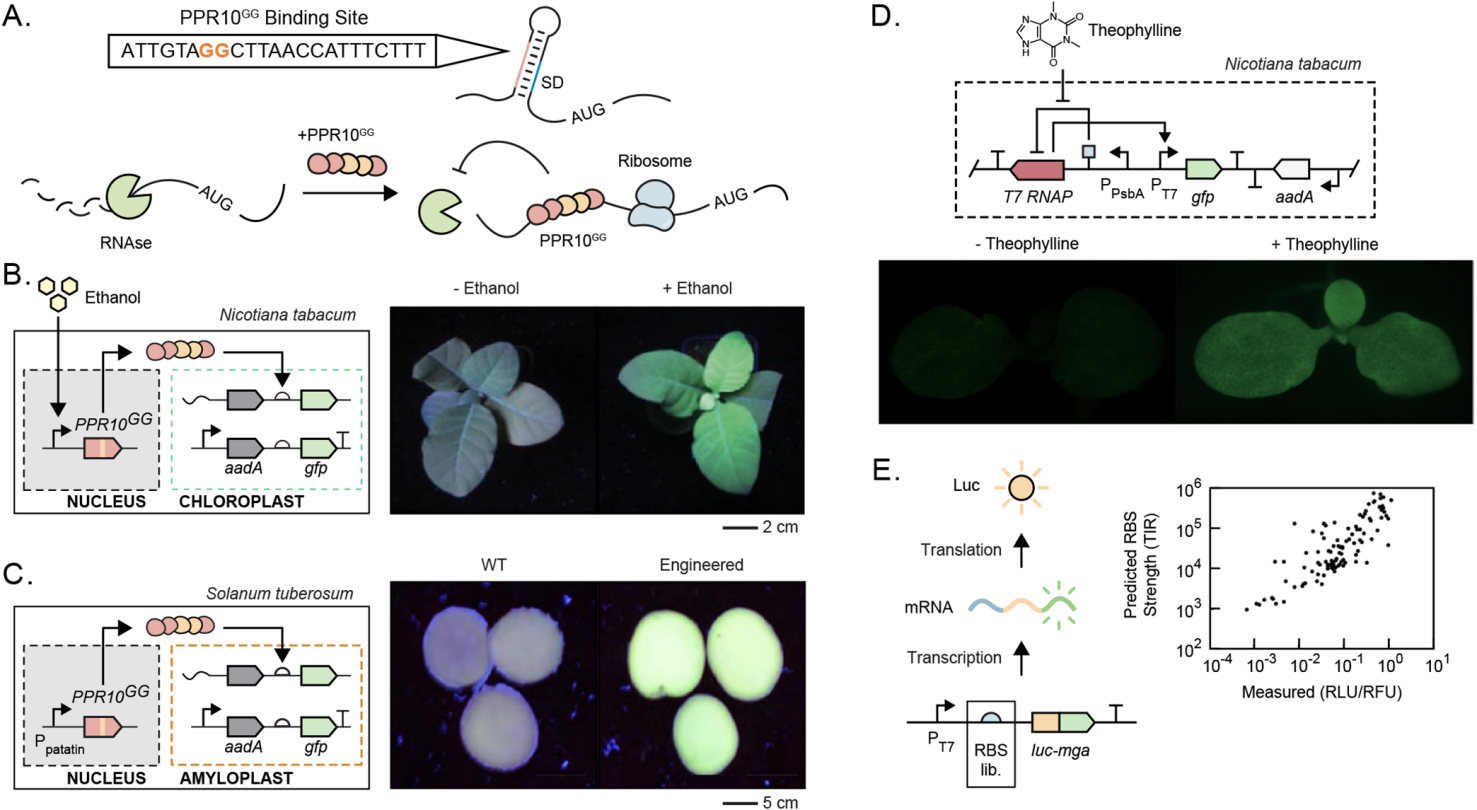
**Transmittal of a signal from the nucleus to the plastid**. **A**. Design of a plastid mRNA containing a PPR10 binding site.^1400^ The maize (pentatricopeptide repeat) PPR10 binds to the 5′-UTR of the *atpH* mRNA and stabilizes the mRNA against degradation by RNase and disrupting secondary structure that prevents ribosome binding. A variant of PPR10 (PPR10^GG^) contains different RNA-binding domains, thus recognizing an orthogonal binding site. SD: Shine-Delgarno. The RNA transcript is shown above the DNA sequence, and *aadA* is the spectinomycin resistance marker. **B**. Inducible expression of a recombinant gene in the plastid by a protein produced in the nucleus.^1401^ PPR10^GG^ was expressed from an ethanol-inducible promoter (P_alc_) in the nucleus. It transports to the plastid, binds to the RNA and increases GFP expression by 20-fold (15% total soluble protein in the leaves after 10 weeks). Plant images reproduced with permission from ref 1401. Copyright 2019, Nature Plants. **C**. Tissue-specific PPR-dependent expression in potato.^1402^ The PPR10^GG^ protein was expressed from a tuber-specific promoter (P_patatin_) and targeted to the chloroplast. Expression of the *gfp* marker was specific to potato tuber for the engineered plant (“Engin.”). WT: wild-type. Plant images reproduced with permission from ref 1402. Copyright 2019, Nature Plants. **D**. Theophyllin-inducible expression in tobacco chloroplasts.^913^ A theophyllin-inducible riboswitch (blue box) was used to control the expression of T7 RNA polymerase (T7 RNAP), which amplified the induction response by transcribing a *gfp* reporter gene. Transformant seedlings were imaged on media +/- 2.5 mM theophylline. Images reproduced with permission from ref 913. Copyright 2015, Nucleic Acids Research. **E**. Using a tobacco chloroplast cell-free system to measure translation of synthetic 5′-UTRs.^881^ The translation rate of a library of synthetic RBS sequences designed by the RBS calculator^311^ was quantified using a firefly luciferase (*luc*) fused to a malachite green RNA aptamer (*mga*). Translation rates were experimentally measured (relative luminescence units (RLU)/relative fluorescence units (RFU)) by normalizing protein concentration (luciferase luminescence) to mRNA concentration (RNA aptamer fluorescence). Data reproduced with permission from ref 881. Copyright 2024, American Chemical Society.

A signal can be transmitted from the nucleus to regulate plastid- and mitochondrial- encoded genes using PPR proteins.^1414^ PPR proteins bind to mRNA to regulate translation by increasing transcript stability and access to the Shine-Delgarno (SD) site of the RBS (Figure 22A).^1414^ Binding to a specific RNA sequence can be programmed by concatenating binding domains, like the modular DNA-binding domains of TALE and ZFP proteins (Section 2.1.1).^1415,1416^ By swapping out two of the repeat nucleotide binding domains in the PPR10 protein, the protein was engineered to selectively recognize a new PPR sequence motif containing the nucleotides GG instead of the wild-type UC.^1400^ The activation of a promoter in the nucleus can be linked to the activation of a gene in the plastid using these proteins. For example, a nuclear ethanol sensor was connected to the expression of a PPR protein that transmitted this signal to the chloroplast and amyloplast (Figure 22B).^1401^ By conditionally expressing the PPR protein from tissue-specific promoters, PPR-dependent translation can be activated in non-green plastids such as the amyloplast in potato (Figure 22C).^1402^

### 2.5 Accelerating Plant Engineering

Technique development in synthetic biology has emphasized accelerating the design-build-test-learn (DBTL) cycle for organism engineering.^1417^ However, plants grow slowly, leading to tediously slow build and test phases.^7^ Constructing new crop cultivars and evaluating them in the field can take years. Even making a stable laboratory strain of *Arabidopsis* or Tobacco can take a year.^1418^ The most immediate impact is that fewer design cycles are possible to optimize the system. This fact, combined with the relatively small number of variants that can be tested for each cycle, reduces the complexity and ambition of the systems constructed. This limitation is due to the rapid increase in the probability of failure as each recombinant gene or genetic part is added to a design.

For microbes and even mammalian cells, computer aided design (CAD) software has helped manage the process (Section 3.6.1).^1352,1419,1420^ These tools encompass algorithms to facilitate DNA design, construction, and application of ML/AI to guide the next round of design.^1421–1429^ Some CAD tools have emerged for plants, but they are far behind in sophistication, are focused on growth simulation and not DNA design, and have not been integrated into unified packages.^1430–1434^ The slow DBTL cycle could also be addressed by developing a method to prototype genetic designs before having to build a complete plant, for example by using protoplasts, single-cell algae, cyanobacteria, or cell-free systems.^1435–1437^

#### 2.5.1 Plant Computer Aided Design (CAD)

Fully realizing the potential of plant genetic engineering will require dedicated design tools that integrate the construction of synthetic regulatory networks, metabolic control, inter-organelle signaling and growth/differentiation. Thinking into the far future, where designs approach the scale of whole genomes, design automation will be required to physically map the phenotypes desired by a user to the DNA sequence. Design automation can address the larger number of constraints that need to be balanced, far beyond what is possible to do “by hand”.^1430,1438,1439^ The output of these design algorithms will automatically connect to pipelines, increasingly robotic, where the physical DNA is constructed, and the plant strains built.^1440^ While plant genetics have generally suffered from a general lack of sequence-to-function models, many CAD tools already developed for bacteria or mammalian cells will continue to be extended to crop plant engineering.

Most software developed for genetic engineering relies on CAD tools that specialize in individual steps of the design process. Some tools allow genetic parts to be manually combined to build up a system; for example, selecting from a set of characterized promoters and terminators to build an expression cassette. Initially, there were few characterized parts from which to choose. Increasingly, an engineer is able to draw from registries of characterized genetic parts, such as JBEI-ICE^1441^ and the Registry of Standard Biological Parts.^1441^ Synthetic biology software packages have not been widely adopted for engineering plants, in part because most designs to date have been fairly simple. As more genetic parts, design rules, and automated strain construction platforms become applied to plants, these can be integrated into the software.

Some CAD tools have been developed to help a user combine genetic parts, often designed to follow assembly rules. Clotho is a software platform that both serves the role of a repository of genetic parts, sequences, and samples and hosts built-in applications that enable genetic design and engineering.^1442^ GenoCAD facilitates the construction of vectors from a set of genetic parts using a user-defined set of rules, such as the order and orientation of proteins tags on a gene-of-interest.^1430^ Golden Gate cloning is a precise part assembly method based on overhangs generated by Type IIs restriction enzymes.^1443^ For plant genetic parts, GoldenBraid uses Golden Gate cut sites but is able to assemble larger and more complex DNA constructs through hierarchical steps.^1444^ GB2.0 is a web-tool that provides both software tools to facilitate the design of DNA sequences for GoldenBraid assembly and a database of characterized plant genetic parts.^1445^

Genetic parts stored in repositories often lack functional information required for them to be used as part of a new design. SynBioHub addresses this problem by providing a user-facing genetic repository that facilitates the sharing of genetic designs encoded in Synthetic Biology Open Language (SBOL).^1419^ SynBioHub allows users to search registries for genetic parts and upload and share genetic designs. Further, it can interface with other applications such as Benchling, which can automate the design of molecular cloning strategies. The output of design software can be connected to experimental planning algorithms, including the control of liquid handling robots, to physically assemble the DNA and transform cells.^1446^

##### 2.5.1.1 Computational Genetic Part Design

Genetic parts can either be selected from a database or generated *de novo* with a computational tool.^1447^ *De novo* part design has been applied to the optimization of metabolic pathways and genetic circuits in prokaryotes.^1413^ Tools have been developed to design RBSs, promoters, and terminators to a strength defined by the user (Section 3.6.1.1.1).^311,735,1448–1450^ Extending such tools to plants is complicated by the inherent complexity of eukaryotic biology.

A potential direct application of the existing tools would be plastid engineering, where transcription and translation mechanisms mimic that of prokaryotes. The RBS calculator is a tool that designs RBS sequences using a biophysical model to obtain a desired strength (translation initiation rate).^311^ In theory, RBSs can be designed for different species by selecting different 16S sequences.^1451^ The UTR Designer uses a similar approach but incorporates additional thermodynamic parameters to quantify alternative mRNA folding dynamics.^1452^ In cyanobacteria, a positive correlation between measured translation efficiency and that predicted by the RBS Calculator and the UTR Designer.^1453^ It was applied to optimize expression of an ethylene producing enzyme in the cyanobacterium *Synechocystis*. It was applied to control expression of a recombinant gene in the tobacco plastid over three orders-of-magnitude.^881^ A transcription-translation cell-free system generated from tobacco plastids was used to measure the translation efficiency of 103 RBSs (Figure 22E).^881^ The characterized library spanned 1300-fold range in expression and had a weak correlation between measured and predicted translation rates.

The efficacy of RNAi (Section 2.2.1.4) is determined by both the on-target and off-target activity of the siRNA derived from the dsRNA/hpRNA design. A suite of computational tools has been developed to aid in the selection of effective, specific, and non-toxic sequences for RNAi.^1454^ The web-tool pssRNAit accepts an mRNA input and screens potential siRNA sequences for off-target homology and toxic sequence motifs, as well as on-target considerations such as target mRNA accessibility and siRNA affinity to RISC complex.^1455^ The output is a ranked list of RNAi sequence candidates for your designated mRNA target along with the expected pool of siRNAs. Other RNAi design software tools include siRNA-Finder,^1456^ AsiDesigner,^1457^ desiRm,^1458^ DSIR,^1459^ and i-Score,^1460^ with each tool using different strategies for on-target and off-target predictions.

##### 2.5.1.2 Codon Optimization

Changing the codons encoding a protein can increase expression of a gene in its native species and facilitate its transfer to species with different codon usages.^1461^ Codon optimization traditionally designs genes by selecting codons corresponding to abundant tRNAs, which differ between organisms. Changing the gene sequence can lead to secondary affects, including altering the mRNA structure and its stability, introducing internal promoters, RNAP/ribosome pause sites, and epigenetic modification sites, all of which affect expression levels. As such, codon optimization can also eliminate (or accidentally introduce) these sequences to affect expression in the same or different host species.^1462^ In fact, these effects have been postulated to have a larger impact on expression than matching codon usage to the tRNA abundances.^1461^ Codon optimization has been used to move genes from other kingdoms to plants and can result in up to 100-fold greater expression.^891,1463–1467^

Codon optimization has also been used to increase recombinant expression in chloroplasts, particularly for large human-derived biologicals that are biased toward human codon usage.^1468,1469^ Eliminating sequence motifs implicated in RNA instability, such as endogenous siRNA binding sites or AT-rich sequence strings for polyadenylation, led to an increase of bacterial and insect gene expression in sugarcane callus.^1470^

##### 2.5.1.3 Protein Design

Computational protein engineering can manipulate existing proteins or even design *de novo* proteins with structures that do not exist in nature.^1471,1472^ Rosetta is a software suite for protein design that has been applied to plant engineering.^1473^ Enzymes can be designed by combining Rosetta’s structure prediction and protein-ligand binding prediction capabilities. For example, it predicted the structure of a cucumber P450 oxidase and calculate all mutations that could be tolerated in the active site. The mutants were constructed and screened to identify a new metabolic route to the sweetener mogroside in cucumber.^1474^

Regulators that can be used to build sensors (Section 2.3.4) can be designed by computationally docking the ligand to the binding site to identify amino acids that facilitate binding (PatchDock), followed by binding pocket optimization by Rosetta.^1331^ Scaffold proteins were engineered to bind to the plant steroid digoxigenin and fentanyl and the binding domain used to build sensors in Arabidopsis.^1331,1332^ *De novo* proteins that bind to pairs of excited state chlorophyll molecules in a way that mimics that natural systems were also designed using Rosetta.^1475^ The ORBIT protein design software was used to engineer a maize lipid transfer protein into a fluorescent biosensor for lipids.^1476,1477^ This was done by using ORBIT to identify disulfide bonds that could selectively destabilize the protein in its unbound form when mutated and conjugating fluorescent probes to these mutated proteins.

##### 2.5.1.4 Computational Metabolic Engineering

Plant metabolic engineering has been aided by computational methods that predict how to make changes to the genome to redirect flux to a desired product (Section 2.2.2). Genome-scale metabolic models (GEMs) capture an organism’s metabolism as a set of stoichiometric reaction equations associated with enzymes. These models can include compartmentalization, for example assigning reactions to the chloroplast (Section 2.2.2.3.1) and interactions between species. GEMs have been developed for *Arabidopsis* ^1478–1480^ as well as many major crops, including maize,^1481–1485^ sorghum,^1485^ barley,^1486^ rice,^1487,1488^ rapeseed,^1489–1491^ sugarcane,^1485^ and tomato.^1492^ GEMs are validated by comparing predictions with experimentally-determined metabolic flux distributions.^1493^ However, gene and protein regulation data are absent and an assessment of *Arabidopsis* GEMs showed that even the most comprehensive models missed many enzymatic reactions, lacked gene-protein-reaction associations, or contained mass and charge imbalances.^1494^ Therefore, the application of these models to guide plant engineering has not been fully realized.

Flux balance analysis (FBA) predicts steady-state flux distributions using a GEM and a set of constraints arising from metabolic reaction stoichiometries and bounded estimates of flux through individual reactions (Figure 23A).^1495^ There are many more fluxes than can be measured, making it a highly under-constrained problem. To address this problem, FBA uses an objective function, such as the metabolite concentrations corresponding to the maximum growth rate, to predict the complete set of fluxes. Optimization software such as COBRA, OptKnock, OptReg, and OptStrain use GEMs and FBA to suggest gene knockouts or even tune enzyme expression levels to maximize the production of a specified metabolite.^1496–1499^

**Figure 23:**
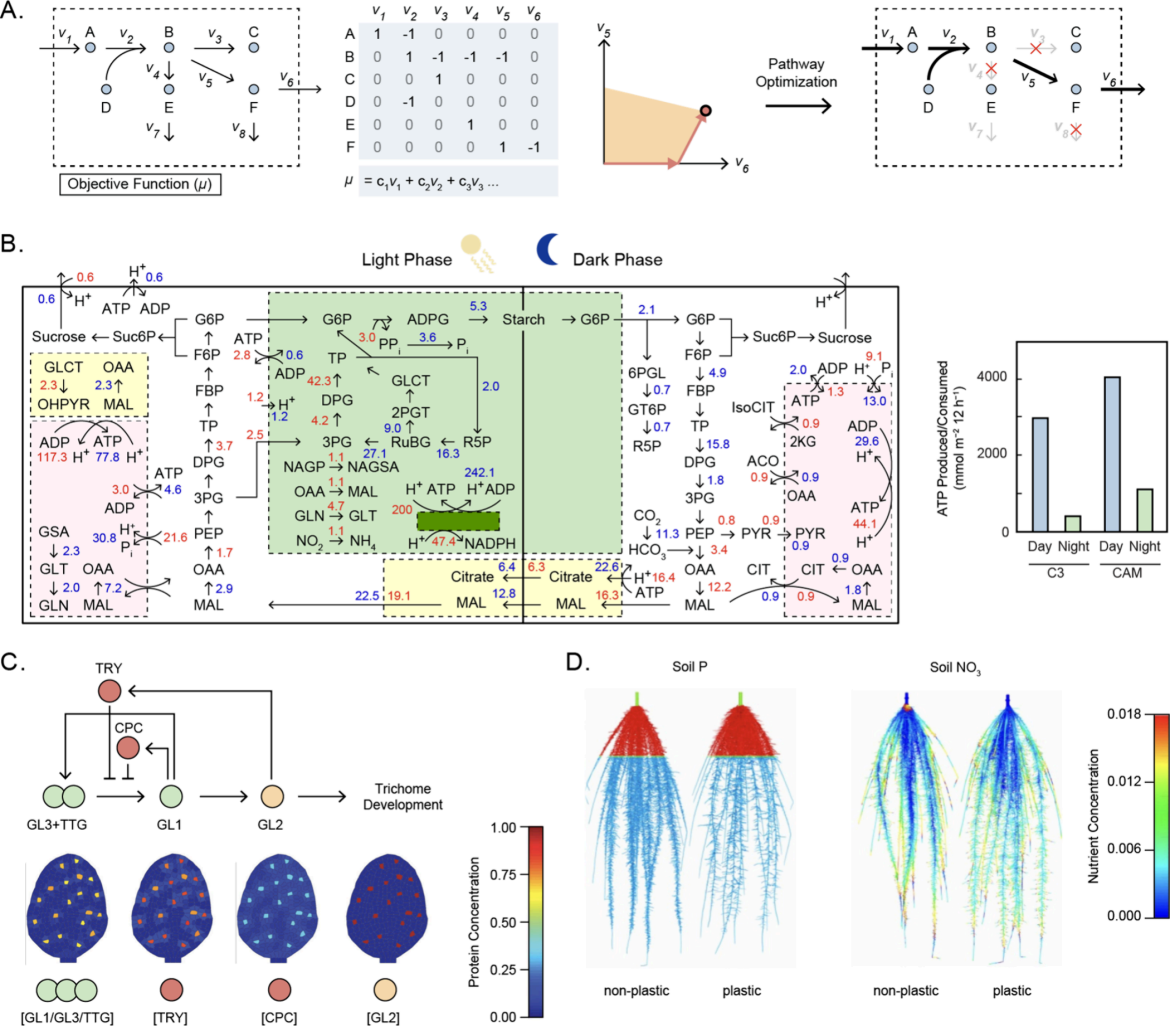
**CAD tools for plant engineering**. **A**. Overview of metabolic modeling using FBA. The GEM consists of metabolites (A, B, C, etc.) and metabolic fluxes (v_1_, v_2_, v_3_, etc.), with the cell indicated by the dashed box. The GEM is converted to a stoichiometric matrix. The objective function *μ* is the weighted (c_1_, c_2_, c_3_, etc.) sum of metabolic fluxes, intended to reflect the metabolite concentrations of biomass. Many combinations of fluxes are consistent with the stoichiometric constraints (orange shaded region), so the set of fluxes that optimizes the objective function (orange arrows) is calculated. Metabolic engineering algorithms seek to remove enzymatic reactions (red X’s) to optimize the flux of a specific reaction, in this case the export of metabolite F. **B**. A compartmentalized FBA model for Crassulacean Acid Metabolism (CAM) photosynthesis for *Arabidopsis*.^1502^ The objective function was the amount of exported sucrose and amino acids. The reactions with the highest flux values are shown with the rate of proton production (blue numbers) or proton consumption (red numbers). To evaluate the effect of CAM photosynthesis on productivity, all ATP consuming and generating fluxes in the C3 and CAM models were summed. Compared to C3 plants, the model of CAM plants predicted that they have higher ATP requirements at night. **C**. Modeling 2D leaf cell development using CellModeller.^1512^ A genetic circuit controls trichome development in leaves to generate precise patterning and frequency. Trichome development genes (G2) are activated following the association of the TFs GL1 and TTG1 mediated by the GL3 regulator protein. The negative regulators TRY and CPC competitively inhibit GL1-TTG1 interactions and are produced in trichome cells to diffuse into neighboring cells. Each leaf is colored by the protein concentration (heat map). Leaf images reproduced with permission from ref 1512. Copyright 2008, Annals of Botany. **D**. SimRoot modeled the effect of phosphate (P) and nitrate (NO_3_) concentration on root architecture with and without root plasticity.^1515,1516^ Root plasticity is a measure of a plant’s ability to adapt root growth and development to local environmental pressures and nutrient availability. The concentrations of P and NO_3_ at the root surface are shown. The growth model predicts that the nutrient concentration results in high root branch density. Root branching is less influenced by NO_3_ because it freely moves through the soil, which prevents high local concentrations. Root images reproduced with permission from ref 1516. Available under a CC BY license. Copyright 2017, New Phytologist.

For plants, FBA models have been used more to understand natural biological processes, as opposed to aiding genetic engineering. They have been used to study tissue-specific metabolism, compartmentalized metabolite transfers, and nutrient transfer between the plant and symbionts in the root nodule.^1487,1488,1500^ Some studies have begun to use them to guide engineering efforts at a high level. For example, FBA was used to simulate the effects of photorespiratory bypass routes on photosynthesis productivity.^1501^ A FBA model that incorporates day and night metabolism showed that introducing synthetic CAM photosynthesis pathways into C_3_ crops could benefit yield, thus demonstrating the carbon-concentrating effects outweigh the associated increase in energy consumption associated with intracellular metabolite transport (Figure 23B).^1502^

Elementary flux mode (EFM) analysis enumerates all possible metabolic routes between a set of substrate and product metabolites.^1503–1505^ This tool is useful for plant engineering, where metabolic pathways are highly branched and redundant. In rapeseed, EFM was used to describe a previously unknown carbon flux pathway used to efficiently convert sugars to oil.^1506^ Although EFM can be powerful for exploring and engineering metabolism, it is computationally intensive and is therefore generally confined to small networks, such as central carbon metabolism.

##### 2.5.1.5 Regulatory Network Design

Simple plant sensors and genetic circuits can be simulated as ordinary differential equations (ODEs) with a solver (*e.g.*, MATLAB) (Section 2.3).^1367^ It is more difficult to design larger circuits involving multiple sensors and a dozen or more regulatory proteins. Cello allows a user to specify a desired circuit function using the Verilog high-level text language used for electronic circuit design.^808,1357,1419,1507–1510^ The software then converts the Verilog text to a wiring diagram using logic minimization and then assigns regulators to each gate within the diagram. The output is a DNA sequence and prediction regarding performance. While it has been mostly applied to bacteria (Section 3.6.1.4), Cello has been extended to yeast (Section 3.7.2.7)^147^ and the design automation software package EQuIP has been applied to mammalian circuit design.^1511^ It could be extended to the plant chromosome or plastid genome by defining a locus to carry the circuit and characterizing a set of simple logic gates at that position (Section 2.4.5.2). Once this is complete, Cello could put the regulators together in many ways to recreate the desired operation and automate the connection of sensors.

##### 2.5.1.6 Multi-cellular Growth Simulation

Plants are multi-cellular organisms with complex tissues that change dynamically during growth. A suite of software tools can simulate 3D growth and morphology, while also incorporating inter- and intra-cellular signaling.^1512,1513^ Some models predict plant resilience to environmental stresses, such as soil moisture, temperature, and dought.^1514–1516^ The most immediate application of these tools in genetic engineering is to link the results of metabolic flux models with phenotypic growth, morphology, and stress response. Multi-tissue GEMs have been developed for *Arabidopsis* that compartmentalize leaf, root, and stem tissues and capture resource partitioning.^1517–1519^ These simulations have been used to predict the whole-plant impact of phytohormones.^1517–1519^

There is potential to use multicellular models to guide the design of more complex plant phenotypes. CellModeller uses cell interaction models to simulate complex morphogenic processes.^1512^ It was used to model trichome differentiation through known gene regulatory networks in *Arabidopsis* (Figure 23C). Another example is SimRoot, which has been used to predict advantageous root phenotypes for nutrient or water-limited growth, such as basal root whorl number, basal root growth angle, hypocotyl-borne roots, and lateral root branching (Figure 23D).^1515,1516^ Whole plant simulations have been getting more sophisticated, including the extension to crop plants.^1520,1521^ One can imagine a future where designers rely on these simulations to predict the phenotypic impact of genetic changes.

#### 2.5.2 Artificial Intelligence (AI) and Machine Learning (ML)

Plant engineering often suffers from an overabundance of data, particularly with -omics methods, and a lack of means to interpret it to drive actionable design choices. To address this challenge, AI/ML models process large quantities of -omics data, annotate genomes, and process images of plants to automatically quantify their phenotypes. While often used interchangeably, ML is a subset of AI algorithms that encompasses the development of computational models to automatically perform complex tasks. These algorithms have the potential to accelerate plant engineering projects. Ultimately, they could be integrated into fully automated plant engineering pipelines to parallelize the design of many more genetic designs than could be created “by hand.”

ML is applied to analyze input data to identify patterns, make predictions, or classify data. ML algorithms implement either supervised or unsupervised learning. Supervised learning uses input data to train the model, which then makes predictions for sets of unlabeled data. Supervised learning was applied to identify pseudogenes in maize without transcriptomic data.^1522^ The input data was a set of maize genes that associated epigenetic markers with expression data and model predicts whether a new gene is a pseudogene.

Unsupervised learning uses unlabeled data to identify patterns or associations and encompass dimension-reduction and clustering algorithms. Neural networks are a form of unsupervised deep learning that can interpret more complex patterns in a dataset, including as nonlinear effects or spatial relationships. Unsupervised learning was used to cluster highly-dimensional single-cell transcriptomic data in order to classify cell types and extract the genetic determinants of root development.^1523^ For plants, ML has been used to for functional gene annotation,^1524,1525^ identify transcriptional start sites,^1526^ predict long non-coding RNAs,^1527,1528^ and find alternative slice site motifs.^1529^

The “learn” step in the DBTL cycle has been developed for high-throughput pipelines for microbial strain engineering (Section 3.6). Tools have been developed to interpret screening data of pathway variants to feed into the design of the next set of strains to be tested.^1530–1533^ AI/ML has the potential to aid the screening of plant phenotypes as part of an automated DBTL pipeline. Characterizing engineered plants can be difficult because plant phenotypes, such as growth morphology or disease tolerance, can be time consuming to assay at scale. Image recognition will assist in the screening of engineered plants. To this end, neural networks have been applied to high-throughput plant phenotyping, including identifying plant stress, growth patterns, plant architecture, morphology, and yield quantification.^1534–1544^

ML can aid the design step of the DBTL cycle. Neural networks have been applied to DNA motif identification, for example to help design promoters or other genetic parts (Section 2.2.1.a). DeepBind and DeepSEA use neural networks to analyze protein-nucleic acid binding data (CHIP-seq, ATAC-seq, etc.) to predict protein binding sites in DNA and RNA sequences.^1545^ This information could be applied to build new sensors (Section 2.3.3) or avoid unintended functional sequences in large designs. ML has been used to extract TF binding motifs that affect chromosome accessibility. For example, a model was trained on DAP-seq data to identify CREs responsible for improved high-salinity response in *Arabidopsis* roots.^1546^ These could aid the design of sensors to program stress response (Section 4.1). ML models have also been trained to predict cleavage and polyadenylation sites in the 3′-UTRs of *Arabidopsis* mRNA and to predict CREs in maize.^1222,1547,1548^

Neural networks can aid the analysis of genome sequencing data to identify native targets for engineering efforts.^1549–1552^ DeepSEA uses a neural network trained on chromatin accessibility data (ATAC-seq) to predict if genetic variants have a functional role in epigenetic regulation and annotate CREs.^1553,1554^ The Plant DeepSEA model was used to identify high-impact SNPs in the promoter region of DEP1, an important gene in rice breeding that regulates leaf and panicle morphology. The SNPs found could be used to inform more efficient breeding strategies.

Regulatory networks and metabolic pathways can be inferred by measuring the correlations between the expression of genes in transcriptomics data. GENIE3 uses ML to build network models by predicting the linkages between genes from RNA-seq datasets.^1555^ It predicted TFs that regulate pathways-of-interest for plant breeding, such as anthocyanin biosynthesis and jasmonic acid biosynthesis.^1229^ Other ML approaches have elucidated plant secondary metabolism; for example, a model was trained to identify *Arabidopsis* enzymes involved in secondary metabolism.^1556^ Similarly, a ML algorithm was trained using metabolomics data to predict previously unidentified metabolic pathways in tomatoes.^1557^

#### 2.5.3 Rapid Prototyping and Automation

Applying principles to optimize the DBTL cycle to plants has been impeded by two challenges. First, transformation is a tedious and labor-intensive low probability event (worsened by the need to achieve homoplasty if transforming plastids). Second, plant growth is slow, especially for non-model plants, including crops of global agricultural interest, and this limits the cycle time. To overcome these problems, several solutions have been proposed including the use of accelerated transformation methods, alternative model plants, cell-free systems, protoplasts, and robotics.

##### 2.5.3.1 Model Plants and Plant Cell Cultures

One way to address these challenges is to pick or create species that are close to high-value plants, but are easier to genetically modify and have faster growth times. For example, *Brachypodium distachyon* often substitutes for wheat, foxtail for millet, and a fast-growing “mini-maize” variant has been developed for corn.^1558–1562^ However, these plants still require months to grow, making them still the slow step of a DBTL cycle.

While *Arabidopsis* has been the workhorse model plant, it still has an 8 - 12 week generation time; thus, it may be beneficial to work with even simpler plants. The moss *Physcomitrella patens* is unique for its capacity for homologous recombination, achieving yeast-like integration efficiencies, and is thus useful for precise genome engineering.^1563–1565^ *Physcomitrella patens* has become a popular for metabolic engineering due to its ease of manipulation, photoauxotrophic metabolism, and cofactor availability.^1566,1567^ To demonstrate the production of structurally diverse terpenes, combinations of diterpene biosynthesis genes were transformed into this species.^1568^ *Marchantia polymorpha*, in the liverwort family, also provides a promising platform for synthetic biology, due to its simple morphology, smaller genome (220Mb), reduced gene redundancy, haploid dominant phase, and ease of propagation and transformation.^1569,1570^ The Parts Registries, MarpoDB and OpenPlant, aid the identification of *M. polymorpha* genes and selection of genetic parts.^1571,1572^ Through *Agrobacterium*-mediated transformation, part libraries can be characterized in the matter of days (Figure 24A).^1573^ *M. polymorpha* can also be individually encapsulated in aqueous microdroplets for high-throughput sorting and quantification, analyzing >100,000 cells per hour.^1574^

**Figure 24:**
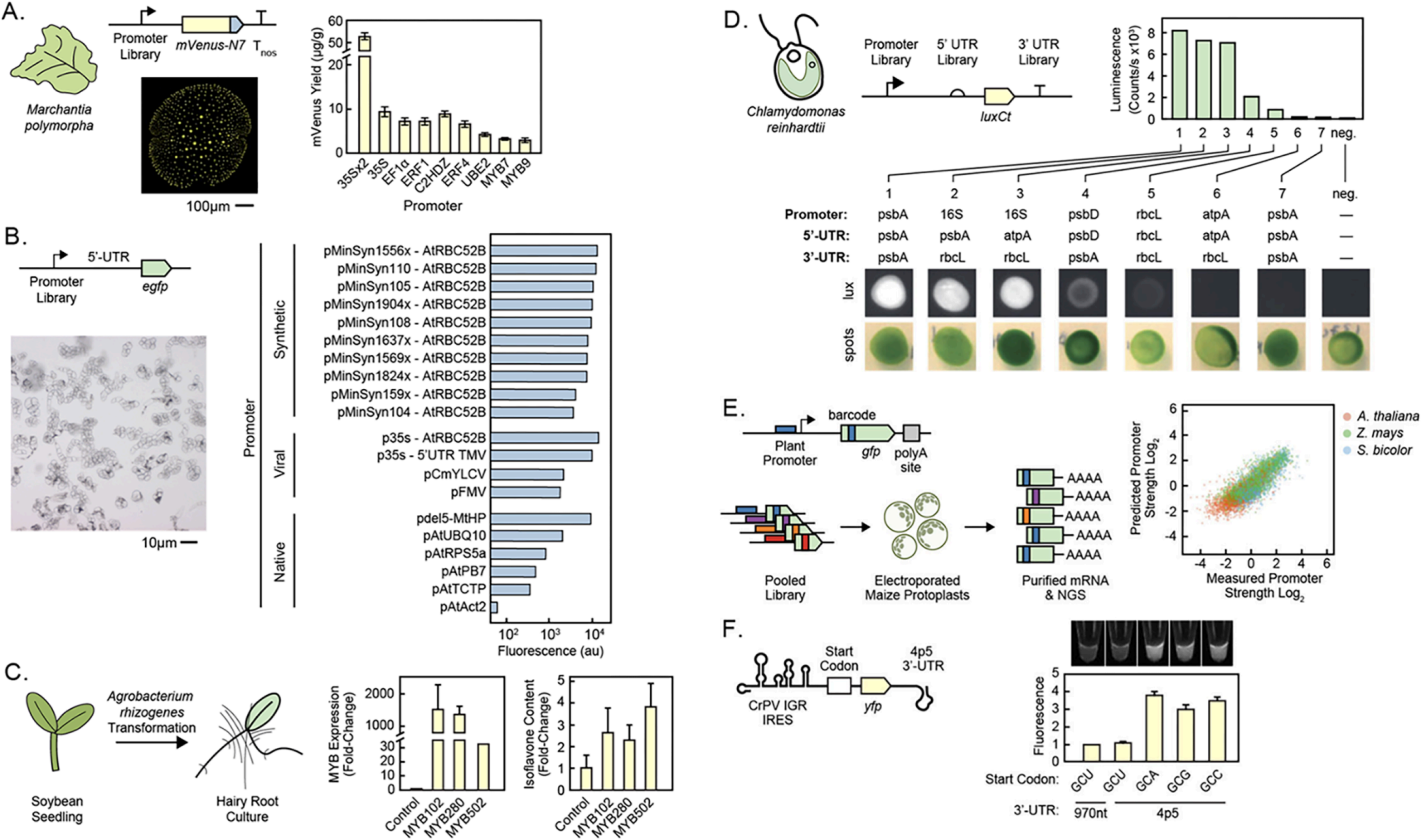
**Rapid prototyping of plant genetic designs**. **A**. Screening promoters using *Marchantia polymorpha*.^1573^ Constructs expressing a *mVenus* fluorescence marker fused to the N7 nuclear localization signal were transformed into *M. polymorpha* using *Agrobacterium*. The promoter library included lant viral promoters (35Sx2 and 35S) as well as promoters found upstream of endogenous *M. polymorpha* genes. Fluorescent microscopy image of a *M. polymorpha* gemmae expressing mVenus-N7 from the endogenous UBE2 promoter. *Marchantia* microscopy image reproduced with permission from ref 1573. Available under a CC BY license. Copyright 2024, Plant Cell Physiology. **B**. Tobacco BY2 cell cultures were used to screen a library of synthetic, viral, and native promoters.^321,1586^ Cell cultures were pelleted and infected with two *Agrobacterium* strains, one of which carried a plasmid. Fluorescence was measured in 96-well microplates 72 h after infection. Synthetic promoters were derived from a previously characterized computationally designed library^356^ and fused to the 5′-UTR of the RbcS2B *Arabidopsis* gene. Viral promoters (p35s, pFMV, pCmYLCV) were either fused to the AtRbcS2B, TMV, or the endogenous viral 5′-UTR. Microscopy images reproduced with permission from ref 1586. Available under a CC BY license. Copyright 2019, Plant Biotechnology Journal. **C**. Soybean hairy root cultures screened the ability of MYB TFs to upregulate isoflavone biosynthesis in root.^1593^ MYB TFs (MYB102, MYB280, and MYB502) were delivered to soybeans via *Agrobacterium rhizogenes*. Expression was normalized to a root housekeeper gene GmELFa (control), and Isoflavone Content was normalized to delivery of an empty *A. rhizogenes* vector. **D**. Screening of chloroplast parts (promoters, 5′-UTRs, and 3′-UTRs) using *Chlamydomonas reinhardtii*.^1599^ Expression was quantified using luminescence (*lux*) in homoplasmic lines. Images of *Chlamydomonas* spots reproduced with permission from ref 1599. Copyright 2011, Plant Biotechnology Journal. **E**. Maize protoplasts were used to screen the activity of a library of *Arabidopsis*, maize, and sorghum promoter elements.^357^ Promoters were cloned upstream of a *gfp* marker tagged with a barcode. Barcode abundance quantified following protoplast transformation was used to measure promoter activity. Promoter activity predictions made by an AI model trained using promoter motifs and activity characterization correlated with experimental measurements. Data figure reproduced with permission from ref 357. Copyright 2021, Nature Plants. **F**. Genetic part characterization using wheat germ CFPS.^1627^ The IRES can initiate translation using various start codons. To quantify translation initiation efficiency, a *yfp* expression cassette downstream of the Cripavirus (CrPV) intergenic region (IGR) IRES was fused to either the complete 970 nt CrPV 3′-UTR or a 36-nt protector region within the native 4p5-10 3′-UTR. Fluorescence images reproduced with permission from ref 1627. Copyright 2019, Bioorganic & Medicinal Chemistry Letters.

Plant cell cultures comprise proliferating, undifferentiated cell suspensions grown in liquid medium.^1575^ The most common plant cell line is Tobacco Bright Yellow 2 (BY2), but suspensions have also been made for carrot, rice, tomato, alfalfa, soybean, and potato, amongst others.^1576–1585^ So-called “plant cell packs” are microtiter plates containing Tobacco BY2 cells that can be transformed at scale, thus simplifying the high-throughput screening of plant cells.^321,1586^ Cell suspensions have been used to manufacture recombinant proteins and natural products in bioreactors.^1587–1589^ They have also been used to characterize genetic parts and metabolic pathways. A set of native promoters was shown to have similar activity in both soybean tissue and a soybean cell culture.^725^ Plant cell packs were used to characterize a library of different promoter-terminator pairs (Figure 24B).^321,1586^ They were used to screen genetic variants for tryptamine production when tryptophan decarboxylase was targeted to different cellular compartments.^321,1586^

Hairy root cultures are generated from the infection of roots by *Agrobacterium rhizogenes*, which causes the overproduction of phytohormones and rapid root proliferation.^1590^ Hairy roots possess the metabolism of normal differentiated plant tissues.^1591^ This system was used to screen combinations of genes from the precursor biosynthesis pathway to identify variants that overproduce the medicinal diterpene tanshinone in the plant *Salvia miltiorrhiza*.^1592^ Soybean hairy root cultures were used to screen TFs for upregulation of isoflavone biosynthesis (Figure 24C).^1593^

##### 2.5.3.2 Surrogate Microbes

Single-cell organisms also can accelerate the DBTL cycle by acting as a surrogate for a complete plant, but the accuracies of their predictions for higher plants are low. The algae *Chlamydomonas reinhardtii* has been used as the model organism to study eukaryotic photosynthesis and chloroplast biogenesis. They are conducive to high-throughput studies. For example, a library of 600,000 barcoded mutants was generated and 58,000 mutants in 121 different conditions were phenotyped.^1594,1595^ *Chlamydomonas reinhardtii* can serve as a platform for plant metabolic engineering and has been engineered to produce diverse diterpenoids.^1596^

Plastid genomes are particularly hard to genetically modify and, therefore, different prototyping species have been proposed. *Chlamydamonas* has been used as a model system to study plastids, as it only has a single one that is easily transformed^1597^ and this has been used to characterize promoters and RBSs (Figure 24D).^1598,1599^ However, algal plastid regulatory signals are very different from higher plants and thus it is usually not a good approach for quantitative characterization.

*Escherichia coli* shares many transcriptional and translational signals with the plastids of higher plants (Section 2.2.1.e). Many promoters work in both hosts. One reason for this observation is that both share the same housekeeping s70 for RNAP. However, it also is an imperfect model. First, translational signals (*e.g.*, the RBS) can differ between the two, making it difficult to use *Escherichia coli* to model an accurate translation rate.^929,1600^ Second, the maturation of mRNA and degradation signals are much different in chloroplasts and change during the day and night, thus complicating the interpretation of results.^947^

##### 2.5.3.3 Protoplasts

The outer wall can be stripped from a plant cell to create protoplasts that are easy to transform and use in high-throughput experiments.^1477,1601,1602^ The complete plants can also be recovered after the protoplast is made. For example, genome-edited cabbage, rapeseed, Chinese cabbage, rice, and wheat have been regenerated from their protoplasts.^1603^ They have been widely used to characterize plant signal transduction, cell phenotypes, and differentiation.^1604–1608^ The key challenge with using protoplasts is that they are difficult to prepare and wide variability has been observed batch-to-batch and between labs.

Protoplasts enable rapid screening of functions relevant for genetic engineering. They have been used to evaluate gene silencing and genome-editing tools because the chromosome is intact and epigenetic enzymes active. Protoplasts can be used with FACS or deep sequencing to characterize synthetic sensors/circuits and genetic part libraries (Figure 24E) (Sections 2.2.1.1.6 and 2.3).^725,1609,1610^ To elucidate sequence motifs that determine promoter strength, the activities of 18,000 Arabidopsis, 34,000 maize and 27,000 sorghum core promoters was measured in maize protoplasts using barcoded transcripts to measure activity.^357^ Two million synthetic CREs were transformed into parsley protoplasts to identify those responsive to stress.^385^

##### 2.5.3.4 Cell-Free Protein Synthesis

Cell-free gene protein synthesis (CFPS) systems reconstitute the cellular machinery for transcription and translation in a mix.^1611,1612^ The mix can either be made by lysing cells or by combining independently-purified proteins to minimally reconstitute these processes (*e.g.*, the PURE system).^1613–1615^ For many bacteria, fungi, and mammalian cell types, CFPSs have become widely used to evaluate many variations of genetic parts and designs,^1616^ to prototype genetic circuits,^1612^ and to identify optimal enzyme levels in multi-step pathways.^1617–1621^ They have been encapsulated in lipid vesicles in to perform high-throughput screening using FACS^1622,1623^ or other liquid handling systems (*e.g.*, the Echo).^1624^ A DNA construct simply must be dropped into the CFPS mix and transcription and translation initiates, thus eliminating the need for transformation and cell growth. One challenge with CFPS systems is that they can exhibit variability due to fluctuations in the component concentrations over time and are sensitive to small changes in the protocol for their preparation.^1625^ They also run out of energy (ATP) and materials (amino acids, nucleotides, etc.) over time, making the responses of some systems difficult to interpret.

There are several ways that CFPS's could be used to prototype plant genetic designs. The first is to characterize large libraries of simple genetic parts, such as promoters. Wheat germ has often been used as an *in vitro* protein expression system and has been used in the context of DBTL pipelines.^1626^ While wheat germ extract cell-free systems are primarily used for eukaryotic protein production, genetic parts have been screened using wheat germ extract to optimize cell-free expression. Wheat germ extract has been used to identify 5′-UTRs that improve mRNA stability and synthetic IRES variants that improve translation initiation (Figure 24F).^1627^ To identify binding sites for endogenous TFs, 647 *Arabidopsis* TFs were individually expressed in wheat germ extract and screened for binding to the 5′-UTRs of two *Arabodopsis* genes.^1627^

Plastid genomes have long been studied using CFPSs, in part because they are difficult to transform. They have been used extensively to deduce regulatory signals involved in transcription and translation in the plastid genome. These experiments often have involved making small libraries of promoters, terminators, mRNA-processing RNAses/protease sites, spicing sequences, and RBSs (Section 2.2.1.f).^882,884,902,903,1628–1632^ While these systems were used to elucidate the basic biology of plastids, another way to interpret the results is that are part libraries whose characterization data could be applied to genetic engineering. A tobacco plastid CFPS was used to quantify the strength of computationally-designed RBSs (Section 2.4.1) (Figure 22E).^881^ T7 RNAP was added to this CFPS to transcribe mRNA containing the RBS to be measured that controlled the expression of a fluorescent reporter. The fluorescence measurements were normalized to mRNA concentration using a malachite green aptamer within the transcript.^1633^ Some care has to be taken in using a parameter value measured using a CFPS system, as differences between *in vitro* and *in plant*a expression have been noted.^931^ They can also be sensitive to lab-to-lab conditions during preparation.^1634^

## 3 Engineering the Soil Microbiota

Plants interact with soil microbes that acquire nutrients, suppress pathogens and pests, and improve stress tolerance.^1635–1642^ Many beneficial crop traits can be obtained by modulating these microbes, either altering which natural microbes are present or engineering them to introduce a new function.

The term “chassis” has been used in Synthetic Biology to describe a strain that can be reliably engineered.^299^ The sheer scale of the soil microbiome provides many potential chassis. A gram of agricultural soil has an estimated 1 mg bacteria (10^9^ cells), 1 mg fungi (10^7^), 10 μg archaea (10^7^), and 0.1 μg viruses (10^8^).^1643^ The specific microbes present depends on the soil chemistry, geography, weather, and agricultural practices.^1644,1645^ There can be thousands of bacterial species in a gram of soil, many of which can be genetically manipulated.^1646^ Filamentous fungi are less easily engineered, but have critical roles in plant health; notably, arbuscular mycorrhizal fungi (AMF) provide nutrients and others defend against pathogens and enhance stress tolerance.^1647^ Archaea have been even less studied, but are critical in agricultural processes, including the impact on climate change through the release of the greenhouse gas methane.^1648–1650^

Plants actively shape their microbiome, both directly through the excretion of metabolites and indirectly by manipulating environmental conditions or recruiting competitive species.^1651–1656^ This enriches the species toward a unique pattern associated with the plant type that can be robust to varying soil types.^1657,1658^ This chemical language is rich and poorly characterized. For example, *Arabidopsis* dedicates 50% of its fixed carbon to make 100s of chemicals excreted from its roots, the composition of which varies under different conditions.^1659,1660^ Networks of interacting species create consortia that are more stable when introduced in the field together and are more resilient to environmental stresses.^1661–1665^ Engineered consortia could support a species carrying a function-of-interest or it could be distributed across multiple strains to reduce its burden.^1666,1667^

For over 100 years, many agricultural products whose active ingredients are living bacteria and fungi have been sold to enhance these effects, including a bacterial insecticide with the unfortunate name “Doom”.^1668–1671^ Engineered microbes have been slower to be adopted, with only a few examples approved to date, most of which involve random mutagenesis, removal of genomic DNA, or the movement of genetic parts from one region of the genome to another, to enhance a function.^6^ However, the potential is huge, as our ability to genetically modify these bacteria has expanded rapidly. With the rise of genome editing, large multi-gene pathways can be combined into a single cell. Further, they can be placed under the control of genetic circuits to regulate when the pathways are active or to create interlocked symbiotic relationships between each other and the plant (Figure 1). Methods are being developed to control microbial persistence in the environment, prototype performance, quantify risk before field trials.^6,1672,1673^ This section focuses on these and future applications as well as the state of methods from Synthetic Biology to engineer soil bacteria, fungi, viruses, and archaea.

### 3.1 Genetically Modified Microbes (GMMs) in Agriculture

Many natural microbes have been marketed to enhance plant growth or protect against stresses. These products have been extensively reviewed elsewhere.^1674–1676^ Examples of commercial inoculants that use natural microbes as biopesticides and fertilizer supplements are from the following genera: *Bacillus* (Nortica, PONCO, VOTIVO, Serenade), *Streptomyces* (Mycostop, Actinovate), *Bradyrhizobium* (Optimize, Vault, Excalibre-SM), *Sinorhizobium* (Optimize), *Azospirillium* (MycoGold, MicroAZ-ST), *Azotobacter* (bio-N, MycoGold). In additional, natural filamentous fungi have been used in products: *Trichoderma* (Excalibre-SM), *Beauveria* (MycoGold), *Penicillium* (JumpStart), and AMF (Mycormax, BioOrganics, MycoGold, BLUEPRINT). Note that bacteria that protect against abiotic and biotic stresses are described in Section 4.1.

All of these examples are not genetically modified. This section describes strains genetically engineered to enhanced performance. Most of these strains have only been tested under laboratory or limited field conditions, with almost none passing regulatory hurdles to be used for agriculture. Reviews have been written regarding the challenges of obtaining regulatory approvals for the commercial release of GMMs.^6,1677,1678^

#### 3.1.1 Microbially-Derived Nutrients

Plants rely on the soil microbiota to convert nutrients to forms that can be taken up by the plant. Fertilizer is a major input to agriculture and critical for maximizing yields. The most energy-intensive input is nitrogenous fertilizer, whose production uses 1% of the world's energy and is up to 55% of the energy input to crops.^1679,1680^ Phosphate is mined to be added to fertilizer, with cheap sources being geographically limited (70% comes from Morocco) and “peak phosphorous” is anticipated in 2040, after which the supply begins to become depleted.^1681^ Potassium, as well as metals and micronutrients, are essential for growth and stress resilience.^1682^ Microbes can be engineered to produce these nutrients, convert them into a form that can be utilized by the plant, or aid their accessibility in the soil.

Nitrogen is limiting for plant growth, because plants are unable to convert atmospheric nitrogen to a fixed form that can be used to build metabolites and proteins. Some bacterial species have the enzyme nitrogenase, which converts N_2_ to ammonia, which can be taken up by the plant. Cereals are generally not able to form associations with N-fixing bacteria and it has been a longstanding challenge to move this capability to them. Different engineering approaches are being taken to modify bacteria and plants. We describe these efforts as a “grand challenge” in Section 4.2.

Orthophosphates (H_2_PO_4_^–^, HPO_4_^2–^) are the preferred form of phosphorus for plant uptake, but much of the phosphorus in the soil is found in inaccessible forms, such as in organic matter, insoluble mineral precipitates, or adsorbed on soil particles. Bacteria can convert phosphorus from inaccessible to bioavailable forms by secreting phosphatase or phytase enzymes to release phosphates from organic matter.^1683,1684^ To this end, 82 phytases were screened in plant-associated bacteria to identify those that increased *Arabidopsis* growth under phosphorus-limited conditions.^1685^ A similar effect can be achieved by secreting organic acids and siderophores to solubilize mineral phosphates. Phosphate solubilization was improved by increasing gluconic acid secretion through the recombinant expression of the pyrroloquinoline quinone (*pqq*) gene cluster in *Pseudomonas fluorescens*, *Burkholderia cepacian*, *Pseudomonas* sp. PSS, and *Herbaspirillum seropedicae*.^48–50^ Citric acid secretion will also solubilize phosphate, which was demonstrated by transferring citrate synthase and citrate transporter genes into *P. fluroescens*.^51^

Iron is required for chlorophyll biosynthesis and deficiencies lead to foliage yellowing and reduced yield. Plants and microbes release siderophores that efficiently chelate and transport iron ions, which would otherwise be insoluble and unavailable for absorption. Siderophores purified from *Chryseobacterium* alleviated iron starvation when applied to tomato roots.^1686^ Further, high siderophore production from *Streptomyces* improved rice and mungbean growth under low iron conditions.^1687^ Replacing the native iron-regulated promoter of the siderophore biosynthesis gene *pvdS* with an inducible promoter resulted in elevated siderophore production and iron leaching.^1688^

Zinc is involved in many metabolic and physiological processes in plants and is required for the activity of some enzymes. *Bacillus* soil isolates selected for their ability to solubilize zinc oxide in halo assays were shown to increase zinc concentrations in soybean likely through the release of organic acids that dissolve insoluble zinc compounds. Other *Bacillus* and *Enterobacter* strains identified for zinc solubilization *in vitro* have been shown to reduce zinc deficiencies in maize, rice, and wheat when inoculated in soil.^1689,1690^ To our knowledge, soil bacteria have not yet been engineered for zinc solubilization.

#### 3.1.2 Hormone Production to Promote Plant Growth

Bacteria can synthesize the phytohormone auxin indole-3-acetic-acid (IAA), which drives cell elongation and division during plant growth, root formation, and leaf development.^1691^ In nature, auxins are produced by over 80% of bacteria in the rhizosphere.^1692^ Overexpressing IAA biosynthesis enzymes in *Pseudomonas* improved root growth in rapeseed.^1693^ Similarly, the transfer of these genes to *Cupriavidus pinatubonensis*, which does not naturally produce IAA, increased *Arabidopsis* root length and weight.^1694^ These genes were placed under the control of a quorum sensor to allow the bacteria to increase in biomass before making IAA. This regulation reduces the metabolic burden of making the molecule before it is needed (Figure 25A).^1694^

**Figure 25:**
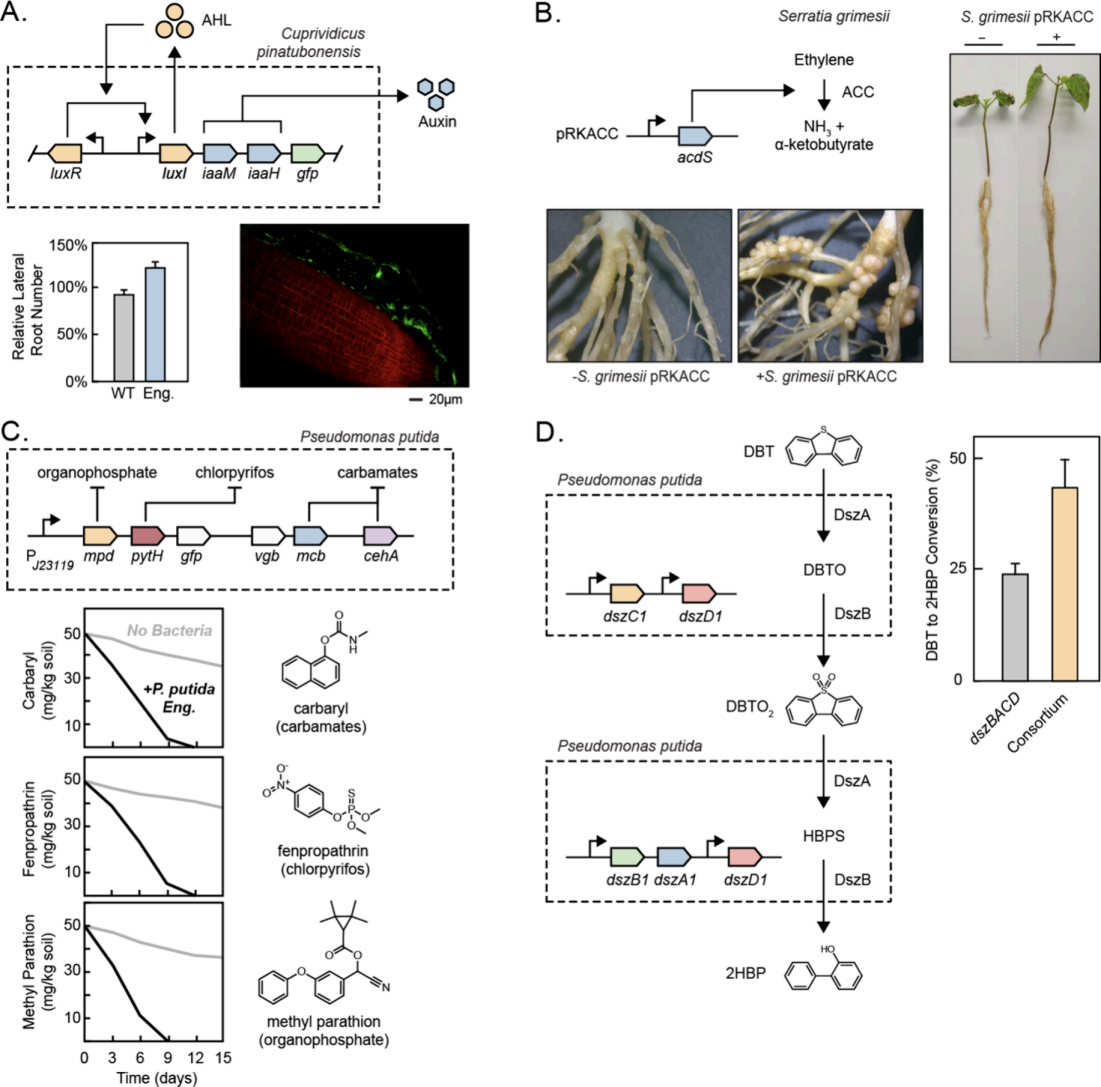
**Engineered bacteria to control and protect plants**. **A**. Cuprividicus strain engineered to control root development through auxin production.^1694^ When bacteria are in the root, expression was turned on at high cell density using a quorum sensor (yellow). *Arabidopsis thaliana* inoculated with auxin-producing *Cuprividicus pinatubonensis* (Eng.) had increased lateral root development compared to wild-type (WT). AHL: acetylhomoserine lactone. Microscopy image reproduced with permission from ref 1694. Copyright 2018, American Chemical Society. **B**. Increased nodulation by a strain engineered to degrade the phytohormone ethylene.^1700^ The recombinant cassette was carried on the pRKACC plasmid. The engineered bacteria increased root biomass (right) and increased nodulation (bottom) in inoculated common bean plants. Images reproduced with permission from ref 1700. Copyright 2018, Letters in Applied Microbiology. **C**. A pesticide-degrading *Pseudomonas putida* strain.^1705^ A constitutively-expressed operon included genes for the degradation of the different classes of pesticides shown. The genes were sourced from *Plesiomonas* (yellow), *Sphingobium* (red), *Rhizobium* (purple), and *Achromobacter* (blue) species. It could degrade six pesticides from different classes of molecules in soil experiments within 15 days. Images reproduced with permission from ref 1705. **D**. Desulfurization of oil compounds by an engineered *P. putida* consortium.^1706,1707^ The pathway was transferred from *Rhodococcus erythropolis*. The two-strain consortium had higher DBT to 2HBP conversion rates than a single *Pseudomonas putida* expressing the entire catabolism pathway (*dszcBACD*).^1706,1707^ Compound names: dibenzothiophene (DBT), dibenzothiophene sulfoxide (DBTO), dibenzothiophene sulfone (DBTO_2_), 2HBP-sulfinate (HBPS), and 2-hydroxybiphenyl (2HBP).

Bacteria can also control plant growth by reducing the presence of the volatile stress hormone ethylene, which regulates plant growth and stress response.^1695–1697^ In legumes, high ethylene levels inhibit nodulation.^1698^ Ethylene production is induced by abiotic or biotic stress, leading to detrimental effects on plant growth in some cases. The enzyme ACC-deaminase degrades an ethylene precursor, thereby reducing its production.^1699^ Beans inoculated with the endophyte *Serratia grimesii* engineered to express ACC-deaminase displayed enhanced nodulation, increased plant growth, and improved disease resistance (Figure 25B).^1700^ Inoculating rice with an engineered endophyte (*Azoarcus*) constitutively expressing ACC-deaminase increased the weight of rice growing in cadmium-contaminated soil.^1701^ Similarly, engineering the banana endophytes *Kosakonia* and *Enterobacter* to express ACC-deaminase to degrade ethylene prevented *Fusarium* wilt disease.^1702–1704^

#### 3.1.3 Protection against Abiotic and Biotic Stresses

Engineered microbes can also confer both biotic and abiotic stress resistance to the plant. Section 4.1.2 describes many of the potential uses to incorporate microbes into systems that can dynamically respond to these stresses. Some natural microbes have been found to protect plants from salt and drought stress. Mechanistically, they perform this function by inducing plant phytohormones, osmolytes, ion transporters and aquaporins, and antioxidation or impeding salt uptake.^1708–1713^ Most cases of their commercial use do not involve genetic engineering.

The first example of a genetically-modified microbe (GMM) that protected a plant was designed to reduce the formation of frost.^1714,1715^ Wild-type *Pseudonomas syringae* contains an ice-nucleating protein that nucleates ice formation and damages plants at temperatures below -5 °C. This problem was addressed by knocking out the nucleation gene to create a strain (Ice^–^) that outcompetes the native strain, thus inhibiting its colonization and frost formation. Field trials testing Ice^–^ were run in 1987 on potato and strawberry.^1714–1717^

Nonpathogenic bacteria promote resistance to various diseases by producing molecules that prime the plant immune system. This mechanism has been used to engineer better biocontrol agents. For example, salicylic acid is known to upregulate plant defenses.^1718^ Inoculating tobacco with a strain of *Pseudomonas fluorescens* engineered to express a biosynthetic pathway for salicylic acid reduced damage caused by tobacco necrosis virus.^1719^

Bacteria can also protect plants against pathogens and insects (Section 4.1.2). Some of these are wild-type strains that naturally produce antibiotics, antifungals, or insecticide compounds. Most GMM commercial products are based on microbes that protect against insects. *Bacillus thuringiensis* or *Pseudomonas fluorescens* engineered to produce the Cry toxin are the active ingredient in Agree, Condor, Design, Foil, Raven, CRYMAX, and MATTCH.^1720–1724^ The Cry toxin is a potent bioinsecticide that kills insects by binding and forming pores in the insect midgut after ingestion.^1721^ Many variants of the Cry toxins have been moved between species in order to change or expand the range of insects targeted.^1725^ Protein engineering has also been applied to the toxins to the same effect.^1726–1729^

Bacteria can also be toxic to fungi and insects by expressing dsRNA that target essential genes when consumed by the pest (Sections 2.3.2 and 4.1.2). Engineered bacteria can also be used to “vaccinate” a plant against future pathogens. Plant defenses can be primed with small peptides (“hairpin proteins”) that are used by some phytopathogens to trigger localized cell death in plants.^1730,1731^ Exposure to hairpin proteins leads to systemic acquired resistance, which protects a plant against later exposures, analogous to the innate immune response in animals. *Bacilli* engineered to express hairpin proteins decreased disease caused by bacterial leaf blight when inoculated on rice.

#### 3.1.4 Toxin Removal from Soil

Microbes can remediate soil pollutants, both to promote plant growth and to reduce the risk to consumers. Heavy metals cause plant tissue damage, stunt growth, and pose a food safety risk.^1732,1733^ Microbes have been engineered to remediate heavy metal contamination by sequestration or chemical conversion. Bacteria have been engineered to sequester cadmium, mercury, lead, copper, and zinc by targeting metal-binding peptides to the cell surface or periplasmic space.^1734–1736^ These strains improve *Arabidopsis* germination and mung bean growth in the presence of heavy metals.^1737,1738^ Another strategy is to sequester heavy metals intracellularly by expressing ion transporters, suppressing efflux systems, and producing intracellular detoxifying molecules, as was demonstrated for cadmium, nickel, cobalt, and arsenic.^1739–1741^ Heavy metals can also be transformed by engineered microbes into volatile forms to eliminate them from soil. For example, recombinant methyltransferase was introduced into *Pseudomonas putida* to convert toxic forms of arsenic to volatile dimethylarsine and trimethylarsine to remove them from soil.^1742^

Chemicals used in agriculture and industry can contaminate soil and water and cause toxicity in plants and humans. Numerous microbes capable of degrading recalcitrant chemicals have been isolated from polluted areas.^1743^ To improve their efficacy, catabolic genes from such microbes have been transferred to other hosts to improve resilience in the environment and expand their activity. For example, a polychlorinated biphenyl (PCB) degradation pathway from *Burkholderia* was expressed in a bacterium that colonizes the alfalfa rhizosphere.^1744^ Genes for organophosphate, pyrethroid, and carbamate pesticide degradation were introduced into *Pseudomonas putida* to create a strain that could simultaneously degrade the pesticides methyl parathion, fenitrothion, chlorpyrifos, permethrin, fenpropathrin, and cypermethrin (Figure 25C).^1705,1745^ Other pseudomonads have been engineered to degrade phenols that are a cancer-causing pollutant from industry and mining.^6^

Combinations of bacterial species performing different degradation steps have been developed. There is a metabolic pathway for the degradation of dibenzothiophene, a sulphur-containing pollutant from coal and fossil fuels. However, one step produces an intermediate metabolite that inhibits upstream enzymes. To address this problem, a consortium of *Pseudomonas putida* strains was designed where one strain performs the first step and a second strain performs the remaining degradation steps (Figure 25D).^1706^ This consortium outperformed a single strain expressing the complete degradation pathway.

When there is no known natural pathway for the breakdown of a pollutant, an artificial one can be created by combining enzymes from different sources or engineering new ones. An example is a pathway designed to remove 1,2,3-trichloropropane, a byproduct of industrial chemical processes, for which there is no known natural degradation pathway.^1746^ A pathway to convert it to glycerol was created by combining an aloalkane dehalogenase from *Rhodococcus rhodochrous* with a haloalcohol dehalogenase and epoxide hydrolase from *Agrobacterium radiobacter*. To improve degradation and reduce toxicity, the aloalkane dehalogenase was mutated and the enzyme levels optimized. Polychlorinated biphenyls (PCBs) are also a byproduct of industrial processes and are difficult to degrade once in the environment. The strain *Cupriavidus necator* can grow on benzoate and biphenyls, but cannot metabolize halogenated versions.^1747^ The recombinant expression of a chlorocatechol metabolizing pathway, and broad-spectrum 2-halobenzoate dioxygenase and toluate dioxygenases in *Cupriavidus necator* conferred the ability to grow on a wide range of mono- and di-chlorinated benzoates and biphenyls.^1747^

#### 3.1.5 Phage Engineering to Manipulate the Microbiome

Phage are bacterial viruses that replicate within their host, ultimately killing it. There is approximately one phage particle for every bacterium in soil, and it has been estimated that 40% of the bacteria in the ocean die daily due to phage attack.^1643,1748^ Since 1924, when Mallmann and Hemstreet used the filtrate of decomposing cabbage (containing phages) to inhibit pathogenic *Xanthomonas campestris*, they have been recognized as potential bio-control agents in agriculture.^1749^ Phages have been tested in field trials for the treatment of *Dickeya*, *Pectobacterium*, *Ralstonia*, *Pseudomonas*, *Xanthomonas* and *Erwinia* infections.^1750,1751^ Phage biocontrol is commercially used to protect potato crops against tuber soft rot (*Enterobacteriacea*), tomatoes from bacterial canker (*Clavibacter michiganensis*), citrus from canker (*Xanthomonas citri*), and apples from fire blight (*Erwinia amylovora*).^1752,1753^ They have also been used to treat leek, lime, grapefruit, orange, mushrooms, radish, lettuce, onion, pepper, rice, cherry trees, and pear trees.^1754^ Phage treatment is not limited to the soil, as they can be taken up by plant roots and translocate to the upper leaves through the plant vascular system.^1615,1755–1757^

Traditionally, phage have been isolated from environmental samples and used without genetic manipulation. Tools from Synthetic Biology could manipulate the phage genome to enhance or control activity for agriculture.^1758,1759^ Phages can be engineered to deliver vectors to re-sensitize bacteria to antibiotics or to deliver Cas9:gRNA to disrupt bacterial virulence genes.^1760,1761^ Incorporating the capsule depolymerase gene of *Erwinia* phage L1 into phage Y2 improved the phage efficiency against *Erwinia* by degrading biofilms and reducing colonization by the pathogen.^1762^ *De novo* DNA synthesis can reconstruct entire phage genomes (Sections 2.1.5 and 3.2.1) and infectious phage have been reconstituted using CFPSs (Section 3.2.5).^1763–1767^ These tools make it possible to reconstitute phage using only sequence data in databases, without needing access to the original infectious material. The potential is vast: the complete viromes of microbiota are being illuminated with sequencing, often finding 100,000’s of viruses in a soil or aquatic sample.^1768^ Mining these phage genomes using *de novo* DNA synthesis (Section 2.1.5.) will lead to the discovery of many with the potential for biocontrol, whose efficacy and specificity could be further enhanced with genetic engineering.^1769^ Powerful AI tools have been used to optimize phage for the food industry and generative AI has been applied to design *de novo* phage genomes, but they have not yet been applied to problems in agriculture.^1770–1772^

Phages generally target specific bacterial species.^1773^ Unlike broad-spectrum antibiotics, they can target individual species inhabiting a complex microbiota without impacting beneficial microbes.^1774^ They can also be engineered to change their host range. In part, species specificity comes from the tail protein binding to the surface of the bacterium. By swapping the tail appendage gene of a phage with related genes, it could be retargeted to new pathogens.^1775,1776^ The phage genome can also be manipulated to improve biocontrol properties; for example, by removing lysogenesis genes to enhance the killing of target species.^1777^

A challenge for effective phage biocontrol is the poor persistence in the field, due to UV exposure and other environmental factors.^1778,1779^ Engineering phage to have protective surface phospholipids can increase their survival in harsh environmental conditions. Another approach is to use carrier bacteria, rather than the purified phage themselves, that encode the phage in their genome. Once the bacterial carriers colonize an environmental niche, the phage are continuously propagated. A virulence-attenuated mutant of *Xanthomonas perforans* was used as a persistent carrier to produce phage on tomato foliage in both greenhouse and field conditions.^1755^

There is a constant evolutionary race between phage and bacteria. Bacteria can become resistant to phage through various mechanisms, including CRISPR/Cas immunity, abortive infection (cell death before replication), blockage of phage DNA replication, and restriction-modification systems.^1763–1767^ The rapid acquisition of resistance has frustrated their use as human therapeutics and biocontrol agents in agriculture.^1780^ For example, the use of phage to treat humans for *Escherichia coli* diarrheal infections worked well the first time, but subsequent treatments were ineffective due to the rapidly acquired resistance.^1780^

There are some genetic engineering approaches to circumvent resistance. Incorporating anti-CRISPR genes into the engineered phage genome can delay or prevent the rise of bacterial resistance.^1750^ Genome-scale screens can identify host proteins that are required for phage infection and are potential resistance hot-spots. Engineering phage infection to be independent of these host proteins or including phages with different host requirements in a phage cocktail can remove potential routes of resistance.^1781^

#### 3.1.6 Manipulating the Plant Microbiome

Plants can curate a favorable microbiome composition and induce beneficial microbial functions.^1782–1784^ This control is implemented through the excretion of chemicals from their roots that encourage colonization of some species, while inhibiting others. For instance, *indica* rice recruits a different microbiota than *japonica* rice that is more diverse, more abundant in nitrogen cycling genes, and linked with better nitrogen use efficiency by the plant.^1785^ Rather than relying on natural variability or selection, plants can be genetically engineered to change the microbial composition of their roots. By mutating TFs to downregulate the plant immune response, *Arabidopsis* was engineered to recruit an alternative microbial composition.^1786^

Plants can also be engineered to excrete chemicals from the root that alter the microbial composition. One approach exploited opines, which are naturally part of an *Agrobacterium* infection. DNA injected by this pathogen causes it to release carbon/nitrogen rich opines that are catabolized by the bacteria.^1787,1788^ Engineered opine excretion from *Arabidopsis, Lotus*, and Tobacco skews the microbiome composition toward opine-consuming species (Figure 26A).^1789–1795^ Genetic sensors and catabolic pathways have been transferred to soil bacteria so that they can respond to opines and consume them as a carbon/nitrogen source.^1794,1796^ Similarly, rice has been engineered to excrete larger amounts of the natural flavone apigenin (Figure 26B).^1797^ The application of these approaches to improving microbial nitrogen delivery to the plant are described in Section 4.2.3.

**Figure 26:**
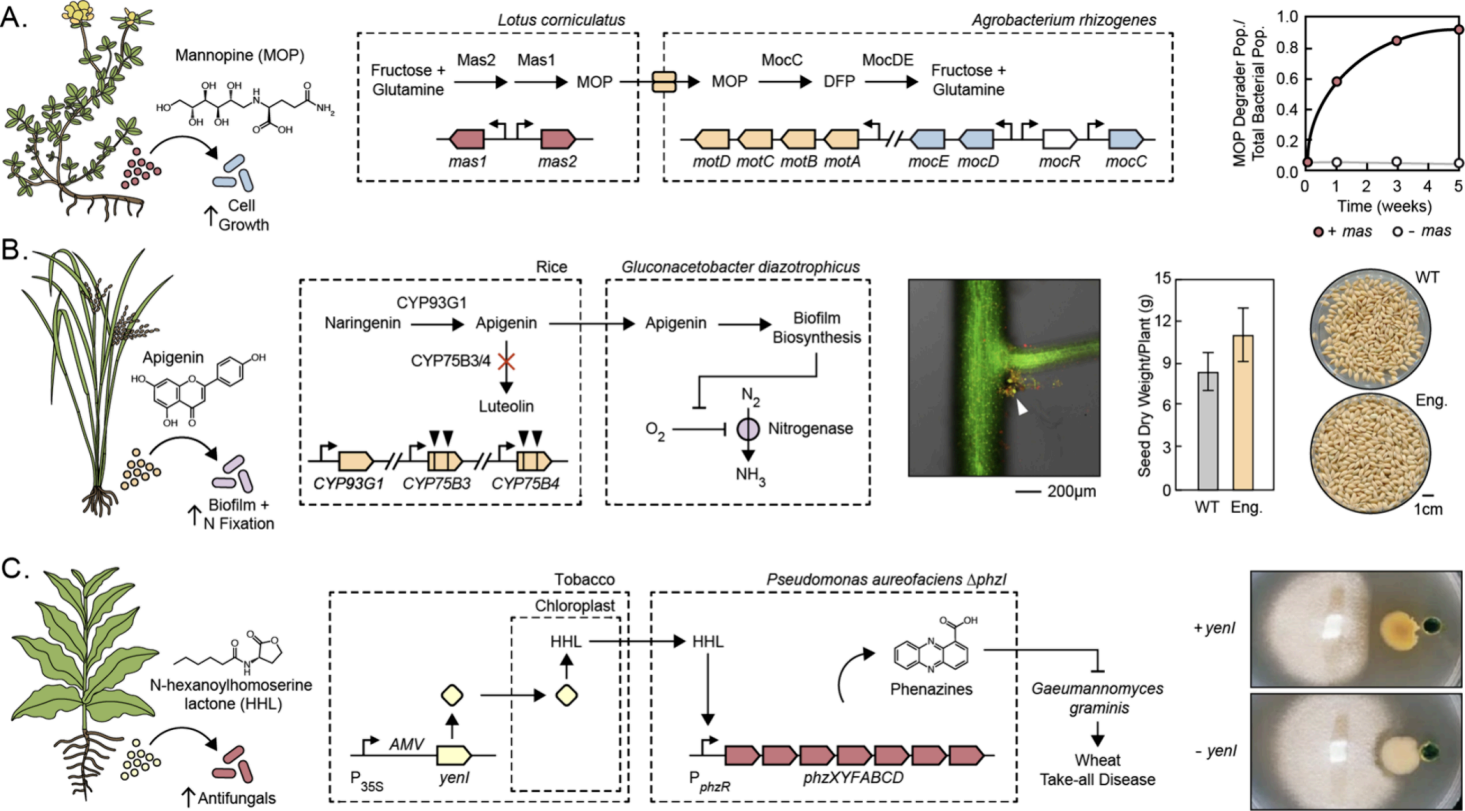
**Manipulating the plant microbiome through plant engineering**. **A**. Opine production to selectively enrich opine-catabolizing bacteria in the rhizosphere.^1792^ The model plant *Lotus corniculatus* was engineered to produce the opine mannopine (MOP) by introducing the biosynthesis enzymes *mas1* and *mas2. Agrobacterium rhizogenes* carrying a plasmid expressing MOP catabolism gene (*motABCDmocCRDE*) can degrade mannopine to support cell growth. When a community of two *Agrobacterium rhizogenes* strains with and without the catabolism plasmid were inoculated into soil at a 1:9 starting ratio, the ratio of MOP degrading bacteria to the total bacterial soil population increased only in pots growing MOP-producing *Lotus corniculatus* plants (*+mas*) but not control plants (*−mas*). **B**. Increasing biological nitrogen fixation and grain yield by manipulating a flavone biosynthetic pathway.^1807^ The flavone apigenin and its derivatives potently induced biofilm formation and increase nitrogenase activity in the bacteria *Gluconacetobacter diazotrophicus* by maintaining low oxygen concentrations. The knock out of two genes encoding flavonoid 3′-hydroxylases (CYP75B3 and CYP75B4) rerouted flux toward apigenin and away from competing pathways. Engineered plants induced expression of a double fluorescently tagged protein biofilm matrix marker (*PgumD::gfp, gumD-rfp*) in *Gluconacetobacter diazotrophicus* (white arrow). Engineered rice inoculated with *Gluconacetobacter diazotrophicus* has higher grain yield as measured by seed dry weight per plant. Fluorescence image and seed image reproduced with permission from ref 1807. Copyright 2022, Plant Biotechnology Journal. **C**. Inducing biosynthesis of antifungals in soil bacteria by production of quorum molecules in tobacco.^1808^ The soil bacteria *Pseudomonas aureofaciens* produces three phenazine antifungals that target the wheat fungal pathogen *Gaeumannomyces graminis* var tritici. The pathways are natively controlled by the quorum molecule N-hexanoylhomoserine lactone (HHL), and the *Pseudomonas aureofaciens* mutant (Δ*phzI*) is unable to produce HHL. A tobacco plant expressing a chloroplast targeted enzyme YenI from *Yersinia enterocolitica* produces HHL. Leaf extract (green dot) from engineered plants (+*yenI*), but not from the control (*−yenI*), induces phenazine production in colonies of *Pseudomonas aureofaciens* Δ*phzI* (yellow colonies) to inhibit growth of *Gaeumannomyces graminis* (white colonies). Agar plate images reproduced with permission from ref 1808. Copyright 1999, Nature Biotechnology.

Gram negative bacteria communicate using acetyl-homoserine lactones (AHLs) that diffuse freely through the membrane and bind to regulatory proteins.^1798,1799^ These “quorum systems” connect gene expression to the density of bacteria or are used to detect the presence of competing or symbiotic species. They can control many genes, including the production of antimicrobials, biofilm formation, chemotaxis, and virulence programs.^1800,1801^ In agriculture, they are important for the colonization of roots and the induction of endosymbiosis or pathogenesis.^1802–1805^

Plants have been engineered to synthesize acetyl-homoserine lactones (AHLs) to hijack these bacterial communication systems with beneficial effect. Tobacco plants engineered to synthesize N-hexanoylhomoserine lactone and N-(3-oxohexanoyl)-L-homoserine lactone secrete these molecules from their roots (Figure 26C).^1806^ When sensed by soil bacteria, these molecules induce cell wall degrading enzymes and phenazine antibiotics that can alter the composition of the microbiota. Bacteria have also been engineered to recombinantly express the enzyme AiiA, which degrades AHLs, to interfere with pathogenesis (Section 3.1.4).

#### 3.1.7 Synthetic Symbiotic Plant-Bacteria Relationships

A symbiotic relationship is defined by a long-term, close association between two species. The symbiotic relationship is mutualistic when the interaction is beneficial to two or more organisms that support each other’s growth. For example, two species can be interlocked if each makes a metabolite that the other needs but cannot produce. Natural mutualistic relationships exist between plants and bacteria, for example when the bacterium fixes nitrogen from air, which is needed by the plant and rewarded with a metabolic carbon source (Section 4.2.4).

Mutualistic relationships between plants and microbes can aid in obtaining regulatory approval for their release.^1809,1810^ If it can be shown that the bacterium only associates with the target plant, not weeds, and dies when the plant is removed, this reduces risks associated with persistence in the environment. The release of genetically-modified *Sinorhizobium* that increases nitrogen delivery to alfalfa passed regulatory approvals, in part, because it was shown that the bacterium cannot associate with weeds and was eliminated from the field when the crop was removed (Section 4.2.4.2).^1809,1811–1813^

Synthetic obligate mutualistic relationships could be designed from scratch, where the microbe and plant each make a nutrient that the other needs.^1814,1815^ Steps have been taken toward this goal, where barley has been engineered to produce rhizopine as a carbon source and *Azorhizobium caulinodans* has been engineered to respond to this signal and fix nitrogen, which can be taken up by the plant.^1816,1817^ If the plant can utilize the nitrogen and the bacterium could catabolize the rhizopine, this would complete a mutualistic relationship. However, to be fully orthogonal from native microbe:plant relationships, both molecules would have to be in a chemical form that cannot be consumed by those species and is entirely absent in the rhizosphere. However, using such a chemical would likely face additional regulatory scrutiny as it potentially introduces a new chemical into the environment and food supply.

### 3.2 Engineering Tools for Soil Bacteria

Numerous tools have been developed to aid microbial strain design and construction.^808^ The scale of engineering projects has increased dramatically with the rise new techniques to edit the genome and insert large DNA payloads into specific sites. New plasmid systems address their instability and remove the requirement for a selectable marker. *De novo* DNA synthesis makes it possible to access and recode large multi-gene constructs, or even whole genomes. Creating multi-gene constructs requires access to libraries of characterized parts, such as promoters, RBSs, and terminators. Most of this work has been focused on model organisms, especially *Escherichia coli*, with much less being applied to agriculturally-relevant species, such as Rhizobia, Pseudomonads and Bacilli. This section describes the tools that are available to genetically manipulate soil bacteria.

#### 3.2.1 Stabilized Plasmids

Plasmids are commonly used to introduce DNA into bacteria because, for most species, they are much easier to manipulate and transform than making changes to the genome.^1818,1819^ Different plasmid origins have different replication dynamics and the number of copies per cell ranges from a few to hundreds. Plasmids with compatible origins can be used together in one cell.^1820^ Early plasmids were selected because they were unstable, due to concerns in the 1970's regarding their propagation in the wild.^1821^ Thus, most require a selection marker, such as an antibiotic gene, without which the plasmid is quickly lost. For agricultural strains, using antibiotics in the field is infeasible, and the release of antibiotic genes into the environment is a regulatory concern. In addition, these plasmids can have surprisingly wide distributions of copy numbers (plasmids per cell) across a cell population, making the design of a genetic system unreliable.^730^

Plasmids rely on host machinery to replicate, and some species may be missing essential components, thus blocking replication. Broad-host-range plasmids have all of the necessary components to replicate across a wide range of species.^1822,1823^ Common broad host range plasmids include pBBR1, RK2/RP4, RSF1010, and RO1600, which have been used in various Gram-negative species, including *Escherichia coli*, *Rhizobium meliloti*, *Pseudomonas putida*, *Pseudomonas aeruginosa*, *Agrobacterium tumefaciens*, *Azotobacter vinelandii*, *Rhizobium tropici*, *Cupriavidus necator, Azorhizobium caulinodans*, *Pantoea ananatis*, and *Pseudomonas protegens*.^1796,1824–1830^ The RSF1010 origin was tested for stability in *Pseudomonas syringae* and only 2% of the cells lost the plasmid in sterile soil after 130 days at ambient temperature.^1831^ It has been suggested that plasmids may be stable in soil because of the reduced growth rates, as most plasmid loss occurs during replication.^1832^

Certain features improve plasmid stability and reliability for carrying DNA in soil bacteria. Some origins use an active partitioning system to push plasmid copies to opposite sides of the cell prior to division.^1833,1834^ The pSC101 *par* locus uses active partitioning and is stable in *Escherichia coli*, with almost all of the cells retaining the plasmid after 100 generations in the absence of selection.^1835–1838^ “Addiction systems” can stabilize an otherwise unstable origin.^1839^ They encode a toxin-antitoxin pair, with the toxin degrading slowly and the antitoxin quickly. If the daughter cell does not inherit the plasmid, then the antitoxin degrades, but the toxin remains and kills the cell. An addiction system has also been developed for *B. subtilis* that stabilized a plasmid for 100 generations.^1840^ The broad-host-range RK2 plasmid has both active partitioning (*par*) and an addiction system that together contribute to a highly-stable system.^1824,1826–1828,1841^ Stabilized mini-RK2 have been constructed by removing unnecessary native DNA.^1825^ A mini-RK2 replicon in *Rhizobium meliloti* was maintained in 100% of the cell population for 100 generations in culture and 4 weeks on alfalfa.^1824^

Plasmids have also been stabilized by knocking out genes on the host genome to introduce a synthetic auxotrophy and providing the complementary gene on a plasmid. GeneGuard is a host-plasmid platform that moves plasmid replication genes into the host genome and essential metabolism genes *thyA* and *dapA* onto the plasmid.^1842^ Essential genes required for the synthesis of amino acids, redox factors, and intermediates of glycolysis have also been transferred to plasmids for this purpose.^1843–1845^ Plasmid stabilization strategies that rely on metabolic auxotrophies can suffer from small populations of cells that lost their plasmids but remain viable by feeding on metabolites secreted by plasmid-harboring cells.^1846^ This problem can be addressed by transferring essential proteins for transcription or translation, which cannot be transferred to neighboring “cheaters.” For example, when the essential transcription initiation factor gene *infA* was removed from the *Escherichia coli* genome and placed onto a plasmid, this led to stable plasmid maintenance and reduced cell-to-cell variation in plasmid copy number.^1846,1847^

#### 3.2.2 Laboratory Strain Evolution

Random mutagenesis has been frequently applied to improve the properties of strains of agricultural importance. It is particularly important in this context because these strains are not regulated as recombinant microbes, easing their regulatory approval.^6^ Some background on directed evolution applied to enzymes and pathways is provided in Section 2.2.2.5 and would be relevant for optimize biosynthesis in microbes as well. Here, we review several more modern approaches to creating diversity to the genome and screening for improved functions.

##### 3.2.2.1 Adaptive Laboratory Evolution (ALE)

ALE is applied to optimize a strain or genetic construct by coupling its amplification to a desired phenotype.^1848^ Coupling can be the overproduction of a metabolite or growth under specific conditions. ALE can optimize strains even without prior knowledge of the system and is therefore a powerful technique for optimizing poorly understood biological phenomena or non-modal organisms.

ALE requires a selection scheme that couples the desired phenotype with the growth rate. One approach is the repetitive growth and dilution of a strain under environmental conditions where improved strain performance is desired. A *Pseudomonas putida* strain was engineered to catabolize xylose and galactose, which are abundant in biomass, but the wild-type strain grew poorly on them.^1849^ By continuously propagating the strains in cultures where glucose was slowly replaced by galactose as the sole carbon source, *Pseudomonas putida* evolved high rates of galactose import and catabolism to recover its growth rate. The selection conditions can be for a complex environment, rather than nutrient preference. For example, *Bacillus* and *Pseudomonas* strains were evolved for better root colonization through repetitive cycles of isolation and inoculation onto *Arabidopsis* roots.^1850–1852^ Mutations were discovered that increased carbohydrate consumption and improved biofilm formation.

The production of a chemical product can be improved with ALE, but this requires a selection that couples its concentration to the growth rate. This can be done by building a genetically-encoded sensor (Section 3.3) to the expression of an essential gene (positive selection) or antitoxin (negative selection). To increase the production of the plant flavonoid naringenin in *Escherichia coli*, the naringenin-responsive TtgR repressor was used to control a dual selection.^1853^

##### 3.2.2.2 Targeting Mutagenesis to Genomic Regions

The pace of evolution can be accelerated by increasing the mutation rate. There are many methods to control the mutation rate, including chemical mutagen concentration, UV/radiation levels, or the induction of low-fidelity DNA polymerases.^1854,1855^ There is a limit to how high the mutation rate can be raised across the entire genome; while most mutations are neutral, deleterious ones will eventually outpace those that are beneficial. As a result, techniques have been developed to target mutagenesis to specific regions. For example, the system to be mutagenized can be encoded on a plasmid that uses an error-prone DNA polymerase for replication.^1856,1857^ Instead of replacing regions of the genome with a single defined DNA sequence, MAGE could also diversify a region using pools of degenerate DNA oligos to build a library to be screened.^1858^

The EvolvR technique directs mutations to specific regions of the genome.^1859–1864^ It uses genome-targeting ability of nCas9 (nickase) to direct an error-prone DNA polymerase to a specific site determined by the sgRNA. Random mutations occur in a 1-350 bp region around the targeted site, the width of which is determined by the processivity of the DNA polymerase.^1861–1864^ There is a tradeoff between the length of the mutated region and the mutagenesis rate. Error rates can be 8 million-fold greater than wild-type cells at the nick site and then decline further from the site.^1859,1860^ EvolvR has been used in *Pseudomonas putida* to evolve the activity and expression of ornithine cyclodeaminase to increase proline production.^1865^ The mutagenized region can be extended by tilling gRNAs.^1859^ Alternatively, MutaT7 techniques utilize a T7 RNA polymerase fused to a mutagenic protein, such as a DNA deaminase, to target mutagenesis to a region downstream of a T7 promoter.^1863,1866–1868^

#### 3.2.3 Genome Editing, Recombineering, and Complete De Novo Synthesis

Strain engineering requires DNA payloads be inserted into the genome or introduced on a plasmid. This step is trivial for model organisms, such as *Escherichia coli* or *Bacillus subtilis*. However, for non-model bacteria, many methods for transforming recombinant DNA are difficult, need extensive optimization, and can only be performed in specific strains.^1869,1870^ Just physically getting into the cell is not enough. Once there, integrating the payload into the chromosome can be blocked by a lack of host cofactors, native restriction endonucleases, or other host defenses.^1871–1873^

##### 3.2.3.1 Advanced Conjugation Methods

Conjugation, where one bacterium transfers its DNA into another through a physical interaction, is a means to deliver DNA payloads into cells.^1874,1875^ The integrative conjugation element (ICE) from *B. subtilis* was engineered to create a system (XPORT) to efficiently transform Gram positive soil bacteria (Figure 27).^1876,1877^ The advantage of ICE as conjugation system is that it can deliver large DNA payloads (100 kb+) and has very broad species range. XPORT was developed to eliminate the possibility of continued propagation and to reduce the size of the DNA containing conjugation machinery that also has to be transferred. A DNA payload can be inserted into XPORT and then transferred to many Gram-positive species, including metabolic pathways, sensors, or nutrient production.^1876,1877^ This system has been used to move reporters, biosynthetic pathways, sensors, and nitrogen fixation gene clusters (Section 4.2.2) into diverse soil bacteria.^1878–1880^ XPORT has also been used to transform mixed populations of species directly in soil (Section 3.4.8).

**Figure 27:**
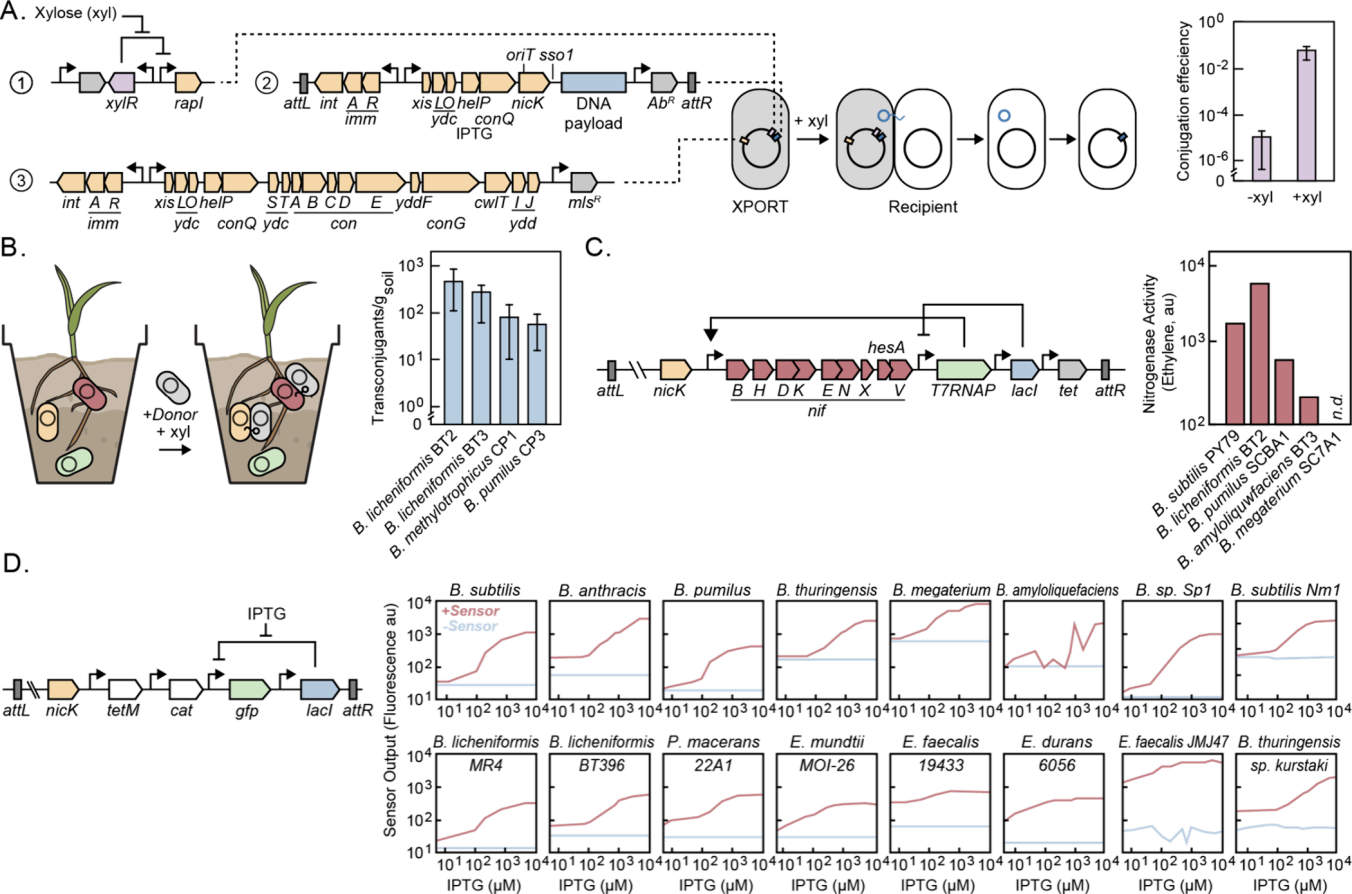
**XPORT transport of DNA payloads into soil bacteria**. **A**. Design of the XPORT conjugation strain.^1876^ The DNA transfer machinery is based on ICE (orange), the minimum amount of which is co-located for the transfer of a DNA payload (blue). The system is controlled by a xylose (xyl) sensor (purple). The ICE region not necessary for transfer was moved to a different location in the genome (3) to avoid propagation. Conjugation efficiency was measured as the number of transconjugants divided by total donor colony forming units (CFU). **B**. Direct transformation of four species in soil by XPORT. Potting soil was inoculated with six Gram positive Bacilli before xyl-induced XPORT was added to the soil. Transformation was detected in six of the recipient strains after two weeks. **C**. Transfer of a nitrogenase gene cluster across Bacillus species using XPORT. A minimal *Paenibacillus nif* cluster was transcribed from the T7 RNAP promoter. The nitrogenase activity of the bacteria containing the gene cluster are shown (ethylene reduction assay). n.d.: no activity observed. **D**. IPTG sensor was conjugated into sixteen gram-positive strains using XPORT. The genetic construct containing the sensor was identical across the strains. The red line shows the strain containing the sensor as compared to the wild-type strain (blue line). The measurements show the medians of flow cytometry experiments.

##### 3.2.3.2 Site-Targeted Delivery of DNA Payloads

Many transposons, such as Tn5, *Mariner*, or Mu, can deliver DNA payloads to random sites in the genome.^1881^ However, random insertion often leads to the disruption of endogenous genes and position effects in expression and is therefore usually reserved for mutagenesis or probing position-dependent expression.^150,1882^ Some transposases and integrases target specific sequences.^1876,1883–1887^ For example, the Tn7 transposon targets the highly-conserved attTn7 site.^1883^ Similarly, phages use integrases that recognize *attB* sites to insert phage genomes into bacterial chromosomes. Site-specific serine integrases have been used to deliver DNA payloads because they have short *attB* sites (<50bp) and do not require host-specific factors.^1888^ They have been used in many agriculturally-relevant species, including *Pseudomonas*, *Agrobacterium*, *Rhizobiua*, *Ralstonia*, *Pantoea*, *Erwinia*, *Yersinia*, *Dickeya*, and *Photorhabdus*.^1883–1885,1889–1892^

“Recombineering” uses homologous recombination to insert a single or double-stranded DNA template with the desired modification into the genome.^1893^ The DNA payload is targeted to a particular genomic location by flanking it with homology arms that match the targeted site. In some species, including *Acinetobacter*, *Bacillus*, *Bradyrhizobium*, and *Azotobacter*, the native recombination machinery is sufficient to integrate homologous DNA sequences, so only the DNA needs to be provided.^1894–1898^ In others, phage recombination proteins are required to catalyze the insertion; the most commonly used is the λ red system.^1858,1899^ These phage proteins tend to lose efficiency in distantly related species,^1900^ so homologous proteins have been identified from phages that infect diverse bacterial species. Recombination proteins have been found for the soil bacteria *Bacillus*,^1900^ *Shewanella*,^1870^ *Pseudomonas*,^1901–1903^ *Xenorhabdus*,^1904^ and *Photorhabdus*.^1904^ One downside with recombineering is that its efficiency is low. Also, off-target recombination can occur, necessitating clean-up steps using phage transduction to transfer a genetic insertion from the modified strain into a clean genomic background.

CRISPR tools can increase the efficiency and specificity of prokaryotic genome editing. CRISPR/Cas9 (or other nuclease proteins) generates a double-stranded DNA break at a specific target site that is then repaired by HR.^1905–1907^ When the repair template has the DNA payload, it will be inserted into the site. The location can be targeted through gRNA design and off-target effects tend to not be a problem in bacteria.^1908^ This strategy improves editing efficiency over recombineering alone because double strand breaks increase DNA repair and recombination activity at the desired site. Furthermore, as unrepaired double-stranded breaks are lethal in most bacteria, CRISPR creates a negative selection pressure against the genomes that have not been repaired with the payload DNA.^1905^ CRISPR can be combined with high efficiency recombination machinery, such as the λ red system (CRISPR-λ Red Recombineering), to perform seamless one-step genome editing without the need for selection markers.^1909^ First shown in *E. coli* and *Streptococcus* *pneumoniae*,^1905^ versions of this system have since been used for *Bacilli* ^1905,1910,1911^ *Azorhizobium*,^1912^ *Streptomyces*,^1913,1914^ *Xanthomonas*,^1915^ *Ralstonia*,^1916^ and *Pseudomonads*.^1917^

Limitations of CRISPR-mediated genome editing include low recombination frequencies and the need for host-specific recombination factors.^1918^ CRISPR-guided transposases (CASTs) overcome these obstacles, attaining the high integration efficiencies of integrases and transposases while maintaining the programmability of gRNA. CASTs have transposases that bind to CRISPR-Cas9 proteins, which guide them to the target encoded on the gRNA.^141,1919,1920^ Optimized CASTs can efficiently insert 10 kb+ DNA payloads at a targeted locations.^1921^ Expressing transposases that target orthogonal insertion sequences permits multiplexed DNA insertions in a single step. CASTs have been used to modify soil bacteria, including *Klebsiella* *oxytoca*,^1921^ *Pseudomonas* *putida*,^1921^ *Klebsiella* *michiganesis*,^1922^ and *Pseudomonas* *simiae* (Figure 28A).^1922^

**Figure 28:**
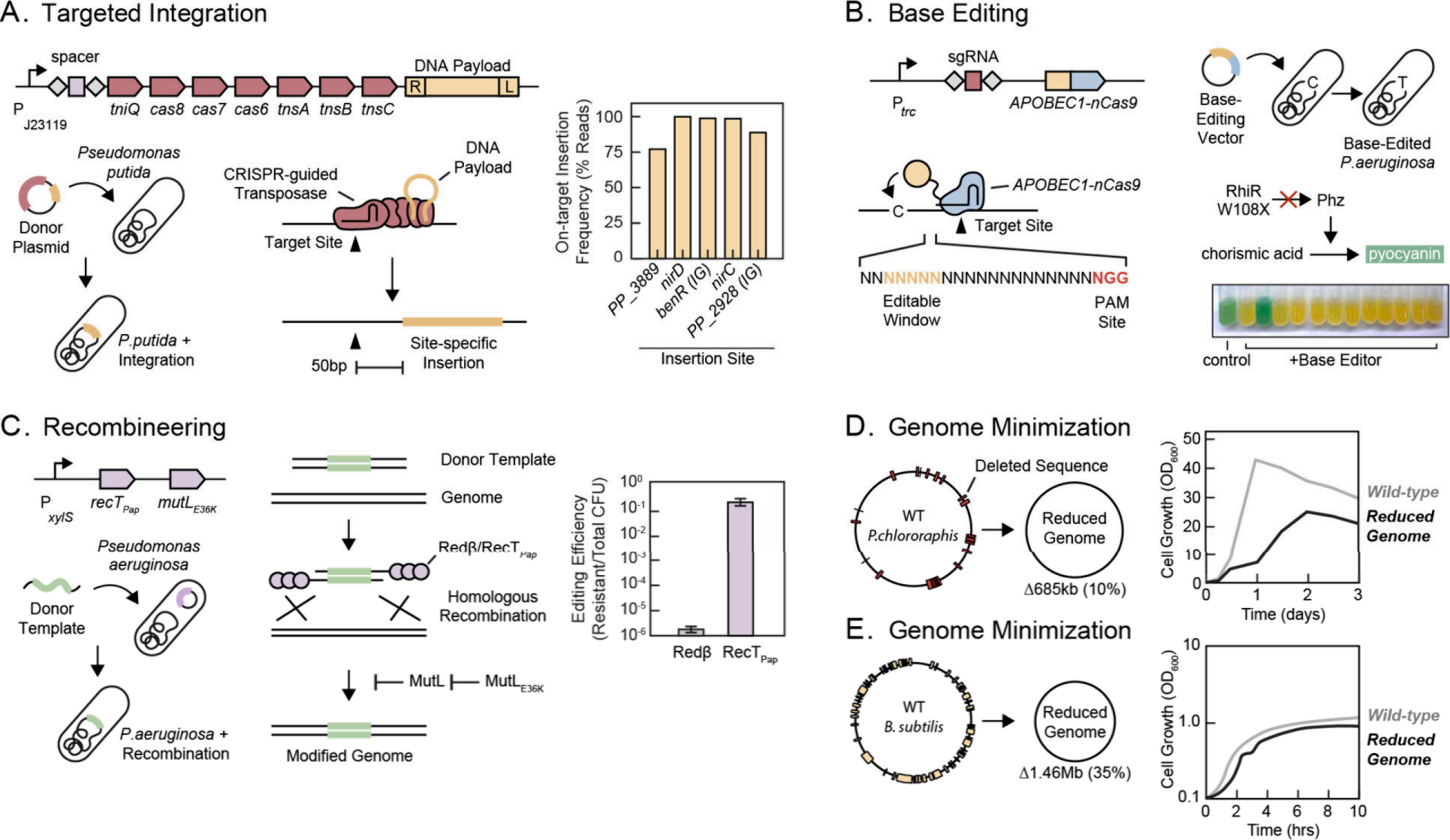
**Genome modification in soil bacteria**. **A**. Marker-free Cas9-guided site-specific transposition in *Pseudomonas* *putida*.^142^ A Type I-F CRISPR-associated transposon system from *Vibrio* *cholerae* was delivered on a single donor plasmid comprising the transposition proteins TnsA, TnsB and TnsC as well as RNA-guided DNA targeting complex TniQ-Cascade (Cas proteins). A gRNA on the plasmid directs delivery of the DNA cargo flanked by left (L) and right (R) transposon ends; DNA cargo is inserted approximately 50bp downstream of the gRNA target site. Deep sequencing was used to quantify on-target insertion frequency in *Pseudomonas* *putida* at various genomic loci, including intergenic regions (IG). **B**. CRISPR-guided base editing in *Pseudomonas* *aeruginosa* PAO1.^1923^ Site-specific base editing is performed by a fusion protein containing the cytidine deaminase APOBEC1 and the Cas9 nickase (nCas9). The APOBEC1 editor mediates the conversion of C->T in a small editable window (yellow nucleotides) upstream of the targeted PAM site (red nucleotides). The CRISPR-guided base editor was used to inactivate the transcription factor RhiR, which induces the production of the green pigment pyocyanin. Successfully edited colonies do not display the characteristic green color of *Pseudomonas* *aeruginosa* PAO1. Image reproduced with permission from ref 1923. Available under a CC BY license. Copyright 2018, iScience. **C**. Recombineering in *Pseudomonas* *aeruginosa* PAO1.^1901^ Recombineering-based methods rely on ssDNA-annealing protein (most commonly Redβ from *Escherichia* phage λ) to anneal donor ssDNA to genomic DNA at the replication fork. Redβ has reduced activity in strains distantly related to *Escherichia coli*. The ssDNA-annealing protein RecT_pap_ was identified through the genetic screen and facilitated high rates of recombination in *Pseudomonas aeruginosa* PAO1 when coexpressed with a dominant-negative mutant MutL, which is responsible for disabling mismatch repair. **D**. Genome reduction in *Pseudomonas* *chlororaphis*.^1924^ A total of 22 genomic regions (red boxes) were deleted in the *Pseudomonas* *chlororaphis* through single crossover homologous recombination. The growth rates of the wild-type and genome reduced strains were measured in rich media. **E**. Genome reduction in *Bacillus* *subtilis* 168.^291^ Through iterative deletion of 94 regions (yellow boxes), 1.46 Mb was deleted through cycles of homologous recombination and counter-selection. Growth curves were measured in LB medium.

##### 3.2.3.3 Base Editing the Genome

Targeting domains used for genome editing have been repurposed to bring other modifying enzymes to specific positions. So-called “base editors” combine a DNA-binding domain (*e.g.*, dCas9:gRNA) with a DNA-modifying enzyme to make single mutations to the genome (Figure 28B).^81,1925–1927^ Their use in plants is described in Section 2.2.1. In prokaryotic genomes, deaminase domains fused to dCas9 introduce single C-to-T or A-to-G edits. This editing has been applied to many soil bacteria, including *Streptomyces, Pseudomonas*, and *Klebsiella*.^1923,1928,1929^

##### 3.2.3.4 Genome-Scale Engineering

Multiplex automated genome engineering (MAGE) performs rapid genome engineering through repeated robot-assisted rounds oligonucleotide transformation that recombine with the genome during replication.^1858^ Edits can be multiplexed by pooling oligonucleotides to simultaneously target many genome locations.^1930^ MAGE is limited by the length of the genome that can be edited by a single oligo (<1 kb). Thus, many iterative steps are required to cover the genome.

Transferring MAGE to bacteria beyond *Escherichia* *coli* has been challenging because the phage recombinase has low activity outside of enteric bacteria and high-efficiency recombination requires inactivating native mismatch repair systems.^1931,1932^ Alternative phage recombinases with broader host-range were identified using bioinformatics.^1903^ A selection was performed on single-stranded DNA-annealing proteins to improve ssDNA-based recombination across diverse bacteria, including *Klebsiella* *pneumoniae* and *Pseudomonas* *aeruginosa* (Figure 28C).^1901^ To move MAGE into *Pseudomonas* *putida*, a ssDNA-annealing protein (Rec2) and an inactive mutant of the native mismatch repair protein MutL were transiently overexpressed during recombineering.^1932^ The mutant MutL inhibited the reversion of MAGE-derived edits by disrupting efficient mismatch repair. Cas9 was used to select for recombination events to replace the TAG stop codons in *Pseudomonas* *putdia*.^1933^

An example of the application of MAGE was to convert 314 occurrences of the UAG stop codon in *Escherichia* *coli* to synonymous UAA codons, thereby freeing up UAG for other uses, such as encoding non-natural amino acids.^1934^ Such amino acids introduce a range of new functions to proteins in living cells, including fluorescence,^1935^ reactive functional groups,^1936,1937^ diverse amino acid structures,^1938,1939^ and entire synthetic peptides.^1940^ To deliver the new amino acid, the corresponding aminoacyl-tRNA synthetase and tRNA (UAG) pair, orthogonal to the host’s translation machinery. This approach has enabled the incorporation of more than 200 non-canonical amino acids into proteins.^1941,1942^ Non-canonical amino acids have introduced into the soil bacteria *Pseudomonas* *aeruginosa*, *Streptomyces* *venezuelae*, and *Bacillus* *subtilis*.^1943–1946^ Notably, non-canonical amino acids allow “click chemistry,” which uses chemical functional groups that can perform highly efficient and selective reactions, to be performed in living *Pseudomonas* *putida*.^1947^

Conjugative assembly genome engineering (CAGE) performs large-scale genome recombination between two related strains.^1948^ CAGE employs an origin of transfer (oriT) inserted onto the genome of an engineered donor cell that directs the transfer of the engineered genome to a recipient bacterium. Following conjugation, the engineered and recipient genomes recombine. It can iteratively replace large fragments of DNA (>100kb) until a genome is completely replaced.^284,1934^ CAGE has only been performed in *Escherichia* *coli*. Similarly large-scale replacements can be made by assembling *de novo* synthesized DNA fragments in yeast that are then used to iteratively replace the *Escherichia* *coli* genome using CRISPR-λ Red recombineering.^298,1949,1950^ Using this approach, all 18,214 instances of the UAG stop codon and the UCG and UCA serine codons were removed.^298,1949^

The complete *de novo* synthesis of a genome and its transformation into a cell lacking a genome opens the possibility for even more radical genome-scale engineering.^1951^ The potential for its application to plant chromosomes and plastid/mitochondrial genomes are described in Section 2.1.5. Entire viral and prokaryotic genomes have been constructed from scratch using DNA synthesis. For example, a 1 Mb synthetic genome was constructed and placed into a cell where the native genome was removed to “boot up” *Mycoplasma* *mycoides* JCV-syn1.0 in a single step.^232^ During assembly, the synthetic genome was divided into multi-kb fragments that were either systemically replaced into a prokaryotic genome or assembled in one step in yeast before the complete genome was transferred into an empty cell.^232,277,283,1952^ The ability to synthesize genomes simplifies the ability to remove unnecessary or harmful regions and massively reorganize it to simplify future engineering (*e.g.*, grouping functionally-related genes in a way that would make sense to a human designer).^283^

Using the natural competency and homologous recombination abilities of *B. subtilis*, the 3.5 Mb genome of *Synechocystis* PCC6803 was inserted onto the *Bacillus* genome in four approximately 900 kbp fragments through reiterative rounds of short genome integrations.^1953^ This method was then used to construct the complete 6.3 kb mitochondrial genome from mice and the 134.5 kb chloroplast genome from rice.^1954^

#### 3.2.4 Minimal Genomes

Genome reduction aims to enhance engineerability by reducing complexity.^1955^ Under field conditions, it has been observed that bacteria with smaller genomes grow more rapidly.^1956^ The elimination of superfluous genetic elements could increase the capacity to make recombinant proteins or produce new chemical products. However, removing genomic DNA could come at the cost of defense mechanisms, metabolic flexibility, and stress responses that benefit in rare, but critical conditions.

The creation of a “minimal” genome seeks to only retain the essential genes necessary for the survival and growth of the organism. In *Escherichia coli*, about 300 out of 4000 genes were estimated to be essential, although estimating the true set of minimal genes difficult.^1957^ For one, genes can have redundant functions. Also, whether a gene is essential depends on which molecules are provided in the growth media.

One approach for genome reduction involves iteratively deleting regions of the genome in a “top-down” approach. Early attempts at creating a reduced *Escherichia coli* genome removed mobile genetic elements.^282^ The reduced genome had improved transformation efficiency and genetic stability.^282^ This approach was taken further, where 40% was removed by deleting 33 genomic regions encoding only non-essential genes.^1958^

Reducing the genomes of soil bacteria could improve their performance in the field. For example, a reduced genome could be designed to remove mechanisms for the disruption of recombinant systems (prophage, transposons, etc.) or no longer produce natural products that could negatively influence the microbial composition of the rhizome (*e.g.*, antimicrobials). Toward this goal, the genome was reduced for the soil bacterium *Pseudomonas putida*, albeit to improve its use in bio-manufacturing. Mobile genetic elements, prophages, and flagellar genes were deleted to improve genetic stability and reduce background energy consumption, respectively.^1959^ The genome-reduced strains outcompeted the parental strain in every industrially-relevant phenotype assessed and displayed a 40% increase in recombinant protein expression.^1960^ Deleting large genetic islands containing nonessential genes in the polyhydroxyalkanoate (PHA) producer *Pseudomonas* *mendocina* led to an 8% reduction in genome size and a 2-fold increase in PHA yield.^1961^ To simplify secondary metabolite engineering in the plant growth-promoting rhizobacterium *Pseudomonas* *chlororaphis*, gene islands not conserved amongst *Pseudomonads*, including five biosynthetic gene clusters (BCGs), were deleted to reduce the genome size by 10% (Figure 28D).^1924^ A minimal version of the *B. subtilis*, referred to as “miniBacillus,” was constructed by removing 35% of its genome (Figure 28E).^291,1962,1963^ Despite the removal of so much genetic material, its growth rate was the same as wild-type and it even had increased translation and protein secretion rates.

*De novo* DNA synthesis has been applied to build and replace entire microbial genomes. Smaller genomes can be custom-designed and synthesized to improve performance and stability in a “bottom-up” approach.^1964^ The smallest reduced genome is 531 kb genome of *Mycoplasma* *mycoides* (JCV-syn3.0) with 473 genes.^283^ The growth rates of genome-reduced strains are often much slower than wild-type strains but can sometimes be recovered through adaptive laboratory evolution (Section 3.2.2).^1965,1966^

#### 3.2.5 Genomic Landing Pads

Landing pads are dedicated sites for the insertion of DNA payloads that have been placed into a host’s genome.^145,1967,1968^ Their role is to increase the integration efficiency and to ensure that a recombinant genetic system performs reliably in the context of the host genome. They also reduce the impact on native gene expression. Landing pads have become important for eukaryote engineering, particularly mammalian cells, and have been implemented in bacteria.^145,1967^ They have begun to be considered in the plant genome (Section 2.1.2).

The first step in building a landing pad is to identify an appropriate position in the genome. The problem is that prokaryotic genomes are efficiently encoded and there are few sites that are free to carry additional genes without disruption. Also, for engineering projects, high recombinant gene expression is generally needed. For example, it might be to express enzymes at the highest level to produce maximum products of a growth hormone. Different regions of the genome can lead to different expression levels due to chromosomal structure and local transcriptional processes.^1969^

Methods have been developed to systematically scan the genome for regions of higher expression.^150^ These sites tend to occur close to the origin of replication because during cell growth and division, the origin is replicated first leading to higher effective copy number.^1970^ The copy number around the origin can be as high as 8, whereas at the chromosomal terminus it approaches 1. The higher copy number comes during rapid growth, during which multiple replication bubbles form in the genome during replication. Expression can vary by up to 200-fold across the genome, an effect that has been observed in *Escherichia coli*, *Bacillus subtilis*, and *Acinetobacter baylyi*.^150,1971–1974^ If higher levels of expression are required, then the same construct can be inserted into multiple landing pads.^1975^

Sites must also avoid disrupting native pathways. Disruption can occur because the site occurs within a gene, but also can affect gene expression indirectly by altering regulation or chromosomal structure. The effect may not be obvious under laboratory conditions, but could impact fitness under stressful or nutrient limitations, as would be expected in the field. For example, in *Escherichia coli*, a landing pad that disrupts the production of a vitamin was not discovered until growth media was used where it is absent and this would have affected its use as a therapeutic bacterium in the human body.^145^ The affect can be crudely measured as a change in growth rate, but RNA-seq provides a clearer picture of the disruption of native transcription.^145^ Computational methods have been developed to identify non-disruptive positions for a landing pad by using ML to assess RNA-seq data taken under a range of conditions to find transcriptionally silent sites.^1976,1977^

Landing pads comprise two features. First, a landing pad may have a sequence to facilitate the insertion of a DNA payload to this site. This sequence could correspond to an integrase or recombinase or a sequence associated with a specific gRNA designed to deliver Cas9 to the target.^1918^ In theory, a gRNA does not need a target site as it can be programmed to target many sequences. However, calculating an ideal sequence that takes the genome into account can improve efficiency and reduce off-target effects.

Second, the landing pad should be insulated from the genome. In prokaryotes, transcription is the dominant mode of interference. Pervasive transcription from the genome could interfere with the recombinant system and vice versa. One way to mitigate this problem is to flank the landing pad with strong terminators that block transcription in both directions.^145,735,736^ A *Pseudomonas putida* landing pad that included two terminators flanking the gene-of-interest was used to screen a library of synthetic promoters.^1978^ Chromosomal structure, interactions with nucleoid associated proteins, supercoiling, and protein occupancy can also affect transcription depending on the genomic location.^150^ Additional insulating elements may be beneficial to include where these mechanisms are prevalent.

CRAGE (chassis-independent recombinase-assisted genome engineering) is a method for landing pad construction that has been used in over 40 species, including many from soil.^1884,1979,1980^ In the first step of CRAGE, a transposase randomly inserts a landing pad containing recombinase recognition sequences. A screen can be performed after the first step to select for insertions with the desired activity, such as high expression.^1980^ Other functions can also be screened; for example, when CRAGE was used to engineer the plant growth promoting bacterium *Pseudomonas simiae* WCS417r using a screen to select for a strain with unimpaired plant colonizing ability.^1981^ CRAGE has also been used to screen gene clusters from symbionts of insect-pathogenic nematodes in various chassis for the potential development of novel bioinsectides.^1884^

#### 3.2.6 Genetic Part Libraries for Soil Bacteria

Advanced genetic engineering hinges on the availability of precision genetic parts.^299,301,310,1447,1982^ Parts are needed to control expression, such as RBSs or promoters, but the concept encompasses any unit of DNA with an assigned function. Characterized part libraries have been used to balance enzyme levels to optimize a biosynthetic pathway or connect regulators to build a genetic circuit. To perform this role, there need to be many parts of varied function, such as a library of promoters of different strengths. In bacteria, homologous recombination can disrupt a synthetic system, so part reuse in a design requires a functionally redundant set with sufficiently divergent DNA sequences.^886,1447,1983^ In Synthetic Biology, there have been major efforts, including by the “BioFAB” and “BioFoundries”, to use robotic automation and high-throughput assays to characterize large sets of genetic parts, as well as to define new ones, to facilitate genetic design.^1984–1987^

The ideal genetic parts will function the same irrespective of where they are encoded in the genome.^301,358^ Very large part libraries, sometimes consisting of thousands of characterized members, have been constructed for model species and these have been used to populate on-line databases.^301,309,735,736,1982^ Here, we focus on part libraries, often relatively small, that have been developed for soil bacteria. These libraries are based on simple non-coding parts to control expression. Table 2 shows part libraries that we have gleaned from the literature for soil bacteria.

**Table 2:** Genetic Part Libraries in Soil Bacteria

Species	Genera	Library Type	# Parts	Ref
*Acinetobacter baylyi*	γ-proteobacteria	promoters	12	^2025^
*Acinetobacter baylyi*	γ-proteobacteria	promoters	17	^1973^
*Acinetobacter baylyi*	γ-proteobacteria	RBS	5	^1973^
*Agrobacterium fabrum*	α-proteobacteria	promoters	12	^2025^
*Agrobacterium tumefaciens*	α-proteobacteria	promoters	10	^2026^
*Aliivibrio fischeri*	γ-proteobacteria	promoters	12	^2025^
*Azorhizobium caulinodans*	α-proteobacteria	promoters	4	^2027^
*Azotobacter vinelandii*	γ-proteobacteria	Promoters	22	^2028^
*Azospirillum brasilense*	α-proteobacteria	Promoters	8	^2029^
*Bacillus amyloliquefaciens*	Bacilli	promoters	8	^2030^
*Bacillus subtilis*	Bacilli	promoters	26	^2031^
*Bacillus subtilis*	Bacilli	promoters	214	^2032^
*Bacillus subtilis*	Bacilli	promoters	84	^2033^
*Bacillus subtilis*	Bacilli	promoters	41	^2007^
*Bacillus subtilis*	Bacilli	promoters	4	^2035^
*Bacillus subtilis*	Bacilli	promoters	114	^2036^
*Bacillus subtilis*	Bacilli	promoters	8	^2037^
*Bacillus subtilis*	Bacilli	promoters	6	^2038^
*Bacillus subtilis*	Bacilli	promoters	4	^2039^
*Bacillus subtilis*	Bacilli	RBS	5	^2035^
*Bacillus subtilis*	Bacilli	RBS	38	^2007^
*Bacillus subtilis*	Bacilli	protein degradation tags	11	^2007^
*Bacillus thuringiensis*	Bacilli	promoters	20	^2040^
*Burkholderia thailandensis*	β-proteobacteria	promoters	12	^2025^
*Comamonas testosteroni*	β-proteobacteria	promoters	3	^2041^
*Cupriavidus necator*	β-proteobacteria	promoters	8	^2042^
*Cupriavidus necator*	β-proteobacteria	promoters	4	^2043^
*Cupriavidus necator*	β-proteobacteria	RBS	3	^2043^
*Herbaspirillum seropedicae*	β-proteobacteria	promoters	7	^2029^
*Klebsiella michiganensis*	γ-proteobacteria	promoters	8	^2029^
*Klebsiella pneumoniae*	γ-proteobacteria	promoters	3	^2044^
*Klebsiella variicola*	γ-proteobacteria	promoters	23	^2028^
*Pseudomonas aeruginosa*	γ-proteobacteria	promoter	12	^2025^
*Pseudomonas putida*	γ-proteobacteria	promoter	12	^2025^
*Pseudomonas putida*	γ-proteobacteria	promoters	38	^2045^
*Pseudomonas putida*	γ-proteobacteria	RBS	11	^2045^
*Pseudomonas putida*	γ-proteobacteria	promoters	9	^2046^
*Pseudomonas putida*	γ-proteobacteria	promoters	7	^2047^
*Pseudomonas putida*	γ-proteobacteria	promoters	4	^2048^
*Pseudomonas putida*	γ-proteobacteria	promoters	5	^2049^
*Pseudomonas putida*	γ-proteobacteria	promoters	8	^2050^
*Pseudomonas protegens*	γ-proteobacteria	promoters	34	^2027^
*Pseudomonas protegens*	γ-proteobacteria	RBS	31	^2027^
*Pseudomonas protegens*	γ-proteobacteria	terminators	10	^2027^
*Pseudomonas stuzeri*	γ-proteobacteria	promoters	22	^2028^
*Rhizobium leguminosarum*	α-proteobacteria	promoters	7	^2029^
*Rhizobium* sp. IRBG74	α-proteobacteria	promoters	33	^2027^
*Rhizobium* sp. IRBG74	α-proteobacteria	RBS	31	^2027^
*Rhizobium* sp. IRBG74	α-proteobacteria	terminators	29	^2027^
*Ruegeria* sp. TM1040	α-proteobacteria	promoters	12	^2025^
*Saccharothrix espanaensis*	Actinomycetes	promoters	5	^2051^
*Salinispora tropica*	Actinomycetes	promoters	5	^2051^
*Shewanella oneidensis*	γ-proteobacteria	promoters	9	^2052^
*Sinorhizobium meliloti*	α-proteobacteria	promoters	10	^2026^
*Streptomyces albus*	Actinomycetes	promoters	32	^2053^
*Streptomyces albus*	Actinomycetes	promoters	65	^2051^
*Streptomyces albus*	Actinomycetes	promoter/RBS	55	^2054^
*Streptomyces albus*	Actinomycetes	promoters	5	^2051^
*Streptomyces avermitilis*	Actinomycetes	promoters	7	^2021^
*Streptomyces coelicolor*	Actinomycetes	5′-UTR	4	^2055^
*Streptomyces lividans*	Actinomycetes	promoters	38	^2056^
*Streptomyces lividans*	Actinomycetes	RBS	70	^2057^
*Streptomyces lividans*	Actinomycetes	terminators	6	^2057^
*Streptomyces lividans*	Actinomycetes	promoters	5	^2051^
*Streptomyces venezuelae*	Actinomycetes	promoters	15	^2058^
*Streptomyces venezuelae*	Actinomycetes	promoters	59	^2021^
*Streptomyces venezuelae*	Actinomycetes	RBS	56	^2021^
*Sulfitobacter* sp. EE-36	α-proteobacteria	promoters	12	^2025^
*Xanthomonas campestris*	γ-proteobacteria	promoters	12	^2025^

##### 3.2.6.1 Promoter Libraries

Constitutive promoters are always on, producing a constant transcription rate. The rate can be quantified as the flux of RNAP on the DNA (RNAP/s).^333,728,730,1988^ Ideally, this rate does not change in different growth phases or environmental conditions. The most common method to report the strength of a promoter is to use a reporter, such as a fluorescent protein, but this also encompasses translation.^333^ More direct measurements of RNAP flux can be obtained using RNA-seq or even direct single-molecule imaging of RNAP progression on DNA strands.^145,1989–1992^ Promoter activity can be compared to a reference standard to report as “relative promoter units” (RPU) and single molecule experiments allow this to be converted to absolute units of RNAP/s (for plants, see Section 2.2.1.1.7).^333,730^ Note that often the lengths of promoter sequences provided in a part library are too short (35-50 bp) and an upstream region needs to be added to avoid interference from the upstream sequence (up to 150bp).^1993,1994^

Promoter libraries also have been built for RNAPs that are orthogonal to native transcriptional processes. Promoters that are turned on by phage RNAP are not transcribed by native RNAPs.^1995^ Libraries containing promoters of different strengths have been built for RNAP from T7 and related phage (Table 2).^919,1996–2002^ A characteristic of these promoters is their small size; the T7 and SP6 promoters are only 18 bp. Phage RNAPs tend to function similarly across species and the rank order of their strengths usually does not change.^2000,2003,2004^ They have been shown to be functional in various soil bacteria.^1995^ However, phage RNAPs and their promoters can exhibit toxicity when used together in a design (either individually is not), possibly due to its fast processivity and decoupling from the ribosome.^919^ The impact on growth rate has been anecdotally observed to be worse for wild strains.

##### 3.2.6.2 Ribosome Binding Sites

The first step of translation is the binding of the ribosome to an mRNA. For many species, the affinity of the ribosome to the RBS is a primary determinant of the translation rate and ultimately the level of gene expression.^311,1447^ Therefore, the RBS has played a key role to modulate expression levels. There are two approaches for creating synthetic RBSs. The first is to characterize the strengths of a large library created using random mutagenesis.^309,1990,2005–2008^ RBS libraries comprising thousands of members have been characterized, including for some soil species (Table 2).

The second approach is to calculate an RBS sequence *de novo* for each gene to be controlled using the RBS Calculator or related tools.^311,2009–2011^ These calculations assume that the only sequence determinant of strength is the ribosome binding to mRNA and there are no other biochemical factors that contribute, which is not always true.^2012,2013^ The calculators predict the strength based on the unfolding of the local mRNA structure, the free energy of binding of the ribosome to the SD and start (ATG) sites, and other thermodynamic factors. In theory, this can be applied to any species by using the 16S rRNA sequence. The RBS Calculator has been used to design sequences for soil bacteria, including *Bacillus* and *Pseudomona*s, and plastids (Section 2.4.1).^2014–2017^

RBSs can be dependent on the local sequence context of the mRNA, especially the first 10 bp of the open reading frame.^2018^ Insulators have been developed to mitigate these effects.^301,302,808,2019^ The insulator RiboJ contains a ribozyme that cleaves the upstream region of the mRNA after transcription, thereby reducing context effects.^302^ It also stabilizes the mRNA by creating a hairpin and removing the hydroxyl at the 5′-end, thus protecting against degradation, and improving access to the RBS.^2019,2020^ In an analysis of 63 promoter:RBS combinations with the ribozyme RiboJ in *Streptomyces venezuelae*, inclusion of the ribozyme RiboJ reduced unexpected changes in gene expression due to interference between the promoter and RBS.^2021^ RiboJ has also been used in the soil bacterium *Pseudomonas taiwanensis*.^2022^ Many variants of RiboJ are available that are sufficiently sequence diverse so that they can be used in a design without leading to homologous recombination.^808^

Another means to reduce the context dependence of an RBS is to use a bicistronic design.^301^ It places an RBS and leader peptide upstream of the RBS that is used to control the protein-of-interest. The first RBS and peptide eliminate secondary structures, making changes to the downstream RBS more reliable. Bicistronic RBS’s have been used in the soil bacteria *Acinetobacter baylyi* and *Pseudomonas taiwanensis*.^1973,2022^

##### 3.2.6.3 Terminators

Intrinsic terminators stop transcription by not allowing RNAP to pass. In prokaryotes, they consist of a hairpin, followed by a poly-T region. Stronger terminators block a larger fraction of RNAPs. Mutations that weaken the hairpin or disrupt the poly-A region led to a less effective terminator. However, creating a mathematical model of terminator strength remains a challenge, pointing to unknown contributing factors.^735,736,2023^ The inability to design terminators computationally has limited the development of terminator libraries to screening large libraries of natural or designed terminator sequences.^2024^ Terminator strength can be measured by placing it between two reporter genes and quantifying the drop in expression between the two.^735,736^ Alternatively, the terminator strength can be quantified as the drop in transcription across a region measured by RNA-seq.^1438,1990^

Placing two terminators in series (a “double terminator”) can lead to the stronger blocking of transcription.^145^ Note that terminator strengths do not always combine additively and the spacer between the two can affect strength, so simply placing terminators in series is not a guarantee of improved knockdown. In fact, if not done correctly, it can negate the effect of both terminators. Terminators also leave a 3′-UTR on the mRNA, the sequence of which can affect transcript stability. Terminators can either block transcription in one direction or in both (bi-directional).

In general, terminator strengths are conserved across prokaryotes. In other words, an intrinsic terminator in *E. coli* is likely to function as such across species. However, care needs to be taken when using any part library that has not been characterized in a specific species. For example, some *E. coli* terminators were shown to function as promoters in soil Rhizobia; completely the opposite function.^1796^ Some terminator libraries have been characterized in soil bacteria (Table 2).

#### 3.2.7 Directed Evolution

Directed evolution has been applied to improve many enzymes and pathways of interest to agriculture (for plants, see Section 2.2.2.5). Iterative rounds of the creation of mutant libraries and the screening of them for function have been shown to improve enzymes, pathways, as well as entire strains. These enzymes could be used as part of biomanufacturing, such as the synthesis of an insecticide in a fermenter, or it could be put into a strain that is then applied in the field.

Evolving enzyme variants with improved catalytic rates, altered substrate specificity, or stability can be useful for the bioproduction of agrochemicals. The biosynthesis of the insecticide limonene in yeast was improved through by targeting mutagenesis to specific residues in a rate-limited enzyme from the terpene pathway.^2059,2060^ Insect pheromones can be released to disrupt mating, but their widespread use was limited by the difficulty of synthesizing these molecules. A key step in pheromone biosynthesis was improved by applying directed evolution to a cytochrome P450 from *Bacillus megaterium* to produce cyclopropane precursors.^2061^ This advance has allowed the pheromones to be commercialized for field use.^2062,2063^

Pesticides and their production hosts have been improved using directed evolution. Derivatives of the antibiotic andrimid were produced by evolving a chimeric NRPS-PKS (non-ribosomal peptide synthase – polyketide synthase) from two soil bacteria (*Pantoea agglomerans* and *Bacillus licheniformis*) that initially had low activity.^2064^

Many plant-growth promoting bacteria (PGPB) release beneficial antimicrobial or antifungal peptides. For example, the soil bacteria *Bacillus subtilis* and *Bacillus amxyloliquefaciens* have been shown to be effective biocontrol agents for the pathogenic *Macrophomina phaseolina* soybean fungus through the production of lipopeptides.^1517,1518^ The soil bacteria *Bacillus subtilis* and *Bacillus amyloliquefaciens* produce lipopeptides that kill *Macrophomina phaseolina* soybean fungus.^2065^ However, overproduction of an antimicrobial peptide can be difficult due to toxicity in its microbial host. To solve this problem, *B. subtilis* was evolved to be tolerant to the lantipeptide nisin by using dCas9 to target mutations to a transporter gene.^2066^

### 3.3 Pathway Engineering

Bacteria contain pathways that can produce many chemicals of agricultural importance. Structurally complex natural products can function as insecticides, antimicrobials, phytohormones, or play roles in colonization or survival. These chemicals may underly the functional effects of a bioinoculant.^2067–2072^ The native species that makes a chemical may not be suitable for a particular application, crop, or may be fragile in the environment. The pathway may also not be expressed (“silent”) under the conditions where it is needed for an application. This section focuses on the engineering of complex multigene pathways, either in their native host or in transferring them to a host-of-interest for an application.^2073^

#### 3.3.1 Biosynthetic Gene Clusters

In bacteria, genes encoding natural product pathways are typically clustered together on the genome. These clusters vary in size, often from 10-20 kilobases, but can be as large as 100 kilobases or more. They also often involve dozens of genes, multiple operons, and complex regulatory mechanisms. The most commonly-studied natural product pathways are those that make chemical natural products, such as polyketides, which have been used as medicines. However, there are many other pathways that are organized as BGCs, including metal nanoparticles, CRISPR immunity, and molecular machines involved in locomotion and pathogenesis.^2074^ Gene clusters are not a unique feature of bacteria; while less common, functional genes can also be clustered in fungi and plants and there are computational tools for their discovery as well.^2075–2078^

BGCs encode pathways for many agriculturally-relevant chemical products. Spinosyns are insecticides that are neurotoxins comprised of a polyketide-derived tetracyclic macrolide with two saccharides that is synthesized by 19 genes on an 80kb cluster.^2079,2080^ Originally isolated from a sugar mill in the Virgin Islands, *Saccharopolyspora spinosa* underwent extensive genetic optimization.^2081–2086^ Globally, it is commercially available as a treatment for many insect pests. There are many other insecticides encoded in BCGs.^2087–2089^

To identify new natural products that mediate bacteria–nematode–insect interactions, 1,000 putative BGCs from 45 nematode-associated bacteria were identified using antiSMASH.^2090^ Homologous overexpression of a subset of six of these BCGs demonstrated their products serve as insect virulence factors, immunosuppressors, and protease inhibitors. Herbicides are also encoded in gene clusters, such as phosphonothrixin in *Saccarhothrix*, which has activity against weeds.^2091,2092^

BCGs also produce chemicals that protect crops against pathogenic bacteria and fungi. For example, phenazine is an antifungal that has been shown to protect wheat against diseases.^2093,2094^ It is encoded on a 7-gene biosynthetic gene cluster encoded in Pseudomonads. 2,4-Diacetylphloroglucinol (DAPG) is another antifungal from Pseudomonads with broad spectrum activity.^2095^ A study of agricultural soils across China and the United States revealed tens of thousands of such clusters.^2096,2097^ The lettuce and tomato rhizomes were found to be enriched with polyketide synthases (PKS) and non-ribosomal peptide synthase (NRPS) gene clusters as compared to soil, indicating an abundance of antimicrobial compounds.^2098^

The increased use of metagenomics to analyze soil samples has led to new computational tools and databases to catalogue BCGs. AntiSMASH is a ML-trained model that can scan genomes for BCGs in bacteria and fungi.^2099,2100^ The AntiSMASH database maintains annotated gene clusters that can be mined for activities-of-interest or compared to a newly sequenced pathway.^2101,2102^ The 2021 release had 47,517 clusters from 25,236 bacterial genomes (388 archaeal and 177 fungal). The Minimum Information about a Biosynthetic Gene cluster (MIBiG) database similarly catalogues gene clusters with a focus on high-quality chemical structures and bioactivities.^2103^ The Natural Product Atlas has over 30,000 unique chemical structures and their taxonomic sources.^2104^

#### 3.3.2 “Waking up” Silent Gene Clusters

Many genomes are rich with BCGs, particularly for bacteria that reside in the plant rhizosphere. However, most do not produce their chemical product under laboratory conditions or under conditions desired by the farmer in the field. These clusters are referred to as being “silent.” The natural regulatory control of BCGs encompasses both the global regulatory network in the bacterium as well as regulation encoded within the cluster itself. It can be difficult to figure out what combination of signals turns a desired pathway on. One approach is to extensively try different media concoctions with various nutrients or stressors in an attempt to turn on the gene cluster. However, genetic methods are required if the organism is going to be used as a bioinoculant and the chemical product needs to be produced *in situ*. Similarly, whether it is for chemical discovery or to optimize bioreactor conditions, BCGs often need to be transferred to a production host under inducible or constitutive control. BCGs also need to be transferred when the function, such as an antifungal, needs to be moved to an organism that associates strongly with a desired crop, for example forms biofilms on the roots.

##### 3.3.2.1 Inducing BCG Transcription

One strategy to overexpress the BCG is to replace the inactive or inducible natural promoters with strong constitutive promoters. This approach is commonly taken for BCG-rich Actinobacteria, including Streptomyces species. Native constitutive promoters of various strengths have been used to turn on BCGs.^2021,2051,2054,2057,2105–2107^ Sometimes a BCG can be a single operon, in which case placing and upstream promoter is relatively easy. However, it is more often the case that there are multiple operons in different orientations. Methods have been developed to perform DNA assembly in yeast, allowing for up to 16 promoters to be replaced simultaneously.^2108^ The simultaneous replacement of promoters enabled the optimized production of the antibiotic tetarimycin in *Streptomyces albus* (Figure 30A).^2108^

Inducible promoters can also be inserted into a BCG so that it is turned on by a particular signal or set of conditions.^2109–2112^ This approach can be used to place the chemical product of a BCG under the control of genetically-encoded sensors or circuits (Section 3.4). Promoters that are transcribed by T7 RNAP have also been used to turn on BCGs.^2113^ The small lengths of T7 promoters simplifies their insertion into multiple sites and the expression of T7 RNAP can turn them all on simultaneously. There are also promoters available of different strengths.

Deleting the transcriptional repressor of a BGC can lead to the activation of a silent cluster.^2114–2116^ For example, the deletion of two repressors in *Streptomyces rapamycinicus* led to an increase in the production of the antifungal rapamycin.^2117^ Overexpressing endogenous transcriptional activators by inserting additional copies downstream of strong promoters can also activate silent gene clusters.^2118–2122^ Random mutagenesis can also be used to wake up a BCG or optimize production.^2123,2124^ Finally, placing additional copies of the entire BGC onto the host genome can improve production.^2125–2127^

Variants of dCas9 can be used to turn on BCGs. The programmability of the gRNA allows it to be directed at specific single or multiple sites through the genome. It can serve as a repressor when it blocks a gene (CRISPRi) or as an activator if fused to a domain that recruits RNAP (CRISPRa). CRISPRa has been used to turn on PKS/NRPS BCGs in *Photorhabdus luminescens* by targeting sgRNAs to 300 bp upstream of the cluster’s native promoters.^2128^ To activate the expression of the polyketide jadomycin B in *Streptomyces venezuelae*, CRISPRi was targeted to the native pathway repressor.^2129^

##### 3.3.2.2 Remodeling

Engineered microbes containing sequences derived from other organisms or synthetic genetic parts risk being designated “intergeneric” by the EPA and face a difficult regulatory process before being approved for field release.^6^ Genome “remodelling” is an approach that seeks to reduce regulatory risk by not adding recombinant DNA to the genome.^2130^ Remodelling restricts genetic changes to deletion, duplication, or reshuffling of genetic sequences sourced from the native genome; for example, moving a promoter from one spot to another to turn on a BCG. The genetic changes also do not lead to the addition of selection markers, construction scars, or plasmids from the cloning process.

Remodelling requires a scarless genetic engineering method. To this end, the “two-step allelic exchange” method was developed (Figure 29).^2131,2132^ The genetic part that needs to be moved in the genome is cloned into a plasmid that cannot replicate in the host (suicide vector), flanked by homologous sequences corresponding to the desired final genomic position. The plasmid also contains selection and counter selection marker. The first step is to transform the plasmid, integrate it into new genomic site, and select for integration using a marker. The second step is to apply the counter selection to recombine the plasmid out of the genome and remove it from the cell. This approach has been used to turn on a nitrogen fixation BCG to make it constitutively express nitrogenase (Section 4.2).

**Figure 29:**
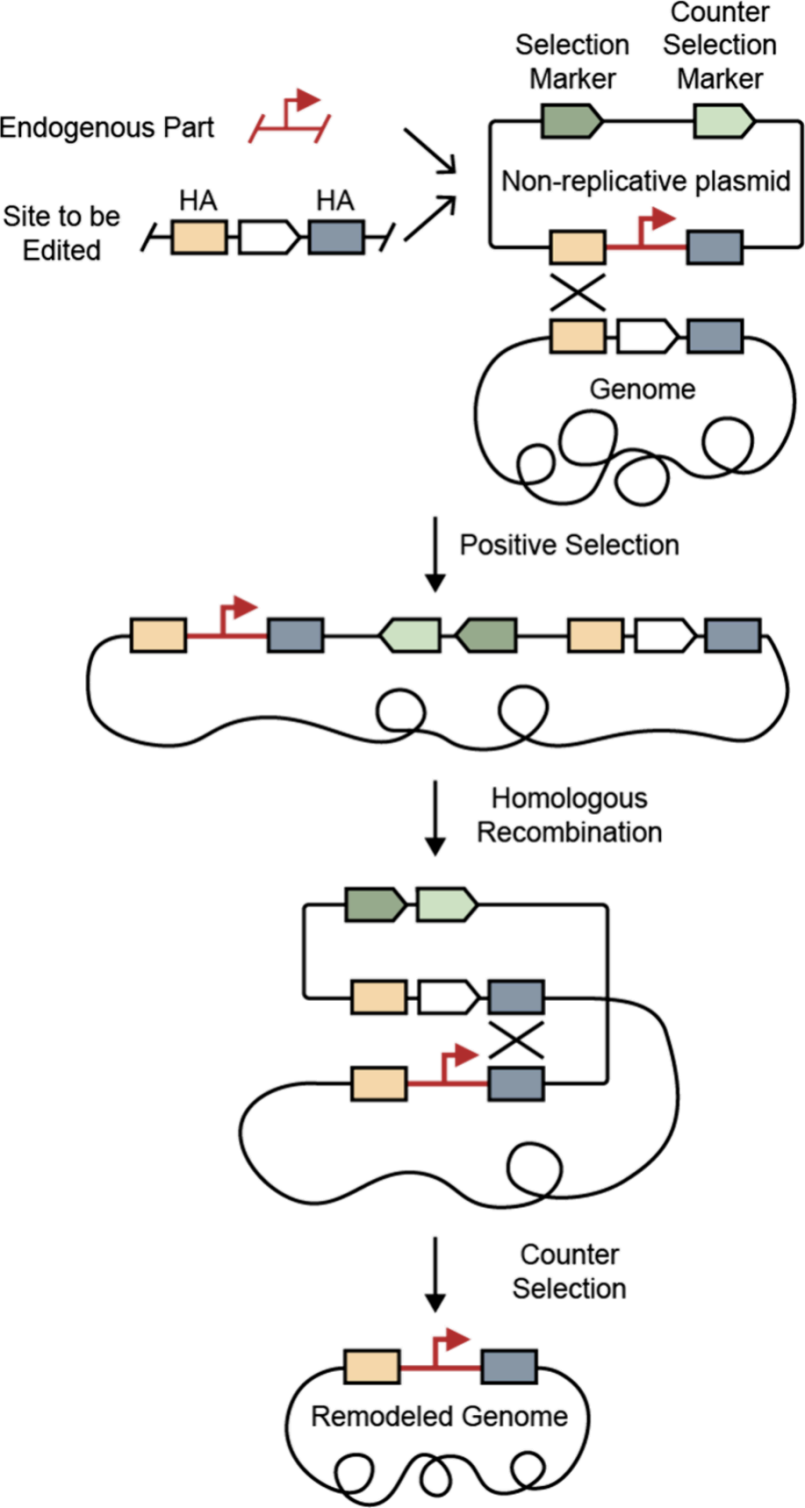
**Genetic remodeling by allelic exchange**. A plasmid incapable of replicating in a recipient microbe contains an antibiotic selection marker and a counter selection marker, as well as two sequences homologous to the genome to be remodeled (HA; homology arms). Upon introduction into the microbe, the plasmid integrates onto the host genome site-specifically through single-crossover homologous recombination and is selected for through positive selection. Following a second single-crossover homologous recombination event selected for using a counter selection marker, the native locus flanked by the homology arms is replaced by the region found on the plasmid.

Allelic exchange relies on the endogenous recombination machinery. Efficiency can be improved by using phage recombinases, which allow the genome engineering to be performed in one-step scarless genome engineering.^2133^ These recombinases can reduce the homology regions to as little as 50 bp. However, a problem with phage recombinases is that they can be very host-specific. To this end, recombinases were mined from *Burkholderia* species to activate *Burkholderia* BGCs by inserting a strong constitutive promoter (P_Apra_) from *Streptomyces clavuligerus*.^2134^ These pathways produced a total of five nonribosomal peptides from two classes of lipopeptides, glidopeptins, and rhizomides.

#### 3.3.3 Tools for Heterologous Expression

For some applications, the BGC must be transferred to a new host. Moving it to a production strain could make it easier to optimize and lead to higher product titers. The insecticide spinosyn BCG has also been reconstituted in Streptomyces, which is easier to genetically manipulate than its native producer.^2135^ It could also be to combine it with a strain that naturally colonizes a niche of interest. For example, a gene cluster producing an antibiotic against crop pathogens (caryoynencin) was moved from a Burkholderia pathogen to a non-pathogenic Paraburkholderia (does not grow at 37°C) so that it could be used as a living inoculant in the field (Figure 30B).^2136^ Production of the molecule using a living cell, versus spraying it in purified form, ensures its delivery when it is chemically unstable.

##### 3.3.3.1 Transferring Gene Clusters to New Species

Delivery of the assembled gene clusters can be carried out through recombination or assisted by transposons and integrases. To efficiently deliver BGCs to diverse proteobacteria, genetic landing pads containing Cre recombinase sites were randomly integrated onto host genomes using Tn5 or mariner transposons.^1884^ Upon introducing the BGC vector flanked by the reciprocal CR recombinase recognition sites, a co-delivered Cre recombinase facilitated integration of the gene cluster. Robust expression of the BCG across hosts was achieved by including a T7 RNAP and T7 promoter within the landing pad. One promoter assumes all the genes are oriented in the same direction. Another approach is to flank the BCG between two promoters so that the genes in both directions are transcribed.^2137^

The removal of alternative pathways from the genome can also wake up a BCG or increase the titer of its product. A genome-reduced (Section 3.2.4) *Streptomyces coelicolor*, where four endogenous NPRS and PKS pathways were removed, produced higher titers of the antibiotic congocidine.^2138^ Another strain of *Streptomyces albus* had 15 BGCs removed in order to express a cryptic cluster from the bacterium *Frankia*.^2139^ A 1.4-Mb region harboring many BGCs, but no essential genes, was removed from *Streptomyces avermitilis* to express twenty BGC from various *Streptomyces* species.^2139^

##### 3.3.3.2 Refactoring

Large gene clusters often contain layers of redundant and poorly understood regulation. Refactoring is a strategy to rebuild a genetic system from the ground up, resynthesizing the component genes under control of synthetic genetic parts to remove native regulation.^1462,2140–2144^ Refactored systems can be placed under control of a sensor or the output of a synthetic circuit (Section 3.4) and are more easily transferred to other species.^1462,1796^ The simultaneous replacement and recoding of all of the parts in large gene clusters requires the use of *de novo* DNA synthesis.

Refactoring was first applied to a 32 kb cluster that encodes a PKS that makes the human antibiotic erythromycin.^2141^ As part of reconstructing the cluster, they placed sets of genes under the control of heterologous genetic parts, including T7 promoters and synthetic RBSs. These changes facilitated its functional transfer from *Streptomyces erythraea* to *Escherichia coli*. The titer increased when the genes were codon optimized, possibly because of the elimination of regulatory sequences internal to the genes.^2145^ Phage genomes have also been refactored, with an emphasis on removing overlapping genes and to introducing restriction sites to aid cloning.^2142,2146,2147^ Since then, these principles have been applied to many BCGs to increase titers or to transfer them between hosts.^2143^

Refactoring has been applied to gene clusters of relevance to agriculture. The 80 kb biosynthetic gene cluster for the insecticide spinosyn was refactored, including the replacement of all promoters and reorganization of operons, leading to a 300-fold titer increase when transferred into a production host.^2148^ The 25 kb nitrogen fixation gene cluster (*nif*) from *Klebsiella oxytoca* was refactored by separating overlapping genes, codon optimizing them, replacing all genetic parts with synthetic variants, and placing operons under T7 RNAP control (Section 4.2).^1462,2027^ A refactored *E. coli* Embden–Meyerhof–Parnas (EMP) glycolytic pathway increased glucose utilization when transferred to the soil bacterium *Pseudomonas putida*.^2149^

Refactoring can improve biosafety by eliminating pathogenic components or moving minimal desirable machinery to a non-pathogenic host. For example, the tumor-inducing (Ti) plasmid responsible for *Agrobacterium*-mediated plant transformation was refactored to limit natural virulence (Figure 30C).^2150^ The minimal set of virulence genes required for transformation were organized into artificial operons controlled by either constitutive or IPTG-inducible promoters. The refactored Ti-plasmid remained functional even when transferred to *Rhizobium rhizogenes*, a non-pathogenic strain incapable of plant transformation.

**Figure 30:**
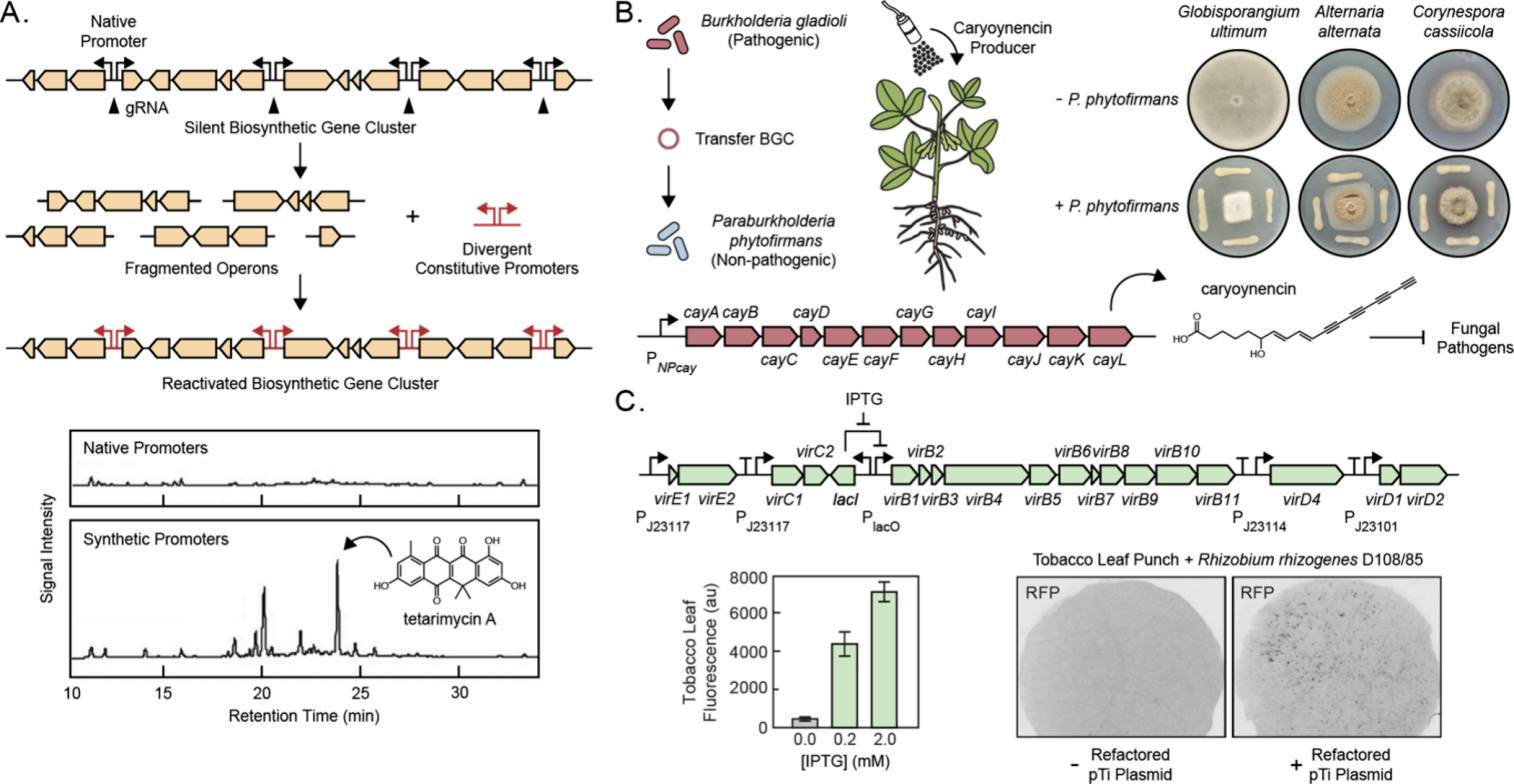
**Engineering expression of gene clusters in agriculturally-relevant microbes**. **A**. Activation of a silent BCG in *Streptomyces*.^2108^ The tetarimycin A (Tam) gene cluster is transcriptionally silent in *Streptomyces albus* under laboratory conditions but can be activated by replacing its native promoters with constitutive promoters. The Tam gene cluster was fragmented *in vitro* using Cas9 targeting the cluster’s native promoter sequences (black arrows). The fragmented sequences were reassembled to insert one of 23 previously characterized synthetic promoters active in *Streptomyces*. Tetarimycin A was extracted and confirmed through HPLC–MS analysis from 7-day-old cultures inoculated with *Streptomyces albus* harboring the engineered cluster. Data figure reproduced with permission from ref 2108. Copyright 2016, American Chemical Society. **B**. Transferring an antifungal biosynthetic gene cluster to a non-pathogenic host.^2136^ *Burkholderia gladioli* produces the polyyne antifungal caryoynencin, but its use as a biopesticide is limited because it is an opportunistic pathogen in humans. The caryoynencin gene cluster was transferred from *Burkholderia gladioli* to the non-pathogenic plant growth promoting bacteria *Paraburkholderia phytofirmans. P. phytofirmans* heterologously expressing the caryoynencin gene cluster inhibited the growth of various plant fungal pathogens including *Globisporangium ultimum* (damping-off disease), *Alternaria alternata*, and *Corynespora cassiicola* (ring spot disease) when streaked onto agar plates inoculated with the fungus (right images). Agar plate images reproduced with permission from ref 2136. Available under a CC BY license. Copyright 2022, Microbial Biotechnology. **C**. Refactoring the *Agrobacterium vir* gene cluster to implement control over plant transformation.^2150^ A refactored pTi plasmid expressed the minimal set of *vir* genes required for plant transformation. The BCG was made inducible by placing the *virB* operon, which encodes the type 4 secretion system under the control of an IPTG-inducible promoter (P_lacO_). The minimal refactored cluster was used for the transformation of tobacco leaves via the non-pathogenic strain *Rhizobium rhizogenes* D108/85, which does not naturally possess the pRi or pTi plasmids required for transformation. Images of tobacco leaf punches following transformation by *Rhizobium* rhizogenes D108/85 carrying the refactored cluster and a binary vector expressing *rfp*. Leaf punch images reproduced with permission from ref 2150. Available under a CC-BY-NC-ND 4.0 license. Copyright 2023, bioRxiv.

#### 3.3.4 Combinatorial Pathway Optimization

Pathways can be optimized by creating libraries that vary the genetic parts used to control each gene. It is easier to build a combinatorial library for a refactored gene cluster because overlapping parts (often genes) are separated and the parts modularized so that alternatives can be easily substituted.^1438^

The combination of automation with genome editing tools has massively scaled up the size of libraries that can be screened. Over 4 billion *Escherichia coli* strains per day were generated using MAGE (Section 3.2.3) to place different RBSs to control the expression of 24 enzymes in a pathway for a key isoprene intermediate (1-deoxy-D-xylulose-5-phosphate).^1858^ The isoprene pathway provides precursors to many phytohormones and plant signalling molecules.^2151^ The three-enzyme pathway to the carotenoid pigment neurosporene was optimized through a multi-dimensional search of RBS seqeuences.^1451^ Neurosporene is present in many environmental bacteria and carotenoid-rich fruits, such as tomatoes.

### 3.4 Programming Soil Bacteria to Sense-and-Respond

Many functions carried by engineered bacteria need to be timed properly to have the desired impact. For example, the production of growth-promoting hormones may have a negative effect if the plant should instead be conserving resources or activating a response to deal with a transient stress. In addition, if always turned on, the function may also impose a growth burden on the bacterium, making it less competitive in the field. Rather than always being active, these functions could be placed under the control of genetic sensors that only turn the function on when needed.

The agricultural system consists of the crop plant as well as many bacteria and fungi that reside in the rhizosphere. Synthetic sensors can program communication between these organisms. These channels could be used to stabilize artificial consortia against environmental fluctuations or establish synthetic symbiotic relationships. They could also be used to divide up functions across species. For example, the plant could “ask” an engineered bacterium to make a needed molecule in response to a stress it is facing.

Genetic circuits can integrate information from multiple sensors to identify an environment with higher specificity. For example, sensors for various metals or nitrogen sources could be integrated to respond to a particular soil type. Circuits can also respond dynamically; for instance, to produce a pulse of insecticide.

#### 3.4.1 Sensors of Soil Conditions

Genetically-encoded sensors convert a signal into a cellular response.^887,1337,2152–2155^ A stimulus, such a small molecule, serves as the input to a sensor and the output is a promoter. This promoter can either be connected to the gene that is the response or be used as an input to a genetic circuit. Many sensors have been built for bacteria, some of which respond to agriculturally-relevant signals, such as nutrients, plant metabolites, signaling molecules, agrochemicals, and heavy metals.^1796,2156–2159^ Most sensors have been designed for laboratory strains that would not survive in the field (notably, *Escherichia coli*), but sensors are increasingly being built for soil bacteria, such as *Pseudomonas, Rhizobia*, and *Bacilli*.^1796,2160,2161^ Sensors have been developed to respond to temperature, light (and color), gases such as oxygen, pH, extracellular peptides, bio-active natural products, extracellular proteins, and adhesion in a biofilm.^60,2162–2173^ Sensors can also respond to the internal state of the cell, including metabolites.^2032,2154,2174–2176^ Native promoters that respond to stress or other conditions can be viewed as sensors.^2177–2179^

Sensors that respond to a desired signal can either be constructed by finding a natural protein or RNA that binds to the ligand-of-interest^1298,1301,2180,2181^ or by engineering and evolving it to do so.^2182–2184^ Once a sensing domain is identified, it can be moved across organisms, including to eukaryotes, with relative ease so long as there are sufficient genetic parts to retool it for the new host. Databases containing ligand-responsive prokaryotic sensors and their associated protein and operator sequences are available to facilitate the transfer of these systems to new organisms.^2185^

Sensors are based on a biomolecule that responds to the signal being detected. Binding of the ligand leads to a conformational change that alters binding to DNA/RNA, thereby affecting a promoter, or changes the activity of a kinanse/phosphatase in a phosphorelay. In bacteria, the common TetR-, LysR- and LuxR- families of transcriptional regulators are single proteins that function as ligand-dependent transcriptional activators or repressors.^1298^ Two-component systems consist of a sensor protein, sometimes membrane-bound, that receives the signal and phosphorylates a second response regulator.^1337,2186^ The phosphate on the response regulator changes its conformation so that it can up- or down-regulate a promoter. Sensors can also be made from riboswitches, which are RNA sequences that bind to specific ligands. By altering the RNA conformation, gene expression is affected by changing RBS accessibility, ribozyme activity, or terminator readthrough.^1318^

A natural sensing domain can usually be found quickly that “works,” that is, changes gene expression to a detectable degree in response to the desired signal. However, the dynamic range is frequently too low or background too high to connect it to a useful response. The dynamic range can be optimized through part engineering or random mutagenesis, but it can be very difficult to decrease the background or alter the limit of detection. It is also very challenging to build a sensor that operates identically across different environmental conditions, genetic contexts, and growth phases.^2187^ Even under laboratory conditions obtaining reliable performance can be difficult, but it becomes much harder for bacteria operating in soil in the field for months. Furthermore, as more synthetic parts are added to the cell, they can interfere with each other’s function, which can change a sensor’s response.^2188^ The application of control theory and the use of genetic insulation to build reliable sensors is a promising area of research, but it has not yet been applied to create sensors that reliably operate under field conditions.^1279,2189–2191^ Finally, most genetic sensors have been characterized using higher-copy plasmids due to the larger response they generate. Ultimately, they must be encoded in the genome to be carried stably in the field and reduce the burden on the cell.^2192^

This section focuses on two classes of sensors. The first are those that have been developed for agriculturally-relevant bacterial species. The second are sensors for agriculturally-relevant signals that have been demonstrated in model species. Many synthetic sensors can be put together in one cell; up to 12 that respond to small molecules have been placed into one strain.^2193,2194^ Collectively, genetic sensors can provide an incredible breadth of operational awareness to the cell.

##### 3.4.1.1 In-Field Inducible Systems

Engineered bacteria could be induced in the field using a sprayed agrochemical. Copper-based pesticides, including copper sulfate, copper hydroxide, copper oxychloride, and copper oxide, are used for crop protection, particularly in organic farming.^2195,2196^ A bacterial sensor responsive to copper sulfate was built by expressing CueR in *Escherichia coli* along with its native cognate promoter and knocking out a copper exporter to increase sensitivity.^2197^ This sensor could detect μM levels of copper in water from field samples.^2197^ Monosodium methylarsonic acid (MSMA) is an herbicide that is approved in the United States.^2198^ A sensor for organic arsenic compounds, including methylarsenite, was built by expressing a mutated ArsR repressor that is insensitive to inorganic arsenic.^2199^ The sensor was turned on in the presence of *Burkholderia* sp. MR1, which reduces MSMA to methylarsenite.^2199^ A sensor that responds to the fungicide Mandipropamid was built by using a split T7 RNAP system fused to protein-ligand interaction domains and shown to function in *Agrobacterium tumefaciens*.^2200^

##### 3.4.1.2 Measuring Soil Nutrients

Genetic sensors have been developed that can monitor nutrient levels. These sensors could be used to regulate microbial activity to create or scavenge for these nutrients, such as nitrogen fixation and phosphate solubilization.

Nitrogen is critical for crop yields due to its role as a vital nutrient for plant growth. A nitrate sensor in *Bacillus subtilis* was constructed by moving the two-component system NarXL from *Escherichia coli* and swapping its DNA binding domain with one from *Bacillus subtilis* (Figure 31A).^2201,2202^ Sensitivity was improved by mutating residues to reduce phosphatase activity of the NarX histidine kinase, resulting in a sensor that could report on nitrate levels from 6 - 600 μM in fertilized soil, noting that the threshold for nitrogen deficiency is 200 μM.^2201,2203^ Sensors have also been developed for free amino acids (aspartate,^2201^ glutamate,^2204^ lysine,^2205^ arginine,^2205^ tryptophan,^2206^ valine,^2207^ β-alanine,^2180^ phenylalanine,^2180^ tyrosine^2180^), which are important sources of nitrogen in low fertility soils.^2208^

**Figure 31:**
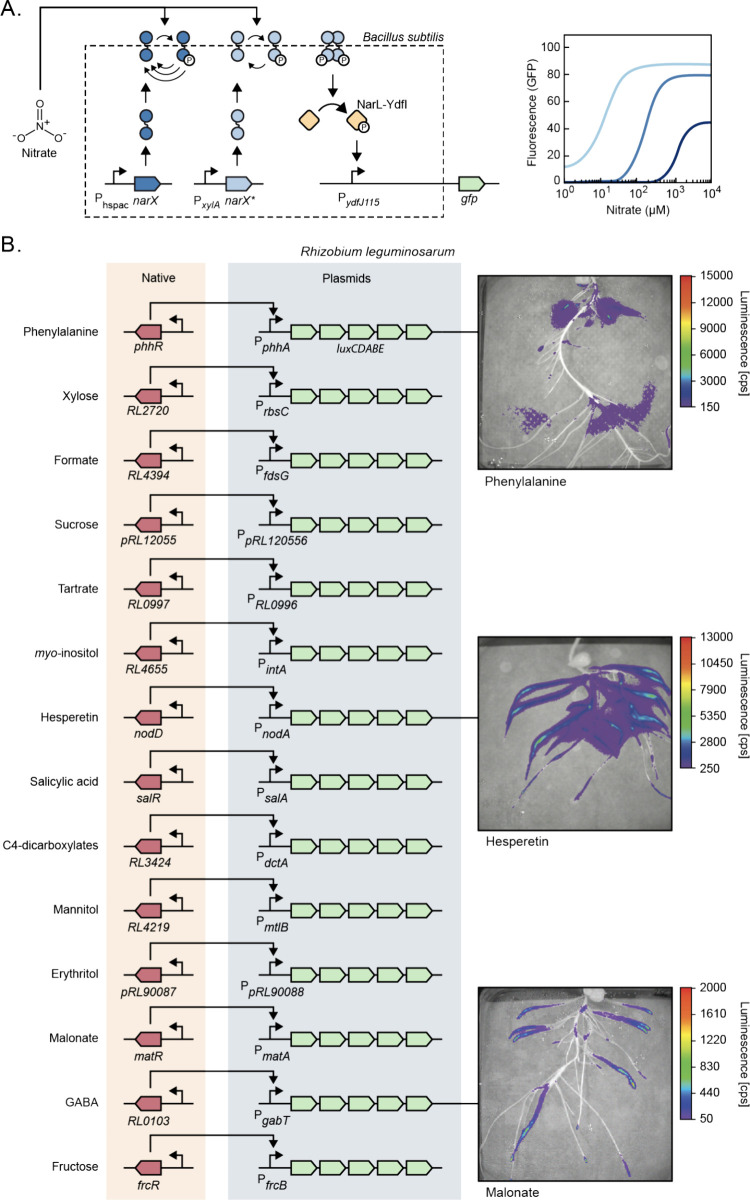
**Microbial sensors for agriculturally-relevant molecules**. **A**. A nitrate sensor with tunable threshold in *Bacillus subtilis*.^2201^ NarX is a nitrate-responsive sensor kinase, whose phosphatase activity was reduced through mutation to create NarX*. Phosphorylated NarX activates the response regulator NarL-YdfL through phosphorylation. Changing the ratio of NarX and NarX* by tuning their individual expression via part selection alters the response function of the NarX-NarL sensor. **B**. A suite of sensors made from native promoters was used to visually map the presence of root exudates in the pea rhizosphere.^2266^ The promoters of genes (orange box) previously identified to be differentially expressed in *Rhizobium leguminosarum bv viciae* strain 3841 during rhizosphere colonization were cloned upstream of a luciferase reporter operon. The strains containing sensors were inoculated on pea seedling roots growing on agar plates. In the images, the bacteria are throughout the agar plate but only produce luciferase when they sense the root exudate that turns on their sensor. Luminescence images reproduced with permission from ref 2266. Available under a CC BY license. Copyright 2017, Plant Physiology.

Phosphate is a component of fertilizer that is required for cell division and plant growth.^2209^ Numerous bacteria sense external phosphate concentrations using a two-component system (*e.g.*, PhoBR).^2210^ Sensors were built using the PhoBR-responsive *phoA* promoter in *Pseudomonas fluorescens*, and *Synechoccus* sp. strain PCC 7942 and used to estimate phosphate concentration in wastewater and environmental water samples.^2211–2213^

There are seven essential plant micronutrients required for growth: iron, zinc, copper, manganese, molybdenum, boron, and chlorine. A Fe^3+^ sensor was built in *Escherichia coli* through the heterologous expression of the regulator DtxR.^2214^ However Fe^3+^ is not the best indicator of iron deficiency, as it is not bioavailable.^2215^ To this end, the ferric uptake regulator (Fur) directly senses Fe^2+^ and derepresses iron importer genes in *Salmonella*.^2216^ The sensitivity of the sensor was tuned by expressing a protein that sequesters Fur. A zinc sensor based on ZntR was capable of detecting Zn^2+^ in soil when transferred to *Pseudomonas fluorescens*.^2217,2218^ Copper sensors include the activator CueR (*Escherichia coli* and Pseudomonads) and the repressor CsoR/YcnK (*Bacillus subtilis*).^2197,2219^ Mn sensors are common in nitrogen-fixing bacteria.^2220,2221^ MntR serves both as an activator at low Mn^2+^ concentrations and a repressor when [Mn^2+^] > 1 μM.^2222^ Mo can be sensed by ModE, which activates a promoter in its absence and can be found in *Escherichia coli*, *Bradyrhizobium*, and *Xanthomonas*.^2223–2225^ No sensors of boron or chlorine ions have been reported, despite evidence that they impact gene expression in soil bacteria.^2226–2228^

##### 3.4.1.3 Responding to Pollution

The detection of polluted soil is important for both plant and consumer health.^2229^ Bacterial sensors have been built for heavy metals, including arsenic,^2230–2232^ gold,^2233^ nickel,^2234^ chromate,^2235^ cadmium,^2236^ lead,^2237^ and mercury.^2218^ Several have been tested on soil samples. An *Escherichia coli* mercury sensor based on MerR was sensitive to 20 - 110 μg/kg soil.^2238^ Foods grown in soil mercury levels of 37 μg/kg are safe for consumption, but foods grown at >120 μg/kg were not, indicating that this sensor is tuned to a relevant range.^1733^ An array of heavy metal sensors was constructed by expressing metal-responsive regulators in various soil bacteria and used to measure cadmium, zinc, and mercury concentrations in soil (CadR, ZntR, MerB/MerR in *Pseudomonas fluorescens*; ZntR, MerR/MerB in *Escherichia coli*; CadC in *Bacillus subtilis*).^2218^ Zinc and copper sensors, based on the ZraSR and CusSR two-component systems have been used to turn on metal bioremediation pathways.^2239^

Sensors have been developed to detect chemical pollutants, such as phenanthrene, phenolic compounds, and organophosphates.^2238,2240^ Sensors for phenols, a common carcinogen in ground water, were developed by mutagenizing DmpR to bind to 2-chlorophenol, 2,4-dichlorophenol, and 4-nitrolphenol in Pseudomonas.^2241–2243^ The protein design software Rosetta (Section 2.4.1) was used to predict PobR mutations that bind *p*-nitrophenol, a byproduct of organophosphate pesticides.^2244^ To make a sensor of the insecticide paraoxon, the mutated PobR was combined with the expression of a phosphotriesterase that degrades paraoxon to *p*-nitrophenol.

Benzene, toluene, ethylbenzene, and xylene (BTEX) are a family of structurally-related hydrocarbons that are pollutants prioritized by the EPA.^2245^ Sensors for BTEX were built by mutating MopR, which binds phenol, to instead bind benzene or m-xylene.^2246^ Sensors for alkanes and alkenes usually respond to compounds of different chain length. Sensors for medium-chain alkanes in *Alcanivorax borkumensis* SK2 and *Escherichia coli* were constructed based on the activator AlkS.^2247–2252^ AlkS has been mutated to respond to various branched-chain alcohols.^2253^ Similarly, alkane/alkene biosensors in *Acinetobacteri baylyi* have been constructed using the activator AlkR.^2251,2254^ A butanol sensor was built in *Escherichia coli* using the BmoR activator.^2255^

Compounds used in explosives are a pervasive environmental contaminant that affects both water and cropland.^2256^ Sensors for TNT and DNT have been developed in *Pseudomonas putida* by evolving XylR, a toluene-responsive repressor, to be responsive to nitrotoluene.^2257,2258^ A riboswitch for DNT has been built that turns on expression from mRNA in the presence of the compound.^2259–2261^ Its response threshold is 9.1 mg/L, which is within the range of contaminated wastewater. The *Escherichia coli yqjF* promoter responds to DNT/TNT and has been used to map landmines in the field.^2262–2264^ In *B. subtilis*, a TNT-responsive translational riboswitch regulated the expression of a phage integrase to invert a promoter upstream of a *rfp* reporter.^2265^

##### 3.4.1.4 Pathogen and Pest Detection

The detection of plant pathogens in the field could guide an intervention with an agrichemical treatment. The sensor could also induce antibiotic production by the cell or even communicate this information to the plant to direct a response. Bacterial pathogens sometimes use quorum sensing molecules to coordinate pathogenesis. For example, some plant pathogens produce C10-HSL (N-decanoyl-L-Homoserine lactone), including *Pseudomonas fuscovaginae*, which causes sheath brown rot in rice.^2267^ Sensors of these pathogens have been constructed by expressing regulator proteins that respond to their quorum sensing molecules (Section 2.3.2).^1799,2268^ *Escherichia coli* has been engineered to detect the soil bacterium *Burkholderia pseudomallei* by sensing C10-HSL and 3OHC10-HSL (N-3-hydroxydecanoyl-L-Homoserine lactone).^2269^

DNA sensors have been developed in *Bacillus subtilis* and *Acinetobacter baylyi* that can detect genetic material shed by pathogens.^2270,2271^ In each of these sensors, the bacteria are naturally competent and take up foreign DNA. The target sequence is pre-encoded in their genome, separated by genetic parts that disrupt the sequence. For example, in one embodiment, there is an upstream promoter and then a terminator in the middle of the target sequence. The uptake of DNA from the environment and its matching to the genomically-encoded sequence causes the terminator to excise and RNAP form the upstream promoter to read through to the end of the target DNA after which there is a reporter or regulatory gene. Another way to implement a DNA sensor is to design the integration to excise a repressor so that a repressed promoter turns on. These sensors could detect gut pathogens and mammalian DNA at concentrations as low as pM. They could be designed to detect agricultural pathogens, insect, or herbivore DNA.

#### 3.4.2 Chemical Communication between Cells

There is a rich chemical language that cells use to communicate. This communication could be within a species of bacteria. For example, it could be there to delay biofilm formation or pathogenesis until a desired cell concentration threshold has been crossed. Bacteria are also constantly surveying the environment for potential competitors based on the chemicals they leave in their wake. Finally, plants can communicate with each other through pheromones, to insects, and to bacteria and fungi via the chemicals they secrete from their roots (Section 3.1.7). The genetics underlying the production and receipt of these chemical signals have been retooled to create a programmable language between cells.

##### 3.4.2.1 Cell Density Sensors

Quorum sensors respond to cell density. The system consists of an enzyme that produces a small molecule that crosses the cell membrane and accumulates in the environment. Gram-negative bacteria use acetyl-homoserine lactones (AHLs) whereas Gram-positive bacteria have systems that rely on peptides as the communication signal. When the concentration passes a threshold due to high cell density, it binds its corresponding regulatory protein and turns on gene expression. These systems often control programs of gene expression to form biofilms, including on the surface of roots and leaves.^2272–2278^ They are important for bacteria that are used as biocontrol agents and plant growth promoters. They are also involved in programs of plant pathogenesis.^1803^ Biocontrol bacteria have been engineered to produce enzymes that disrupt these signals as a means to block plant pathogens (Section 4.1.1.2).^2279,2280^ Quorum sensing also is used by bacteria to determine proximity for the horizontal transfer of DNA.^2281^

Quorum systems are widespread across soil bacteria; for example, in the oat rhizosphere, 24% of the species produce AHLs.^2282^ They are essential for the beneficial effects of plant-growth promoting bacteria (Rhizobium and Pseudomonas) and for the damage of pathogens (Agrobacterium, Erwinia, Pantoea, Xanthomonas).^2283^ Soil particles promote quorum sensing and increase biofilm formation by *Pseudomonas aeruginosa* by constraining the bacteria in micro-niches and facilitating cell aggregation.^2284^

Quorum sensors can be used synthetically as a means to implement a response after a threshold in cell density has been crossed. For example, it was used to create a set-point for the maximum cell density by using it to control a protein that is toxic to the bacterium or to switch from growth to production modes in a bioreactor.^2285–2287^ Outside of laboratory cultures, the quorum signal could be used a proximity sensor to determine when the bacteria are occupying a physically confined niche.^2288^

Quorum sensors have been used for applications in agriculture. *Cupriavidus pinatubonesis* has been engineered to put the auxin IAA under the control of a quorum sensor to enhance *Arabidopsis* root growth to avoid making the hormone too early when it can inhibit plant growth.^2289^ The nitrogen fixation gene cluster has been transferred to *Escherichia coli* and placed under the control of different quorum systems, thus allowing bacterial biomass to accumulate before turning on this energy-intensive enzyme.^2027^ The soil bacterium *Bacillus subtilis* possesses a quorum system whose signal is received by both a membrane receptor (ComQXP) and a cytoplasmic regulatory protein (Rap-Phr).^2290^ Using this system, genetic switches were developed for cell density-dependent repression or activation of recombinant genes.^2291^

##### 3.4.2.2 Microbe-Microbe Communication Channels

Bacteria naturally communicate amongst each other using small molecules or peptides that diffuse between them. Gaining control over them to build synthetic communication channels allows information to be transmitted between cells. These channels are a common tool in Synthetic Biology, where programmable communication consists of a “sender device” that produces the signal and a “receiver device” that responds to it.^728^ The input to a sender device is a transcriptional signal (a promoter) and the output is the expression of the enzyme(s) that synthesize the communication signal. The “receiver device” expresses the regulator that binds to this signal and turns on an output promoter. Orthogonal communication channels, consisting of different chemical signals, allow independent lines of information to be transmitted between cells.^2292–2294^

The AHL class of quorum sensing molecules have been co-opted for this purpose. These molecules diffuse rapidly through cell membranes. Multiple sender:receiver pairs have been characterized, including (synthase→molecule→TF): LuxI→3OC6-HSL→LuxR, LasI→3OC12-HSL→LasR, CinI→3OHC14-HSL→CinR, TraI→3OC8-HSL→TraR.^2295,2296^ Directed evolution has been used to identify mutants of the enzyme and regulator with improved specificity and dynamic range.^2292^ These sensors are associated with Gram-negative bacteria, but they have been shown to function in many bacterial species, including Gram-positives, as well as across fungi, plants, and mammalian cells.^2293^ Most AHLs diffuse across all of the membranes of these cells.

Soil chemistry can affect the lifetime, diffusion, and behavior of AHLs in soil and therefore affect their performance as a communication signal. Biochar, a common component of soil, reduces the effective concentration of many AHLs through absorption and pH-dependent hydrolysis.^2297^ The effect of biochar is dependent on its physical properties. Biochar manufactured with a high surface area inhibited 3OC12-HSL-mediated signaling 10-fold more than low-surface area biochar.^2298^ Likewise, the rate of absorption of AHLs to colloidal clays differed depending on the identity of the AHL and the identity of the clay.^2299^

Gram-positive bacteria use peptide-based quorum systems.^2300^ A pre-peptide is expressed and modified as it is exported from the cell. The receiver is a membrane-bound protein that binds to the peptide and transmits the signal to a TF, which regulates a promoter. For example, the *agr* system from *Staphylococcus aureus* encodes the propeptide AgrD that is processed by the enzyme AgrB to produce AIP (auto-inducing peptide).^2301^ AIP is sensed by a two-component membrane-bound receptor AgrC and the signal transferred to ArgA, which regulates a promoter. This system has been transferred to the soil bacterium *Bacillus megaterium*.^2302,2303^

##### 3.4.2.3 Plant-Microbe Communication Channels

Plants nurture communities of bacteria and fungi in the rhizosphere by excreting carbon sources (sugars, organic acids, amino acids, etc.) and signaling compounds from their roots.^2304^ Engineering microbes to respond to plant exudates could be used to only activate microbes when a specific need occurs or when they are proximal to a plant. In some cases, root exudates may be an indirect indication of the plant health. For example, nitrogen deficiency decreases amino acid exudation in maize,^2305^ phosphorus deficiency increases organic acid (citrate, malate, and succinate) exudation,^2306^ and iron deficiency leads to coumarin exudation.^2306^

Sensors have been developed that respond to root exudates and turn on in bacteria adhered to plant roots. Microbial biosensors were constructed in the plant-associated species *Pantoea agglomerans* by fusing native sucrose-, fructose-, tryptophan-, or galactose-responsive promoters to reporter genes.^2307–2310^ A genetic sensor based on the native galactose/galactoside-induced *melA* promoter in *Sinorhizobium meliloti* could sense galactosides, and sugars found in root exudates up to 200 μm from the root surface.^2310^ To identify promoters that respond to root exudates, a set was gleaned from genome of the legume symbiont *Rhizobium leguminosarum* and screened, yielding sensors for erythritol, myo-inositol, carboxylates (formate, malonate, tartrate), amino acids (phenylalanine, GABA) and flavonoids (hesperetin).^2311^ Bacteria carrying these sensors were inoculated onto pea roots to study the spatial and temporal changes in exudates in the nodule (Figure 31B).^2266^

Many sensors for root exudates have been constructed by introducing heterologous regulators and promoters. The simplest sensors are based on a regulatory protein that binds to a sugar, organic acid, flavonoid, amino acid or other natural product. Sensors have been built for the following compounds (regulator name): cumate (CymR),^2039,2312^ vanillate (VanR, QacR),^1796,2193,2313^ benzoate (BenM),^2180^ *p*-coumarate (PadR),^2314,2315^ protocatechuic acid (PcaV),^2316^ tartrate (TtdR),^2180^ succinate (DcuRS),^2255^ malate (MalKZ),^2317^ fumarate (DcuSZ),^2318^ glutarate (CsiR),^2319^ arabinose (AraC),^1796,2180,2193^ xylose (XylR),^2039^ cellobiose (CelR),^2320^ galactose (GalR),^2320^ ribose (RbsR),^2320^ fructose (ScrR),^2320^ gentiobiose (LacI mutant),^2321^ fucose (LacI mutant),^2321^ naringenin (FdeR, TtgR),^1796,2193,2322,2323^ kaempferol (QdoR),^2323^ quercetin (QdoR),^2323^ lysine (LysG),^2205^ arginine (ArgP),^2205^ tryptophan (TrpR),^2206^ β-alanine (OapR),^2180^ phenylalanine (PhhR),^2180^ GABA (GabR),^2180^ tyrosine (HpdA),^2180^ stilbenes (Saro_0803),^2324^ and xanthine (XdhR).^2180^ Note that, while these sensors are functional, most have been demonstrated in a model organism, usually *Escherichia coli*, and have not been shown to operate in soil or respond to exudates coming from a plant root.

Bacteria have also been engineered to sense several volatile chemicals and phytohormones naturally produced by plants. Salicylic acid is a major plant hormone best known for its role in the plant defense response, but it also is involved in plant growth, development, and abiotic stress tolerance.^2325^ Salicylic acid is recognized by the activator NahR, which has been used to build sensors in *Escherichia coli* ^1338,2180^ and in the soil bacteria *Cupriavidus necator*,^2180^ *Pseudomonas putida*,^2180^ *Rhizobium* sp. IRBG79,^2326^ and *Azorhizobium caulinodans*.^2326^ Isoprene is an abundant plant-produced volatile that may be involved in thermo- and photo-protection.^2327,2328^ *Pseudomonas putida* expressing the TbuT regulator responded to isoprene.^2329^ A sensor for the phytohormone cytokinin *trans*-zeatin was built by fusing the *Arabidopsis* AHK4 sensory domain to the PhoQP two-component system from *Escherichia coli*.^2330^

The sensors described thus far respond to natural exudates that are common to many plant species. Bacteria engineered to respond to these molecules would also respond to other plants producing the same exudate. Building a bacterium to respond to a specific plant could be done by engineering the plant to secrete a molecule not normally produced in exudate and then designing a receiver for this molecule in the microbe. An implementation of this approach used phloroglucinol as the signaling molecule. It is an antimicrobial precursor made by some plant-growth-promoting Pseudomonads. The PKS producing this molecule can be expressed in *Arabidopsis*.^2331^ Synthetic sensors that respond to phloroglucinol were built in *Pseudomonas protegens* Pf-5 and *Rhizobium sp*. IRBG74.^1338,1796^ *Arabidopsis* and barley have also been engineered to artificially excrete opines and rhizopine from their roots (Figure 32).^1816,2332,2333^ Sensors for these molecules have been built for *Azorhizobium caulinodans*. Barley has been engineered to excrete the rhizopine *scyllo*-inosamine and this can be sensed by *Rhizobium leguminosarum* or *Azorhizobium caulinodans* carrying sensors for this molecule.^1796,1816^

**Figure 32:**
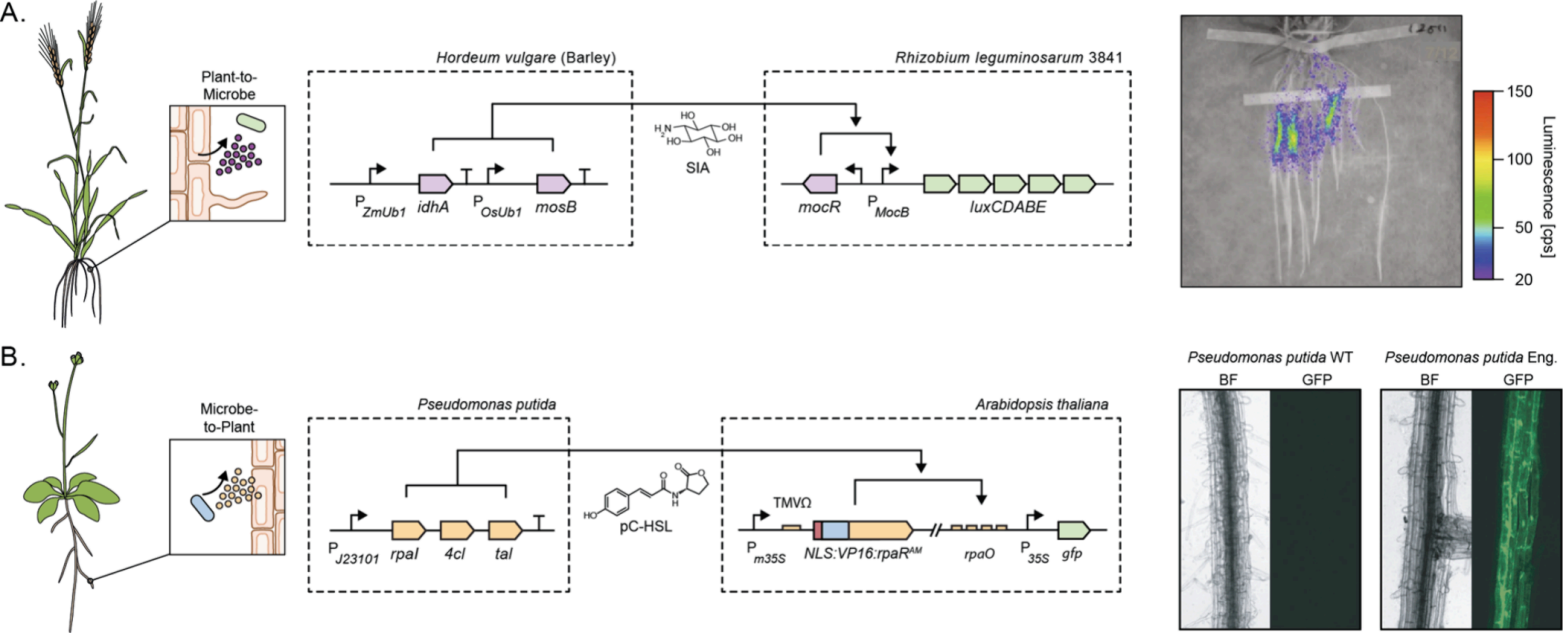
**Engineered plant-to-microbe communication**. **A**. Engineered barley communicating to engineered *R. leguminosarum* through scyllo-inosamine (SIA) biosynthesis. The MocR biosensor expressed luciferase, which can be visualized in a light box when bacteria are close to the roots producing SIA (the bacteria are throughout the agar plate). cps: counts per second. Luminescence images reproduced with permission from ref 1816. Available under a CC BY license. Copyright 2019, Nature Communications. **B**. *Pseudomonas putida* using a pC-HSL sender device to communicate with an *Arabidopsis* plant receiver device.^1310^ A synthetic pC-HSL biosensor composed of the pC-HSL-responsive transcriptional regulator rpaR^AM^ fused to a SV40 nuclear localization signal (NLS) and activation domain (VP16) and expressed from a minimal 35S promoter and tobacco mosaic virus translation enhancer (TMVΩ) was stably transformed into *Arabidopsis*. In the presence of pC-HSL, rpaR^AM^ binds to its cognate operate sites (rpaO) and activates transcription of *gfp* from the adjacent minimal 35S promoter (P_m35S_). Bright-field (BF) and fluorescent (GFP) microscopy images show an *Arabidopsis* root with either wild-type *P. putida* (WT) or a *P. putida* strain expressing the pC-HSL biosynthesis pathway (Engin.). Root images reproduced with permission from ref 1310. Available under a CC BY license. Copyright 2024, Nature Communications.

To date, the molecules that have been used for plant-microbe communication are naturally produced somewhere in the rhizosphere. Molecules such as opines are uncommon, but still present, leading to the potential for off-target interactions. From the perspective of establishing orthogonal communication signals that are unique to a plant:microbe pair, the molecule would have to be not present in the rhizosphere. The ideal molecules would be non-toxic compounds that diffuse through soil without sticking to soil particles and also easily pass through the plant and microbial membranes. In designing pathways to these molecules, computational tools have been developed to combine enzymes from different sources to build non-natural compounds.^997,2334^ Corresponding sensors for these compounds could be built by mutating binding proteins that can transduce their signal to a promoter (Section 3.2.8).

#### 3.4.3 Synthetic Genetic Circuits

An individual sensor produces a simple response: as the input signal increases, the output promoter monotonically turns on or off. To change the response, sensors can be connected to genetic circuits, implemented through interacting TFs.^887,1352^ In plants, synthetic circuits were described in Section 2.3.5, but the range of circuits that have been built in bacteria is much greater. However, the majority of these circuits have been implemented in model species, such as *Escherichia coli*. The classes of responses include:^887^

- **Switches** that alter the threshold, cooperativity, and amplification or implement noise filters.^1337,1374,2335–2339^

- **NOT gates** that invert the response, for example turn a light sensor into a dark sensor.^1340,2340,2341^

- **Biphasic circuits** that are on at only intermediate levels of input, implementable with incoherent feedback loops or promoter design.^1337,2342^

- **Digital and fuzzy logic** to integrate multiple sensors.^2343–2348^ This integration can improve the specificity of a response (*e.g.*, turning on in low O_2_ AND high T AND N stress).^2170,2349,2350^

- **Analog circuits** to perform addition, ratiometric comparisons, or serve as perceptrons in a neural network.^1351,2351–2353^

- **Dynamic circuits**, such as a pulse generator or oscillator.^1263,1264,2354–2356^

- **Adaptive circuits** that return to their initial state despite a step change in input, sometimes described as a “pulse generator”.^2357,2358^

- **Feedback controllers** that apply principles from control theory to make the output more robust to perturbations in the environment or cell state.^358,388,2359^

- **Memory** to permanently record a transient signal or combination of singals.^1367,2360–2363^

- **Algorithms** from computer science. Genetic circuits have been developed that implement entire algorithm, including the binary-coded digit (BDC) 7-segment display algorithm and a 2-bit version of the MD5 cryptography algorithm.^2364,2365^

Transcriptional sensors and circuits are easy to connect because the input/output signals are the same (extensible): a promoter.^2344^ The output promoter of a sensor can serve as the input promoter for a circuit. Similarly, both the inputs and outputs of a circuit are promoters so that they can be connected to each together, where the output of an upstream circuit serves as the input to the downstream circuit. Multiple circuits can be connected in series (“layered”) to create more complex operations, most commonly referred to as a “cascade” in biology. While this makes them easy to connect, transcriptional circuits are slow, requiring 0.5 - 2 hours for each layer to complete. Delays in the calculation can lead to transient errors (“faults”) where genes are mis-expressed as the circuit changes state.^1363,2344^

This extensibility has led to rapid growth in the scale of genetic circuits in *Escherichia coli*, now incorporating dozens of TFs solve complex computational problems.^2366–2368^ Further, the division of circuits across a consortium of interacting cells has increased their scale dramatically.^2369–2371^ As the designs have gotten more complicated and require more genetic parts, design automation and simulation software packages have become more prominent (Section 3.6.1.4).^1509,2362,2372,2373^ One can imagine a future where the bacteria occupying a biofilm on a seed, root, or leaf work together to compute something about the environment and report this information to the plant.

This section highlights the circuits that have been built in soil bacteria or are applicable to agriculture. The most advanced capabilities of genetic circuits have not yet been applied to these species. One challenge in using circuits in strains that will be applied to agricultural fields is that the additional genetic parts and TF genes are usually sourced from diverse genera and are therefore more difficult to obtain regulatory approval from USDA/EPA^6^.

##### 3.4.3.1 Layering Circuits (Transcriptional Cascade)

When building a synthetic genetic circuit, the layering of TFs has been used to change the response of the output.^2374–2376^ It can make it more switch-like, change the threshold, amplify the output, or reduce the cell-cell variability (noise).

Circuits have been layered to make sensors more sensitive to environmental pollutants. A transcriptional cascade was used to amplify the output from arsenic (ArsR) and mercury (MerR) sensors.^2231^ Adding a three-layer cascade to the arsenic sensor shifted its limit of detection from 10 ppb to 0.8 ppb and increased the output 20-fold (Figure 33A).^2231^

**Figure 33:**
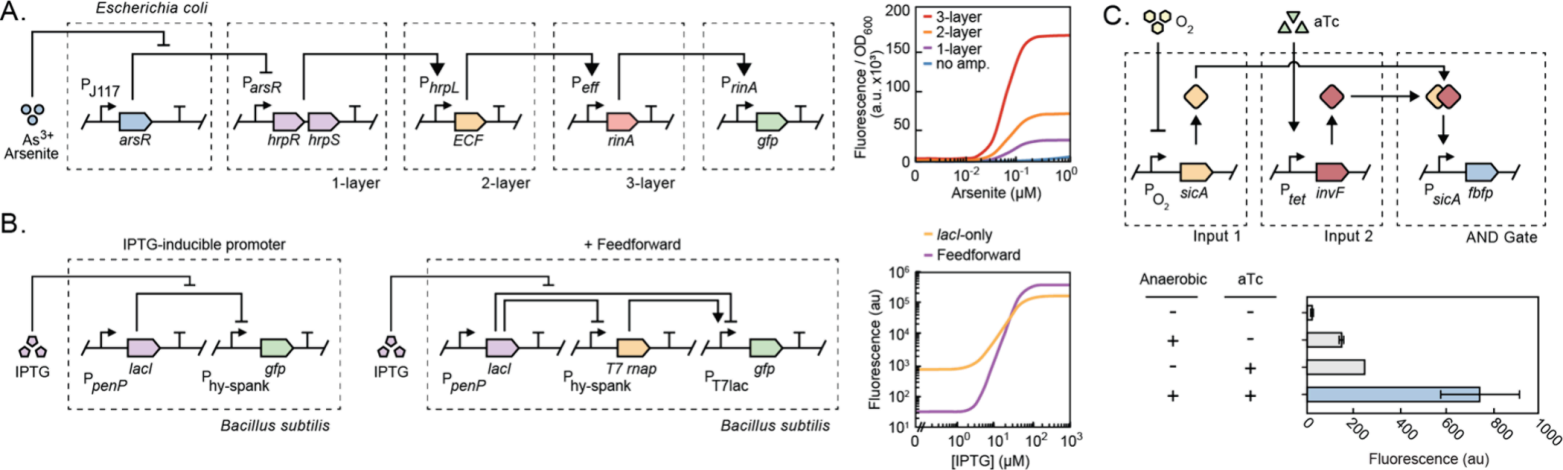
**Layered genetic circuits in soil bacteria**. **A**. A synthetic cascade to control the threshold of an arsenite (As^3+^) sensor in *Escherichia coli*.^2231^ The output of the sensor (P_arsR_) has a weak response (no amp.). In the graph, the promoter is fused to create the 1-, 2-, and 3-layer cascades (P_hrpL_, P_eff_, and P_rniA_, respectively). **B**. Amplifying induction in *Bacillus subtilis* using a feedforward circuit.^2377^ The IPTG-inducible promoter P_hy‑spank_ has a limited dynamic range in *Bacillus subtilis*. By introducing a feedforward loop, where *lacI* regulates the expression of both T7 RNAP and its downstream output (P_T7lac_) the dynamic range increased to 10,000-fold. **C**. Building a two-input AND gate in the cyanobacteria *Synechocystis* sp. PCC 6803.^2172^ The fluorescent protein (*fbfp*) output is on only under anaerobic conditions and in the presence of the inducer anhydrotetracycline (aTc). The AND gate involves the expression of the TF InvF and the chaperon SicA required for its activity. The output promoter of the gate is P_sicA_.

##### 3.4.3.2 Feedforward and Feedback Loops

A feedforward motif splits the input signal, which then reconverges on the output. Feedforward motifs are prevalent in natural regulatory networks and synthetic feedforward motifs have been built.^2354,2378–2388^ Feedforward motifs can be “coherent” where both the input and the split signal converge with the same sign (both turn the output on or both turn it off) or “incoherent” where one has a different sign than the other (one turns it on and the other off).^2389^ Incoherent feedforward motifs occur frequently in natural regulatory networks and synthetic feedforward motifs have been shown to filter noise or produce a pulse of activity.^1210^ They can also reduce the context effects of carrying a sensor at different copy numbers due to being carried at different locations in the genome or changes in the growth rate or medium.^358^ A feedforward motif was constructed in *Bacillus subtilis* to increase the dynamic range of an IPTG-sensor from 280-fold to 10,000-fold (Figure 33B).^2377^

Positive feedback loops occur when a gene product activates its own expression. They are a common motif in natural regulatory networks.^2390^ Synthetic positive feedback loops can amplify outputs from a sensor. They can also be used to build a bistable switch, which exhibits hysteresis and memory.^2391^ The output of an aTc sensor in the nitrogen-fixing cyanobacterium *Anabaena* sp. PCC7120 was amplified using positive feedback.^2392^

Negative feedback occurs when a promoter turns on the expression of a repressor that then turns off the promoter. They are common in natural regulatory networks and can reduce the toxicity of TF expression, reduce cell-to-cell variability, make a switch response faster, and decrease the impact of mutations that accumulate during evolution.^2393–2396^ Negative feedback loops have been used to maintain a constant level of recombinant gene expression when moved between species. To this end, the autoregulation of a T7 RNAP dependent promoter reduced the variability of a reporter when transferred between the soil bacteria *Pseudomonas putida* and *Bacillus subtilis*.^2003^

Cross-repression occurs when two repressors influence each other’s expression.^2397^ This motif is common in natural networks.^1367^ Synthetic circuits that have this motif are referred to as “toggle switches” because they can exist in a state expressing one repressor or the other, but not both. It can lead to a bistable switch, where at a particular level of input, the circuit could be in either a high or low state. Bistability leads to different thresholds for the switch to turn on and off (hysteresis), which makes the switch resilient at the threshold. A toggle switch has been built on the *Bacillus subtilis* genome using the LacI and XylR repressors.^1895^

##### 3.4.3.3 Logic Operations

Logic circuits perform a function on a set of inputs to convert them to a set of outputs. In electronic circuits, the inputs and outputs are binary (0 or 1), but in biological systems they can take on intermediate “fuzzy” levels. Logic circuits can have any number of inputs or outputs. Familiar 2-input, 1-ouput logic operations include OR, NOR, NAND, AND, and XOR. They can be connected to build more complex multi-input multi-output logic circuits. Such genetic circuits can turn on different genes or phenotypes under different combinations of input activities.

Logic circuits can be used to increase the specificity of a response. Metal sensors sometimes have limited specificity due to crosstalk between divalent metal ions (Hg^2+^, Cd^2+^, Pb^2+^, Zn^2+^) binding to the regulatory protein.^2218,2398^ To create sensors with higher specificity, an AND gates were built in *Escherichia coli* by having two sensors express different TFs, both of which were required for an output promoter to turn on.^2399^ This approach was used to build a specific Zn^2+^ sensor out of regulators that bind nonspecifically to Zn^2+^, Pb^2+^, Zn^2+^, and Cd^2+^. Additional AND gates were also built for combinations of the heavy metals As^3+^, Hg^2+^, and Cu^2+^ and small molecules IPTG, arabinose, and 3OC6-HSL.

AND gates can also make a sensor active only under defined environmental conditions. For example, an AND gate was built in the cyanobacteria *Synechocystic* sp. PCC 6803 to turn on an output promoter in the presence of a small molecule inducer, but only under conditions of low oxygen (Figure 33C).^2172^ The AND gate was based on two TFs, both of which are needed to activate the output promoter. This gate could be used applied to induce nitrogenase expression only under conditions where it is not inactivated by oxygen (Section 4.2).

#### 3.4.4 Remote Detection of Microbial and Plant Biosensors

Precision agriculture uses remote imaging technologies to monitor plant health.^2400,2401^ Various camera types mounted on equipment, from tractors to unmanned aerial vehicles (UAVs), interpret images to look for signs of water stress, nutrient dynamics, and pathogen damage. The cameras do this by quantifying chlorophyll fluorescence, thermal infrared imaging, and multi- and hyper-spectral imaging, and other signals. Precision agriculture provides large-scale, real-time assessments of agricultural systems, from the farm scale down to individual plants.

Precision agriculture has relied on the measurement of natural features of the plant. It could also be used to detect changes that are programmed by genetic engineering. The output promoter of a sensor or circuit could drive the expression of a gene that leads to the production of a reporter. The reporter could be a protein on metabolite that is measurable by the camera used in precision agriculture, such as those mounted on UAVs. For example, detecting the early stages of the response to an insect or pathogen would allow the farmer to react before damage to the crop become visible. This section describes different reporters that could be used by microbes to report information via off-the-shelf cameras.

Fluorescent proteins (FPs) are widely-used reporters, compatible with nearly any cell type, that are detected by absorbing one laser wavelength and emitting at another. They have been used in microbial and plant biosensors that monitor soil health, pollutants, and pathogens. For instance, fluorescent proteins can be integrated into plants to create osmotic stress “phytosensors” that can be detected optically from a distance of 3 meters with a pixel resolution of 2.5 cm (Section 2.4.4).^2402^ Microbial biosensors have used fluorescent proteins to report TNT via the degradation product of the TNT residue DNT in soil from 20 meters (Figure 34A).^2264^ The field measurements required a bespoke land-based imaging system that used a laser exciter and telescope to capture the emission of bacteria concentrated in alginate beads filled with growth medium. Sequestering the bacteria enhances the signal and reduces regulatory concerns. Sunlight can be used to excite fluorescent proteins, but the resulting signal is weak.

**Figure 34:**
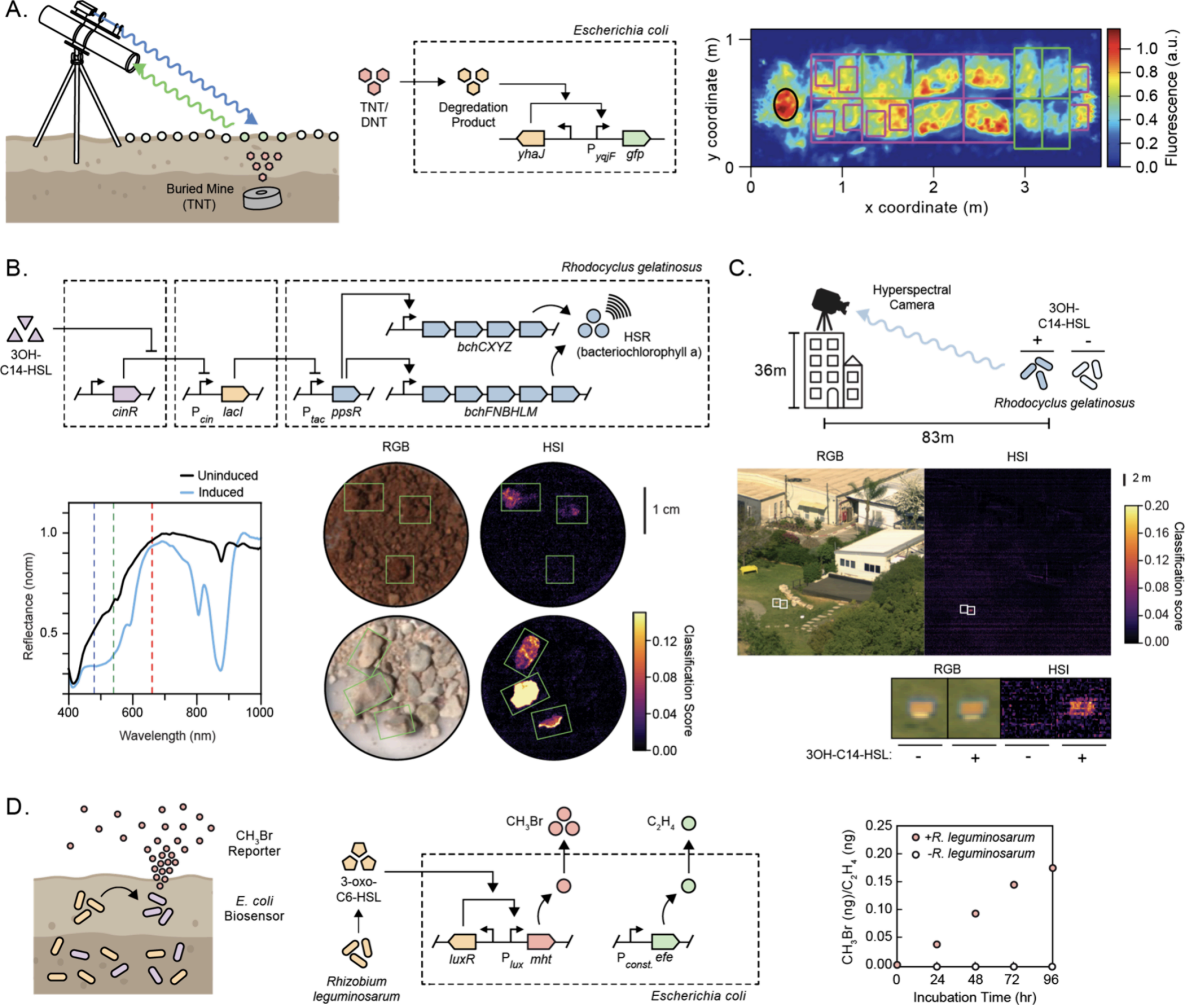
**Remote detection methods utilizing engineered soil bacteria**. **A**. Detection of buried landmines using an encapsulated microbial biosensor (white circles).^2411^ An unknown degradation product of TNT and its main impurity DNT (2,4-dinitrotoluene) activates the transcriptional regulator YhaJ, which induces expression from the *yqjF* promoter. *Escherichia coli* containing the YhaJ sensor were encased in alginate beads, which were spread on top of soil burying TNT anti-personnel landmines and imaged at a distance of 20 m using a laser-imaging device (right figure). The fluorescent output was detected in plots where DNT was applied directly to soil (black circle) or either a land mine or TNT flakes were buried in soil plots for 3-5 months (purple boxes). Fluorescence was not detected in the negative control plots or plots where the land mines were buried 24 h before the test (green boxes). Heat map image reproduced with permission from ref 2411. Copyright 2017, Nature Biotechnology. **B**. Hyperspectral reporters in a microbial sensor.^2409^ The sensor responds to the quorum molecule 3OH-C14-HSL. The sensor output was inverted using a NOT gate (*lacI*) and then connected to the repressor of the bacteriochlorophyll a biosynthetic operon (PpsR). Induction of bacteriochlorophyll a biosynthesis by addition of 3OH-C14-HSL altered the reflectance spectra of engineered *Rubrivivax gelatinosus* outside of the RGB wavelengths (vertical dashed lines). Soil substrates (green boxes) coated in different concentrations of induced *Rubrivivax gelatinosus* could be classified without being visually disguisable in an RGB image. Data figure and soil images reproduced with permission from ref 2409. Copyright 2025, Nature Biotechnology. **C**. Long distance and wide area detection of a bacterial hyperspectral reporter.^2409^ *R. gelatinosus* cultures were spread atop 30 × 30 cm plots of sand that contained filter paper soaked in either 3OH-C14-HSL (+) or no inducer (−). After 29 h the sand plots (white boxes) were imaged inside a 1-acre image using a hyperspectral camera (400–1000 nm) atop a 9-story building. Images reproduced with permission from ref 2409. Copyright 2025, Nature Biotechnology. **D**. Monitoring gene expression in soil using methyl halides.^2410^ The enzyme methyl halide transferase (MHT) produces CH_3_Br, which is volatile and diffuses out of soil. An *Escherichia coli* biosensor strain was engineered to express MHT only in the presence of the quorum molecule 3-oxo-C6-HSL, which is synthesized by *Rhizobium leguminosarum*. To normalize the CH_3_Br concentration to the population of the biosensor strain, the *Escherichia coli* strain constitutively expressed ethylene forming enzyme (EFE) and produced ethylene (C_2_H_4_). The ratio of CH_3_Br to C_2_H_4_ measured in the headspace of soil vials containing the *Escherichia coli* biosensor increased only when *Rhizobium leguminosarum* was added to the sample.

Luminescent reporters are used for remote detection by measuring the light that they produce. Light is generated via biochemical reactions performed by enzymes, sourced from fireflies and other organisms, the output of which is detectable in low-light environments. The instruments required for detection are CCD cameras. The DNT sensor was used to express luciferase and the signal was detectable up to several meters away.^2403^ Plants have also been engineered to be bioluminescent and could be used as biosensors.^2404^ Petunias engineered to be bioluminescent and are commercially sold as an ornamental in the United States (Figure 14).^2405^ They produce a signal that can be clearly seen in the dark by eye.

Hyperspectral imaging (HSI) measures the near-continuous spectrum of light (hundreds of narrow bands). HSI cameras are often used in precision agriculture to detect plant stress indicators, such as nutrient deficiencies and diseases, before they become visible.^2406,2407^ Plant biosensors have been genetically engineered to change their hyperspectral signature in response to a signal in the soil. A synthetic gene circuit in *Arabidopsis* that responds to estrogen was connected to a pathway that causes de-greening (the plant turns white).^2408^

Hyperspectral reporters (HSRs) have been developed to be detectable by HSI cameras. Metabolites with unique spectral signatures were identified using a combination of quantum calculations and spectroscopy.^2409^ Biliverdin IXα and bacteriochlorophyll *a* were identified as metabolites with unique spectral signatures that could be detected above background. The reporter for each was defined as the biosynthetic pathway that can make the molecule. Biosensors were built based on the soil bacterium *Pseudomonas putida* and aquatic bacterium *Rubrivivax gelatinosus* (Figure 34B). They were connected to sensors and could quantify the molecules buried in soil using a UAV-mounted camera. They were detectable on complex backgrounds and under ambient light from long distances (Figure 34C).

Biosensors based on visual reporters need to be near the soil surface. Even then, the soil surface can also be obscured from aerial views, particularly in the field environment where crop canopies or debris are prevalent. There are several ways that a signal could be enhanced from under the surface and detected above ground. First, microbial biosensors could report a signal to a plant root, which then relays it to the leaves to create a measurable signal (Section 3.4.2.3). Second, a volatile chemical could be chosen that diffuses through the soil to the surface. To this end, an enzyme reporter produces methyl bromide. An AHL sensor was connected to this gene and it was shown to be detectable in gas collected above soil (Figure 34D).^2410^ This approach was used to measure the presence of the nitrogen-fixing bacteria *Rhizobium leguminosarum*.

#### 3.4.5 Control of Native Gene Expression

There are applications where it is necessary to control the expression of native genes in the genome. This control can be connected to the output of a genetic sensor or circuit. One example would be to implement dynamic control over metabolism to shunt carbon flux into a different pathway or to shift from growth to a production phase.^2412,2413^ This ability could also be used to turn phenotypes on or off, such as the utilization of different carbon sources.^2414^ Lastly, it can also reduce the expression of a gene that would be lethal if knocked out completely.

##### 3.4.5.1 Regulating Host Transcription

Programmable DNA-binding domains can knock down expression from the genome by interfering with transcription. dCas9 is easy to re-program to new sites as it can be targeted to different genes through the design of gRNA (CRISPRi).^2415^ This technique can simultaneously target multiple genes by transcribing an arrays of multiple gRNAs.^2416,2417^ dCas9 cannot be used to target any site in the genome because of the need for a PAM site; however, new variants of Cas9 are available that have broader PAM specificities, thus allowing gRNAs to be designed for more positions.^41^ CRISPRi has been used in soil bacteria, including *Bacillus subtilis*,^2418,2419^ *Streptomyces coelicolor*,^2420^ *Streptomyces venezuelae*,^2129^ *Pseudomonas putida*,^2048,2421–2423^ *Pseudomonas fluorescens*,^2422^ *Pseudomonas aeruginosa*,^2419,2422^ *Klebsiella pneumoniae*,^2419^ and *Acinetobacter baylyi*.^2424^

A limitation in using dCas9 in non-model hosts is toxicity.^389^ It can arise from transient nonspecific binding of dCas9 to the chromosome even in the absence of gRNAs.^2425,2426^ dCas9 is also a large (>1,000 amino acid) protein that burdens cellular resources when expressed. The expression of dCas9 in *Pseudomonas putida*, for example, has been shown to lead to growth defects.^2421^

dCas9 can also be used to activate gene expression (CRISPRa) by fusing it to an activating domain that recruits RNAP, thereby turning on a promoter. Because the activating domain must interact with the host RNAP, CRISPRa has been more difficult to move across species. CRISPRa has been used in *Streptomyces venezuelae* ^2129^ and *Pseudomonas putida*.^2421^

TALEs and ZFPs can also be programmed to target specific DNA sequences by concatenating DNA-binding domains (Section 2.1.1.).^2427,2428^ They are often used in eukaryotes to control native gene expression and have been used in bacteria in both repressing and activating capacities.^358,2429–2431^ However, they have not seen widespread adoption in bacteria. Their use is hindered in prokaryotes because of their large and repetitive gene structure, low affinity, and large DNA binding sites that are difficult to place within promoters.

##### 3.4.5.2 Post-transcriptional Control

Gene expression can be knocked down post-transcriptionally using small RNAs (sRNAs).^2432,2433^ They can be designed to bind to a target mRNA via base pairing and downregulate gene expression by blocking the RBS so that it cannot recruit the ribosome.^2434^ The dynamic range of the knockdown is tuned by modulating the affinity of the sRNA to its target mRNA or by tuning the sRNA expression levels.^2432,2435^ These strategies have been used to balance gene expression of multiple proteins simultaneously.^2436^ sRNA can be advantageous over CRISPRi as it exerts lower burden on the cells. sRNAs have been used in soil bacteria, including *Pseudomonas putida*,^2437^ *Bacillus subtilis*,^2438^ *Bacillus amyloliquefaciens*,^2439^ and *Streptomyces coelicolor*.^2440^

##### 3.4.5.3 Targeted Protein Degradation

Proteases can tune gene expression by controlling protein stability. For example, Lon protease selectively degrades proteins that are tagged by the mf-ssrA peptide.^2441^ The most well-known is the “LVA tag,” which requires the amino acid sequence RPAANDENYALVA be added to the C-terminus of the targeted protein.^1260^ The LVA tag corresponds to a half-life of 40 min for GFP, and by changing the last three amino acids, this can be tuned between 60 to 110 min for GFP.^1260,1261^ It can make the half-life even shorter (4 min) for repressors and it has been used to accelerate the response speed of genetic circuits.^1263^

Libraries of degradation tags corresponding to differing half-lives have been characterized in *Bacillus subtilis* and *Pseudomonas putida* and their activity is likely conserved amongst a wide range of bacteria.^1260,2007^ Encoding mutated variants of the LVA tag on target proteins in tandem with inducible expression of the mf-lon protease can modulate steady-state protein levels by at least 10-fold in both Gram-negative (*Escherichia coli)* and Gram-positive (*Lactococcus lactis)* bacteria.^2442^

Protein degradation can also be controlled through the inducible expression of proteases that expose a N-terminal degron on the target protein.^2443^ An orthogonal set of proteases sourced from *Potyvirus*, including TEV protease, were used to activate gene expression by exposing a degron tag on a repressor.^2444^ While this strategy has not been applied to non-model bacteria, the strategy should be widely applicable as N-terminal degron sequences are highly conserved across bacterial species and even with bacteria-derived eukaryotic organelles.^2445^ Proteases can also be used to induce protein activity. For example, the antimicrobial peptide abaecin accumulated extracellularly in *Bacillus subtilis* after fusing the peptide to a TEV protease site and a secretory transit signal from β-glucanase.^2446^ Activation of abaecin required the expression of TEV protease, which was slowly released into culture after IPTG induction, to remove the transit tag.

### 3.5 Ensuring Reliable Field Performance

Engineered microbial inoculants must operate as expected in the field, where there is nutrient limitation, competition, and extreme environmental variability.^2447^ Temperature, oxygen, desiccation, and osmolarity all differ depending on the season, timing of the day, soil type and weather. The microbial composition of bacteria and fungi, as well as viruses and animals all vary by climate, region, and plants/animals.^2448–2450^ Carrying a recombinant function places energetic and material burdens on the cell that will compound with the growth effects from these stresses. Therefore, engineered constructs need to be designed to minimize burden, maintain expression levels across variable conditions, and limit breakage due to evolutionary forces.

#### 3.5.1 Minimizing Resource Burden

Cells have finite resources, including RNAPs, ribosomes, tRNAs, amino acids and other metabolites, nucleotides, and ATP.^2451^ When a synthetic genetic system is added to the cell, the expression of recombinant proteins draws from the same resources, placing pressure on processes needed for maintenance, growth, and survival. This effect has been empirically quantified, where the expression of recombinant proteins leads to a corresponding increase in doubling time.^2452^ The impact is continuous, where more burden leads to slower growth. Beyond just growth, burden negatively impacts many processes in the cell.^2453,2454^ It also invokes evolutionary incentive for mutations to “break” the functions encoded by the recombinant DNA.^1372^ It can also cause synthetic sensors and circuits to function errantly and metabolic pathways to decrease product titer.^2455,2456^

Burden could impact the performance of an inoculant in the field by reducing its persistence, ability to colonize and compete, or reducing the expression of a functional pathway. Methods to quantify burden could be used to predict its impact on performance in the field. Burden is specific to the recombinant DNA being introduced and the species. It depends on which genetic parts are present and genes expressed and can vary over time for a dynamic system.

Protein expression requires resources and the number of proteins, their length, the amino acid content and expression level all affect burden.^2457,2458^ Enzymes can either reduce or increase the burden based on their energetic and materials needs. For example, nitrogenase requires approximately 45 ATP per N_2_ fixed (all in) and, if produced constitutively, the bacterium is quickly outcompeted in the field.^2326^ In contrast, a recombinant phenol degradation pathway in Pseudomonas contributes 9 ATP and provides carbon metabolites to central metabolism, making the pathway stable in the field over years.^2459–2462^

There are few means to predict or experimentally quantify the burden imposed by a recombinant system.^2463–2465^ Such a measure could be used to compare alternative designs or to predict the impact of a system on evolutionary robustness. To this end, a “capacity monitor” was developed to measure burden through the constitutive expression of a fluorescent reporter from the *Escherichia coli* genome.^2466^ Increased burden causes the fluorescence to go down. Thus, it serves as a metric for cellular resource availability and correlated well with the stress caused by the induction of heterologous proteins or high-burden metabolic pathways.

Measurements of transcriptional and translational burden can also be made using RNA-seq and ribosome profiling to estimate the number of RNAP and ribosomes that are available to the cell and what fraction are being diverted to the synthetic system being carried.^1989,1990^ Ribosome availability in particular has been linked to the rate of growth and low concentrations can lead to a sharp increase in the system noise.^2467–2469^

##### 3.5.1.1 Sensor/Circuit Design to Reduced Burden

Feedback loops (Section 3.4.3) can be used to maintain the burden below a desired threshold. A synthetic system may have different states of expression that have different degrees of burden. For example, different states of a genetic circuit have different combinations of recombinant TFs expressed.^2163,2170,2470^ The burden could also go up due to shifts in the cell state or environmental fluctuations. The *Escherichia coli* heat shock promoter has been used to respond in real-time to changes in the burden imposed on a cell.^2471^ Its output was used to tune down recombinant gene expression using CRISPRi (Section 3.2.6). If the burden ever gets too large for the cell to handle, the feedback acts to decrease it to acceptable levels.

TFs used to build genetic sensors can also impart burden, especially when overexpressed, due to non-specific DNA binding.^1990^ In natural regulatory networks, TFs often have a negative feedback loop where they downregulate their own expression.^2472^ This principle can also be applied for synthetic sensors.^2473,2474^ For example, to reduce the toxicity of a TF that increased deoxyviolacein production, a negative feedback loop was incorporated into the design.^2475^

##### 3.5.1.2 Metabolic Engineering for Reduced Burden

In addition to enzyme expression, metabolic pathways can impart additional burden through the accumulation of toxic intermediates. One approach to correcting this problem is to screen expression libraries to properly balance enzyme expression so that intermediates do not accumulate.^2476,2477^ For example, a strain of *Pseudomonas putida* used for environmental remediation of ethylene glycol showed reduced growth at high substrate concentrations due to the toxic intermediate glycolaldehyde.^2464,2478^ This burden was corrected by rebalancing enzyme expression.

Burden can also occur as the result of the accumulation of intermediates that are toxic. Sensors can respond to these metabolites and reduce the expression of upstream enzymes until the intermediates are cleared.^2477,2479–2481^ *Bacillus subtilis* engineered to make N-acetylglucosamine experienced low yields due to the accumulation of such an intermediate.^2482^ A sensor was built to use a ribozyme that reacts to this intermediate and degrades pathway mRNA in response, thereby decreasing the burden.

##### 3.5.1.3 Resource Allocation in Nutrient-Poor Soil and during Inoculant Storage

Soil can be nutrient poor compared to the rich media conditions where strains are evaluated. Additionally, soil has more environmental fluctuations requiring stress responses and nutrient limitations that require different pathways to be turned on under different conditions.^1956^ There is a trade-off between how cells divide resources between ribosomal synthesis (fast-growing) and synthesis of various metabolic enzymes (more efficient).^2483–2485^ *Escherichia coli* strains with fewer copies of rRNA genes were favored under low nutrient conditions^2486^ and low rRNA copy number is not disadvantageous under native conditions in soil.^1956^ Only under conditions dominated by an abundant substrate led to the favoring of rRNA copy number for fast growth.^2485^ Downregulating the growth rate by reducing RNAP expression in *Escherichia coli* led to increased production of glycerol, indicative of a more efficient metabolism.^2487^ Therefore, tuning down the growth rate by reducing transcription or translation machinery may be beneficial for the performance of a bio-inoculant in the field.

Bio-inoculants often must be stored under conditions that are detrimental to the bacteria. It could be in a consumer package held at room temperature for months, as a liquid formulation, freeze-dried power, or coated seed. The ability for a microbe to stay viable during storage would be beneficial to their activity in the field. Long-term stationary phase cultures of *Escherichia coli* are dominated by those that have gained a growth advantage through mutations in global TFs, amino acid transporters, and RNAP core subunits.^2488–2492^ When stored under nutrient-limiting conditions, various mutations in global regulatory networks governing stress response (RpoS) and metabolism (LRD) have been observed in the soil bacteria *Pseudomonas* and *Xenorhabdus*.^2493,2494^ Transferring these mutations to wild-type strains confers the same growth advantages in nutrient-limited conditions, suggesting that these mutations may be a transferrable genotype to confer advantage in stationary phase.^2488,2489^

#### 3.5.2 Building “Evolution Hard” Chassis

Microbes are under constant evolutionary pressure in the environment. Their genomes, and recombinant genetic systems, change over time due to mutations, homologous recombination, and transposons.^2495,2496^ When these changes impart a growth advantage, they rapidly take over a population.^2496–2498^ While it is not possible to eliminate evolutionary forces completely in a living cell, there are design strategies to reduce the number of mechanisms for mutagenesis and increase the lifetime of the engineered function.^2499^ Note that engineered cells do not have to be robust forever; rather, they only need to stay viable for the time required to impart their function in the field, which differs depending on the application. A microbial insecticide may only have to function for days or weeks whereas microbial nitrogen fixation needs to remain viable for months during a growing season.

##### 3.5.2.1 Blocking Recombination

Homologous recombination is a common mechanism for recombinant DNA to be disrupted. In *Escherichia coli*, if a design has repetitive DNA sequences that share about 20 contiguous base pairs, then homologous recombination is likely to occur.^2500^ In *Bacillus subtilis*, this increases to 70 base pairs.^2501^ This was a big problem early in Synthetic Biology, because many characterized part libraries were based on a common scaffold (*e.g.*, mutants of a promoter core) or there were few alternatives for commonly used parts (*e.g.*, terminators). Thus, designs had repetitive sequences and were prone to disruption due to recombination.

A simple solution is to not reuse parts in a design. To avoid reuse, part libraries have been constructed that have many variations of commonly-needed parts.^303,735,736,808^ Still, in large designs, even if parts are not explicitly reused, then there could be regions of sufficient sequence identity to promote recombination. To address this problem, the EFM (evolutionary failure mode) Calculator scores sequences for stability by searching for repeats that could cause HR and multiple repeats of short sequences that could cause DNA polymerase slippage during replication.^1983^

##### 3.5.2.2 Eliminating Transposons in the Genome

Transposons are another means by which genetic systems can be disrupted. Some transposons have specific sequence preferences for insertion sites. There are various ways in which these sites could inadvertently find their way into a design. For instance, genes are often recoded using codon optimization software to move them into a new organism or to increase expression (Section 2.4.1).^2502,2503^ However, these sequences are rarely screened for the presence of transposon integration sequences. In one case, the codon optimization of nitrogenase genes in *Klebsiella oxytoca* led to the accidental insertion of one transposon insertion site in one gene, which rendered early designs non-functional.^1462^

Evolutionary forces can also indirectly reduce the function of a recombinant system without mutating it. Indirect mutations could reduce flux into a metabolic pathway, reduce protein expression, or change the copy number of a plasmid. This effect occurred in *Escherichia coli* when passaged for 90 hours with a plasmid that imparted a growth defect.^1372^ Over time, it was noticed that cells grew better and the function encoded on the plasmid declined. However, sequencing revealed that the plasmid was unmutated. Subsequently, it was discovered that a transposon mutation to the genome-encoded native *pcnB* reduced the copy number of the ColE1 plasmid, thus reducing the expression of the recombinant genes it carried.^1372^

There are several methods to reduce transposon mutagenesis. First, designs could be scanned for common insertion sequences found in databases such as ISFinder.^2504^ However, insertion sequences are often unknown, particularly for more wild strains. Second, native transposases have been targeted using CRISPRi, which reduced the frequency that recombinant DNA was disrupted, even when burdensome on the cell.^2505^ Third, transposon-related genes can be removed from the genome. This process was completed for *Escherichia coli* to create strains where all mobile DNA elements were removed.^282^ These strains stabilized the genome against insertions and deletions under selective pressure. The genomes of the soil species *Bacillus subtilis*, *Pseudomonas putida, Pseudomonas chlororaphis*, *Pseudomonas chlororaphis, Pseudomonas taiwanensis*, and *Acinetobacter baylyi* have also been reduced, including genes associated with mobile DNA elements, to improve genetic stability (Figure 35) (Section 3.2.4).^291,1898,1962,2506–2511^

**Figure 35:**
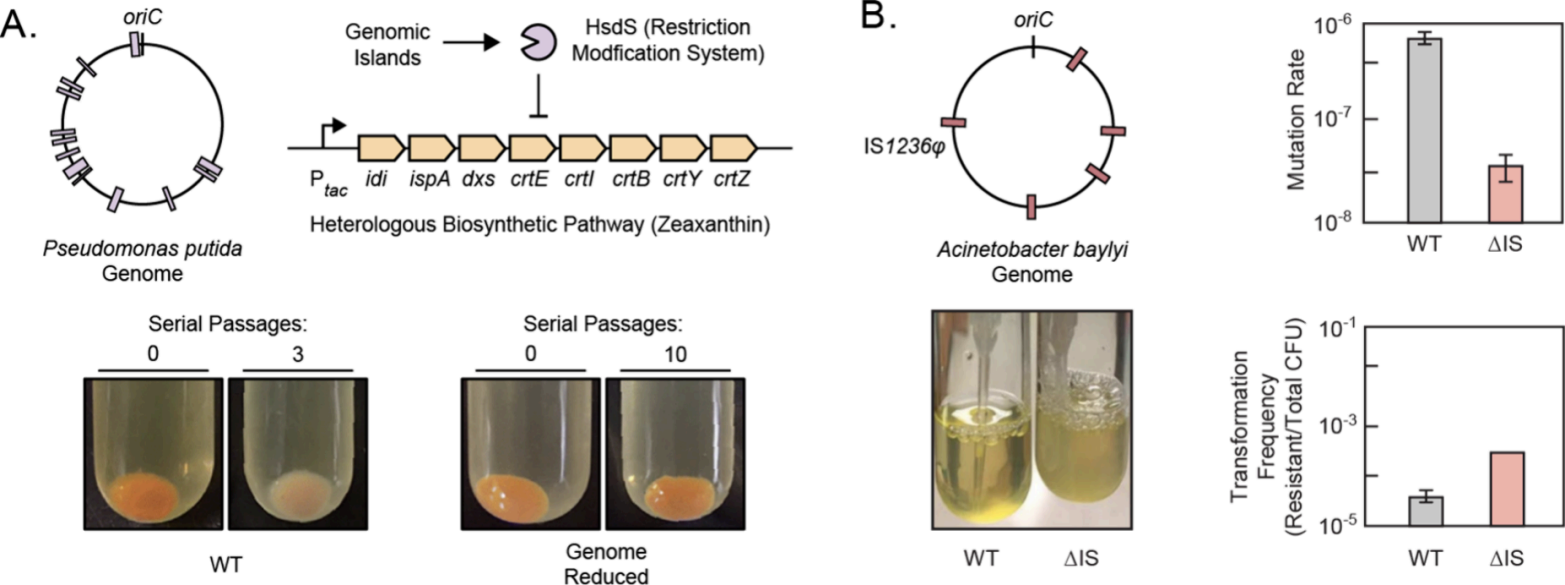
**Improving strain performance and stability by removing mobile DNA elements**. **A**. Deletion of genomic islands encoding horizontal gene transfer sequences improves the stability of a recombinant pathway in *Pseudomonas putida* KT2440.^2507^ Thirteen regions of the *Pseudomonas putida* genome (purple boxes), encompassing 43 genomic islands and 4% of the total genome, were deleted through HR. Wild-type (WT) and genome reduced strains expressing the heterologous zeaxanthin biosynthetic pathway (orange) were serially passaged in culture. The zeaxanthin pathway was deleted from the wild-type after only 3 passages but was still present in the genome reduced strain after 10 passages. Deletion of a single genomic island encoding the HsdS restriction modification system was sufficient to achieve stability improvement (data not shown). Images reproduced with permission from ref 2507. Available under a CC BY license. Copyright 2020, Microbial Cell Factories. **B**. Eliminating Insertion sequence (IS) elements in *Acinetobacter baylyi* ADP1.^2510^ Five genomic regions (red boxes) were deleted in *Acinetobacter baylyi* to remove all six instances of the IS1236 IS element family, including an inactive copy (IS1236φ). The mutation rate was measured through the inactivation of an azidothymidine antibiotic resistance gene, and the transformation rate was measured by counting the number of colonies transformed with a kanamycin resistance marker. The genome-reduced strain (ΔIS) had reduced settling behavior compared to the wild-type strain (WT) in saturated cultures after 48 h. Image reproduced with permission from ref 2510. Copyright 2017, Applied and Environmental Microbiology.

##### 3.5.2.3 Linking a Synthetic Function to Cell Survival

Stability can also be improved by “entangling” two genes. Entanglement refers to the encoding of an entire gene within the open reading frame of another gene, but in a different translational frame. This organization is a common motif in natural genetics, particularly in bacteriophage genomes.

To stabilize a recombinant gene, it can be entangled with a native essential gene that has been deleted form the genome.^2512^ If the recombinant gene is lost, then the essential gene is lost as well and the cells will die. Computational methods have been developed to aid the design of entangled genes through codon selection and register shifting the gene orientations. In an entangled system, 13% of mutations within the entangled recombinant gene led to a >10-fold decrease in growth rate and were thus selected against. The entangled gene was stable for over 150 generations, whereas the unentangled gene was disrupted after only 50.^2512^

##### 3.5.2.4 Selection of Spore-Forming Species

Species that form spores have natural mechanisms to preserve genetic integrity while persisting for long times in the environment. Spores form a hard shell that protects against radiation, UV, desiccation, and many other stresses.^2513^ It has also shown to preserve the integrity of the encoded DNA sequence, including recombinant genes that are being carried.^2514^ The spore-forming strain *Bacillus frigotolerans* was engineered to express a recombinant gene.^1879^ This strain was mixed with dirt and incubated under ambient, non-sterile conditions for two years. Isolates retained the ability to express GFP. Furthermore, the recombinant gene did not mutate after this extended period of incubation. Indeed, only six mutations occurred throughout the genome.

Spore formation machinery can be manipulated to alter a strain’s persistence in the environment. Placing the *Streptomyces* sporulation regulator WblA under the control of a sensor made sporulation inducible, whereas its deletion abolished sporulation altogether.^2515^ Bacterial and fungal spores engineered to contain a unique nucleotide barcode can be used in agricultural supply chains for tracking and origin determination.^2516,2517^ For this purpose, *Bacillus subtilis* spores can be made unable to germinate by deleting receptors and cell wall lytic enzymes.^2516^

#### 3.5.3 Design to Block Horizontal Gene Transfer

Bacteria continuously exchange DNA to and from the environment.^2518^ Sometimes, it is taken up an incorporated into the genome of the new host, in a process known as horizontal gene transfer (HGT). This transfer is a frequent event in soil and rhizosphere.^2518–2520^ Some soil bacteria have mechanisms to actively import external DNA into their cytoplasm, including *Bacillus subtilis, Acinetobacter calcoaceticus, Acinetobacter baylyi, Azotobacter vinelandii*, and *Pseudomonas stutzeri*.^2518,2521–2531^ The rate of transfer is high on plant surfaces; while 10^–8^ to 10^–5^ transconjugants per donor were obtained in bulk soil, frequencies up to 0.1 were measured on root surfaces.^2529,2530^

DNA can also be spread between bacteria by phages.^2532–2535^ Phage transfer in contaminated soil led to the proliferation of genes involved in arsenic detoxification.^2533^ The chances of HGT are increased when the transferred genes confer a growth advantage. An example of this occurred when an engineered phenol-degrading *Pseudomonas putida* strain was released into the environment. The recombinant plasmid encoding the phenol degrading genes could be found in at least nine Pseudomonad isolates six years later.^2536^

Interkingdom DNA transfer from the plant to its resident bacteria can occur. For example, a recombinant antibiotic resistance gene was found to transfer from engineered tobacco to *Acinetobacter* and *Ralstonia* species.^2537^ The likelihood that these transfer events will lead to the functional expression of the gene are much lower because the signals for transcription and translation are so divergent between eukaryotes and prokaryotes.

##### 3.5.3.1 Measuring Horizontal Gene Transfer

Tools have been developed to quantify the extent of horizontal gene transfer in an environment. Traditionally, this has required the direct measurement of DNA by PCR or the use of antibiotic markers or fluorescent reporters. A reporter enzyme that allows the detection of DNA transfer without recovering the bacteria is MHT (Section 3.4.4), which produces a gas that can be measured in a soil sample by GC-MS.^2538^ The rate of transfer was quantified in soil by introducing a donor strain encoding the MHT gene on a conjugative plasmid.

HGT can also be measured using CRISPR machinery (Section 3.2.1). The spacer acquisition proteins (Cas1 and Cas2) integrate short DNA sequences into CRISPR arrays that are later transcribed and processed into RNA that guides Cas9 to its targets.^2539^ When expressed in *Escherichia coli*, these genes record imported DNA sequences into an array (Figure 36A).^2540^ After recording the DNA transfer events, the bacteria can be recovered and sequenced to obtain the transfer history. This system was used to track the movement of non-replicative plasmids and mobile genetic elements in fecal material.^2540^

**Figure 36:**
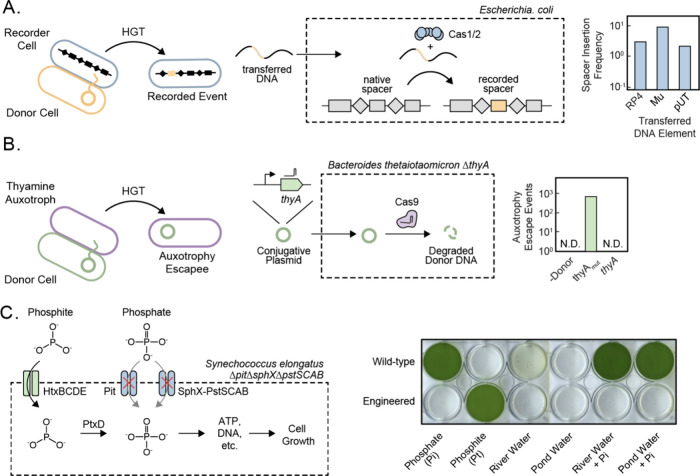
**Strategies to limit horizontal gene transfer**. **A**. Recording transfer events using CRISPR.^2540^ A Recorder cell expressing the spacer acquisition proteins Cas1/Cas2 from a plasmid was incubated with cells carrying one of three mobile DNA elements (the RP4 conjugative plasmid, phage Mu, and plasmids pUT). The “spacer insertion frequency” was measured as the number of spacers recorded per 1000 native spacers, normalized to the size of the transferred DNA element. **B**. Preventing gene transfer into a bacterium.^2548^ *Bacteroides thetaiotaomicron* was engineered to be auxotrophic for thiamine, which could be rescued through the import of foreign DNA containing *thyA* in the form of a conjugative donor plasmid. The import was disrupted by including a Cas9 cassette that targets *thyA* (thymidylate synthase). A variant of *thyA* (*thyA*_mut_) contained mutations to the gRNA binding site and was resistant to Cas9 targeting. Auxotrophy escapees were counted on media containing an antibiotic co-delivered on the conjugative plasmid. **C**. Engineering synthetic auxotrophy to prevent environmental release.^2555^ Phosphate importers were deleted from cyanobacteria *Synechococcus elongatus*, which was then engineered to import and utilize phosphite as a sole phosphorous source. The wild-type and engineered (Δ*pst* Δ*pit* +*htxBCDE* +*ptxD*) strains were grown in minimal medias and environmental water sources supplemented with various phosphorous sources. Images of cell cultures reproduced with permission from ref 2555. Copyright 2018, American Chemical Society.

##### 3.5.3.2 Designing for Reduced Horizontal Gene Transfer

Engineering strategies to reduce the frequency of HGT have been developed. These methods work either directly by stopping entry or integration or by making it unreadable by the receiver species. One approach is to disrupt native genes known to facilitate HGT. The deletion of genes that impact competence decreased the rate of escape from a phosphate auxotrophy and plasmid mobility.^2541–2545^ Placing conjugation genes under arabinose-inducible control led to a 3000-fold reduction in HGT.^2546^ The activity of conjugation proteins can also be inhibited by expressing recombinant antibodies, as demonstrated with a relaxase that initiates and terminates DNA transfer.^2547^

Another approach is to harness the natural function of CRISPR machinery, which is to block foreign DNA to enter the cell. An engineered human gut commensal *Bacteroides thetaiotaomicron* strain was made auxotrophic for thymidylate to improve biocontainment. However, *thyA* transfer from the natural microbiota led to a high rate of escape. Introducing a CRISPR system targeting the wild-type allele *thyA* into the engineered strain destroyed the gene if it entered the cell, thus preventing escape (Figure 36B).^2548^

The introduction of a recombinant strain into the environment also has the potential to have its DNA transferred to native species. Plasmids can be easily transferred between hosts. One way to reduce plasmid transfer is to move the replication genes from the plasmid to the genome, so that the plasmid can only replicate in that host.^1842^ Plasmids can also be prevented from spreading by encoding a toxin gene on the plasmid and then encoding the antitoxin gene in the host genome.^1842^ If the plasmid transfers, then it would kill the new host. Gene entanglement (Section 3.4.2) can also be used to block gene transfer. If there is a gene-of-interest that encodes a function that one does not want to be transferred, it can be entangled with a toxin gene and the anti-toxin gene encoded far away in the genome.^2512^ This strategy reduced the rate of transfer of the recombinant gene by 3000-fold.

One way to reduce the likelihood of gene transfer is to degrade the recombinant or genomic DNA after the function has been performed in the field. CRISPR nucleases can be targeted to the genome of the strain or synthetic DNA to degrade it before it can be taken up by native bacteria in the microbiota. By targeting Cas9 to plasmids or regions of the genome, up to 10^8^-fold less DNA was present after cell death.^2549^ Similarly, the frequency of a recombinant plasmid was reduced >10^8^-fold following the induction of a Cas3 nuclease targeted to a plasmid.^2549^

The ability to *de novo* synthesize whole bacterial genomes (Section 3.2.3) has led to radical re-designs to block horizontal DNA transfer. Genome-scale changes make it impossible for the host DNA to be read by other cells in the environment and vice versa for environmental DNA to be read in the host. These changes involve comprehensive alterations in the codon usage or parts that only work in the host (*e.g.*, RBS sequences that require a synthetic rRNA). A recoded *Escherichia coli* strain was built using MAGE that removed all instances of the UCG/UCA serine codons, as well the UAG stop codon, by replacing them with alterative serine/stop codons.^298^ The UCG/UCA serine codon were reassigned to encode alanine, histidine, leucine, or proline codons by expressing chimeric tRNAs engineered to recognize UCG/UCA.^2550,2551^ Genes that transferred into this host that contained these codons would put the newly encoded amino acid where serine should be, thus causing them to misfold and lose function. When a gene was transferred from the “recoded” *Escherichia coli* to a new host, a serine would be translated in place of the correct alanine, histidine, leucine, or proline causing misfolding and preventing HGT. Similar approaches can block phage infections, which can facilitate HGT (Section 3.5.2).

Orthogonal ribosomes (o-ribosomes) recognize alternative RBSs by mutating the 16S rRNA sequence that binds the SD sequence.^2552^ Recombinant genes downstream of these alternative RBS sequences can only be expressed in host strains containing the o-ribosome and are nonfunctional in wild-type microbes.^2553,2554^ When the strain does not have the o-ribosome rRNA, there is a >99% reduction in expression of genes with this SD.^2553^

#### 3.5.4 Engineering Phage Resistance

Phage are viruses that infect bacteria.^2556^ In rich agricultural soil, there are up to 10^10^ phage/g soil.^2557^ Phage play a major role in shaping the soil microbiome.^2558–2560^ They can drive HGT between bacteria.^2561,2562^ They can reduce the abundance of beneficial microbes or impair their function upon release in the field.^2563,2564^ For example, the ability of a bio-control strain based on *Pseudomonas fluorescens* to protect cucumber plants from infection was reduced by natural bacteriophage ΦGP100.^2563^ Therefore, phage pose a barrier to the use of GMMs for agricultural applications. Hence, there have been efforts to create “phage hard” chassis that can resist infection.

Bacteria have evolved diverse phage resistance mechanisms that either prevent virus binding and entry or target the viral genome once it has entered the cell.^2565^ Bacteria can be engineered or evolved in the lab in several ways to reduce phage adsorption to their surface. These include altering, disguising, modifying, or masking receptors and the use of decoy outer membrane structures. For example, mutations in the *Vibrio cholerae* OmpU receptor increases phage resistance.^2566^ The expression of decoy receptors that bind phage acts as a sponge to decrease productive infections.^2566^

Steps can also be taken to block the injection of the phage genome into a bacterium. Restriction-modification systems are a mechanism used by bacteria to destroy foreign DNA from different exogenous sources. Modification enzymes chemically modify DNA sequence motifs, marking them as “self,” and restriction enzymes cut the motif if unmodified. Because foreign DNA is unmodified, it will be degraded when it is taken up by the cell. Transferring heterologous restriction-modification systems into a host improves its phage resistance. For example, transferring the restriction-modification system DISARM (Defense Island System Associated with Restriction–Modification) from *Bacillus paralicheniformis* 9945a into *Bacillus subtilis* BEST7003 conferred resistance to seven phage across three families.^1764^

Sometimes referred to as the bacterial “immune system,” CRISPR arrays encoded in the genome are used by bacteria to defend against phage.^1765^ CRISPR enables bacteria to “remember” infections by integrating short sequences from the phage genome into the CRISPR array. The array is transcribed and processed into gRNAs that direct Cas nucleases to cut matching DNA sequences.^2567^ Bacteria with CRISPR systems can be engineered to resist particular phage by transforming phage-derived DNA sequences so that they are processed and added to the CRISPR array.^2568^

Transferring a CRISPR system into a new host protects it from the phage whose DNA were encoded in the array.^2569^ One was transferred into *Bacillus subtilis* and reduced susceptibility to the phage DNA in the array by 10^6^-fold.^2570^ It is also possible modify the native CRISPR systems in the strain-of-interest. Inserting spacer sequences into a native CRISPR array resulted in resistance to the phage.^2571^ Note that the number of potential phages in the environment is much larger than can be protected against in an engineered CRISPR array. Metagenomic sequencing suggests the average bacterium contains 20-40 spacers and the largest number of spacers in a natural host is 587 in *Haliangium ochraceum*.^2572,2573^

*De novo* genome synthesis (Sections 3.2.3.4 and 3.5.3.2) offers an opportunity to design bacteria that are immune to phage.^284^ Codons can be removed or reassigned throughout the genome *en mass*, causing any viral genes that enter the cell to be mistranslated. This whole genome replacement strategy has only been accomplished in *Escherichia coli*.^284,298,1934,1949,2550,2574–2576^ A genetically-recoded *E. coli* strain was engineered by replacing all instances of the amber stop codon (UAG) with the synonymous UAA codon, as well as knock out the UAG release factor 1. The UCG and UCA serine codons were then swapped with the synonymous AGC and AGU serine codons, as well as eliminating the genes encoding tRNA^Ser^_UGA_ and tRNA^Ser^_CGA_. The final strain contained 18,214 recoded codons and 482 additional mutations resulting from adaptive laboratory evolution to recover growth rate. Phage transcripts containing the recoded amber or sense codons would cause ribosome stalling and halt phage infection. This strain was resistant to a cocktail of phages (λ, P1vir, T4, T6, T7).^2574^

However, it was shown that on rare occasions, viruses can overcome this genetic recoding strategy by carrying tRNAs to limit dependence on host translation machinery (up to 27 have been observed). Expressing chimeric tRNAs that encoded the missing UCG/UCA serine anticodons, but carried alanine, histidine, leucine, or proline resulted in the mistranslation of serine codons as the swapped amino acid and a reduction in phage replication (Figure 37).^2550^ There is an ongoing effort to build a 57-codon *E. coli* strain, with the UAG (stop), AGG (Arg), AGA (Arg), AGC (Ser), AGU (Ser), UUG (Leu) and UUA (Leu) codons replaced, which should be resistant to all known coliphages^2577,2578^

**Figure 37:**
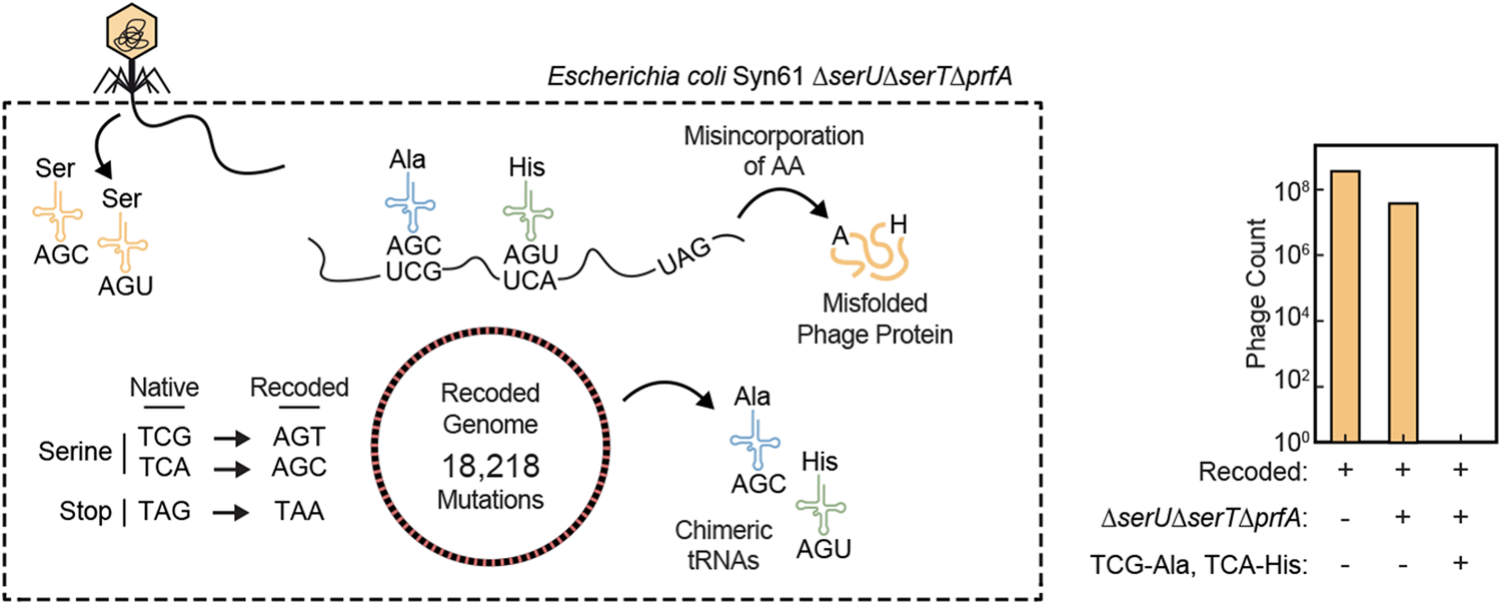
**Genome engineering to harden bacteria against phage**. A genome-recoded *Escherichia coli* replacing the amber codon and two serine codons (*Escherichia coli* Syn61) was further engineered by deleting the release factor 1(*prfA*) and two serine tRNAs (*serU* and *serT*) and evolved through adaptive laboratory evolution.^2551^ The strain then expressed chimeric tRNAs encoding the missing serine codons, but carrying alternative amino acids (alanine, histidine, leucine, and proline). Phage resistance was measured using plaque counts when plated onto agar containing the recoded *Escherichia coli* (Syn61), recoded *Escherichia coli* with tRNA deletions (Syn61Δp*rfA*Δ*serU*Δ*serT*), or recoded *Escherichia coli* carrying chimeric tRNAs (Syn61ΔprfAΔserUΔserT + TCG-Ala/TCA-His).

#### 3.5.5 Safety Switches

When an engineered microbe is released into the field, it is desirable to be able to control its activity in space and time.^1673,2579,2580^ Temporal control would program the loss of the microbe in the environment after a season, a number of days has passed, or after a preprogrammed number of cell divisions. Microbes could also be controlled with respect to space, where if they exit a defined area or proximity to a plant, they die. To implement this control, “safety switches” (also referred to in the literature as “kill switches”) have been designed to be carried in the genome.

Auxotrophy containment connects cell division to the presence of a small molecule. Therapeutic gut bacteria, where the deletion of *dapA* rendered *Escherichia coli* unable to growth without an external source of DAP.^2581,2582^ DAP could be fed to the patient, like a drug, keeping the bacteria viable in the body. After the patient stops consuming DAP, or if the bacteria are released into the environment, the bacteria cannot continue to replicate. A similar approach was taken to make the cyanobacterium *Synechococcus elongatus* PCC7942 require phosphite for growth (Figure 36C).^2555,2583^ Two endogenous phosphate importers were deleted to prevent cell growth on phosphate as a sole P source. The strain then engineered to have a phosphite importer and an enzyme that reduces phosphite to phosphate, allowing for the uptake and incorporation of P. Phosphite is rare in soil and needs to be spread over the desired area to promote the growth of the engineered bacteria. Auxotrophy-based safety switches can be broken when the genes required for replication are acquired from the environment. The use of multiple auxotrophies reduces this effect by making cells dependent on more than one small molecule for growth.^2584^

Sometimes, it is difficult to find a unique and required molecule that is practical to spread over an area for microbial containment. An alternative is to connect a genetic sensor for a molecule to an antitoxin that inactivates a constitutively-expressed toxin. When the bacteria leave the contained area, the molecule is not present, the antitoxin stops being made, and the cells die. For example, a safety switch in *Pseudomonas fluorescens* used an IPTG sensor to control an antitoxin so that the cells die in the absence of IPTG.^2585^ The signal does not have to be a small molecule; safety switches have been made using sensors that respond to pH and temperature.^2586–2588^ This approach was taken to build a containment system for the soil bacterium *Acinetobacter sp*. Tol5 engineered to remediate toluene (Figure 38).^2589^ An essential protein (BapA) was placed under the control of a toluene sensor so that the bacteria stopped dividing after exiting an environment containing toluene.

**Figure 38:**
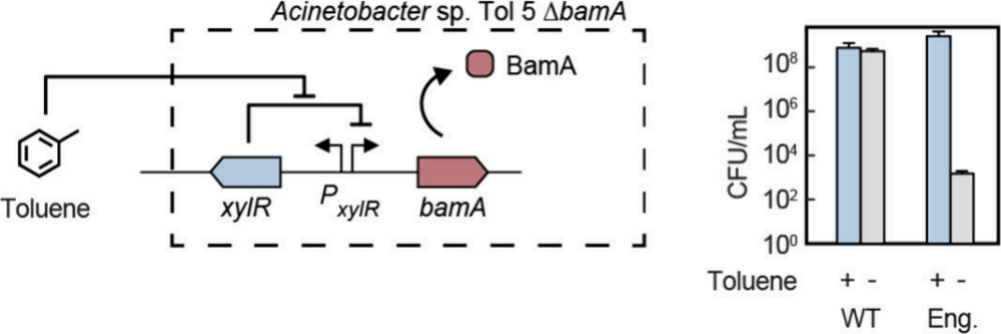
**Biocontainment via a small molecule switch.** ^2589^ This Acinetobacter strain was developed for the bio-remediation of the pollutant toluene. The essential *bapA* was knocked out and placed under the control of a toluene-responsive promoter (Engin.) and compared with wild-type (WT). Cell growth was measured after 3 days in media with and without 0.05% (vol/vol) toluene. WT: wild-type.

Integrating multiple signals leads to higher specificity in defining an environment. Logic was used to build a safety switch for *Escherichia coli Nissle* designed to perform a therapeutic function in the mouse gut.^2590^ It was desired that cells die after exiting the animal. To sense that they were no longer in the body, an OR gate was designed to react to the absence of a chemical fed to the animal in the food or when the temperature drops below 32°C. Similarly, the “Passcode” switch uses a logic circuit to identify the environment by integrating signals to keep the switch off with high galactose and cellobiose and no IPTG.^2591^ Outside this environment, the switch flips and a pair of toxin genes are expressed, causing a 10^7^-fold decline in viable cells after only 6 hours. Using more functionally-distinct toxins reduces the prevalence of escape mutants.^2592^ The reliability of a safety switch can be improved by using multilayered regulatory control at both the transcriptional and translational levels of the essential or toxin gene to reduce the likelihood of breakage by mutations.^2592^ Note, however, that the use of more complex regulatory control also increases the ways mutations can lead to escapees.

Synthetic autotrophies can be introduced by making cells require a non-natural amino acid to grow. The re-coding of the bacterial genome using MAGE can be used as a platform to make the species dependent on a synthetic amino acid for growth.^2575,2593^ If a codon is freed, then a new tRNA can be introduced that incorporates an unnatural amino acid not found in nature. If this amino acid is not provided, then the proteins that use it are not made and the cells do not grow. This approach was taken for three essential genes in *Escherichia coli*, which led to a drop in escapee frequencies to less than the detection limit and no escapees appeared after a week of growth.^2576^

Timers have been constructed, where cells die after a defined number of cell divisions once a signal is removed. A timer was built for *Salmonella* by knocking out two essential genes and placing them under arabinose-inducible control.^2594–2596^ When cells are grown in the presence of arabinose, the essential proteins are produced. But when the cells are moved to an environment without arabinose, the essential proteins dilute by half with every cell division and eventually cross a threshold where they can no longer divide. The constraint on these timers is that there is little control over the length of time or number of cell divisions before the bacteria die. For applications in agriculture, one would want to be able to tune the length of time bacteria survive in the field from days to months (a season).

#### 3.5.6 Synthetic Communities (SynComs)

Using a community of species, rather than just one, can impart several advantages.^2597^ First, multiple functions can be combined into a product without having to stack them in a single species, such as nutrient scavenging and growth promotion. Second, consortia may have advantages in colonization a surface, such as a leaf or root, and stopping the invasion of other species.^2598^ Finally, stability against environmental fluctuations can be improved by engineering linkages between the species, such as metabolic interdependencies.

High-throughput screens can identify artificial combinations of natural bacteria that enhance a function-of-interest carried by one. A droplet assay encapsulated 19 soil isolates and 13 carbon sources to create 100,000 multispecies communities.^2599^ These were screened to identify the combination that supported the highest concentration of *Herbaspirillum frisingense*, a plant-growth-promoting bacterium. Similarly, species from the maize rhizosphere were combined to create a representative seven-species consortium to colonize maize roots.^2600^ Changes to the consortium composition due to the removal of species pointed to the keystone species essential the consortium stability and protection against invasion by plant pathogens. Similar synthetic communities (SynComs) have been identified to aid resistance against other pathogens, increase survival under drought stress, inhibit fungal growth, serve as biosensors, promote plant growth, and improve soil quality for various crops, including cucumber, cabbage, maize and other crops.^2597,2601–2616^ Typically, these communities consist of designed combinations of natural bacteria, rather than incorporating engineered variants.

Cooperation and competition between strains is important for a community to colonize a plant and remain stable despite environmental fluctuations.^2617^ Stability refers to the species surviving and maintaining their relative ratios. Environmental fluctuations could be due to changes in nutrient availability, ambient conditions, or invading organisms. A common cooperation strategy is cross-feeding, where members of the community display complementary autotrophies and require other members to produce the essential nutrient. Cooperative interactions are stronger when the exchanged nutrients are metabolically costly to biosynthesize.^2618^ Cross-feeding communities, where each member feeds the other, can be highly resistant to environmental fluctuations. An *Escherichia coli* and *Acinetobacter baylyi* community that exchanged gluconate and acetate returned to consistent equilibrium ratios after changes to substrate availability or initial culture conditions.^2619^ Cross-feeding between an ammonia-overproducing *Rhodopseudomonas palustris* and an organic acid-secreting *Escherichia coli* was stable over 30 generations and converged to a constant ratio even when the starting ratios spanned 12 orders-of-magnitude.^2620^ The cross-feeding circuits can be tuned to obtain different equilibrium strain ratios.^2620,2621^

Interactions between consortium members can also be antagonistic, where members create a stable community by competing for shared resources or producing toxic metabolites targeting a species. While seemingly contradictory, antagonistic interactions can aid stability by implementing feedback to stop some strains from dominating the population. Antimicrobial peptides have been used to establish interactions between *Lactococcus lactis* strains.^2622^ This system was tuned to different community structures, including amensalism (strain A inhibits strain B), competition (strain A and strain B inhibit each other), and predation (stain A inhibits strain B and strain B benefits A). A challenge with antimicrobial peptides is that they can have a non-specific effect that is detrimental to the natural microbiota.

An alternative approach is to place a toxic gene under the control of an inducer. In effect, this makes the inducer molecule an antimicrobial only to the intended engineered strain. A quorum signal sensor has been connected to the expression of an intracellular protein that is toxic to the bacterium so that one species can downregulate another without producing an antimicrobial.^2623^ A quorum signalling circuit controlling the expression of lysis proteins in two *Escherichia coli* strains with different growth rates was used to achieve a stable population ratio.^2294^

#### 3.5.7 In Situ Transformation of Native Microbiota

Engineering a microbe to persist in the rhizosphere or colonize a plant surface can be difficult because the native microbiota blocks initial infection and growth. This challenge has limited the development of therapeutic bacteria that need to persist in the human gut to impart their effect.^2624–2626^ Instead of trying to introduce a living inoculant, the DNA payload encoding the desired function can be directly transformed into the natural community already in residence. To this end, tools for *in situ* genetic engineering have been developed.

Conjugative systems have been adapted to deliver DNA payloads *in situ* from a donor strain to microbial communities in soil and animal microbiomes.^2627,2628^ In soil, a *Pseudomonas putida* donor transferred DNA to Peudomonads in a complex microbial community for a month.^2627^ For a bioremediation experiment, rather than inoculating poplar trees, *Burkholderia cepacia* was used to transfer a plasmid containing a toluene degradation pathway to natural poplar endophytes.^2629^ To remediate petroleum from soil, *Escherichia coli* donors were used to transfer degradation pathways to native bacteria.^2630^ After two months, the donor was undetectable whereas the pathway continued to propagate in native species. Similarly, to clean up phenol pollutants, an engineered *Pseudomonas putida* strain containing a degradation pathway on a plasmid was introduced in the environment.^2459^ Seven years later, the inoculant was undetectable but the pathway persisted in other species due to HGT.^2459^

The transfer of recombinant genes to a native community could be advantageous for the long-term maintenance of the functional DNA payload in the environment, but could also pose challenges for the uncontrolled spread of recombinant DNA. Therefore, it would be useful to knock out genes to prevent the continued transfer of recombinant DNA. This approach was taken to control a conjugative system based on the *Bacillus subtilis* integrative and conjugative element 1 (ICE).^887^ ICE has been shown to have a wide species range and high conjugation efficiency and can transfer a DNA payload into multiple species residing in the soil. To reduce the possibility for the continued transfer of the DNA payload, the secretion system needed to transfer DNA between cells was separated from the DNA payload to be transferred. In addition, the *Bacillus subtilis* donor strain was engineered to contain an auxotrophy so that it cannot propagate once introduced into the environment. This system, called XPORT (Section 3.2.3.1), was shown to transfer a DNA payload into multiple soil strains residing in artificial soil (Figure 27).

An approach that encourages propagation is MAGIC, a DNA conjugation method capable of transferring DNA payloads into both Gram-negative and Gram-positive species *in situ*.^2628^ The *Escherichia coli* donor was made auxotrophic to ensure that it cannot persist once released, but the introduced DNA can continue to transfer between bacteria in the environment. It transfers a broad-host range plasmid that contains a transposase to integrate the DNA payload into the recipient's genome. The MAGIC donor containing a DNA payload was fed to a mouse and directly transformed resident bacterial communities in its gut. It was shown to be able to conjugate into members of the Proteobacteria, Firmicutes, Actinobacteria, Fusobacteria, and Bacteroidetes phyla while residing in the gut.

The *in situ* delivery of the DNA payload can be targeted to specific species and specific genomic sites. An engineered phage encoding dCas9 and targeting sgRNA was used to deliver payload DNA specifically to *Escherichia coli* residing in a mouse gut.^2631^ Within a complex community, CRISPR-guided transposases (Section 3.2.1) can be targeted to specific species and genomic locations.^1922^ This strategy was used to disrupt a native gene in the soil strains *Klebsiella michiganensis* and *Pseudomonas simiae*.

### 3.6 Accelerating the DBTL Cycle for Agricultural Bacteria

As a field, Synthetic Biology has focused on accelerating the engineering of biology, often articulated as the Design-Build-Test-Learn (DBTL) cycle.^1985,2632^ In Section 2.4.3, it was described with respect to plants, where the slow steps are usually transformation and growth, leading to intrinsically slow cycles. For microbes, which can grow to saturation in hours, the cycle time is order-of-magnitude more rapid. Methods for model organisms, such as *Escherichia coli* and *Saccharomyces cerevisiae*, have been developed specifically to automate the design process to make many variations of pathways or genomes that can then be screened at high-throughput, producing the datasets required to create AI/ML models to drive the next round of design.^2633^ So-called “BioFoundries” have emerged in industry and academia that have robotic automation pipelines for strain construction and software needs to guide the processes.^2634,2635^ These can be used to drive the cycle time down to weeks or even days. Reviews have been written about these technologies;^1426,1440,2636,2637^ here, we focus on their application to soil bacteria or pathways of agricultural relevance.

#### 3.6.1 Computer Aided Design (CAD) for Microbes

CAD has become a crucial driver of genetic engineering projects. The software can be as simple as plasmid/genome editors and databases of genetic parts to aid the selection of genetic parts. There are various tools for biomolecular design, metabolic engineering, and genetic circuit design. Increasingly, AI and ML aid with managing the complexity of the systems. They can inform design choices based on large sets of screening data. Generative AI is just beginning to automatically compose the sequences of proteins, genetic systems, plasmids, and even whole genomes.^1772,2638,2639^ The challenge remains that the deluge of tools available are one-off software packages, and a complex project requires the manual passaging of information between tools. Here, we review these tools from the perspective of engineering agriculturally-relevant bacteria.

##### 3.6.1.1 Software to Accelerate the Design of Genetic Systems and Genomes

The simplest software environments provide a means to store genetic parts and visualize designs and some can connect to simulators (genetic circuit dynamics, metabolic flux, etc.).^1442,2640–2647^ Databases for plasmid backbones and genetic parts for soil bacteria are listed in Table 2. To date, the databases are over-populated with parts for model organisms. As of 2023, the Registry of Standard Biological Parts had 47,000 entries.^2648–2650^ However, for soil microbes, there were only 300 for Pseudomonads, 200 for *Bacillus subtilis*, and less than 10 for *Klebsiella* and *Streptomyces*, respectively. There are some parts databases that encompass taxa relevant to agriculture, including *Bacillus subtilis* (SubtiWiki^2651^ and DBTBS^2652^), Pseudomonads (PseudoCAP^2653^) and Streptomyces (StreptoBase^2654^). Note that these part databases only contain the sequences and not the functional characterization necessary to inform CAD software.

Synthetic Biology has strived to make parts models easily shared between groups. Several standards have been adopted as a result. The Systems Biology Markup Language (SBML) is a formal language for representing a mathematical model of a biological system.^2655,2656^ It allows the model to be simulated across different simulation platforms. The synthetic biology open language (SBOL) standardizes genetic part sequences along with their functional information (and visual glyphs using SBOLv).^2657–2661^ The CAD software iBioSim brings all of this together allowing a user to combine parts and parameterize and simulate the system performance using models.^2657^

###### 3.6.1.1.1 Mining and Design of Genetic Parts

Parts can be gleaned from genomes. Computational tools would scan genomes looking for signatures of a part. For example, promoters could be identified using mathematical models of RNAP binding to the DNA or bioinformatics/ML looking for sequence signatures. This software could be used to stock a part library or identify promoters that could serve as sensors by turning on under a particular set of conditions (Section 3.2.6). Promoter prediction algorithms have been used for soil species, including *Cyanobacteria*, *Pseudomonads*, *Bacilli*, and *Streptomyces*.^2662–2665^ Libraries of natural terminators can also be gleaned from genomes using TransTermHP,^2666^ ARNold,^2667^ and RNIE.^2668^ They have been applied to the soil bacteria *Pseudomonads, Bacilli, Streptomyces*, and *Acinetobacter*.^2666,2668–2670^

*De novo* part design tools use an underlying biophysical model to design part sequences.^1447^ The RBS Calculator can design the sequence of an RBS to obtain a user-defined target level of expression.^1448,2671^ Theoretically, it can be used in any host where the 16S rRNA sequence is known, although there are differences in other binding factors and spacing between the SD and start sites that, when unknown, reduce its accuracy. It has been used to design RBSs for soil species, including *Pseudomonads*, *Streptomyces*, *Bacilli*, and *Rhizobia*.^2014,2027,2057,2672^ Additional related computational tools are available to create RBS libraries, operons, and promoters.^1450,2673,2674^

###### 3.6.1.1.2 Codon Optimization

Codon optimization tools aid the transfer of genes between species and can improve expression within the source species. The algorithms need to have the codon usage table for the species.^2675^ Many commercial tools can optimize genes for soil bacteria, including *Pseudomonas*, *Bacillus*, *Streptomyces*, and *Klebsiella*.^2676–2678^ Codon optimization has had a key role in strain engineering of soil bacteria. *Pseudomonas putida* KT2440 was engineered to consume lignin in part through the codon optimization of a three-gene pathway.^2679^ *Streptomyces lividans* was engineered to secrete greater quantities of a recombinant enzyme by replacing rare codons.^2680^

Codon optimization does not just increase expression by eliminating codons that are rare or not present in the host. Rather, it also serves to eliminate internal regulatory sequences, such as promoters, that can reduce expression.^1462^ This effect can increase expression even when the gene is being expressed in the same host.

###### 3.6.1.1.3 Genome Editing

Software can also design genome editing machinery to target particular sequences while avoiding off-target insertions. For bacteria, these tools are for the design of gRNAs to guide Cas9 to the target while avoiding off-target sequences in the genome.^2681^ The web-based tools CHOPCHOP, CRISPOR and CRISPRscan enable users to design gRNAs for common soil bacteria and provide the option to upload a reference genome.^112,2682–2684^ Tools for more specialized CRISPR-based applications also exist, such CRISPR-ERA (CRISPRi/CRISPRa) and CRISPR library designer (CRISPR screens).^2685,2686^

Software tools have also been developed for whole genome engineering. MAGE requires degenerate oligonucleotide pools to create thousands of different genome variants (Section 3.2.1).^1858,2687,2688^ MAGE Oligo Design Tool (MODEST) suggests optimized MAGE oligos that perform a user-defined list of genome modifications.^2689^ These could encompass RBS libraries or codon changes distributed throughout the genome. The oligos are designed to avoid secondary structure and shared sequence identify with off-target locations in the genome.

##### 3.6.1.2 Predictive Metabolic Engineering

Metabolic modelling seeks to predict genomic changes that will increase the titer of a desired product.^2690,2691^ For the optimization of agricultural strains, the objective could be to maximize the production of a phytohormone, nutrient, or antimicrobial. To optimize a product, the software predicts which enzymes can be knocked out to redirect the carbon flux to the precursor of the product of interest.

Genome-scale metabolic models (GEMs) capture a complete metabolic map of an organism in the form of a stochiometric matrix that enumerates reactions and metabolites.^2692^ Each species needs its own GEM and many are available in the BiGG and ModelSEED databases.^2693,2694^ GEMs developed for species of soil bacteria are listed in Table 3. There are software packages to aid the creation of GEMs for species for which one is not yet available, requiring only the genome sequence.^2695–2700^ MEMOTE is an automated tool that uses the standard SBML language to assess the quality of GEMs, such as the presence of blocked reactions (meaning they are predicted to have no flux) or incorrect stoichiometries.^2701^

**Table 3:** Genome-Scale Metabolic Models in Soil Bacteria

Species Name	Metabolite Number	Reaction Number	Model ID	Ref
*Bacillus subtilis*	988	1020	Model v3	^2714^
*Bacillus subtilis*	1138	1437	iBsu1103	^2715^
*Bacillus subtilis*	1456	1742	iBsu1147	^2716^
*Bacillus subtilis*	1103	1955	iBsu1144	^2717^
*Bacillus subtilis*	1291	1577	iBB1018	^2718^
*Bacillus megaterium*	1349	1709	iJA1121	^2719^
*Bacillus megaterium*	993	1112	iMZ1055	^2720^
*Bacillus licheniformis*	1141	1762	iWX1009	^2721^
*Pseudomonas putida*	911	950	iJN746	^2706^
*Pseudomonas putida*	886	877	iJP815	^2722^
*Pseudomonas putida*	1044	1071	PpuMBEL1071	^2723^
*Pseudomonas putida*	1104	1171	PpuQY1140	^2724^
*Pseudomonas putida*	2155	2929	iJN1462	^2725^
*Pseudomonas fluorescens*	1734	1721	iCW1057	^2726^
*Pseudomonas stutzeri*	813	1135	iPB890	^2727^
*Pseudomonas aeruginosa*		883	iMO1056	^2728^
*Paenarthrobacter aurescens*	2848	2541	iRZ1179	^2729^
*Rhizobium etli*	371	387	iOR363	^2730^
*Rhizobium etli*	377	405	iOR450	^2731^
*Sinorhizobium meliloti*	522	503	iHZ565	^2732^
*Bradyrhizobium diazoefficiens*	661	1031	iYY1101	^2733^
*Rhizobium leguminosarum*	984	1257	iCS1224	^2708^
*Agrobacterium tumefaciens*	1106	1441	iNX1344	^2734^
*Buchnera aphidicola*	240	263	iGT196	^2735^
*Klebsiella pneumoniae*		1970	iYL1228	^2736^
*Klebsiella oxytoca*	1099	1457	KoxGSC1457	^2737^
*Ralstonia solanacearum*	1203	1825		^2738^
*Spirulina platensis*	837	875	iAK692	^2739^
*Xanthomonas oryzae*	808	839	iXOO673	^2740^
*Xanthomonas campestris*	338	437		^2741^
*Xanthomonas phaseoli*	1527	1556	iXpm1556	^2742^

Once in hand, a GEM is a powerful tool to direct genomic changes for metabolic engineering projects. Algorithms can predict the impact of enzyme knockouts on the production of a desired metabolite.^1496,1499^ The metabolite could be the product or it could be the precursor for a recombinant pathway. OptKnock has been used to increase auxin production to improve plant growth promotion in *Bacillus subtilis* by increasing the precursor pool.^2702^ OptReg and EMILiO can suggest gene over- or underexpression^1497,1498,2703,2704^ and k-OptForce can use kinetic information.^2705^

GEMs can also model metabolic phenomena relevant to agriculture. For example, the GEM for the soil bacterium *Pseudomonas putida* KT2440 was used to predict growth on different carbon sources.^2706^ This information could be used to predict which root exudates it can consume or sensitivities to soil composition. Draft GEMs for over 200 soil microbes were constructed to investigate how microbial physiology and environmental parameters impact soil carbon cycling.^2707^ These predictions inform the optimization of the microbiome composition for soil carbon sequestration. GEMs have been used to study the symbiosis between bacteria and legumes at different stages of the biogenesis of nitrogen-fixating nodules.^1500,2708^ To guide the engineering of more competitive nitrogen-fixing rhizobia, a GEM was used to identify key plant metabolites catabolized during each stage of *Rhizobium leguminosarum* infection and differentiation (Figure 39A).^2708^ Transient myo-inositol catabolism was predicted to be critical for invasion and differentiation, but not rhizosphere colonization or nitrogen-fixation. Flux through the core metabolism of differentiated *Rhizobium leguminosarum* also differed from that predicted for other differentiated rhizobia species, meaning flux optimization may need to consider host-plant specific differences in nutrient availability.

**Figure 39:**
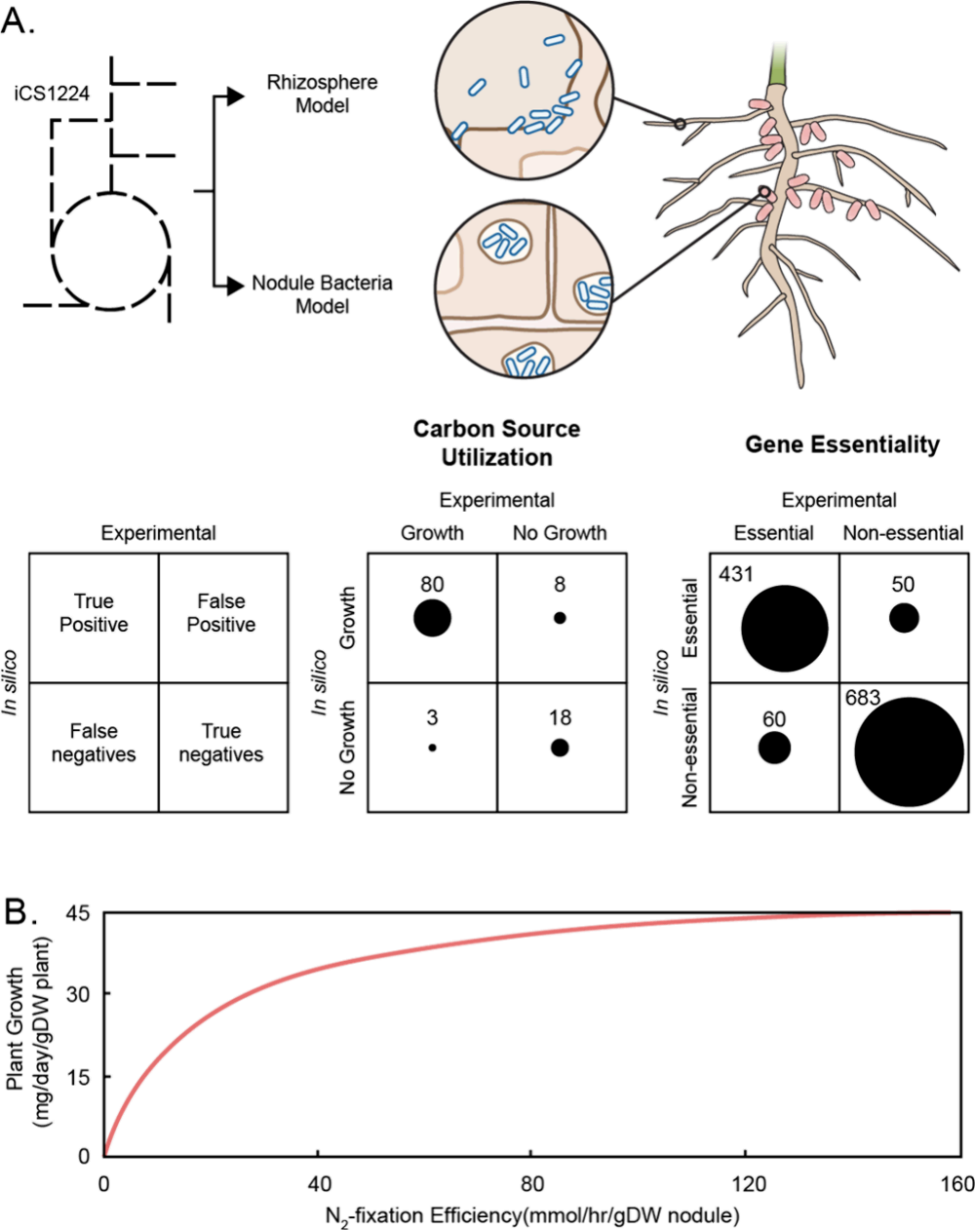
**Computational modeling of plant-associated microbe metabolism**. **A**. A GEM for *Rhrizobium leguminosarum* during growth in the rhizosphere and nodulation.^2708^ A GEM (iCS1224) was constructed using RNA-seq and gene essentiality experiments to inform life-stage-specific metabolic models. The tables visualize the agreement between GEM predictions (*in silico*) and experimentally-determined phenotypes using phenotype microarrays (Carbon Source Utilization) and INSeq data (Gene Essentiality). Circle size corresponds to the number of genes. **B**. Predicting the effect of nitrogen fixation on legume growth.^1500^ A multi-species GEM encompassing nodulated *Medicago truncatula* and nitrogen-fixing *Sinorhizobium meliloti* was used to predict nitrogen exchange and growth of *Medicago truncatula* at various nitrogen-fixation rates of *Sinorhizobium meliloti* within the modeled nodule. Data reproduced with permission from ref 1500. Available under a CC BY license. Copyright 2020, Nature Communication.

Software can also aid the design of recombinant pathways. OptStrain optimizes the production of a new or existing metabolite by recommending recombinant enzyme additions.^1498^ It can predict the minimum number of non-native reactions required to convert native metabolites to a target molecule and then suggest genes to perform those reactions from an enzyme database. In some cases, the pathway to a desired molecule may be unknown or it may be a molecule not found anywhere in the natural world.

“Retrosynthesis” refers to the artificial combination of enzymes sourced from different pathways in a new way to create a desired unnatural chemical.^997^ Algorithms have been developed to automate the selection of enzymes based on a molecule that a user wants to make.^2709–2713^ Enzymatic synthesis planning has also been introduced into programs that have traditionally focused more on chemical synthesis planning.^2334^ Retrosynthetic software could be used to build pathways to agrochemicals for which no natural pathway exists.

##### 3.6.1.3 Simulation of Microbial Communities

In the field, microbes interact with one another and organize into consortia. These interactions can be through metabolic interdependencies, antimicrobials, resource competition, quorum communication signals, and other mechanisms.^2743^ Section 3.5.6 outlines how cells can be engineered to enhance these interactions. Here, simulations are described that can aid the design of consortia and predict their spatial and temporal organization.

###### 3.6.1.3.1 Microbes as Interacting Agents

Agent-based models simulate microbial communities by individually representing each microbe (an agent) and measuring how the system progresses due to their interactions. NetLogo and FLAME provide generalized software platforms for agent-based models.^2744–2746^ Each allows users to customize the behaviour and characteristics of the microbe and environment. For example, NetLogo has been used to model the dynamics of soil organic matter and predict levels of CO_2_, soluble N, and mineralized N in the topsoil.^2747^ It can make these predictions for different soil types and also simulate plant-microbe interactions by defining plant growth as dependent on nitrate concentrations in the soil.^2748^

Specialized agent-based modelling tools have been developed for different biological processes.^2749^ For example, some software packages simulate 2D and 3D bacterial colony and biofilm growth.^2744,2750–2756^ They have been used to map the spatial distribution of soil microbiomes during rhizosphere colonization. The colonization of clover roots by *Escherichia coli* expressing *gfp* was simulated and validated under different environmental conditions, including pH and nutrient availability.^2757^

###### 3.6.1.3.2 Metabolic Modelling of Microbial Communities

Genome-scale metabolic modelling (Section 2.5.2) has been scaled to simulate microbial communities to make predictions regarding interconnected metabolic networks amongst the residents.^2758,2759^ The symbiosis between legumes and N-fixing bacteria was studied by combining a multi-tissue GEM model of the legume *Medicago truncatula* with a GEM of the diazotrophic symbiont *Sinorhizobium meliloti*.^2760–2762^ This model predicted that increasing nitrogen fixation efficiency leads to diminishing returns to plant growth, meaning engineering efforts to optimize nitrogen fixation in *Rhizobia* may have limited benefit to legume productivity (Figure 39B).

The metabolic modelling of consortia has been used to improve bioremediation. A GEM for *Arthrobacter*, which can degrade the herbicide atrazine, was combined with GEMs of other species to predict which could enhance degradation when added to the consortium.^2763^ This model suggested that *Halomonas* and *Halobacillus*, which do not degrade atrazine, could enhance overall degradation by cross-feeding with *Arthrobacter. Arthrobacter* can degrade atrazine and its coupling with these strains increase the prevalence of this species. Other models have predicted the impact of consortia on the degradation of naphthalene, uranium, and methane.^2764–2766^

AI tools can be applied to build complex synthetic consortia. AutoCD (Community Designer) uses a Bayesian approach to select the appropriate set of interactions to achieve a user-defined mathematical representation of the intended community behaviour.^2767^ AI tools are being used to design consortia that benefit soil and plant health.^2768–2772^ They can identify microbiome properties that most influence plant health, growth, and productivity.^2773–2776^ A neural network was developed to predict combinations of species that could aid phosphate accumulation in *Arabidopsis thaliana* shoots.^2777^

##### 3.6.1.4 Designing Genetic Sensors and Circuits

Genetic circuits are difficult to design by hand because they require the balancing of TF expression, the combination of many genetic parts, and their function is defined by multiple states (Section 3.3.4). Early simulations of genetic circuits were based on solving ordinary differential equations (ODEs) (*e.g.*, using MATLAB) and bifurcation analysis (*e.g.*, AUTO).^887,1263,1367,2778^ These models can predict how design choices can impact the response, including the placement of TF operators in a promoter and optimal expression levels that could then guide the selection of an RBS from a characterized library.^2779–2781^ As an example in soil bacteria, these tools have been used to design NOR gates in the soil bacterium *Pseudomonas putida*.^2782^ Bifurcation algorithms, such as AUTO, can analyse sets of ODEs to determine if a switch can have multi-stable states (*e.g.*, a bistable switch) or Hopf bifurcations (oscillators).^1263,1367,2778^ These approaches are limited because they only provide qualitative guidance, rather than making the design choice; for example, that expression needs to be increased but not being able to identify a specific RBS sequence to do so.

Design automation seeks to abstract the problem so that a user can specify the circuit that they want using a text-based language, like programming a computer.^729,2372,2373,2783–2787^ The software then generates the DNA sequence encoding the desired circuit. Cello allows a user to upload sensors and specify the desired circuit operation using Verilog, the same language used to design electronic circuits.^158,1975^ The DNA sequence encoding the circuit is designed by the software by combining gates provided in a “user constraint file” (UCF). The first UCF was for *E. coli* using only NOR/NOT gates based on repressors.^1340^ Subsequently, UCFs have been developed for *Bacteroides* and yeast (*Saccharomyces cerevisiae*). The same Verilog-specified circuit can be designed for different species simply by selecting it from a drop-down menu. Large circuits have been built using Cello, including multi-input multi-output logic, integrated toggle switches, and the recoding of entire computer chips inside cells.^2367,2397^ Moving it to soil bacteria would only require the development of UCFs for the species desired.

#### 3.6.2 Automated Engineering of Agricultural Bacteria

DBTL cycles are being carried out by BioFoundries, where computer aided design software informs the design, construction, and characterization of engineered strains facilitated by automation and high-throughput robotic platforms.^1440,1986,2634,2635^ AI/ML tools can be used to interpret experimental outcomes to inform the next round of designs.^2633,2788^ The BioFoundry model has been adopted by industry to develop high-performance strains to various commercial products.^2635,2789,2790^ Currently, BioFoundries tend to focus on model bacteria and yeasts because of the ease of automating their transformation and growth and the availability of part libraries. However, they could expand into agriculturally relevant strains, such as bacilli, pseudomonads, and filamentous fungi.^2791,2792^ An agricultural example is the rapid optimization of a strain to produce the pesticide limonine.^1616,2793^

A fully automated pipeline was developed to produce the flavonoid (2S)-pinocembrin in *Escherichia coli*, an antibacterial/antifungal against plant pathogens.^2632^ The pipeline encompassed the design of the biosynthesis route (RetroPath), enzyme selection (Selenzyme), sequence optimization (PartsGenie), construct assembly, automated strain characterization, and ML to generate the next round of designs. A similar semi-automated approach guided the optimization of naringenin and other related plant flavanones in *Escherichia coli*.^2794^

#### 3.6.3 Rapid Optimization of Pathways Using Inducible Systems

A problem with using strain construction to test variants is that it is relatively expensive and time consuming to build each strain individually, thereby limiting the scale of the libraries. This consideration is especially true when testing something relatively simple, like varying enzyme expression levels to balance flux through a metabolic pathway. Techniques have been developed in order to find the optimum using a single strain using alternative methods to scan through expression levels or to use RNAi or dCas9 to knock down combinations of native genes.^2795^

A simple approach is to use inducible systems to control each gene and then test various combinations of inducers to identify the optimum level. This requires that the strain contain multiple inducible systems that do not interfere with each other. To this end, the “Marionette” sensor array offers 12 orthogonal inducible systems, each of which was optimized using directed evolution to increase the dynamic range, reduce the off state, and increase orthogonality. Originally developed in *Escherichia coli*, the sensors have been moved into agriculturally-relevant bacteria, including *Azorhizobium calinodans*, *Azotobacter vinelandii*, Pseudomonas protogens PF-5, *Pseudomonas stutzeri, Rhizobium sp. IRGB74*, and *Klebsiella variicola*.^2028,2133^ They have been applied to optimize nitrogen fixation and herbicide/nitrification inhibitor alkylresorcinol products. The challenge with using this approach is that inducible systems may not scan a large enough range of expression in a graded manner the concentration of inducers

#### 3.6.4 Prototyping Genetic Designs in Cell-Free Systems of Soil Bacteria

Cell-free protein synthesis (CFPS) mixes contain the essential components for transcription and translation *in vitro*.^1611^ They are made either by lysing bacteria to make the mix or are prepared from the ground-up by combining purified components required for transcription and translation. DNA can be cloned or synthesized and added to the mixture and expression measured using a fluorescent protein or mRNA reporter or using qRT-PCR. CFPS mixes have been used as agricultural diagnostics; for example, CFPS-based sensors have been used to measure water and soil pollution.^2796–2798^ The sensor is based on a regulatory protein that is included in the CFPS mix and turns on gene expression when its signal is sensed.

CFPS mixes also offer the means to prototype a genetic part or system before testing it in a living cell.^2799^ The DNA encoding the system is added to the CFPS mix, incubated, and then evaluated for performance by measuring mRNA or protein concentrations. This process is very rapid, allowing a construct to be tested immediately after construction and is conducive to high-throughput testing.^1612,2672,2800,2801^ It also allows for variables to be tested that would be difficult in a living cell, such as the impact of varying ATP concentration on the performance of a pathway. A downside is that the CFPS components change dynamically during the test and will run out of metabolites and energy as the reaction progresses. The measurement changes as a function of these concentrations, making the data sometimes hard to interpret. Despite this possibility, *in vitro* results have been observed to correlate well with *in vivo* activity.^2802^

CFPS systems are available for many bacterial species, including agriculturally-relevant *Bacilli*,^2672,2803,2804^ *Klebisella*,^2804,2805^ *Pseudomonads*,^2014,2672^ and *Streptomyces*.^2806^ They have been used for part characterization by adding DNA containing the part and an appropriate reporter. For example, a library of 15 RBSs were screened using a *Pseudomonas putida* CFPS.^2014^ To increase the throughput, CFPS systems can be parallelized or multiplexed. A library of 1,300 promoters was screened for the soil bacteria *Klebsiella oxytoca, Pseudomonas putida, Pseudomonas agglomerans*, and *Bacillus subtilis*.^2804^ The throughput can be increased even further by combining CFPS-containing droplets with flow sorting and next-generation sequencing (NGS). This high-throughput screen was used to assay more than a million combinations of promoters/RBSs.^1624^

Genetically-encoded sensors can be characterized using CFPS systems. They aid sensor design when the ligand cannot cross the cell membrane or exhibits toxicity. For *Bacillus megaterium*, a xylose sensor was characterized, including biophysical parameters that would be difficult to otherwise measure *in vivo*, such as the impact of DNA concentration.^2672^ A sensor for the plant root exudate vanillic acid was developed using a CFPS system by screening a library of TetR-family mutants for activity.^2313^ Sensors have also been developed using CFPSs for the herbicide atrazine, antibiotics, heavy metals, water contaminants and other signals of relevance to agriculture.^2796,2797^ Genetic circuits also can be characterized using CFPS systems, although measuring dynamic circuits can be difficult to the changing resources in the CFPS over time.^2807–2810^

Metabolic pathways have also been prototyped in CFPS systems.^2811,2812^ Combining crude cellular lysates enriched with overexpressed enzymes can reconstitute complete multi-enzyme biosynthetic pathways, which can be optimized by simply adjusting the volumes or identities of the enzymes added to the final reaction mixture. For example, hundreds of pathway combinations were tested to improve the production of 3-hydroxybutyrate and butanol in *Clostridium*.^1620^ *Streptomyces* CFPS systems have reconstituted biosynthetic pathways, NRPSs, glycosylated proteins, and proteins made with non-canonical amino acids.^2806,2813,2814^

#### 3.6.5 Learning from Large Datasets Using AI/ML

The evaluation of large strain libraries leads to datasets that are difficult to interpret to plan the next round of strain creation. Computational methods have been developed to aid this process, including numerical search methods and ML.^1451,2815–2818^ This enables rounds of optimization to be repeated, where each round interprets data for the previous one. ML has been applied to optimize pathways and species-of-interest in agriculture. It was applied in Bacillus subtilis to optimize the coding sequence for recombinant gene expression and surfactant production.^2819,2820^ Pseudomonads have been optimized for flavolin production.^2795^ Pseudomonas putida was optimized for isoprenol production by using ML to interpret data over four rounds of optimization and nearly one million variants that changed the native genes knocked down by RNAi.^2821^

### 3.7 Expanding Microbial Chassis

Bacteria are only a fraction of the soil microbiota. In soil, for every 100 bacterial cells, there are approximately 10 archaea and 10 fungal cells.^1643^ Because bacteria are easier to genetically manipulate, they have been disproportionately used in Synthetic Biology. However, archaea and fungi have critical roles in agriculture and more manipulation tools are needed. This section reviews the nascent tools that have been developed for these kingdoms.

#### 3.7.1 Archaea

Archaea are prevalent in the rhizosphere and influence soil chemistry and composition. Archaea associate with crops and play a role in nutrient exchange, growth, and immunity.^2822^ Ammonia-oxidizing archaea contribute to nitrification.^2823,2824^ Halophilic archaea capable of colonizing nutrient-poor soils have been effective at phosphate solubilization.^2825^ This process works by inhibiting a methane-producing enzyme in the methanogen. Archaea are promising hosts for agricultural applications because of their unique metabolisms and the fact that there are no known archaeal plant pathogens.^2822,2826^ Despite sparse genetic engineering tools, they have been engineered to overproduce chemicals and simple genetic circuits have been designed for them.^2827–2830^

Archaea are responsible for major emissions of the greenhouse gas methane from ruminants, such as cattle, and from rice paddies.^2831,2832^ *Methanosarcinales, Methanomicrobiales*, and *Methanobrevibacter* convert H_2_ and CO_2_ generated in these anaerobic environments into methane. The methane generated during rice and ruminant livestock production accounts for 11% and 15% of total anthropogenic methane production, respectively.^2833,2834^ The product Bovear, marketed as a cattle feed additive, is based on molecule 3-nitrooxypropanol, which is an enzyme inhibitor that blocks methane release.^2835^

Archaea have the potential to be used to enhance plant growth. Genomic evidence for auxin biosynthesis is widespread and the thermophile *Sulfolobus acidocaldarius* produces high levels of the plant hormone IAA.^2836,2837^ *Nitrosocosmicus oleophilus* colonizes the roots of Arabidopsis and its emission of volatile organics mediates the induction of the plant antimicrobial and systemic resistance responses.^2838^

There is a growing list of genetic engineering techniques available for archaea. Efficient DNA transformation is based on spheroplasts, where DNA can be introduced using liposomes or with polyethylene glycol (PEG).^2839^ Conjugation-based methods have also been developed for methanogens.^2840^ There are genes that can be used for selection and counter selection markers.^2841^ Genome editing is possible using site-specific integrases and CRISPR/Cas9 in specific strains.^2842–2849^ Archaeal GEMs have been published (Section 2.4.1), although they have been limited to industrial methanogenic species.^2850–2852^

Genetic part libraries for archaea are available. Promoters are a simplified version of their eukaryotic counterparts, requiring only a TATA box and TF B recognition element (BRE).^2853^ Promoters are recognized by a single eukaryotic-like RNAP. This relatively simple promoter architecture has led to the identification and characterization of many promoter libraries across *Archaea*.^2846,2847,2854,2855^ Archaeal translation has features similar to prokaryotic and eukaryotic signals. While some mRNA contains the SD sequence like a bacterial RBS, most archaeal mRNAs lack a 5′-UTR altogether and rely on protein factors to facilitate ribosome binding and translation initiation.^2856^ While the mechanisms of archaeal termination are unclear, efficient terminators have been characterized and generally rely on polyT tracts.^2857^ The promoter/terminator genetics and RBS biochemistry complicate the use of computational tools for the *de novo* design genetic parts for archaea (Section 2.5.1).

TFs in archaea mimic prokaryotic repressors and activators and function by controlling RNAP access to the promoter.^2858–2861^ Thus, bacterial TFs can be easily transferred to archaea by placing their operator sites in an archaeal promoter backbone.^2862^ Sensors have been built using native archaeal TFs to respond to nutrients (sulfur, phosphorous, nitrogen, carbohydrates),^2863–2869^ metabolites (phenolic compounds, acetate, methanol, amino acids),^2859,2870–2875^ and metals.^2876–2879^ Sensors based on riboswitches have also been constructed that regulate translation and reveal the SD sequence in response to fluoride or theophylline.^2830,2880^

CRISPR tools also work in archaea (Section 2.2.1). Cas9 has been used in very limited set of archaea for genome deletions and mutant generation.^2881^ Genome insertions and deletions were made using Cas9 in methanogenic *Methanosarcina acetivorans* by supplying HR templates or expressing NHEJ machinery from other archaea.^2848^ However, in some archaeal species, CRISPR nucleases were found to be unstable and were unable to be expressed.^2882^ To control native genes, CRISPRi works and has been used to turn off nitrogen fixation genes in *Methanosarcina acetivorans*.^2883^ Native archaeal CRISPR systems can be co-opted to target and cleave mRNAs to silence endogenous genes post-transcriptionally.^2884^

#### 3.7.2 Agriculturally-Relevant Fungi

Filamentous fungi and yeast play critical roles in agriculture.^2885^ Beneficial fungi colonize both plant tissues (endophytic) and plant surfaces (epiphytic).^2886–2890^ Arbuscular mycorrhizal fungi (AMF) are root-colonizing endophytic fungi that form symbiotic relationships with over 80% of plants.^2598,2891^ AMF provide phosphorus and minerals to the plant; in return, the plant shares fatty acids, amino acids, and sugars.^2891^ For example, *Rhizophagus irregularis* delivers phosphate to the plant and is marketed as a product that enhances cassava yield by improving root growth.^2892,2893^ Endophytic fungi enhance tolerance to environmental stress and improve plant growth and development.^2889,2894^ Fungi also increase resistance to pests and diseases and play an important role in biocontrol.^2895^ For example, *Trichoderma*, can sense-and-kill other plant-pathogenic fungi and comprise more than 60% of biopesticides.^2896–2898^ Genetic control of disease-causing fungi has been demonstrated. In the cereal pathogen *Fusarium graminearum*, a naturally occurring selfish gene encoding a toxin-antidote system was used to spread a nonfunction allele for a blight virulence factor.^2899^ Fungi can also kill insects and nematodes.^2900–2902^ The use of the entomopathogenic fungi *Metarhizium* against sugarcane spittlebugs is considered one of the most successful biological control programs, with an estimated 2 million hectares of sugarcane treated annually.^2903,2904^

Fungi also interact with bacteria in the soil, thus participating in the complex array of interspecies interactions (Section 3.4.8). Interactions in natural communities in the native *Arabidopsis* rhizome were inferred by correlating the abundance of specific members across populations.^2905^ Most filamentous fungi naturally associated with *Arabidopsis* roots restricted plant growth when reinoculated alone but could maximize plant growth and survival when co-inoculated with various native bacteria. The growth-promoting fungus *Serendipita indica* is auxotrophic for thiamine but can grow in the presence of *Bacillus subtilis*, suggesting *Serendipita indica* requires nutrient exchange with soil bacteria.^2906^ *Pseudomonas fluorescens* and the mycorrhizae *Laccaria bicolor* have been shown to reciprocally exchange thiamine and trehalose to support pre-symbiotic growth.^2907^ To improve their symbiotic competitiveness in low iron soils, the iron siderophore receptor *fhuA* from *Escherichia coli* was transferred to rhizobial isolates from pigeon pea lacking the ability to uptake siderophores.^2908^ The engineered rhizobial strains displayed increased growth rates when co-cultured under iron-limited conditions with the siderophore-producing mycorrhizal fungus *Ustilago maydis*.

##### 3.7.2.1 Fungal Transformation and Genome Editing

Compared to bacteria and yeast, filamentous fungi suffer from low transformation efficiencies. One approach is to degrade the cell wall to make it easier for DNA to pass. *Aspergillus, Neurospora, Fusarium*, and *Trichoderma*, can produce viable spheroplasts (partially digested cell wall) or protoplast (fully digested cell wall) following chemical and enzymatic treatment.^2909,2910^ Transformation of protoplasts is mediated by CaCl_2_/PEG treatment,^2911^ liposomes,^2912^ or electroporation.^2913^ Cell wall composition differs between species and cell types and so protoplast preparation protocols are not generalizable.^2910^

Some filamentous fungi can be transformed using *Agrobacterium* co-cultures containing plant-derived chemical cues (*e.g.*, acetosyringone) that induce mobilization of Ti-plasmid by *Agrobacterium*.^2914^ Transformation by *Agrobacterium* can utilize different fungal tissue material (protoplasts, spores, hyphae, or fruiting bodies) and is suitable for delivery of very large DNA cargo in excess of 150 kbp.^2910^ Another advantage of Agrobacterium is that it uses virulence transfer machinery that transports recombinant DNA into the nucleus and integrates it into the fungal genome.^2915^ More rarely, particle bombardment can be used to deliver DNA to fungal strains recalcitrant to other transformation methods.^2916^ Artificial plasmid vectors containing the *AMA1* sequence can replicate autonomously in some filamentous fungi and increase transformation frequency because they do not need to also be integrated onto the genome.^2917–2919^ However, these artificial plasmids are unstable, even under strong selection.^2920^

Gene editing tools (Section 2.1) have moved to industrially- and agriculturally-relevant fungal species.^2921^ Cas9 can either be integrated into the fungal genome, maintained on a plasmid, or transiently expressed.^2922,2923^ To facilitate inserting recombinant DNA payloads in different fungal species, landing pads with a shared gRNA target site and adjacent homology arms were introduced into the genomes of four *Aspergillus* strains (Figure 40A).^2924^ Cas9-mediated base editors were used to introduce early stop codons and deactivate polyketide synthases in *Aspergillus niger* (Figure 40B).^2925^ TALENs were used to make sequence specific knock-outs and gene replacements in a melanin biosynthesis gene and DNA helicase in the rice blast pathogen *Pyricularia oryzae*.^2926^ The efficiency of site-specific DNA insertion mediated by double stranded breaks was improved by inactivating the NHEJ pathway.^2927^

**Figure 40:**
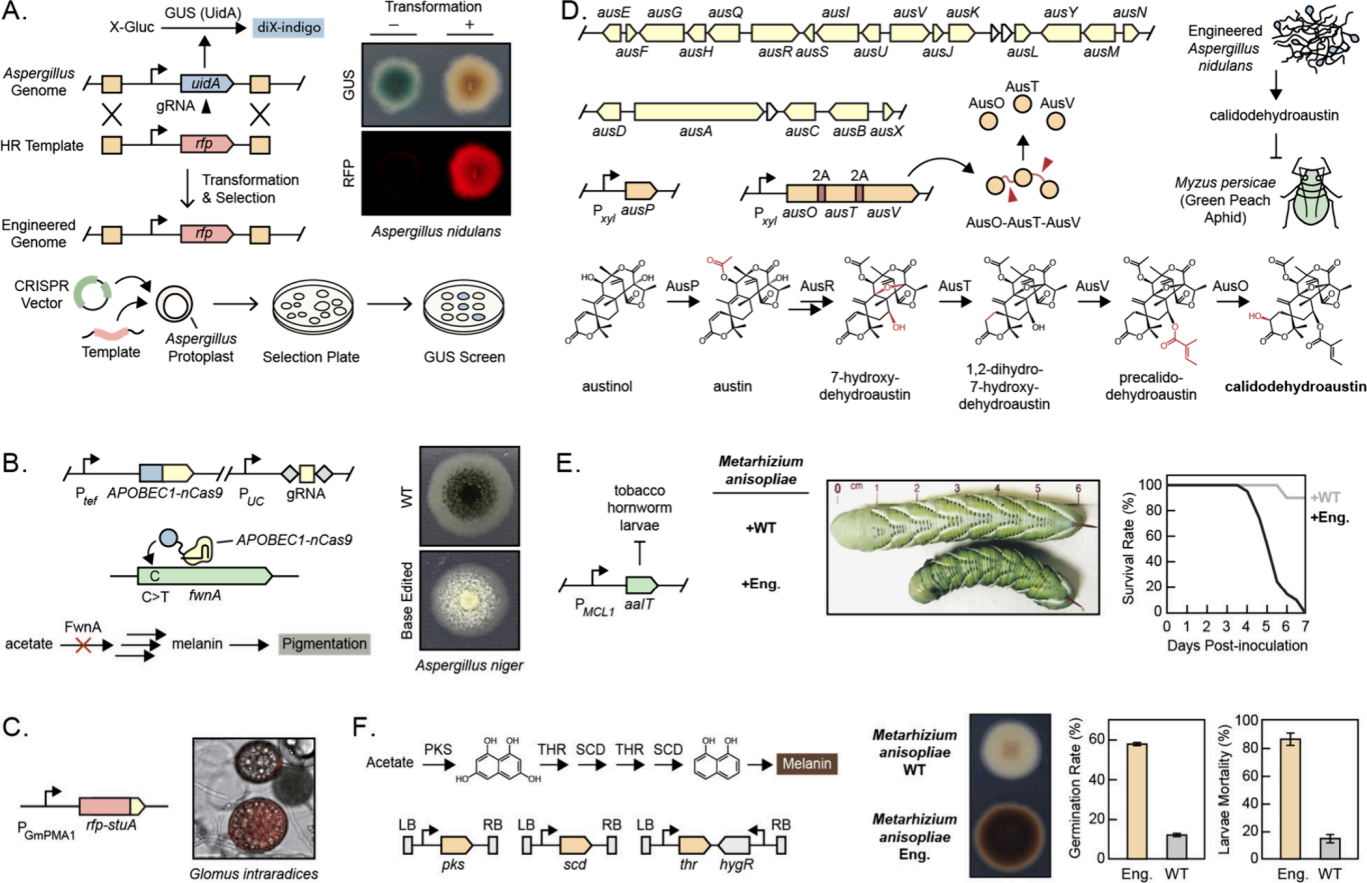
**Engineering agriculturally-relevant filamentous fungi**. **A**. Landing pads for engineering *Aspergillus* fungi.^2924^ Genomic landing pads encompassing a *uidA* (GUS) reporter gene and a gRNA target site were inserted onto the genomes of *Aspergillus aculeatus, Aspergillus niger, Aspergillus nidulans*, and *Aspergillus oryzae*. To integrate the DNA payload onto the landing pad, *Aspergillus* protoplasts were transformed with an AMA1-based vector harboring a selection marker, *cas9*, and a gRNA targeting *uidA*, and a linear repair template carrying the payload cassette (*rfp*). Colonies that survived selection can be screened for GUS activity on media containing X-Gluc to screen for the disruption of *uidA*. Images of fluorescent colonies reproduced with permission from ref 2924. Copyright 2021, American Chemical Society. **B**. Cas9-guided base editing in *Aspergillus niger*.^2925^ A base editor composed of a Sas9 nickase (nCas9) fused to a cytidine deaminase (rAPOBEC1) along with an gRNA was expressed from an Ama1 plasmid. The base editor disrupted the melanin precursor biosynthesis enzyme FwnA, which caused conidial bleaching. Images of fungal colonies reproduced with permission from ref 2925. Copyright 2019, Microbiological Research. **C**. Transformation of the AMF *Glomus intraradices* using particle bombardment.^2928^ Spores were bombarded with plasmids carrying *rfp* targeted to the nucleus with a StuA localization tag and expressing from the native AMF promoter *GmPMA1*. Expression of the fluorescent marker was transient and the vector did not integrate onto the genome. Fluorescent microscopy image reproduced with permission from ref 2928. Copyright 2008, New Phytologist. **D**. Metabolically engineering *Aspergillus nidulans* to produce austinoid insecticides.^2929^ *Aspergillus nidulans* possesses the biosynthetic pathway (yellow) to produce the insecticide austinol; the austinoid modifying enzymes AusP, AusO, AusT, and AuxV (orange) from *Aspergillus calidoustus* and *Penicillium brasilianum* were transferred to *A. nidulans* to produce austinoid derivatives including the product calidodehydroaustin, which targets green aphids. The modifying enzymes were expressed from the inducible xylose promoter (P_xyl_) as a polycistronic mRNA separated by 2A viral peptides that separate the proteins during translation (red arrows). Chemical modifications performed during each step are highlighted in red. **E**. Expressing a scorpion neurotoxin to increase the efficacy of the entomopathogenic fungus *M. anisopliae*.^2930^ The potent neurotoxin AaIT from the scorpion *Androctonus australis* was expressed from the native MCL1 promoter following protoplast transformation. To assess insecticidal activity, *Manduca sexta* larvae were infected with either wild-type (WT) or engineered (Eng.) *Metarhizium anisopliae* conidial suspensions. Caterpillar images reproduced with permission from ref 2930. Copyright 2007, Nature Biotechnology. **F**. Improving stress resistance and virulence of an antomopathogenic fungus by introducing melanin biosynthesis genes.^2931^ The efficacy of the insecticidal fungus *Metarhizium anisopliae* suffers in the field from environmental stresses, including as UV radiation. The dihydroxynaphthalene melanin biosynthetic pathway was expressed in *Metarhizium anisopliae* by introducing the polyketide synthase (PKS), scytalone dehydratase (SCD), and 1,3,8-trihydroxynaphthalene reductase (THR) genes from *Alternaria alternata* through *Agrobacterium*-mediated transformation. The ability of engineered (Eng.) and wild-type (WT) *Metarhizium anisopliae* spores to germinate and induce mortality in diamondback moth larvae (*Plutella xylostella*) was assessed after exposure to UV-B radiation. Images of fungal colonies reproduced with permission from ref 2931. Copyright 2011, Applied and Environmental Microbiology.

##### 3.7.2.2 Engineering Arbuscular Mycorrhizal Fungi (AMF)

Laboratory cultivation of AMF is difficult because most AMF are obligate symbionts that require their host plant to complete their life cycle. Dual-compartment culture systems have been developed and optimized so that fungal spores can be harvested without being contaminated by plant material.^2932^ Certain fatty acids can stimulate the branching of hyphae and sporulation of AMF in the absence of their plant hosts.^2933^

Introducing recombinant DNA into AMF is also difficult. The large variety in cell wall structures amongst filamentous fungi poses a challenge in developing transformation methods and the production of stable protoplasts.^2934^ *Gigaspora rosea* was transformed by particle bombardment to integrate the bacterial β-glucuronidase GUS gene into the genome.^2935^ Fluorescent reporter genes *dsRed* and *gfp* were introduced into *Glomus intraradices* using particle bombardment and *Agrobacterium tumefaciens* (Figure 40C).^2928^

Another problem that inhibits the development of genome editing tools is the lack of genome sequences. Only a few AMF species have fully sequenced genomes *(Gigaspora rosea, Rhizophagus irregularis*, *Rhizophagus cerebriforme, Rhizacophagus diaphanus*).^2936^

##### 3.7.2.3 Fungal Genetic Part Libraries

Genetic part libraries have been developed and characterized for filamentous fungi. A collection of 96 genetic parts containing natural and synthetic promoters (constitutive and inducible), terminators, fluorescent reporters, and selection markers have been developed for modular cloning in *Aspergillus* or *Penicillium* species.^2937^ A library of promoters mined from *Penicillium* and *Trichoderma* were characterized using a dual luciferase assay in two *Penicillium* strains and spanned four orders-of-magnitude in transcriptional activity. FungalBraid contains a database of genetic parts for filamentous fungi, including promoters and 5′-UTRs, selection markers, and 3′-UTR terminator sequences.^2938^ As of 2024, the Registry of Standard Biology Parts has 700 entries for fungal taxa.^2650^

Protein parts for fungi are similar to those used in yeasts, but there are some differences. Codon optimization can play an important role in improving heterologous gene expression.^2940^ Codon optimization is also beneficial to expression because it removes premature poly-A tracts and improves mRNA stability. Polycistronic operons can be constructed by expressing polyproteins separated by viral 2A peptides which self-cleave to generate individual proteins.^2929,2941^ For example, combinations of modifying enzymes were expressed as a polycistronic mRNA separated by viral 2A peptides in *Aspergillus nidulans* to produce derivatives of the austinoid insecticides (Figure 40D).^2929^

##### 3.7.2.4 Optimizing Fungal Bio-control Agents

Insecticidal fungi have been genetically modified to improve biocontrol properties. A potent scorpion neurotoxin was placed under the control of a strong constitutive promoter to increase anti-fungal toxicity of *Metarhizium anisopliae* (Figure 40E).^2930^ Potency was further improved by adding copies of a gene encoding a regulated cuticle-degrading protease to degrade an insect’s cytoskeleton.^2942,2943^ *Beauveria bassiana* has been engineered to overexpress an insect midgut-acting toxin targeting conidia to protect cabbage.^2944^ Toxicity was increased further by expressing the toxin from a promoter upregulated specifically during infection by fungal spores (conidia).^2945^

The fitness of fungi in the field has been improved by expressing melanin biosynthesis enzymes to protect against UV damage and superoxide dismutase to increase tolerance to oxidative stress (Figure 40F).^2931,2946,2947^ *Metarhizium anisoplia* was evolved for thermotolerance by culturing the strain continuously while slowly elevating the temperature in a growth chamber.^2948^ The resulting strains grew at higher temperatures but had diminished virulence when infecting grasshoppers. *Metarhizium brunneum* is a promising biocontrol agent for Varroa mites but is inhibited at the elevated temperatures in active honeybee hives (Section 2.3.1). A *Metarhizium brunneum* strain was subjected to ALE (Section 3.2.2) with a dual selection of elevated temperature and virulence.^2949^ Bee hives were iteratively infected with Varroa mites the fungal spores from dead mites were collected. Mycotoxins produced by *Fusarium moniliforme* in maize are damaging to livestock that are exposed to infected feed and can be degraded by amine oxidase enzymes in the fungus *Exophiala spinifera*.^2950^ Directed evolution (Section 3.2.7) was applied to amine oxidase to improve its activity in low pH and to increase protein secretion.

##### 3.7.2.5 Fungal Metabolic Engineering

Filamentous fungi can produce terpenoids, polyketides, indole alkaloids, and non-ribosomal peptides.^2951^ They have been used for decades in industrial bio-reactors to produce small molecule products and there are reviews of these efforts.^2952,2953^ Here, we summarize efforts to produce compounds of agricultural relevance that could be deployed by living fungi in the field. The number of GEMs (Section 2.4.1) is growing and facilitates computationally-guided metabolic engineering (Table 4).

**Table 4:** Genome-Scale Metabolic Models in Archaea and Filamentous Fungi

Species Name	Metabolite Number	Reaction Number	Model ID	Ref
**Archaea**				
*Methanosarcina barkeri*	558	509	iAF692	^2962^
**Filamentous Fungi**				
*Aspergillus niger*	1045	2240	iMA871	^2963^
*Aspergillus niger*	902	1,764	iHL1210	^2964^
*Aspergillus oryzae*	1073	1846		^2965^
*Aspergillus terreus*	1155	1451	iJL1454	^2966^
*Cordyceps militaris*	894	1267	iWV1170	^2967^
*Mucor circinelloides*	1413	1326	iWV1213	^2967^
*Neurospora crassa*	737	1046	iJDZ836	^2968^

Like bacteria, fungi have biosynthetic gene clusters that are responsible for the production of chemicals.^2954,2955^ Often explored in the context of human therapeutics, these chemicals can also be relevant to agriculture, for example as insecticides and herbicides. Many of the techniques described for bacterial biosynthetic gene clusters (Section 3.3) are also applicable to engineering their fungal counterparts. There are software packages and databases specific to identifying and cataloguing fungal gene clusters.^2075,2076,2100,2956,2957^

Austinoids are terpenes insecticides produced by fungi. New insecticides were made by screening combinations of four heterologous austinoid-modifying enzymes in *Aspergillus nidulans*.^2929^ Such secondary metabolites are produced at too low of titers for an environmental application. Low titers can be addressed by inserting strong constitutive promoters in front of the BCG, eliminating feedback inhibition, or repressing competing pathways.^2929^ Curcumin and atrochrysone carboxylic acid were overproduced in *Aspergillus oryzae* by increasing the malonyl-CoA pool by deleting a consuming enzyme and negative regulator.^2958,2959^

Native genes can be upregulated using CRISPRa, where an activation domain is fused to the DNA-binding domain (Section 3.3.5). CRISPRa was developed for filamentous fungi by fusing the VPR activator to dCas12.^2960^ Targeting CRISPRa to otherwise silent BCGs (Section 3.3.2) in *Aspergillus nidulans* led to the production of the pathway products. A similar system has been demonstrated in *Penicillium rubens* and applied to activating a silent BCG that makes the antimicrobial macrophorin.^2961^

##### 3.7.2.6 Fungal Cell Free Protein Synthesis (CFPS)

A limited number of CFPS systems have been published for filamentous fungi, produced for only a couple model species.^2969^ Like other eukaryotic cell-free platforms, filamentous fungi CFPS reactions contain glycosylation machinery and are capable of functionally expressing eukaryotic proteins.^2970^ CFPS reactions from *Neurospora crassa* and *Aspergillus niger* were used to express and purify fungal non-specific peroxygenases from *Cyclocybe aegerita*, which were previously unable to be expressed in common bacterial and fungal cells.^2969^ A *Neurospora crassa* lysate system was used to incorporate an unnatural photoreactive lysine analog into a fungal peptide to study native translation regulation.^2971^ Ribosome footprinting in *Neurospora crassa* lysates has also been performed to study native translation initiation and termination.^2972^

##### 3.7.2.7 Genetic Sensors and Circuits in Fungi

Libraries of inducible promoters from native metabolite-responsive systems have been compiled.^2973^ One of the most common inducible promoters is the *glaA* promoter from *Aspergillus niger*, where induction is driven by the action of maltodextrine, maltose, or glucose.^2974^ Other metabolite-inducible promoters include those induced by ethanol (P_alcA_),^2975^ cellulose (P_cbh_),^2976^ maltose (P_amyB_),^2977^ benzoic acid (P_bphA_),^2978^ arabitol (P_abf_),^2979^ glucose (P_cre1_),^2980^ xylose (P_exyl_),^2981^ methionine (P_met3_),^2982^ hydrogen peroxide (P_sodM_),^2983^ thiamine (P_thiA_),^2984^ copper (P_tcu_),^2985^ pH (P_gas_),^2986^ nitrate (P_niiA_),^2987^ and zearalenone (P_zeaR_).^2988^

The challenge with using natural promoters as sensors is that that they may be sensitive to other, sometimes uncharacterized, signals or changes in growth conditions. Synthetic promoters can be built that are not susceptible to endogenous regulation. A synthetic universal promoter was built containing a TATA box and upstream DNA-binding repeats for a LexA/VP16-based synthetic TF, which was expressed from a separate weak core promoter.^2989^ The universal promoter showed high activity in the bioproduction strains *Saccharomyces cerevisiae, Aspergillus niger, Trichoderma reesei, Pichia kudriavzevii, Pichia pastoris, Yarrowia lipolytica* as well as two osmotolerant yeast that not been previously engineered: *Candida apicola* and *Zygosaccharomyces lentus*. Expression from the promoter could be tuned by varying the number of LexA-binding repeat sequences upstream of the TATA box.

Sensors have been built using ligand-binding TFs moved from other species. When they were obtained from a prokaryote, the proteins needed to be fused to a NLS and activation domain (*e.g.*, VP16). Sensors for xylose, estrogen and tetracycline have been built for *Aspergillus*.^2990–2993^ A light sensor for *Trichoderma* was built by fusing the Gal4 DNA binding domain to a blue-light sensing domain.^2994^ Note that some commonly-used NLS tags in yeast, such as SV40, have been shown to be nonfunctional in some fungi, including *Fusarium oxysporum* and *Phytophthora sojae*.^2995,2996^ For these species, native NLS tags have been identified and tested.^2995,2996^

Simple genetic circuits have been constructed in filamentous fungi. A positive feedback loop was built using a cellulose-inducible promoter to drive the expression of its transcriptional activator CLR-2.^2997^

This circuit upregulated multiple cellulase genes 10-fold to drive lignocellulosic breakdown in *Neurospora crassa*. Building more sophisticated genetic circuits has been complicated by the lack of precision genetic parts and the growth of multi-cellular mycelia, which makes single cell analyses difficult.

##### 3.7.2.8 Advanced Yeast Tools

Synthetic biology tools have been developed for yeast, notably *Saccharomyces cerevisiae*, that have the potential to be moved to filamentous fungi. To aid design, large yeast part libraries have been developed, including constitutive promoters and terminators.^148,2998^ Many genetic sensors have been built for yeast including some that respond to agriculturally-relevant signals: DAPG,^1305^ salicylate,^2999^ vanillic acid,^3000^ naringenin,^3001^ xylose,^3002^ and environmental pollutants.^3003^ Prokaryotic quorum sensors (Section 3.1.7) have been moved to yeast that respond to signals produced by bacteria in the rhizosphere.^2293^ Up to four sensors have been introduced into the yeast genome at once (Section 3.6.3).^3004,3005^ These sensors have been applied to the high-throughput optimization of biosynthetic pathways.

Many genetic circuits have been built for yeast, including logic gates, bistable switches, and memory.^3006–3009^ The genetic circuit design automation software Cello has been developed for yeast (Section 3.6.1.4).^158,1357^ The software can design multi-input, multi-output circuits using gates built using phage- and TetR-family repressors. Similar circuits were build using dCas9-based NOR gates and simulation software.^3009^

The output of sensors and circuits can be connected to native gene expression using CRISPRi in *Saccharomyces cerevisiae* and *Candida albicans* (Section 3.3.5).^3010^ This tool has been applied to metabolic engineering projects to dynamically turn enzyme expression on or off to redirect carbon flux.^3004^ Genome scale changes can be guided with software that use GEMs to predict enzyme knock-outs that redirect carbon flux to the desired precursor.^3011,3012^

Extensive genome editing tools are available for yeasts, including those based on CRISPR, ZNFs, and TALEs (Section 2.1.1).^28,3013,3014^ Landing pads have been integrated into the chromosomes to insert DNA payloads rapidly and without disturbing native gene expression.^158,1968,3015^ Genome-scale engineering is also possible. MAGE (Section 3.2.1) has been extended to yeast (eMAGE).^3016^ eMAGE involves removing mismatch repair proteins while overexpressing a ssDNA-annealing protein. Flux through a heterologous β-carotene pathway was optimized in yeast by targeting 74 oligonucleotides to promoters, ORFs, and terminators in the pathway.^3016^ To guide mutations to specific regions of the genome, dCas9 fused to an error-prone DNA polymerase (EvolveR) has been moved into yeast (Section 3.2.2.2).^1860^

Entire yeast chromosomes have been built using *de novo* synthesis and minimization (Section 3.2.1).^277,278^ The chromosomes were designed to be easily engineered; all instances of the TAG codon were removed to facilitate codon reassignment, and repeat genetic elements, tRNAs, and some introns were removed.^277^ Remarkably, all 16 yeast chromosomes have been fused to make a single connected genome, which could simplify engineering efforts.^297^

##### 3.7.2.9 Yeast Applications in Agriculture

Yeast have been engineered to overproduce potent antimicrobial peptides when applied to plant tissue. Antifungal peptides are effective biocontrol agents against plant diseases, particularly in seeds and fruits.^3017^ However, economically producing and purifying these peptides at scale is challenging, making it desirable to express them in a strain that could be used as a living bioinoculant. To this end, cecropin A was expressed in *Saccharomyces cerevisiae* and *Pichia pastoris*.^3018^ These strains inhibited the germination of pathogenic mold spores on tomatoes and apple fruits, respectively. *Pichia pastoris* engineered to produce a pea defensin peptide (Psd1) reduced the severity of post-harvest decay caused by *Penicillium expansum*.^3019^ Engineering a Cecropin A-Psd1 peptide in *Pichia pastoris* delayed sour rot (*Geotrichum citriaurantii*) in peaches.

Yeast do not use RNAi machinery for regulation. Therefore, they can express dsRNA to knockdown the expression of genes when they are consumed by insects and other pests. *Saccharomyces cerevisiae* was engineered to express dsRNA targeting the y-tubulin gene of the invasive fruit pest *Drosophila suzukii*.^3020^ When larvae fed on food containing the engineered yeast, they died.

Many plant-associated yeast produce phytohormones in the rhizosphere and are suggested to be plant growth promoting.^3021^ The gibberellin precursor *ent*-kaurenoic acid was produced in the yeast *Yarrowia lipolytica* by upregulating its endogenous mevalonate pathway and the recombinant expression of three biosynthetic genes from *Arabidopsis*.^3022^ Expressing downstream biosynthesis enzymes from the fungus *Fusarium fujikuroi* led to the accumulation of various gibberellins in the production strains. A fluorescence-based biosensor of auxin was developed in *Saccharomyces cerevisiae* using an *Arabidopsis* auxin receptor.^3023^ This enabled strains to be rapidly sorted based on their auxin production rate. Plant-associated yeast have also solubilize mineralized phosphate, likely through the release of organic acids.^3024,3025^ Some soil yeasts produce siderophores that inhibit growth of pathogenic fungi by scavenging bioavailable iron.^3026^

Yeast has been developed as a bio-remediation tool. Heavy metals are taken up by the cells by expressing ion transporters, suppressing efflux systems, and producing intracellular detoxifying molecules, as was demonstrated for cadmium, nickel, cobalt, and arsenic.^1739–1741^ This approach was taken to make hyperaccumulating yeast strains that sequester heavy metals as well as oxy-polyatomic ions (arsenate AsO_4_^2–^, chromate CrO_4_^2–^), trivalent ions (aluminum Al^3+^), and strontium.^3027^ Further, yeast engineered to overproduce hydrogen sulfide gas, which reacts with metals to form insoluble metal-sulfide particles, were used to precipitate copper, cadmium, mercury, lead, and zinc from oil sands.^3028^

Yeast can also efficiently metabolize agrochemicals in agricultural soils and wastewater. Immobilized *Trametes versicolor* could efficiently degrade the pesticides diuron and bentazon in a bioreactor.^3029^ *Pichia pastori* transformed with two methyltransferases involved in degrading the herbicide atrazine from rice experienced less toxicity and were able to more quickly degrade it in culture.^3030^

## 4 Integrated System Engineering

Integrated design will be required for the most complex agriculture engineering projects that span the complete living system across kingdoms, rather than focusing on one species. Within the plant, these projects will combine sensing/computing with metabolic engineering, distributed across organelles, cell types, and tissues. Collectively, these will be connected to native regulatory pathways, stress responses, and morphogenesis. Further, they will integrate microbial capabilities within the designs, creating a signaling and metabolic network of interacting species.

In this section, we outline two “grand challenges” in plant synthetic biology: 1. smart plants that actively react to the environment and 2. self-fertilizing crop systems. These challenges were selected because they involve all aspects of cellular design, are large ambitious projects, and portions of the designs need to be distributed across species. We review steps, currently individual, toward these goals, noting that the solutions will ultimately require an integrated approach. Their full implementation will require CAD, prototyping, and automated screening to fully realize, aided by simulations/AI to predict performance before growth. Note that these challenges could also incorporate elements of insect and animal engineering into an even larger integrated system, although these topics are not covered in this review.

This section focuses on grand challenges that require a holistic approach that could integrate simultaneous plant and microbial engineering. In other sections of this review, we have focused on grand challenges that focus on just plant engineering: 1. increasing photosynthetic efficiency (Section 2.2.2.6) and 2. introducing apomixis into crops (Section 2.2.2.7). Similarly, there are grand challenges that focus only on microbial engineering alone: 1. stopping honeybee collapse (Section 2.3.1), 2. eliminating methane release from rice paddies (Section 3.7.1), and 3. overcoming “peak phosphorous” through AMF engineering (Sections 3.1.1. and 3.7.2).

### 4.1 “Smart Plants” That Surveil the Environment and Respond

Improving plants’ resilience to stress is a common objective in plant engineering and breeding.^593,3031–3052^ Many plants have been developed with traits to improve survival in extreme hot or cold, drought, pests, salt, pathogens, nutrient stress, soil conditions, and flooding.^3053–3059^ The traits span the expression of enzymes/TFs, metabolic pathways to make protecting molecules, or RNAs that target pests, amongst other genetic approaches. It is hard to combine these traits into one plant because stacking traits that require multiple genes is difficult, especially when they are constitutively expressed. To our knowledge, the largest number of traits put into a single commercial crop is Smartstax PRO (maize), which combines 6 insecticides targeting above-and below- ground insects and an herbicide resistance trait.^125^ Each gene is constitutively expressed, either in promoters that are on throughout the plant or are tissue-specific.

The vision of a “smart plant” is an engineered crop that has sensors/circuits to respond to stress that can then be implemented (Figure 41). The signals could either be autonomous or provided by farmers as agrochemicals in anticipation of an upcoming weather or climate event or the emergence of a new pest. The response genes can be distributed across the plant genome (nucleus, plastid, mitochondria, specific tissues) or introduced into plant-associated microbes that can be communicated with when the trait is needed. Note that this approach is different than using microbes expressing a function (*e.g.*, insecticide gene) that is used as a spray (Sections 3.1.3 and 3.7.2.9); rather, these would be microbes applied with the seed that turn on the response pathways when needed.

**Figure 41:**
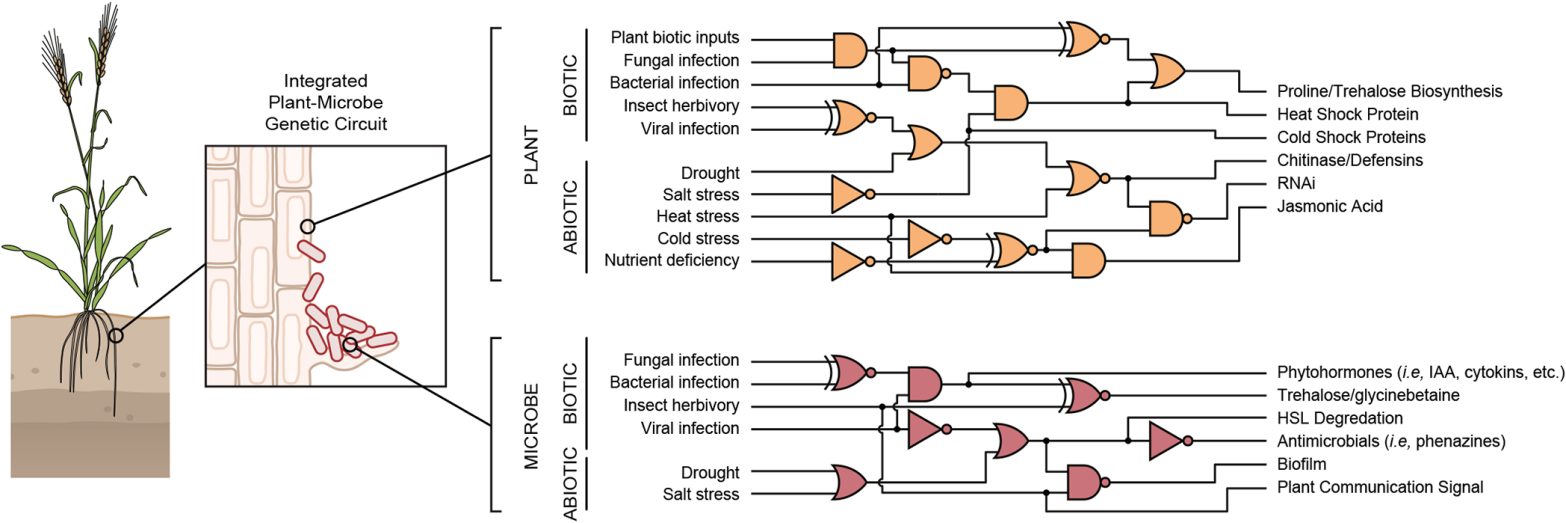
**Future vision of “smart” crops processing information to dynamically respond to their environment**. Large genetic circuits implementing complex biological logic could be used to integrate many environmental and biological signals to anticipate or optimally respond to stress. Not all the circuits have to be in the plant. Plant-associated microbes could sense threats to the plant and relay chemical signals to communicate with and prime the crop.

The description of this grand challenge starts with the outputs, in other words how different stresses can be mitigated by expressing genes in the plant or interactions with microbes. These traits are usually constitutively expressed and there are examples on the market. Then, genetic sensors that respond to various stresses are described. Ultimately, these inputs would be integrated by large synthetic circuits (Figure 41). Finally, work to create 1-input, 1-output simple sense-and-respond systems are described. These results could be scaled up and distributed across species to create agricultural systems that could actively respond to dozens or even hundreds of stress signals in the environment.

#### 4.1.1 Genes to Confer Stress Resistance

Abiotic stresses encompass extreme temperatures, flooding/drought, salt, soil content (metals, nutrients), or high/low light. This subsection describes the genes that can be introduced into a plant that confer resistance. Biotic stress can come from microbial pathogens, insects, animal herbivores, and plant parasites. Here, the focus is on those genes that can be expressed to increase the resistance against these threats, with an eye toward putting them under the control of sensors/circuits that respond to pests and pathogens.

##### 4.1.1.1 Abiotic Stress Resistance

The overexpression or knockout of native TFs can increase resilience to abiotic stresses.^3060–3077^ For example, the expression of a TF associated with drought resistance in *Arabidopsis* led to root structures normally found during drought.^3078^ However, native TFs may be problematic because they impact many genes that may lead to undesirable plant phenotypes. This problem can be addressed by targeting specific genes with synthetic TFs. For example, salt tolerant ryegrass was made by expressing ZFPs to target known genetic determinants of this trait.^1245^

Stress can cause intracellular proteins to unfold, which can be corrected by chaperones. Tobacco expressing a chaperone from halotolerant *Aphanothece halophytica* had higher heat and salt tolerance.^3079,3080^ Rice and maize exhibit improved cold and drought resistance when a cold shock protein that prevents RNA misfolding was expressed (marketed as DroughtGard).^3081,3082^

Recombinant enzymes can also protect against stress. One mechanism is to remove a toxic metabolite that accumulates. In rice, toxic methyloglyoxal accumulates during salt and drought stress and this can be cleared by expressing an enzyme to degrade it.^3083^ Other metabolites can confer a protective effect, particularly to osmotic stress and desiccation, including trehalose, fructans, sucrose, betaines, glycinebetaine, and proline.^3081,3084–3094^ Salt tolerant maize, rice, and wheat has been made by expressing enzymes that make these chemicals.^3095–3098^ Heat can stress a plant by producing reactive oxygen species. Heat-tolerant Arabidopsis was made by expressing an archaeal superoxide dismutase.^3099^

Soil properties and composition can also induce stress. Changes in pH can impact plant viability. At high pH, plants can experience iron deficiency because its endogenous scavenging enzyme are inactive. Tobacco with increased tolerance to alkaline soils was made by expressing a mutant yeast ferric reductase that is active at high pH.^3100^

##### 4.1.1.2 Biotic Stress Resistance

Some approaches to resist biotic stress are similar to those used for abiotic stress (above). For example, disease resistance was improved in *Arabidopsis* by tuning the expression of native TFs.^1199^ Pathogens can also be inhibited by expressing enzymes. Enzymes can be expressed to attack the microbial cell wall; for example, the expression of chitinase in broccoli blocked infection by Trichoderma.^3101,3102^ Similar examples have been shown in potato,^3103^ tomato,^3104^ lemon,^3105^ and guava.^3106^

Defensins are small peptides involved in innate immunity and confer broad-host-range antimicrobial functions.^3107^ Expressing a defensin sourced from Dahlia flowers in papaya reduced fungal infection by inhibiting hyphae growth.^3108^ Overexpressing a defensin in chili peppers made unripe fruit resistant to a fungal pathogen that causes anthracnose disease.^3109^

Pathogens and pests are susceptible to defense mechanisms that target their RNA, including plant viruses, bacteria, fungi, and insects.^3110^ GMO crops have been developed that transcribe hpRNA, siRNA, or dsRNA (Section 2.4.1) that target conserved and essential sequences in the pathogen.^3111,3112^ This approach has been used to make papaya trees that resist Papaya ringspot virus,^3113,3114^ tomatoes that are immune to crown gall disease caused by bacteria,^3115^ and barley that resists rust-causing *Fusarium graminearum*.^3116^ Insects are also sensitive to dsRNA that they can uptake when they eat the plant.^3117,3118^ Plants have been engineered to be insect resistant using this mechanism, including cotton that resists bollworm and corn that resists root-feeding larvae (SmartStax® PRO).^125,3119,3120^

Bacterial CRISPR Immunity has been moved to plants by expressing Cas9 and designing gRNAs that target plant viruses.^3121,3122^ Following this approach, tobacco was engineered to provide protection against bean yellow dwarf and beet severe curly top geminiviruses. The output of a circuit can be used to control plant immunity to viruses by driving the transcription of a gRNA that directs the Cas9 nuclease to a viral genome.

##### 4.1.1.3 Stress Response Genes Carried in Plastids

Chloroplasts offer a place to carry genes to confer stress response. They can be engineered to produce high titers of proteins or RNA and differentiated plastids can be plant tissue-specific (Section 2.2.2.3.2). Some metabolites that concentrate there are precursors for the biosynthesis of chemical protectants (Section 2.2.2.3.1). The chloroplast genome is also a good place to carry multigene pathways, including BCGs sourced from prokaryotes, that may encode antimicrobials difficult to produce in the plant genome (NRPS/PKSs) (Section 3.3.1). Finally, plastids lack RNAi processing proteins and accumulate dsRNAs (up to 0.4% total cellular content), thus improving pest resistance.^3123^ Colorado potato beetle larvae died when feeding on potato plants expressing dsRNA from the chloroplast, but not from the nucleus.^239^

#### 4.1.2 Engineered Microbes to Enhance Plant Stress Response

Some microbes can protect plants from salt and drought stress. Mechanistically, they perform this function by inducing osmolytes, ion transporters and aquaporins, and antioxidation or impeding salt uptake.^1708–1713^ Bacteria can also produce phytohormones that improve plant health during episodes of environmental stress.^3124^ For example, inoculation of wheat with the IAA-producing Pseudomonads increased plant growth >50% when exposed to salt.^3125^ Rice inoculated with *Bacillus amyloliquefaciens* had increased production of the osmoprotectants betaine and trehalose.^3126^ The tropical grass *Dichanthelium lanuginosum* can grow at high temperature (>65°C) in the presence of the fungus *Curvularia protuberata*, which induces heat shock proteins, melanin, osmoprotectants.^3127,3128^

Bacteria can kill pathogens by producing antimicrobials. *Rhizobium rhizogenes* K84 is a commercial biocontrol agent for fruit trees, nut trees, and roses that produces an antibiotic that kills the bacterial causative agent of crown gall disease.^3129,3130^ Wheat became resistant to fungi when inoculated with bacteria isolated from the wheat rhizosphere engineered to produce antifungals including phenazines, phenazine-1-carboxylic acid, diacetylphloroglucinol (DAPG), or pyrrolnitrin.^3131–3137^ Tomatoes are protected against damping off disease when inoculated with *Bacillus subtilis* engineered to overproduce mycosubtilin.^3138^

Protective bacteria can also be designed to interfere with the virulence pathways in a pathogen, rather than killing it outright. Common targets for interference are quorum signals required for virulence, the disruption of which is known as “quorum quenching.” The enzyme AiiA from *Bacillus thuringiensis* degrades AHLs used by many bacteria to communicate. Potato soft rot is caused by *Erwinia carotovora*,^3139^ which requires quorum signals for infection. It was blocked by expressing AiiA on the surface of *Bacillus thuringiensis* and *Pseudomonas putida* (Figure 42A).^3139,3140^

**Figure 42:**
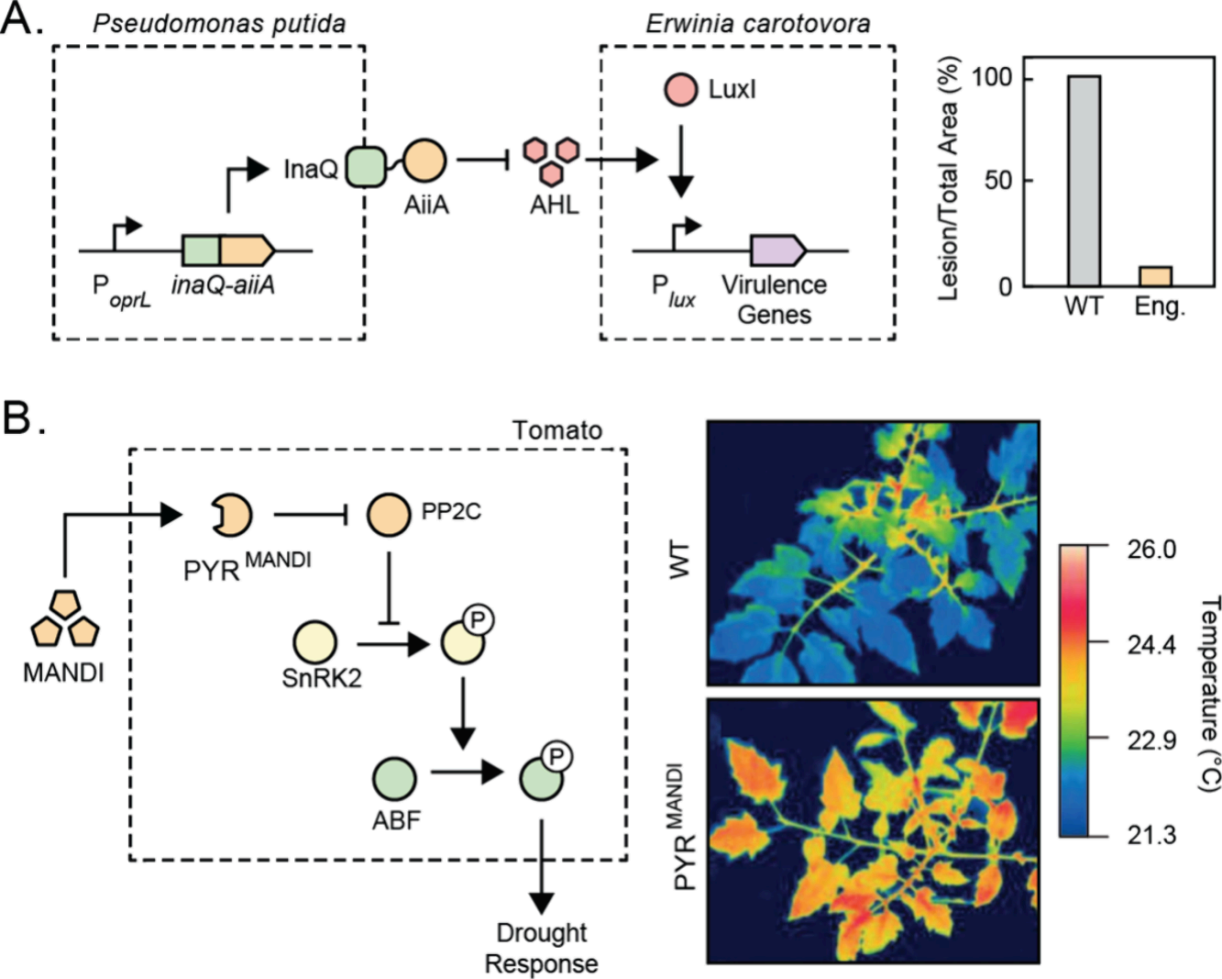
**Mitigating plant stress**. **A**. *Pseudomonas putida* engineered to degrade AHLs (Eng.) decreased root lesions caused by *Erwinia carotovora*.^3140^ This pathogen produces the quorum molecule AHL, which induces the expression of virulence genes required for plant pathogenicity. AHLs are degraded by AiiA, which is displayed on the cell surface of *Pseudomonas putida* by fusion to a native protein anchored on the surface. **B**. Control of drought response in tomato.^3143^ The sensor was based on a mutant of the ABA-sensing protein (PYR^MANDI^) that is responsive to the agrochemical mandipropamid (MANDI). MANDI activated the downstream ABA-induced drought response: type 2C protein phosphatases (PP2C), SnRK2 kinases (SnRK2), and ABA RESPONSE-ELEMENT-BINDING FACTORS (ABF). Engineered tomato plants (PYR^MANDI^) treated with 1 μM mandipropamid displayed elevated leaf temperatures, which is indicative of the closure of guard cells and decreased transpiration. Images reproduced with permission from ref 3143. Copyright 2015, Nature.

Archaea can also play a role in plant stress response. Some archaea can alleviate lead stress in rice paddies.^3141^ *Nitrosocosmicus oleophilus* induces Arabidopsis systemic resistance against the plant pathogens *Pectobacterium carotovorum* and *Pseudomonas syringae*.^3142^

#### 4.1.3 Plant and Microbial Stress Sensors

Most stress response traits involve the continuous expression of the genes, whether the stress is present or not. Placing these genes under the control of stress-responsive promoters has several beneficial effects. First, it allows may traits to be combined while reducing the burden on the plant. This burden could result in undesirable phenotypes, such as growth retardation. Second, it reduces the amount of recombinant protein and additional metabolites in the food supply. It can also allow a single plant to resist multiple opposing stress responses, such as hot/cold, low/high pH, or high/low light. This subsection describes promoters that respond to stress conditions, most of which have been characterized in plants. Microbes also have sensors that respond to stresses (Section 3.4.1) and they could be used to turn on protective factors from the microbial genome or the information communicated to the plant (Section 3.4.2).

Many individual sensors have been constructed in plants that respond to stress conditions (Table 5). Thousands of natural promoters in the nuclear and chloroplast genomes have been identified in plants that could be used as stress sensors.^255,3144–3149^ A problem with using natural promoters is that their response goes through a regulatory node that controls many genes (Section 2.2.1.1.1). This effect is pronounced when the promoter responds to TFs that are central nodes of the plant’s regulatory network.

**Table 5:**
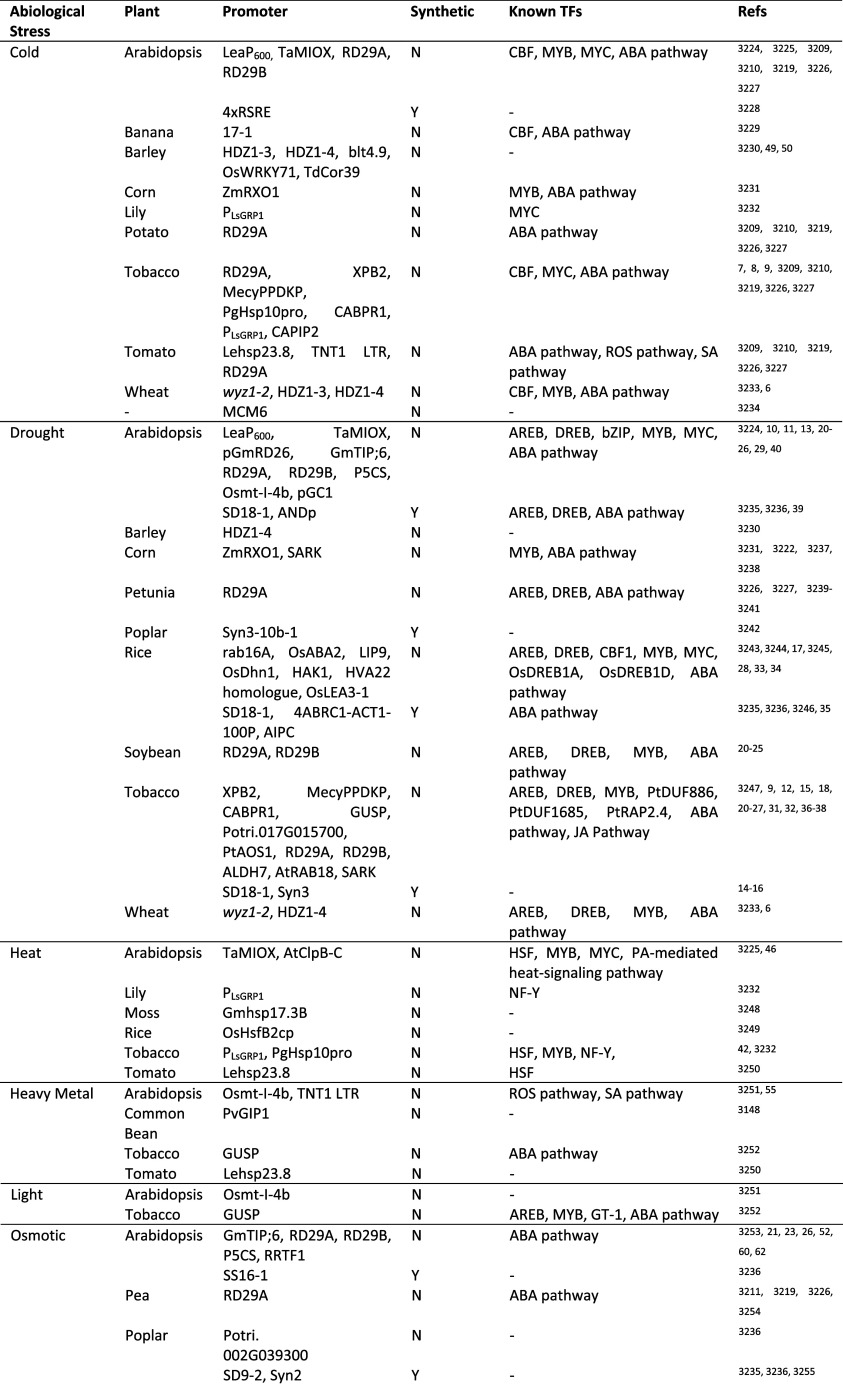

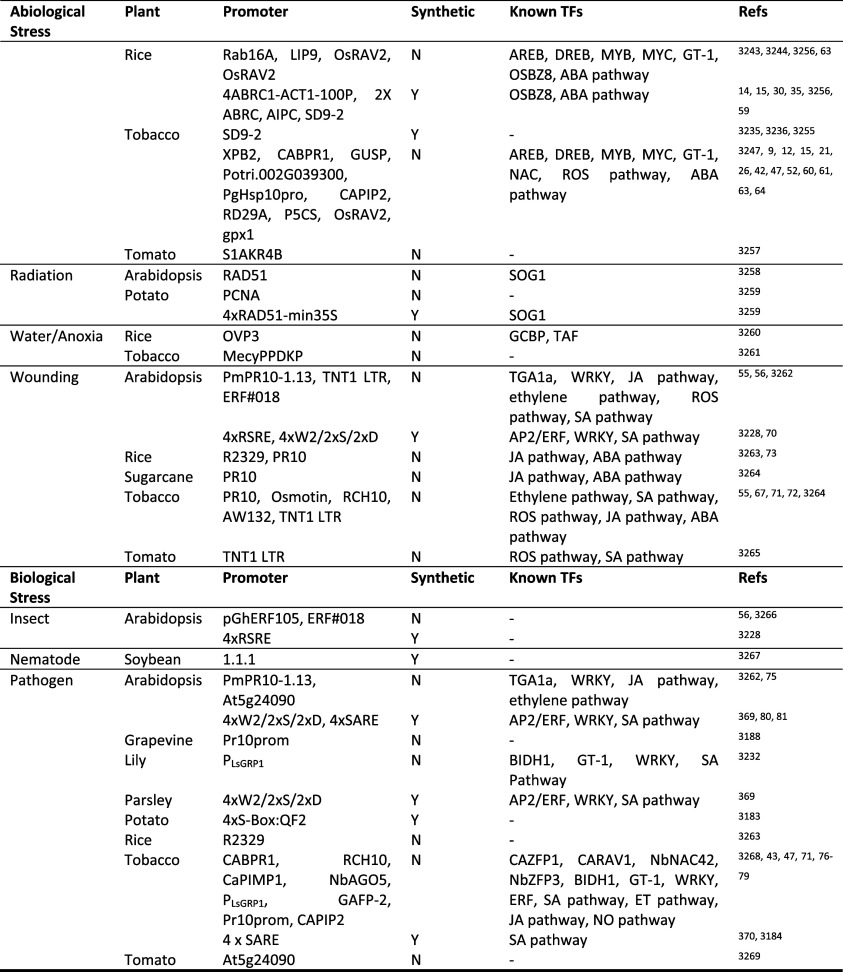
Stress-Specific Promoters in Plants^3148,3209,3210,3219,3224,3225,3226,3227,3228,3229,3230,3231,3232,3233,3234,3235,3236,3237,3238,3239,3240,3241,3242,3243,3244,3245,3246,3247,3248,3249,3250,3251,3252,3253,3254,3255,3256,3257,3258,3259,3260,3261,3262,3263,3264,3265,3266,3267,3268,3269^

Abiotic stressors (*e.g.*, drought) converge on abscisic acid (ABA) which controls seed dormancy and physiological changes to conserve water.^3150–3153^ Biologic stresses converge on the salicylic acid (SA) and jasmonic acid (JA) regulatory pathway, which regulate defense pathways.^3154–3158^ Pathways controlled by ethylene (ET), reactive oxygen species (ROS), and calcium (Ca^2+^) have been linked to both abiotic and biotic stress.^3159–3174^ They can alter leaves, root growth, fruit ripening, cell wall thickness and other ways that the plant can adapt. There is crosstalk between these major pathways; thus, natural promoters may respond to different stressors due to different TF binding sites.^1199,3075,3175–3179^ Alternatively, synthetic promoters can be built by inserting specific TF operators into a minimal promoter scaffold (Section 2.3.3).^3073,3180,3181^

Pathogen sensors turn on in response to an infection.^3182–3188^ Many natural promoters have been characterized as being inducible by pathogens.^3189–3194^ These promoters can be turned on by general changes in the regulatory networks involved in defense or in response to cell-surface receptors that recognize pathogens.^3195,3196^ Pathogens inject proteins into cells (*e.g.*, DNA-binding TALEs) that regulate native plant promoters to facilitate the virulence program.^3197–3201^ Pathogen-inducible synthetic promoters have also been designed by placing CREs into a promoter scaffold (Table 5).^369,1199,3183,3202–3205^

#### 4.1.4 Genetic Circuits to Integrate Sensory Information

Logic circuits could integrate sensory information and select which response is appropriate (Section 3.4.3). Logic increases the specificity in identifying which stress is being experienced. For example, drought induces ABA (AND NOT) ethylene. Salt stress induces ABA (AND) ethylene. In this example, the same two sensors can be used to differentiate stresses using logic. A logic circuit can have multiple outputs, one of which would be connected to the salt response and the other, the drought response. The logic can get more complicated if some resistance genes protect against multiple stresses.^3206^ Logic circuits can be designed where the outputs take this requirement into account. Logic circuits can be very large,^2364^ integrating dozens of signals and could be divided between the plant and microbes (Figure 41), where each communicates the result of the calculation to each other when necessary using cell-cell communication signals^1310,3207^ (Section 3.4.2).

There are also cases where the resistance genes need to be expressed at particular times. There are two ways that this can be done. The first is to have another input into a logic circuit that responds to the condition(s) where the response should be expressed. For example, a stress response gene may need to only be turned on at night, so a light sensor could be connected to the circuit. Logic operations can also incorporate tissue-specific sensors (Table 1) so that responses only turn on in certain locations of the plant.

Dynamic circuits can also time a response. For example, if an insect is sensed, it may be appropriate to turn on a pulse of insecticide production. They could also be used to stop producing a chemical protectant after enough has built up in the cell or to only produce a limited amount of a gene product that could be toxic.

Memory could be used to make a response permanent. It is desirable when it is important to continue to express stress-response genes even after the stress stimulus is removed. Examples might be to maintain a defense response after a pathogen is initially detected or if an abiotic stress (*e.g.*, drought) is likely to occur again after the first occurrence is sensed.

Circuits based on cell-cell communication and signaling between regions of the plant (*e.g.*, root-to-shoot) could propagate a signal from one region throughout the plant. Similarly, it could be possible to create volatile signals that are released by one plant and then signal the stress, such as an insect attack, to other plants in the vicinity.

#### 4.1.5 Stress Sense-and-Respond

The output of a stress sensor can drive the expression of a gene that alleviates the stress.^3208–3211^ Examples to date have been limited to single stresses that are sensed and simple responses. However, they represent the types of sensing and circuitry that could be expanded upon as envisioned above.

Temperature sensors have been used to turn on pathways to protect against extreme hot or cold. In transgenic barley and rice, a cold sensor was used to express a native TF that upregulates cold-responsive genes to make it resistant to frost.^3212^ To improve cold tolerance in rice, an abiotic stress sensor controlled the expression of chalcone isomerase, which increases flavonoid biosynthesis and leads to the production of protective antioxidants (Figure 43A).^3213^ *Eucalyptus* was also made frost tolerant using a cold sensor expressing a recombinant *Arabidopsis* TF (Figure 43B).^3214,3215^ It is now being grown commercially by ArborGen for timber and pulpwood in the southeastern US.

**Figure 43:**
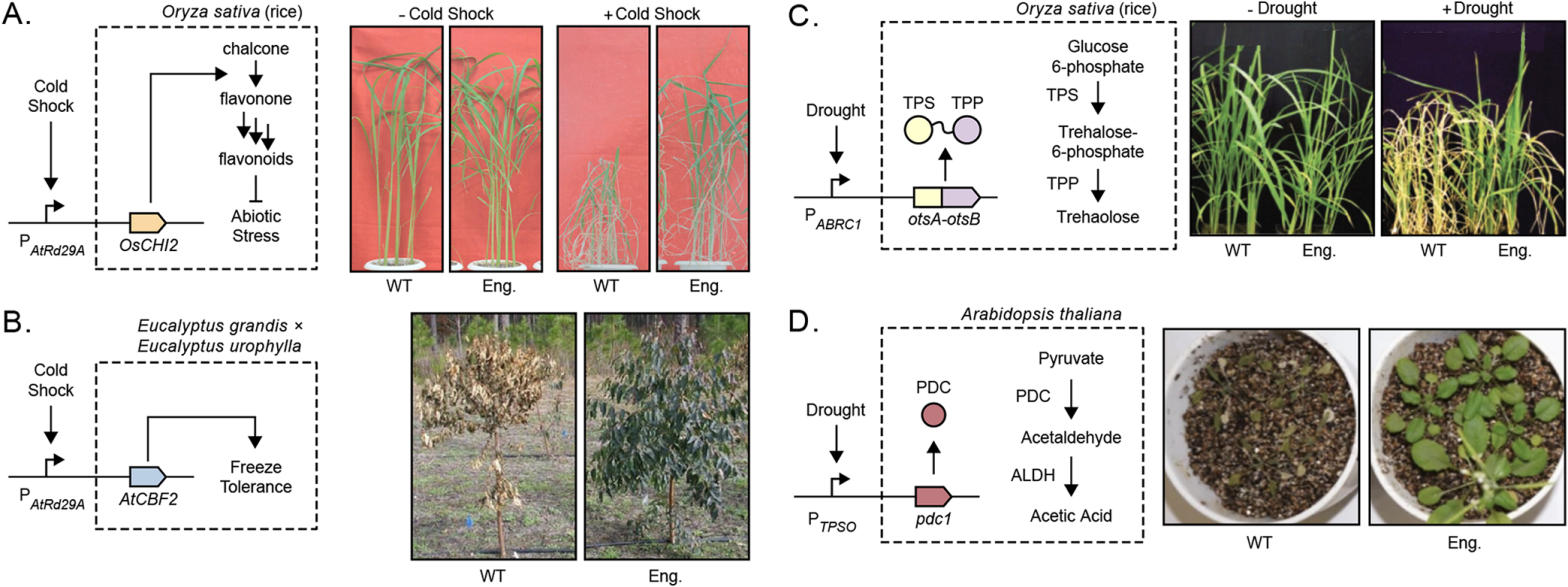
**Using genetic sensors to respond to plant stress**. **A**. Cold-stress resistant rice.^3213^ The rice enzyme chalcone isomerase (*OsCHI2*) controls the biosynthesis of flavonoids, which serve as antioxidants to protect plants from free radicals generated by various abiotic stress events. Engineered rice (Eng.) expressing *OsCHI2* from the *Arabidopsis* stress-responsive promoter P_*AtRd29a*_. Rice plants at the 4^th^ or 5^th^ stage were exposed to cold stress at 12 °C for 12 days. Wild-type: WT. Images of rice plants reproduced with permission from ref 3213. Copyright 2021, Environmental and Experimental Botany. **B**. Frost tolerant *Eucalyptus*.^3215^ *Eucalyptus* is desirable for its fast growth and dense canopy but is not hardy enough to grow in many intensive lumber regions. The high productivity *Eucalyptus* hybrid *E. grandis* × *E. urophylla* was engineered to express that the cold-tolerance TF (AtCBF2) from *Arabidopsis* was introduced downstream of the *Arabidopsis* P_rd29a_ stress-inducible promoter. The engineered trees (Eng.), but not the wild-type hybrid, survived winter temperatures of 16 °F in the southeastern US. Images of *Eucalyptus* trees reproduced with permission from ref 3215. Available under a CC BY-NC license. Copyright 2011, Springer. **C**. Stress-inducible trehalose biosynthesis in rice.^3086^ The *Escherichia coli* trehalose biosynthetic genes (*otsA* and *otsB*) encoding the enzymes trehalose-6-phosphate synthase (TPS) and trehalose-6-phosphate phosphatase (TPP) were fused and driven by a synthetic drought-responsive promoter (P_ABRC1_) containing four tandem copies of ABA-inducible CREs within a minimal rice promoter in engineered (Eng.) rice. Drought stress was assessed by two cycles of withholding irrigation for 3 days. Images of rice plants reproduced with permission from ref 3086. Copyright 2002, National Academy of Sciences. **D**. Acetic acid biosynthesis protects *Arabidopsis* from drought.^3218^ A drought-responsive *Arabidopsis* promoter (P_TPSO_) was used to drive the expression of pyruvate decarboxylase (PDC), which converts pyruvate to acetic acid with enzyme aldehyde dehydrogenase, in engineered *Arabidopsis* plants (Eng.). *Arabidopsis* plants were imaged after 15 days of drought stress. Images of *Arabidopsis* plants reproduced with permission from ref 3218. Available under a CC BY license. Copyright 2018, Scientific Reports.

In rice, the gene controlling the production of the protectant proline was placed under the control of an abiotic stress-responsive promoter.^3216^ This trait improved salt and drought tolerance. Also in rice, trehalose biosynthetic genes were placed under the control of a stress sensor, which decreased damage due to high salt and low temperatures (Figure 43C).^3086^

There are several cases where the resistance genes produce a negative phenotype if always expressed. For example, acetic acid can improve drought survival. However, overexpressing the acetic acid biosynthesis genes is detrimental to growth and yield. These effects were eliminated by placing it under the control of a drought-inducible promoter (Figure 43D).^3217,3218^ A similar observation led to the connection of a desiccation sensor to the overexpression of a native TF in Arabidopsis. If only the TF was expressed, then this led to a decrease in yield, but this was mollified by making it responsive.^3219^ Rice that overproduce proline grew to higher biomass when it was placed under the control of a stress-inducible promoter.^3216^

Plants naturally alter their morphology during periods of stress and engineering this response can better balance robustness and yield for needs in agriculture.^3220^ Under drought conditions, plants shed leaves (senescence) to decrease canopy size and reallocate resources. Leaf senescence can increase survival under severe stress, but often at the expense of yield.^3221^ Tobacco expressing the enzyme isopentenyltransferase from a senescence-inducible promoter overproduced the hormone cytokinin, which suppresses leaf senescence.^3222^ The resulting plants did not shed leaves during transient drought episodes and more quickly resumed growth following watering.

A farmer may know a stress is coming days before a plant could; for example, weather reports of an upcoming deluge or drought. To prepare the plants, an agrochemical could be sprayed in advance to induce stress response genes that protect the plant. This approach requires the design of sensors that respond to agrochemicals. Using directed evolution, the ABA-binding receptor PYR1 was mutated to bind to the fungicide mandipropamid.^3143^ This sensor was expressed in *Arabidopsis* and tomato and used to induce drought tolerant genes when exposed to mandipropamid (Figure 42B).^3143–3147,3149^ Sensors could also induce changes in root architecture in response to forecasted drought.^3223^ To this end, synthetic TF-promoter pairs were used to tune the expression of a mutant of TF that controls root branching to modify root morphology.^314^ These systems would allow plants to be prepared for drought, based on weather reports, prior to having to experience its damaging effects.

### 4.2 Self-Fertilizing Agriculture

Nitrogenous fertilizer is produced in industrial chemical plants using the Haber-Bosch process, which fixes atmospheric N_2_ to ammonia. This process is one of the most impactful in history – nearly 80% of the nitrogen in humans was derived in such a facility – but it also has an enormous impact on the environment.^3270,3271^ It is an energy-intensive process, accounting for approximately 30% of the energy input to agriculture and 1% of total global energy usage. Its production leads to greenhouse gas emissions and when it is applied to soil, 50% is volatilized as N_2_O (300-fold stronger than CO_2_ as a GHG) or run-off water pollution and dead zones. Some bacteria are able to fix nitrogen using the enzyme nitrogenase, where the energy and reducing potential come from ATP and NADH, respectively. Since the dawn of genetic engineering, harnessing this potential in agriculture has been recognized as a grand challenge.

The bacteria that can fix nitrogen naturally associate with legumes (beans, peanuts, etc.) by residing in specialized nodules along the roots. There, they are provided with a carbon source and the low-O_2_ environment needed by nitrogenase in exchange for fixed nitrogen (ammonia or nitrate). In contrast, cereals (rice, wheat, corn, etc.) are mostly unable to form these associations with bacteria, so synthetic nitrogen needs to be added for them to grow. (A fascinating counterexample is an indigenous corn variety in Mexico that derives nitrogen from various nitrogen-fixing bacteria that live in a mucilage excreted by the plant’s aerial roots).^3272^ In practice, synthetic nitrogen is also added to legumes on industrialized farms to maximize yields and this shuts down the formation of nodules and represses bacterial nitrogen fixation. Therefore, the nitrogen fixation pathway needs to be moved to relevant crops and turned on under field conditions.

Since the problem was first articulated in the 1970’s, there have been many approaches to make biological nitrogen fixation viable for cereals (Figure 44).^3273^ First, bacteria and archaea that naturally associate with cereal roots could be made to fix nitrogen. Second, cereals could be engineered to create nodules that recruit N-fixing bacteria. Third, the nitrogen fixation (*nif*) genes could be moved to the plant, either the nuclear chromosomes, plastid, or mitochondrial genome. Finally, bacteria have been engineered to use the energy from solar cells to fix and store nitrogen that can be applied to the field later. Each of these strategies has its advantages and the 100% replacement of synthetic nitrogen will likely require a combination of approaches, including new green chemical sources.^3274^

**Figure 44:**
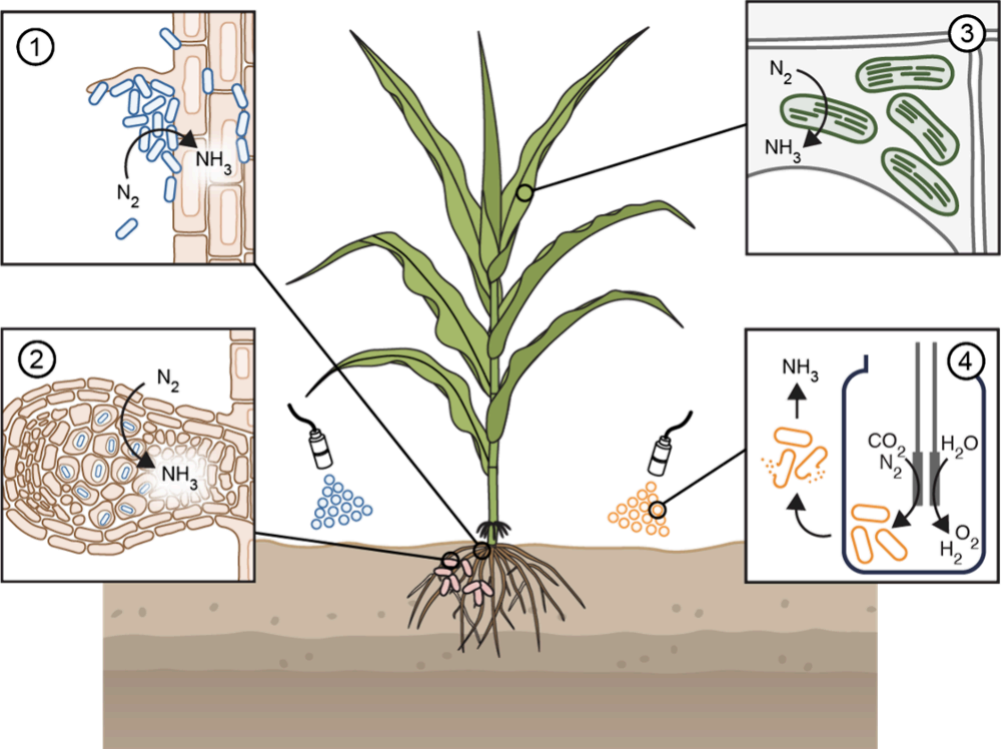
**Self-fertilizing cereal crops through genetic engineering. (1)** Plant roots are inoculated with engineered nitrogen-fixing microbes that fix and secrete nitrogen to be taken up by the plant. **(2)** Engineered nodulation in a cereal crop. The nodule would be able to recruit bacteria that normally inoculate only legumes. **(3)** The *nif* cluster is transferred to plant cells, whether it be the plastid (shown), mitochondrial, or nuclear genome. **(4)** An “artificial leaf” uses electricity, which could be from a green source, to support the diazotrophic growth of a microbe that can be applied to the field as a nitrogenous fertilizer.

#### 4.2.1 Engineering N-Fixing Species

Many bacteria that associate with cereal roots have the genes necessary to fix nitrogen. The challenge is that these genes are strongly repressed in this context, especially when nitrogenous fertilizer is applied. Even in the nodule environment, the application of synthetic nitrogen shuts down the natural ability of the bacteria to fix nitrogen. Genetically engineered bacteria have been developed to circumvent this problem. Genetic part libraries and inducible systems have been developed for N-fixing species, thus improving the ability to optimize N-fixation and the secretion of fixed nitrogen in a form that can be taken up by the plant (Table 2).^2027,2028^

To improve N-fixation in legumes, *Sinorhizobium meliloti* was engineered to secrete ammonia constitutively in the presence of fixed nitrogen. The genome was modified to overexpress the NifA activator, which turns on the nitrogen fixation (*nif*) genes (Figure 45A).^3275–3277^ Additionally, the pathway to C4-dicarboxylic acid uptake (*dctABD)* was introduced to support the increased metabolic demand. This bacterium improved alfalfa yield in the field.^3275,3278,3279^ This strain obtained regulatory approval because the bacterium cannot associate with weeds and could not survive after the alfalfa plants were removed.^1809,1811–1813^ Despite being approved for field trials, commercial approval was not pursued. The challenge was that the pathways and genetic parts were from different genera: the *dctABD* from *Rhizobium leguminosarum* and promoter/terminator from *Bradyrhizobium japonicum*/*Escherichia coli*. Most problematic was the antibiotic selection marker *aadA* from the human pathogen *Shigella flexneri*.

**Figure 45:**
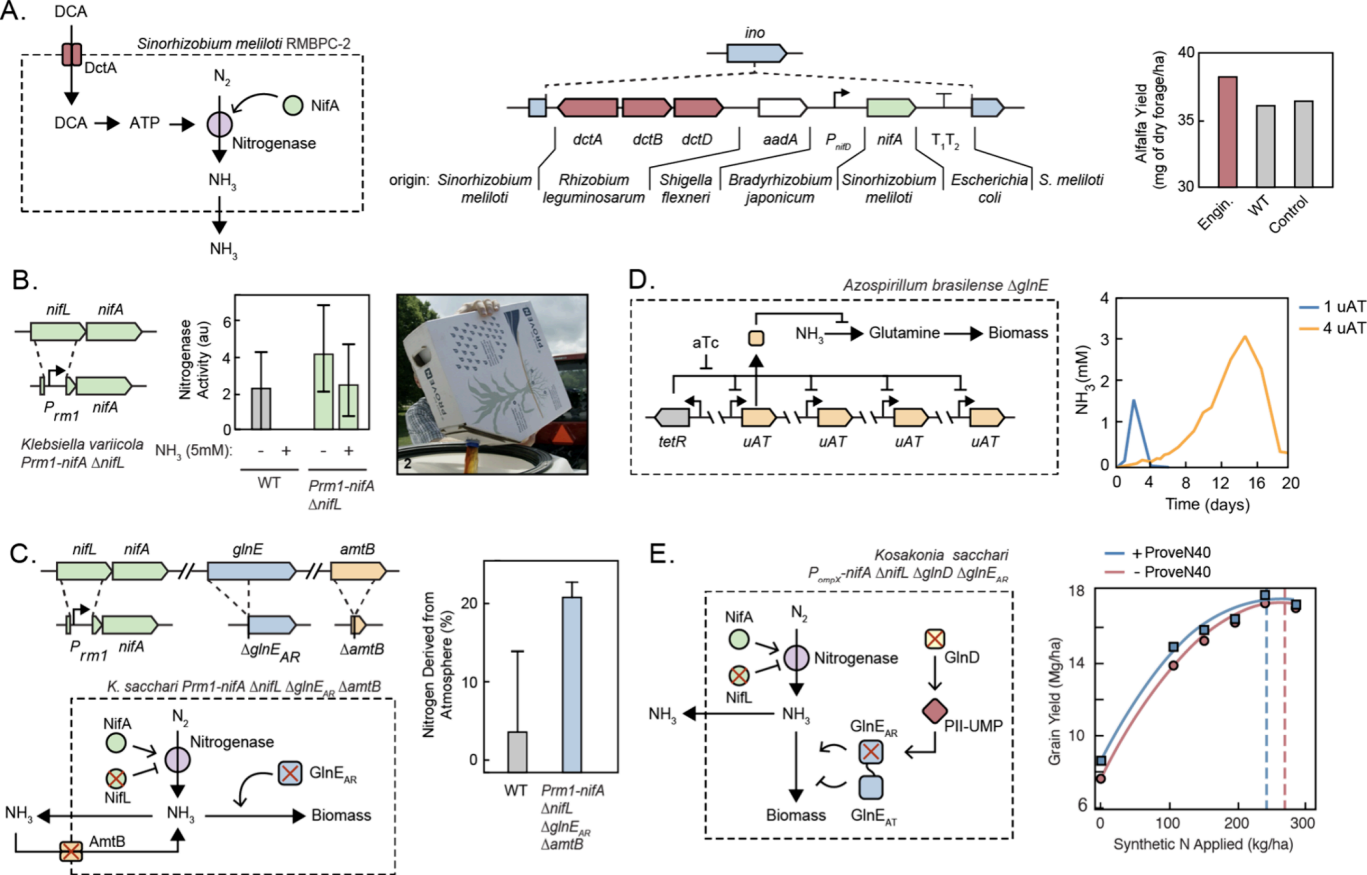
**Continuous N production by engineered N-fixing species**. **A**. Delivery of ammonia to alfalfa by engineered *Sinorhizobium meliloti*.^3291^ A large cassette was inserted into the *ino* locus that included the upregulation of the NifA activator and the expression of a transporter for C4-dicarboxylic acid (DCA). This engineered strain (Engin.) produced higher yields than wild-type (WT) and the uninoculated control. **B**. Remodeled *Klebsiella variiocola* to deliver fixed nitrogen to maize.^2326,3280^ The two genetic changes were made by moving a copy of P_rm1_ from within the genome to disrupt the negative regulator of nitrogenase *nifL* and upregulate activator *nifA*. The remodeled strain (P_rm1_-*nifA* Δ*nifL*) displayed higher nitrogenase activity both with and without excess ammonia in media. The picture shows media formulation being added to a seed inoculant mixture. Image of ProveN product reproduced with permission from ref 3280. Copyright 2021, American Chemical Society. **C**. Remodeled *Kosakonia sacchari* to deliver ammonia to maize.^2326,3292^ The remodeled strain was constructed through deletions (dashed lines) and native sequence duplication (P_rm1_). A partial deletion of the *glnE* gene was performed such that only the adenyl-removing domain (AR) was deleted, leaving the adenyl-transferase (AT) domain intact. In greenhouse studies, maize inoculated with the remodeled strain had more nitrogen in plant biomass derived from the atmosphere than maize inoculate with the WT (wild-type) bacteria. **D**. Engineered cereal endophyte *Azospirillum brasilense* to secrete ammonia.^3287^ The native regulator of glutamine synthetase GlnE was mutated and either 1 or 4 copies of its adenyl-transferase domain (uAT) were inserted under aTc-inducible control to suppress glutamine synthetase activity. Ammonia secretion into the media is shown over time. **E**. Remodeled *Klebsiella variiocola* and *Kosakonia sacchari* (ProveN40) reduces the agronomic optimum N fertilizer rate in maize. The vertical dashed lines represent the amount of synthetic N required to achieve the maximum yield with and without ProveN40. The commercial product ProveN40 combines genetic changes to *Kosakonia sacchari* to derepress nitrogenase (P_ompC_-*nifA* Δ*nifL*) and disrupt NH_3_ assimilation (Δ*glnE*_*AR*_) with a deletion to the nitrogen regulator GlnD. The *Klebsiella variiocola* strain possesses similar genome manipulations (P_ompC_-*nifA* Δ*nifL* Δ*glnD*). At low glutamine conditions, GlnD upregulates NH_3_ assimilation via the PII signaling protein.

Because intergeneric parts lead to tough regulatory hurdles, “remodeling” was developed to achieve a desired functional effect with the restriction of only using DNA native to the genome (Section 3.3.2.2). *Klebsiella variicola* was isolated from maize roots and has the *nif* gene cluster, but it is repressed under the conditions when needed (Figure 45B).^2326^ The strong constitutive promoter P_rrn_ was copied to a position internal to the *nifL* repressor, which both knocked it out and upregulated the downstream *nifA* activator. In addition, the N-terminal domain of GlnE was removed. A similar process was applied to *Kosakonia sacchari*, a strain isolated from the sugarcane rhizome that can fix nitrogen but does not naturally when inoculated with cereals. In addition, two genes were disrupted: an ammonia importer gene (*amtB*) and one involved in nitrogen assimilation (*glnD*) (Figure 45C).^2326,3280^ Both bacteria produce nitrogen and consume different carbon sources excreted from the root.^3281^ Together, these strains have been commercialized as PROVEN40 by Pivot Bio (Figure 45E).^3280,3282^

Engineered strains may not excrete fixed nitrogen because it rapidly enters cellular metabolism. Inactivating glutamine synthase, an enzyme responsible for nitrogen assimilation, increases ammonia excretion.^3283^ It is lethal when knocked out but can be placed under inducible control.^3284^ Glutamine synthase is activated by GlnE through deadenylation.^3285^ Modifying GlnE to remove this activity leads to constitutive ammonia secretion.^2133,3286^ However, when secreting ammonia, mutations quickly disrupt the activity. The time before mutation increased to 32 days when multiple copies of the engineered pathway were carried (Figure 45D).^3287^

Archaea also occupy the rhizome and fix nitrogen, notably species that reside in the water of rice fields.^3288–3290^ However, far less is known about the nitrogen fixation pathways in these organisms and genetic tools are behind those of bacteria. They are a potential target for optimization as these tools develop (Section 3.7.1). CRISPRi has been used to turn off nitrogen fixation genes in the archaeon *Methanosarcina acetivorans*.^2883^ This methane-producing species is found in oil wells and trash dumps.

#### 4.2.2 *nif* Transfer to Cereal-Associated Bacteria

There are many bacterial species that adhere to cereal roots or are in close proximity in the rhizosphere. Most of these species do not have the genes necessary to fix nitrogen. One strategy is to move these genes into an organism that already is used as a cereal inoculant (*e.g.*, used as a plant-growth-promoting bacterium) or is known to coat roots or enter them and survive (endophyte).^3293^ The possibility of functionally moving the *nif* gene cluster as a whole is inspired from work in 1972 where the 25 kb cluster was moved from *Klebsiella oxytoca* to *Escherichia coli*, an incredible feat at the time.^3294^ This work has inspired subsequent experiments to transfer many *nif* clusters from many species into strains of agricultural interest. A challenge with commercializing these strains is that they are recombinant, to various degrees, and more difficult to obtain regulatory approval than remodeled strains or even GMO crops.

*Pseudomonas protegens* Pf-5 is used as a corn inoculant in agriculture and has many benefits, including disease suppression, plant-growth promotion, and improved nutrient uptake. The nitrogenase cluster from *Pseudomonas stutzeri* has been functionally moved into it.^2027,3295–3297^ *Pseudomonas protegens* Pf-5 has also been shown to fix nitrogen when the *nif* clusters from *Klebsiella oxytoca* and *Azotobacter vinelandii* were transferred.^2027^ *Rhizobium* sp. IRBG74 is a rice endophyte that enhances growth and yield. While it forms a N-fixing symbiosis with a legume, it does not turn these genes on when inside a cereal root. It was engineered to fix nitrogen under free-living conditions by transferring complete *nif* clusters from different Rhizobia.^2027^ Paenibacillus has one of the smallest *nif* gene clusters, encoded as a single 11 kb operon with 9 genes. This size makes it easier to transfer. XPORT (Section 3.2.3) was used to functionally transfer this operon into *Bacillus licheniformis, Bacillus amyloliquefaciens, Bacillus methylotrophicus*, and *Bacillus pumilus*.

Refactoring is a means by which a gene cluster can be re-organized, making it easier to engineer and transfer to new species (Section 3.3.3.2).^2141,2142^ The objective is to strip out all native regulation, including that which is unknown, and reorganize the cluster so that it is a set of well-defined parts. This approach was initially applied to the *Klebsiella oxytoca nif* gene cluster.^1462^ Refactored gene clusters have been transferred between bacterial species.^1438,2027^ Refactoring is required for any transfer of *nif* genes to eukaryotes, as the regulatory encoding is more different.^3298^ Refactoring also simplifies changes to the genetic parts, for example, to scan an RBS library to optimize expression levels. These changes have been made to build large libraries of *nif* pathways to optimize activity in a new host.^3299^

A challenge with making a *nif* cluster constitutively active is that it imposes a high burden on the cell carrying it. This burden leads to rapid loss of the strain in the environment^3295^ and can put evolutionary pressure on a genetic system to break by mutating. A way to address this problem is to place the system under the control of synthetic sensors and circuits (Section 3.4.3). To do this to a native *nif* cluster, the natural regulator (NifA) was knocked out and placed under the control of the desired sensor. NifA is sensitive to ammonia levels, but mutants can be made to eliminate most of this repression.^3300,3301^ This approach was taken to make the *nif* clusters in *Pseudomonas putida* Pf-5, *Azorhizobium calinodans*, and *Azotobacter vinelandii* under the control of phytohormones and agrochemicals.^2027^ Further, they could be placed under the control of artificial root exudates produced by introducing recombinant genes into plants, including rhizopines and octopines (Section 4.2.4). These sensors act as proximity sensors, where only bacteria close to the root fix nitrogen, thus decreasing the burden on those strains further away.^2156^

#### 4.2.3 Engineering Crops to Encourage Natural N-Fixers

Plants release up to 50% of the carbon fixed by photosynthesis into the soil by exuding it from their roots.^1659^ Microbes that can utilize these forms of carbon become enriched in the root microbiome (Section 3.1.6).^3302–3304^ Not all bacteria and plants are compatible in this regard. Some plants do not produce an exudate needed by the bacterium. To make a bacterium and plant compatible, either the plant needs to be engineered to make the required exudate or the bacterium needs to be engineered to consume an exudate made by the plant.

A N-fixing bacterium can be engineered to include a recombinant catabolic pathway to consume a carbon exudate of the desired crop. *Azospirillum brasilense* Sp7 can fix nitrogen and produces growth-promoting phytohormones. However, it is a poor colonizer of rice roots due its inability to eat D-glucose, which can make up >90% of rice root exudates. Transferring five D-glucose catabolism genes into *Azospirillum brasilense* Sp7 enabled glucose utilization and led to a 15-fold improvement in rice colonization.^3305^

Plants can be engineered to encourage colonization by specific desirable bacteria in other ways. Rice has been engineered to excrete larger amounts of the natural flavone apigenin.^1797^ A chemical screen discovered that the flavonoid apigenin was a potent biofilm stimulant for the nitrogen-fixing *Gluconacetobacter diazotrophicus*.^1807^ Rice engineered to overproduce apigenin and inoculated with *G. diazotrophicus* had had increased biofilm formation and nitrogenase activity, likely due to the biofilm mediating root attachment and protecting the enzyme from oxygen.

#### 4.2.4 Engineering Crops to Form Symbiotic Relationships with N-Fixers

Symbiosis occurs when two species are in a relationship where resources produced by one are consumed by the other and vice versa. It can manifest as commensalism or mutualism, where either one or both species benefit from the relationship. There are numerous natural examples of both types of relationships in agriculture, including between microbial species and between microbes and plants. The former help stabilize mixed communities of bacteria by maintaining constant ratios between members and improving resilience to perturbations (Section 3.4.7). There are innumerable natural symbiotic relationships between bacteria and plants, where many bacteria consume the carbon in root exudates (sugars, organic acids, etc.) and provide nutrients or protective functions to the plants (Section 3.1.1).^3306–3308^

Nitrogen fixation is a classic mutualistic relationship. Bacteria provide fixed nitrogen to the plant and are rewarded with a carbon source. Symbiosis can be enhanced by specialized plant organs (*e.g.*, a legume root nodule or aerial root of corn.^1815^ It could also occur with bacteria that are non-specifically attached to the root or in the soil. The previous sections have focused on harnessing natural symbiosis. Here, we focus on two approaches. The first is to transfer the nodule-formation genes into a cereal so that they can form the specialized nodules to recruit bacteria that naturally associate with legumes.^3309^ The second is to create synthetic symbiosis where the plant provides the microbe with a new carbon source not naturally excreted from the roots.

##### 4.2.4.1 Nodule Formation in Cereals

Designing a cereal that builds a nodule *de novo* would require control over plant morphology beyond what is currently possible.^3309^ However, it is simpler when the target plant has most of the pathway already and is only missing easily identifiable components. Cereal roots develop specialized interactions with AMF and there are commonalities to the signaling pathway for rhizobia to interact with cereals (Figure 46A).^3310,3311^ In fact, rice signaling proteins from this pathway fully complement knockouts in the legume *Lotus japonicus* (Figure 46B).^3312^ This result implied that adding missing genes to rice could cause nodules to form.

**Figure 46:**
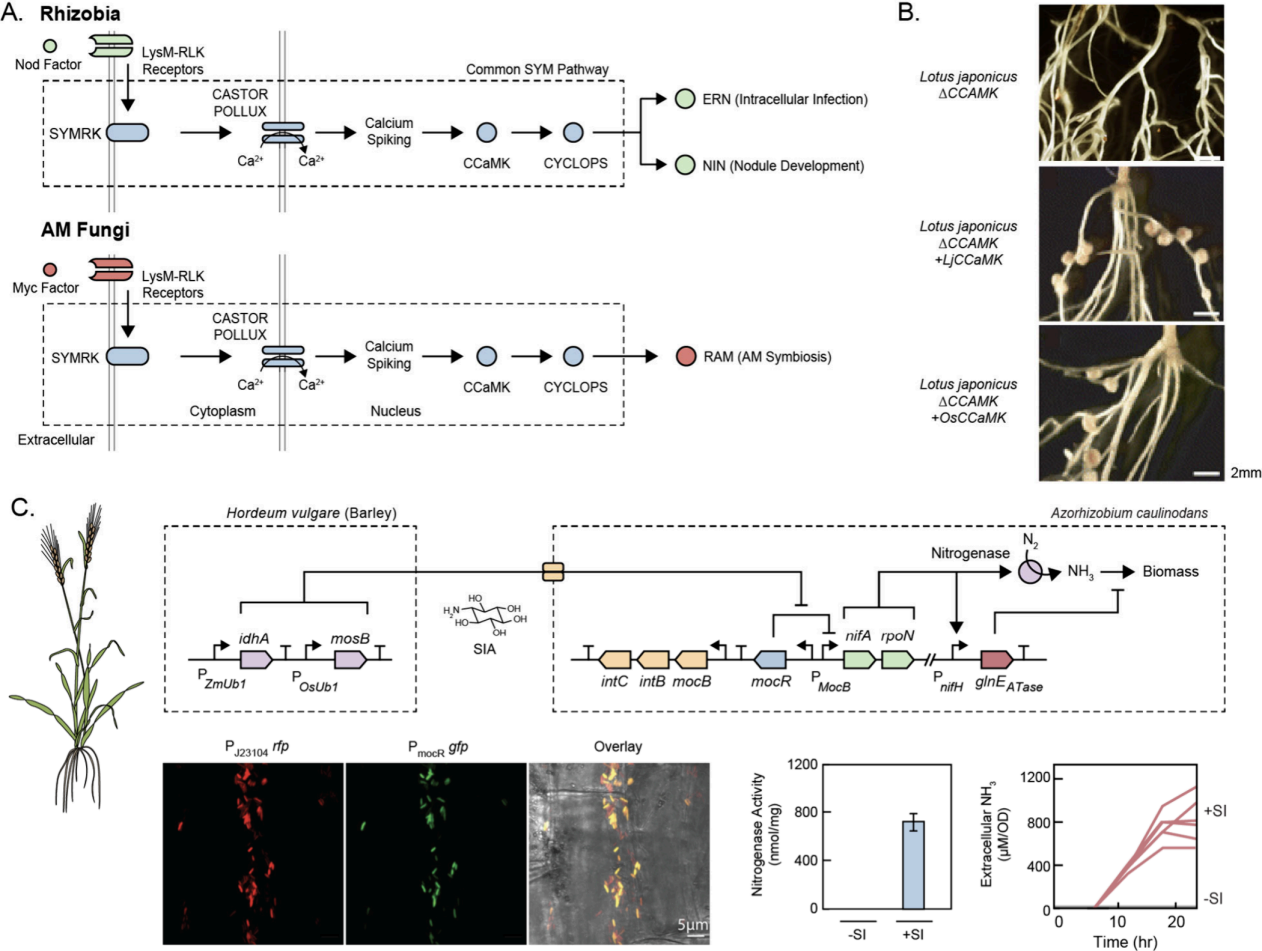
**Engineering cereals symbiosis with N-fixing bacteria**. **A**. Comparison of the legume pathway for establishing interactions with rhizobial bacteria with the cereal pathway establishing interactions with AMF. The dashed lines indicate the shared signaling components of the two pathways. The signals are received by SYMBIOSIS RECEPTOR KINASE (SYMRK) and the output is CYCLOPS, which controls the expression of master regulators of the plant responses. **B**. Legume (*Lotus japonicus*) knockouts of the core pathway (dashed box) in A can be complemented with the expression of the homologs (LjCCaMK or OsCCaMK) from rice.^3312^ Images reproduced with permission from ref 3312. Copyright 2008, Plant & Cell Physiology. **C**. An engineered symbiotic relationship between barley and *Azorhizobium caulinodans*. A barley plant expresses two genes to produce SI.^2156^ *A. caulindoans* was engineered to sense SI and induce nitrogen fixation and ammonia release. The engineered strain is shown colonizing the roots of the transgenic barley, which induces a SI sensor (P_mocR_ output promoter) *in vivo*. The picture of a barley root shows RFP as the bacterium (expressed from the constitutive promoter P_J23104_), and the output of the SI sensor is GFP. The nitrogenase activity and NH_3_ production of engineered *A. caulinodans* cultures was quantified with and without 10 μM SI. Microcopy images reproduced with permission from ref 2156. Available under a CC BY license. Copyright 2022, National Academy of Sciences.

Thus, engineering efforts have focused on introducing genes into cereals to recognize the bacteria, activate the SYM pathway, and govern organogenesis and infection.^3313^ Nodulation is initiated through the exchange of signaling factors (Nod-factors) by *Rhizobia* and flavonoids by legume roots. When exposed to Nod-factors, transgenic rice expressing nod-factor receptors and their receptor kinases from the legume *Medicago truncatula* developed root hair deformities similar to those responsible for the entrapping rhizobia in legumes.^3314^ Likewise, an intracellular calcium spiking response, a signaling step in the nodulation pathway, was observed in rice lines expressing chimeric receptors containing *Medicago* nod-factor receptors fused to receptor kinases involved in rice-AMF symbiosis.^3315^

Successfully transferring downstream components of the legume symbiosis pathway has proven more difficult. Eight essential rhizobia symbiosis genes from *Medicago truncatula*, including NFRPs, NFRKs, nuclear calcium channels, and nodulation-associated transcription factors, were transformed into a variety of non-legumes, but neither a change to root morphology nor induction of the *Medicago truncatula* nodulation reporter gene MtNIN was observed.^3316^

##### 4.2.4.2 Synthetic Symbiosis

Plants naturally feed microbes in the rhizosphere by excreting a cornucopia of carbon sources, including organic acids and sugars. Bacteria near the roots can catabolize these molecules, some of which provide beneficial functions to the plant. When introducing a synthetic bacterium, it would compete with the natural consumers of these molecules. To tighten the symbiotic relationship, the plant could be engineered to produce molecules not normally found in the rhizosphere that can only be catabolized by the engineered bacterium.^1815^ If the bacterium provides a benefit to the plant, such as the release of fixed nitrogen, this completes the mutualism. A benefit is that all the carbon that is released is only available to the engineered bacterium. A second benefit is that the bacterium can be made reliant on that as a carbon source, making it unable to associate with weeds or other plants and when the crop is removed from the field, the bacteria would die.

Plants can be engineered to secrete new exudates designed to enrich bacteria engineered to consume it. This ensures that nitrogen fixation only occurs when close to the GMO plant roots. These systems contain two parts: 1. a pathway introduced into the plant to make a chemical to be excreted and 2. a genetic sensor in the bacteria that respond the chemical and turn on nitrogen fixation. Various systems have been developed including opines (Arabidopsis: *Agrobacterium rhizogenes*)^1795^ and rhizopines (barley: *Azorhizobium caulinodans, Rhizobium leguminosarum*) (Figure 46C).^2156,3207,3317^ The bacteria can be engineered to contain the catabolic pathway for the consumption of these synthetic exudates. When *Pseudomonas fluorescens* was engineered to catabolize opines, this species became enriched in the rhizosphere.^1795^ Placing the catabolic genes under the control of a sensor ensures that they only turn on in the presence of the exuded chemical.^3207,3318^

#### 4.2.5 Direct Transfer of *nif* to Cereal Crops

One of the first grand challenges of genetic engineering was to move the nitrogen fixation genes from a bacterium to a cereal crop.^3319^ Over 50 years later, the feat remains unmet for several reasons. Nitrogenase is a dynamic enzyme with a complex electron transport chain and high energy (ATP) requirements. Its function is due to a complex co-factor (FeMoCo) that is difficult to synthesize and requires precursors whose accessibility in the plant is uncertain. FeMoCo’s insertion into the apoprotein requires chaperones in a process that is sensitive to expression levels. Finally, nitrogenase is stringently oxygen sensitive, thus limiting where and under what conditions it can be expressed. There are also many genes that need to be co-expressed that have been difficult to do so in a plant due to the size and number of genetic parts needed and insolubility of the proteins. Still, there have been advances toward the goal of having a nitrogen-fixing plant that does not require an association with bacteria to do so.

A choice that must be made is where nitrogenase should be expressed. One option is the chloroplast, which is desirable because it has a prokaryotic-like regulation, thereby making it easier to express a BGC with fewer changes to the genetic parts. It is also likely to have the electron transport machinery and iron-sulfur co-factors required for FeMoCo biosynthesis. The challenge is that photosynthesis occurs in chloroplasts, leading to high O_2_ tension, which inactivates nitrogenase. In theory, this problem could be addressed by expressing nitrogenase at night or in non-photosynthetic differentiated chloroplasts (*e.g.*, amyloplasts).

Bacterial *nif* genes can be integrated directly into the genome of the plastid using plastid genetic parts (Section 2.2.1.5). Homocitrate synthase (NifV) is important for making functional FeMoCo. *Klebsiella oxytoca* NifV was expressed in the chloroplast of the eukaryotic algae *Chlamydomonas reinhardtii*.^3320^ NifB is also critical for FeMoCo production and it, along with NifU and FdxN, where expressed from the plastic and NifU was able to insert [Fe_4_S_4_] clusters into NifB, whose activity was confirmed *in vitro* using a cell-free enzymatic assay.^3321^ The central component of nitrogenase (NifH) was expressed in the nucleus and targeted to tobacco chloroplasts and it was found that it required NifM, NifU, and NifS to be co-localized for it to be soluble and functional *in vitro*.^3322^ In these cases, the *nif* genes were selected from *Azotobacter vinelandii*, in part because of its ability to fix nitrogen under aerobic conditions relative to other species.

Mitochondria are also possible targets for expressing functional nitrogenase. They are the “powerhouse” of the cell and can produce the high concentrations of ATP and NADH required. Because this organelle is where respiration is occurring, they have a very low O_2_ concentration. They also make the essential [4Fe-4FS] clusters needed by the *nif* electron transport chain. While the mitochondria have their own genome, it is very hard to engineer and genes have not been inserted into it in plants. Thus, the nitrogenase components would need to be expressed in the nucleus and transported to the mitochondria using transport tags. This step can result in misfolding or mis-integration of co-factors, and can be more difficult to control relative expression levels of components.

Yeast are much easier to engineer than plants, so it has been used as a surrogate host to test the targeting of Nif proteins to mitochondria. It was shown that mitochondria-targeted NifH and NifM could accumulate in *Saccharomyces cerevisiae* and can incorporate native mitochondrial Fe-S clusters under aerobic conditions.^3323^ Expression in the cytosol required anoxic growth conditions demonstrating the advantage of the low-O_2_ conditions of the mitochondrion. A combinatorial library of 96-monocistronic expression cassettes constructed from 9 refactored *Azotobacter vinelandii nif* genes and various combinations of promoters, terminators, and mitochondrial transit tags was expressed in yeast. Cofactor biosynthesis enzymes (NifB) from 28 species were screened to identify a set that remained functional when co-expressed with NifS, NifU, NifX, and fdxN from *Azotobacter vinelandii*.^3324^ A polyprotein strategy was developed for yeast where groups of *nif* proteins were expressed as single proteins separated by recognition sites for yeast mitochondrial-processing peptidases, which processed into monomers in the mitochondria.^3325^

Individual components of the *nif* pathway have been expressed in rice and targeted to the mitochondrion, where they have been found to be functional. NifH was fused to a mitochondrion-targeting peptide and once in the organelle it was able to associate with its [4Fe-4S] co-factor.^3326^ However, the NifH from *Hydrogenobacter thermophilus* was found to be insoluble in this environment when targeted by itself and had to be co-localized with NifM from *Azotobacter vinelandii*. The isolated NifH was found to be functional *in vitro* and transfer electrons to NifDK, which is where fixation takes place. It was observed that [Fe-S] cluster availability in the mitochondrion was limiting. NifB, which catalyzes the first step in FeMoCo biosynthesis, has also been transferred. Archaeal NifB’s from *Methanocaldococcus infernus* and *Methanothermobacter thermautotrophicus* were found to accumulate in rice mitochondria in functional form.^3327^ In tobacco, NifB from *Azotobacter vinelandii* was co-expressed with NifU and FdxN and the [Fe_4_S_4_] clusters produced by NifU were found to integrate into NifB, rendering it functional.^3321^

#### 4.2.6 Artificial Leaf

A problem with the Haber-Bosch process is that it is difficult to scale-down economically, requiring large production facilities. It also requires an input of natural gas. These issues are addressed by the so-called “artificial leaf,” which combines a photovoltaic cell with an engineered bacterium.^3283^ Hydrogen is generated by the green electricity derived from the photovoltaic cell. The bacterium *Xanthobacter autotrophicus* is able to take the H_2_ stream and use it as an energy source to fix N_2_ and CO_2_. These products accumulate as ammonia and biomass. The bacteria can either be wild-type or engineered to make complex products, such as vitamins, or theoretically other beneficial agrochemicals.^3328^

The bacteria can also be collected and applied to crops, similar to the spreading of synthetic nitrogen. One can imagine farm-scale bioreactors generating fertilizer in this way. This approach has the potential benefit of being able to put a lot of nitrogenous fertilizer on the ground quickly, which could be advantageous at the end of a growth season when high levels are needed to maximize yield and biological routes may be slow. However, there is also the potential for nitric oxide production and run-off if the fertilizer is used early in the growth season when the crop has not developed sufficiently to quickly take up the nutrients.

## 5 Discussion

This review encompasses the impact of Synthetic Biology across the agricultural system. Considering both the crop plant as well as the microbes holistically as part of the system, collectively they contain over 100 gigabases of DNA.^14,15^ We are far from being able to design systems at this scale, but genetic engineering advances are moving forward in complexity, scale, and the number of organisms simultaneously engineered as part of a project. System integration, CAD, and AI will enable agricultural design far beyond what is currently possible or even imaginable. It is amazing to consider that these integrated systems could be encoded on a single treated seed, with microbes and supporting insects emerging with the growth of the plant. It also allows for a rational approach to engineering challenges. For example, develop a firm quantitative basis for selecting the organism that is the best for carrying a particular function, such as nitrogen fixation, which is now *ad hoc* or based on the species specialties of a particular lab. Agriculture is also changing, where there is increased indoor farming, use of hydroponics, and alternative crops, such as ocean-grown seaweed or crops specialized for space travel.^3329–3333^

Predicting the performance of a plant or microbe before field trials remains a challenge. This problem becomes more acute when the system involves not only a plant, but many additional bacteria or fungi in one system. Measurements in the laboratory or even greenhouse systems are notorious for not being predictive of how engineered organisms will function in the field.^3334^ This problem is similar to that observed in industrial biotechnology, where the scale-up of microbial processes from the bench to large bioreactors is fraught with failures.^2187,3335,3336^ Metabolic pathways can lead to unreliable product yields and genetic circuits can break as the growth conditions change during scale-up.

To address these problems, there have been efforts to scale-down soil microsystems to better replicate diverse field conditions in the laboratory.^3337–3341^ These systems can include the effects of weather, insects, plants, and soil types. They can be scaled down even further for high-throughput screening. For example, the EcoFAB uses robotic systems to rapidly visualize the impact of soil bacteria colonizing plant roots.^3342,3343^ The further development high-throughput imaging, coupled to modern -omics techniques, could improve the reliability of prototyping complex and highly-engineered agricultural systems before field testing. In addition, the approvals process could be helped through the development of new engineering approaches to control microbial persistence in the environment and methods to prototype their performance and quantify the risk-of-failure before field trials.^6,1672,1673^

Traditional land-based agriculture has reached the maximum amount of farmable area.^3344–3346^ While yield increases over the last hundred years have been impressive, it is hard to imagine they will continue to grow to match the needs of rapid population growth. Further, farming has both an ecological impact and is a major contributor to climate change, by releasing about 25% of human-produced greenhouse gases.^3347,3348^ Indoor agriculture will require a new generation of highly-engineered plants as well as the engineering of microbes to obtain proteins, fats, vitamins as well as the flavors and fragrances that make up foods.^3349–3353^ Originally used to generate simple ingredients, cellular production systems can produce more complex plant- and animal- derived foods, including human milk and artificial meat.^3350,3354,3355^ For these products, if the bioreactors are fed with sugar and Haber-Bosch derived ammonia, this just pushes the problem to other sectors. Solving this issue will require new approaches, whether they are biological or electrocatalysis, to directly feed the bio-reactors by fixing CO_2_ and N_2_, respectively as feedstocks.^3356–3358^ Some companies are already moving entirely to a CO_2_-to-food process, bypassing land-based agriculture entirely.^3359,3360^

The coming decades are poised to bring transformative advances in crop performance through Synthetic Biology. The crops of tomorrow will require fewer inputs to achieve greater yields by employing complex, engineered metabolic pathways (nitrogen fixation and improved photosynthesis) while sensing, reporting, and responding to harsh environmental conditions, pests, and disease. Bioenergy crops like maize and sugarcane currently grown for bioethanol will be engineered to produce higher-value outputs, including secondary metabolites, nutritious foods and animal feeds, and therapeutics; this will boost the value of these crops for farmers despite waning demand for biofuels. Plant architecture will become optimized using engineered genetic circuits that consider environmental conditions and grower needs. Hybrid crops that have revolutionized agriculture in the industrialized world will reproduce asexually (apomixis), making elite hybrid lines, and their associated increased yields, accessible to farmers across the globe.^3361^ To realize these developments, plant synthetic biologists will need to overcome key technical obstacles bottlenecking the next generation of these biotechnologies (Table 6). The long generation time of agriculturally-relevant crops means making iterative improvements to engineered systems infeasible and a lack of efficient genome editing tools and regeneration protocols makes large, combinatorial engineering efforts impractical. Unlike many microbial systems, it is not yet possible to use well-defined design rules and CAD software to design a functional genetic system in one go. A suite of new synthetic biology tools and strategies will be required to accelerate plant engineering efforts that lead to novel and complex agronomic traits.

**Table 6:** Technical Challenges Limiting Agricultural Synthetic Biology

Goals	Technologies Needed
Accelerate plant engineering cycle times	1. Cell lines and model plants with rapid (<week) generation times
	2. Automated, high-throughput systems for the parallel evaluation of plants and plant-microbe interactions
	3. Validation of cell-free protein systems (CFPSs) for genetic system prototyping
	4. AI/ML methods to predict field performance based on laboratory data
Organelle genome engineering	1. Chloroplast genome engineering in cereal crops
	2. Mitochondrial genome engineering in any plants
	3. Organelle genome complete synthesis and replacement
	4. Circuits to rapidly drive systems to homoplasty
	5. Programmable differentiation of specialized DNA-containing organelles in defined tissue/cell types
	6. Creation of specialized organelles to sequester toxic products or serve as the “central processing unit” for computation
Nuclear genome engineering	1. Rapid site-specific integration of large DNA sequences (100 kb+)
	2. Whole chromosome synthesis and replacement
	3. Artificial chromosomes that are stable and easily manipulated
Plant genetic parts and devices	1. Biophysical models for genetic part design
	2. Quantitative understanding of the interference between parts within an expression unit; insulation methods to reduce it.
	3. Implementation of complex logic via rules for CRE addition to promoters up to 5kb
	4. Genetic circuit design automation software based on simpler logic operations that can be layered
	5. Standards for genetic part characterization
	6. Method to send regulatory signal from chloroplast to nucleus
Computer aided design	1. Plant CAD with dedicated design tools that integrate the construction of synthetic regulatory networks, metabolic control, inter-organelle signaling and growth/differentiation.
	2. Predictive consortia GEMs encompassing communities of bacteria, fungi and the plant
	3. Application of plant GEMs to metabolic engineering
	4. Genetic circuit design automation for circuits carried in the plant chromosomes, plastid genome, and soil bacteria/fungi
	5. Linkage of growth/morphology/phenotype algorithms with predictions of the performance of synthetic systems
	6. Prediction of the impact of recombinant systems on microbial growth and their evolutionary stability in the field
Microbial parts and devices	1. Control theory applied to develop regulatory control to make bacterial sensors perform reliably in the field
	2. Ability to measure gene expression from plants remotely
	3. Synthetic symbiotic interlocking of plants to microbes and microbes within a consortium
	4. Genetic control of microbial survival in the field, tunable from days to years
	5. Improved tools for the engineering of agriculturally-relevant Archaea
	6. Transformation and genetic manipulation of AMF
	7. Creation of libraries of parts that come from within the same genus that can be used with fewer regulatory hurdles
	8. Devices to link the survival of a microbe to the presence of a specific engineered plant
Communication and control in the field	1. Methods to induce plant gene expression in the field without chemicals
	2. Ability to measure gene expression from microbes below the soil surface remotely
	3. Methods to control gene expression in plants and microbes using UAVs/satellite
Microbial discovery	1. Global strain isolation from agricultural fields and crops, sequencing, strain banking, and systematic screening of engineering tools
	2. Isolation, characterization, and genetic tools for agriculturally-relevant archaea
	3. Full genome sequences of filamentous fungi relevant to agriculture, particularly AMF
Crop Grand Challenges	1. Complete replacement of Haber Bosch nitrogen, involving plant/microbe engineering and addition of bio-manufactured “green” nitrogen
	2. “Smart plant” that can receive dozens of signals, process this information with synthetic circuits, and control stress responses, traits, and plant growth.
	3. Improvement of photosynthetic efficiency of crops to >8%
	4. Apomixis (asexual reproduction) of cereals so that they can maintain desired hybrid traits over generations without cross breeding
	5. Methods to reduce methane emissions from Archaea, particularly in rice paddies

Synthetic Biology has progressed in microbes to the point where nearly any plant-derived product can be produced in the fermenter and obtained in this way.^3362^ The pathways for complex plant-derived medicines have been moved to production strains, including precursors to cancer chemotherapeutics (*e.g.*, taxol), pain killers (*e.g.*, opioids), and antimicrobials (*e.g.*, artemisinin).^3363–3367^ Dozens of plant genes can be combined from enzymes of other species into one cell that converts sugars to these high-value pharmaceuticals.^3368,3369^ Complex plant-derived flavors and fragrances can also be produced, such as sweeteners (*e.g.*, steviol glycosides), vanillin, and saffron.^3370–3372^ These further reduce the need to grow plants in the field, and as feedstocks move from sugar to CO_2_, even the feedstock can be derived from air.

Beyond agriculture, there are many applications for highly-engineered plant-microbial communities. They have already been used as bio-sensors in the field, for example to detect landmines^2411^ or to identify toxins in the soil.^3373^ They have been used for bio-remediation, to sequester heavy metals or degrade toxins.^3374^ Plants have been used to mine metals from soils.^3375–3377^ Miles-long berms protect communities from the ocean can be stabilized.^3378^ Note, however, that these examples are already being done at some level that would be improved by the integration of engineering tools described in this review. The potential applications are fantastical, expanding on the most forward-thinking science fiction. At the dawn of the Synthetic Biology era, architects and artists envisions houses and cities being grown from seeds.^3379^ While this verges on hyperbole, it captures the fact that we do not fully grasp what is possible when we have full engineering control over plants and their microbial supporters.

## References

[1] Ray D. K., Ramankutty N., Mueller N. D., West P. C., Foley J. A. (2012). Recent
patterns of crop yield growth and stagnation. Nat Commun.

[2] von
Braun J. (2018). Bioeconomy - The global trend and its implications for sustainability
and food security. Glob Food Secur-Agr.

[3] ISAAA . Global Status of Commercialized Biotech/GM Crops in 2018: Biotech Crops Continue to Help Meet the Challenges of Increased Population and Climate Change. ISAAA Brief 2018, 54.

[4] Long S. P., Marshall-Colon A., Zhu X.-g. (2015). Meeting the Global Food Demand of
the Future by Engineering Crop Photosynthesis and Yield Potential. Cell.

[5] Food and Agriculture Organization of the United Nations . The future of food and agriculture: Trends and challenges; 2017.

[6] Chemla Y., Sweeney C. J., Wozniak C. A., Voigt C. A. (2024). Engineering Bacteria
for Environmental Release: Regulatory Challenges and Design Strategies. Authorea Preprints.

[7] Yang X., Medford J. I., Markel K., Shih P. M., De Paoli H. C., Trinh C. T., McCormick A. J., Ployet R., Hussey S. G., Myburg A. A. (2020). Plant
Biosystems Design Research Roadmap 1.0. BioDesign
Research.

[8] Savary S., Willocquet L., Pethybridge S. J., Esker P., McRoberts N., Nelson A. (2019). The global burden of pathogens and pests on major food
crops. Nature Ecology and Evolution.

[9] Sultan B., Defrance D., Iizumi T. (2019). Evidence of
crop production losses
in West Africa due to historical global warming in two crop models. Scientific Reports.

[10] Carter, S. R. ; Rodemeyer, M. ; Garfinkel, M. S. ; Friedman, R. M. Synthetic biology and the US biotechnology regulatory system: Challenges and options; J. Craig Venter Institute, Rockville, MD (United States), 2014.

[11] Baltes N. J., Voytas D. F. (2015). Enabling plant synthetic biology
through genome engineering. Trends Biotechnol.

[12] Wurtzel E. T., Vickers C. E., Hanson A. D., Millar A. H., Cooper M., Voss-Fels K. P., Nikel P. I., Erb T. J. (2019). Revolutionizing
agriculture with synthetic biology. Nat Plants.

[13] Liu W., Stewart C. N. (2015). Plant synthetic
biology. Trends
in Plant Science.

[14] Eckardt N. A. (2000). Sequencing
the rice genome. Plant Cell.

[15] Frisli T., Haverkamp T. H., Jakobsen K. S., Stenseth N. C., Rudi K. (2013). Estimation
of metagenome size and structure in an experimental soil microbiota
from low coverage next-generation sequence data. J Appl Microbiol.

[16] Knott G. J., Doudna J. A. (2018). CRISPR-Cas guides the future of genetic engineering. Science.

[17] Baltes N. J., Voytas D. F. (2015). Enabling plant synthetic
biology through genome engineering. Trends Biotechnol.

[18] Puchta H., Dujon B., Hohn B. (1996). Two different but related mechanisms
are used in plants for the repair of genomic double-strand breaks
by homologous recombination. P Natl Acad Sci
USA.

[19] Lloyd A., Plaisier C. L., Carroll D., Drews G. N. (2005). Targeted mutagenesis
using zinc-finger nucleases in Arabidopsis. P Natl Acad Sci USA.

[20] Mahfouz M. M., Li L., Shamimuzzaman M., Wibowo A., Fang X., Zhu J. K. (2011). De novo-engineered
transcription activator-like effector
(TALE) hybrid nuclease with novel DNA binding specificity creates
double-strand breaks. Proc Natl Acad Sci U S
A.

[21] Shan Q., Wang Y., Li J., Zhang Y., Chen K., Liang Z., Zhang K., Liu J., Xi J. J., Qiu J. L. (2013). Targeted genome modification of crop plants using a
CRISPR-Cas system. Nat Biotechnol.

[22] Reiss B., Schubert I., Kopchen K., Wendeler E., Schell J., Puchta H. (2000). RecA stimulates sister
chromatid exchange and the fidelity
of double-strand break repair, but not gene targeting, in plants transformed
by Agrobacterium. Proc Natl Acad Sci U S A.

[23] Gao H., Smith J., Yang M., Jones S., Djukanovic V., Nicholson M. G., West A., Bidney D., Falco S. C., Jantz D. (2010). Heritable targeted mutagenesis in maize using a designed
endonuclease. Plant J.

[24] D'Halluin K., Vanderstraeten C., Van Hulle J., Rosolowska J., Van Den Brande I., Pennewaert A., D'Hont K., Bossut M., Jantz D., Ruiter R. (2013). Targeted molecular trait
stacking in cotton through targeted double-strand break induction. Plant Biotechnol J.

[25] Djukanovic V., Smith J., Lowe K., Yang M., Gao H., Jones S., Nicholson M. G., West A., Lape J., Bidney D. (2013). Male-sterile
maize plants produced by targeted mutagenesis
of the cytochrome P450-like gene (MS26) using a re-designed I-CreI
homing endonuclease. Plant J.

[26] Ainley W. M., Sastry-Dent L., Welter M. E., Murray M. G., Zeitler B., Amora R., Corbin D. R., Miles R. R., Arnold N. L., Strange T. L. (2013). Trait stacking via targeted genome editing. Plant Biotechnol J.

[27] Christian M., Cermak T., Doyle E. L., Schmidt C., Zhang F., Hummel A., Bogdanove A. J., Voytas D. F. (2010). Targeting DNA double-strand
breaks with TAL effector nucleases. Genetics.

[28] Cermak T., Doyle E. L., Christian M., Wang L., Zhang Y., Schmidt C., Baller J. A., Somia N. V., Bogdanove A. J., Voytas D. F. (2011). Efficient design and assembly of custom TALEN and other
TAL effector-based constructs for DNA targeting. Nucleic Acids Res.

[29] Li J. F., Norville J. E., Aach J., McCormack M., Zhang D., Bush J., Church G. M., Sheen J. (2013). Multiplex
and homologous recombination-mediated genome editing in Arabidopsis
and Nicotiana benthamiana using guide RNA and Cas9. Nat Biotechnol.

[30] Nekrasov V., Staskawicz B., Weigel D., Jones J. D., Kamoun S. (2013). Targeted mutagenesis
in the model plant Nicotiana benthamiana using Cas9 RNA-guided endonuclease. Nat Biotechnol.

[31] Menz J., Modrzejewski D., Hartung F., Wilhelm R., Sprink T. (2020). Genome Edited
Crops Touch the Market: A View on the Global Development and Regulatory
Environment. Front Plant Sci.

[32] Cong L., Ran F. A., Cox D., Lin S. L., Barretto R., Habib N., Hsu P. D., Wu X. B., Jiang W. Y., Marraffini L. A. (2013). Multiplex Genome Engineering
Using CRISPR/Cas
Systems. Science.

[33] Jinek M., Chylinski K., Fonfara I., Hauer M., Doudna J. A., Charpentier E. (2012). A programmable
dual-RNA-guided DNA endonuclease in
adaptive bacterial immunity. Science.

[34] Isaac, R. S. ; Jiang, F. ; Doudna, J. A. ; Lim, W. A. ; Narlikar, G. J. ; Almeida, R. Nucleosome breathing and remodeling constrain CRISPR-Cas9 function. Elife 2016, 5, 10.7554/eLife.13450. PMC4880442 27130520

[35] Champer J., Kim I. K., Champer S. E., Clark A. G., Messer P. W. (2020). Performance
analysis of novel toxin-antidote CRISPR gene drive systems. BMC Biol.

[36] Kim D., Kim J., Hur J. K., Been K. W., Yoon S. H., Kim J. S. (2016). Genome-wide
analysis reveals specificities of Cpf1 endonucleases in human cells. Nat Biotechnol.

[37] Kleinstiver B. P., Tsai S. Q., Prew M. S., Nguyen N. T., Welch M. M., Lopez J. M., McCaw Z. R., Aryee M. J., Joung J. K. (2016). Genome-wide
specificities of CRISPR-Cas Cpf1 nucleases in human cells. Nat Biotechnol.

[38] Jia H., Orbovic V., Wang N. (2019). CRISPR-LbCas12a-mediated
modification
of citrus. Plant Biotechnol J.

[39] Miller S.
M., Wang T., Randolph P. B., Arbab M., Shen M. W., Huang T. P., Matuszek Z., Newby G. A., Rees H. A., Liu D. R. (2020). Continuous
evolution of SpCas9 variants compatible
with non-G PAMs. Nat Biotechnol.

[40] Walton R. T., Christie K. A., Whittaker M. N., Kleinstiver B. P. (2020). Unconstrained
genome targeting with near-PAMless engineered CRISPR-Cas9 variants. Science.

[41] Kleinstiver B. P., Prew M. S., Tsai S. Q., Topkar V. V., Nguyen N. T., Zheng Z., Gonzales A. P., Li Z., Peterson R. T., Yeh J. R. (2015). Engineered CRISPR-Cas9
nucleases with altered PAM specificities. Nature.

[42] Nishimasu H., Shi X., Ishiguro S., Gao L., Hirano S., Okazaki S., Noda T., Abudayyeh O. O., Gootenberg J. S., Mori H. (2018). Engineered CRISPR-Cas9 nuclease with expanded targeting
space. Science.

[43] Niu Q., Wu S., Li Y., Yang X., Liu P., Xu Y., Lang Z. (2020). Expanding
the scope of CRISPR/Cas9-mediated genome editing in plants
using an xCas9 and Cas9-NG hybrid. J Integr
Plant Biol.

[44] Ren B., Liu L., Li S., Kuang Y., Wang J., Zhang D., Zhou X., Lin H., Zhou H. (2019). Cas9-NG Greatly Expands
the Targeting Scope of the Genome-Editing Toolkit by Recognizing NG
and Other Atypical PAMs in Rice. Mol Plant.

[45] Zhong Z., Sretenovic S., Ren Q., Yang L., Bao Y., Qi C., Yuan M., He Y., Liu S., Liu X. (2019). Improving Plant Genome Editing with High-Fidelity xCas9 and Non-canonical
PAM-Targeting Cas9-NG. Mol Plant.

[46] Li W., Li X., Wang C., Huo G., Zhang X., Yu J., Yu X., Li J., Zhang C., Zhao J. (2024). Expanding
the targeting scope of CRISPR/Cas9-mediated genome editing by Cas9
variants in Brassica. aBIOTECH.

[47] Ren Q., Sretenovic S., Liu S., Tang X., Huang L., He Y., Liu L., Guo Y., Zhong Z., Liu G. (2021). PAM-less plant genome
editing using a CRISPR-SpRY toolbox. Nat Plants.

[48] Wu Z. W., Zhang Y. F., Yu H. P., Pan D., Wang Y. J., Wang Y. N., Li F., Liu C., Nan H., Chen W. Z. (2021). Programmed
genome editing by a miniature CRISPR-Cas12f
nuclease. Nat Chem Biol.

[49] Xu X., Chemparathy A., Zeng L., Kempton H. R., Shang S., Nakamura M., Qi L. S. (2021). Engineered miniature CRISPR-Cas system
for mammalian genome regulation and editing. Mol Cell.

[50] Kim D. Y., Lee J. M., Moon S. B., Chin H. J., Park S., Lim Y., Kim D., Koo T., Ko J. H., Kim Y. S. (2022). Efficient
CRISPR editing with a hypercompact Cas12f1 and engineered guide RNAs
delivered by adeno-associated virus. Nat Biotechnol.

[51] Pausch P., Al-Shayeb B., Bisom-Rapp E., Tsuchida C. A., Li Z., Cress B. F., Knott G. J., Jacobsen S. E., Banfield J. F., Doudna J. A. (2020). CRISPR-Cas Phi from huge phages is a hypercompact genome
editor. Science.

[52] Li Z., Zhong Z., Wu Z., Pausch P., Al-Shayeb B., Amerasekera J., Doudna J. A., Jacobsen S. E. (2023). Genome editing in
plants using the compact editor CasPhi. Proc
Natl Acad Sci U S A.

[53] Haun W., Coffman A., Clasen B. M., Demorest Z. L., Lowy A., Ray E., Retterath A., Stoddard T., Juillerat A., Cedrone F. (2014). Improved
soybean oil quality by targeted mutagenesis
of the fatty acid desaturase 2 gene family. Plant Biotechnol J.

[54] Satterlee J. W., Alonso D., Gramazio P., Jenike K. M., He J., Arrones A., Villanueva G., Plazas M., Ramakrishnan S., Benoit M. (2024). Convergent evolution of plant prickles by repeated
gene co-option over deep time. Science.

[55] Li T., Yang X., Yu Y., Si X., Zhai X., Zhang H., Dong W., Gao C., Xu C. (2018). Domestication
of wild tomato is accelerated by genome editing. Nat Biotechnol.

[56] Shen L., Hua Y., Fu Y., Li J., Liu Q., Jiao X., Xin G., Wang J., Wang X., Yan C. (2017). Rapid
generation of genetic diversity by multiplex CRISPR/Cas9 genome editing
in rice. Sci China Life Sci.

[57] Qiao D., Wang J., Lu M. H., Xin C., Chai Y., Jiang Y., Sun W., Cao Z., Guo S., Wang X. C. (2023). Optimized prime editing efficiently generates
heritable
mutations in maize. J Integr Plant Biol.

[58] Shalev G., Sitrit Y., Avivi-Ragolski N., Lichtenstein C., Levy A. A. (1999). Stimulation of homologous recombination
in plants by
expression of the bacterial resolvase ruvC. Proc Natl Acad Sci U S A.

[59] Sheehy R. E., Kramer M., Hiatt W. R. (1988). Reduction
of polygalacturonase activity
in tomato fruit by antisense RNA. Proc Natl
Acad Sci U S A.

[60] Wang D., Samsulrizal N. H., Yan C., Allcock N. S., Craigon J., Blanco-Ulate B., Ortega-Salazar I., Marcus S. E., Bagheri H. M., Perez
Fons L. (2019). Characterization of CRISPR Mutants Targeting
Genes Modulating Pectin Degradation in Ripening Tomato. Plant Physiol.

[61] Kim J., Kim J. S. (2016). Bypassing GMO regulations
with CRISPR gene editing. Nat Biotechnol.

[62] Waltz E. (2018). With a free
pass, CRISPR-edited plants reach market in record time. Nat Biotechnol.

[63] Waltz E. (2016). Gene-edited
CRISPR mushroom escapes US regulation. Nature.

[64] Waltz E. (2022). GABA-enriched
tomato is first CRISPR-edited food to enter market. Nat. Biotechnol.

[65] Kannan B., Jung J. H., Moxley G. W., Lee S. M., Altpeter F. (2018). TALEN-mediated
targeted mutagenesis of more than 100 COMT copies/alleles in highly
polyploid sugarcane improves saccharification efficiency without compromising
biomass yield. Plant Biotechnol J.

[66] Ma X., Zhang Q., Zhu Q., Liu W., Chen Y., Qiu R., Wang B., Yang Z., Li H., Lin Y. (2015). A Robust CRISPR/Cas9 System for Convenient,
High-Efficiency Multiplex
Genome Editing in Monocot and Dicot Plants. Mol Plant.

[67] Xing H. L., Dong L., Wang Z. P., Zhang H. Y., Han C. Y., Liu B., Wang X. C., Chen Q. J. (2014). A CRISPR/Cas9
toolkit for multiplex
genome editing in plants. BMC Plant Biol.

[68] Wang M., Mao Y., Lu Y., Tao X., Zhu J. K. (2017). Multiplex Gene Editing
in Rice Using the CRISPR-Cpf1 System. Mol Plant.

[69] Cermak T., Curtin S. J., Gil-Humanes J., Cegan R., Kono T. J. Y., Konecna E., Belanto J. J., Starker C. G., Mathre J. W., Greenstein R. L. (2017). A Multipurpose Toolkit to Enable Advanced Genome
Engineering in Plants. Plant Cell.

[70] Gao Y., Zhao Y. (2014). Self-processing
of ribozyme-flanked RNAs into guide RNAs in vitro
and in vivo for CRISPR-mediated genome editing. Journal of integrative plant biology.

[71] Xie K., Minkenberg B., Yang Y. (2015). Boosting CRISPR/Cas9 multiplex editing
capability with the endogenous tRNA-processing system. Proc Natl Acad Sci U S A.

[72] Li, X. D. ; Wang, Y. N. ; Chen, S. ; Tian, H. Q. ; Fu, D. Q. ; Zhu, B. Z. ; Luo, Y. B. ; Zhu, H. L. Lycopene Is Enriched in Tomato Fruit by CRISPR/Cas9-Mediated Multiplex Genome Editing. Frontiers in Plant Science 2018, 9, 10.3389/fpls.2018.00559. PMC5935052 29755497

[73] Nakayasu M., Akiyama R., Lee H. J., Osakabe K., Osakabe Y., Watanabe B., Sugimoto Y., Umemoto N., Saito K., Muranaka T. (2018). Generation
of alpha-solanine-free hairy roots
of potato by CRISPR/Cas9 mediated genome editing of the St16DOX gene. Plant Physiol Biochem.

[74] Hanania U., Ariel T., Tekoah Y., Fux L., Sheva M., Gubbay Y., Weiss M., Oz D., Azulay Y., Turbovski A. (2017). Establishment of a tobacco
BY2 cell line devoid
of plant-specific xylose and fucose as a platform for the production
of biotherapeutic proteins. Plant Biotechnol
J.

[75] Rodriguez-Leal D., Lemmon Z. H., Man J., Bartlett M. E., Lippman Z. B. (2017). Engineering
Quantitative Trait Variation for Crop Improvement by Genome Editing. Cell.

[76] Sulis D. B., Jiang X., Yang C., Marques B. M., Matthews M. L., Miller Z., Lan K., Cofre-Vega C., Liu B., Sun R. (2023). Multiplex CRISPR editing of wood for sustainable
fiber production. Science.

[77] Liu H. J., Jian L., Xu J., Zhang Q., Zhang M., Jin M., Peng Y., Yan J., Han B., Liu J. (2020). High-Throughput CRISPR/Cas9
Mutagenesis Streamlines Trait Gene Identification
in Maize. Plant Cell.

[78] Hu Y., Patra P., Pisanty O., Shafir A., Belew Z. M., Binenbaum J., Ben Yaakov S., Shi B., Charrier L., Hyams G. (2023). Multi-Knock-a multi-targeted genome-scale CRISPR toolbox
to overcome functional redundancy in plants. Nat Plants.

[79] Sanchez-Leon S., Gil-Humanes J., Ozuna C. V., Gimenez M. J., Sousa C., Voytas D. F., Barro F. (2018). Low-gluten, nontransgenic
wheat engineered
with CRISPR/Cas9. Plant Biotechnol J.

[80] Sauer N. J., Mozoruk J., Miller R. B., Warburg Z. J., Walker K. A., Beetham P. R., Schopke C. R., Gocal G. F. (2016). Oligonucleotide-directed
mutagenesis for precision gene editing. Plant
Biotechnol J.

[81] Anzalone A. V., Koblan L. W., Liu D. R. (2020). Genome editing with CRISPR-Cas nucleases,
base editors, transposases and prime editors. Nat Biotechnol.

[82] Li C., Zhang R., Meng X., Chen S., Zong Y., Lu C., Qiu J. L., Chen Y. H., Li J., Gao C. (2020). Targeted,
random mutagenesis of plant genes with dual cytosine and adenine base
editors. Nat Biotechnol.

[83] Shimatani Z., Kashojiya S., Takayama M., Terada R., Arazoe T., Ishii H., Teramura H., Yamamoto T., Komatsu H., Miura K. (2017). Targeted base editing in rice and tomato using a CRISPR-Cas9
cytidine deaminase fusion. Nat Biotechnol.

[84] Zong Y., Song Q. N., Li C., Jin S., Zhang D. B., Wang Y. P., Qiu J. L., Gao C. X. (2018). Efficient
C-to-T
base editing in plants using a fusion of nCas9 and human APOBEC3A. Nature Biotechnology.

[85] Tian S., Jiang L., Cui X., Zhang J., Guo S., Li M., Zhang H., Ren Y., Gong G., Zong M. (2018). Engineering herbicide-resistant watermelon variety through CRISPR/Cas9-mediated
base-editing. Plant Cell Rep.

[86] Svitashev S., Schwartz C., Lenderts B., Young J. K., Mark
Cigan A. (2016). Genome editing in maize directed by CRISPR-Cas9 ribonucleoprotein
complexes. Nat Commun.

[87] Li J., Meng X., Zong Y., Chen K., Zhang H., Liu J., Li J., Gao C. (2016). Gene replacements and insertions
in rice by intron targeting using CRISPR-Cas9. Nat Plants.

[88] Hummel A. W., Chauhan R. D., Cermak T., Mutka A. M., Vijayaraghavan A., Boyher A., Starker C. G., Bart R., Voytas D. F., Taylor N. J. (2018). Allele exchange
at the EPSPS locus confers glyphosate
tolerance in cassava. Plant Biotechnol J.

[89] Li, C. ; Zong, Y. ; Wang, Y. P. ; Jin, S. ; Zhang, D. B. ; Song, Q. N. ; Zhang, R. ; Gao, C. X. Expanded base editing in rice and wheat using a Cas9-adenosine deaminase fusion. Genome Biology 2018, 19, 10.1186/s13059-018-1443-z. PMC5972399 29807545

[90] Xing S., Chen K., Zhu H., Zhang R., Zhang H., Li B., Gao C. (2020). Fine-tuning sugar content
in strawberry. Genome Biol.

[91] Yu Q. H., Wang B., Li N., Tang Y., Yang S., Yang T., Xu J., Guo C., Yan P., Wang Q. (2017). CRISPR/Cas9-induced
Targeted Mutagenesis and Gene Replacement
to Generate Long-shelf Life Tomato Lines. Sci
Rep.

[92] Anzalone A. V., Randolph P. B., Davis J. R., Sousa A. A., Koblan L. W., Levy J. M., Chen P. J., Wilson C., Newby G. A., Raguram A. (2019). Search-and-replace
genome editing without double-strand
breaks or donor DNA. Nature.

[93] Jiang Y. Y., Chai Y. P., Lu M. H., Han X. L., Lin Q., Zhang Y., Zhang Q., Zhou Y., Wang X. C., Gao C. (2020). Prime
editing efficiently generates W542L and S621I
double mutations in two ALS genes in maize. Genome Biol.

[94] Butt H., Rao G. S., Sedeek K., Aman R., Kamel R., Mahfouz M. (2020). Engineering herbicide resistance via prime editing
in rice. Plant Biotechnol J.

[95] Vu T. V., Nguyen N. T., Kim J., Song Y. J., Nguyen T. H., Kim J. Y. (2024). Optimized dicot
prime editing enables heritable desired
edits in tomato and Arabidopsis. Nat Plants.

[96] Ni P., Zhao Y., Zhou X., Liu Z., Huang Z., Ni Z., Sun Q., Zong Y. (2023). Efficient and versatile multiplex
prime editing in hexaploid wheat. Genome Biol.

[97] Vu T. V., Nguyen N. T., Kim J., Hong J. C., Kim J. Y. (2024). Prime editing:
Mechanism insight and recent applications in plants. Plant Biotechnol J.

[98] Packer M. S., Liu D. R. (2015). Methods for the
directed evolution of proteins. Nat Rev Genet.

[99] Yang K. K., Wu Z., Arnold F. H. (2019). Machine-learning-guided
directed evolution for protein
engineering. Nat Methods.

[100] Morrison M. S., Podracky C. J., Liu D. R. (2020). The developing
toolkit
of continuous directed evolution. Nat Chem Biol.

[101] Lin J. L., Wagner J. M., Alper H. S. (2017). Enabling tools for
high-throughput detection of metabolites: Metabolic engineering and
directed evolution applications. Biotechnol
Adv.

[102] Wang Y., Xue P., Cao M., Yu T., Lane S. T., Zhao H. (2021). Directed Evolution: Methodologies
and Applications. Chem Rev.

[103] Butt H., Eid A., Momin A. A., Bazin J., Crespi M., Arold S. T., Mahfouz M. M. (2019). CRISPR
directed
evolution of the spliceosome for resistance to splicing inhibitors. Genome Biol.

[104] Collonnier C., Guyon-Debast A., Maclot F., Mara K., Charlot F., Nogue F. (2017). Towards mastering CRISPR-induced
gene knock-in in plants: Survey of key features and focus on the model
Physcomitrella patens. Methods.

[105] Capdeville N., Schindele P., Puchta H. (2020). Application of CRISPR/Cas-mediated
base editing for directed protein evolution in plants. Sci China Life Sci.

[106] Doyle E. L., Booher N. J., Standage D. S., Voytas D. F., Brendel V. P., Vandyk J. K., Bogdanove A. J. (2012). TAL Effector-Nucleotide
Targeter (TALE-NT) 2.0: tools for TAL effector design and target prediction. Nucleic Acids Res.

[107] Zhang C., Hua Q. (2016). Applications of Genome-Scale
Metabolic
Models in Biotechnology and Systems Medicine. Front Physiol.

[108] Liang Z., Zhang K., Chen K., Gao C. (2014). Targeted mutagenesis
in Zea mays using TALENs and the CRISPR/Cas system. J Genet Genomics.

[109] Wendt T., Holm P. B., Starker C. G., Christian M., Voytas D. F., Brinch-Pedersen H., Holme I. B. (2013). TAL effector nucleases
induce mutations at a pre-selected location in the genome of primary
barley transformants. Plant Mol Biol.

[110] Wen S., Liu H., Li X., Chen X., Hong Y., Li H., Lu Q., Liang X. (2018). TALEN-mediated targeted mutagenesis
of fatty acid desaturase 2 (FAD2) in peanut (Arachis hypogaea L.)
promotes the accumulation of oleic acid. Plant
Mol Biol.

[111] Wang C., Zhang X., Fan Y., Gao Y., Zhu Q., Zheng C., Qin T., Li Y., Che J., Zhang M. (2015). XA23 Is an Executor R Protein and Confers Broad-Spectrum
Disease Resistance in Rice. Molecular Plant.

[112] Concordet J. P., Haeussler M. (2018). CRISPOR: intuitive guide selection
for CRISPR/Cas9 genome editing experiments and screens. Nucleic Acids Res.

[113] Park J., Bae S., Kim J. S. (2015). Cas-Designer:
a
web-based tool for choice of CRISPR-Cas9 target sites. Bioinformatics.

[114] Liu H., Ding Y., Zhou Y., Jin W., Xie K., Chen L. L. (2017). CRISPR-P 2.0: An Improved CRISPR-Cas9
Tool for Genome
Editing in Plants. Mol Plant.

[115] Lei Y., Lu L., Liu H. Y., Li S., Xing F., Chen L. L. (2014). CRISPR-P: a web tool for synthetic
single-guide RNA
design of CRISPR-system in plants. Mol Plant.

[116] Hwang G.
H., Jeong Y. K., Habib O., Hong S. A., Lim K., Kim J. S., Bae S. (2021). PE-Designer and PE-Analyzer: web-based
design and analysis tools for CRISPR prime editing. Nucleic Acids Res.

[117] Kim Y. A., Moon H., Park C. J. (2019). CRISPR/Cas9-targeted
mutagenesis of Os8N3 in rice to confer resistance to Xanthomonas oryzae
pv. oryzae. Rice (N Y).

[118] Liu L., Gallagher J., Arevalo E. D., Chen R., Skopelitis T., Wu Q., Bartlett M., Jackson D. (2021). Enhancing grain-yield-related traits
by CRISPR-Cas9 promoter editing of maize CLE genes. Nat Plants.

[119] Naim F., Shand K., Hayashi S., O'Brien M., McGree J., Johnson A. A. T., Dugdale B., Waterhouse P. M. (2020). Are the
current gRNA ranking prediction algorithms useful for genome editing
in plants?. PLoS One.

[120] D’Orso, F. ; Forte, V. ; Baima, S. ; Possenti, M. ; Palma, D. ; Morelli, G. Methods and Techniques to Select Efficient Guides for CRISPR-Mediated Genome Editing in Plants. In A Roadmap for Plant Genome Editing; Springer Nature Switzerland Cham, 2023; pp 89-117.

[121] Zambryski P., Joos H., Genetello C., Leemans J., Vanmontagu M., Schell J. (1983). Ti-Plasmid Vector for
the Introduction of DNA into Plant-Cells without Alteration of Their
Normal Regeneration Capacity. Embo J.

[122] Vaeck M., Reynaerts A., Hofte H., Jansens S., Debeuckeleer M., Dean C., Zabeau M., Vanmontagu M., Leemans J. (1987). Transgenic Plants Protected from Insect Attack. Nature.

[123] Lemaux P. G. (2008). Genetically
Engineered Plants and Foods: A Scientist's
Analysis of the Issues (Part I). Annu Rev Plant
Biol.

[124] Christou P. (2013). Plant genetic
engineering and agricultural biotechnology
1983-2013. Trends Biotechnol.

[125] Darlington, M. ; Reinders, J. D. ; Sethi, A. ; Lu, A. L. ; Ramaseshadri, P. ; Fischer, J. R. ; Boeckman, C. J. ; Petrick, J. S. ; Roper, J. M. ; Narva, K. E. , RNAi for Western Corn Rootworm Management: Lessons Learned, Challenges, and Future Directions. Insects 2022, 13 (1), 57 10.3390/insects13010057. 35055900 PMC8779393

[126] Asojo O. A., Damania A., Turner T. L., Slaughter G., Hirschi K. D. (2017). RETRACTED ARTICLE: Summer Research
Training Provides
Effective Tools for Underrepresented Minorities to Obtain Doctoral
Level Degrees. J Racial Ethn Health Disparities.

[127] Zhu Q., Yu S., Zeng D., Liu H., Wang H., Yang Z., Xie X., Shen R., Tan J., Li H. (2017). Development of “Purple Endosperm Rice”
by Engineering Anthocyanin Biosynthesis in the Endosperm with a High-Efficiency
Transgene Stacking System. Mol Plant.

[128] Zhu Q., Zeng D., Yu S., Cui C., Li J., Li H., Chen J., Zhang R., Zhao X., Chen L. (2018). From Golden Rice to aSTARice: Bioengineering Astaxanthin Biosynthesis
in Rice Endosperm. Mol Plant.

[129] Luo M., Xie L., Chakraborty S., Wang A., Matny O., Jugovich M., Kolmer J. A., Richardson T., Bhatt D., Hoque M. (2021). A five-transgene
cassette
confers broad-spectrum resistance to a fungal rust pathogen in wheat. Nat Biotechnol.

[130] Shih P. M., Vuu K., Mansoori N., Ayad L., Louie K. B., Bowen B. P., Northen T. R., Loque D. (2016). A robust gene-stacking
method utilizing yeast assembly for plant synthetic biology. Nat Commun.

[131] Hamilton C. M., Frary A., Lewis C., Tanksley S. D. (1996). Stable
transfer of intact high molecular weight DNA into plant chromosomes. Proc Natl Acad Sci U S A.

[132] Butler N. M., Baltes N. J., Voytas D. F., Douches D. S. (2016). Geminivirus-Mediated
Genome Editing in Potato (Solanum tuberosum L.) Using Sequence-Specific
Nucleases. Front Plant Sci.

[133] Cermak T., Baltes N. J., Cegan R., Zhang Y., Voytas D. F. (2015). High-frequency, precise modification of the tomato
genome. Genome Biol.

[134] Gil-Humanes J., Wang Y., Liang Z., Shan Q., Ozuna C. V., Sanchez-Leon S., Baltes N. J., Starker C., Barro F., Gao C. (2017). High-efficiency gene
targeting in hexaploid wheat using DNA replicons and CRISPR/Cas9. Plant J.

[135] Lu Y., Tian Y., Shen R., Yao Q., Wang M., Chen M., Dong J., Zhang T., Li F., Lei M. (2020). Targeted, efficient sequence insertion and
replacement
in rice. Nat Biotechnol.

[136] Wang M., Lu Y., Botella J. R., Mao Y., Hua K., Zhu J. K. (2017). Gene Targeting by Homology-Directed
Repair in Rice
Using a Geminivirus-Based CRISPR/Cas9 System. Mol Plant.

[137] Miki D., Zhang W., Zeng W., Feng Z., Zhu J. K. (2018). CRISPR/Cas9-mediated gene targeting in Arabidopsis
using sequential transformation. Nat Commun.

[138] Hanin M., Paszkowski J. (2003). Plant genome
modification by homologous
recombination. Curr Opin Plant Biol.

[139] Dong, O. X. ; Ronald, P. C. Targeted DNA insertion in plants. Proc Natl Acad Sci U S A 2021, 118 (22), 10.1073/pnas.2004834117. PMC8179203 34050013

[140] Strecker J., Ladha A., Gardner Z., Schmid-Burgk J. L., Makarova K. S., Koonin E. V., Zhang F. (2019). RNA-guided DNA insertion
with CRISPR-associated transposases. Science.

[141] Klompe S. E., Vo P. L. H., Halpin-Healy T. S., Sternberg S. H. (2019). Transposon-encoded
CRISPR-Cas systems direct RNA-guided
DNA integration. Nature.

[142] Vo P. L. H., Ronda C., Klompe S. E., Chen E. E., Acree C., Wang H. H., Sternberg S. H. (2021). CRISPR
RNA-guided integrases for high-efficiency, multiplexed bacterial genome
engineering. Nat Biotechnol.

[143] Lampe G. D., King R. T., Halpin-Healy T. S., Klompe S. E., Hogan M. I., Vo P. L. H., Tang S., Chavez A., Sternberg S. H. (2024). Targeted DNA integration in human
cells without double-strand breaks using CRISPR-associated transposases. Nat Biotechnol.

[144] Liu P., Panda K., Edwards S. A., Swanson R., Yi H., Pandesha P., Hung Y. H., Klaas G., Ye X., Collins M. V. (2024). Transposase-assisted target-site integration
for efficient plant genome engineering. Nature.

[145] Park Y., Espah Borujeni A., Gorochowski T. E., Shin J., Voigt C. A. (2020). Precision
design of stable genetic
circuits carried in highly-insulated E. coli genomic landing pads. Mol Syst Biol.

[146] Gaidukov L., Wroblewska L., Teague B., Nelson T., Zhang X., Liu Y., Jagtap K., Mamo S., Tseng W. A., Lowe A. (2018). A multi-landing pad
DNA integration platform for mammalian cell engineering. Nucleic Acids Res.

[147] Chen Y., Zhang S., Young E. M., Jones T. S., Densmore D., Voigt C. A. (2020). Genetic circuit
design automation
for yeast. Nat Microbiol.

[148] Lee M. E., DeLoache W. C., Cervantes B., Dueber J. E. (2015). A Highly Characterized Yeast Toolkit for Modular, Multipart
Assembly. ACS Synth Biol.

[149] Gao H., Mutti J., Young J. K., Yang M., Schroder M., Lenderts B., Wang L., Peterson D., St Clair G., Jones S. (2020). Complex
Trait Loci in Maize Enabled by CRISPR-Cas9
Mediated Gene Insertion. Front Plant Sci.

[150] Scholz S. A., Diao R., Wolfe M. B., Fivenson E. M., Lin X. N., Freddolino P. L. (2019). High-Resolution
Mapping of the Escherichia
coli Chromosome Reveals Positions of High and Low Transcription. Cell Syst.

[151] Brady J. R., Tan M. C., Whittaker C. A., Colant N. A., Dalvie N. C., Love K. R., Love J. C. (2020). Identifying
Improved Sites for Heterologous Gene Integration Using ATAC-seq. ACS Synth Biol.

[152] Dhiman H., Campbell M., Melcher M., Smith K. D., Borth N. (2020). Predicting favorable landing pads
for targeted integrations in Chinese
hamster ovary cell lines by learning stability characteristics from
random transgene integrations. Comput Struct
Biotechnol J.

[153] Buenrostro J. D., Wu B., Chang H. Y., Greenleaf W. J. (2015). ATAC-seq:
A Method for Assaying Chromatin Accessibility Genome-Wide. Curr Protoc Mol Biol.

[154] Maher K. A., Bajic M., Kajala K., Reynoso M., Pauluzzi G., West D. A., Zumstein K., Woodhouse M., Bubb K., Dorrity M. W. (2018). Profiling of Accessible
Chromatin Regions across Multiple Plant Species and Cell Types Reveals
Common Gene Regulatory Principles and New Control Modules. Plant Cell.

[155] Bajic M., Maher K. A., Deal R. B. (2018). Identification of
Open Chromatin Regions in Plant Genomes Using ATAC-Seq. Methods Mol Biol.

[156] Lu Z., Hofmeister B. T., Vollmers C., DuBois R. M., Schmitz R. J. (2017). Combining ATAC-seq with nuclei sorting for discovery
of cis-regulatory regions in plant genomes. Nucleic Acids Res.

[157] Aznauryan E., Yermanos A., Kinzina E., Devaux A., Kapetanovic E., Milanova D., Church G. M., Reddy S. T. (2022). Discovery
and validation of human genomic safe harbor sites for gene and cell
therapies. Cell Rep Methods.

[158] Chen Y., Zhang S., Young E. M., Jones T. S., Densmore D., Voigt C. A. (2020). Genetic circuit
design automation
for yeast. Nat Microbiol.

[159] Matzat L. H., Lei E. P. (2014). Surviving an identity
crisis: a revised
view of chromatin insulators in the genomics era. Biochim Biophys Acta.

[160] Murray A. W., Szostak J. W. (1983). Construction of
artificial chromosomes
in yeast. Nature.

[161] Burke D. T., Carle G. F., Olson M. V. (1987). Cloning
of large
segments of exogenous DNA into yeast by means of artificial chromosome
vectors. Science.

[162] Harrington J. J., Van Bokkelen G., Mays R. W., Gustashaw K., Willard H. F. (1997). Formation of de
novo centromeres and construction of
first-generation human artificial microchromosomes. Nat Genet.

[163] Ikeno M., Grimes B., Okazaki T., Nakano M., Saitoh K., Hoshino H., McGill N. I., Cooke H., Masumoto H. (1998). Construction
of YAC-based mammalian artificial chromosomes. Nat Biotechnol.

[164] Birchler J. A., Han F. (2009). Maize centromeres: structure, function,
epigenetics. Annu Rev Genet.

[165] Carlson S. R., Rudgers G. W., Zieler H., Mach J. M., Luo S., Grunden E., Krol C., Copenhaver G. P., Preuss D. (2007). Meiotic transmission of an in vitro-assembled
autonomous
maize minichromosome. PLoS Genet.

[166] Gaeta R. T., Masonbrink R. E., Krishnaswamy L., Zhao C., Birchler J. A. (2012). Synthetic chromosome
platforms in
plants. Annu Rev Plant Biol.

[167] Xu C., Cheng Z., Yu W. (2012). Construction
of rice mini-chromosomes
by telomere-mediated chromosomal truncation. Plant J.

[168] Liang Y., Richardson S., Yan J., Benites V. T., Cheng-Yue C., Tran T., Mortimer J., Mukhopadhyay A., Keasling J. D., Scheller H. V. (2017). Endoribonuclease-Based
Two-Component Repressor Systems for Tight Gene Expression Control
in Plants. ACS Synth Biol.

[169] Kapusi E., Ma L., Teo C. H., Hensel G., Himmelbach A., Schubert I., Mette M. F., Kumlehn J., Houben A. (2012). Telomere-mediated truncation of barley
chromosomes. Chromosoma.

[170] Yuan J., Shi Q., Guo X., Liu Y., Su H., Guo X., Lv Z., Han F. (2017). Site-specific
transfer
of chromosomal segments and genes in wheat engineered chromosomes. J Genet Genomics.

[171] Nelson A. D., Lamb J. C., Kobrossly P. S., Shippen D. E. (2011). Parameters affecting telomere-mediated chromosomal
truncation in Arabidopsis. Plant Cell.

[172] Teo C. H., Ma L., Kapusi E., Hensel G., Kumlehn J., Schubert I., Houben A., Mette M. F. (2011). Induction
of telomere-mediated chromosomal truncation and stability of truncated
chromosomes in Arabidopsis thaliana. Plant J.

[173] Swyers N. C., Cody J. P., McCaw M. E., Graham N. D., Zhao C., Gaeta R. T., Birchler J. A. (2016). Telomere-Mediated
Chromosomal Truncation for Generating Engineered Minichromosomes in
Maize. Curr Protoc Plant Biol.

[174] Gaeta R. T., Danilova T. V., Zhao C., Masonbrink R. E., McCaw M. E., Birchler J. A. (2011). Recovery of a telomere-truncated
chromosome via a compensating translocation in maize. Genome.

[175] Yu W., Han F., Gao Z., Vega J. M., Birchler J. A. (2007). Construction
and behavior of engineered minichromosomes in maize. Proc Natl Acad Sci U S A.

[176] Yu W., Lamb J. C., Han F., Birchler J. A. (2006). Telomere-mediated
chromosomal truncation in maize. Proc Natl Acad
Sci U S A.

[177] Yin X., Zhang Y., Chen Y., Wang J., Wang R. R., Fan C., Hu Z. (2021). Precise Characterization and Tracking of Stably Inherited
Artificial Minichromosomes Made by Telomere-Mediated Chromosome Truncation
in Brassica napus. Front Plant Sci.

[178] Han F., Gao Z., Yu W., Birchler J. A. (2008). Minichromosome analysis
of chromosome pairing, disjunction, and sister chromatid cohesion
in maize. Plant Cell.

[179] Masonbrink R. E., Gaeta R. T., Birchler J. A. (2012). Multiple
maize minichromosomes
in meiosis. Chromosome Res.

[180] Birchler J. A., Yu W., Han F. (2008). Plant engineered
minichromosomes
and artificial chromosome platforms. Cytogenet
Genome Res.

[181] Birchler J. A., Kelly J., Singh J., Liu H., Zhang Z., Char S. N., Sharma M., Yang H., Albert P. S., Yang B. (2024). Synthetic minichromosomes in plants:
past, present, and promise. Plant J.

[182] Kan M., Huang T., Zhao P. (2022). Artificial
chromosome technology
and its potential application in plants. Front.
Plant Sci..

[183] Jakubiec A., Sarokina A., Choinard S., Vlad F., Malcuit I., Sorokin A. P. (2021). Replicating minichromosomes
as a
new tool for plastid genome engineering. Nature
Plants.

[184] Yan X., Li C., Yang J., Wang L., Jiang C., Wei W. (2017). Induction
of telomere-mediated chromosomal truncation and behavior
of truncated chromosomes in Brassica napus. Plant J.

[185] Gaeta R. T., Masonbrink R. E., Zhao C., Sanyal A., Krishnaswamy L., Birchler J. A. (2013). In vivo modification of a maize engineered
minichromosome. Chromosoma.

[186] Mahmood, M. A. ; Naqvi, R. Z. ; Rahman, S. U. ; Amin, I. ; Mansoor, S. Plant Virus-Derived Vectors for Plant Genome Engineering. Viruses 2023, 15 (2), 531 10.3390/v15020531. 36851743 PMC9958682

[187] Zhang, C. ; Liu, S. ; Li, X. ; Zhang, R. ; Li, J. Virus-Induced Gene Editing and Its Applications in Plants. Int J Mol Sci 2022, 23 (18), 10202 10.3390/ijms231810202. 36142116 PMC9499690

[188] Zerbini F. M., Briddon R. W., Idris A., Martin D. P., Moriones E., Navas-Castillo J., Rivera-Bustamante R., Roumagnac P., Varsani A., Ictv Report C. (2017). ICTV Virus
Taxonomy Profile: Geminiviridae. J Gen Virol.

[189] Ellison E. E., Chamness J. C., Voytas D. F. (2021). Viruses as vectors
for the delivery of gene-editing reagents. Genome
editing for precision crop breeding.

[190] Zhao X. X., Coats I., Fu P., Gordon-Kamm B., Lyznik L. A. (2003). T-DNA recombination and replication in maize cells. Plant Journal.

[191] Richter K. S., Kleinow T., Jeske H. (2014). Somatic homologous
recombination in plants is promoted by a geminivirus in a tissue-selective
manner. Virology.

[192] Dahan-Meir T., Filler-Hayut S., Melamed-Bessudo C., Bocobza S., Czosnek H., Aharoni A., Levy A. A. (2018). Efficient
in planta gene targeting in tomato using geminiviral replicons and
the CRISPR/Cas9 system. Plant Journal.

[193] Ruiz M. T., Voinnet O., Baulcombe D. C. (1998). Initiation
and maintenance of virus-induced gene silencing. Plant Cell.

[194] Singh U., Wilkens P. W., Henao J., Chien S. H., Hellums D. T., Hammond L. L. (2003). An expert system for estimating agronomic
effectiveness of freshly applied phosphate rock. Direct Application of Phosphate Rock and Related Appropriate Technology-Latest
Developments and Practical Experiences, Proceedings.

[195] Scholthof H. B., Scholthof K. B., Jackson A. O. (1996). Plant virus gene
vectors for transient expression of foreign proteins in plants. Annu Rev Phytopathol.

[196] Kolliopoulou A., Taning C. N. T., Smagghe G., Swevers L. (2017). Viral Delivery
of dsRNA for Control of Insect Agricultural Pests and Vectors of Human
Disease: Prospects and Challenges. Front Physiol.

[197] Beernink B. M., Lappe R. R., Bredow M., Whitham S. A. (2022). Impacts
of RNA Mobility Signals on Virus Induced Somatic and Germline Gene
Editing. Front Genome Ed.

[198] Ellison E. E., Nagalakshmi U., Gamo M. E., Huang P. J., Dinesh-Kumar S., Voytas D. F. (2021). Multiplexed heritable gene editing
using RNA viruses and mobile single guide RNAs (vol 6, pg 620, 2020). Nature Plants.

[199] Ellison, E. E. ; Chamness, J. C. ; Voytas, D. F. Viruses as vectors for the delivery of gene editing reagents. Genome editing for precision crop breeding 2021, 97 10.19103/AS.2020.0082.28.

[200] Ali Z., Abul-faraj A., Li L., Ghosh N., Piatek M., Mahjoub A., Aouida M., Piatek A., Baltes N. J., Voytas D. F. (2015). Efficient Virus-Mediated Genome Editing in
Plants Using the CRISPR/Cas9 System. Mol Plant.

[201] Mei Y., Beernink B. M., Ellison E. E., Konecna E., Neelakandan A. K., Voytas D. F., Whitham S. A. (2019). Protein expression and gene editing
in monocots using foxtail mosaic virus vectors. Plant Direct.

[202] Weiss T., Kamalu M., Shi H., Li Z., Amerasekera J., Zhong Z., Adler B. A., Song M. M., Vohra K., Wirnowski G. (2025). Viral delivery of an
RNA-guided genome editor for transgene-free germline editing in Arabidopsis. Nat Plants.

[203] Gao Q., Xu W. Y., Yan T., Fang X. D., Cao Q., Zhang Z. J., Ding Z. H., Wang Y., Wang X. B. (2019). Rescue
of a plant cytorhabdovirus as versatile expression platforms for planthopper
and cereal genomic studies. New Phytol.

[204] Li C., Zhang K., Zeng X., Jackson S., Zhou Y., Hong Y. (2009). A cis element within
flowering locus T mRNA determines its mobility
and facilitates trafficking of heterologous viral RNA. J Virol.

[205] Nagalakshmi U., Meier N., Liu J. Y., Voytas D. F., Dinesh-Kumar S. P. (2022). High-efficiency multiplex biallelic
heritable editing
in Arabidopsis using an RNA virus. Plant Physiol.

[206] Liu D. G., Xuan S. Y., Prichard L. E., Donahue L. I., Pan C. T., Nagalakshmi U., Ellison E. E., Starker C. G., Dinesh-Kumar S. P., Qi Y. P. (2022). Heritable base-editing
in Arabidopsis using RNA viral vectors. Plant
Physiology.

[207] Weiss T., Kamalu M., Shi H., Li Z., Amerasekera J., Zhong Z., Adler B. A., Song M., Vohra K., Wirnowski G. (2024). Viral delivery of an RNA-guided genome
editor for transgene-free germline editing in Arabidopsis. bioRxiv.

[208] Frary A., Hamilton C. M. (2001). Efficiency and stability
of high
molecular weight DNA transformation: an analysis in tomato. Transgenic Res.

[209] Hansen G., Das A., Chilton M. D. (1994). Constitutive
expression
of the virulence genes improves the efficiency of plant transformation
by Agrobacterium. Proc Natl Acad Sci U S A.

[210] Liu C. N., Li X. Q., Gelvin S. B. (1992). Multiple copies
of virG enhance the transient transformation of celery, carrot and
rice tissues by Agrobacterium tumefaciens. Plant
Mol Biol.

[211] Yadava P., Abhishek A., Singh R., Singh I., Kaul T., Pattanayak A., Agrawal P. K. (2017). Advances in Maize
Transformation Technologies and Development of Transgenic Maize. Front Plant Sci.

[212] van der Fits L., Deakin E. A., Hoge J. H., Memelink J. (2000). The ternary
transformation system: constitutive virG on a compatible plasmid dramatically
increases Agrobacterium-mediated plant transformation. Plant Mol Biol.

[213] Nonaka S., Someya T., Zhou S., Takayama M., Nakamura K., Ezura H. (2017). An Agrobacterium tumefaciens
Strain
with Gamma-Aminobutyric Acid Transaminase Activity Shows an Enhanced
Genetic Transformation Ability in Plants. Sci
Rep.

[214] Nonaka S., Sugawara M., Minamisawa K., Yuhashi K., Ezura H. (2008). 1-Aminocyclopropane-1-carboxylate
deaminase enhances Agrobacterium tumefaciens-mediated gene transfer
into plant cells. Appl Environ Microbiol.

[215] Raman V., Rojas C. M., Vasudevan B., Dunning K., Kolape J., Oh S., Yun J., Yang L., Li G., Pant B. D. (2022). Agrobacterium
expressing a type III secretion system delivers Pseudomonas effectors
into plant cells to enhance transformation. Nat Commun.

[216] Szarzanowicz M. J., Waldburger L. M., Busche M., Geiselman G. M., Kirkpatrick L. D., Kehl A. J., Tahmin C., Kuo R. C., McCauley J., Pannu H. (2024). Binary vector copy number
engineering improves Agrobacterium-mediated transformation. Nat Biotechnol.

[217] Oltmanns H., Frame B., Lee L. Y., Johnson S., Li B., Wang K., Gelvin S. B. (2010). Generation of backbone-free, low
transgene copy plants by launching T-DNA from the Agrobacterium chromosome. Plant Physiol.

[218] Sastry G. R. K., Miles C. A., Miller I. S., Borland P. A., Saeed N., May C. A. (1986). Tryptophan Auxotrophs
for Increasing
Safety of Agrobacterium Ti-based Recombinant Plasmid Work. Plant Molecular Biology Reporter.

[219] Kuraya Y., Ohta S., Fukuda M., Hiei Y., Murai N., Hamada K., Ueki J., Imaseki H., Komari T. (2004). Suppression of transfer of non-T-DNA
‘vector
backbone’sequences by multiple left border repeats in vectors
for transformation of higher plants mediated by Agrobacterium tumefaciens. Molecular Breeding.

[220] Denkovskiene E., Paskevicius S., Werner S., Gleba Y., Razanskiene A. (2015). Inducible
Expression of Agrobacterium Virulence Gene
VirE2 for Stringent Regulation of T-DNA Transfer in Plant Transient
Expression Systems. Mol Plant Microbe Interact.

[221] Broothaerts W., Mitchell H. J., Weir B., Kaines S., Smith L. M., Yang W., Mayer J. E., Roa-Rodriguez C., Jefferson R. A. (2005). Gene transfer to plants by diverse species of bacteria. Nature.

[222] Lacroix B., Citovsky V. (2016). A Functional Bacterium-to-Plant
DNA
Transfer Machinery of Rhizobium etli. PLoS Pathog.

[223] Wendt T., Doohan F., Mullins E. (2012). Production of Phytophthora
infestans-resistant potato (Solanum tuberosum) utilising Ensifer adhaerens
OV14. Transgenic Res.

[224] Cho H. J., Moy Y., Rudnick N. A., Klein T. M., Yin J., Bolar J., Hendrick C., Beatty M., Castaneda L., Kinney A. J. (2022). Development
of an efficient marker-free soybean
transformation method using the novel bacterium Ochrobactrum haywardense
H1. Plant Biotechnol J.

[225] Marillonnet S., Thoeringer C., Kandzia R., Klimyuk V., Gleba Y. (2005). Systemic Agrobacterium
tumefaciens-mediated transfection of viral
replicons for efficient transient expression in plants. Nat Biotechnol.

[226] Hahn S., Giritch A., Bartels D., Bortesi L., Gleba Y. (2015). A novel and fully scalable Agrobacterium
spray-based process for
manufacturing cellulases and other cost-sensitive proteins in plants. Plant Biotechnol J.

[227] Gleba Y. Y., Tuse D., Giritch A. (2013). Plant viral
vectors
for delivery by Agrobacterium. Curr Top Microbiol
Immunol.

[228] Torti S., Schlesier R., Thummler A., Bartels D., Romer P., Koch B., Werner S., Panwar V., Kanyuka K., Wiren N. V. (2021). Transient reprogramming
of crop plants for agronomic performance. Nat
Plants.

[229] Fuentes P., Armarego-Marriott T., Bock R. (2018). Plastid transformation
and its application in metabolic engineering. Curr Opin Biotechnol.

[230] Buren, S. ; Rubio, L. M. State of the art in eukaryotic nitrogenase engineering. FEMS Microbiol Lett 2018, 365 (2), 10.1093/femsle/fnx274. PMC5812491 29240940

[231] Jarvis P., Lopez-Juez E. (2013). Biogenesis
and homeostasis of chloroplasts
and other plastids. Nat Rev Mol Cell Biol.

[232] Gibson D.
G., Glass J. I., Lartigue C., Noskov V. N., Chuang R. Y., Algire M. A., Benders G. A., Montague M. G., Ma L., Moodie M. M. (2010). Creation of a bacterial cell controlled
by a chemically synthesized genome. Science.

[233] Oey M., Lohse M., Kreikemeyer B., Bock R. (2009). Exhaustion of the chloroplast
protein synthesis capacity by massive expression of a highly stable
protein antibiotic. Plant J.

[234] Boeke J. D., Church G., Hessel A., Kelley N. J., Arkin A., Cai Y., Carlson R., Chakravarti A., Cornish V. W., Holt L. (2016). GENOME
ENGINEERING.
The Genome Project-Write. Science.

[235] Mellor S. B., Behrendorff J. B. Y.
H., Nielsen A. Z., Jensen P. E., Pribil M. (2018). Non-photosynthetic plastids as hosts
for metabolic engineering. Essays Biochem.

[236] Maliga, P. Chloroplast biotechnology: methods and protocols; Springer, 2014.

[237] Lu Y., Rijzaani H., Karcher D., Ruf S., Bock R. (2013). Efficient
metabolic pathway engineering in transgenic tobacco and tomato plastids
with synthetic multigene operons. Proc Natl
Acad Sci U S A.

[238] Yabuta Y., Tanaka H., Yoshimura S., Suzuki A., Tamoi M., Maruta T., Shigeoka S. (2013). Improvement
of vitamin E quality and quantity in tobacco and lettuce by chloroplast
genetic engineering. Transgenic Res.

[239] Zhang J., Khan S. A., Hasse C., Ruf S., Heckel D. G., Bock R. (2015). Pest control. Full crop protection
from an insect pest by expression of long double-stranded RNAs in
plastids. Science.

[240] De Marchis F., Wang Y., Stevanato P., Arcioni S., Bellucci M. (2009). Genetic transformation of the sugar
beet plastome. Transgenic Res.

[241] Yu Q., Lutz K. A., Maliga P. (2017). Efficient
Plastid Transformation
in Arabidopsis. Plant Physiol.

[242] Ruf S., Forner J., Hasse C., Kroop X., Seeger S., Schollbach L., Schadach A., Bock R. (2019). High-efficiency generation
of fertile transplastomic Arabidopsis plants. Nat Plants.

[243] Dufourmantel N., Pelissier B., Garcon F., Peltier G., Ferullo J. M., Tissot G. (2004). Generation of fertile transplastomic
soybean. Plant Mol Biol.

[244] Jin S., Daniell H. (2015). The Engineered Chloroplast
Genome Just Got Smarter. Trends Plant Sci.

[245] Okumura S., Sawada M., Park Y. W., Hayashi T., Shimamura M., Takase H., Tomizawa K. (2006). Transformation of poplar
(Populus alba) plastids and expression of foreign proteins in tree
chloroplasts. Transgenic Res.

[246] Maliga P. (2004). Plastid transformation in higher
plants. Annu Rev Plant Biol.

[247] Yu Q., LaManna L. M., Kelly M. E., Lutz K. A., Maliga P. (2019). New Tools
for Engineering the Arabidopsis Plastid Genome. Plant Physiol.

[248] Khan M. S., Maliga P. (1999). Fluorescent antibiotic resistance
marker for tracking plastid transformation in higher plants. Nat Biotechnol.

[249] Lee S. M., Kang K., Chung H., Yoo S. H., Xu X. M., Lee S. B., Cheong J. J., Daniell H., Kim M. (2006). Plastid transformation in the monocotyledonous
cereal crop, rice
(Oryza sativa) and transmission of transgenes to their progeny. Mol Cells.

[250] Day A., Goldschmidt-Clermont M. (2011). The chloroplast transformation
toolbox:
selectable markers and marker removal. Plant
Biotechnol J.

[251] Scotti N., Cardi T. (2014). Transgene-induced pleiotropic
effects
in transplastomic plants. Biotechnol Lett.

[252] Liu Y.-x., Li F., Gao L., Tu Z.-l., Zhou F., Lin Y.-j. (2023). Advancing
approach and toolbox in
optimization of chloroplast genetic transformation technology. Journal of Integrative Agriculture.

[253] Yoo B. C., Yadav N. S., Orozco E. M., Sakai H. (2020). Cas9/gRNA-mediated genome editing
of yeast mitochondria
and Chlamydomonas chloroplasts. PeerJ.

[254] Li R., Char S. N., Liu B., Liu H., Li X., Yang B. (2021). High-efficiency plastome base editing
in rice with TAL cytosine deaminase. Mol Plant.

[255] Nakazato I., Okuno M., Yamamoto H., Tamura Y., Itoh T., Shikanai T., Takanashi H., Tsutsumi N., Arimura S. I. (2021). Targeted
base editing in the plastid
genome of Arabidopsis thaliana. Nat Plants.

[256] Kang B. C., Bae S. J., Lee S., Lee J. S., Kim A., Lee H., Baek G., Seo H., Kim J., Kim J. S. (2021). Chloroplast
and mitochondrial DNA editing in plants. Nat
Plants.

[257] Cho S. I., Lee S., Mok Y. G., Lim K., Lee J., Lee J. M., Chung E., Kim J. S. (2022). Targeted A-to-G
base editing in human mitochondrial DNA with programmable deaminases. Cell.

[258] Mok Y. G., Hong S., Bae S. J., Cho S. I., Kim J. S. (2022). Targeted A-to-G base editing of chloroplast DNA in
plants. Nat Plants.

[259] Staub J. M., Maliga P. (1995). Marker rescue from
the Nicotiana
tabacum plastid genome using a plastid/Escherichia coli shuttle vector. Mol Gen Genet.

[260] Staub J. M., Maliga P. (1994). Extrachromosomal elements
in tobacco
plastids. Proc Natl Acad Sci U S A.

[261] Muhlbauer S. K., Lossl A., Tzekova L., Zou Z., Koop H. U. (2002). Functional
analysis of plastid DNA replication origins
in tobacco by targeted inactivation. Plant J.

[262] Min S. R., Davarpanah S. J., Jung S. H., Park Y.-i., Liu J. R., Jeong W.-J. (2015). An episomal vector system for plastid
transformation in higher plants. Plant Biotechnology
Reports.

[263] Occhialini A., Pfotenhauer A. C., Li L., Harbison S. A., Lail A. J., Burris J. N., Piasecki C., Piatek A. A., Daniell H., Stewart C. N. (2022). Mini-synplastomes for
plastid genetic engineering. Plant Biotechnol
J.

[264] Fox T. D., Sanford J. C., Mcmullin T. W. (1988). Plasmids Can Stably
Transform Yeast Mitochondria Lacking Endogenous Mtdna. P Natl Acad Sci USA.

[265] Johnston S. A., Anziano P. Q., Shark K., Sanford J. C., Butow R. A. (1988). Mitochondrial Transformation in Yeast
by Bombardment
with Microprojectiles. Science.

[266] Larosa V., Coosemans N., Motte P., Bonnefoy N., Remacle C. (2012). Reconstruction
of a human mitochondrial complex I mutation
in the unicellular green alga Chlamydomonas. Plant J.

[267] Remacle C., Cardol P., Coosemans N., Gaisne M., Bonnefoy N. (2006). High-efficiency biolistic transformation
of Chlamydomonas mitochondria can be used to insert mutations in complex
I genes. Proc Natl Acad Sci U S A.

[268] Gammage P. A., Moraes C. T., Minczuk M. (2018). Mitochondrial Genome
Engineering: The Revolution May Not Be CRISPR-Ized. Trends Genet.

[269] Mok B. Y., de Moraes M. H., Zeng J., Bosch D. E., Kotrys A. V., Raguram A., Hsu F., Radey M. C., Peterson S. B., Mootha V. K. (2020). A bacterial cytidine
deaminase toxin enables CRISPR-free mitochondrial base editing. Nature.

[270] Farre J. C., Araya A. (2001). Gene expression in
isolated plant
mitochondria: high fidelity of transcription, splicing and editing
of a transgene product in electroporated organelles. Nucleic Acids Res.

[271] Koulintchenko M., Konstantinov Y., Dietrich A. (2003). Plant mitochondria
actively import DNA via the permeability transition pore complex. Embo J.

[272] Buren S., Jiang X., Lopez-Torrejon G., Echavarri-Erasun C., Rubio L. M. (2017). Purification and In Vitro Activity
of Mitochondria Targeted Nitrogenase Cofactor Maturase NifB. Front Plant Sci.

[273] Kazama T., Okuno M., Watari Y., Yanase S., Koizuka C., Tsuruta Y., Sugaya H., Toyoda A., Itoh T., Tsutsumi N. (2019). Curing cytoplasmic male
sterility via TALEN-mediated mitochondrial genome editing. Nat Plants.

[274] Arimura S. I., Ayabe H., Sugaya H., Okuno M., Tamura Y., Tsuruta Y., Watari Y., Yanase S., Yamauchi T., Itoh T. (2020). Targeted gene disruption
of ATP synthases 6-1 and 6-2 in the mitochondrial genome of Arabidopsis
thaliana by mitoTALENs. Plant J.

[275] Forner J., Kleinschmidt D., Meyer E. H., Fischer A., Morbitzer R., Lahaye T., Schottler M. A., Bock R. (2022). Targeted introduction
of heritable point mutations into the plant
mitochondrial genome. Nat Plants.

[276] Smith H. O., Hutchison C. A., Pfannkoch C., Venter J. C. (2003). Generating a synthetic
genome by whole genome assembly:
phiX174 bacteriophage from synthetic oligonucleotides. Proc Natl Acad Sci U S A.

[277] Richardson S. M., Mitchell L. A., Stracquadanio G., Yang K., Dymond J. S., DiCarlo J. E., Lee D., Huang C. L., Chandrasegaran S., Cai Y. (2017). Design
of a synthetic yeast genome. Science.

[278] Zhao Y., Coelho C., Hughes A. L., Lazar-Stefanita L., Yang S., Brooks A. N., Walker R. S. K., Zhang W., Lauer S., Hernandez C. (2023). Debugging and consolidating
multiple synthetic chromosomes reveals combinatorial genetic interactions. Cell.

[279] Yu B. J., Sung B. H., Koob M. D., Lee C. H., Lee J. H., Lee W. S., Kim M. S., Kim S. C. (2002). Minimization
of the Escherichia coli genome using a Tn5-targeted Cre/loxP excision
system. Nat Biotechnol.

[280] Kolisnychenko V., Plunkett G., Herring C. D., Feher T., Posfai J., Blattner F. R., Posfai G. (2002). Engineering
a reduced Escherichia coli genome. Genome Res.

[281] Hashimoto M., Ichimura T., Mizoguchi H., Tanaka K., Fujimitsu K., Keyamura K., Ote T., Yamakawa T., Yamazaki Y., Mori H. (2005). Cell size
and nucleoid organization of engineered Escherichia coli cells with
a reduced genome. Mol Microbiol.

[282] Posfai G., Plunkett G., Feher T., Frisch D., Keil G. M., Umenhoffer K., Kolisnychenko V., Stahl B., Sharma S. S., de Arruda M. (2006). Emergent properties of reduced-genome Escherichia coli. Science.

[283] Hutchison C. A., Chuang R. Y., Noskov V. N., Assad-Garcia N., Deerinck T. J., Ellisman M. H., Gill J., Kannan K., Karas B. J., Ma L. (2016). Design
and synthesis of a minimal bacterial genome. Science.

[284] Lajoie M. J., Rovner A. J., Goodman D. B., Aerni H. R., Haimovich A. D., Kuznetsov G., Mercer J. A., Wang H. H., Carr P. A., Mosberg J. A. (2013). Genomically recoded
organisms expand biological functions. Science.

[285] Piatek A. A., Lenaghan S. C., Neal Stewart C. (2018). Advanced editing
of the nuclear and plastid genomes
in plants. Plant Sci.

[286] O'Neill B. M., Mikkelson K. L., Gutierrez N. M., Cunningham J. L., Wolff K. L., Szyjka S. J., Yohn C. B., Redding K. E., Mendez M. J. (2012). An exogenous chloroplast
genome for
complex sequence manipulation in algae. Nucleic
Acids Res.

[287] Scharff L. B., Bock R. (2014). Synthetic biology in plastids. Plant J.

[288] Gibson D. G., Smith H. O., Hutchison C. A., Venter J. C., Merryman C. (2010). Chemical synthesis
of the mouse mitochondrial genome. Nat Methods.

[289] Hirokawa Y., Kawano H., Tanaka-Masuda K., Nakamura N., Nakagawa A., Ito M., Mori H., Oshima T., Ogasawara N. (2013). Genetic manipulations
restored the
growth fitness of reduced-genome Escherichia coli. J Biosci Bioeng.

[290] Tanaka K., Henry C. S., Zinner J. F., Jolivet E., Cohoon M. P., Xia F., Bidnenko V., Ehrlich S. D., Stevens R. L., Noirot P. (2013). Building the repertoire
of dispensable
chromosome regions in Bacillus subtilis entails major refinement of
cognate large-scale metabolic model. Nucleic
Acids Res.

[291] Reuss D. R., Altenbuchner J., Mader U., Rath H., Ischebeck T., Sappa P. K., Thurmer A., Guerin C., Nicolas P., Steil L. (2017). Large-scale reduction
of the Bacillus subtilis genome: consequences for the transcriptional
network, resource allocation, and metabolism. Genome Res.

[292] Eleveld T. F., Bakali C., Eijk P. P., Stathi P., Vriend L. E., Poddighe P. J., Ylstra B. (2021). Engineering large-scale
chromosomal deletions by CRISPR-Cas9. Nucleic
Acids Res.

[293] Zhou H., Liu B., Weeks D. P., Spalding M. H., Yang B. (2014). Large chromosomal deletions and heritable small genetic changes induced
by CRISPR/Cas9 in rice. Nucleic Acids Res.

[294] Choi J. H., Chen W., Suiter C. C., Lee C., Chardon F. M., Yang W., Leith A., Daza R. M., Martin B., Shendure J. (2022). Precise genomic deletions using paired
prime editing. Nature Biotechnology.

[295] Annaluru N., Muller H., Mitchell L. A., Ramalingam S., Stracquadanio G., Richardson S. M., Dymond J. S., Kuang Z., Scheifele L. Z., Cooper E. M. (2014). Total synthesis of a
functional designer eukaryotic chromosome. Science.

[296] Schindler D., Walker R. S. K., Jiang S., Brooks A. N., Wang Y., Muller C. A., Cockram C., Luo Y., Garcia A., Schraivogel D. (2023). Design, construction,
and functional characterization of a tRNA neochromosome in yeast. Cell.

[297] Shao Y., Lu N., Wu Z., Cai C., Wang S., Zhang L. L., Zhou F., Xiao S., Liu L., Zeng X. (2018). Creating a functional single-chromosome yeast. Nature.

[298] Fredens J., Wang K., de la Torre D., Funke L. F. H., Robertson W. E., Christova Y., Chia T., Schmied W. H., Dunkelmann D. L., Beranek V. (2019). Total synthesis of Escherichia coli with a recoded
genome. Nature.

[299] Endy D. (2005). Foundations for engineering biology. Nature.

[300] Egelkrout E., Rajan V., Howard J. A. (2012). Overproduction
of
recombinant proteins in plants. Plant Sci.

[301] Mutalik V. K., Guimaraes J. C., Cambray G., Lam C., Christoffersen M. J., Mai Q. A., Tran A. B., Paull M., Keasling J. D., Arkin A. P. (2013). Precise and reliable
gene expression via standard transcription and translation initiation
elements. Nat Methods.

[302] Lou C., Stanton B., Chen Y. J., Munsky B., Voigt C. A. (2012). Ribozyme-based
insulator parts buffer synthetic circuits from genetic context. Nat Biotechnol.

[303] Hossain A., Lopez E., Halper S. M., Cetnar D. P., Reis A. C., Strickland D., Klavins E., Salis H. M. (2020). Automated
design of thousands of nonrepetitive parts for engineering stable
genetic systems. Nat Biotechnol.

[304] Parry G., Patron N., Bastow R., Matthewman C. (2016). Meeting report:
GARNet/OpenPlant CRISPR-Cas workshop. Plant
Methods.

[305] Coll A., Wilson M. L., Gruden K., Peccoud J. (2015). Rule-Based
Design of Plant Expression Vectors Using GenoCAD. PLoS One.

[306] Vazquez-Vilar M., Quijano-Rubio A., Fernandez-del-Carmen A., Sarrion-Perdigones A., Ochoa-Fernandez R., Ziarsolo P., Blanca J., Granell A., Orzaez D. (2017). GB3. 0: a
platform for plant bio-design
that connects functional DNA elements with associated biological data. Nucleic acids research.

[307] Kohli A., Twyman R. M., Abranches R., Wegel E., Stoger E., Christou P. (2003). Transgene integration,
organization and interaction in plants. Plant
Molecular Biology.

[308] Collier R., Dasgupta K., Xing Y. P., Hernandez B. T., Shao M., Rohozinski D., Kovak E., Lin J., de Oliveira M. L. P., Stover E. (2017). Accurate measurement
of transgene copy number in crop plants using droplet digital PCR. Plant Journal.

[309] Kosuri S., Goodman D. B., Cambray G., Mutalik V. K., Gao Y., Arkin A. P., Endy D., Church G. M. (2013). Composability of
regulatory sequences controlling transcription and translation in
Escherichia coli. P Natl Acad Sci USA.

[310] Nielsen A. A., Segall-Shapiro T. H., Voigt C. A. (2013). Advances in genetic
circuit design: novel biochemistries, deep part mining, and precision
gene expression. Curr Opin Chem Biol.

[311] Salis H. M., Mirsky E. A., Voigt C. A. (2009). Automated design
of synthetic ribosome binding sites to control protein expression. Nat Biotechnol.

[312] Zhou X., Carranco R., Vitha S., Hall T. C. (2005). The dark
side of green fluorescent protein. New Phytol.

[313] Hraška M., Rakouský S., Čurn V. (2006). Green fluorescent
protein as a vital marker for non-destructive detection of transformation
events in transgenic plants. Plant cell, tissue
and organ culture.

[314] Brophy J. A. N., Magallon K. J., Duan L., Zhong V., Ramachandran P., Kniazev K., Dinneny J. R. (2022). Synthetic genetic
circuits as a means of reprogramming plant roots. Science.

[315] Gonzalez-Grandio E., Demirer G. S., Ma W., Brady S., Landry M. P. (2021). A Ratiometric Dual Color Luciferase Reporter for Fast
Characterization of Transcriptional Regulatory Elements in Plants. ACS Synth Biol.

[316] Kinney J. B., Murugan A., Callan C. G., Cox E. C. (2010). Using deep
sequencing to characterize the biophysical mechanism of a transcriptional
regulatory sequence. P Natl Acad Sci USA.

[317] Schaumberg K. A., Antunes M. S., Kassaw T. K., Xu W., Zalewski C. S., Medford J. I., Prasad A. (2016). Quantitative characterization
of genetic parts and circuits for plant synthetic biology. Nat Methods.

[318] Reed K. M., Bargmann B. O. R. (2021). Protoplast Regeneration and Its Use
in New Plant Breeding Technologies. Front Genome
Ed.

[319] Lin Q. P., Zong Y., Xue C. X., Wang S. X., Jin S., Zhu Z. X., Wang Y. P., Anzalone A. V., Raguram A., Doman J. L. (2020). Prime
genome editing in rice and wheat. Nature Biotechnology.

[320] Jin S., Lin Q., Luo Y., Zhu Z., Liu G., Li Y., Chen K., Qiu J.-L., Gao C. (2021). Genome-wide specificity
of prime editors in plants. Nature Biotechnology.

[321] Shakhova, E. S. ; Markina, N. M. ; Mitiouchkina, T. ; Bugaeva, E. N. ; Karataeva, T. A. ; Palkina, K. A. ; Fakhranurova, L. I. ; Yampolsky, I. V. ; Sarkisyan, K. S. ; Mishin, A. S. Systematic Comparison of Plant Promoters in Nicotiana spp. Expression Systems. Int J Mol Sci 2022, 23 (23), 15441 10.3390/ijms232315441. 36499768 PMC9740895

[322] Liu W., Stewart C. N. (2016). Plant synthetic promoters and transcription
factors. Current opinion in biotechnology.

[323] Sonenberg N., Hinnebusch A. G. (2009). Regulation
of translation initiation
in eukaryotes: mechanisms and biological targets. Cell.

[324] Yamamoto Y. Y., Ichida H., Matsui M., Obokata J., Sakurai T., Satou M., Seki M., Shinozaki K., Abe T. (2007). Identification of plant promoter constituents by analysis of local
distribution of short sequences. BMC Genomics.

[325] Yamamoto Y. Y., Ichida H., Abe T., Suzuki Y., Sugano S., Obokata J. (2007). Differentiation of
core promoter
architecture between plants and mammals revealed by LDSS analysis. Nucleic Acids Res.

[326] Kumari S., Ware D. (2013). Genome-wide computational
prediction
and analysis of core promoter elements across plant monocots and dicots. PLoS One.

[327] Brooks E. G., Elorriaga E., Liu Y., Duduit J. R., Yuan G., Tsai C. J., Tuskan G. A., Ranney T. G., Yang X., Liu W. (2023). Plant Promoters and Terminators for
High-Precision Bioengineering. Biodes Res.

[328] Yaschenko A. E., Fenech M., Mazzoni-Putman S., Alonso J. M., Stepanova A. N. (2022). Deciphering the molecular basis of
tissue-specific gene expression in plants: Can synthetic biology help?. Curr Opin Plant Biol.

[329] Shahmuradov I. A., Gammerman A. J., Hancock J. M., Bramley P. M., Solovyev V. V. (2003). PlantProm:
A database of plant promoter sequences. Nucleic
Acids Research.

[330] Feike D., Korolev A. V., Soumpourou E., Murakami E., Reid D., Breakspear A., Rogers C., Radutoiu S., Stougaard J., Harwood W. A. (2019). Characterizing standard genetic parts and establishing
common principles for engineering legume and cereal roots. Plant Biotechnology Journal.

[331] Perez-Gonzalez, A. ; Caro, E. Benefits of using genomic insulators flanking transgenes to increase expression and avoid positional effects. Scientific Reports 2019, 9, 10.1038/s41598-019-44836-6. PMC6560062 31186481

[332] Lai X., Stigliani A., Vachon G., Carles C., Smaczniak C., Zubieta C., Kaufmann K., Parcy F. (2019). Building Transcription
Factor Binding Site Models to Understand Gene Regulation in Plants. Molecular Plant.

[333] Kelly J. R., Rubin A. J., Davis J. H., Ajo-Franklin C. M., Cumbers J., Czar M. J., de Mora K., Glieberman A. L., Monie D. D., Endy D. (2009). Measuring the activity
of BioBrick
promoters using an in vivo reference standard. J Biol Eng.

[334] Bush M. S., Hutchins A. P., Jones A. M. E., Naldrett M. J., Jarmolowski A., Lloyd C. W., Doonan J. H. (2009). Selective
recruitment
of proteins to 5′ cap complexes during the growth cycle in
Arabidopsis. Plant Journal.

[335] Smale S. T., Kadonaga J. T. (2003). The RNA polymerase
II core promoter. Annual Review of Biochemistry.

[336] Dutt M., Dhekney S. A., Soriano L., Kandel R., Grosser J. W. (2014). Temporal and spatial control of gene
expression in
horticultural crops. Horticulture Research.

[337] Vollen K., Zhao C., Alonso J. M., Stepanova A. N. (2024). Sourcing
DNA parts for synthetic biology applications in plants. Current Opinion in Biotechnology.

[338] Rushton P. J. (2016). What have we learned about synthetic promoter construction?. Plant synthetic promoters: methods and protocols.

[339] Zrimec J., Börlin C. S., Buric F., Muhammad A. S., Chen R., Siewers V., Verendel V., Nielsen J., Töpel M., Zelezniak A. (2020). Deep learning suggests that gene
expression is encoded in all parts of a co-evolving interacting gene
regulatory structure. Nature Communications.

[340] Sun Q., Zybailov B., Majeran W., Friso G., Olinares P. D. B., van Wijk K. J. (2009). PPDB, the Plant
Proteomics Database at Cornell. Nucleic Acids
Research.

[341] Shahmuradov, I. A. ; Umarov, R. K. ; Solovyev, V. V. TSSPlant: A new tool for prediction of plant Pol II promoters. Nucleic Acids Research 2017, 45 (8), gkw1353 10.1093/nar/gkw1353. PMC5416875 28082394

[342] Butaye K. M., Goderis I. J., Wouters P. F., Pues J. M., Delaure S. L., Broekaert W. F., Depicker A., Cammue B. P., De Bolle M. F. (2004). Stable high-level
transgene expression in Arabidopsis
thaliana using gene silencing mutants and matrix attachment regions. Plant J.

[343] Mlynarova L., Loonen A., Heldens J., Jansen R. C., Keizer P., Stiekema W. J., Nap J. P. (1994). Reduced
Position
Effect in Mature Transgenic Plants Conferred by the Chicken Lysozyme
Matrix-Associated Region. Plant Cell.

[344] Zhou A., Kirkpatrick L. D., Ornelas I. J., Washington L. J., Hummel N. F. C., Gee C. W., Tang S. N., Barnum C. R., Scheller H. V., Shih P. M. (2023). A Suite of Constitutive Promoters
for Tuning Gene Expression in Plants. ACS Synth
Biol.

[345] Tian C., Zhang Y., Li J., Wang Y. (2022). Benchmarking
Intrinsic Promoters and Terminators for Plant Synthetic Biology Research. Biodes Res.

[346] Venter M. (2007). Synthetic
promoters: genetic control through cis engineering. Trends in plant science.

[347] Amack S. C., Antunes M. S. (2020). CaMV35S promoter
- A plant biology
and biotechnology workhorse in the era of synthetic biology. Current Plant Biology.

[348] Kiselev K. V., Aleynova O. A., Ogneva Z. V., Suprun A. R., Dubrovina A. S. (2021). 35S promoter-driven transgenes are variably expressed
in different organs of Arabidopsis thaliana and in response to abiotic
stress. Mol Biol Rep.

[349] Gupta D., Dey N., Leelavathi S., Ranjan R. (2021). Development of efficient synthetic promoters derived
from pararetrovirus suitable for translational research. Planta.

[350] Comai L., Moran P., Maslyar D. (1990). Novel and useful properties
of a chimeric plant promoter combining CaMV 35S and MAS elements. Plant Mol Biol.

[351] Ni M., Cui D., Einstein J., Narasimhulu S., Vergara C. E., Gelvin S. B. (1995). Strength and tissue
specificity of
chimeric promoters derived from the octopine and mannopine synthase
genes. The Plant Journal.

[352] Dey N., Maiti I. B. (1999). Structure and promoter/leader
deletion analysis of
mirabilis mosaic virus (MMV) full-length transcript promoter in transgenic
plants. Plant Molecular Biology.

[353] Odell J. T., Nagy F., Chua N. H. (1985). Identification
of
DNA sequences required for activity of the cauliflower mosaic virus
35S promoter. Nature.

[354] Ranjan R., Patro S., Pradhan B., Kumar A., Maiti I. B., Dey N. (2012). Development and functional
analysis
of novel genetic promoters using DNA shuffling, hybridization and
a combination thereof. PLoS One.

[355] Kummari, D. ; Palakolanu, S. R. ; Kishor, P. B. K. ; Bhatnagar-Mathur, P. ; Singam, P. ; Vadez, V. ; Sharma, K. K. An update and perspectives on the use of promoters in plant genetic engineering. Journal of Biosciences 2020, 45 (1), 10.1007/s12038-020-00087-6. 33097676

[356] Cai Y.-M., Kallam K., Tidd H., Gendarini G., Salzman A., Patron N. J (2020). Rational design of
minimal synthetic
promoters for plants. Nucleic Acids Research.

[357] Jores T., Tonnies J., Wrightsman T., Buckler E. S., Cuperus J. T., Fields S., Queitsch C. (2021). Synthetic
promoter designs enabled by a comprehensive analysis of plant core
promoters. Nat Plants.

[358] Segall-Shapiro T. H., Sontag E. D., Voigt C. A. (2018). Engineered
promoters
enable constant gene expression at any copy number in bacteria. Nat Biotechnol.

[359] Kushwaha, M. ; Salis, H. M. A portable expression resource for engineering cross-species genetic circuits and pathways. Nature Communications 2015, 6, 10.1038/ncomms8832. PMC4518296 26184393

[360] Yasmeen E., Wang J., Riaz M., Zhang L., Zuo K. (2023). Designing artificial synthetic promoters
for accurate, smart, and
versatile gene expression in plants. Plant Commun.

[361] Zrimec J., Fu X., Muhammad A. S., Skrekas C., Jauniskis V., Speicher N. K., Borlin C. S., Verendel V., Chehreghani M. H., Dubhashi D. (2022). Controlling
gene expression
with deep generative design of regulatory DNA. Nat Commun.

[362] Bernard V., Brunaud V., Lecharny A. (2010). TC-motifs at the TATA-box
expected position in plant genes: a novel class of motifs involved
in the transcription regulation. BMC genomics.

[363] Civan P., Svec M. (2009). Genome-wide analysis
of rice (Oryza
sativa L. subsp. japonica) TATA box and Y Patch promoter elements. Genome.

[364] Mehrotra R., Mehrotra S. (2010). Promoter activation by ACGT in response
to salicylic and abscisic acids is differentially regulated by the
spacing between two copies of the motif. J Plant
Physiol.

[365] Belcher M. S., Vuu K. M., Zhou A., Mansoori N., Agosto Ramos A., Thompson M. G., Scheller H. V., Loqué D., Shih P. M. (2020). Design of orthogonal regulatory systems for modulating
gene expression in plants. Nat Chem Biol.

[366] Amack S. C., Ferreira S. S., Antunes M. S. (2023). Tuning
the Transcriptional
Activity of the CaMV 35S Promoter in Plants by Single-Nucleotide Changes
in the TATA Box. ACS Synth Biol.

[367] Maqbool S. B., Christou P. (1999). Multiple traits of
agronomic importance
in transgenic indica rice plants: analysis of transgene integration
patterns, expression levels and stability. Molecular
Breeding.

[368] Aysha J., Noman M., Wang F., Liu W., Zhou Y., Li H., Li X. (2018). Synthetic Promoters:
Designing the cis Regulatory Modules for Controlled Gene Expression. Mol Biotechnol.

[369] Rushton P. J., Reinstadler A., Lipka V., Lippok B., Somssich I. E. (2002). Synthetic plant
promoters containing defined regulatory
elements provide novel insights into pathogen- and wound-induced signaling. Plant Cell.

[370] Liu W., Mazarei M., Rudis M. R., Fethe M. H., Stewart C. N. (2011). Rapid in vivo analysis of synthetic promoters for plant
pathogen phytosensing. BMC Biotechnol.

[371] Yamaguchi-Shinozaki K., Shinozaki K. (1994). A novel cis-acting
element in an
Arabidopsis gene is involved in responsiveness to drought, low-temperature,
or high-salt stress. Plant Cell.

[372] Kirsch C., Logemann E., Lippok B., Schmelzer E., Hahlbrock K. (2001). A highly specific pathogen-responsive
promoter element
from the immediate-early activated CMPG1 gene in Petroselinum crispum. Plant J.

[373] Abe H., Yamaguchi-Shinozaki K., Urao T., Iwasaki T., Hosokawa D., Shinozaki K. (1997). Role of arabidopsis
MYC and MYB homologs
in drought- and abscisic acid-regulated gene expression. Plant Cell.

[374] Shen Q., Zhang P., Ho T. H. (1996). Modular
nature of
abscisic acid (ABA) response complexes: composite promoter units that
are necessary and sufficient for ABA induction of gene expression
in barley. Plant Cell.

[375] Puente P., Wei N., Deng X. W. (1996). Combinatorial
interplay
of promoter elements constitutes the minimal determinants for light
and developmental control of gene expression in Arabidopsis. EMBO J.

[376] Sun C., Palmqvist S., Olsson H., Boren M., Ahlandsberg S., Jansson C. (2003). A novel WRKY transcription factor, SUSIBA2, participates
in sugar signaling in barley by binding to the sugar-responsive elements
of the iso1 promoter. Plant Cell.

[377] Storozhenko S., De Pauw P., Van Montagu M., Inze D., Kushnir S. (1998). The heat-shock
element is a functional
component of the Arabidopsis APX1 gene promoter. Plant Physiol.

[378] Higo K., Ugawa Y., Iwamoto M., Korenaga T. (1999). Plant cis-acting
regulatory DNA elements (PLACE) database: 1999. Nucleic Acids Res.

[379] Matys V., Fricke E., Geffers R., Gossling E., Haubrock M., Hehl R., Hornischer K., Karas D., Kel A. E., Kel-Margoulis O. V. (2003). TRANSFAC: transcriptional regulation, from patterns to profiles. Nucleic Acids Res.

[380] Lescot M., Dehais P., Thijs G., Marchal K., Moreau Y., Van de Peer Y., Rouze P., Rombauts S. (2002). PlantCARE,
a database of plant cis-acting regulatory elements and a portal to
tools for in silico analysis of promoter sequences. Nucleic Acids Res.

[381] Wang R., Zhu M., Ye R., Liu Z., Zhou F., Chen H., Lin Y. (2016). Novel green tissue-specific
synthetic promoters and cis-regulatory elements in rice. Sci Rep.

[382] Roccaro M., Ahmadinejad N., Colby T., Somssich I. E. (2013). Identification
of functional cis-regulatory elements by sequential enrichment from
a randomized synthetic DNA library. BMC Plant
Biol.

[383] Koschmann J., Machens F., Becker M., Niemeyer J., Schulze J., Bülow L., Stahl D. J., Hehl R. (2012). Integration
of Bioinformatics and Synthetic Promoters Leads to the Discovery of
Novel Elicitor-Responsive cis-Regulatory Sequences in Arabidopsis. Plant Physiology.

[384] Peleke F. F., Zumkeller S. M., Gültas M., Schmitt A., Szymański J. (2024). Deep learning
the cis-regulatory
code for gene expression in selected model plants. Nature Communications.

[385] Cai Y. M., Kallam K., Tidd H., Gendarini G., Salzman A., Patron N. J. (2020). Rational design of minimal synthetic
promoters for plants. Nucleic Acids Res.

[386] Sawant S. V., Kiran K., Mehrotra R., Chaturvedi C. P., Ansari S. A., Singh P., Lodhi N., Tuli R. (2005). A variety
of synergistic and antagonistic interactions mediated by cis-acting
DNA motifs regulate gene expression in plant cells and modulate stability
of the transcription complex formed on a basal promoter. J Exp Bot.

[387] Wan X., Pinto F., Yu L., Wang B. (2020). Synthetic protein-binding
DNA sponge as a tool to tune gene expression and mitigate protein
toxicity. Nature communications.

[388] Del Vecchio, D. ; Dy, A. J. ; Qian, Y. Control theory meets synthetic biology. J R Soc Interface 2016, 13 (120), 20160380. 10.1098/rsif.2016.0380 27440256 PMC4971224

[389] Zhang S., Voigt C. A. (2018). Engineered dCas9 with reduced toxicity
in bacteria: implications for genetic circuit design. Nucleic Acids Res.

[390] Chen, P.-Y. ; Qian, Y. ; Del Vecchio, D. A model for resource competition in CRISPR-mediated gene repression. In 2018 IEEE Conference on Decision and Control (CDC); 2018; IEEE: pp 4333-4338.

[391] Shrestha A., Khan A., Dey N. (2018). cis-trans Engineering:
Advances and Perspectives on Customized Transcriptional Regulation
in Plants. Mol Plant.

[392] Li B., Gaudinier A., Tang M., Taylor-Teeples M., Nham N. T., Ghaffari C., Benson D. S., Steinmann M., Gray J. A., Brady S. M. (2014). Promoter-based integration
in plant defense regulation. Plant Physiol.

[393] Gupta S., Kesarwani V., Bhati U., Jyoti, Shankar R. (2024). PTFSpot: deep co-learning
on transcription factors and their binding regions attains impeccable
universality in plants. Briefings in Bioinformatics.

[394] Matsuoka M., Kyozuka J., Shimamoto K., Kano-Murakami Y. (1994). The promoters of two carboxylases in a C4 plant (maize)
direct cell-specific, light-regulated expression in a C3 plant (rice). Plant J.

[395] Streatfield S. J., Magallanes-Lundback M.
E., Beifuss K. K., Brooks C. A., Harkey R. L., Love R. T., Bray J., Howard J. A., Jilka J. M., Hood E. E. (2004). Analysis of the
maize polyubiquitin-1 promoter heat shock elements and generation
of promoter variants with modified expression characteristics. Transgenic Res.

[396] Carvalho P., Gomes C., Saibo N. J. M. (2023). C4 Phosphoenolpyruvate
Carboxylase: Evolution and transcriptional regulation. Genet Mol Biol.

[397] Hamilton D. A., Schwarz Y. H., Mascarenhas J. P. (1998). A monocot
pollen-specific promoter contains separable pollen-specific and quantitative
elements. Plant Mol Biol.

[398] Taniguchi M., Izawa K., Ku M. S., Lin J. H., Saito H., Ishida Y., Ohta S., Komari T., Matsuoka M., Sugiyama T. (2000). The promoter for the
maize C4 pyruvate,
orthophosphate dikinase gene directs cell- and tissue-specific transcription
in transgenic maize plants. Plant Cell Physiol.

[399] McElroy D., Zhang W., Cao J., Wu R. (1990). Isolation
of an efficient actin promoter for use in rice transformation. Plant Cell.

[400] Assem S. K., El-Itriby H. A., Hussein E., Saad M., Madkour M. A. (2002). Comparison of the efficiency of some novel maize promoters
in monocot and dicot plants. Arab J. Biotechnol.

[401] Iwamoto M., Higo H., Higo K. (2004). Strong expression
of
the rice catalase gene CatB promoter in protoplasts and roots of both
a monocot and dicots. Plant Physiology and Biochemistry.

[402] Khan M., Makhdoom R., Husnain T., Saleem M., Malik K., Latif Z., Altosaar I., Riazuddin S. (2001). Expression
of Bt gene in a dicot plant under promoter derived from a monocot
plant. Pak J Biol Sci.

[403] Luan S., Bogorad L. (1992). A rice cab gene promoter
contains
separate cis-acting elements that regulate expression in dicot and
monocot plants. The Plant Cell.

[404] Biłas R., Szafran K., Hnatuszko-Konka K., Kononowicz A. K. (2016). Cis-regulatory elements used to control gene expression
in plants. Plant Cell, Tissue and Organ Culture
(PCTOC).

[405] Wilmink A., Van de Ven B., Dons J. (1995). Activity of constitutive
promoters in various species from the Liliaceae. Plant molecular biology.

[406] McElroy D., Brettell R. I. (1994). Foreign gene expression in transgenic
cereals. Trends in Biotechnology.

[407] Schenk P. M., Sagi L., Remans T., Dietzgen R. G., Bernard M. J., Graham M. W., Manners J. M. (1999). A promoter
from
sugarcane bacilliform badnavirus drives transgene expression in banana
and other monocot and dicot plants. Plant Mol
Biol.

[408] Yanagisawa S., Izui K. (1992). MNF1, a leaf tissue-specific DNA-binding
protein of maize, interacts with the cauliflower mosaic virus 35S
promoter as well as the C4 photosynthetic phosphoenolpyruvate carboxylase
gene promoter. Plant Molecular Biology.

[409] Pogue G. P., Holzberg S. (2012). Transient virus expression systems
for recombinant protein expression in dicot-and monocotyledonous plants. Plant Science.

[410] Shirasawa-Seo N., Sano Y., Nakamura S., Murakami T., Gotoh Y., Naito Y., Hsia C. N., Seo S., Mitsuhara I., Kosugi S. (2005). The promoter of Milk
vetch dwarf virus component 8 confers effective gene expression in
both dicot and monocot plants. Plant Cell Rep.

[411] Beringer J., Chen W., Garton R., Sardesai N., Wang P. H., Zhou N., Gupta M., Wu H. (2017). Comparison
of the impact of viral and plant-derived promoters regulating selectable
marker gene on maize transformation and transgene expression. Plant Cell Rep.

[412] Al-Saady N. A., Gopalraj M., Olszewski N. E., Somers D. A. (2010). Deletion analysis of the sugarcane bacilliform virus
promoter activity in monocot and dicot plants. Biotechnology.

[413] Keith B., Chua N. H. (1986). Monocot and dicot pre-mRNAs are processed
with different efficiencies in transgenic tobacco. EMBO J.

[414] Chandra, S. ; Leon, R. G. Genome-Wide Evolutionary Analysis of Putative Non-Specific Herbicide Resistance Genes and Compilation of Core Promoters between Monocots and Dicots. In Genes, 2022; Vol. 13, 1171. 10.3390/genes13071171 35885954 PMC9316059

[415] Khan Z. H., Patel R., Mehrotra S., Mehrotra R. (2019). In-silico
analysis of seed storage protein gene promoters reveals differential
occurrence of 7 cis-regulatory elements in monocot and 14 in dicot
plants. Gene Reports.

[416] Sharma A. K., Sharma M. K. (2009). Plants as bioreactors: recent developments
and emerging opportunities. Biotechnology advances.

[417] Abu
El-Heba G. A., Hussein G. M., Fahmy I. F., Abdou S. M., Faisal A., Taha O., Abdallah N. A. (2015). Impact of cis-acting
elements' frequency in transcription activity in dicot and monocot
plants. 3 Biotech.

[418] Ishige F., Takaichi M., Foster R., Chua N. H., Oeda K. (1999). AG-box motif (GCCACGTGCC) tetramer
confers high-level constitutive
expression in dicot and monocot plants. The
Plant Journal.

[419] Hauptmann R. M., Ashraf M., Vasil V., Hannah L. C., Vasil I. K., Ferl R. (1988). Promoter strength comparisons
of
maize shrunken 1 and alcohol dehydrogenase 1 and 2 promoters in mono-
and dicotyledonous species. Plant Physiol.

[420] Mitsuhara I., Ugaki M., Hirochika H., Ohshima M., Murakami T., Gotoh Y., Katayose Y., Nakamura S., Honkura R., Nishimiya S. (1996). Efficient promoter cassettes for enhanced expression
of foreign genes
in dicotyledonous and monocotyledonous plants. Plant Cell Physiol.

[421] Kim H. U., Chung T. Y. (1997). Characterization
of three anther-specific
genes isolated from Chinese cabbage. Plant molecular
biology.

[422] Jeong H. J., Jung K. H. (2015). Rice tissue-specific promoters and
condition-dependent promoters for effective translational application. Journal of integrative plant biology.

[423] Li H., Pinot F., Sauveplane V., Werck-Reichhart D., Diehl P., Schreiber L., Franke R., Zhang P., Chen L., Gao Y. (2010). Cytochrome
P450 family member CYP704B2
catalyzes the ω-hydroxylation of fatty acids and is required
for anther cutin biosynthesis and pollen exine formation in rice. The Plant Cell.

[424] Potenza C., Aleman L., Sengupta-Gopalan C. (2004). Targeting
transgene expression in research, agricultural, and environmental
applications: promoters used in plant transformation. In vitro cellular & developmental biology-Plant.

[425] Jeon J.-S., Chung Y.-Y., Lee S., Yi G.-H., Oh B.-G., An G. (1999). Isolation and characterization
of
an anther-specific gene, RA8, from rice (Oryza sativa L.). Plant molecular biology.

[426] Chen L., Li Y., Wang Y., Li W., Feng X., Zhao L. (2021). Use of High Resolution Spatiotemporal
Gene Expression Data to Uncover Novel Tissue-Specific Promoters in
Tomato. Agriculture.

[427] Smirnova O. G., Kochetov A. V. (2020). Choice of the promoter
for tissue
and developmental stage-specific gene expression. Biolistic DNA Delivery in Plants: Methods and Protocols.

[428] Hsu S.-W., Liu M.-C., Zen K.-C., Wang C.-S. (2014). Identification
of the tapetum/microspore-specific promoter of the pathogenesis-related
10 gene and its regulation in the anther of Lilium longiflorum. Plant Science.

[429] Luo H., Lee J.-Y., Hu Q., Nelson-Vasilchik K., Eitas T. K., Lickwar C., Kausch A. P., Chandlee J. M., Hodges T. K. (2006). RTS, a rice anther-specific gene
is required for male
fertility and its promoter sequence directs tissue-specific gene expression
in different plant species. Plant molecular
biology.

[430] Suzuki K.-i., Yun D.-J., Chen X.-Y., Yamada Y., Hashimoto T. (1999). An Atropa
belladonna hyoscyamine 6β-hydroxylase
gene is differentially expressed in the root pericycle and anthers. Plant Molecular Biology.

[431] NAKAJIMA K., OSHITA Y., KAYA M., YAMADA Y., HASHIMOTO T. (1999). Structures and Expression Patterns
of Two Tropinone
Reductase Genes from Hyoscyamus niger. Bioscience,
Biotechnology, and Biochemistry.

[432] Liu M.-C., Yang C.-S., Yeh F.-L., Wei C.-H., Jane W.-N., Chung M.-C., Wang C.-S. (2014). A novel
lily anther-specific
gene encodes adhesin-like proteins associated with exine formation
during anther development. Journal of experimental
botany.

[433] Koltunow A. M., Truettner J., Cox K. H., Wallroth M., Goldberg R. B. (1990). Different
temporal and spatial gene expression patterns
occur during anther development. The Plant Cell.

[434] Jiang S.-Y., Ramamoorthy R., Bhalla R., Luan H.-F., Venkatesh P. N., Cai M., Ramachandran S. (2008). Genome-wide
survey of the RIP domain family in Oryza sativa and their expression
profiles under various abiotic and biotic stresses. Plant molecular biology.

[435] Paul W., Hodge R., Smartt S., Draper J., Scott R. (1992). The isolation and characterisation of the tapetum-specific Arabidopsis
thaliana A9 gene. Plant molecular biology.

[436] Zierer W., Anjanappa R. B., Lamm C. E., Chang S.-H., Gruissem W., Sonnewald U. (2022). A promoter
toolbox for tissue-specific
expression supporting translational research in cassava (Manihot esculenta). Frontiers in Plant Science.

[437] Lu W., Tang X., Huo Y., Xu R., Qi S., Huang J., Zheng C., Wu C.-a. (2012). Identification and
characterization of fructose 1, 6-bisphosphate aldolase genes in Arabidopsis
reveal a gene family with diverse responses to abiotic stresses. Gene.

[438] Mitra A., Han J., Zhang Z. J., Mitra A. (2009). The intergenic
region of Arabidopsis thaliana cab 1 and cab 2 divergent genes functions
as a bidirectional promoter. Planta.

[439] Kyozuka J., McElroy D., Hayakawa T., Xie Y., Wu R., Shimamoto K. (1993). Light-regulated and cell-specific
expression of tomato
rbcS-gusA and rice rbcS-gusA fusion genes in transgenic rice. Plant physiology.

[440] Dong H., Liu L., Fan X., Asghar S., Li Y., Wang Y., Xu X., Wu T., Zhang X., Qiu C. (2019). The artificial promoter rMdAG2I confers
flower-specific activity
in malus. International Journal of Molecular
Sciences.

[441] Savidge B., Rounsley S. D., Yanofsky M. F. (1995). Temporal relationship
between the transcription of two Arabidopsis MADS box genes and the
floral organ identity genes. The Plant Cell.

[442] Manavella P. A., Chan R. L. (2006). Development of tissue-specific promoters
for plant transformation. Ornamental Plant Biotechnol.

[443] Lim C. J., Lee H. Y., Kim W. B., Lee B.-S., Kim J., Ahmad R., Kim H. A., Yi S. Y., Hur C.-G., Kwon S.-Y. (2012). Screening of Tissue-Specific
Genes and Promoters in
Tomato by Comparing Genome Wide Expression Profiles of Arabidopsis
Orthologues. Molecules and Cells.

[444] Endo T., Shimada T., Fujii H., Moriguchi T., Omura M. (2007). Promoter analysis of a type 3 metallothionein-like
gene abundant
in Satsuma mandarin (Citrus unshiu Marc.) fruit. Scientia Horticulturae.

[445] Pattanaik S., Kong Q., Zaitlin D., Werkman J. R., Xie C. H., Patra B., Yuan L. (2010). Isolation
and functional
characterization of a floral tissue-specific R2R3 MYB regulator from
tobacco. Planta.

[446] Li Y., Dong C., Hu M., Bai Z., Tong C., Zuo R., Liu Y., Cheng X., Cheng M., Huang J. (2019). Identification
of flower-specific promoters through comparative transcriptome analysis
in Brassica napus. International journal of
molecular sciences.

[447] Kim D.-H., Park S., Lee J.-Y., Ha S.-H., Lim S.-H. (2018). Enhancing flower color through simultaneous expression
of the B-peru and mPAP1 transcription factors under control of a flower-specific
promoter. International journal of molecular
sciences.

[448] Livramento K. G. d., Freitas N. C., Trindade L. d. O. R., Teixeira L. G. d. S., Paiva L. V., Bordallo P. d. N., Diniz L. E. C. (2021). Gene
expression analysis associated with tissue-specific promoters in Musa
spp. Acta Scientiarum. Agronomy.

[449] Schaart J., Salentijn E., Krens F. (2002). Tissue-specific expression
of the β-glucuronidase reporter gene in transgenic strawberry
(Fragaria× ananassa) plants. Plant Cell
Reports.

[450] Wen Z., Yang Y., Zhang J., Wang X., Singer S., Liu Z., Yang Y., Yan G., Liu Z. (2014). Highly interactive
nature of flower-specific enhancers and promoters, and its potential
impact on tissue-specific expression and engineering of multiple genes
or agronomic traits. Plant biotechnology journal.

[451] Moon H., Callahan A. M. (2004). Developmental regulation
of peach
ACC oxidase promoter-GUS fusions in transgenic tomato fruits. Journal of experimental botany.

[452] Du L., Lou Q., Zhang X., Jiao S., Liu Y., Wang Y. (2014). Construction of flower-specific
chimeric promoters and analysis of
their activities in transgenic torenia. Plant
molecular biology reporter.

[453] Twell D., Wing R., Yamaguchi J., McCormick S. (1989). Isolation and expression of an anther-specific gene
from tomato. Molecular and general genetics
Mgg.

[454] Sassa H., Ushijima K., Hirano H. (2002). A pistil-specific thaumatin/PR5-like
protein gene of Japanese pear (Pyrus serotina): sequence and promoter
activity of the 5′ region in transgenic tobacco. Plant molecular biology.

[455] Ribeiro T. P., Basso M. F., de Carvalho M. H., de Macedo L. L. P., da Silva D. M. L., Lourenço-Tessutti I. T., de Oliveira-Neto O. B., de Campos-Pinto E. R., Lucena W. A., da Silva M. C. M. (2019). Stability
and tissue-specific Cry10Aa overexpression improves cotton resistance
to the cotton boll weevil. Biotechnology Research
and Innovation.

[456] Artico S., Ribeiro-Alves M., Oliveira-Neto O. B., de Macedo L. L. P., Silveira S., Grossi-de-Sa M. F., Martinelli A. P., Alves-Ferreira M. (2014). Transcriptome analysis of Gossypium
hirsutum flower buds infested by cotton boll weevil (Anthonomus grandis)
larvae. BMC Genomics.

[457] Yang Y., Singer S. D., Liu Z. (2011). Evaluation and comparison
of the insulation efficiency of three enhancer-blocking insulators
in plants. Plant Cell, Tissue and Organ Culture
(PCTOC).

[458] Kim J.-S., Ezura K., Lee J., Ariizumi T., Ezura H. (2019). Genetic engineering
of parthenocarpic tomato plants using transient
SlIAA9 knockdown by novel tissue-specific promoters. Scientific Reports.

[459] Mann V., Harker M., Pecker I., Hirschberg J. (2000). Metabolic
engineering of astaxanthin production in tobacco flowers. Nature biotechnology.

[460] Golovkin M., Reddy A. S. N. (2003). Expression of
U1 Small Nuclear Ribonucleoprotein
70K Antisense Transcript Using APETALA3 Promoter Suppresses the Development
of Sepals and Petals. Plant Physiology.

[461] Jack T., Brockman L. L., Meyerowitz E. M. (1992). The homeotic
gene APETALA3 of Arabidopsis thaliana encodes a MADS box and is expressed
in petals and stamens. Cell.

[462] Tilly J. J., Allen D. W., Jack T. (1998). The CArG boxes
in the
promoter of the Arabidopsis floral organ identity gene APETALA3 mediate
diverse regulatory effects. Development.

[463] Xu X., Jiang C.-Z., Donnelly L., Reid M. S. (2007). Functional analysis
of a RING domain ankyrin repeat protein that is highly expressed during
flower senescence. Journal of Experimental Botany.

[464] Benfey P. N., Takatsuji H., Ren L., Shah D. M., Chua N. H. (1990). Sequence
Requirements of the 5-Enolpyruvylshikimate-3-phosphate
Synthase 5[prime]-Upstream Region for Tissue-Specific Expression in
Flowers and Seedlings. The Plant Cell.

[465] Hatton D., Smith C., Bevan M. (1996). Tissue-specific expression
of the PAL3 promoter requires the interaction of two conserved cis
sequences. Plant Molecular Biology.

[466] Faktor O., Kooter J. M., Dixon R. A., Lamb C. J. (1996). Functional
dissection of a bean chalcone synthase gene promoter in transgenic
tobacco plants reveals sequence motifs essential for floral expression. Plant Molecular Biology.

[467] Sato T., Thorsness M. K., Kandasamy M. K., Nishio T., Hirai M., Nasrallah J. B., Nasrallah M. E. (1991). Activity of an S Locus Gene Promoter in Pistils and
Anthers of Transgenic Brassica. The Plant Cell.

[468] Ficker M., Wemmer T., Thompson R. D. (1997). A promoter directing
high level expression in pistils of transgenic plants. Plant Molecular Biology.

[469] Annadana S., Beekwilder M. J., Kuipers G., Visser P. B., Outchkourov N., Pereira A., Udayakumar M., De Jong J., Jongsma M. A. (2002). Cloning
of the Chrysanthemum UEP1
Promoter and Comparative Expression in Florets and Leaves of Dendranthema
Grandiflora. Transgenic Research.

[470] Kim S.-H., Lee J.-R., Kim S.-R. (2006). Characterization
of an apple anthocyanidin synthase gene in transgenic tobacco plants. Journal of Plant Biology.

[471] Dalal M., Chinnusamy V., Bansal K. C. (2010). Isolation and functional
characterization of Lycopene β-cyclase (CYC-B) promoter from
Solanum habrochaites. BMC Plant Biology.

[472] Nishikawa F., Endo T., Shimada T., Fujii H., Shimizu T., Omura M. (2008). Isolation and characterization
of
a Citrus FT/TFL1 homologue (CuMFT1), which shows quantitatively preferential
expression in Citrus seeds. Journal of the Japanese
Society for Horticultural Science.

[473] Borghi M., Xie D.-Y. (2016). Tissue-specific
production of limonene
in Camelina sativa with the Arabidopsis promoters of genes BANYULS
and FRUITFULL. Planta.

[474] Nguyen, A. L. Transcriptional regulation of FRUITFULL: a MADS-box gene involved in Arabidopsis fruit development. UC San Diego, 2008.

[475] Atkinson R. G., Bolitho K. M., Wright M. A., Iturriagagoitia-Bueno T., Reid S. J., Ross G. S. (1998). Apple ACC-oxidase and polygalacturonase:
ripening-specific gene expression and promoter analysis in transgenic
tomato. Plant Molecular Biology.

[476] Bara ski R., Puddephat I. J. (2004). Tissue
specific expression of β-glucuronidase
gene driven by heterologous promoters in transgenic cauliflower plants. Acta Physiologiae Plantarum.

[477] Unni, S. C. ; Vivek, P. J. ; Maju, T. T. ; Varghese, R. T. ; Soniya, E. V. Molecular cloning and characterization of fruit specific promoter from Cucumis sativus L. AJMB 2012, 02, 132. 10.4236/ajmb.2012.22015

[478] Spolaore S., Trainotti L., Pavanello A., Casadoro G. (2003). Isolation and promoter analysis of two genes encoding
different endo-β-1,4-glucanases in the non-climacteric strawberry1. Journal of Experimental Botany.

[479] Corona V., Aracri B., Kosturkova G., Bartley G. E., Pitto L., Giorgetti L., Scolnik P. A., Giuliano G. (1996). Regulation of a carotenoid biosynthesis
gene promoter during plant development. The
Plant Journal.

[480] Deikman J., Fischer R. L. (1988). Interaction of a
DNA binding factor
with the 5′-flanking region of an ethylene-responsive fruit
ripening gene from tomato. The EMBO Journal.

[481] Fraser P. D., Romer S., Shipton C. A., Mills P. B., Kiano J. W., Misawa N., Drake R. G., Schuch W., Bramley P. M. (2002). Evaluation
of transgenic tomato plants expressing an
additional phytoene synthase in a fruit-specific manner. Proceedings of the National Academy of Sciences.

[482] Saed Taha R., Ismail I., Zainal Z., Abdullah S. N. A. (2012). The
stearoyl-acyl-carrier-protein desaturase promoter (Des) from oil palm
confers fruit-specific GUS expression in transgenic tomato. Journal of Plant Physiology.

[483] Yamagata H., Yonesu K., Hirata A., Aizono Y. (2002). TGTCACA Motif
Is a Novel cis-Regulatory Enhancer Element Involved in Fruit-specific
Expression of the cucumisin Gene*. Journal of
Biological Chemistry.

[484] Langenkämper G., Manac'h N., Broin M., Cuiné S., Becuwe N., Kuntz M., Rey P. (2001). Accumulation of plastid
lipid-associated proteins (fibrillin/CDSP34) upon oxidative stress,
ageing and biotic stress in Solanaceae and in response to drought
in other species. Journal of Experimental Botany.

[485] Kuntz, Chen, Simkin, Römer, Shipton, Drake, Schuch, Bramley (1998). Upregulation of two ripening-related
genes from a non-climacteric plant (pepper) in a transgenic climacteric
plant (tomato). The Plant Journal.

[486] Wu H.-Y., Liu J.-M., Yang X.-T., Zhu Z.-J., Shou S.-Y. (2003). [Primary targeting of functional
regions involved in
transcriptional regulation on watermelon fruit-specific promoter WSP]. Sheng Wu Gong Cheng Xue Bao.

[487] Wu H. Y., Liu J. M., Zhu Z. J., Yang X. T., Chen D. M. (2003). [Transformation of wml1 5 promoter
region into tomato
plants and studies on its transcriptional regulation role]. Shi Yan Sheng Wu Xue Bao.

[488] Yin T., Wu H., Zhang S., Liu J., Lu H., Zhang L., Xu Y., Chen D. (2008). Two negative
cis-regulatory
regions involved in fruit-specific promoter activity from watermelon
(Citrullus vulgaris S.). Journal of Experimental
Botany.

[489] Nishikawa F., Endo T., Shimada T., Fujii H., Shimizu T., Omura M. (2008). Isolation and Characterization of
a <i>Citrus FT/TFL1</i> Homologue (<i>CuMFT1</i>),
Which
Shows Quantitatively Preferential Expression in <i>Citrus</i>
Seeds. Journal of the Japanese Society for Horticultural
Science.

[490] Zubaidah R. (2018). Tissue-specific promoters: the importance and potential
application for genetic engineering in oil palm. Journal of Oil Palm Research.

[491] Ramli Z., Abdullah S. N. A. (2010). Functional Characterisation
of the
Oil Palm Type 3 Metallothionein-like Gene (MT3-B) Promoter. Plant Molecular Biology Reporter.

[492] Konda A. K., Singh P., Soren K. R., Singh N. P. (2019). A modified
pod specific promoter for high level heterologous expression of genes
in legumes. Legume Research-An International
Journal.

[493] Agius F., Amaya I., Botella M. A., Valpuesta V. (2004). Functional
analysis of homologous and heterologous promoters in strawberry fruits
using transient expression*. Journal of Experimental
Botany.

[494] Chen H.-C., Klein A., Xiang M., Backhaus R. A., Kuntz M. (1998). Drought- and
wound-induced expression in leaves of a gene encoding
a chromoplast carotenoid-associated protein. The Plant Journal.

[495] Xiao Y.-y., Chen J.-y., Kuang J.-f., Shan W., Xie H., Jiang Y.-m., Lu W.-j. (2013). Banana
ethylene response factors
are involved in fruit ripening through their interactions with ethylene
biosynthesis genes. Journal of Experimental
Botany.

[496] Wang Z.-Y., MacRae E. A., Wright M. A., Bolitho K. M., Ross G. S., Atkinson R. G. (2000). Polygalacturonase gene expression
in kiwifruit: relationship to fruit softening and ethylene production. Plant Molecular Biology.

[497] Park J.-I., Lee Y.-K., Chung W.-I., Lee I.-H., Choi J.-H., Lee W.-M., Ezura H., Lee S.-P., Kim I.-J. (2006). Modification of Sugar Composition
in Strawberry Fruit
by Antisense Suppression of an ADP-glucose Pyrophosphorylase. Molecular Breeding.

[498] Xue M., Long Y., Zhao Z., Huang G., Huang K., Zhang T., Jiang Y., Yuan Q., Pei X. (2018). Isolation
and Characterization of a Green-Tissue Promoter from Common Wild Rice
(Oryza rufipogon Griff.). International Journal
of Molecular Sciences.

[499] Miroshnichenko D., Firsov A., Timerbaev V., Kozlov O., Klementyeva A., Shaloiko L., Dolgov S. (2020). Evaluation
of Plant-Derived Promoters for Constitutive and Tissue-Specific Gene
Expression in Potato. Plants.

[500] Jeong H.-J., Jung K.-H. (2015). Rice tissue-specific
promoters and
condition-dependent promoters for effective translational application. Journal of Integrative Plant Biology.

[501] Jung K.-H., Hur J., Ryu C.-H., Choi Y., Chung Y.-Y., Miyao A., Hirochika H., An G. (2003). Characterization of a Rice Chlorophyll-Deficient Mutant Using the
T-DNA Gene-Trap System. Plant and Cell Physiology.

[502] Huang HaiQun, H. H. ; Lin YongJun, L. Y. Cloning and functional analysis of the rice rbcS gene promoter. 2007.

[503] Cai M., Wei J., Li X., Xu C., Wang S. (2007). A rice promoter
containing both novel positive and negative cis-elements for regulation
of green tissue-specific gene expression in transgenic plants. Plant Biotechnology Journal.

[504] Lin M., Yan J., Ali M. M., Wang S., Tian S., Chen F., Lin Z. (2022). Isolation
and Functional Characterization
of a Green-Tissue Promoter in Japonica Rice (Oryza sativa subsp. Japonica). Biology.

[505] Datta K., Vasquez A., Tu J., Torrizo L., Alam M. F., Oliva N., Abrigo E., Khush G. S., Datta S. K. (1998). Constitutive and tissue-specific differential expression
of the cryIA(b) gene in transgenic rice plants conferring resistance
to rice insect pest. Theoretical and Applied
Genetics.

[506] Liu W., Mazarei M., Ye R., Peng Y., Shao Y., Baxter H. L., Sykes R. W., Turner G. B., Davis M. F., Wang Z.-Y. (2018). Switchgrass (Panicum virgatum L.) promoters for green
tissue-specific expression of the MYB4 transcription factor for reduced-recalcitrance
transgenic switchgrass. Biotechnology for Biofuels.

[507] Zaidi M. A., Mohammadi M., Postel S., Masson L., Altosaar I. (2005). The Bt gene cry2Aa2
driven by a tissue specific ST-LS1
promoter from potato effectively controls Heliothis virescens. Transgenic Research.

[508] Vorst O., van Dam F., Oosterhoff-Teertstra R., Smeekens S., Weisbeek P. (1990). Tissue-specific expression directed
by an Arabidopsis thaliana pre-ferredoxin promoter in transgenic tobacco
plants. Plant Molecular Biology.

[509] Nishiuchi T., Kodama H., Yanagisawa S., Iba K. (1999). Wound-Induced Expression of the FAD7Gene Is Mediated by Different
Regulatory Domains of Its Promoter in Leaves/Stems and Roots1. Plant Physiology.

[510] Dedonder A., Rethy R., Fredericq H., Van Montagu M., Krebbers E. (1993). Arabidopsis rbcS Genes Are Differentially
Regulated by Light. Plant Physiology.

[511] Su P., Jin X., Sun T., Chen L., Shi F., Li K., Chang J., Yang G., He G. (2019). Identification and
validation of organ-preferential genes and analysis of corresponding
upstream tissue-specific promoters in wheat. Biologia plantarum.

[512] Outchkourov N. S., Peters J., de Jong J., Rademakers W., Jongsma M. A. (2003). The promoter-terminator of chrysanthemum rbcS1 directs
very high expression levels in plants. Planta.

[513] Marraccini P., Freire L. P., Alves G. S. C., Vieira N. G., Vinecky F., Elbelt S., Ramos H. J. O., Montagnon C., Vieira L. G. E., Leroy T. (2011). RBCS1 expression in
coffee: Coffea orthologs, Coffea arabica homeologs, and expression
variability between genotypes and under drought stress. BMC Plant Biology.

[514] Rahamkulov I., Bakhsh A. (2020). Tissue-specific and stress-inducible
promoters establish their suitability for containment of foreign gene(s)
expression in transgenic potatoes. 3 Biotech.

[515] Orozco B. M., Ogren W. L. (1993). Localization of
light-inducible and
tissue-specific regions of the spinach ribulose bisphosphate carboxylase/oxygenase
(rubisco) activase promoter in transgenic tobacco plants. Plant Molecular Biology.

[516] Kudo T., Makita N., Kojima M., Tokunaga H., Sakakibara H. (2012). Cytokinin Activity of cis-Zeatin
and Phenotypic Alterations
Induced by Overexpression of Putative cis-Zeatin-O-glucosyltransferase
in Rice. Plant Physiology.

[517] Wang R., Zhu M., Ye R., Liu Z., Zhou F., Chen H., Lin Y. (2016). Novel green tissue-specific
synthetic promoters and cis-regulatory elements in rice. Scientific Reports.

[518] Wang R., Zhu M.-L., Gao F.-Y., Ren J.-S., Lu X.-J., Ren G.-J., Lin Y.-J. (2017). Designing, Construction
and Functional Characterization of Tissue-specific Synthetic Promoter
in Rice. Acta Agronomica Sinica.

[519] YI J.-Y., Lee S.-W., SEO H.-W., PARK K.-W. (1998). Tissue
Specific Expression of Tomato Phenylalanine Ammonia-lyase Gene in
Transgenic Tobacco Plants. Korean Journal of
Plant Tissue Culture.

[520] Thilmony R., Guttman M., Thomson J. G., Blechl A. E. (2009). The LP2
leucine-rich repeat receptor kinase gene promoter directs organ-specific,
light-responsive expression in transgenic rice. Plant Biotechnology Journal.

[521] Rasori A., Bertolasi B., Furini A., Bonghi C., Tonutti P., Ramina A. (2003). Functional
analysis of peach ACC
oxidase promoters in transgenic tomato and in ripening peach fruit. Plant Science.

[522] Danila F., Schreiber T., Ermakova M., Hua L., Vlad D., Lo S.-F., Chen Y.-S., Lambret-Frotte J., Hermanns A. S., Athmer B. (2022). A single promoter-TALE
system for tissue-specific and tuneable expression of multiple genes
in rice. Plant Biotechnology Journal.

[523] GIRIN T., LEJAY L., WIRTH J., WIDIEZ T., PALENCHAR P. M., NAZOA P., TOURAINE B., GOJON A., LEPETIT M. (2007). Identification of a 150 bp cis-acting
element of the
AtNRT2.1 promoter involved in the regulation of gene expression by
the N and C status of the plant. Plant, Cell
& Environment.

[524] Kong K., Makabe S., Ntui V. O., Khan R. S., Nakamura I. (2014). Synthetic chitinase gene driven by
root-specific LjNRT2
and AtNRT2.1 promoters confers resistance to Fusarium oxysporum in
transgenic tobacco and tomato. Plant Biotechnology
Reports.

[525] Li P., Wu Y., Han X., Li H., Wang L., Chen B., Yu S., Wang Z. (2023). BrrA02.LMI1
Encodes
a Homeobox Protein That Affects Leaf Margin Development in Brassica
rapa. International Journal of Molecular Sciences.

[526] Taniguchi M., Izawa K., Ku M. S. B., Lin J.-H., Saito H., Ishida Y., Ohta S., Komari T., Matsuoka M., Sugiyama T. (2000). The Promoter for the
Maize C4 Pyruvate,
orthophosphate Dikinase Gene Directs Cell- and Tissue-Specific Transcription
in Transgenic Maize Plants. Plant and Cell Physiology.

[527] Yu Z.-H., Han Y.-N., Xiao X.-G. (2015). A PPO Promoter from
Betalain-Producing Red Swiss Chard, Directs Petiole- and Root-Preferential
Expression of Foreign Gene in Anthocyanins-Producing Plants. International Journal of Molecular Sciences.

[528] Vaughan S. P., James D. J., Lindsey K., Massiah A. J. (2006). Characterization
of FaRB7, a near root-specific gene from strawberry (Fragaria×ananassa
Duch.) and promoter activity analysis in homologous and heterologous
hosts. Journal of Experimental Botany.

[529] Huynh L. N., VanToai T., Streeter J., Banowetz G. (2005). Regulation
of flooding tolerance of SAG12:ipt Arabidopsis plants by cytokinin. Journal of Experimental Botany.

[530] Gan S., Amasino R. M. (1995). Inhibition of Leaf
Senescence by Autoregulated Production
of Cytokinin. Science.

[531] Ori N., Juarez M. T., Jackson D., Yamaguchi J., Banowetz G. M., Hake S. (1999). Leaf Senescence Is
Delayed in Tobacco
Plants Expressing the Maize Homeobox Gene knotted1 under the Control
of a Senescence-Activated Promoter. The Plant
Cell.

[532] Jordi W., Schapendonk A., Davelaar E., Stoopen G. M., Pot C. S., De Visser R., Rhijn J. A. V., Gan S., Amasino R. M. (2000). Increased cytokinin levels in transgenic PSAG12-IPT
tobacco plants have large direct and indirect effects on leaf senescence,
photosynthesis and N partitioning. Plant, Cell
& Environment.

[533] Cowan A. K., Freeman M., Björkman P.-O., Nicander B., Sitbon F., Tillberg E. (2005). Effects of senescence-induced
alteration in cytokinin metabolism on source-sink relationships and
ontogenic and stress-induced transitions in tobacco. Planta.

[534] Fu Y., Ding Y., Liu X., Sun C., Cao S., Wang D., He S., Wang X., Li L., Tian W. (1998). Rice transformation with a senescence-inhibition chimeric gene. Chinese Science Bulletin.

[535] McCabe M. S., Garratt L. C., Schepers F., Jordi W. J. R. M., Stoopen G. M., Davelaar E., van Rhijn J. H. A., Power J. B., Davey M. R. (2001). Effects of PSAG12-IPT Gene Expression
on Development and Senescence in Transgenic Lettuce. Plant Physiology.

[536] Chang H., Jones M. L., Banowetz G. M., Clark D. G. (2003). Overproduction
of Cytokinins in Petunia Flowers Transformed with PSAG12-IPT Delays
Corolla Senescence and Decreases Sensitivity to Ethylene. Plant Physiology.

[537] Swartzberg D., Kirshner B., Rav-David D., Elad Y., Granot D. (2008). Botrytis cinerea induces senescence
and is inhibited by autoregulated expression of the IPT gene. European Journal of Plant Pathology.

[538] Swartzberg D., Hanael R., Granot D. (2011). Relationship
between
hexokinase and cytokinin in the regulation of leaf senescence and
seed germination. Plant Biology.

[539] Sýkorová B., Kurešová G., Daskalova S., Trčková M., Hoyerová K., Raimanová I., Motyka V., Trávníčková A., Elliott M. C., Kamínek M. (2008). Senescence-induced ectopic expression
of the A. tumefaciens ipt gene in wheat delays leaf senescence, increases
cytokinin content, nitrate influx, and nitrate reductase activity,
but does not affect grain yield. Journal of
Experimental Botany.

[540] Xu Y., Gianfagna T., Huang B. (2010). Proteomic changes associated with
expression of a gene (ipt) controlling cytokinin synthesis for improving
heat tolerance in a perennial grass species. Journal of Experimental Botany.

[541] Merewitz E. B., Gianfagna T., Huang B. (2011). Photosynthesis, water
use, and root viability under water stress as affected by expression
of SAG12-ipt controlling cytokinin synthesis in Agrostis stolonifera. Journal of Experimental Botany.

[542] Zhang P., Wang W.-Q., Zhang G.-L., Kaminek M., Dobrev P., Xu J., Gruissem W. (2010). Senescence-Inducible
Expression of Isopentenyl Transferase Extends Leaf Life, Increases
Drought Stress Resistance and Alters Cytokinin Metabolism in Cassava. Journal of Integrative Plant Biology.

[543] Lai Q.-x., Bao Z.-y., Zhu Z.-j., Qian Q.-q., Mao B.-z. (2007). Effects of osmotic stress on antioxidant
enzymes activities
in leaf discs of PSAG12-IPT modified gerbera. Journal of Zhejiang University SCIENCE B.

[544] Noh Y.-S., Amasino R. M. (1999). Regulation of developmental
senescence
is conserved between Arabidopsis and Brassica napus. Plant Molecular Biology.

[545] Li Q., Robson P. R. H., Bettany A. J. E., Donnison I. S., Thomas H., Scott I. M. (2004). Modification of
senescence in ryegrass transformed
with IPT under the control of a monocot senescence-enhanced promoter. Plant Cell Reports.

[546] Robson P. R. H., Donnison I. S., Wang K., Frame B., Pegg S. E., Thomas A., Thomas H. (2004). Leaf senescence
is
delayed in maize expressing the Agrobacterium IPT gene under the control
of a novel maize senescence-enhanced promoter. Plant Biotechnology Journal.

[547] Sato Y., Morita R., Nishimura M., Yamaguchi H., Kusaba M. (2007). Mendel's green cotyledon gene
encodes
a positive regulator of the chlorophyll-degrading pathway. Proceedings of the National Academy of Sciences.

[548] Furukawa K., Ichikawa S., Nigorikawa M., Sonoki T., Ito Y. (2014). Enhanced production
of reducing sugars
from transgenic rice expressing exo-glucanase under the control of
a senescence-inducible promoter. Transgenic
Research.

[549] Peleg Z., Reguera M., Tumimbang E., Walia H., Blumwald E. (2011). Cytokinin-mediated source/sink modifications
improve drought tolerance and increase grain yield in rice under water-stress. Plant Biotechnology Journal.

[550] Rivero R. M., Kojima M., Gepstein A., Sakakibara H., Mittler R., Gepstein S., Blumwald E. (2007). Delayed leaf
senescence
induces extreme drought tolerance in a flowering plant. Proceedings of the National Academy of Sciences.

[551] Qin H., Gu Q., Zhang J., Sun L., Kuppu S., Zhang Y., Burow M., Payton P., Blumwald E., Zhang H. (2011). Regulated
Expression of an Isopentenyltransferase Gene (IPT) in Peanut
Significantly Improves Drought Tolerance and Increases Yield Under
Field Conditions. Plant and Cell Physiology.

[552] Kuppu S., Mishra N., Hu R., Sun L., Zhu X., Shen G., Blumwald E., Payton P., Zhang H. (2013). Water-Deficit
Inducible Expression of a Cytokinin Biosynthetic Gene IPT Improves
Drought Tolerance in Cotton. PLOS ONE.

[553] Décima Oneto C., Otegui M. E., Baroli I., Beznec A., Faccio P., Bossio E., Blumwald E., Lewi D. (2016). Water deficit
stress tolerance in maize conferred by expression of an isopentenyltransferase
(IPT) gene driven by a stress- and maturation-induced promoter. Journal of Biotechnology.

[554] Truernit E., Sauer N. (1995). The promoter of the
Arabidopsis thaliana
SUC2 sucrose-H+ symporter gene directs expression of β-glucuronidase
to the phloem: Evidence for phloem loading and unloading by SUC2. Planta.

[555] Dutt M., Ananthakrishnan G., Jaromin M. K., Brlansky R. H., Grosser J. W. (2012). Evaluation
of four phloem-specific promoters in vegetative
tissues of transgenic citrus plants. Tree Physiology.

[556] Medberry S. L., Lockhart B. E., Olszewski N. E. (1992). The Commelina
yellow mottle virus promoter is a strong promoter in vascular and
reproductive tissues. The Plant Cell.

[557] Koramutla, M. K. ; Bhatt, D. ; Negi, M. ; Venkatachalam, P. ; Jain, P. K. ; Bhattacharya, R. Strength, Stability, and cis-Motifs of In silico Identified Phloem-Specific Promoters in Brassica juncea (L.). Frontiers in Plant Science 2016, 7, 10.3389/fpls.2016.00457. PMC4834444 27148290

[558] Yin Y., Chen L., Beachy R. (1997). Promoter elements
required for phloem-specific
gene expression from the RTBV promoter in rice. The Plant Journal.

[559] Schmülling T., Schell J., Spena A. (1989). Promoters
of the rolA,
B, and C genes of Agrobacterium rhizogenesare differentially regulated
in transgenic plants. The Plant Cell.

[560] Wang M.-B., Boulter D., Gatehouse J. A. (1992). A complete
sequence of the rice sucrose synthase-1 (RSs1) gene. Plant Molecular Biology.

[561] Puente P., Wei N., Deng X. W. (1996). Combinatorial
interplay
of promoter elements constitutes the minimal determinants for light
and developmental control of gene expression in Arabidopsis. The EMBO Journal.

[562] Oo M. M., Bae H.-K., Nguyen T. D., Moon S., Oh S. A., Kim J. H., Soh M.-S., Song J. T., Jung K.-H., Park S. K. (2014). Evaluation of rice
promoters conferring
pollen-specific expression in a heterologous system, Arabidopsis. Plant Reproduction.

[563] Gupta V., Khurana R., Tyagi A. K. (2007). Promoters
of two
anther-specific genes confer organ-specific gene expression in a stage-specific
manner in transgenic systems. Plant Cell Reports.

[564] Rao G. S., Deveshwar P., Sharma M., Kapoor S., Rao K. V. (2018). Evolvement
of transgenic male-sterility and fertility-restoration
system in rice for production of hybrid varieties. Plant Molecular Biology.

[565] Hamilton D. A., Schwarz Y. H., Mascarenhas J. P. (1998). A monocot
pollen-specific promoter contains separable pollen-specific and quantitative
elements. Plant Molecular Biology.

[566] Singh M., Bhalla P. L., Xu H., Singh M. B. (2003). Isolation
and characterization of a flowering plant male gametic cell-specific
promoter 1. FEBS Letters.

[567] van Tunen A. J., Mur L. A., Brouns G. S., Rienstra J. D., Koes R. E., Mol J. N. (1990). Pollen- and anther-specific
chi promoters
from petunia: tandem promoter regulation of the chiA gene. The Plant Cell.

[568] Zimmermann P., Zardi G., Lehmann M., Zeder C., Amrhein N., Frossard E., Bucher M. (2003). Engineering
the root-soil
interface via targeted expression of a synthetic phytase gene in trichoblasts. Plant Biotechnology Journal.

[569] Liu Y., Cui S., Wu F., Yan S., Lin X., Du X., Chong K., Schilling S., Theißen G., Meng Z. (2013). Functional Conservation of MIKC*-Type
MADS Box Genes in Arabidopsis
and Rice Pollen Maturation. The Plant Cell.

[570] Han M.-J., Jung K.-H., Yi G., Lee D.-Y., An G. (2006). Rice Immature
Pollen 1 (RIP1) is a Regulator of Late Pollen Development. Plant and Cell Physiology.

[571] Peng, J. ; Qi, X. ; Chen, X. ; Li, N. ; Yu, J. ZmDof30 Negatively Regulates the Promoter Activity of the Pollen-Specific Gene Zm908. Frontiers in Plant Science 2017, 8, 10.3389/fpls.2017.00685. PMC5410603 28507558

[572] Wakeley P. R., Rogers H. J., Rozycka M., Greenland A. J., Hussey P. J. (1998). A maize pectin methylesterase-like gene, ZmC5, specifically
expressed in pollen. Plant Molecular Biology.

[573] Rogers H. J., Bate N., Combe J., Sullivan J., Sweetman J., Swan C., Lonsdale D. M., Twell D. (2001). Functional
analysis of cis-regulatory elements within the promoter of the tobacco
late pollen gene g10. Plant Molecular Biology.

[574] Albani D., Robert L. S., Donaldson P. A., Altosaar I., Arnison P. G., Fabijanski S. F. (1990). Characterization
of a pollen-specific gene family from Brassica napus which is activated
during early microspore development. Plant Molecular
Biology.

[575] Jiang S.-Y., Vanitha J., Bai Y., Ramachandran S. (2014). Identification
and molecular characterization of tissue-preferred rice genes and
their upstream regularly sequences on a genome-wide level. BMC Plant Biology.

[576] Gómez M. D., Beltrán J.-P., Cañas L. A. (2004). The pea
END1 promoter drives anther-specific gene expression in different
plant species. Planta.

[577] Shahan, R. ; Li, D. ; Liu, Z. Identification of genes preferentially expressed in wild strawberry receptacle fruit and demonstration of their promoter activities. Horticulture Research 2019, 6, 10.1038/s41438-019-0134-6. PMC6491448 31044078

[578] Yamamoto Y. T., Taylor C. G., Acedo G. N., Cheng C. L., Conkling M. A. (1991). Characterization
of cis-acting sequences regulating
root-specific gene expression in tobacco. The
Plant Cell.

[579] Barone P., Rosellini D., LaFayette P., Bouton J., Veronesi F., Parrott W. (2008). Bacterial citrate synthase
expression and soil aluminum tolerance in transgenic alfalfa. Plant Cell Reports.

[580] Chan Y.-L., Prasad V., Sanjaya, Chen K. H., Liu P. C., Chan M.-T., Cheng C.-P. (2005). Transgenic tomato plants expressing
an Arabidopsis thionin (Thi2.1) driven by fruit-inactive promoter
battle against phytopathogenic attack. Planta.

[581] Radi A., Dina P., Guy A. (2006). Expression of sarcotoxin
IA gene via a root-specific tob promoter enhanced host resistance
against parasitic weeds in tomato plants. Plant
Cell Reports.

[582] Chen L., Jiang B., Wu C., Sun S., Hou W., Han T. (2014). GmPRP2 promoter drives root-preferential expression
in transgenic Arabidopsis and soybean hairy roots. BMC Plant Biology.

[583] Xun H., Zhang X., Yu J., Pang J., Wang S., Liu B., Dong Y., Jiang L., Guo D. (2021). Analysis of expression
characteristics of soybean leaf and root tissue-specific promoters
in Arabidopsis and soybean. Transgenic Research.

[584] Schünmann P. H. D., Richardson A. E., Vickers C. E., Delhaize E. (2004). Promoter Analysis
of the Barley Pht1;1
Phosphate Transporter Gene Identifies Regions Controlling Root Expression
and Responsiveness to Phosphate Deprivation. Plant Physiology.

[585] Nitz I., Berkefeld H., Puzio P. S., Grundler F. M. W. (2001). Pyk10,
a seedling and root specific gene and promoter from Arabidopsis thaliana. Plant Science.

[586] Suzuki K.-i., Yamada Y., Hashimoto T. (1999). Expression
of Atropa belladonna Putrescine N-Methyltransferase Gene in Root Pericycle. Plant and Cell Physiology.

[587] Suzuki H., Fowler T. J., Tierney M. L. (1993). Deletion
analysis
and localization of SbPRP1, a soybean cell wall protein gene, in roots
of transgenic tobacco and cowpea1. Plant Molecular
Biology.

[588] Feuillet C., Lauvergeat V., Deswarte C., Pilate G., Boudet A., Grima-Pettenati J. (1995). Tissue- and cell-specific expression
of a cinnamyl alcohol dehydrogenase promoter in transgenic poplar
plants. Plant Molecular Biology.

[589] Kaur C., Mustafiz A., Sarkar A. K., Ariyadasa T. U., Singla-Pareek S. L., Sopory S. K. (2014). Expression of abiotic
stress inducible
ETHE1-like protein from rice is higher in roots and is regulated by
calcium. Physiologia Plantarum.

[590] Li Y., Liu S., Yu Z., Liu Y., Wu P. (2013). Isolation
and characterization of two novel root-specific promoters in rice
(Oryza sativa L.). Plant Science.

[591] Sasaki A., Yamaji N., Yokosho K., Ma J. F. (2012). Nramp5
Is a Major Transporter Responsible for Manganese and Cadmium Uptake
in Rice. The Plant Cell.

[592] Ueno D., Yamaji N., Kono I., Huang C. F., Ando T., Yano M., Ma J. F. (2010). Gene limiting
cadmium
accumulation in rice. Proceedings of the National
Academy of Sciences.

[593] Ben Saad R., Ben Romdhane W., Zouari N., Ben Hsouna A., Harbaoui M., Brini F., Ghneim-Herrera T. (2020). Characterization
of a novel LmSAP gene promoter from Lobularia maritima: Tissue specificity
and environmental stress responsiveness. PLOS
ONE.

[594] Xu Y., Buchholz W. G., DeRose R. T., Hall T. C. (1995). Characterization
of a rice gene family encoding root-specific proteins. Plant Molecular Biology.

[595] Gao S., Fang J., Xu F., Wang W., Sun X., Chu J., Cai B., Feng Y., Chu C. (2014). CYTOKININ OXIDASE/DEHYDROGENASE4
Integrates Cytokinin and Auxin Signaling to Control Rice Crown Root
Formation. Plant Physiology.

[596] Park S.-H., Jeong J. S., Han E. H., Redillas M. C. F. R., Bang S. W., Jung H., Kim Y. S., Kim J.-K. (2013). Characterization
of the root-predominant gene promoter HPX1 in transgenic rice plants. Plant Biotechnology Reports.

[597] Manavella P. A., Dezar C. A., Bonaventure G., Baldwin I. T., Chan R. L. (2008). HAHB4, a sunflower HD-Zip protein,
integrates signals from the jasmonic acid and ethylene pathways during
wounding and biotic stress responses. The Plant
Journal.

[598] Liu J.-J., Ekramoddoullah A. K.
M. (2003). Root-specific expression
of a western white pine PR10 gene is mediated by different promoter
regions in transgenic tobacco. Plant Molecular
Biology.

[599] Xu X., Guo S., Chen K., Song H., Liu J., Guo L., Qian Q., Wang H. (2010). A 796 bp PsPR10 gene promoter fragment
increased root-specific expression of the GUS reporter gene under
the abiotic stresses and signal molecules in tobacco. Biotechnology Letters.

[600] Leach F., Aoyagi K. (1991). Promoter analysis of
the highly expressed
rolC and rolD root-inducing genes of Agrobacterium rhizogenes: enhancer
and tissue-specific DNA determinants are dissociated. Plant Science.

[601] Mohan C., Jayanarayanan A. N., Narayanan S. (2017). Construction
of a novel synthetic root-specific promoter and its characterization
in transgenic tobacco plants. 3 Biotech.

[602] Marquès-Bueno M. M., Morao A. K., Cayrel A., Platre M. P., Barberon M., Caillieux E., Colot V., Jaillais Y., Roudier F., Vert G. (2016). A versatile
Multisite Gateway-compatible promoter and transgenic line collection
for cell type-specific functional genomics in Arabidopsis. The Plant Journal.

[603] Szymanski D. B., Jilk R. A., Pollock S. M., Marks M. D. (1998). Control
of GL2 expression in Arabidopsis leaves and trichomes. Development.

[604] Masucci J. D., Rerie W. G., Foreman D. R., Zhang M., Galway M. E., Marks M. D., Schiefelbein J. W. (1996). The homeobox
gene GLABRA 2 is required for position-dependent cell differentiation
in the root epidermis of Arabidopsis thaliana. Development.

[605] Mustroph A., Zanetti M. E., Jang C. J. H., Holtan H. E., Repetti P. P., Galbraith D. W., Girke T., Bailey-Serres J. (2009). Profiling
translatomes of discrete cell populations resolves altered cellular
priorities during hypoxia in *Arabidopsis*. Proceedings of the National Academy of Sciences.

[606] Puig J., Meynard D., Khong G. N., Pauluzzi G., Guiderdoni E., Gantet P. (2013). Analysis of the expression of the
AGL17-like clade of MADS-box transcription factors in rice. Gene Expression Patterns.

[607] Willemsen V., Bauch M., Bennett T., Campilho A., Wolkenfelt H., Xu J., Haseloff J., Scheres B. (2008). The NAC Domain
Transcription Factors FEZ and SOMBRERO Control the Orientation of
Cell Division Plane in Arabidopsis Root Stem Cells. Developmental Cell.

[608] Friml J., Vieten A., Sauer M., Weijers D., Schwarz H., Hamann T., Offringa R., Jürgens G. (2003). Efflux-dependent
auxin gradients establish the apical-basal axis of Arabidopsis. Nature.

[609] Friml J., Benková E., Blilou I., Wisniewska J., Hamann T., Ljung K., Woody S., Sandberg G., Scheres B., Jürgens G. (2002). AtPIN4 Mediates Sink-Driven
Auxin Gradients and Root Patterning in Arabidopsis. Cell.

[610] Baima S., Nobili F., Sessa G., Lucchetti S., Ruberti I., Morelli G. (1995). The expression of the Athb-8 homeobox
gene is restricted to provascular cells in Arabidopsis thaliana. Development.

[611] Savaldi-Goldstein S., Peto C., Chory J. (2007). The epidermis
both
drives and restricts plant shoot growth. Nature.

[612] Sarkar A. K., Luijten M., Miyashima S., Lenhard M., Hashimoto T., Nakajima K., Scheres B., Heidstra R., Laux T. (2007). Conserved factors regulate signalling
in Arabidopsis thaliana shoot and root stem cell organizers. Nature.

[613] Lee J.-Y., Colinas J., Wang J. Y., Mace D., Ohler U., Benfey P. N. (2006). Transcriptional
and posttranscriptional
regulation of transcription factor expression in *Arabidopsis* roots. Proceedings of the National Academy
of Sciences.

[614] Heidstra R., Welch D., Scheres B. (2004). Mosaic analyses using
marked activation and deletion clones dissect Arabidopsis SCARECROW
action in asymmetric cell division. Genes &
Development.

[615] Sieberer T., Seifert G. J., Hauser M.-T., Grisafi P., Fink G. R., Luschnig C. (2000). Post-transcriptional control of the *Arabidopsis* auxin efflux carrier EIR1 requires AXR1. Current
Biology.

[616] Jones M. O., Manning K., Andrews J., Wright C., Taylor I. B., Thompson A. J. (2008). The promoter from SlREO, a highly-expressed,
root-specific Solanum lycopersicum gene, directs expression to cortex
of mature roots. Functional Plant Biology.

[617] Wysocka-Diller J. W., Helariutta Y., Fukaki H., Malamy J. E., Benfey P. N. (2000). Molecular analysis
of SCARECROW function reveals a
radial patterning mechanism common to root and shoot. Development.

[618] Ai P., Sun S., Zhao J., Fan X., Xin W., Guo Q., Yu L., Shen Q., Wu P., Miller A. J. (2009). Two rice phosphate transporters, OsPht1;2 and OsPht1;6, have different
functions and kinetic properties in uptake and translocation. The Plant Journal.

[619] Lee M. M., Schiefelbein J. (1999). WEREWOLF,
a MYB-Related Protein in
Arabidopsis, Is a Position-Dependent Regulator of Epidermal Cell Patterning. Cell.

[620] Shrawat A. K., Carroll R. T., DePauw M., Taylor G. J., Good A. G. (2008). Genetic engineering of improved nitrogen use efficiency
in rice by the tissue-specific expression of alanine aminotransferase. Plant Biotechnology Journal.

[621] Kakrana, A. ; Kumar, A. ; Satheesh, V. ; Abdin, M. Z. ; Subramaniam, K. ; Bhattacharya, R. C. ; Srinivasan, R. ; Sirohi, A. ; Jain, P. K. Identification, Validation and Utilization of Novel Nematode-Responsive Root-Specific Promoters in Arabidopsis for Inducing Host-Delivered RNAi Mediated Root-Knot Nematode Resistance. Frontiers in Plant Science 2017, 8, 10.3389/fpls.2017.02049. PMC5733009 29312363

[622] ZhiMing Y., Bo K., XiaoWei H., ShaoLei L., YouHuang B., WoNa D., Ming C., Hyung-Taeg C., Ping W. (2011). Root hair-specific
expansins modulate root hair elongation in rice. The Plant Journal.

[623] Kim C. M., Park S. H., Je B. I., Park S. H., Park S. J., Piao H. L., Eun M. Y., Dolan L., Han C.-d. (2007). OsCSLD1, a Cellulose Synthase-Like
D1 Gene, Is Required
for Root Hair Morphogenesis in Rice. Plant Physiology.

[624] Tsukagoshi H., Busch W., Benfey P. N. (2010). Transcriptional
Regulation of ROS Controls Transition from Proliferation to Differentiation
in the Root. Cell.

[625] Moreno-Risueno M. A., Van Norman J. M., Moreno A., Zhang J., Ahnert S. E., Benfey P. N. (2010). Oscillating
Gene Expression Determines
Competence for Periodic *Arabidopsis* Root Branching. Science.

[626] De Rybel B., Vassileva V., Parizot B., Demeulenaere M., Grunewald W., Audenaert D., Van Campenhout J., Overvoorde P., Jansen L., Vanneste S. (2010). A Novel
Aux/IAA28 Signaling Cascade Activates GATA23-Dependent Specification
of Lateral Root Founder Cell Identity. Current
Biology.

[627] Ma J. F., Tamai K., Yamaji N., Mitani N., Konishi S., Katsuhara M., Ishiguro M., Murata Y., Yano M. (2006). A silicon transporter in rice. Nature.

[628] Carsolio C., Campos F., Sánchez F., Rocha-Sosa M. (1994). The expression
of a chimeric Phaseolus vulgaris nodulin
30-GUS gene is restricted to the rhizobially infected cells in transgenic
Lotus corniculatus nodules. Plant Molecular
Biology.

[629] Frühling M., Schröder G., Hohnjec N., Pühler A., Perlick A. M., Küster H. (2000). The promoter of the Vicia faba L.
gene VfEnod12 encoding an early nodulin is active in cortical cells
and nodule primordia of transgenic hairy roots of Vicia hirsuta as
well as in the prefixing zone II of mature transgenic V. hirsuta root
nodules. Plant Science.

[630] Hansen A. C., Busk H., Marcker A., Marcker K. A., Jensen E. Ø. (1999). VsENBP1 regulates the expression
of the early nodulin
PsENOD12B. Plant Molecular Biology.

[631] Szabados L., Ratet P., Grunenberg B., de Bruijn F. J. (1990). Functional analysis of the Sesbania rostrata leghemoglobin
glb3 gene 5-upstream region in transgenic Lotus corniculatus and Nicotiana
tabacum plants. The Plant Cell.

[632] Bonke M., Thitamadee S., Mähönen A. P., Hauser M.-T., Helariutta Y. (2003). APL regulates
vascular tissue identity
in Arabidopsis. Nature.

[633] Imlau A., Truernit E., Sauer N. (1999). Cell-to-Cell
and Long-Distance
Trafficking of the Green Fluorescent Protein in the Phloem and Symplastic
Unloading of the Protein into Sink Tissues. The Plant Cell.

[634] Sahoo D. K., Sarkar S., Raha S., Maiti I. B., Dey N. (2014). Comparative analysis of synthetic DNA promoters for high-level gene
expression in plants. Planta.

[635] Helariutta Y., Fukaki H., Wysocka-Diller J., Nakajima K., Jung J., Sena G., Hauser M.-T., Benfey P. N. (2000). The SHORT-ROOT Gene Controls Radial Patterning of the
Arabidopsis Root through Radial Signaling. Cell.

[636] Kamiya N., Nagasaki H., Morikami A., Sato Y., Matsuoka M. (2003). Isolation
and characterization of a rice WUSCHEL-type
homeobox gene that is specifically expressed in the central cells
of a quiescent center in the root apical meristem. The Plant Journal.

[637] Müller I., Wagner W., Völker A., Schellmann S., Nacry P., Küttner F., Schwarz-Sommer Z., Mayer U., Jürgens G. (2003). Syntaxin specificity
of cytokinesis in Arabidopsis. Nature Cell Biology.

[638] Men S., Boutté Y., Ikeda Y., Li X., Palme K., Stierhof Y.-D., Hartmann M.-A., Moritz T., Grebe M. (2008). Sterol-dependent endocytosis
mediates post-cytokinetic acquisition
of PIN2 auxin efflux carrier polarity. Nature
Cell Biology.

[639] Onyango S. O., Roderick H., Tripathi J. N., Collins R., Atkinson H. J., Oduor R. O., Tripathi L. (2016). The ZmRCP-1 promoter
of maize provides root tip specific expression of transgenes in plantain. Journal of Biological Research-Thessaloniki.

[640] Zhao Y., Hu Y., Dai M., Huang L., Zhou D.-X. (2009). The WUSCHEL-Related Homeobox Gene
WOX11 Is Required
to Activate Shoot-Borne Crown Root Development in Rice. The Plant Cell.

[641] Cho H.-T., Cosgrove D. J. (2002). Regulation of Root
Hair Initiation
and Expansin Gene Expression in Arabidopsis[W]. The Plant Cell.

[642] Lee Y., Bak G., Choi Y., Chuang W.-I., Cho H.-T., Lee Y. (2008). Roles of Phosphatidylinositol
3-Kinase in Root Hair Growth. Plant Physiology.

[643] Bernhardt C., Tierney M. L. (2000). Expression of AtPRP3, a Proline-Rich
Structural Cell Wall Protein from Arabidopsis, Is Regulated by Cell-Type-Specific
Developmental Pathways Involved in Root Hair Formation1. Plant Physiology.

[644] Xu J., Scheres B. (2005). Dissection of Arabidopsis
ADP-RIBOSYLATION FACTOR 1
Function in Epidermal Cell Polarity. The Plant
Cell.

[645] Vert G. g., Grotz N., Dédaldéchamp F., Gaymard F. d. r., Guerinot M. L., Briat J.-F. o., Curie C. (2002). IRT1, an Arabidopsis
Transporter Essential for Iron Uptake from the Soil and for Plant
Growth. The Plant Cell.

[646] Koehorst-van Putten H.
J. J., Wolters A.-M. A., Pereira-Bertram I. M., van den Berg H. H. J., van der Krol A. R., Visser R. G. F. (2012). Cloning and characterization
of a tuberous root-specific promoter from cassava (Manihot esculenta
Crantz). Planta.

[647] Salehuzzaman S. N. I.
M., Jacobsen E., Visser R. G. F. (1993). Isolation
and characterization of a cDNA encoding granule-bound starch synthase
in cassava (Manihot esculenta Crantz) and its antisense expression
in potato. Plant Molecular Biology.

[648] Song Z., Mietkiewska E., Weselake R. J. (2017). The linin promoter
is highly effective in enhancing punicic acid production in Arabidopsis. Plant Cell Reports.

[649] Naoumkina M., Dixon R. A. (2011). Characterization
of the mannan synthase
promoter from guar (Cyamopsis tetragonoloba). Plant Cell Reports.

[650] Sunilkumar G., Connell J. P., Smith C. W., Reddy A. S., Rathore K. S. (2002). Cotton α-Globulin Promoter:
Isolation and Functional
Characterization in Transgenic Cotton, Arabidopsis, and Tobacco. Transgenic Research.

[651] Sunilkumar G., Campbell L. M., Puckhaber L., Stipanovic R. D., Rathore K. S. (2006). Engineering cottonseed for use in
human nutrition by tissue-specific reduction of toxic gossypol. Proceedings of the National Academy of Sciences.

[652] Sunkara S., Bhatnagar-Mathur P., Sharma K. K. (2014). Isolation and Functional
Characterization of a Novel Seed-Specific Promoter Region from Peanut. Applied Biochemistry and Biotechnology.

[653] Zavallo D., Lopez Bilbao M., Hopp H. E., Heinz R. (2010). Isolation
and functional characterization of two novel seed-specific promoters
from sunflower (Helianthus annuus L.). Plant
Cell Reports.

[654] O'Keefe B. R., Murad A. M., Vianna G. R., Ramessar K., Saucedo C. J., Wilson J., Buckheit K. W., da Cunha N. B., Araújo A. C. G., Lacorte C. C. (2015). Engineering
soya bean
seeds as a scalable platform to produce cyanovirin-N, a non-ARV microbicide
against HIV. Plant Biotechnology Journal.

[655] Bustos M.
M., Guiltinan M. J., Jordano J., Begum D., Kalkan F. A., Hall T. C. (1989). Regulation
of beta-glucuronidase
expression in transgenic tobacco plants by an A/T-rich, cis-acting
sequence found upstream of a French bean beta-phaseolin gene. The Plant Cell.

[656] de Pater S., Pham K., Klitsie I., Kijne J. (1996). The 22 bp
W1 element in the pea lectin promoter is necessary and, as a multimer,
sufficient for high gene expression in tobacco seeds. Plant Molecular Biology.

[657] Kluth A., Sprunck S., Becker D., Lörz H., Lütticke S. (2002). 5′ deletion of a gbss1 promoter
region from
wheat leads to changes in tissue and developmental specificities. Plant Molecular Biology.

[658] Nesi N., Lucas M.-O., Auger B., Baron C., Lécureuil A., Guerche P., Kronenberger J., Lepiniec L., Debeaujon I., Renard M. (2009). The promoter of the
Arabidopsis thalianaBAN gene is active in proanthocyanidin-accumulating
cells of the Brassica napus seed coat. Plant
Cell Reports.

[659] Buchner P., Rochat C., Wuillème S., Boutin J.-P. (2002). Characterization of a tissue-specific and developmentally
regulated β-1,3-glucanase gene in pea (Pisum sativum). Plant Molecular Biology.

[660] Colombo L., Franken J., Van der
Krol A. R., Wittich P. E., Dons H. J., Angenent G. C. (1997). Downregulation
of
ovule-specific MADS box genes from petunia results in maternally controlled
defects in seed development. The Plant Cell.

[661] Kuwano M., Masumura T., Yoshida K. T. (2011). A novel endosperm
transfer cell-containing region-specific gene and its promoter in
rice. Plant Molecular Biology.

[662] Kim J. H., Jung I. J., Kim D. Y., Fanata W. I., Son B. H., Yoo J. Y., Harmoko R., Ko K. S., Moon J. C., Jang H. H. (2011). Proteomic
identification
of an embryo-specific 1Cys-Prx promoter and analysis of its activity
in transgenic rice. Biochemical and Biophysical
Research Communications.

[663] Furtado A., Henry R. J. (2005). The wheat Em promoter
drives reporter
gene expression in embryo and aleurone tissue of transgenic barley
and rice. Plant Biotechnology Journal.

[664] Furtado A., Henry R. J., Pellegrineschi A. (2009). Analysis of
promoters in transgenic barley and wheat. Plant
Biotechnology Journal.

[665] Qu L. Q., Takaiwa F. (2004). Evaluation of tissue
specificity
and expression strength of rice seed component gene promoters in transgenic
rice. Plant Biotechnology Journal.

[666] Kuwano M., Mimura T., Takaiwa F., Yoshida K. T. (2009). Generation
of stable ‘low phytic acid’ transgenic rice through
antisense repression of the 1d-myo-inositol 3-phosphate synthase gene
(RINO1) using the 18-kDa oleosin promoter. Plant
Biotechnology Journal.

[667] Ezcurra I., Ellerström M., Wycliffe P., Stålberg K., Rask L. (1999). Interaction between
composite elements in the napA promoter: both
the B-box ABA-responsive complex and the RY/G complex are necessary
for seed-specific expression. Plant Molecular
Biology.

[668] Ellerström M., Stålberg K., Ezcurra I., Rask L. (1996). Functional
dissection of a napin gene promoter: identification of promoter elements
required for embryo and endosperm-specific transcription. Plant Molecular Biology.

[669] Liu X., Li S., Yang W., Mu B., Jiao Y., Zhou X., Zhang C., Fan Y., Chen R. (2018). Synthesis
of Seed-Specific Bidirectional Promoters for Metabolic Engineering
of Anthocyanin-Rich Maize. Plant and Cell Physiology.

[670] Chopin F., Orsel M., Dorbe M.-F., Chardon F., Truong H.-N., Miller A. J., Krapp A., Daniel-Vedele F. o. (2007). The Arabidopsis
ATNRT2.7 Nitrate Transporter Controls Nitrate Content in Seeds. The Plant Cell.

[671] Xi D.-M., Liu W.-S., Yang G.-D., Wu C.-A., Zheng C.-C. (2010). Seed-specific overexpression of antioxidant
genes in
Arabidopsis enhances oxidative stress tolerance during germination
and early seedling growth. Plant Biotechnology
Journal.

[672] Kovalchuk N., Smith J., Pallotta M., Singh R., Ismagul A., Eliby S., Bazanova N., Milligan A. S., Hrmova M., Langridge P. (2009). Characterization of
the wheat endosperm transfer cell-specific protein TaPR60. Plant Molecular Biology.

[673] Kovalchuk N., Smith J., Bazanova N., Pyvovarenko T., Singh R., Shirley N., Ismagul A., Johnson A., Milligan A. S., Hrmova M. (2012). Characterization
of
the wheat gene encoding a grain-specific lipid transfer protein TdPR61,
and promoter activity in wheat, barley and rice. Journal of Experimental Botany.

[674] Tamás C., Kisgyörgy B. N., Rakszegi M., Wilkinson M. D., Yang M.-S., Láng L., Tamás L., Bedő Z. (2009). Transgenic approach to improve wheat
(Triticum aestivum
L.) nutritional quality. Plant Cell Reports.

[675] Oszvald M., Gardonyi M., Tamas C., Takacs I., Jenes B., Tamas L. (2008). Development and characterization
of a chimaeric tissue-specific promoter in wheat and rice endosperm. In Vitro Cellular & Developmental Biology - Plant.

[676] Russell D. A., Fromm M. E. (1997). Tissue-specific expression in transgenic
maize of four endosperm promoters from maize and rice. Transgenic Research.

[677] Shure M., Wessler S., Fedoroff N. (1983). Molecular
identification
and isolation of the Waxy locus in maize. Cell.

[678] Zheng Z., Kawagoe Y., Xiao S., Li Z., Okita T., Hau T. L., Lin A., Murai N. (1993). 5′
distal and proximal cis-acting regulator elements are required for
developmental control of a rice seed storage protein glutelin gene. The Plant Journal.

[679] Vignesh M., Nepolean T., Hossain F., Singh A. K., Gupta H. S. (2013). Sequence variation in 3′UTR
region of crtRB1
gene and its effect on β-carotene accumulation in maize kernel. Journal of Plant Biochemistry and Biotechnology.

[680] Qu L. Q., Xing Y. P., Liu W. X., Xu X. P., Song Y. R. (2008). Expression pattern and activity of
six glutelin gene
promoters in transgenic rice*. Journal of Experimental
Botany.

[681] Li Q.-F., Sun S. S.-M., Liu Q.-Q. (2013). Characterization
of the spatial and temporal expression of the OsSSII-3 gene encoding
a key soluble starch synthase in rice. Journal
of the Science of Food and Agriculture.

[682] Rasmussen T. B., Donaldson I. A. (2006). Investigation
of the endosperm-specific
sucrose synthase promoter from rice using transient expression of
reporter genes in guar seed tissue. Plant Cell
Reports.

[683] Li Q.-F., Zhang G.-Y., Dong Z.-W., Yu H.-X., Gu M.-H., Sun S. S. M., Liu Q.-Q. (2009). Characterization
of expression of the OsPUL gene encoding a pullulanase-type debranching
enzyme during seed development and germination in rice. Plant Physiology and Biochemistry.

[684] Thomas M. S., Flavell R. B. (1990). Identification of
an enhancer element
for the endosperm-specific expression of high molecular weight glutenin. The Plant Cell.

[685] Schernthaner J. P., Matzke M. A., Matzke A. J. M. (1988). Endosperm-specific
activity of a zein gene promoter in transgenic tobacco plants. The EMBO Journal.

[686] Tosi P., D’Ovidio R., Napier J. A., Bekes F., Shewry P. R. (2004). Expression of epitope-tagged
LMW glutenin subunits
in the starchy endosperm of transgenic wheat and their incorporation
into glutenin polymers. Theoretical and Applied
Genetics.

[687] Song F., Cui C.-J., Chen L., Sun Y.-L., Wang F.-F., Hussain J., Li Y., Wang C., Wang C., Chen M.-J. (2012). Isolation and Characterization
of an Endosperm-Specific Promoter from Wheat (Triticum aestivum L.)..

[688] Stoger E., Williams S., Keen D., Christou P. (1999). Constitutive
versus seed specific expression in transgenic wheat: temporal and
spatial control. Transgenic Research.

[689] Wang K., Zhang X., Zhao Y., Chen F., Xia G. (2013). Structure,
variation and expression analysis of glutenin gene promoters
from Triticum aestivum cultivar Chinese Spring shows the distal region
of promoter 1Bx7 is key regulatory sequence. Gene.

[690] Chen Z. L., Pan N. S., Beachy R. N. (1988). A DNA sequence element
that confers seed-specific enhancement to a constitutive promoter. The EMBO Journal.

[691] Chen X., Wang Z., Wang J., Wang M., Zhao L., Wang G. (2007). Isolation and characterization
of
Brittle2 promoter from Zea Mays and its comparison with Ze19 promoter
in transgenic tobacco plants. Plant Cell, Tissue
and Organ Culture.

[692] van der Steege G., Nieboer M., Swaving J., Tempelaar M. J. (1992). Potato
granule-bound starch synthase promoter-controlled GUS expression:
regulation of expression after transient and stable transformation. Plant Molecular Biology.

[693] Visser R. G. F., Stolte A., Jacobsen E. (1991). Expression
of a chimaeric
granule-bound starch synthase-GUS gene in transgenic potato plants. Plant Molecular Biology.

[694] Rocha-Sosa M., Sonnewald U., Frommer W., Stratmann M., Schell J., Willmitzer L. (1989). Both developmental
and metabolic
signals activate the promoter of a class I patatin gene. The EMBO Journal.

[695] Bevan M., Barker R., Goldsbrough A., Jarvis M., Kavanagh T., Iturriaga G. (1986). The structure
and transcription start site of major potato tuber protine gene. Nucleic Acids Research.

[696] Liu X.-Y., Rocha-Sosa M., Hummel S., Willmitzer L., Frommer W. B. (1991). A detailed study
of the regulation and evolution of
the two classes of patatin genes in Solanum tuberosum L. Plant Molecular Biology.

[697] Li M., Song B., Zhang Q., Liu X., Lin Y., Ou Y., Zhang H., Liu J. (2013). A synthetic
tuber-specific and cold-induced
promoter is applicable in controlling potato cold-induced sweetening. Plant Physiology and Biochemistry.

[698] Zhang L., Yang T., Li X., Hao H., Xu S., Cheng W., Sun Y., Wang C. (2014). Cloning and
characterization
of a novel Athspr promoter specifically active in vascular tissue. Plant Physiology and Biochemistry.

[699] Xu W., Liu W., Ye R., Mazarei M., Huang D., Zhang X., Stewart C. N. (2018). A profilin
gene promoter from switchgrass
(Panicum virgatum L.) directs strong and specific transgene expression
to vascular bundles in rice. Plant Cell Reports.

[700] Yu H., Khalid M. H. B., Lu F., Sun F., Qu J., Liu B., Li W., Fu F. (2019). Isolation and identification of a
vegetative organ-specific promoter from maize. Physiology and Molecular Biology of Plants.

[701] Cho J.-S., Jeon H.-W., Kim M.-H., Vo T. K., Kim J., Park E.-J., Choi Y.-I., Lee H., Han K.-H., Ko J.-H. (2019). Wood forming tissue-specific bicistronic
expression of PdGA20ox1
and PtrMYB221 improves both the quality and quantity of woody biomass
production in a hybrid poplar. Plant Biotechnology
Journal.

[702] Zheng H., Lei Y., Lin S., Zhang Q., Zhang Z. (2011). Bidirectionalization
of a methyl jasmonate-inducible plant promoter. Biotechnology Letters.

[703] Ko J.-H., Kim H.-T., Hwang I., Han K.-H. (2012). Tissue-type-specific
transcriptome analysis identifies developing xylem-specific promoters
in poplar. Plant Biotechnology Journal.

[704] Arnaiz A., Martinez M., Gonzalez-Melendi P., Grbic V., Diaz I., Santamaria M. E. (2019). Plant Defenses
Against Pests Driven by a Bidirectional Promoter. Front Plant Sci.

[705] Mitra A., Han J., Zhang Z. J., Mitra A. (2009). The intergenic
region of Arabidopsis thaliana cab1 and cab2 divergent genes functions
as a bidirectional promoter. Planta.

[706] Wang R., Yan Y., Zhu M., Yang M., Zhou F., Chen H., Lin Y. (2016). Isolation and Functional
Characterization of Bidirectional Promoters in Rice. Front Plant Sci.

[707] Dhadi S. R., Deshpande A., Driscoll K., Ramakrishna W. (2013). Major cis-regulatory
elements for rice bidirectional promoter activity reside in the 5-untranslated
regions. Gene.

[708] Xie M., He Y., Gan S. (2001). Bidirectionalization
of polar promoters
in plants. Nat Biotechnol.

[709] Kumar S., AlAbed D., Whitteck J. T., Chen W., Bennett S., Asberry A., Wang X., DeSloover D., Rangasamy M., Wright T. R. (2015). A combinatorial
bidirectional
and bicistronic approach for coordinated multi-gene expression in
corn. Plant Mol Biol.

[710] Ren Q., Zhong Z., Wang Y., You Q., Li Q., Yuan M., He Y., Qi C., Tang X., Zheng X. (2019). Bidirectional Promoter-Based
CRISPR-Cas9 Systems for
Plant Genome Editing. Front Plant Sci.

[711] Gondalia N., Quiroz L. F., Lai L., Singh A. K., Khan M., Brychkova G., McKeown P. C., Chatterjee M., Spillane C. (2025). Harnessing promoter
elements to enhance gene editing
in plants: perspectives and advances. Plant
Biotechnol J.

[712] Kor S. D., Chowdhury N., Keot A. K., Yogendra K., Chikkaputtaiah C., Sudhakar Reddy P. (2023). RNA Pol III promoters-key players
in precisely targeted plant genome editing. Front Genet.

[713] Massel K., Lam Y., Hintzsche J., Lester N., Botella J. R., Godwin I. D. (2022). Endogenous U6 promoters
improve CRISPR/Cas9 editing efficiencies in Sorghum bicolor and show
potential for applications in other cereals. Plant Cell Reports.

[714] Duttke S. H. (2014). RNA polymerase III accurately initiates transcription
from RNA polymerase II promoters in vitro. J
Biol Chem.

[715] Schramm L., Hernandez N. (2002). Recruitment of RNA polymerase III
to its target promoters. Genes Dev.

[716] Hao Y., Zong W., Zeng D., Han J., Chen S., Tang J., Zhao Z., Li X., Ma K., Xie X. (2020). Shortened snRNA promoters for efficient CRISPR/Cas-based
multiplex genome editing in monocot plants. Sci China Life Sci.

[717] Long L., Guo D. D., Gao W., Yang W. W., Hou L. P., Ma X. N., Miao Y. C., Botella J. R., Song C. P. (2018). Optimization of CRISPR/Cas9 genome editing in cotton
by improved sgRNA expression. Plant Methods.

[718] Sun X., Hu Z., Chen R., Jiang Q., Song G., Zhang H., Xi Y. (2015). Targeted mutagenesis
in soybean using
the CRISPR-Cas9 system. Sci Rep.

[719] Di Y. H., Sun X. J., Hu Z., Jiang Q. Y., Song G. H., Zhang B., Zhao S. S., Zhang H. (2019). Enhancing
the CRISPR/Cas9 system based on multiple GmU6 promoters in soybean. Biochem Biophys Res Commun.

[720] Dai X., Yang X., Wang C., Fan Y., Xin S., Hua Y., Wang K., Huang H. (2021). CRISPR/Cas9-mediated
genome editing
in Hevea brasiliensis. Industrial Crops and
Products.

[721] Zhu X., Xu W., Liu B., Zhan Y., Xia T. (2023). Adaptation
of high-efficiency CRISPR/Cas9-based multiplex genome editing system
in white lupin by using endogenous promoters. Physiol Plant.

[722] Zhang S., Wu S., Hu C., Yang Q., Dong T., Sheng O., Deng G., He W., Dou T., Li C. (2022). Increased mutation efficiency of CRISPR/Cas9
genome editing in banana by optimized construct. PeerJ.

[723] Ren C., Liu Y., Guo Y., Duan W., Fan P., Li S., Liang Z. (2021). Optimizing
the CRISPR/Cas9 system for genome editing
in grape by using grape promoters. Hortic Res.

[724] Mikami M., Toki S., Endo M. (2015). Comparison
of CRISPR/Cas9
expression constructs for efficient targeted mutagenesis in rice. Plant Mol Biol.

[725] Sultana M. S., Frazier T. P., Millwood R. J., Lenaghan S. C., Stewart C. N. (2019). Development and
validation of a novel
and robust cell culture system in soybean (Glycine max (L.) Merr.)
for promoter screening. Plant Cell Rep.

[726] Pfotenhauer A. C., Occhialini A., Nguyen M. A., Scott H., Dice L. T., Harbison S. A., Li L., Reuter D. N., Schimel T. M., Stewart C. N. (2022). Building the Plant SynBio
Toolbox through Combinatorial Analysis
of DNA Regulatory Elements. ACS Synth Biol.

[727] Rathus C., Bower R., Birch R. G. (1993). Effects of promoter,
intron and enhancer elements on transient gene expression in sugar-cane
and carrot protoplasts. Plant Mol Biol.

[728] Canton B., Labno A., Endy D. (2008). Refinement and standardization
of synthetic biological parts and devices. Nat
Biotechnol.

[729] Nielsen A. A., Der B. S., Shin J., Vaidyanathan P., Paralanov V., Strychalski E. A., Ross D., Densmore D., Voigt C. A. (2016). Genetic circuit design automation. Science.

[730] Shao B., Rammohan J., Anderson D. A., Alperovich N., Ross D., Voigt C. A. (2021). Single-cell measurement of plasmid
copy number and promoter activity. Nat Commun.

[731] Kong C., Yang Y., Qi T., Zhang S. (2025). Predictive
genetic circuit design for phenotype reprogramming in plants. Nat Commun.

[732] de Felippes F., McHale M., Doran R. L., Roden S., Eamens A. L., Finnegan E. J., Waterhouse P. M. (2020). The key
role of terminators on the expression and post-transcriptional gene
silencing of transgenes. Plant Journal.

[733] Kuehner J.
N., Pearson E. L., Moore C. (2011). Unravelling the means
to an end: RNA polymerase II transcription termination. Nature reviews Molecular cell biology.

[734] Xie J., Aiello U., Clement Y., Haidara N., Girbig M., Schmitzova J., Pena V., Muller C. W., Libri D., Porrua O. (2022). An integrated
model for termination of RNA polymerase
III transcription. Sci Adv.

[735] Chen, Y.-J. ; Liu, P. ; Nielsen, A. A. ; Brophy, J. A. ; Clancy, K. ; Peterson, T. ; Voigt, C. A. J. N. m. Characterization of 582 natural and synthetic terminators and quantification of their design constraints. Nat Methods 2013, 10 (7), 659. 10.1038/nmeth.2515 23727987

[736] Cambray G., Guimaraes J. C., Mutalik V. K., Lam C., Mai Q. A., Thimmaiah T., Carothers J. M., Arkin A. P., Endy D. (2013). Measurement and modeling
of intrinsic
transcription terminators. Nucleic Acids Res.

[737] O'Sullivan J. M., Tan-Wong S. M., Morillon A., Lee B., Coles J., Mellor J., Proudfoot N. J. (2004). Gene loops
juxtapose promoters and terminators in yeast. Nat Genet.

[738] Curran K. A., Karim A. S., Gupta A., Alper H. S. (2013). Use of
expression-enhancing terminators in Saccharomyces cerevisiae to increase
mRNA half-life and improve gene expression control for metabolic engineering
applications. Metab Eng.

[739] Zhao L., Wang S., Cao Z., Ouyang W., Zhang Q., Xie L., Zheng R., Guo M., Ma M., Hu Z. (2019). Chromatin loops associated
with active genes
and heterochromatin shape rice genome architecture for transcriptional
regulation. Nat Commun.

[740] Liu C., Wang C., Wang G., Becker C., Zaidem M., Weigel D. (2016). Genome-wide analysis
of chromatin packing in Arabidopsis
thaliana at single-gene resolution. Genome Res.

[741] Zhao, B. ; Xi, Y. ; Kim, J. ; Sung, S. Chromatin architectural proteins regulate flowering time by precluding gene looping. Sci Adv 2021, 7 (24), 10.1126/sciadv.abg3097. PMC8195489 34117065

[742] Randise-Hinchliff C. E., Brickner J. H. (2012). A new direction for gene looping. Dev Cell.

[743] Moabbi A. M., Agarwal N., El Kaderi B., Ansari A. (2012). Role for gene looping in intron-mediated enhancement
of transcription. Proc Natl Acad Sci U S A.

[744] Tan-Wong S. M., Zaugg J. B., Camblong J., Xu Z., Zhang D. W., Mischo H. E., Ansari A. Z., Luscombe N. M., Steinmetz L. M., Proudfoot N. J. (2012). Gene loops enhance transcriptional
directionality. Science.

[745] Mukundan B., Ansari A. (2013). Srb5/Med18-mediated
termination of
transcription is dependent on gene looping. J Biol Chem.

[746] Medler S., Ansari A. (2015). Gene looping facilitates TFIIH kinase-mediated
termination of transcription. Sci Rep.

[747] Wang C., Liu C., Roqueiro D., Grimm D., Schwab R., Becker C., Lanz C., Weigel D. (2015). Genome-wide
analysis of local chromatin packing in Arabidopsis thaliana. Genome Res.

[748] Grob S., Schmid M. W., Grossniklaus U. (2014). Hi-C analysis
in Arabidopsis identifies the KNOT, a structure with similarities
to the flamenco locus of Drosophila. Mol Cell.

[749] Dong P., Tu X., Chu P. Y., Lu P., Zhu N., Grierson D., Du B., Li P., Zhong S. (2017). 3D Chromatin
Architecture of Large Plant Genomes Determined by Local A/B Compartments. Mol Plant.

[750] Dong P., Tu X., Li H., Zhang J., Grierson D., Li P., Zhong S. (2020). Tissue-specific
Hi-C
analyses of rice, foxtail millet and maize suggest non-canonical function
of plant chromatin domains. J Integr Plant Biol.

[751] Ricci W. A., Lu Z., Ji L., Marand A. P., Ethridge C. L., Murphy N. G., Noshay J. M., Galli M., Mejia-Guerra M. K., Colome-Tatche M. (2019). Widespread long-range
cis-regulatory elements in the maize genome. Nat Plants.

[752] Dong Q., Li N., Li X., Yuan Z., Xie D., Wang X., Li J., Yu Y., Wang J., Ding B. (2018). Genome-wide Hi-C analysis reveals extensive hierarchical
chromatin interactions in rice. Plant J.

[753] Yang Q., Zuo D., Cheng H., Zhang Y., Wang Q., Javaria A., Feng X., Li S., Chen X., Liu S. (2021). Improved Gossypium raimondii genome
using a Hi-C-based proximity-guided assembly. Journal of Cotton Research.

[754] Du, Y. ; Liu, L. ; Peng, Y. ; Li, M. ; Li, Y. ; Liu, D. ; Li, X. ; Zhang, Z. UNBRANCHED3 Expression and Inflorescence Development is Mediated by UNBRANCHED2 and the Distal Enhancer, KRN4, in Maize. PLoS Genet 2020, 16, e1008764. 10.1371/journal.pgen.1008764 32330129 PMC7202667

[755] Nagaya S., Kawamura K., Shinmyo A., Kato K. (2010). The HSP terminator
of arabidopsis thaliana increases gene expression in plant cells. Plant and Cell Physiology.

[756] Diamos A. G., Mason H. S. (2018). Chimeric 3 flanking
regions strongly
enhance gene expression in plants. Plant Biotechnol
J.

[757] Bernardes W. S., Menossi M. (2020). Plant 3 Regulatory Regions From mRNA-Encoding
Genes and Their Uses to Modulate Expression. Front Plant Sci.

[758] de Felippes F. F., Waterhouse P. M. (2023). Plant terminators:
the unsung heroes
of gene expression. J Exp Bot.

[759] Merchante C., Stepanova A. N., Alonso J. M. (2017). Translation regulation
in plants: an interesting past, an exciting present and a promising
future. Plant Journal.

[760] Miras, M. ; Miller, W. A. ; Truniger, V. ; Aranda, M. A. Non-canonical translation in Plant RNA viruses. Frontiers in Plant Science 2017, 8 (April), 10.3389/fpls.2017.00494. PMC5382211 28428795

[761] Mao Y., Liu H., Liu Y., Tao S. (2014). Deciphering the rules
by which dynamics of mRNA secondary structure affect translation efficiency
in Saccharomyces cerevisiae. Nucleic Acids Res.

[762] Harvey R. F., Smith T. S., Mulroney T., Queiroz R. M. L., Pizzinga M., Dezi V., Villenueva E., Ramakrishna M., Lilley K. S., Willis A. E. (2018). Trans-acting translational
regulatory RNA binding proteins. Wiley Interdiscip
Rev RNA.

[763] Wang H. L., Chekanova J. A. (2016). Small RNAs: essential regulators
of gene expression and defenses against environmental stresses in
plants. Wiley Interdiscip Rev RNA.

[764] Kanoria S., Burma P. K. (2012). A 28 nt long synthetic
5 ′
UTR (synJ) as an enhancer of transgene expression in dicotyledonous
plants. BMC Biotechnology.

[765] Dorokhov Y. L., Skulachev M. V., Ivanov P. A., Zvereva S. D., Tjulkina L. G., Merits A., Gleba Y. Y., Hohn T., Atabekov J. G. (2002). Polypurine (A)-rich
sequences promote cross-kingdom
conservation of internal ribosome entry. P Natl
Acad Sci USA.

[766] Srivastava A. K., Lu Y., Zinta G., Lang Z., Zhu J.-K. (2018). UTR-dependent control of gene expression in plants. Trends in Plant Science.

[767] Fan Q., Treder K., Miller W. A. (2012). Untranslated
regions of diverse plant
viral RNAs vary greatly in translation enhancement efficiency. BMC Biotechnol.

[768] Martınez-Trujillo M., Limones-Briones V., Chávez-Bárcenas T., Herrera-Estrella L. (2003). Functional
analysis of the 5′ untranslated region of the sucrose phosphate
synthase rice gene (sps1). Plant Science.

[769] Satoh J., Kato K., Shinmyo A. (2004). The 5′-untranslated
region of the tobacco alcohol dehydrogenase gene functions as an effective
translational enhancer in plant. Journal of
bioscience and bioengineering.

[770] Nyikó T., Sonkoly B., Mérai Z., Benkovics A. H., Silhavy D. (2009). Plant upstream ORFs can trigger nonsense-mediated
mRNA decay in a size-dependent manner. Plant
molecular biology.

[771] Ivanov I. P., Atkins J. F., Michael A. J. (2010). A profusion of upstream
open reading frame mechanisms in polyamine-responsive translational
regulation. Nucleic acids research.

[772] Hayden C. A., Jorgensen R. A. (2007). Identification
of novel conserved
peptide uORF homology groups in Arabidopsis and rice reveals ancient
eukaryotic origin of select groups and preferential association with
transcription factor-encoding genes. BMC biology.

[773] Xu G., Yuan M., Ai C., Liu L., Zhuang E., Karapetyan S., Wang S., Dong X. (2017). uORF-mediated
translation
allows engineered plant disease resistance without fitness costs. Nature.

[774] De Amicis F., Patti T., Marchetti S. (2007). Improvement
of the pBI121 plant expression vector by leader replacement with a
sequence combining a poly(CAA) and a CT motif. Transgenic Res.

[775] Tanaka H., Suzuki Y., Yamasaki S., Yoshino K., Kato K., Nakamura S. (2018). R-STEINER: Generation Method of 5UTR
for Increasing the Amount of Translated Proteins. IPSJ Transactions on Bioinformatics.

[776] Peyret H., Brown J. K., Lomonossoff G. P. (2019). Improving
plant transient expression through the rational design of synthetic
5′ and 3′ untranslated regions. Plant methods.

[777] Zhu B., Zhang W., Zhang T., Liu B., Jiang J. (2015). Genome-wide
prediction and validation of intergenic enhancers in arabidopsis using
open chromatin signatures. Plant Cell.

[778] Bally J., Fishilevich E., Bowling A. J., Pence H. E., Narva K. E., Waterhouse P. M. (2018). Improved insect-proofing: expressing
double-stranded RNA in chloroplasts. Pest Manag
Sci.

[779] Gallie D. R., Lucas W. J., Walbot V. (1989). Visualizing mRNA expression
in plant protoplasts: factors influencing efficient mRNA uptake and
translation. Plant Cell.

[780] Matsui T., Sawada K., Takita E., Kato K. (2015). Compatibility
of translational enhancers with various plant species. Plant Biotechnology.

[781] Samadder P., Sivamani E., Lu J., Li X., Qu R. (2008). Transcriptional and post-transcriptional enhancement
of gene expression
by the 5 UTR intron of rice rubi3 gene in transgenic rice cells. Mol Genet Genomics.

[782] Le Hir H., Nott A., Moore M. J. (2003). How introns
influence
and enhance eukaryotic gene expression. Trends
in Biochemical Sciences.

[783] Bartlett J. G., Snape J. W., Harwood W. A. (2009). Intron-mediated
enhancement as a method for increasing transgene expression levels
in barley. Plant Biotechnol J.

[784] Laxa M. (2017). Intron-mediated enhancement: A tool
for heterologous gene expression
in plants?. Frontiers in Plant Science.

[785] Liao L., Ning G., Liu C., Zhang W., Bao M. (2013). The intron from the 5-UTR of the
FBP11 gene in petunia displays promoter-
and enhancer-like functions. Scientia Horticulturae.

[786] Morita S., Tsukamoto S., Sakamoto A., Makino H., Nakauji E., Kaminaka H., Masumura T., Ogihara Y., Satoh S., Tanaka K. (2012). Differences
in intron-mediated enhancement
of gene expression by the first intron of cytosolic superoxide dismutase
gene from rice in monocot and dicot plants. Plant Biotechnology.

[787] Rose A. B., Elfersi T., Parra G., Korf I. (2008). Promoter-proximal
introns in Arabidopsis thaliana are enriched in dispersed signals
that elevate gene expression. The Plant Cell.

[788] Parra G., Bradnam K., Rose A. B., Korf I. (2011). Comparative
and functional analysis of intron-mediated enhancement signals reveals
conserved features among plants. Nucleic acids
research.

[789] Bartlett J. G., Snape J. W., Harwood W. A. (2009). Intron-mediated
enhancement as a method for increasing transgene expression levels
in barley. Plant biotechnology journal.

[790] Smith N. A., Singh S. P., Wang M.-B., Stoutjesdijk P. A., Green A. G., Waterhouse P. M. (2000). Total silencing by intron-spliced
hairpin RNAs. Nature.

[791] Baird S. D., Turcotte M., Korneluk R. G., Holcik M. (2006). Searching
for IRES. Rna.

[792] Zong Y., Zhang H. M., Lyu C., Ji X., Hou J., Guo X., Ouyang Q., Lou C. (2017). Insulated
transcriptional
elements enable precise design of genetic circuits. Nat Commun.

[793] Hughes J. R., Roberts N., McGowan S., Hay D., Giannoulatou E., Lynch M., De Gobbi M., Taylor S., Gibbons R., Higgs D. R. (2014). Analysis of hundreds of cis-regulatory
landscapes at high resolution in a single, high-throughput experiment. Nat Genet.

[794] Lieberman-Aiden E., van Berkum N. L., Williams L., Imakaev M., Ragoczy T., Telling A., Amit I., Lajoie B. R., Sabo P. J., Dorschner M. O. (2009). Comprehensive mapping
of long-range interactions reveals folding principles of the human
genome. Science.

[795] Wang C., Liu C., Roqueiro D., Grimm D., Schwab R., Becker C., Lanz C., Weigel D. (2015). Genome-wide
analysis of local chromatin packing in Arabidopsis thaliana. Genome research.

[796] Han J., Wang S., Wu H., Zhao T., Guan X., Fang L. (2023). An upgraded method
of high-throughput chromosome conformation capture
(Hi-C 3.0) in cotton (Gossypium spp.). Front
Plant Sci.

[797] Wei G. H., Liu D. P., Liang C. C. (2005). Chromatin
domain
boundaries: insulators and beyond. Cell Res.

[798] Dolgova A. S., Dolgov S. V. (2019). Matrix attachment regions as a tool
to influence plant transgene expression. 3 Biotech.

[799] Van der Geest A. H., Welter M. E., Woosley A. T., Pareddy D. R., Pavelko S. E., Skokut M., Ainley W. M. (2004). A short
synthetic
MAR positively affects transgene expression in rice and Arabidopsis. Plant Biotechnol J.

[800] Liebich I., Bode J., Frisch M., Wingender E. (2002). S/MARt DB:
a database on scaffold/matrix attached regions. Nucleic Acids Research.

[801] Ratcliff F., Harrison B. D., Baulcombe D. C. (1997). A similarity
between viral defense and gene silencing in plants. Science.

[802] de Felippes F., McHale M., Doran R. L., Roden S., Eamens A. L., Finnegan E. J., Waterhouse P. M. (2020). The key
role of terminators on the expression and post-transcriptional gene
silencing of transgenes. Plant J.

[803] Luo Z., Chen Z. (2007). Improperly terminated,
unpolyadenylated mRNA of sense
transgenes is targeted by RDR6-mediated RNA silencing in Arabidopsis. Plant Cell.

[804] Dadami E., Moser M., Zwiebel M., Krczal G., Wassenegger M., Dalakouras A. (2013). An endogene-resembling transgene
delays the onset of silencing and limits siRNA accumulation. FEBS Lett.

[805] Vaistij F. E., Jones L., Baulcombe D. C. (2002). Spreading
of RNA targeting and DNA methylation in RNA silencing requires transcription
of the target gene and a putative RNA-dependent RNA polymerase. Plant Cell.

[806] Christie M., Croft L. J., Carroll B. J. (2011). Intron splicing
suppresses RNA silencing in Arabidopsis. Plant
J.

[807] Lakatos L., Szittya G., Silhavy D., Burgyán J. (2004). Molecular
mechanism of RNA silencing suppression mediated by p19 protein of
tombusviruses. Embo J.

[808] Nielsen A. A. K., Der B. S., Shin J., Vaidyanathan P., Paralanov V., Strychalski E. A., Ross D., Densmore D., Voigt C. A. (2016). Genetic circuit
design automation. Science.

[809] Guo Q., Liu Q., Smith N. A., Liang G., Wang M. B. (2016). RNA Silencing
in Plants: Mechanisms, Technologies and Applications in Horticultural
Crops. Curr Genomics.

[810] Ogita S., Uefuji H., Yamaguchi Y., Koizumi N., Sano H. (2003). Producing decaffeinated coffee plants. Nature.

[811] Waterhouse P. M., Graham H. W., Wang M. B. (1998). Virus resistance
and gene silencing in plants can be induced by simultaneous expression
of sense and antisense RNA. P Natl Acad Sci
USA.

[812] Ledger S. E., Janssen B. J., Karunairetnam S., Wang T. C., Snowden K. C. (2010). Modified CAROTENOID CLEAVAGE DIOXYGENASE8
expression correlates with altered branching in kiwifruit (Actinidia
chinensis). New Phytol.

[813] Nozawa A., Ogasawara T., Matsunaga S., Iwasaki T., Sawasaki T., Endo Y. (2011). Production
and partial
purification of membrane proteins using a liposome-supplemented wheat
cell-free translation system. BMC Biotechnol.

[814] Redenbaugh, K. Safety assessment of genetically engineered fruits and vegetables: a case study of the Flavr Savr Tomato; CRC Press, 2014.

[815] Lamke J., Baurle I. (2017). Epigenetic and chromatin-based
mechanisms
in environmental stress adaptation and stress memory in plants. Genome Biol.

[816] Dommes A. B., Gross T., Herbert D. B., Kivivirta K. I., Becker A. (2019). Virus-induced gene silencing: empowering genetics in
non-model organisms. J Exp Bot.

[817] Gao Z., Liu H. L., Daxinger L., Pontes O., He X., Qian W., Lin H., Xie M., Lorkovic Z. J., Zhang S. (2010). An RNA polymerase II-
and AGO4-associated protein acts
in RNA-directed DNA methylation. Nature.

[818] Wesley S. V., Helliwell C. A., Smith N. A., Wang M. B., Rouse D. T., Liu Q., Gooding P. S., Singh S. P., Abbott D., Stoutjesdijk P. A. (2001). Construct design for
efficient, effective and high-throughput gene silencing in plants. Plant J.

[819] Burch-Smith T. M., Anderson J. C., Martin G. B., Dinesh-Kumar S. P. (2004). Applications
and advantages of virus-induced gene silencing for gene function studies
in plants. Plant J.

[820] Lindbo J. A., Dougherty W. G. (2005). Plant pathology
and RNAi: a brief
history. Annu Rev Phytopathol.

[821] Tang G., Galili G. (2004). Using RNAi to improve
plant nutritional
value: from mechanism to application. Trends
Biotechnol.

[822] Das P. R., Sherif S. M. (2020). Application of Exogenous dsRNAs-induced
RNAi in Agriculture: Challenges and Triumphs. Front Plant Sci.

[823] Liu S., Geng S., Li A., Mao Y., Mao L. (2021). RNAi technology
for plant protection and its application in wheat. aBIOTECH.

[824] Nakatsuka T., Mishiba K., Kubota A., Abe Y., Yamamura S., Nakamura N., Tanaka Y., Nishihara M. (2010). Genetic engineering
of novel flower colour by suppression of anthocyanin modification
genes in gentian. J Plant Physiol.

[825] Barros J., Temple S., Dixon R. A. (2019). Development and
commercialization of reduced lignin alfalfa. Curr Opin Biotechnol.

[826] Guo D., Chen F., Inoue K., Blount J. W., Dixon R. A. (2001). Downregulation
of caffeic acid 3-O-methyltransferase and caffeoyl CoA 3-O-methyltransferase
in transgenic alfalfa. impacts on lignin structure and implications
for the biosynthesis of G and S lignin. Plant
Cell.

[827] Abbott J. C., Barakate A., Pincon G., Legrand M., Lapierre C., Mila I., Schuch W., Halpin C. (2002). Simultaneous
suppression of multiple genes by single transgenes. Down-regulation
of three unrelated lignin biosynthetic genes in tobacco. Plant Physiology.

[828] Dodo H. W., Konan K. N., Chen F. C., Egnin M., Viquez O. M. (2008). Alleviating peanut allergy using
genetic engineering:
the silencing of the immunodominant allergen Ara h 2 leads to its
significant reduction and a decrease in peanut allergenicity. Plant biotechnology journal.

[829] Ossowski S., Schwab R., Weigel D. (2008). Gene silencing
in plants
using artificial microRNAs and other small RNAs. Plant J.

[830] Mickiewicz A., Rybarczyk A., Sarzynska J., Figlerowicz M., Blazewicz J. (2016). AmiRNA Designer
- new method of artificial
miRNA design. Acta Biochim Pol.

[831] Fahlgren N., Hill S. T., Carrington J. C., Carbonell A. (2016). P-SAMS: a web site for plant artificial microRNA and
synthetic trans-acting small interfering RNA design. Bioinformatics.

[832] Cisneros A. E., Martin-Garcia T., Primc A., Kuziuta W., Sanchez-Vicente J., Aragones V., Daros J. A., Carbonell A. (2023). Transgene-free,
virus-based gene silencing in plants by artificial microRNAs derived
from minimal precursors. Nucleic Acids Res.

[833] Niu Q. W., Lin S. S., Reyes J. L., Chen K. C., Wu H. W., Yeh S. D., Chua N. H. (2006). Expression of artificial
microRNAs in transgenic Arabidopsis thaliana confers virus resistance. Nat Biotechnol.

[834] Liu Q., Wang F., Axtell M. J. (2014). Analysis
of complementarity requirements
for plant microRNA targeting using a Nicotiana benthamiana quantitative
transient assay. Plant Cell.

[835] Schwab R., Palatnik J. F., Riester M., Schommer C., Schmid M., Weigel D. (2005). Specific effects of
microRNAs on
the plant transcriptome. Dev Cell.

[836] Schwab R., Ossowski S., Riester M., Warthmann N., Weigel D. (2006). Highly specific gene silencing by
artificial microRNAs
in Arabidopsis. Plant Cell.

[837] Warthmann N., Chen H., Ossowski S., Weigel D., Herve P. (2008). Highly specific gene silencing by
artificial miRNAs in rice. PLoS One.

[838] Kavuri, N. R. ; Ramasamy, M. ; Qi, Y. ; Mandadi, K. Applications of CRISPR/Cas13-Based RNA Editing in Plants. Cells 2022, 11 (17), 2665 10.3390/cells11172665. 36078073 PMC9454418

[839] Yu L., Zou J., Hussain A., Jia R., Fan Y., Liu J., Nie X., Zhang X., Jin S. (2024). Systemic evaluation
of various CRISPR/Cas13 orthologs for knockdown of targeted transcripts
in plants. Genome Biol.

[840] Aman R., Ali Z., Butt H., Mahas A., Aljedaani F., Khan M. Z., Ding S., Mahfouz M. (2018). RNA virus
interference via CRISPR/Cas13a system in plants. Genome Biol.

[841] Aman, R. ; Mahas, A. ; Butt, H. ; Aljedaani, F. ; Mahfouz, M. Engineering RNA Virus Interference via the CRISPR/Cas13 Machinery in Arabidopsis. Viruses 2018, 10 (12), 732 10.3390/v10120732. 30572690 PMC6315463

[842] Gupta R., Ghosh A., Chakravarti R., Singh R., Ravichandiran V., Swarnakar S., Ghosh D. (2022). Cas13d: A New Molecular Scissor for Transcriptome Engineering. Front Cell Dev Biol.

[843] Zhan X., Liu W., Nie B., Zhang F., Zhang J. (2023). Cas13d-mediated multiplex
RNA targeting confers a broad-spectrum
resistance against RNA viruses in potato. Commun
Biol.

[844] Konermann S., Lotfy P., Brideau N. J., Oki J., Shokhirev M. N., Hsu P. D. (2018). Transcriptome Engineering with RNA-Targeting
Type VI-D CRISPR Effectors. Cell.

[845] Cox D. B. T., Gootenberg J. S., Abudayyeh O. O., Franklin B., Kellner M. J., Joung J., Zhang F. (2017). RNA editing
with CRISPR-Cas13. Science.

[846] Sharma V. K., Marla S., Zheng W., Mishra D., Huang J., Zhang W., Morris G. P., Cook D. E. (2022). CRISPR
guides induce gene silencing in plants in the absence of Cas. Genome Biol.

[847] Kosugi S., Hasebe M., Matsumura N., Takashima H., Miyamoto-Sato E., Tomita M., Yanagawa H. (2009). Six classes
of nuclear localization signals specific to different binding grooves
of importinα. Journal of Biological Chemistry.

[848] von Heijne G., Steppuhn J., Herrmann R. G. (1989). Domain structure
of mitochondrial and chloroplast targeting peptides. Eur J Biochem.

[849] Song S. J., Diao H. P., Guo Y. F., Hwang I. (2024). Advances in
Subcellular Accumulation Design for Recombinant Protein Production
in Tobacco. Biodes Res.

[850] Spatola
Rossi T., Kriechbaumer V. (2023). An Interplay between Mitochondrial
and ER Targeting of a Bacterial Signal Peptide in Plants. Plants.

[851] Liaci A. M., Förster F. (2021). Take me home, protein roads: Structural
insights into signal peptide interactions during ER translocation. International Journal of Molecular Sciences.

[852] Napier R. M., Fowke L. C., Hawes C., Lewis M., Pelham H. R. (1992). Immunological evidence that plants
use both HDEL and
KDEL for targeting proteins to the endoplasmic reticulum. Journal of Cell Science.

[853] Yoshida K., Matsui T., Shinmyo A. (2004). The plant
vesicular
transport engineering for production of useful recombinant proteins. Journal of Molecular Catalysis B: Enzymatic.

[854] Xiang L., Etxeberria E., Van den Ende W. (2013). Vacuolar protein
sorting mechanisms in plants. FEBS J.

[855] Lu J., Wu T., Zhang B., Liu S., Song W., Qiao J., Ruan H. (2021). Types of nuclear localization signals
and mechanisms of protein import into the nucleus. Cell Commun Signal.

[856] Patron N. J., Waller R. F. (2007). Transit peptide diversity and divergence:
A global analysis of plastid targeting signals. Bioessays.

[857] Razzak M. A., Lee D. W., Yoo Y. J., Hwang I. (2017). Evolution
of rubisco complex small subunit transit peptides from algae to plants. Sci Rep.

[858] Chaumont F., de Castro Silva Filho M., Thomas D., Leterme S., Boutry M. (1994). Truncated presequences of mitochondrial
F1-ATPase beta subunit from Nicotiana plumbaginifolia transport CAT
and GUS proteins into mitochondria of transgenic tobacco. Plant Mol Biol.

[859] Glaser E., Sjoling S., Tanudji M., Whelan J. (1998). Mitochondrial
protein import in plants. Signals, sorting, targeting, processing
and regulation. Plant Mol Biol.

[860] van der Krol A. R., Chua N. H. (1991). The basic domain of plant B-ZIP proteins
facilitates import of a reporter protein into plant nuclei. Plant Cell.

[861] Lee D. W., Lee S., Lee J., Woo S., Razzak M. A., Vitale A., Hwang I. (2019). Molecular Mechanism
of the Specificity of Protein Import into Chloroplasts and Mitochondria
in Plant Cells. Mol Plant.

[862] Habib S. J., Neupert W., Rapaport D. (2007). Analysis and
prediction
of mitochondrial targeting signals. Methods
Cell Biol.

[863] von Heijne G. (1986). Mitochondrial
targeting sequences may form amphiphilic
helices. EMBO J.

[864] Allen, R. S. ; Tilbrook, K. ; Warden, A. C. ; Campbell, P. C. ; Rolland, V. ; Singh, S. P. ; Wood, C. C. Expression of 16 Nitrogenase Proteins within the Plant Mitochondrial Matrix. Frontiers in Plant Science 2017, 8, 10.3389/fpls.2017.00287. PMC5334340 28316608

[865] Lee S., Lee D. W., Yoo Y. J., Duncan O., Oh Y. J., Lee Y. J., Lee G., Whelan J., Hwang I. (2013). Mitochondrial
targeting of the Arabidopsis F1-ATPase gamma-subunit via multiple
compensatory and synergistic presequence motifs. Plant Cell.

[866] Chung K. P., Zeng Y. (2017). An Overview of Protein Secretion
in Plant Cells. Methods Mol Biol.

[867] Pool M. R. (2022). Targeting of proteins for translocation
at the endoplasmic
reticulum. International journal of molecular
sciences.

[868] Blake W. J., Kærn M., Cantor C. R., Collins J. J. (2003). Noise in
eukaryotic gene expression. Nature.

[869] Dacheux E., Malys N., Meng X., Ramachandran V., Mendes P., McCarthy J. E. (2017). Translation initiation events on
structured eukaryotic mRNAs generate gene expression noise. Nucleic Acids Research.

[870] Halpin C., Cooke S. E., Barakate A., El Amrani A., Ryan M. D. (1999). Self-processing 2A-polyproteins -
A system for co-ordinate
expression of multiple proteins in transgenic plants. Plant Journal.

[871] Gong Y. N., Tang R. Q., Zhang Y., Peng J., Xian O., Zhang Z. H., Zhang S. B., Zhang D. Y., Liu H., Luo X. W. (2020). The
NIa-Protease Protein Encoded by the Pepper
Mottle Virus Is a Pathogenicity Determinant and Releases DNA Methylation
of Nicotiana benthamiana. Front Microbiol.

[872] Goh C. J., Hahn Y. (2021). Analysis of proteolytic
processing
sites in potyvirus polyproteins revealed differential amino acid preferences
of NIa-Pro protease in each of seven cleavage sites. PLoS One.

[873] Yang D., Kim W. J., Yoo S. M., Choi J. H., Ha S. H., Lee M. H., Lee S. Y. (2018). Repurposing
type
III polyketide synthase as a malonyl-CoA biosensor for metabolic engineering
in bacteria. Proceedings of the National Academy
of Sciences.

[874] Ha S. H., Liang Y. S., Jung H., Ahn M. J., Suh S. C., Kweon S. J., Kim D. H., Kim Y. M., Kim J. K. (2010). Application of two bicistronic systems involving 2A
and IRES sequences to the biosynthesis of carotenoids in rice endosperm. Plant Biotechnol J.

[875] Stirdivant S. M., Crossland L. D., Bogorad L. (1985). DNA supercoiling affects
in vitro transcription of two maize chloroplast genes differently. Proc Natl Acad Sci U S A.

[876] Drechsel O., Bock R. (2011). Selection of Shine-Dalgarno
sequences
in plastids. Nucleic Acids Res.

[877] Scharff L. B., Childs L., Walther D., Bock R. (2011). Local absence
of secondary structure permits translation of mRNAs that lack ribosome-binding
sites. PLoS Genet.

[878] Schein A., Sheffy-Levin S., Glaser F., Schuster G. (2008). The RNase
E/G-type endoribonuclease of higher plants is located in the chloroplast
and cleaves RNA similarly to the E. coli enzyme. RNA.

[879] Ait-Bara S., Carpousis A. J. (2015). RNA degradosomes in bacteria and
chloroplasts: classification, distribution and evolution of RNase
E homologs. Mol Microbiol.

[880] Sporlein B., Streubel M., Dahlfeld G., Westhoff P., Koop H. U. (1991). PEG-mediated plastid transformation:
a new system for
transient gene expression assays in chloroplasts. Theor Appl Genet.

[881] Clark L., Voigt C. A., Jewett M. C. (2024). Establishing a High-Yield
Chloroplast Cell-Free System for Prototyping Genetic Parts. ACS Synth Biol.

[882] Suzuki J. Y., Sriraman P., Svab Z., Maliga P. (2003). Unique architecture
of the plastid ribosomal RNA operon promoter recognized by the multisubunit
RNA polymerase in tobacco and other higher plants. Plant Cell.

[883] Liere K., Maliga P. (1999). In vitro characterization of the
tobacco rpoB promoter reveals a core sequence motif conserved between
phage-type plastid and plant mitochondrial promoters. EMBO J.

[884] Hirose T., Sugiura M. (1996). Cis-acting elements and trans-acting
factors for accurate translation of chloroplast psbA mRNAs: development
of an in vitro translation system from tobacco chloroplasts. EMBO J.

[885] Yukawa M., Kuroda H., Sugiura M. (2007). A new in vitro
translation
system for non-radioactive assay from tobacco chloroplasts: effect
of pre-mRNA processing on translation in vitro. Plant J.

[886] Sleight S. C., Bartley B. A., Lieviant J. A., Sauro H. M. (2010). Designing
and engineering evolutionary robust genetic circuits. J Biol Eng.

[887] Brophy J. A., Voigt C. A. (2014). Principles of genetic
circuit design. Nat Methods.

[888] Iamtham S., Day A. (2000). Removal of antibiotic
resistance
genes from transgenic tobacco plastids. Nat
Biotechnol.

[889] Lovett S. T., Hurley R. L., Sutera V. A., Aubuchon R. H., Lebedeva M. A. (2002). Crossing
over between regions of limited homology in
Escherichia coli: RecA-dependent and RecA-independent pathways. Genetics.

[890] Bi X., Liu L. F. (1994). recA-independent and recA-dependent
intramolecular
plasmid recombination. Differential homology requirement and distance
effect. J Mol Biol.

[891] Dufourmantel N., Dubald M., Matringe M., Canard H., Garcon F., Job C., Kay E., Wisniewski J. P., Ferullo J. M., Pelissier B. (2007). Generation and characterization
of soybean and marker-free tobacco plastid transformants over-expressing
a bacterial 4-hydroxyphenylpyruvate dioxygenase which provides strong
herbicide tolerance. Plant Biotechnol J.

[892] Kode V., Mudd E. A., Iamtham S., Day A. (2006). Isolation
of precise plastid deletion mutants by homology-based excision: a
resource for site-directed mutagenesis, multi-gene changes and high-throughput
plastid transformation. Plant J.

[893] Chen Y. J., Liu P., Nielsen A. A., Brophy J. A., Clancy K., Peterson T., Voigt C. A. (2013). Characterization
of 582 natural and synthetic terminators and quantification of their
design constraints. Nat Methods.

[894] Reis A. C., Halper S. M., Vezeau G. E., Cetnar D. P., Hossain A., Clauer P. R., Salis H. M. (2019). Simultaneous
repression
of multiple bacterial genes using nonrepetitive extra-long sgRNA arrays. Nat Biotechnol.

[895] Xie G., Allison L. A. (2002). Sequences upstream
of the YRTA core region are essential
for transcription of the tobacco atpB NEP promoter in chloroplasts
in vivo. Curr Genet.

[896] Ortelt J., Link G. (2021). Plastid Gene Transcription:
An Update
on Promoters and RNA Polymerases. Methods Mol
Biol.

[897] Allison L. A., Maliga P. (1995). Light-responsive and transcription-enhancing
elements regulate the plastid psbD core promoter. EMBO J.

[898] Occhialini A., Piatek A. A., Pfotenhauer A. C., Frazier T. P., Stewart C. N., Lenaghan S. C. (2019). MoChlo:
A Versatile, Modular Cloning Toolbox for Chloroplast Biotechnology. Plant Physiol.

[899] Sun E., Wu B. W., Tewari K. K. (1989). In vitro
analysis of the pea chloroplast
16S rRNA gene promoter. Mol Cell Biol.

[900] Hirata N., Yonekura D., Yanagisawa S., Iba K. (2004). Possible involvement
of the 5-flanking region and the 5UTR of plastid
accD gene in NEP-dependent transcription. Plant
Cell Physiol.

[901] Gruissem W., Greenberg B. M., Zurawski G., Prescott D. M., Hallick R. B. (1983). Biosynthesis
of chloroplast transfer RNA in a spinach
chloroplast transcription system. Cell.

[902] Gruissem W., Zurawski G. (1985). Identification and mutational analysis
of the promoter for a spinach chloroplast transfer RNA gene. EMBO J.

[903] Bradley D., Gatenby A. A. (1985). Mutational analysis
of the maize
chloroplast ATPase-beta subunit gene promoter: the isolation of promoter
mutants in E. coli and their characterization in a chloroplast in
vitro transcription system. EMBO J.

[904] Eisermann A., Tiller K., Link G. (1990). In vitro transcription
and DNA binding characteristics of chloroplast and etioplast extracts
from mustard (Sinapis alba) indicate differential usage of the psbA
promoter. EMBO J.

[905] Kim M., Thum K. E., Morishige D. T., Mullet J. E. (1999). Detailed architecture
of the barley chloroplast psbD-psbC blue light-responsive promoter. J Biol Chem.

[906] Gruissem W., Zurawski G. (1985). Analysis of promoter
regions for
the spinach chloroplast rbcL, atpB and psbA genes. EMBO J.

[907] Tanaka M., Obokata J., Chunwongse J., Shinozaki K., Sugiura M. (1987). Rapid splicing and stepwise processing
of a transcript from the psbB operon in tobacco chloroplasts: determination
of the intron sites in petB and petD. Mol Gen
Genet.

[908] Miyagi T., Kapoor S., Sugita M., Sugiura M. (1998). Transcript
analysis of the tobacco plastid operon rps2/atpI/H/F/A reveals the
existence of a non-consensus type II (NCII) promoter upstream of the
atpI coding sequence. Mol Gen Genet.

[909] Kahlau S., Bock R. (2008). Plastid transcriptomics and translatomics
of tomato fruit development and chloroplast-to-chromoplast differentiation:
chromoplast gene expression largely serves the production of a single
protein. Plant Cell.

[910] Valkov V. T., Scotti N., Kahlau S., Maclean D., Grillo S., Gray J. C., Bock R., Cardi T. (2009). Genome-wide
analysis of plastid gene expression in potato leaf chloroplasts and
tuber amyloplasts: transcriptional and posttranscriptional control. Plant Physiol.

[911] Zhang J., Ruf S., Hasse C., Childs L., Scharff L. B., Bock R. (2012). Identification of cis-elements
conferring
high levels of gene expression in non-green plastids. Plant J.

[912] Valkov V. T., Gargano D., Manna C., Formisano G., Dix P. J., Gray J. C., Scotti N., Cardi T. (2011). High efficiency
plastid transformation in potato and regulation of transgene expression
in leaves and tubers by alternative 5 and 3 regulatory sequences. Transgenic Res.

[913] Emadpour M., Karcher D., Bock R. (2015). Boosting riboswitch
efficiency by RNA amplification. Nucleic Acids
Res.

[914] Buhot L., Horvath E., Medgyesy P., Lerbs-Mache S. (2006). Hybrid transcription
system for controlled plastid transgene expression. Plant J.

[915] Hoelscher M. P., Forner J., Calderone S., Kramer C., Taylor Z., Loiacono F. V., Agrawal S., Karcher D., Moratti F., Kroop X. (2022). Expression
strategies for the efficient synthesis of antimicrobial peptides in
plastids. Nat Commun.

[916] McBride K. E., Schaaf D. J., Daley M., Stalker D. M. (1994). Controlled
expression of plastid transgenes in plants based on a nuclear DNA-encoded
and plastid-targeted T7 RNA polymerase. Proc
Natl Acad Sci U S A.

[917] Magee A. M., Coyne S., Murphy D., Horvath E. M., Medgyesy P., Kavanagh T. A. (2004). T7 RNA polymerase-directed
expression
of an antibody fragment transgene in plastids causes a semi-lethal
pale-green seedling phenotype. Transgenic Res.

[918] Magee A. M., MacLean D., Gray J. C., Kavanagh T. A. (2007). Disruption
of essential plastid gene expression caused by T7 RNA polymerase-mediated
transcription of plastid transgenes during early seedling development. Transgenic Res.

[919] Temme K., Hill R., Segall-Shapiro T. H., Moser F., Voigt C. A. (2012). Modular control of multiple pathways
using engineered orthogonal T7 polymerases. Nucleic Acids Res.

[920] Davidson E. A., Van Blarcom T., Levy M., Ellington A. D. (2009). Emulsion
based selection of T7 promoters of varying activity. Pac Symp Biocomput.

[921] Schindel H. S., Piatek A. A., Stewart C. N., Lenaghan S. C. (2018). The plastid
genome as a chassis for synthetic biology-enabled metabolic engineering:
players in gene expression. Plant Cell Reports.

[922] Shine J., Dalgarno L. (1974). The 3-terminal sequence of Escherichia
coli 16S ribosomal RNA: complementarity to nonsense triplets and ribosome
binding sites. Proc Natl Acad Sci U S A.

[923] McCarthy J. E., Gualerzi C. (1990). Translational control of prokaryotic
gene expression. Trends Genet.

[924] Bonham-Smith P. C., Bourque D. P. (1989). Translation of chloroplast-encoded
mRNA: potential initiation and termination signals. Nucleic Acids Res.

[925] Gillham N. W., Boynton J. E., Hauser C. R. (1994). Translational
regulation
of gene expression in chloroplasts and mitochondria. Annu Rev Genet.

[926] Hirose T., Sugiura M. (2004). Multiple elements required
for translation
of plastid atpB mRNA lacking the Shine-Dalgarno sequence. Nucleic Acids Res.

[927] Sugiura M., Hirose T., Sugita M. (1998). Evolution
and mechanism
of translation in chloroplasts. Annu Rev Genet.

[928] Scharff L. B., Ehrnthaler M., Janowski M., Childs L. H., Hasse C., Gremmels J., Ruf S., Zoschke R., Bock R. (2017). Shine-Dalgarno Sequences Play an
Essential Role in the Translation
of Plastid mRNAs in Tobacco. Plant Cell.

[929] Zoschke R., Bock R. (2018). Chloroplast Translation: Structural
and Functional Organization, Operational Control, and Regulation. Plant Cell.

[930] Chotewutmontri P., Barkan A. (2016). Dynamics of Chloroplast Translation
during Chloroplast Differentiation in Maize. PLoS Genet.

[931] Boyer S. K., Mullet J. E. (1986). Characterization of P. sativum chloroplast
psbA transcripts produced in vivo, in vitro and in E. coli. Plant Mol Biol.

[932] Gallaher, S. D. ; Craig, R. J. ; Ganesan, I. ; Purvine, S. O. ; McCorkle, S. R. ; Grimwood, J. ; Strenkert, D. ; Davidi, L. ; Roth, M. S. ; Jeffers, T. L. ; Widespread polycistronic gene expression in green algae. Proc Natl Acad Sci U S A 2021, 118 (7), 10.1073/pnas.2017714118. PMC7896298 33579822

[933] Yang H., Gray B. N., Ahner B. A., Hanson M. R. (2013). Bacteriophage
5 untranslated regions for control of plastid transgene expression. Planta.

[934] Kuroda H., Maliga P. (2001). Complementarity of the 16S rRNA penultimate
stem with sequences downstream of the AUG destabilizes the plastid
mRNAs. Nucleic Acids Res.

[935] Eibl C., Zou Z., Beck a., Kim M., Mullet J., Koop H. U. (1999). In vivo analysis of plastid psbA,
rbcL and rpl32 UTR elements by chloroplast transformation: tobacco
plastid gene expression is controlled by modulation of transcript
levels and translation efficiency. Plant J.

[936] Zou Z., Eibl C., Koop H. U. (2003). The stem-loop region of the tobacco
psbA 5UTR is an important determinant of mRNA stability and translation
efficiency. Mol Genet Genomics.

[937] Legen J., Ruf S., Kroop X., Wang G., Barkan A., Bock R., Schmitz-Linneweber C. (2018). Stabilization
and translation of synthetic operon-derived mRNAs in chloroplasts
by sequences representing PPR protein-binding sites. Plant J.

[938] Kuroda H., Suzuki H., Kusumegi T., Hirose T., Yukawa Y., Sugiura M. (2007). Translation of psbC mRNAs starts
from the downstream GUG, not the upstream AUG, and requires the extended
Shine-Dalgarno sequence in tobacco chloroplasts. Plant Cell Physiol.

[939] Kuroda H., Sugiura M. (2014). Processing of the 5-UTR
and existence
of protein factors that regulate translation of tobacco chloroplast
psbN mRNA. Plant Mol Biol.

[940] Baecker J. J., Sneddon J. C., Hollingsworth M. J. (2009). Efficient
translation in chloroplasts requires element(s) upstream of the putative
ribosome binding site from atpI. Am J Bot.

[941] Hirose T., Kusumegi T., Sugiura M. (1998). Translation of tobacco
chloroplast rps14 mRNA depends on a Shine-Dalgarno-like sequence in
the 5-untranslated region but not on internal RNA editing in the coding
region. FEBS Lett.

[942] Rojas M., Yu Q., Williams-Carrier R., Maliga P., Barkan A. (2019). Engineered PPR proteins as inducible
switches to activate the expression of chloroplast transgenes. Nat Plants.

[943] Plader W., Sugiura M. (2003). The Shine-Dalgarno-like sequence
is a negative regulatory element for translation of tobacco chloroplast
rps2 mRNA: an additional mechanism for translational control in chloroplasts. Plant J.

[944] Hirose T., Sugiura M. (2004). Functional Shine-Dalgarno-like
sequences
for translational initiation of chloroplast mRNAs. Plant Cell Physiol.

[945] Suzuki H., Kuroda H., Yukawa Y., Sugiura M. (2011). The downstream
atpE cistron is efficiently translated via its own cis-element in
partially overlapping atpB-atpE dicistronic mRNAs in chloroplasts. Nucleic Acids Res.

[946] Böhm C. V., Inckemann R., Burgis M., Baumann J., Brinkmann C. K., Lipinska K. E., Gilles S., Freudigmann J., Seiler V. N., Clark L. G. (2024). Chloroplast cell-free systems from
different plant species as a rapid prototyping platform. ACS Synthetic Biology.

[947] Stern D. B., Goldschmidt-Clermont M., Hanson M. R. (2010). Chloroplast
RNA metabolism. Annu Rev Plant Biol.

[948] Germain A., Hotto A. M., Barkan A., Stern D. B. (2013). RNA processing
and decay in plastids. Wiley Interdiscip Rev
RNA.

[949] Kuroda H., Maliga P. (2002). Overexpression of the clpP 5-untranslated
region in a chimeric context causes a mutant phenotype, suggesting
competition for a clpP-specific RNA maturation factor in tobacco chloroplasts. Plant Physiol.

[950] Chakrabarti S. K., Lutz K. A., Lertwiriyawong B., Svab Z., Maliga P. (2006). Expression of the cry9Aa2 B.t. gene
in tobacco chloroplasts confers resistance to potato tuber moth. Transgenic Res.

[951] Zhou F., Karcher D., Bock R. (2007). Identification
of a
plastid intercistronic expression element (IEE) facilitating the expression
of stable translatable monocistronic mRNAs from operons. Plant J.

[952] Fuentes, P. ; Zhou, F. ; Erban, A. ; Karcher, D. ; Kopka, J. ; Bock, R. A new synthetic biology approach allows transfer of an entire metabolic pathway from a medicinal plant to a biomass crop. Elife 2016, 5, 10.7554/eLife.13664. PMC4907697 27296645

[953] Lin M. T., Occhialini A., Andralojc P. J., Parry M. A., Hanson M. R. (2014). A faster Rubisco with potential to
increase photosynthesis in crops. Nature.

[954] Chen T., Hojka M., Davey P., Sun Y., Dykes G. F., Zhou F., Lawson T., Nixon P. J., Lin Y., Liu L. N. (2023). Engineering alpha-carboxysomes into plant chloroplasts
to support autotrophic photosynthesis. Nat Commun.

[955] Barkan A. (1988). Proteins encoded by a complex chloroplast
transcription
unit are each translated from both monocistronic and polycistronic
mRNAs. EMBO J.

[956] Zoschke R., Barkan A. (2015). Genome-wide analysis
of thylakoid-bound
ribosomes in maize reveals principles of cotranslational targeting
to the thylakoid membrane. Proc Natl Acad Sci
U S A.

[957] Yu Q., Tungsuchat-Huang T., Verma K., Radler M. R., Maliga P. (2020). Independent translation of ORFs in dicistronic operons,
synthetic building blocks for polycistronic chloroplast gene expression. Plant J.

[958] Macedo-Osorio K. S., Perez-Espana V. H., Garibay-Orijel C., Guzman-Zapata D., Duran-Figueroa N. V., Badillo-Corona J. A. (2018). Intercistronic
expression elements (IEE) from the chloroplast of Chlamydomonas reinhardtii
can be used for the expression of foreign genes in synthetic operons. Plant Mol Biol.

[959] Tangphatsornruang S., Birch-Machin I., Newell C. A., Gray J. C. (2011). The effect
of different 3 untranslated regions on the accumulation and stability
of transcripts of a gfp transgene in chloroplasts of transplastomic
tobacco. Plant Mol Biol.

[960] Barshad G., Marom S., Cohen T., Mishmar D. (2018). Mitochondrial
DNA Transcription and Its Regulation: An Evolutionary Perspective. Trends Genet.

[961] Basu U., Bostwick A. M., Das K., Dittenhafer-Reed K. E., Patel S. S. (2020). Structure, mechanism, and regulation of mitochondrial
DNA transcription initiation. J Biol Chem.

[962] Tan B. G., Gustafsson C. M., Falkenberg M. (2024). Mechanisms
and regulation of human mitochondrial transcription. Nat Rev Mol Cell Biol.

[963] Planchard N., Bertin P., Quadrado M., Dargel-Graffin C., Hatin I., Namy O., Mireau H. (2018). The translational
landscape
of Arabidopsis mitochondria. Nucleic Acids Res.

[964] Cahoon A. B., Qureshi A. A. (2018). Leaderless mRNAs are circularized
in Chlamydomonas reinhardtii mitochondria. Curr
Genet.

[965] Proulex, G. C. R. ; Meade, M. J. ; Manoylov, K. M. ; Cahoon, A. B. Mitochondrial mRNA Processing in the Chlorophyte Alga Pediastrum duplex and Streptophyte Alga Chara vulgaris Reveals an Evolutionary Branch in Mitochondrial mRNA Processing. Plants (Basel) 2021, 10 (3), 576. 10.3390/plants10030576 33803683 PMC8003010

[966] Herrmann J. M., Woellhaf M. W., Bonnefoy N. (2013). Control of
protein
synthesis in yeast mitochondria: the concept of translational activators. Biochim Biophys Acta.

[967] D'Souza A. R., Minczuk M. (2018). Mitochondrial transcription
and translation:
overview. Essays Biochem.

[968] Hu Z., Fan Z., Zhao Z., Chen J., Li J. (2012). Stable expression
of antibiotic-resistant gene ble from Streptoalloteichus hindustanus
in the mitochondria of Chlamydomonas reinhardtii. PLoS One.

[969] Hu Z., Zhao Z., Wu Z., Fan Z., Chen J., Wu J., Li J. (2011). Successful expression
of heterologous egfp gene in
the mitochondria of a photosynthetic eukaryote Chlamydomonas reinhardtii. Mitochondrion.

[970] Mireau H., Arnal N., Fox T. D. (2003). Expression
of Barstar
as a selectable marker in yeast mitochondria. Mol Genet Genomics.

[971] Ding M. G., Butler C. A., Saracco S. A., Fox T. D., Godard F., di Rago J. P., Trumpower B. L. (2008). Introduction
of cytochrome b mutations in Saccharomyces cerevisiae by a method
that allows selection for both functional and non-functional cytochrome
b proteins. Biochim Biophys Acta.

[972] Gruschke S., Rompler K., Hildenbeutel M., Kehrein K., Kuhl I., Bonnefoy N., Ott M. (2012). The Cbp3-Cbp6
complex coordinates cytochrome b synthesis with bc(1) complex assembly
in yeast mitochondria. J Cell Biol.

[973] Golik P., Bonnefoy N., Szczepanek T., Saint-Georges Y., Lazowska J. (2003). The Rieske FeS protein encoded and
synthesized within mitochondria complements a deficiency in the nuclear
gene. Proc Natl Acad Sci U S A.

[974] Arntzen C. (2015). Plant-made
pharmaceuticals: from 'Edible Vaccines'
to Ebola therapeutics. Plant Biotechnol J.

[975] Yao J., Weng Y., Dickey A., Wang K. Y. (2015). Plants as Factories
for Human Pharmaceuticals: Applications and Challenges. Int J Mol Sci.

[976] Capell T., Twyman R. M., Armario-Najera V., Ma J. K., Schillberg S., Christou P. (2020). Potential Applications
of Plant Biotechnology against SARS-CoV-2. Trends
Plant Sci.

[977] Shulaev, V. Plant Metabolic Engineering: Methods and Protocols; Springer, 2022.

[978] Lau W., Sattely E. S. (2015). Six enzymes from
mayapple that complete the biosynthetic
pathway to the etoposide aglycone. Science.

[979] Caputi L., Franke J., Farrow S. C., Chung K., Payne R. M. E., Nguyen T. D., Dang T. T. T., Carqueijeiro I. S. T., Koudounas K., de Bernonville T. D. (2018). Missing enzymes in the
biosynthesis of the anticancer drug vinblastine in Madagascar periwinkle. Science.

[980] Payne, R. M. E. ; Xu, D. Y. ; Foureau, E. ; Carqueijeiro, M. I. S. T. ; Oudin, A. ; de Bernonville, T. D. ; Novak, V. ; Burow, M. ; Olsen, C. E. ; Jones, D. M. ; An NPF transporter exports a central monoterpene indole alkaloid intermediate from the vacuole. Nat Plants 2017, 3 (2), 10.1038/nplants.2016.208. PMC5238941 28085153

[981] Geu-Flores F., Sherden N. H., Courdavault V., Burlat V., Glenn W. S., Wu C., Nims E., Cui Y. H., O'Connor S. E. (2012). An alternative route to cyclic terpenes
by reductive cyclization in iridoid biosynthesis. Nature.

[982] McClune C. J., Liu J. C., Wick C., De La Pena R., Lange B. M., Fordyce P. M., Sattely E. S. (2025). Discovery of FoTO1
and Taxol genes enables biosynthesis of baccatin III. Nature.

[983] Rajniak J., Barco B., Clay N. K., Sattely E. S. (2015). A new cyanogenic
metabolite in Arabidopsis required for inducible pathogen defence. Nature.

[984] Jeon J. E., Kim J. G., Fischer C. R., Mehta N., Dufour-Schroif C., Wemmer K., Mudgett M. B., Sattely E. (2020). A Pathogen-Responsive
Gene Cluster for Highly Modified Fatty Acids in Tomato. Cell.

[985] Martin L. B. B., Kikuchi S., Rejzek M., Owen C., Reed J., Orme A., Misra R. C., El-Demerdash A., Hill L., Hodgson H. (2024). Complete biosynthesis
of the potent vaccine adjuvant QS-21. Nat Chem
Biol.

[986] Kautsar S. A., Suarez Duran H. G., Blin K., Osbourn A., Medema M. H. (2017). PlantiSMASH: Automated identification, annotation and
expression analysis of plant biosynthetic gene clusters. Nucleic Acids Research.

[987] Topfer N., Fuchs L. M., Aharoni A. (2017). The PhytoClust
tool
for metabolic gene clusters discovery in plant genomes. Nucleic Acids Research.

[988] Almeida, R. D. ; Valente, G. T. Predicting metabolic pathways of plant enzymes without using sequence similarity: Models from machine learning. Plant Genome-Us 2020, 13 (3), e20043. 10.1002/tpg2.20043 PMC12806933 33217216

[989] Moore B. M., Wang P., Fan P., Leong B., Schenck C. A., Lloyd J. P., Lehti-Shiu M. D., Last R. L., Pichersky E., Shiu S. H. (2019). Robust predictions
of specialized metabolism genes through machine learning. Proc Natl Acad Sci U S A.

[990] Tian Y. S., Wang B., Peng R. H., Xu J., Li T., Fu X. Y., Xiong A. S., Gao J. J., Yao Q. H. (2019). Enhancing
carotenoid biosynthesis in rice endosperm by metabolic engineering. Plant Biotechnol J.

[991] Zuk M., Prescha A., Stryczewska M., Szopa J. (2012). Engineering Flax Plants
To Increase Their Antioxidant Capacity and Improve Oil Composition
and Stability. J Agr Food Chem.

[992] Alagoz Y., Gurkok T., Zhang B., Unver T. (2016). Manipulating
the Biosynthesis of Bioactive Compound Alkaloids for Next-Generation
Metabolic Engineering in Opium Poppy Using CRISPR-Cas 9 Genome Editing
Technology. Sci Rep.

[993] Zeng L., Zhang Q., Jiang C., Zheng Y., Zuo Y., Qin J., Liao Z., Deng H. (2021). Development of Atropa
belladonna L. plants with high-yield hyoscyamine and without its derivatives
using the CRISPR/Cas9 system. International
Journal of Molecular Sciences.

[994] Hui S., Li H., Mawia A. M., Zhou L., Cai J., Ahmad S., Lai C., Wang J., Jiao G., Xie L. (2022). Production of aromatic three-line hybrid rice using novel alleles
of BADH2. Plant biotechnology journal.

[995] Frabel S., Wagner B., Krischke M., Schmidts V., Thiele C. M., Staniek A., Warzecha H. (2018). Engineering
of new-to-nature
halogenated indigo precursors in plants. Metabolic
Engineering.

[996] Reed J., Stephenson M. J., Miettinen K., Brouwer B., Leveau A., Brett P., Goss R. J. M., Goossens A., O'Connell M. A., Osbourn A. (2017). A translational synthetic
biology platform for rapid access to gram-scale quantities of novel
drug-like molecules. Metab Eng.

[997] Lin G.-M., Warden-Rothman R., Voigt C. A. (2019). Retrosynthetic design
of metabolic pathways to chemicals not found in nature. Current Opinion in Biotechnology.

[998] Calgaro-Kozina A., Vuu K. M., Keasling J. D., Loque D., Sattely E. S., Shih P. M. (2020). Engineering Plant
Synthetic Pathways
for the Biosynthesis of Novel Antifungals. ACS
Cent Sci.

[999] Reinecke D. M., Ozga J. A., Magnus V. (1995). Effect of Halogen Substitution
of Indole-3-Acetic-Acid on Biological-Activity in Pea Fruit. Phytochemistry.

[1000] Davis, K. ; Gkotsi, D. S. ; Smith, D. R. M. ; Goss, R. J. M. ; Caputi, L. ; O'Connor, S. E. Nicotiana benthamiana as a Transient Expression Host to Produce Auxin Analogs. Frontiers in Plant Science 2020, 11, 10.3389/fpls.2020.581675. PMC7714751 33329644

[1001] Tetali S. D. (2019). Terpenes and isoprenoids: a wealth
of compounds for
global use. Planta.

[1002] Mewalal R., Rai D. K., Kainer D., Chen F., Kulheim C., Peter G. F., Tuskan G. A. (2017). Plant-Derived
Terpenes:
A Feedstock for Specialty Biofuels. Trends Biotechnol.

[1003] Lu X., Tang K., Li P. (2016). Plant Metabolic Engineering Strategies
for the Production of Pharmaceutical Terpenoids. Front Plant Sci.

[1004] Geisler K., Hughes R. K., Sainsbury F., Lomonossoff G. P., Rejzek M., Fairhurst S., Olsen C. E., Motawia M. S., Melton R. E., Hemmings A. M. (2013). Biochemical analysis of a multifunctional cytochrome P450 (CYP51)
enzyme required for synthesis of antimicrobial triterpenes in plants. Proc Natl Acad Sci U S A.

[1005] Andersen-Ranberg J., Kongstad K. T., Nielsen M. T., Jensen N. B., Pateraki I., Bach S. S., Hamberger B., Zerbe P., Staerk D., Bohlmann J. (2016). Expanding
the Landscape of Diterpene Structural Diversity through Stereochemically
Controlled Combinatorial Biosynthesis. Angew
Chem Int Ed Engl.

[1006] Runguphan W., Qu X. D., O'Connor S. E. (2010). Integrating
carbon-halogen
bond formation into medicinal plant metabolism. Nature.

[1007] Glenn W. S., Nims E., O'Connor S. E. (2011). Reengineering
a
Tryptophan Halogenase To Preferentially Chlorinate a Direct Alkaloid
Precursor. J Am Chem Soc.

[1008] Sandmann G., Romer S., Fraser P. D. (2006). Understanding
carotenoid
metabolism as a necessity for genetic engineering of crop plants. Metab Eng.

[1009] Breitenbach J., Bai C., Rivera S. M., Canela R., Capell T., Christou P., Zhu C., Sandmann G. (2014). A novel carotenoid,
4-keto-alpha-carotene, as an unexpected by-product during genetic
engineering of carotenogenesis in rice callus. Phytochemistry.

[1010] Shindo K., Hasunuma T., Asagi E., Sano A., Hotta E., Minemura N., Miyake C., Maoka T., Misawa N. (2008). 4-ketoantheraxanthin, a novel carotenoid
produced by
the combination of the bacterial enzyme beta-carotene ketolase CrtW
and endogenous carotenoid biosynthetic enzymes in higher plants. Tetrahedron Lett.

[1011] Armbruster U., Strand D. D. (2020). Regulation of chloroplast
primary
metabolism. Photosynth Res.

[1012] Hernandez M. L., Cejudo F. J. (2021). Chloroplast Lipids
Metabolism and
Function. A Redox Perspective. Front Plant Sci.

[1013] Krichevsky A., Meyers B., Vainstein A., Maliga P., Citovsky V. (2010). Autoluminescent plants. PLoS One.

[1014] Wu S. Q., Schalk M., Clark A., Miles R. B., Coates R., Chappell J. (2006). Redirection of cytosolic or plastidic
isoprenoid precursors elevates terpene production in plants. Nature Biotechnology.

[1015] Harada H., Maoka T., Osawa A., Hattan J., Kanamoto H., Shindo K., Otomatsu T., Misawa N. (2014). Construction
of transplastomic lettuce (Lactuca sativa) dominantly producing astaxanthin
fatty acid esters and detailed chemical analysis of generated carotenoids. Transgenic Res.

[1016] Hasunuma T., Miyazawa S.-I., Yoshimura S., Shinzaki Y., Tomizawa K.-I., Shindo K., Choi S.-K., Misawa N., Miyake C. (2008). Biosynthesis of astaxanthin in tobacco
leaves by transplastomic engineering. The Plant
Journal.

[1017] Lossl A., Bohmert K., Harloff H., Eibl C., Muhlbauer S., Koop H. U. (2005). Inducible trans-activation
of plastid
transgenes: expression of the R. eutropha phb operon in transplastomic
tobacco. Plant Cell Physiol.

[1018] Agrawal S., Karcher D., Ruf S., Erban A., Hertle A. P., Kopka J., Bock R. (2022). Riboswitch-mediated
inducible expression of an astaxanthin biosynthetic operon in plastids. Plant Physiol.

[1019] Enfissi E. M. A., Fraser P. D., Lois L. M., Boronat A., Schuch W., Bramley P. M. (2005). Metabolic engineering
of the mevalonate
and non-mevalonate isopentenyl diphosphate-forming pathways for the
production of health-promoting isoprenoids in tomato. Plant Biotechnol J.

[1020] Li J., Mutanda I., Wang K., Yang L., Wang J., Wang Y. (2019). Chloroplastic metabolic
engineering coupled with isoprenoid pool
enhancement for committed taxanes biosynthesis in Nicotiana benthamiana. Nat Commun.

[1021] Fuentes P., Zhou F., Erban A., Karcher D., Kopka J., Bock R. (2016). A new synthetic biology approach
allows transfer of an entire metabolic pathway from a medicinal plant
to a biomass crop. eLife.

[1022] Kumar S., Hahn F. M., Baidoo E., Kahlon T. S., Wood D. F., McMahan C. M., Cornish K., Keasling J. D., Daniell H., Whalen M. C. (2012). Remodeling the isoprenoid
pathway
in tobacco by expressing the cytoplasmic mevalonate pathway in chloroplasts. Metab Eng.

[1023] Gnanasekaran T., Karcher D., Nielsen A. Z., Martens H. J., Ruf S., Kroop X., Olsen C. E., Motawie M. S., Pribil M., Moller B. L. (2016). Transfer of the cytochrome P450-dependent dhurrin
pathway from Sorghum bicolor into Nicotiana tabacum chloroplasts for
light-driven synthesis. J Exp Bot.

[1024] Nielsen A. Z., Ziersen B., Jensen K., Lassen L. M., Olsen C. E., Moller B. L., Jensen P. E. (2013). Redirecting Photosynthetic
Reducing Power toward Bioactive Natural Product Synthesis. ACS Synth. Biol..

[1025] Jensen K., Jensen P. E., Moller B. L. (2011). Light-Driven
Cytochrome
P450 Hydroxylations. Acs Chem Biol.

[1026] Mellor S. B., Nielsen A. Z., Burow M., Motawia M. S., Jakubauskas D., Moller B. L., Jensen P. E. (2016). Fusion
of Ferredoxin
and Cytochrome P450 Enables Direct Light-Driven Biosynthesis. Acs Chem Biol.

[1027] de Jesus M. P. R. H., Nielsen A. Z., Mellor S. B., Matthes A., Burow M., Robinson C., Jensen P. E. (2017). Tat proteins
as
novel thylakoid membrane anchors organize a biosynthetic pathway in
chloroplasts and increase product yield 5-fold. Metab. Eng..

[1028] Engels B., Dahm P., Jennewein S. (2008). Metabolic
engineering of taxadiene biosynthesis in yeast as a first step towards
Taxol (Paclitaxel) production. Metab Eng.

[1029] Nowrouzi B., Li R. A., Walls L. E., d'Espaux L., Malci K., Liang L., Jonguitud-Borrego N., Lerma-Escalera A. I., Morones-Ramirez J. R., Keasling J. D. (2020). Enhanced
production of taxadiene in Saccharomyces cerevisiae. Microb Cell Fact.

[1030] Liu J. C., De La Pena R., Tocol C., Sattely E. S. (2024). Reconstitution
of early paclitaxel biosynthetic network. Nat
Commun.

[1031] McClune C. J., Liu J. C.-T., Wick C., De La Peña R., Lange B. M., Fordyce P. M., Sattely E. S. (2024). Multiplexed perturbation
of yew reveals cryptic proteins that enable a total biosynthesis of
baccatin III and Taxol precursors. bioRxiv.

[1032] Apel W., Bock R. (2009). Enhancement of carotenoid
biosynthesis
in transplastomic tomatoes by induced lycopene-to-provitamin A conversion. Plant Physiol.

[1033] Besumbes O., Sauret-Gueto S., Phillips M. A., Imperial S., Rodriguez-Concepcion M., Boronat A. (2004). Metabolic engineering of isoprenoid
biosynthesis in Arabidopsis for the production of taxadiene, the first
committed precursor of Taxol. Biotechnol Bioeng.

[1034] Jensen P. E., Scharff L. B. (2019). Engineering of plastids to optimize
the production of high-value metabolites and proteins. Curr Opin Biotechnol.

[1035] Gallage N. J., Jorgensen K., Janfelt C., Nielsen A. J. Z., Naake T., Dunski E., Dalsten L., Grisoni M., Moller B. L. (2018). The Intracellular Localization of the Vanillin Biosynthetic
Machinery in Pods of Vanilla planifolia. Plant
and Cell Physiology.

[1036] Brillouet J. M., Verdeil J. L., Odoux E., Lartaud M., Grisoni M., Conejero G. (2014). Phenol homeostasis is ensured in
vanilla fruit by storage under solid form in a new chloroplast-derived
organelle, the phenyloplast. Journal of Experimental
Botany.

[1037] Yuan H., Owsiany K., Sheeja T. E., Zhou X. J., Rodriguez C., Li Y. X., Welsch R., Chayut N., Yang Y., Thannhauser T. W. (2015). A Single Amino Acid
Substitution in an ORANGE Protein Promotes Carotenoid Overaccumulation
in Arabidopsis. Plant Physiology.

[1038] Yazdani M., Sun Z., Yuan H., Zeng S., Thannhauser T. W., Vrebalov J., Ma Q., Xu Y., Fei Z., Van Eck J. (2019). Ectopic expression of
ORANGE promotes
carotenoid accumulation and fruit development in tomato. Plant Biotechnol J.

[1039] Lopez A. B., Van Eck J., Conlin B. J., Paolillo D. J., O'Neill J., Li L. (2008). Effect of the cauliflower
Or transgene
on carotenoid accumulation and chromoplast formation in transgenic
potato tubers. Journal of Experimental Botany.

[1040] Majer E., Llorente B., Rodríguez-Concepción M., Daròs J.-A. (2017). Rewiring carotenoid biosynthesis in plants using a
viral vector. Scientific Reports.

[1041] Lv X., Wang F., Zhou P., Ye L., Xie W., Xu H., Yu H. (2016). Dual regulation of
cytoplasmic and mitochondrial acetyl-CoA
utilization for improved isoprene production in Saccharomyces cerevisiae. Nat Commun.

[1042] Yee D. A., DeNicola A. B., Billingsley J. M., Creso J. G., Subrahmanyam V., Tang Y. (2019). Engineered mitochondrial
production of monoterpenes in Saccharomyces cerevisiae. Metab Eng.

[1043] Avalos J. L., Fink G. R., Stephanopoulos G. (2013). Compartmentalization
of metabolic pathways in yeast mitochondria improves the production
of branched-chain alcohols. Nat Biotechnol.

[1044] Chen B., Foo J. L., Ling H., Chang M. W. (2020). Mechanism-Driven
Metabolic Engineering for Bio-Based Production of Free R-Lipoic Acid
in Saccharomyces cerevisiae Mitochondria. Front
Bioeng Biotechnol.

[1045] Ivleva N. B., Groat J., Staub J. M., Stephens M. (2016). Expression
of Active Subunit of Nitrogenase via Integration into Plant Organelle
Genome. PLoS One.

[1046] Flechsler, J. ; Heimerl, T. ; Huber, H. ; Rachel, R. ; Berg, I. A. Functional compartmentalization and metabolic separation in a prokaryotic cell. Proc Natl Acad Sci U S A 2021, 118 (25), 10.1073/pnas.2022114118. PMC8237620 34161262

[1047] Yeates T. O., Crowley C. S., Tanaka S. (2010). Bacterial
microcompartment
organelles: protein shell structure and evolution. Annu Rev Biophys.

[1048] Kirst H., Kerfeld C. A. (2019). Bacterial microcompartments:
catalysis-enhancing
metabolic modules for next generation metabolic and biomedical engineering. BMC Biol.

[1049] Chowdhury C., Chun S., Pang A., Sawaya M. R., Sinha S., Yeates T. O., Bobik T. A. (2015). Selective molecular
transport through the protein shell of a bacterial microcompartment
organelle. P Natl Acad Sci USA.

[1050] Sutter M., Melnicki M. R., Schulz F., Woyke T., Kerfeld C. A. (2021). A catalog
of the diversity and ubiquity of bacterial
microcompartments. Nat Commun.

[1051] Grewal P. S., Samson J. A., Baker J. J., Choi B., Dueber J. E. (2021). Peroxisome compartmentalization of
a toxic enzyme improves
alkaloid production. Nat Chem Biol.

[1052] Cheah L. C., Stark T., Adamson L. S. R., Abidin R. S., Lau Y. H., Sainsbury F., Vickers C. E. (2021). Artificial Self-assembling
Nanocompartment for Organizing Metabolic Pathways in Yeast. ACS Synth Biol.

[1053] Lau Y. H., Giessen T. W., Altenburg W. J., Silver P. A. (2018). Prokaryotic nanocompartments form synthetic organelles
in a eukaryote. Nat Commun.

[1054] Whitaker W. R., Davis S. A., Arkin A. P., Dueber J. E. (2012). Engineering
robust control of two-component system phosphotransfer using modular
scaffolds. Proc Natl Acad Sci U S A.

[1055] Dueber J. E., Wu G. C., Malmirchegini G. R., Moon T. S., Petzold C. J., Ullal A. V., Prather K. L., Keasling J. D. (2009). Synthetic protein
scaffolds provide modular control
over metabolic flux. Nat Biotechnol.

[1056] Chapman K. D., Dyer J. M., Mullen R. T. (2012). Biogenesis
and functions
of lipid droplets in plants: Thematic Review Series: Lipid Droplet
Synthesis and Metabolism: from Yeast to Man. J Lipid Res.

[1057] Zhao C., Kim Y., Zeng Y., Li M., Wang X., Hu C., Gorman C., Dai S. Y., Ding S. Y., Yuan J. S. (2018). Co-Compartmentation of Terpene Biosynthesis
and Storage via Synthetic Droplet. ACS Synth
Biol.

[1058] Portnoy V. A., Bezdan D., Zengler K. (2011). Adaptive laboratory
evolution-harnessing the power of biology for metabolic engineering. Curr Opin Biotechnol.

[1059] Chen G., Xu Y., Siloto R. M. P., Caldo K. M. P., Vanhercke T., Tahchy A. E., Niesner N., Chen Y., Mietkiewska E., Weselake R. J. (2017). High-performance
variants of plant
diacylglycerol acyltransferase 1 generated by directed evolution provide
insights into structure function. Plant J.

[1060] Siloto R. M., Truksa M., Brownfield D., Good A. G., Weselake R. J. (2009). Directed
evolution of acyl-CoA:diacylglycerol
acyltransferase: development and characterization of Brassica napus
DGAT1 mutagenized libraries. Plant Physiol Biochem.

[1061] Castle L. A., Siehl D. L., Gorton R., Patten P. A., Chen Y. H., Bertain S., Cho H. J., Duck N., Wong J., Liu D. (2004). Discovery and directed
evolution of a glyphosate tolerance gene. Science.

[1062] Wang H., Liu B., Lei P., Zhu J., Chen L., He Q., He J. (2022). Improving the herbicide
resistance of 4-hydroxyphenylpyruvate dioxygenase SpHPPD by directed
evolution. Enzyme Microb Technol.

[1063] Chen L., Liu R., Tan Q., Luo H., Chen Y., Jin Y., Zheng Z., Zhang B., Guo D. (2023). Improving the Herbicide Resistance of Rice 4-Hydroxyphenylpyruvate
Dioxygenase by DNA Shuffling Basis-Directed Evolution. J Agric Food Chem.

[1064] Siehl D. L., Tao Y., Albert H., Dong Y., Heckert M., Madrigal A., Lincoln-Cabatu B., Lu J., Fenwick T., Bermudez E. (2014). Broad 4-hydroxyphenylpyruvate
dioxygenase inhibitor herbicide tolerance in soybean with an optimized
enzyme and expression cassette. Plant Physiol.

[1065] Dixon D. P., McEwen A. G., Lapthorn A. J., Edwards R. (2003). Forced evolution
of a herbicide detoxifying glutathione transferase. J Biol Chem.

[1066] Ioannou, E. ; Papageorgiou, A. C. ; Labrou, N. E. Directed Evolution of Phi Class Glutathione Transferases Involved in Multiple-Herbicide Resistance of Grass Weeds and Crops. Int J Mol Sci 2022, 23 (13), 7469 10.3390/ijms23137469. 35806486 PMC9267659

[1067] Garcia-Garcia J. D., Van Gelder K., Joshi J., Bathe U., Leong B. J., Bruner S. D., Liu C. C., Hanson A. D. (2022). Using continuous
directed evolution to improve enzymes for plant applications. Plant Physiol.

[1068] Shakhova E. S., Karataeva T. A., Markina N. M., Mitiouchkina T., Palkina K. A., Perfilov M. M., Wood M. G., Hoang T. T., Hall M. P., Fakhranurova L. I. (2024). An improved pathway
for autonomous bioluminescence imaging in eukaryotes. Nat Methods.

[1069] Liu D., Evans T., Zhang F. (2015). Applications and advances
of metabolite
biosensors for metabolic engineering. Metab
Eng.

[1070] Mao Y., Huang C., Zhou X., Han R., Deng Y., Zhou S. (2023). Genetically Encoded Biosensor Engineering
for Application in Directed
Evolution. J Microbiol Biotechnol.

[1071] Andon J. S., Lee B., Wang T. (2022). Enzyme directed evolution
using genetically encodable biosensors. Org
Biomol Chem.

[1072] Esvelt K. M., Carlson J. C., Liu D. R. (2011). A system for the
continuous directed evolution of biomolecules. Nature.

[1073] Craveiro K. I., Gomes Junior J. E., Silva M. C., Macedo L. L., Lucena W. A., Silva M. S., de Souza Junior J. D., Oliveira G. R., de Magalhaes M. T., Santiago A. D. (2010). Variant
Cry1Ia toxins generated by DNA shuffling are active against sugarcane
giant borer. J Biotechnol.

[1074] Dominguez-Flores T., Romero-Bosquet M. D., Gantiva-Diaz D. M., Luque-Navas M. J., Berry C., Osuna A., Vilchez S. (2017). Using phage
display technology to obtain Crybodies active against non-target insects. Sci Rep.

[1075] Badran A. H., Guzov V. M., Huai Q., Kemp M. M., Vishwanath P., Kain W., Nance A. M., Evdokimov A., Moshiri F., Turner K. H. (2016). Continuous evolution
of Bacillus thuringiensis toxins overcomes insect resistance. Nature.

[1076] Zhang A., Shan T., Sun Y., Chen Z., Hu J., Hu Z., Ming Z., Zhu Z., Li X., He J. (2023). Directed evolution rice
genes with randomly multiplexed
sgRNAs assembly of base editors. Plant Biotechnol
J.

[1077] Beadle C., Long S. (1985). Photosynthesis—is it limiting
to biomass production?. Biomass.

[1078] Ort D. R., Merchant S. S., Alric J., Barkan A., Blankenship R. E., Bock R., Croce R., Hanson M. R., Hibberd J. M., Long S. P. (2015). Redesigning
photosynthesis
to sustainably meet global food and bioenergy demand. Proc Natl Acad Sci U S A.

[1079] Zhu X. G., Ort D. R., Parry M. A. J., von
Caemmerer S. (2020). A wish list for synthetic biology in photosynthesis
research. J Exp Bot.

[1080] Kubis A., Bar-Even A. (2019). Synthetic biology approaches
for
improving photosynthesis. J Exp Bot.

[1081] Bar-Even A. (2018). Daring metabolic
designs for enhanced plant carbon
fixation. Plant Sci.

[1082] Blankenship R. E., Tiede D. M., Barber J., Brudvig G. W., Fleming G., Ghirardi M., Gunner M. R., Junge W., Kramer D. M., Melis A. (2011). Comparing
photosynthetic
and photovoltaic efficiencies and recognizing the potential for improvement. Science.

[1083] Langdale J. A. (2011). C4 cycles: past, present, and future
research on C4
photosynthesis. Plant Cell.

[1084] Ermakova M., Arrivault S., Giuliani R., Danila F., Alonso-Cantabrana H., Vlad D., Ishihara H., Feil R., Guenther M., Borghi G. L. (2021). Installation of C(4)
photosynthetic pathway enzymes in rice using a single construct. Plant Biotechnol J.

[1085] South, P. F. ; Cavanagh, A. P. ; Liu, H. W. ; Ort, D. R. Synthetic glycolate metabolism pathways stimulate crop growth and productivity in the field. Science 2019, 363 (6422), 10.1126/science.aat9077. PMC7745124 30606819

[1086] Bond W. J., Woodward F. I., Midgley G. F. (2005). The global
distribution
of ecosystems in a world without fire. New Phytol.

[1087] Osborne C. P., Beerling D. J. (2006). Nature's green revolution: the remarkable
evolutionary rise of C4 plants. Philos Trans
R Soc Lond B Biol Sci.

[1088] Kellogg E. A. (2013). C4 photosynthesis. Curr Biol.

[1089] Gowik U., Westhoff P. (2011). The path from C3 to
C4 photosynthesis. Plant Physiol.

[1090] Leegood R. C. (2002). C(4) photosynthesis: principles of
CO(2) concentration
and prospects for its introduction into C(3) plants. J Exp Bot.

[1091] Karp A., Richter G. M. (2011). Meeting the challenge
of food and
energy security. J Exp Bot.

[1092] Schuler M. L., Mantegazza O., Weber A. P. (2016). Engineering C4 photosynthesis
into C3 chassis in the synthetic biology age. Plant J.

[1093] Liu Z., Cheng J. (2024). C(4) rice engineering, beyond installing a C(4) cycle. Plant Physiol Biochem.

[1094] Roell, M. S. ; Schada von Borzykowski, L. ; Westhoff, P. ; Plett, A. ; Paczia, N. ; Claus, P. ; Urte, S. ; Erb, T. J. ; Weber, A. P. M. A synthetic C4 shuttle via the beta-hydroxyaspartate cycle in C3 plants. Proc Natl Acad Sci U S A 2021, 118 (21), 10.1073/pnas.2022307118. PMC8166194 34001608

[1095] Yadav S., Mishra A. (2020). Ectopic expression
of C(4) photosynthetic
pathway genes improves carbon assimilation and alleviate stress tolerance
for future climate change. Physiol Mol Biol
Plants.

[1096] Ermakova M., Danila F. R., Furbank R. T., von Caemmerer S. (2020). On the road
to C(4) rice: advances and perspectives. Plant
J.

[1097] Reeves G., Grange-Guermente M. J., Hibberd J. M. (2017). Regulatory gateways
for cell-specific gene expression in C4 leaves with Kranz anatomy. J Exp Bot.

[1098] Wiludda C., Schulze S., Gowik U., Engelmann S., Koczor M., Streubel M., Bauwe H., Westhoff P. (2012). Regulation
of the photorespiratory GLDPA gene in C(4) flaveria: an intricate
interplay of transcriptional and posttranscriptional processes. Plant Cell.

[1099] Dickinson P. J., Knerova J., Szecowka M., Stevenson S. R., Burgess S. J., Mulvey H., Bagman A. M., Gaudinier A., Brady S. M., Hibberd J. M. (2020). A bipartite transcription factor
module controlling expression in the bundle sheath of Arabidopsis
thaliana. Nat Plants.

[1100] Wang P., Khoshravesh R., Karki S., Tapia R., Balahadia C. P., Bandyopadhyay A., Quick W. P., Furbank R., Sage T. L., Langdale J. A. (2017). Re-creation of a Key Step in the
Evolutionary Switch from C(3) to C(4) Leaf Anatomy. Curr Biol.

[1101] Lung S.-C., Yanagisawa M., Chuong S. D. X. (2012). Recent progress
in the single-cell C4 photosynthesis in terrestrial plants. Frontiers in Biology.

[1102] Parry M. A., Andralojc P. J., Mitchell R. A., Madgwick P. J., Keys A. J. (2003). Manipulation of
Rubisco: the amount, activity, function
and regulation. J Exp Bot.

[1103] Ishikawa C., Hatanaka T., Misoo S., Miyake C., Fukayama H. (2011). Functional incorporation of sorghum
small subunit increases
the catalytic turnover rate of Rubisco in transgenic rice. Plant Physiol.

[1104] Matsumura H., Shiomi K., Yamamoto A., Taketani Y., Kobayashi N., Yoshizawa T., Tanaka S. I., Yoshikawa H., Endo M., Fukayama H. (2020). Hybrid Rubisco
with Complete Replacement
of Rice Rubisco Small Subunits by Sorghum Counterparts Confers C4-Plant-like
High Catalytic Activity. Mol Plant.

[1105] Prywes N., Phillips N. R., Tuck O. T., Valentin-Alvarado L. E., Savage D. F. (2023). Rubisco Function, Evolution, and
Engineering. Annu Rev Biochem.

[1106] Parikh M. R., Greene D. N., Woods K. K., Matsumura I. (2006). Directed evolution
of RuBisCO hypermorphs through genetic selection in engineered E.coli. Protein Eng Des Sel.

[1107] Zhou, Y. ; Whitney, S. Directed Evolution of an Improved Rubisco; In Vitro Analyses to Decipher Fact from Fiction. Int J Mol Sci 2019, 20 (20), 5019 10.3390/ijms20205019. 31658746 PMC6834295

[1108] Wilson R. H., Martin-Avila E., Conlan C., Whitney S. M. (2018). An improved
Escherichia coli screen for Rubisco identifies a protein-protein interface
that can enhance CO(2)-fixation kinetics. J
Biol Chem.

[1109] Prywes N., Phillips N. R., Oltrogge L. M., Lindner S., Taylor-Kearney L. J., Tsai Y. C., de Pins B., Cowan A. E., Chang H. A., Wang R. Z. (2025). A map of the rubisco
biochemical landscape. Nature.

[1110] Mueller-Cajar O., Whitney S. M. (2008). Evolving improved Synechococcus Rubisco
functional expression in Escherichia coli. Biochem
J.

[1111] Mueller-Cajar O., Morell M., Whitney S. M. (2007). Directed evolution
of rubisco in Escherichia coli reveals a specificity-determining hydrogen
bond in the form II enzyme. Biochemistry.

[1112] Greene D. N., Whitney S. M., Matsumura I. (2007). Artificially
evolved Synechococcus PCC6301 Rubisco variants exhibit improvements
in folding and catalytic efficiency. Biochem
J.

[1113] Wilson R. H., Alonso H., Whitney S. M. (2016). Evolving Methanococcoides
burtonii archaeal Rubisco for improved photosynthesis and plant growth. Sci Rep.

[1114] Aigner H., Wilson R. H., Bracher A., Calisse L., Bhat J. Y., Hartl F. U., Hayer-Hartl M. (2017). Plant RuBisCo
assembly in E. coli with five chloroplast chaperones including BSD2. Science.

[1115] Yeates T. O., Wheatley N. M. (2017). Putting the RuBisCO
pieces together. Science.

[1116] Kurek I., Chang T. K., Bertain S. M., Madrigal A., Liu L., Lassner M. W., Zhu G. H. (2007). Enhanced
thermostability of Arabidopsis
Rubisco activase improves photosynthesis and growth rates under moderate
heat stress. Plant Cell.

[1117] Huang F., Kong W. W., Sun Y., Chen T., Dykes G. F., Jiang Y. L., Liu L. N. (2020). Rubisco
accumulation
factor 1 (Raf1) plays essential roles in mediating Rubisco assembly
and carboxysome biogenesis. Proc Natl Acad Sci
U S A.

[1118] Whitney S. M., Birch R., Kelso C., Beck J. L., Kapralov M. V. (2015). Improving recombinant Rubisco biogenesis, plant photosynthesis
and growth by coexpressing its ancillary RAF1 chaperone. Proc Natl Acad Sci U S A.

[1119] Salesse-Smith C. E., Sharwood R. E., Busch F. A., Kromdijk J., Bardal V., Stern D. B. (2018). Overexpression of
Rubisco subunits
with RAF1 increases Rubisco content in maize. Nat Plants.

[1120] Long B. M., Hee W. Y., Sharwood R. E., Rae B. D., Kaines S., Lim Y. L., Nguyen N. D., Massey B., Bala S., von Caemmerer S. (2018). Carboxysome encapsulation
of the CO2-fixing enzyme Rubisco in tobacco chloroplasts. Nat Commun.

[1121] Wang L., Jonikas M. C. (2020). The pyrenoid. Curr Biol.

[1122] Findinier J., Grossman A. R. (2023). One step further toward a crop CO2-concentrating
mechanism. J Exp Bot.

[1123] Mackinder L. C. M., Chen C., Leib R. D., Patena W., Blum S. R., Rodman M., Ramundo S., Adams C. M., Jonikas M. C. (2017). A Spatial Interactome Reveals the
Protein Organization
of the Algal CO2-Concentrating Mechanism. Cell.

[1124] Freeman Rosenzweig E. S., Xu B., Kuhn Cuellar L., Martinez-Sanchez A., Schaffer M., Strauss M., Cartwright H. N., Ronceray P., Plitzko J. M., Forster F. (2017). The Eukaryotic
CO2-Concentrating Organelle Is Liquid-like and Exhibits Dynamic Reorganization. Cell.

[1125] Itakura A. K., Chan K. X., Atkinson N., Pallesen L., Wang L., Reeves G., Patena W., Caspari O., Roth R., Goodenough U. (2019). A Rubisco-binding protein
is required for normal pyrenoid number and starch sheath morphology
in Chlamydomonas reinhardtii. Proc Natl Acad
Sci U S A.

[1126] Mackinder L. C., Meyer M. T., Mettler-Altmann T., Chen V. K., Mitchell M. C., Caspari O., Freeman
Rosenzweig E. S., Pallesen L., Reeves G., Itakura A. (2016). A repeat protein links Rubisco to form the eukaryotic carbon-concentrating
organelle. Proc Natl Acad Sci U S A.

[1127] Hennacy J. H., Jonikas M. C. (2020). Prospects for Engineering Biophysical
CO2 Concentrating Mechanisms into Land Plants to Enhance Yields. Annu Rev Plant Biol.

[1128] Atkinson N., Feike D., Mackinder L. C., Meyer M. T., Griffiths H., Jonikas M. C., Smith A. M., McCormick A. J. (2016). Introducing an algal carbon-concentrating mechanism
into higher plants: location and incorporation of key components. Plant Biotechnol J.

[1129] Atkinson N., Mao Y., Chan K., McCormick A. (2020). Condensation
of Rubisco into a proto-pyrenoid in higher plant chloroplasts. Nat Commun..

[1130] Hennacy J. H., Atkinson N., Kayser-Browne A., Ergun S. L., Franklin E., Wang L., Eicke S., Kazachkova Y., Kafri M., Fauser F. (2024). SAGA1 and MITH1 produce
matrix-traversing membranes in the CO2-fixing pyrenoid. Nature Plants.

[1131] Kebeish R., Niessen M., Thiruveedhi K., Bari R., Hirsch H. J., Rosenkranz R., Stabler N., Schonfeld B., Kreuzaler F., Peterhansel C. (2007). Chloroplastic photorespiratory bypass increases photosynthesis
and biomass production in Arabidopsis thaliana. Nat Biotechnol.

[1132] Maier A., Fahnenstich H., von Caemmerer S., Engqvist M. K., Weber A. P., Flugge U. I., Maurino V. G. (2012). Transgenic
Introduction of a Glycolate Oxidative Cycle into A. thaliana Chloroplasts
Leads to Growth Improvement. Front Plant Sci.

[1133] Wang L. M., Shen B. R., Li B. D., Zhang C. L., Lin M., Tong P. P., Cui L. L., Zhang Z. S., Peng X. X. (2020). A Synthetic
Photorespiratory Shortcut Enhances Photosynthesis to Boost Biomass
and Grain Yield in Rice. Mol Plant.

[1134] Wurtzel E. T., Vickers C. E., Hanson A. D., Millar A. H., Cooper M., Voss-Fels K. P., Nikel P. I., Erb T. J. (2019). Revolutionizing
agriculture with synthetic biology. Nat Plants.

[1135] Schwander T., Schada von Borzyskowski L., Burgener S., Cortina N. S., Erb T. J. (2016). A synthetic pathway for the fixation
of carbon dioxide in vitro. Science.

[1136] Bar-Even A., Noor E., Lewis N. E., Milo R. (2010). Design and
analysis of synthetic carbon fixation pathways. Proc Natl Acad Sci U S A.

[1137] Miller T. E., Beneyton T., Schwander T., Diehl C., Girault M., McLean R., Chotel T., Claus P., Cortina N. S., Baret J. C. (2020). Light-powered
CO2 fixation in a chloroplast mimic with natural and synthetic parts. Science.

[1138] Xiong J., Hu F., Ren J., Huang Y., Liu C., Wang K. (2023). Synthetic apomixis:
the beginning of a new era. Curr Opin Biotechnol.

[1139] Underwood C. J., Mercier R. (2022). Engineering apomixis:
clonal seeds
approaching the fields. Annual review of plant
biology.

[1140] d'Erfurth I., Jolivet S., Froger N., Catrice O., Novatchkova M., Mercier R. (2009). Turning meiosis into
mitosis. PLoS biology.

[1141] Mieulet D., Jolivet S., Rivard M., Cromer L., Vernet A., Mayonove P., Pereira L., Droc G., Courtois B., Guiderdoni E. (2016). Turning rice
meiosis into mitosis. Cell Research.

[1142] Liang R., Gao C. (2024). Creating one-line hybrid crops by
synthetic apomixis. Mol Plant.

[1143] Ravi M., Chan S. W. (2010). Haploid plants produced
by centromere-mediated
genome elimination. Nature.

[1144] Marimuthu M. P., Jolivet S., Ravi M., Pereira L., Davda J. N., Cromer L., Wang L., Nogué F., Chan S. W., Siddiqi I. (2011). Synthetic clonal reproduction
through
seeds. Science.

[1145] Wang C., Liu Q., Shen Y., Hua Y., Wang J., Lin J., Wu M., Sun T., Cheng Z., Mercier R. (2019). Clonal seeds from hybrid
rice by simultaneous genome engineering of meiosis and fertilization
genes. Nat Biotechnol.

[1146] Yao L., Zhang Y., Liu C., Liu Y., Wang Y., Liang D., Liu J., Sahoo G., Kelliher T. (2018). OsMATL mutation
induces haploid seed formation in indica rice. Nat Plants.

[1147] Khanday I., Skinner D., Yang B., Mercier R., Sundaresan V. (2019). A male-expressed rice embryogenic trigger redirected
for asexual propagation through seeds. Nature.

[1148] Song M., Wang W., Ji C., Li S., Liu W., Hu X., Feng A., Ruan S., Du S., Wang H. (2024). Simultaneous production of high-frequency synthetic
apomixis with high fertility and improved agronomic traits in hybrid
rice. Mol Plant.

[1149] Underwood C. J., Vijverberg K., Rigola D., Okamoto S., Oplaat C., Camp R. H. O. d., Radoeva T., Schauer S. E., Fierens J., Jansen K. (2022). A PARTHENOGENESIS
allele from apomictic
dandelion can induce egg cell division without fertilization in lettuce. Nature Genetics.

[1150] Vernet A., Meynard D., Lian Q., Mieulet D., Gibert O., Bissah M., Rivallan R., Autran D., Leblanc O., Meunier A. C. (2022). High-frequency
synthetic
apomixis in hybrid rice. Nat Commun.

[1151] Wei X., Liu C., Chen X., Lu H., Wang J., Yang S., Wang K. (2023). Synthetic apomixis
with normal hybrid
rice seed production. Mol Plant.

[1152] Sever M. (2022). Genetic Approaches to Weed Control. Crops &
Soils.

[1153] Legros M., Marshall J. M., Macfadyen S., Hayes K. R., Sheppard A., Barrett L. G. (2021). Gene drive strategies
of pest control in agricultural systems: Challenges and opportunities. Evol Appl.

[1154] Brooks, J. E. ; Fiedler, L. A. ; Mejia, D. . Vertebrate pests: damage on stored foods 1999.

[1155] Leonard S. P., Powell J. E., Perutka J., Geng P., Heckmann L. C., Horak R. D., Davies B. W., Ellington A. D., Barrick J. E., Moran N. A. (2020). Engineered symbionts activate honey
bee immunity and limit pathogens. Science.

[1156] Jin L., Walker A. S., Fu G., Harvey-Samuel T., Dafa'alla T., Miles A., Marubbi T., Granville D., Humphrey-Jones N., O'Connell S. (2013). Engineered female-specific
lethality for control of pest Lepidoptera. ACS
Synth Biol.

[1157] Yadav A. K., Butler C., Yamamoto A., Patil A. A., Lloyd A. L., Scott M. J. (2023). CRISPR/Cas9-based split homing gene
drive targeting doublesex for population suppression of the global
fruit pest Drosophila suzukii. Proc Natl Acad
Sci U S A.

[1158] Oberhofer G., Johnson M. L., Ivy T., Antoshechkin I., Hay B. A. (2024). Cleave and Rescue gamete killers create conditions
for gene drive in plants. Nat Plants.

[1159] Motta E. V. S., Powell J. E., Leonard S. P., Moran N. A. (2022). Prospects
for probiotics in social bees. Philos Trans
R Soc Lond B Biol Sci.

[1160] Dosch C., Manigk A., Streicher T., Tehel A., Paxton R. J., Tragust S. (2021). The gut microbiota
can provide viral tolerance in the honey bee. Microorganisms.

[1161] Qadri M., Short S., Gast K., Hernandez J., Wong A. C.-N. (2020). Microbiome innovation in agriculture: development of
microbial based tools for insect pest management. Frontiers in Sustainable Food Systems.

[1162] Attia, S. ; Renoz, F. ; Pons, I. ; Louâpre, P. ; Foray, V. ; Piedra, J.-M. ; Sanané, I. ; Le Goff, G. ; Lognay, G. ; Hance, T. The aphid facultative symbiont Serratia symbiotica influences the foraging behaviors and the life-history traits of the parasitoid Aphidius ervi. Entomologia Generalis 2022, 42 (1), 21. 10.1127/entomologia/2021/1274

[1163] Somerville, J. ; Zhou, L. ; Raymond, B. Aseptic Rearing and Infection with Gut Bacteria Improve the Fitness of Transgenic Diamondback Moth, Plutella xylostella. Insects 2019, 10 (4), 89 10.3390/insects10040089 . 30925791 PMC6523322

[1164] Whitten, M. ; Dyson, P. Gene silencing in non-model insects: Overcoming hurdles using symbiotic bacteria for trauma-free sustainable delivery of RNA interference: Sustained RNA interference in insects mediated by symbiotic bacteria: Applications as a genetic tool and as a biocide. Bioessays 2017, 39 (3), 10.1002/bies.201600247. 28234404

[1165] Whitten M. M. A., Xue Q., Taning C. N. T., James R., Smagghe G., Del Sol R., Hitchings M., Dyson P. (2023). A narrow host-range and lack of persistence in two non-target insect
species of a bacterial symbiont exploited to deliver insecticidal
RNAi in Western Flower Thrips. Front Insect
Sci.

[1166] Elston, K. M. ; Perreau, J. ; Maeda, G. P. ; Moran, N. A. ; Barrick, J. E. Engineering a Culturable Serratia symbiotica Strain for Aphid Paratransgenesis. Appl Environ Microbiol 2021, 87 (4), 10.1128/AEM.02245-20. PMC7851701 33277267

[1167] Ricigliano V. A., Fine J. D., Nicklisch S. C. T. (2025). Harnessing
biotechnology for bee pollinator health. Trends
Biotechnol.

[1168] Elston K. M., Leonard S. P., Geng P., Bialik S. B., Robinson E., Barrick J. E. (2022). Engineering insects
from the endosymbiont
out. Trends Microbiol.

[1169] Lariviere P. J., Leonard S. P., Horak R. D., Powell J. E., Barrick J. E. (2023). Honey bee functional genomics using
symbiont-mediated
RNAi. Nat Protoc.

[1170] Leonard S. P., Perutka J., Powell J. E., Geng P., Richhart D. D., Byrom M., Kar S., Davies B. W., Ellington A. D., Moran N. A. (2018). Genetic
Engineering
of Bee Gut Microbiome Bacteria with a Toolkit for Modular Assembly
of Broad-Host-Range Plasmids. ACS Synth Biol.

[1171] Machado R. A., Thönen L., Arce C. C., Theepan V., Prada F., Wüthrich D., Robert C. A., Vogiatzaki E., Shi Y.-M., Schaeren O. P. (2020). Engineering bacterial symbionts of
nematodes improves their biocontrol potential to counter the western
corn rootworm. Nature biotechnology.

[1172] Proverbs M. (1969). Induced sterilization and control
of insects. Annual Review of Entomology.

[1173] Vreysen M. J., Robinson A. S. (2011). Ionising radiation and area-wide
management of insect pests to promote sustainable agriculture. Sustainable Agriculture Volume 2.

[1174] Fu G., Condon K. C., Epton M. J., Gong P., Jin L., Condon G. C., Morrison N. I., Dafa'alla T. H., Alphey L. (2007). Female-specific insect lethality engineered using alternative
splicing. Nat Biotechnol.

[1175] Asadi R., Elaini R., Lacroix R., Ant T., Collado A., Finnegan L., Siciliano P., Mazih A., Koukidou M. (2020). Preventative releases of self-limiting
Ceratitis capitata provide pest suppression and protect fruit quality
in outdoor netted cages. International Journal
of Pest Management.

[1176] Palmer, C. Genetic Engineering Aims to Take a Bite Out of Insect Pests. Elsevier: 2022.

[1177] Waltz E. (2015). Oxitec trials
GM sterile moth to combat agricultural infestations. Nature Biotechnology.

[1178] Schwindenhammer S. (2020). The rise, regulation and risks of
genetically modified
insect technology in global agriculture. Science,
Technology and Society.

[1179] Carey J. (2018). The race to extinguish insect pests
by enlisting their own kind. Proceedings of
the National Academy of Sciences.

[1180] Rhoades M. (1942). Preferential segregation in maize. Genetics.

[1181] Wang C., Wang J., Lu J., Xiong Y., Zhao Z., Yu X., Zheng X., Li J., Lin Q., Ren Y. (2023). A natural gene drive system confers reproductive isolation
in rice. Cell.

[1182] Yu X., Zhao Z., Zheng X., Zhou J., Kong W., Wang P., Bai W., Zheng H., Zhang H., Li J. (2018). A selfish genetic element
confers non-Mendelian inheritance in rice. Science.

[1183] Buchman A., Marshall J. M., Ostrovski D., Yang T., Akbari O. S. (2018). Synthetically
engineered Medea gene
drive system in the worldwide crop pest Drosophila suzukii. Proc Natl Acad Sci U S A.

[1184] Stokstad E. (2024). Two teams supercharge gene spread
in plants. Science.

[1185] Chan Y. S., Huen D. S., Glauert R., Whiteway E., Russell S. (2013). Optimising homing endonuclease gene
drive performance
in a semi-refractory species: the Drosophila melanogaster experience. PLoS One.

[1186] Burt A. (2003). Site-specific selfish genes as tools for the control
and genetic
engineering of natural populations. Proceedings
of the Royal Society of London. Series B: Biological Sciences.

[1187] Esvelt, K. ; Smidler, A. ; Catteruccia, F. ; Church, G. Emerging technology: concerning RNA-guided gene drives for the alteration of wild populations. elife 2014, 3, e03401. 10.7554/eLife.03401 25035423 PMC4117217

[1188] DiCarlo J. E., Chavez A., Dietz S. L., Esvelt K. M., Church G. M. (2015). Safeguarding CRISPR-Cas9 gene drives
in yeast. Nat Biotechnol.

[1189] Carrami E. M., Eckermann K. N., Ahmed H. M. M., Sanchez C. H., Dippel S., Marshall J. M., Wimmer E. A. (2018). Consequences of
resistance evolution in a Cas9-based sex conversion-suppression gene
drive for insect pest management. Proc Natl
Acad Sci U S A.

[1190] Gantz V. M., Bier E. (2015). The mutagenic chain
reaction: a method
for converting heterozygous to homozygous mutations. Science.

[1191] Ma S., Ni X., Chen S., Qiao X., Xu X., Chen W., Champer J., Huang J. (2024). A small-molecule approach
to restore female sterility phenotype targeted by a homing suppression
gene drive in the fruit pest Drosophila suzukii. PLoS Genet.

[1192] Asad M., Liu D., Li J., Chen J., Yang G. (2022). Development of CRISPR/Cas9-Mediated Gene-Drive Construct Targeting
the Phenotypic Gene in Plutella xylostella. Front Physiol.

[1193] Meccariello A., Hou S., Davydova S., Fawcett J. D., Siddall A., Leftwich P. T., Krsticevic F., Papathanos P. A., Windbichler N. (2024). Gene drive
and genetic sex conversion
in the global agricultural pest Ceratitis capitata. Nat Commun.

[1194] Godwin J., Serr M., Barnhill-Dilling S. K., Blondel D. V., Brown P. R., Campbell K., Delborne J., Lloyd A. L., Oh K. P., Prowse T. A. (2019). Rodent gene drives
for conservation: opportunities and data needs. Proceedings of the Royal Society B.

[1195] Grunwald H. A., Gantz V. M., Poplawski G., Xu X. S., Bier E., Cooper K. L. (2019). Super-Mendelian
inheritance mediated by CRISPR-Cas9 in the female mouse germline. Nature.

[1196] Barrett L. G., Legros M., Kumaran N., Glassop D., Raghu S., Gardiner D. M. (2019). Gene drives in plants:
opportunities
and challenges for weed control and engineered resilience. Proc Biol Sci.

[1197] Neve P. (2018). Gene drive systems: do they have a place in agricultural weed management?. Pest Management Science.

[1198] Liu Y., Jiao B., Champer J., Qian W. (2024). Overriding Mendelian
inheritance in Arabidopsis with a CRISPR toxin-antidote gene drive
that impairs pollen germination. Nat Plants.

[1199] Fujita M., Fujita Y., Noutoshi Y., Takahashi F., Narusaka Y., Yamaguchi-Shinozaki K., Shinozaki K. (2006). Crosstalk
between abiotic and biotic stress responses: a current view from the
points of convergence in the stress signaling networks. Current opinion in plant biology.

[1200] Zhu X.-G., Ort D. R., Parry M. A. J., von
Caemmerer S. (2020). A wish list for synthetic biology in photosynthesis
research. Journal of Experimental Botany.

[1201] Farmer E. E. (2001). Surface-to-air
signals. Nature.

[1202] Steinbrenner A. D., Munoz-Amatriain M., Chaparro A. F., Jessica
Montserrat A.-V., Lo S., Okuda S., Glauser G., Dongiovanni J., Shi D., Hall M. (2020). A receptor-like
protein mediates plant immune responses to herbivore-associated molecular
patterns. P Natl Acad Sci USA.

[1203] Tena G., Boudsocq M., Sheen J. (2011). Protein kinase signaling
networks in plant innate immunity. Current Opinion
in Plant Biology.

[1204] Ueda Y., Yanagisawa S. (2019). Delineation of Nitrogen Signaling
Networks: Computational Approaches in the Big Data Era. Molecular Plant.

[1205] Sheen J. (2014). Master regulators in plant glucose
signaling networks. Journal of Plant Biology.

[1206] Scotti N., Cardi T. (2014). Transgene-induced pleiotropic effects
in transplastomic plants. Biotechnol Lett.

[1207] Eom J.-S., Luo D., Atienza-Grande G., Yang J., Ji C., Thi Luu V., Huguet-Tapia J. C., Char S. N., Liu B., Nguyen H. (2019). Diagnostic
kit for rice blight resistance. Nature Biotechnology.

[1208] Khodakovskaya M., McAvoy R., Peters J., Wu H., Li Y. (2006). Enhanced cold
tolerance in transgenic tobacco expressing a chloroplast
ω-3 fatty acid desaturase gene under the control of a cold-inducible
promoter. Planta.

[1209] Hoshika S., Leal N. A., Kim M. J., Kim M. S., Karalkar N. B., Kim H. J., Bates A. M., Watkins N. E., SantaLucia H. A., Meyer A. J. (2019). Hachimoji
DNA and RNA: A genetic system with eight building blocks. Science.

[1210] Basu S., Mehreja R., Thiberge S., Chen M.-T., Weiss R. (2004). Spatiotemporal control of gene expression
with pulse-generating networks. Proceedings
of the National Academy of Sciences.

[1211] Sohka T., Heins R. A., Phelan R. M., Greisler J. M., Townsend C. A., Ostermeier M. (2009). An externally
tunable bacterial band-pass
filter. Proceedings of the National Academy
of Sciences.

[1212] Blount B. A., Weenink T., Ellis T. (2012). Construction of synthetic
regulatory networks in yeast. FEBS Lett..

[1213] Boni R., Chauhan H., Hensel G., Roulin A., Sucher J., Kumlehn J., Brunner S., Krattinger S. G., Keller B. (2018). Pathogen-inducible Ta-Lr34res expression in heterologous
barley confers disease resistance without negative pleiotropic effects. Plant Biotechnol J.

[1214] Yang, X. ; Liu, D. ; Lu, H. ; Weston, D. J. ; Chen, J.-G. ; Muchero, W. ; Martin, S. ; Liu, Y. ; Hassan, M. M. ; Yuan, G. Biological Parts for Plant Biodesign to Enhance Land-Based Carbon Dioxide Removal. BioDesign Research 2021, 2021, 9798714. 10.34133/2021/9798714 37849951 PMC10521660

[1215] Endo S., Betsuyaku S., Fukuda H. (2014). Endogenous peptide
ligand-receptor systems for diverse signaling networks in plants. Current Opinion in Plant Biology.

[1216] Liu S.-M., Li J., Zhu J.-Q., Wang X.-W., Wang C.-S., Liu S.-S., Chen X.-X., Li S. (2016). Transgenic
plants expressing the AaIT/GNA fusion protein show increased resistance
and toxicity to both chewing and sucking pests. Insect Science.

[1217] Dóczi R., Brader G., Pettkó-Szandtner A., Rajh I., Djamei A., Pitzschke A., Teige M., Hirt H. (2007). The Arabidopsis mitogen-activated
protein kinase kinase MKK3 is upstream of group C mitogen-activated
protein kinases and participates in pathogen signaling. Plant Cell.

[1218] Wang J., Mei J., Ren G. (2019). Plant microRNAs:
Biogenesis,
homeostasis, and degradation. Frontiers in Plant
Science.

[1219] Yilmaz A., Mejia-Guerra M. K., Kurz K., Liang X., Welch L., Grotewold E. (2011). AGRIS: The arabidopsis gene regulatory
information server, an update. Nucleic Acids
Research.

[1220] Jones D. M., Vandepoele K. (2020). Identification and evolution of gene
regulatory networks: insights from comparative studies in plants. Current Opinion in Plant Biology.

[1221] Haque S., Ahmad J. S., Clark N. M., Williams C. M., Sozzani R. (2019). Computational prediction of gene
regulatory networks
in plant growth and development. Current Opinion
in Plant Biology.

[1222] Tu X., Mejia-Guerra M. K., Valdes Franco J. A., Tzeng D., Chu P. Y., Shen W., Wei Y., Dai X., Li P., Buckler E. S. (2020). Reconstructing the maize
leaf regulatory network using ChIP-seq data of 104 transcription factors. Nat Commun.

[1223] O'Malley R. C., Huang S. C., Song L., Lewsey M. G., Bartlett A., Nery J. R., Galli M., Gallavotti A., Ecker J. R. (2016). Cistrome and Epicistrome Features
Shape the Regulatory
DNA Landscape. Cell.

[1224] Hussey S. G., Grima-Pettenati J., Myburg A. A., Mizrachi E., Brady S. M., Yoshikuni Y., Deutsch S. (2019). A Standardized Synthetic
Eucalyptus Transcription Factor and Promoter Panel for Re-engineering
Secondary Cell Wall Regulation in Biomass and Bioenergy Crops. ACS Synth Biol.

[1225] Ikeuchi M., Shibata M., Rymen B., Iwase A., Bagman A. M., Watt L., Coleman D., Favero D. S., Takahashi T., Ahnert S. E. (2018). A Gene
Regulatory Network
for Cellular Reprogramming in Plant Regeneration. Plant Cell Physiol.

[1226] Taylor-Teeples M., Lin L., de Lucas M., Turco G., Toal T. W., Gaudinier A., Young N. F., Trabucco G. M., Veling M. T., Lamothe R. (2015). An Arabidopsis gene
regulatory network for secondary cell wall synthesis. Nature.

[1227] Li G.-W., Burkhardt D., Gross C., Weissman J. S. (2014). Quantifying
absolute protein synthesis rates reveals principles underlying allocation
of cellular resources. Cell.

[1228] Tian F., Yang D. C., Meng Y. Q., Jin J., Gao G. (2019). PlantRegMap: charting functional regulatory maps in
plants. Nucleic Acids Res.

[1229] Zhou P., Li Z., Magnusson E., Cano F. G., Crisp P. A., Noshay J. M., Grotewold E., Hirsch C. N., Briggs S. P., Springer N. M. (2020). Meta gene regulatory
networks in maize highlight functionally relevant regulatory interactions. Plant Cell.

[1230] Genoud T., Trevino Santa Cruz M.
B., Métraux J. P. (2001). Numeric
simulation of plant signaling networks. Plant
Physiology.

[1231] Gao Y., Zhu N., Zhu X., Wu M., Jiang C. Z., Grierson D., Luo Y., Shen W., Zhong S., Fu D. Q. (2019). Diversity and redundancy of the ripening regulatory
networks revealed by the fruitENCODE and the new CRISPR/Cas9 CNR and
NOR mutants. Hortic Res.

[1232] Butelli E., Titta L., Giorgio M., Mock H.-P., Matros A., Peterek S., Schijlen E. G. W. M., Hall R. D., Bovy A. G., Luo J. (2008). Enrichment
of tomato fruit with health-promoting anthocyanins by expression of
select transcription factors. Nature Biotechnology.

[1233] Zhang Q., Feng C., Li W., Qu Z., Zeng M., Xi W. (2019). Transcriptional regulatory networks
controlling taste and aroma quality of apricot (Prunus armeniaca L.)
fruit during ripening. BMC Genomics.

[1234] Guseman J. M., Webb K., Srinivasan C., Dardick C. (2017). DRO1 influences root system architecture in Arabidopsis
and Prunus species. Plant J.

[1235] Freiman A., Shlizerman L., Golobovitch S., Yablovitz Z., Korchinsky R., Cohen Y., Samach A., Chevreau E., Le Roux P. M., Patocchi A. (2012). Development
of a transgenic early flowering pear (Pyrus communis L.) genotype
by RNAi silencing of PcTFL1-1 and PcTFL1-2. Planta.

[1236] Wang J., Zhou L., Shi H., Chern M., Yu H., Yi H., He M., Yin J., Zhu X., Li Y. (2018). A single transcription factor promotes both yield and
immunity in rice. Science.

[1237] Liu M., Shi Z., Zhang X., Wang M., Zhang L., Zheng K., Liu J., Hu X., Di C., Qian Q. (2019). Inducible overexpression
of Ideal Plant Architecture1
improves both yield and disease resistance in rice. Nat Plants.

[1238] Peng J., Richards D. E., Hartley N. M., Murphy G. P., Devos K. M., Flintham J. E., Beales J., Fish L. J., Worland A. J., Pelica F. (1999). 'Green revolution'
genes
encode mutant gibberellin response modulators. Nature.

[1239] Bennett A. B., Pankievicz V. C. S., Ané J. M. (2020). A Model
for Nitrogen Fixation in Cereal Crops. Trends
in Plant Science.

[1240] Gao Y., Wei W., Fan Z., Zhao X., Zhang Y., Jing Y., Zhu B., Zhu H., Shan W., Chen J. (2020). Re-evaluation of the
nor mutation and the role of the
NAC-NOR transcription factor in tomato fruit ripening. J Exp Bot.

[1241] Yoshida K., Sakamoto S., Kawai T., Kobayashi Y., Sato K., Ichinose Y., Yaoi K., Akiyoshi-Endo M., Sato H., Takamizo T. (2013). Engineering
the Oryza
sativa cell wall with rice NAC transcription factors regulating secondary
wall formation. Front Plant Sci.

[1242] Ohta M., Matsui K., Hiratsu K., Shinshi H., Ohme-Takagi M. (2001). Repression domains of class II ERF
transcriptional
repressors share an essential motif for active repression. Plant Cell.

[1243] Hiratsu K., Matsui K., Koyama T., Ohme-Takagi M. (2003). Dominant repression
of target genes by chimeric repressors that include the EAR motif,
a repression domain, in Arabidopsis. Plant J.

[1244] Mahfouz M. M., Li L., Piatek M., Fang X., Mansour H., Bangarusamy D. K., Zhu J. K. (2012). Targeted transcriptional
repression using a chimeric TALE-SRDX repressor protein. Plant Molecular Biology.

[1245] Cen H., Ye W., Liu Y., Li D., Wang K., Zhang W. (2016). Overexpression of a Chimeric Gene,
OsDST-SRDX, Improved Salt Tolerance
of Perennial Ryegrass. Sci Rep.

[1246] Piatek A., Ali Z., Baazim H., Li L., Abulfaraj A., Al-Shareef S., Aouida M., Mahfouz M. M. (2015). RNA-guided
transcriptional regulation in planta via synthetic dCas9-based transcription
factors. Plant Biotechnol J.

[1247] Matsui K., Umemura Y., Ohme-Takagi M. (2008). AtMYBL2, a
protein with a single MYB domain, acts as a negative regulator of
anthocyanin biosynthesis in Arabidopsis. Plant
J.

[1248] Ikeda M., Ohme-Takagi M. (2009). A novel group
of transcriptional
repressors in Arabidopsis. Plant Cell Physiol.

[1249] Kassaw T. K., Xu W., Zalewski C. S., Kiwimagi K., Weiss R., Antunes M. S., Prasad A., Medford J. I. (2025). A Genetic
Toggle Switch in Plants. bioRxiv.

[1250] Sadowski I., Ma J., Triezenberg S., Ptashne M. (1988). GAL4-VP16 is an unusually potent transcriptional activator. Nature.

[1251] Campbell M. E., Palfreyman J. W., Preston C. M. (1984). Identification of
herpes simplex virus DNA sequences which encode a trans-acting polypeptide
responsible for stimulation of immediate early transcription. J Mol Biol.

[1252] Zhou H., Xu L., Li F., Li Y. (2022). Transcriptional
regulation by CRISPR/dCas9 in common wheat. Gene.

[1253] Lowder L. G., Zhou J., Zhang Y., Malzahn A., Zhong Z., Hsieh T. F., Voytas D. F., Zhang Y., Qi Y. (2018). Robust Transcriptional Activation
in Plants Using Multiplexed CRISPR-Act2.0
and mTALE-Act Systems. Molecular Plant.

[1254] Miglani, G. S. ; Kaur, A. ; Kaur, L. Plant gene expression control using genome- and epigenome-editing technologies. Journal of Crop Improvement 2020, 34 (1), 1. 10.1080/15427528.2019.1678541

[1255] Lowder L. G., Zhang D., Baltes N. J., Paul J. W., Tang X., Zheng X., Voytas D. F., Hsieh T. F., Zhang Y., Qi Y. (2015). A CRISPR/Cas9 Toolbox
for Multiplexed Plant Genome Editing and Transcriptional Regulation. Plant Physiol.

[1256] Beerli R. R., Segal D. J., Dreier B., Barbas C. F. (1998). 3rd. Toward
controlling gene expression at will: specific regulation of the erbB-2/HER-2
promoter by using polydactyl zinc finger proteins constructed from
modular building blocks. Proc Natl Acad Sci
U S A.

[1257] Liu W., Mazarei M., Rudis M. R., Fethe M. H., Peng Y., Millwood R. J., Schoene G., Burris J. N., Stewart C. N. (2013). Bacterial
pathogen phytosensing in transgenic tobacco and Arabidopsis plants. Plant Biotechnol J.

[1258] Raikhel N. (1992). Nuclear targeting in plants. Plant Physiol.

[1259] Iacopino S., Jurinovich S., Cupellini L., Piccinini L., Cardarelli F., Perata P., Mennucci B., Giuntoli B., Licausi F. (2019). A Synthetic
Oxygen Sensor for Plants
Based on Animal Hypoxia Signaling. Plant Physiology.

[1260] Andersen J. B., Sternberg C., Poulsen L. K., Bjorn S. P., Givskov M., Molin S. (1998). New unstable variants of green fluorescent
protein for studies of transient gene expression in bacteria. Appl Environ Microbiol.

[1261] Keiler K. C., Waller P. R., Sauer R. T. (1996). Role of
a peptide
tagging system in degradation of proteins synthesized from damaged
messenger RNA. Science.

[1262] Jadhav P., Chen Y., Butzin N., Buceta J., Urchueguia A. (2022). Bacterial degrons in synthetic circuits. Open Biol.

[1263] Elowitz M. B., Leibler S. (2000). A synthetic oscillatory network of
transcriptional regulators. Nature.

[1264] Stricker J., Cookson S., Bennett M. R., Mather W. H., Tsimring L. S., Hasty J. (2008). A fast, robust
and tunable synthetic
gene oscillator. Nature.

[1265] Gander, M. W. ; Vrana, J. D. ; Voje, W. E. ; Carothers, J. M. ; Klavins, E. Digital logic circuits in yeast with CRISPR-dCas9 NOR gates. Nature Communications 2017, 8, 10.1038/ncomms15459. PMC5458518 28541304

[1266] Weinmann P., Gossen M., Hillen W., Bujard H., Gatz C. (1994). A chimeric transactivator allows
tetracycline-responsive gene expression
in whole plants. The Plant Journal.

[1267] Gardner M. J., Baker A. J., Assie J.-M., Poethig R. S., Haseloff J. P., Webb A. A. R. (2009). GAL4 GFP enhancer
trap lines for
analysis of stomatal guard cell development and gene expression. Journal of Experimental Botany.

[1268] Nielsen A. A., Voigt C. A. (2014). Multi-input CRISPR/Cas
genetic circuits
that interface host regulatory networks. Mol
Syst Biol.

[1269] Holmes-Davis R., Li G., Jamieson A. C., Rebar E. J., Liu Q., Kong Y., Case C. C., Gregory P. D. (2005). Gene regulation
in planta by plant-derived engineered zinc finger protein transcription
factors. Plant Molecular Biology.

[1270] Van Eenennaam A. L., Li G., Venkatramesh M., Levering C., Gong X., Jamieson A. C., Rebar E. J., Shewmaker C. K., Case C. C. (2004). Elevation of seed alpha-tocopherol
levels using plant-based transcription factors targeted to an endogenous
locus. Metab. Eng..

[1271] Dandekar A. M., Gouran H., Ibáñez A. M., Uratsu S. L., Agüero C. B., McFarland S., Borhani Y., Feldstein P. A., Bruening G., Nascimento R. (2012). An engineered innate immune defense protects grapevines from Pierce
disease. P Natl Acad Sci USA.

[1272] Boch J., Bonas U. (2010). Xanthomonas AvrBs3
Family-Type III
Effectors: Discovery and Function. Annual Review
of Phytopathology.

[1273] Kurbidaeva, A. ; Purugganan, M. Insulators in Plants: Progress and Open Questions. Genes (Basel) 2021, 12 (9), 1422 10.3390/genes12091422. 34573404 PMC8470105

[1274] Fujioka M., Mistry H., Schedl P., Jaynes J. B. (2016). Determinants
of Chromosome Architecture: Insulator Pairing in cis and in trans. PLoS Genet.

[1275] Gan, S. ; Xie, M. Genetic insulator for preventing influence by another gene promoter. US20060174370A1, 2009.

[1276] Vain P., Worland B., Kohli A., Snape J. W., Christou P., Allen G. C., Thompson W. F. (1999). Matrix
attachment
regions increase transgene expression levels and stability in transgenic
rice plants and their progeny. The Plant Journal.

[1277] Keung A. J., Bashor C. J., Kiriakov S., Collins J. J., Khalil A. S. (2014). Using targeted
chromatin regulators to engineer combinatorial
and spatial transcriptional regulation. Cell.

[1278] Wang H., Huang B., Wang J. (2021). Predict long-range
enhancer regulation based on protein-protein interactions between
transcription factors. Nucleic Acids Res.

[1279] Brophy J. A. N., Voigt C. A. (2014). Principles of genetic circuit design. Nature Methods.

[1280] Nemhauser J. L., Torii K. U. (2016). Plant synthetic
biology for molecular
engineering of signalling and development. Nat
Plants.

[1281] Walia A., Waadt R., Jones A. M. (2018). Genetically Encoded
Biosensors in Plants: Pathways to Discovery. Annu Rev Plant Biol.

[1282] Park S., Lee K., Kim Y. S., Chi Y. T., Shin J. S., Back K. (2012). Induced tyramine overproduction
in
transgenic rice plants expressing a rice tyrosine decarboxylase under
the control of methanol-inducible rice tryptophan decarboxylase promoter. Bioprocess Biosyst Eng.

[1283] Kang K., Park S., Natsagdorj U., Kim Y. S., Back K. (2011). Methanol is an endogenous elicitor
molecule for the synthesis of tryptophan and tryptophan-derived secondary
metabolites upon senescence of detached rice leaves. The Plant Journal.

[1284] Hammond J. P., Bennett M. J., Bowen H. C., Broadley M. R., Eastwood D. C., May S. T., Rahn C., Swarup R., Woolaway K. E., White P. J. (2003). Changes in gene
expression in Arabidopsis
shoots during phosphate starvation and the potential for developing
smart plants. Plant Physiology.

[1285] Kamiya T., Yamagami M., Hirai M. Y., Fujiwara T. (2012). Establishment
of an in planta magnesium monitoring system using CAX3 promoter-luciferase
in Arabidopsis. Journal of Experimental Botany.

[1286] Krizek B. A., Prost V., Joshi R. M., Stoming T., Glenn T. C. (2003). Developing
transgenic arabidopsis plants to be metal-specific
bioindicators. Environmental Toxicology and
Chemistry.

[1287] Zarka D. G., Vogel J. T., Cook D., Thomashow M. F. (2003). Cold Induction
of Arabidopsis CBF Genes Involves Multiple ICE (Inducer of CBF Expression)
Promoter Elements and a Cold-Regulatory Circuit That Is Desensitized
by Low Temperature. Plant Physiology.

[1288] Brey L. F., Włodarczyk A. J., Bang Thøfner J.
F., Burow M., Crocoll C., Nielsen I., Zygadlo
Nielsen A. J., Jensen P. E. (2020). Metabolic engineering of Synechocystis
sp. PCC 6803 for the production of aromatic amino acids and derived
phenylpropanoids. Metab. Eng..

[1289] Nishizawa A., Yabuta Y., Yoshida E., Maruta T., Yoshimura K., Shigeoka S. (2006). Arabidopsis heat shock
transcription
factor A2 as a key regulator in response to several types of environmental
stress. Plant J..

[1290] Zhang J., Yu W.-J., Xiong A.-S., Bahramnejad B., Erickson L. R. (2011). Isolation and characterization of
a harvest-induced
promoter of an alfalfa gene, hi7. Plant Growth
Regulation.

[1291] Rushton P. J., Reinstädler A., Lipka V., Lippok B., Somssich I. E. (2002). Synthetic Plant Promoters Containing Defined Regulatory
Elements Provide Novel Insights into Pathogen- and Wound-Induced Signaling. The Plant Cell.

[1292] Cheng W., Song X.-S., Li H.-P., Cao L.-H., Sun K., Qiu X.-L., Xu Y.-B., Yang P., Huang T., Zhang J.-B. (2015). Host-induced
gene silencing of an essential
chitin synthase gene confers durable resistance to Fusarium head blight
and seedling blight in wheat. Plant Biotechnol
J.

[1293] Higo K., Ugawa Y., Iwamoto M., Korenaga T. (1999). Plant cis-acting
regulatory DNA elements (PLACE) database: 1999. Nucleic Acids Research.

[1294] Lescot M., Déhais P., Thijs G., Marchal K., Moreau Y., Van de Peer Y., Rouzé P., Rombauts S. (2002). PlantCARE, a database of plant cis-acting
regulatory
elements and a portal to tools for in silico analysis of promoter
sequences. Nucleic Acids Research.

[1295] Hieno A., Naznin H. A., Hyakumachi M., Sakurai T., Tokizawa M., Koyama H., Sato N., Nishiyama T., Hasebe M., Zimmer A. D. (2014). ppdb:
plant promoter database version 3.0. Nucleic
Acids Research.

[1296] Chow C.-N., Zheng H.-Q., Wu N.-Y., Chien C.-H., Huang H.-D., Lee T.-Y., Chiang-Hsieh Y.-F., Hou P.-F., Yang T.-Y., Chang W.-C. (2016). PlantPAN 2.0: an
update of plant promoter analysis navigator for reconstructing transcriptional
regulatory networks in plants. Nucleic Acids
Research.

[1297] Khakhar, A. ; Leydon, A. R. ; Lemmex, A. C. ; Klavins, E. ; Nemhauser, J. L. Synthetic hormone-responsive transcription factors can monitor and re-program plant development. Elife 2018, 7, 10.7554/eLife.34702. PMC5976440 29714687

[1298] Cuthbertson L., Nodwell J. R. (2013). The TetR Family of Regulators. Microbiology and Molecular Biology Reviews.

[1299] Gatz C., Frohberg C., Wendenburg R. (1992). Stringent
repression and homogeneous de-repression by tetracycline of a modified
CaMV 35S promoter in intact transgenic tobacco plants. Plant J.

[1300] Arst H. N., Holden D. W., Caddick M. X. (1997). Evolution
of transcription-regulating proteins: caveat lector!. Gene.

[1301] Stanton B. C., Siciliano V., Ghodasara A., Wroblewska L., Clancy K., Trefzer A. C., Chesnut J. D., Weiss R., Voigt C. A. (2014). Systematic transfer of prokaryotic
sensors and circuits to mammalian cells. ACS
Synth. Biol..

[1302] Ramos J. L., Martinez-Bueno M., Molina-Henares A. J., Teran W., Watanabe K., Zhang X., Gallegos M. T., Brennan R., Tobes R. (2005). The TetR family
of transcriptional
repressors. Microbiol Mol Biol Rev.

[1303] Weber W., Fussenegger M. (2012). Emerging biomedical
applications
of synthetic biology. Nat Rev Genet.

[1304] Xie M., Fussenegger M. (2018). Designing
cell function: assembly of synthetic gene
circuits for cell biology applications. Nat
Rev Mol Cell Biol.

[1305] Ikushima S., Boeke J. D. (2017). New Orthogonal Transcriptional Switches
Derived from Tet Repressor Homologues for Saccharomyces cerevisiae
Regulated by 2,4-Diacetylphloroglucinol and Other Ligands. ACS Synth Biol.

[1306] Zuo J., Niu Q.-W., Chua N.-H. (2000). An estrogen
receptor-based transactivator
XVE mediates highly inducible gene expression in transgenic plants. The Plant Journal.

[1307] Persad, R. ; Reuter, D. N. ; Dice, L. T. ; Nguyen, M.-A. ; Rigoulot, S. B. ; Layton, J. S. ; Schmid, M. J. ; Poindexter, M. R. ; Occhialini, A. ; Stewart, C. N., Jr. ; The Q-System as a Synthetic Transcriptional Regulator in Plants. Frontiers in Plant Science 2020, 11, 10.3389/fpls.2020.00245. PMC7078239 32218793

[1308] Zuo, J. ; Chua, N.-H. Chemical-inducible Systems for Regulated Expression of Plant Genes. Curr Opin Biotech 2000, 11, 146. 10.1016/S0958-1669(00)00073-2 10753773

[1309] Samalova M., Brzobohaty B., Moore I. (2005). pOp6/LhGR: a stringently
regulated and highly responsive dexamethasone-inducible gene expression
system for tobacco. The Plant Journal.

[1310] Boo A., Toth T., Yu Q., Pfotenhauer A., Fields B. D., Lenaghan S. C., Stewart C. N., Voigt C. A. (2024). Synthetic microbe-to-plant communication channels. Nat Commun.

[1311] Dohmen R. J., Wu P., Varshavsky A. (1994). Heat-inducible
degron: a method for constructing temperature-sensitive mutants. Science.

[1312] Faden F., Ramezani T., Mielke S., Almudi I., Nairz K., Froehlich M. S., Hockendorff J., Brandt W., Hoehenwarter W., Dohmen R. J. (2016). Phenotypes
on demand via switchable target protein degradation in multicellular
organisms. Nat Commun.

[1313] Banaszynski L. A., Chen L. C., Maynard-Smith L. A., Ooi A. G., Wandless T. J. (2006). A rapid, reversible, and tunable
method to regulate protein function in living cells using synthetic
small molecules. Cell.

[1314] Bonger K. M., Chen L. C., Liu C. W., Wandless T. J. (2011). Small-molecule
displacement of a cryptic degron causes conditional protein degradation. Nat Chem Biol.

[1315] Padidam M., Gore M., Lily Lu D., Smirnova O. (2003). Chemical-Inducible,
Ecdysone Receptor-Based Gene Expression System for Plants. Transgenic Research.

[1316] Tavva V. S., Dinkins R. D., Palli S. R., Collins G. B. (2006). Development
of a methoxyfenozide-responsive gene switch for applications in plants. Plant J.

[1317] Martinez A., Sparks C., Hart C. A., Thompson J., Jepson I. (1999). Ecdysone agonist inducible transcription
in transgenic
tobacco plants. Plant J.

[1318] Serganov A., Nudler E. (2013). A decade of riboswitches. Cell.

[1319] Jang S., Jang S., Noh M. H., Lim H. G., Jung G. Y. (2018). Novel Hybrid Input Part Using Riboswitch and Transcriptional
Repressor for Signal Inverting Amplifier. ACS
Synth. Biol..

[1320] Shanidze N., Lenkeit F., Hartig J. S., Funck D. (2020). A Theophylline-Responsive
Riboswitch Regulates Expression of Nuclear-Encoded Genes. Plant Physiology.

[1321] Ramundo S., Rahire M., Schaad O., Rochaix J.-D. (2013). Repression
of Essential Chloroplast Genes Reveals New Signaling Pathways and
Regulatory Feedback Loops in Chlamydomonas. The Plant Cell.

[1322] Bocobza S., Adato A., Mandel T., Shapira M., Nudler E., Aharoni A. (2007). Riboswitch-dependent gene regulation
and its evolution in the plant kingdom. Genes
Dev.

[1323] Ellington A. D., Szostak J. W. (1990). In vitro selection of RNA molecules
that bind specific ligands. Nature.

[1324] Tuerk C., Gold L. (1990). Systematic evolution
of ligands by
exponential enrichment: RNA ligands to bacteriophage T4 DNA polymerase. Science.

[1325] Bayer T. S., Smolke C. D. (2005). Programmable ligand-controlled
riboregulators
of eukaryotic gene expression. Nat Biotechnol.

[1326] Auslander S., Ketzer P., Hartig J. S. (2010). A ligand-dependent
hammerhead ribozyme switch for controlling mammalian gene expression. Mol Biosyst.

[1327] Bick, M. J. ; Greisen, P. J. ; Morey, K. J. ; Antunes, M. S. ; La, D. ; Sankaran, B. ; Reymond, L. ; Johnsson, K. ; Medford, J. I. ; Baker, D. Computational design of environmental sensors for the potent opioid fentanyl. Elife 2017, 6, 10.7554/eLife.28909. PMC5655540 28925919

[1328] Tinberg, C. E. ; Khare, S. D. ; Dou, J. ; Doyle, L. ; Nelson, J. W. ; Schena, A. ; Jankowski, W. ; Kalodimos, C. G. ; Johnsson, K. ; Stoddard, B. L. ; Baker, D. ; Computational Design of Ligand-Binding Proteins With High Affinity and Selectivity. Nature 2013, 501, 212. 10.1038/nature12443 24005320 PMC3898436

[1329] Tang S. Y., Cirino P. C. (2011). Design and application of a mevalonate-responsive
regulatory protein. Angewandte Chemie - International
Edition.

[1330] Darmostuk M., Rimpelova S., Gbelcova H., Ruml T. (2015). Current approaches
in SELEX: An update to aptamer selection technology. Biotechnol Adv.

[1331] Bick M. J., Greisen P. J., Morey K. J., Antunes M. S., La D., Sankaran B., Reymond L., Johnsson K., Medford J. I., Baker D. (2017). Computational design
of environmental sensors for the potent opioid
fentanyl. eLife.

[1332] Feng J., Jester B. W., Tinberg C. E., Mandell D. J., Antunes M. S., Chari R., Morey K. J., Rios X., Medford J. I., Church G. M. (2015). A general strategy to
construct small molecule biosensors in eukaryotes. eLife.

[1333] Tinberg C. E., Khare S. D., Dou J., Doyle L., Nelson J. W., Schena A., Jankowski W., Kalodimos C. G., Johnsson K., Stoddard B. L. (2013). Computational
design of ligand-binding proteins with high affinity and selectivity. Nature.

[1334] Uchida N., Takahashi K., Iwasaki R., Yamada R., Yoshimura M., Endo T. A., Kimura S., Zhang H., Nomoto M., Tada Y. (2018). Chemical hijacking of
auxin signaling with an engineered auxin-TIR1 pair. Nat Chem Biol.

[1335] Antunes M. S., Morey K. J., Tewari-Singh N., Bowen T. A., Smith J. J., Webb C. T., Hellinga H. W., Medford J. I. (2009). Engineering key components in a synthetic eukaryotic
signal transduction pathway. Molecular Systems
Biology.

[1336] Antunes, M. S. ; Morey, K. J. ; Smith, J. J. ; Albrecht, K. D. ; Bowen, T. A. ; Zdunek, J. K. ; Troupe, J. F. ; Cuneo, M. J. ; Webb, C. T. ; Hellinga, H. W. ; Programmable Ligand Detection System in Plants through a Synthetic Signal Transduction Pathway. PLoS ONE 2011, 6 (1), e16292 10.1371/journal.pone.0016292. 21283542 PMC3026823

[1337] Voigt C. A. (2006). Genetic parts to program bacteria. Curr Opin Biotechnol.

[1338] Meyer A. J., Segall-Shapiro T. H., Glassey E., Zhang J., Voigt C. A. (2019). Escherichia coli
“Marionette” strains
with 12 highly optimized small-molecule sensors. Nat Chem Biol.

[1339] Lee S. K., Chou H. H., Pfleger B. F., Newman J. D., Yoshikuni Y., Keasling J. D. (2007). Directed evolution of AraC for improved
compatibility of arabinose- and lactose-inducible promoters. Appl Environ Microbiol.

[1340] Stanton B. C., Nielsen A. A., Tamsir A., Clancy K., Peterson T., Voigt C. A. (2014). Genomic mining of
prokaryotic repressors
for orthogonal logic gates. Nat Chem Biol.

[1341] Daber R., Sochor M. A., Lewis M. (2011). Thermodynamic analysis
of mutant lac repressors. J Mol Biol.

[1342] Corrado G., Karali M. (2009). Inducible gene expression systems
and plant biotechnology. Biotechnol Adv.

[1343] Bull T., Khakhar A. (2023). Design principles for synthetic control
systems to engineer plants. Plant Cell Rep.

[1344] Fu Y., Bannach O., Chen H., Teune J. H., Schmitz A., Steger G., Xiong L., Barbazuk W. B. (2009). Alternative splicing
of anciently exonized 5S rRNA regulates plant transcription factor
TFIIIA. Genome Res.

[1345] Hammond M. C., Wachter A., Breaker R. R. (2009). A plant
5S ribosomal
RNA mimic regulates alternative splicing of transcription factor IIIA
pre-mRNAs. Nat Struct Mol Biol.

[1346] Hickey S. F., Sridhar M., Westermann A. J., Qin Q., Vijayendra P., Liou G., Hammond M. C. (2012). Transgene regulation
in plants by alternative splicing of a suicide exon. Nucleic Acids Res.

[1347] Gonzalez T. L., Liang Y., Nguyen B. N., Staskawicz B. J., Loqué D., Hammond M. C. (2015). Tight regulation
of plant immune
responses by combining promoter and suicide exon elements. Nucleic acids research.

[1348] Teo J. J., Woo S. S., Sarpeshkar R. (2015). Synthetic
Biology: A Unifying View and Review Using Analog Circuits. IEEE Trans Biomed Circuits Syst.

[1349] Rubens J. R., Selvaggio G., Lu T. K. (2016). Synthetic mixed-signal
computation in living cells. Nat Commun.

[1350] Sarpeshkar R. (2014). Analog synthetic biology. Philos
Trans A Math Phys Eng Sci.

[1351] Daniel R., Rubens J. R., Sarpeshkar R., Lu T. K. (2013). Synthetic analog computation in living cells. Nature.

[1352] Slusarczyk A. L., Lin A., Weiss R. (2012). Foundations for the
design and implementation of synthetic genetic circuits. Nat Rev Genet.

[1353] Bittihn P., Din M. O., Tsimring L. S., Hasty J. (2018). Rational engineering
of synthetic microbial systems: from single cells to consortia. Curr Opin Microbiol.

[1354] Ceroni F., Ellis T. (2018). The challenges facing synthetic biology
in eukaryotes. Nat Rev Mol Cell Biol.

[1355] Brophy J. A. N., LaRue T., Dinneny J. R. (2018). Understanding and
engineering plant form. Seminars in Cell &
Developmental Biology.

[1356] Cox, R. S. ; Surette, M. G. ; Elowitz, M. B. Programming gene expression with combinatorial promoters. Molecular Systems Biology 2007, 3 (145), 10.1038/msb4100187. PMC2132448 18004278

[1357] Jones T. S., Oliveira S. M. D., Myers C. J., Voigt C. A., Densmore D. (2022). Genetic circuit design automation
with Cello 2.0. Nat Protoc.

[1358] Khan M. A., Herring G., Zhu J. Y., Oliva M., Fourie E., Johnston B., Zhang Z., Potter J., Pineda L., Pflueger J. (2025). CRISPRi-based
circuits
to control gene expression in plants. Nat Biotechnol.

[1359] Schreiber T., Prange A., Hoppe T., Tissier A. (2019). Split-tale:
A tale-based two-component system for synthetic biology applications
in planta. Plant Physiology.

[1360] Anderson C. E., Ferreira S. S., Antunes M. S. (2023). Integration
of multiple
stress signals in plants using synthetic Boolean logic gates. Plant Physiol.

[1361] Reinke A. W., Grant R. A., Keating A. E. (2010). A synthetic
coiled-coil
interactome provides heterospecific modules for molecular engineering. J Am Chem Soc.

[1362] Ferreira S. S., Antunes M. S. (2024). Genetically encoded
Boolean logic
operators to sense and integrate phenylpropanoid metabolite levels
in plants. New Phytol.

[1363] Fontanarrosa P., Doosthosseini H., Borujeni A. E., Dorfan Y., Voigt C. A., Myers C. (2020). Genetic Circuit
Dynamics: Hazard
and Glitch Analysis. ACS Synth Biol.

[1364] Purnick P. E. M., Weiss R. (2009). The second wave of
synthetic biology:
From modules to systems. Nature Reviews Molecular
Cell Biology.

[1365] Medford J. I., Prasad A. (2016). Towards programmable plant genetic
circuits. The Plant Journal.

[1366] Kassaw T. K., Donayre-Torres A. J., Antunes M. S., Morey K. J., Medford J. I. (2018). Engineering synthetic
regulatory circuits in plants. Plant Science.

[1367] Gardner T. S., Cantor C. R., Collins J. J. (2000). Construction
of
a genetic toggle switch in Escherichia coli. Nature.

[1368] Tan C., Marguet P., You L. (2009). Emergent bistability by a growth-modulating
positive feedback circuit. Nat Chem Biol.

[1369] Siuti P., Yazbek J., Lu T. K. (2013). Synthetic circuits
integrating logic and memory in living cells. Nature Biotechnology.

[1370] Yang L., Nielsen A. A. K., Fernandez-Rodriguez J., McClune C. J., Laub M. T., Lu T. K., Voigt C. A. (2014). Permanent
genetic memory with >1-byte capacity. Nature
Methods.

[1371] Bernabé-Orts J. M., Quijano-Rubio A., Vazquez-Vilar M., Mancheño-Bonillo J., Moles-Casas V., Selma S., Gianoglio S., Granell A., Orzaez D. (2020). A memory switch
for plant synthetic biology based on the phage ϕC31 integration
system. Nucleic Acids Research.

[1372] Fernandez-Rodriguez J., Yang L., Gorochowski T. E., Gordon D. B., Voigt C. A. (2015). Memory
and Combinatorial Logic Based
on DNA Inversions: Dynamics and Evolutionary Stability. ACS Synth Biol.

[1373] Ham T. S., Lee S. K., Keasling J. D., Arkin A. P. (2006). A tightly
regulated inducible expression system utilizing the fim inversion
recombination switch. Biotechnol Bioeng.

[1374] Bonnet J., Yin P., Ortiz M. E., Subsoontorn P., Endy D. (2013). Amplifying genetic
logic gates. Science.

[1375] Lear S. K., Shipman S. L. (2023). Molecular recording:
transcriptional
data collection into the genome. Curr Opin Biotechnol.

[1376] Bonnet J., Subsoontorn P., Endy D. (2012). Rewritable digital
data storage in live cells via engineered control of recombination
directionality. Proc Natl Acad Sci U S A.

[1377] Lloyd J. P. B., Ly F., Gong P., Pflueger J., Swain T., Pflueger C., Fourie E., Khan M. A., Kidd B. N., Lister R. (2022). Synthetic memory circuits for stable
cell reprogramming in plants. Nat Biotechnol.

[1378] Cordero T., Rosado A., Majer E., Jaramillo A., Rodrigo G., Daròs J.-A. (2018). Boolean
Computation in Plants Using
Post-translational Genetic Control and a Visual Output Signal. ACS Synth. Biol..

[1379] Feng, Y. ; Cao, C.-M. ; Vikram, M. ; Park, S. ; Kim, H. J. ; Hong, J. C. ; Cisneros-Zevallos, L. ; Koiwa, H. A Three-Component Gene Expression System and Its Application for Inducible Flavonoid Overproduction in Transgenic Arabidopsis thaliana. PLoS ONE 2011, 6 (3), e17603. 10.1371/journal.pone.0017603 21408135 PMC3050924

[1380] Liang Y.-C., Reid M. S., Jiang C.-Z. (2014). Controlling plant
architecture by manipulation of gibberellic acid signalling in petunia. Horticulture Research.

[1381] Okada R., Nemoto Y., Endo-Higashi N., Izawa T. (2017). Synthetic control of flowering in rice independent of the cultivation
environment. Nat Plants.

[1382] Lewis N. E., Nagarajan H., Palsson B. O. (2012). Constraining the
metabolic genotype-phenotype relationship using a phylogeny of in
silico methods. Nat Rev Microbiol.

[1383] Liu S., Yoder J. I. (2016). Chemical induction
of hairpin RNAi molecules to silence
vital genes in plant roots. Scientific Reports.

[1384] Mahfouz M. M., Li L. X., Piatek M., Fang X. Y., Mansour H., Bangarusamy D. K., Zhu J. K. (2012). Targeted transcriptional
repression using a chimeric TALE-SRDX repressor protein. Plant Molecular Biology.

[1385] Li Z., Zhang D., Xiong X., Yan B., Xie W., Sheen J., Li J. F. (2017). A potent Cas9-derived
gene activator
for plant and mammalian cells. Nat Plants.

[1386] Gupta M., DeKelver R. C., Palta A., Clifford C., Gopalan S., Miller J. C., Novak S., Desloover D., Gachotte D., Connell J. (2012). Transcriptional activation
of Brassica napus beta-ketoacyl-ACP synthase II with an engineered
zinc finger protein transcription factor. Plant
Biotechnol J.

[1387] Lee J. E., Neumann M., Duro D. I., Schmid M. (2019). CRISPR-based
tools for targeted transcriptional and epigenetic regulation in plants. PLoS One.

[1388] Lowder L. G., Zhou J., Zhang Y., Malzahn A., Zhong Z., Hsieh T. F., Voytas D. F., Zhang Y., Qi Y. (2018). Robust Transcriptional Activation
in Plants Using Multiplexed CRISPR-Act2.0
and mTALE-Act Systems. Mol Plant.

[1389] Jayanthi S., Nilgiriwala K. S., Del Vecchio D. (2013). Retroactivity
controls the temporal dynamics of gene transcription. ACS Synth Biol.

[1390] Chen S., Hofius D., Sonnewald U., Börnke F. (2003). Temporal and spatial control of gene silencing in transgenic
plants by inducible expression of double-stranded RNA. The Plant Journal.

[1391] Guo H. S., Fei J. F., Xie Q., Chua N. H. (2003). A chemical-regulated
inducible RNAi system in plants. Plant J.

[1392] Aman R., Ali Z., Butt H., Mahas A., Aljedaani F., Khan M. Z., Ding S., Mahfouz M. (2018). RNA virus
interference via CRISPR/Cas13a system in plants. Genome Biology.

[1393] Mahas A., Neal Stewart C., Mahfouz M. M. (2018). Harnessing CRISPR/Cas
systems for programmable transcriptional and post-transcriptional
regulation. Biotechnology Advances.

[1394] Otoupal P. B., Cress B. F., Doudna J. A., Schoeniger J. S. (2022). CRISPR-RNAa:
targeted activation of translation using dCas13 fusions to translation
initiation factors. Nucleic Acids Res.

[1395] Du M., Jillette N., Zhu J. J., Li S., Cheng A. W. (2020). CRISPR
artificial splicing factors. Nat Commun.

[1396] Johnson L. M., Du J., Hale C. J., Bischof S., Feng S., Chodavarapu R. K., Zhong X., Marson G., Pellegrini M., Segal D. J. (2014). SRA-and SET-domain-containing
proteins link RNA polymerase v occupancy to DNA methylation. Nature.

[1397] Gallego-Bartolomé J., Gardiner J., Liu W., Papikian A., Ghoshal B., Kuo H. Y., Zhao J. M. C., Segal D. J., Jacobsen S. E. (2018). Targeted DNA demethylation of the
arabidopsis genome using the human TET1 catalytic domain. P Natl Acad Sci USA.

[1398] Veley K. M., Elliott K., Jensen G., Zhong Z., Feng S., Yoder M., Gilbert K. B., Berry J. C., Lin Z. D., Ghoshal B. (2023). Improving
cassava bacterial
blight resistance by editing the epigenome. Nat Commun.

[1399] Papikian A., Liu W., Gallego-Bartolomé J., Jacobsen S. E. (2019). Site-specific manipulation
of Arabidopsis loci using
CRISPR-Cas9 SunTag systems. Nature Communications.

[1400] Barkan A., Rojas M., Fujii S., Yap A., Chong Y. S., Bond C. S., Small I. (2012). A combinatorial amino
acid code for RNA recognition by pentatricopeptide repeat proteins. PLoS Genet.

[1401] Rojas M., Yu Q., Williams-Carrier R., Maliga P., Barkan A. (2019). Engineered PPR proteins as inducible
switches to activate the expression of chloroplast transgenes. Nat Plants.

[1402] Yu Q., Barkan A., Maliga P. (2019). Engineered RNA-binding
protein for
transgene activation in non-green plastids. Nat Plants.

[1403] Carignano, A. ; Chen, D. H. ; Mallory, C. ; Wright, R. C. ; Seelig, G. ; Klavins, E. Modular, robust, and extendible multicellular circuit design in yeast. Elife 2022, 11, 10.7554/eLife.74540. PMC9000959 35312478

[1404] Auslander D., Auslander S., Pierrat X., Hellmann L., Rachid L., Fussenegger M. (2018). Programmable full-adder computations
in communicating three-dimensional cell cultures. Nat Methods.

[1405] Bashor C. J., Patel N., Choubey S., Beyzavi A., Kondev J., Collins J. J., Khalil A. S. (2019). Complex signal processing
in synthetic gene circuits using cooperative regulatory assemblies. Science.

[1406] Moon T. S., Clarke E. J., Groban E. S., Tamsir A., Clark R. M., Eames M., Kortemme T., Voigt C. A. (2011). Construction
of a genetic multiplexer to toggle between chemosensory pathways in
Escherichia coli. J Mol Biol.

[1407] Friedland A. E., Lu T. K., Wang X., Shi D., Church G., Collins J. J. (2009). Synthetic gene networks that count. Science.

[1408] Shin, J. ; Zhang, S. Y. ; Der, B. S. ; Nielsen, A. A. K. ; Voigt, C. A. Programming Escherichia coli to function as a digital display. Molecular Systems Biology 2020, 16 (3), 10.15252/msb.20199401. PMC7058928 32141239

[1409] Chen Z., Kibler R. D., Hunt A., Busch F., Pearl J., Jia M., VanAernum Z. L., Wicky B. I. M., Dods G., Liao H. (2020). De novo
design of protein logic gates. Science.

[1410] Chiang A. J., Hasty J. (2023). Design of synthetic bacterial biosensors. Curr
Opin Microbiol.

[1411] Piccinini L., Iacopino S., Cazzaniga S., Ballottari M., Giuntoli B., Licausi F. (2022). A synthetic switch
based on orange carotenoid protein to control blue-green light responses
in chloroplasts. Plant Physiol.

[1412] Verhounig A., Karcher D., Bock R. (2010). Inducible gene expression
from the plastid genome by a synthetic riboswitch. Proc Natl Acad Sci U S A.

[1413] Xu W., Li S., Bock R., Zhang J. (2024). A heat-inducible expression
system for external control of gene expression in plastids. Plant Biotechnol J.

[1414] Barkan A., Small I. (2014). Pentatricopeptide Repeat
Proteins
in Plants. Annual Review of Plant Biology.

[1415] Yin P., Li Q., Yan C., Liu Y., Liu J., Yu F., Wang Z., Long J., He J., Wang H. W. (2013). Structural basis for the modular recognition
of single-stranded RNA
by PPR proteins. Nature.

[1416] Pfalz J., Bayraktar O. A., Prikryl J., Barkan A. (2009). Site-specific
binding of a PPR protein defines and stabilizes 5 and 3 mRNA termini
in chloroplasts. EMBO J.

[1417] Opgenorth P., Costello Z., Okada T., Goyal G., Chen Y., Gin J., Benites V., de Raad M., Northen T. R., Deng K. (2019). Lessons
from Two Design-Build-Test-Learn
Cycles of Dodecanol Production in Escherichia coli Aided by Machine
Learning. ACS Synth Biol.

[1418] Chen K., Wang Y., Zhang R., Zhang H., Gao C. (2019). CRISPR/Cas Genome Editing and Precision
Plant Breeding in Agriculture. Annu Rev Plant
Biol.

[1419] McLaughlin J. A., Myers C. J., Zundel Z., Misirli G., Zhang M., Ofiteru I. D., Goni-Moreno A., Wipat A. (2018). SynBioHub: A Standards-Enabled
Design Repository for Synthetic Biology. ACS
Synth Biol.

[1420] Myers C. J., Beal J., Gorochowski T. E., Kuwahara H., Madsen C., McLaughlin J. A., Misirli G., Nguyen T., Oberortner E., Samineni M. (2017). A standard-enabled workflow for synthetic biology. Biochem Soc Trans.

[1421] Marchisio M. A., Stelling J. (2009). Computational design
tools for synthetic
biology. Curr Opin Biotechnol.

[1422] Wu K., Rao C. V. (2012). Computational methods
in synthetic biology: towards
computer-aided part design. Curr Opin Chem Biol.

[1423] Chandran D., Bergmann F. T., Sauro H. M., Densmore D. (2011). Computer-Aided
Design for Synthetic Biology. Design and Analysis
of Biomolecular Circuits:Engineering Approaches to Systems and Synthetic
Biology.

[1424] MacDonald J. T., Barnes C., Kitney R. I., Freemont P. S., Stan G. B. (2011). Computational design approaches and
tools for synthetic
biology. Integr Biol (Camb).

[1425] Choi K. R., Jang W. D., Yang D., Cho J. S., Park D., Lee S. Y. (2019). Systems Metabolic
Engineering Strategies:
Integrating Systems and Synthetic Biology with Metabolic Engineering. Trends Biotechnol.

[1426] Carbonell P., Radivojevic T., Garcia Martin H. (2019). Opportunities
at the Intersection of Synthetic Biology, Machine Learning, and Automation. ACS Synth Biol.

[1427] Welborn V. V., Head-Gordon T. (2019). Computational
Design of Synthetic
Enzymes. Chem Rev.

[1428] Naseri G., Koffas M. A. G. (2020). Application of
combinatorial optimization
strategies in synthetic biology. Nat Commun.

[1429] Lin G.-M., Warden-Rothman R., Voigt C. A. (2019). Retrosynthetic design
of metabolic pathways to chemicals not found in nature. Current Opinion in Systems Biology.

[1430] Coll, A. ; Wilson, M. L. ; Gruden, K. ; Peccoud, J. Rule-Based Design of Plant Expression Vectors Using GenoCAD. Plos One 2015, 10 (7), e0132502. 10.1371/journal.pone.0132502 26148190 PMC4492961

[1431] McCarthy, D. M. ; Medford, J. I. Quantitative and Predictive Genetic Parts for Plant Synthetic Biology. Frontiers in Plant Science 2020, 11, 10.3389/fpls.2020.512526. PMC7573182 33123175

[1432] Dubey K. K., Luke G. A., Knox C., Kumar P., Pletschke B. I., Singh P. K., Shukla P. (2018). Vaccine and
antibody
production in plants: developments and computational tools. Brief Funct Genomics.

[1433] Omony J., Nussbaumer T., Gutzat R. (2020). DNA methylation analysis
in plants: review of computational tools and future perspectives. Brief Bioinform.

[1434] Martinez M. (2016). Computational Tools for Genomic Studies
in Plants. Current Genomics.

[1435] Schaumberg K. A., Antunes M. S., Kassaw T. K., Xu W., Zalewski C. S., Medford J. I., Prasad A. (2016). Quantitative characterization
of genetic parts and circuits for plant synthetic biology. Nature Methods.

[1436] Navarro F. J., Baulcombe D. C. (2019). MiRNA-Mediated
Regulation of Synthetic
Gene Circuits in the Green Alga Chlamydomonas reinhardtii. ACS Synth. Biol..

[1437] Pfeiffer A., Kunkel T., Hiltbrunner A., Neuhaus G., Wolf I., Speth V., Adam E., Nagy F., Schäfer E. (2009). A cell-free
system for light-dependent
nuclear import of phytochrome. Plant Journal.

[1438] Smanski M. J., Bhatia S., Zhao D. H., Park Y., Woodruff L. B. A., Giannoukos G., Ciulla D., Busby M., Calderon J., Nicol R. (2014). Functional optimization
of gene clusters by combinatorial design and assembly. Nature Biotechnology.

[1439] Bhatia S. P., Smanski M. J., Voigt C. A., Densmore D. M. (2017). Genetic
Design via Combinatorial Constraint Specification. ACS Synth. Biol..

[1440] Chao R., Yuan Y., Zhao H. (2015). Building biological
foundries for next-generation synthetic biology. Sci China Life Sci.

[1441] Ham T. S., Dmytriv Z., Plahar H., Chen J., Hillson N. J., Keasling J. D. (2012). Design, implementation and practice
of JBEI-ICE: an open source biological part registry platform and
tools. Nucleic Acids Res.

[1442] Xia B., Bhatia S., Bubenheim B., Dadgar M., Densmore D., Anderson J. C. (2011). Developer's
and user's guide to Clotho v2.0 A software
platform for the creation of synthetic biological systems. Methods Enzymol.

[1443] Engler C., Kandzia R., Marillonnet S. (2008). A one pot,
one step, precision cloning method with high throughput capability. PLoS One.

[1444] Sarrion-Perdigones A., Falconi E. E., Zandalinas S. I., Juarez P., Fernandez-del-Carmen A., Granell A., Orzaez D. (2011). GoldenBraid: an iterative cloning system for standardized
assembly of reusable genetic modules. PLoS One.

[1445] Sarrion-Perdigones A., Vazquez-Vilar M., Palací J., Castelijns B., Forment J., Ziarsolo P., Blanca J., Granell A., Orzaez D. (2013). GoldenBraid 2.0: A
Comprehensive
DNA Assembly Framework for Plant Synthetic Biology. Plant Physiology.

[1446] Vrana J., de Lange O., Yang Y., Newman G., Saleem A., Miller A., Cordray C., Halabiya S., Parks M., Lopez E. (2021). Aquarium:
open-source
laboratory software for design, execution and data management. Synth Biol (Oxf).

[1447] Chen, Y.-J. ; Clancy, K. ; Voigt, C. Modeling Genetic Parts for Synthetic Biology. In Quantitative Biology: From Molecular to Cellular Systems; Wall, M. E. Ed.; CRC Press/Taylor & Francis Group, 2012.

[1448] Salis, H. M. Chapter two - The Ribosome Binding Site Calculator. In Methods in Enzymology; Voigt, C. Ed.; Vol. 498; Academic Press, 2011; pp 19-42. 10.1016/B978-0-12-385120-8.00002-4 21601672

[1449] Lee, S. Y. ; Sohn, S. B. ; Kim, Y. B. ; Shin, J. H. ; Kim, J. E. ; Kim, T. Y. Chapter 8 - Computational Methods for Strain Design. In Synthetic Biology; Zhao, H. Ed.; Academic Press, 2013; pp 141-156.

[1450] LaFleur T. L., Hossain A., Salis H. M. (2022). Automated model-predictive
design of synthetic promoters to control transcriptional profiles
in bacteria. Nat Commun.

[1451] Farasat I., Kushwaha M., Collens J., Easterbrook M., Guido M., Salis H. M. (2014). Efficient search,
mapping, and optimization
of multi-protein genetic systems in diverse bacteria. Mol Syst Biol.

[1452] Seo S. W., Yang J. S., Kim I., Yang J., Min B. E., Kim S., Jung G. Y. (2013). Predictive design
of mRNA translation initiation region to control prokaryotic translation
efficiency. Metab Eng.

[1453] Markley A. L., Begemann M. B., Clarke R. E., Gordon G. C., Pfleger B. F. (2015). Synthetic biology toolbox for controlling
gene expression
in the cyanobacterium Synechococcus sp. strain PCC 7002. ACS Synth Biol.

[1454] Ahmed F., Dai X., Zhao P. X. (2015). Bioinformatics
tools
for achieving better gene silencing in plants. Methods Mol Biol.

[1455] Ahmed F., Senthil-Kumar M., Dai X., Ramu V. S., Lee S., Mysore K. S., Zhao P. X. (2020). pssRNAit: A Web Server for Designing
Effective and Specific Plant siRNAs with Genome-Wide Off-Target Assessment. Plant Physiol.

[1456] Luck S., Kreszies T., Strickert M., Schweizer P., Kuhlmann M., Douchkov D. (2019). siRNA-Finder (si-Fi)
Software for RNAi-Target Design and Off-Target Prediction. Front Plant Sci.

[1457] Park Y. K., Park S. M., Choi Y. C., Lee D., Won M., Kim Y. J. (2008). AsiDesigner: exon-based siRNA design server considering
alternative splicing. Nucleic Acids Res.

[1458] Ahmed F., Raghava G. P. (2011). Designing of highly effective complementary
and mismatch siRNAs for silencing a gene. PLoS
One.

[1459] Vert J. P., Foveau N., Lajaunie C., Vandenbrouck Y. (2006). An accurate
and interpretable model for siRNA efficacy prediction. BMC Bioinformatics.

[1460] Ichihara M., Murakumo Y., Masuda A., Matsuura T., Asai N., Jijiwa M., Ishida M., Shinmi J., Yatsuya H., Qiao S. (2007). Thermodynamic instability
of siRNA duplex is a prerequisite for dependable prediction of siRNA
activities. Nucleic Acids Res.

[1461] Gustafsson C., Govindarajan S., Minshull J. (2004). Codon bias and heterologous
protein expression. Trends in Biotechnology.

[1462] Temme K., Zhao D., Voigt C. A. (2012). Refactoring the
nitrogen fixation gene cluster from Klebsiella oxytoca. Proceedings of the National Academy of Sciences.

[1463] Webster G. R., Teh A. Y. H., Ma J. K. C. (2017). Synthetic gene
design—The rationale for codon optimization and implications
for molecular pharming in plants. Biotechnol
Bioeng.

[1464] Perlak F. J., Fuchs R. L., Dean D. A., McPherson S. L., Fischhoff D. A. (1991). Modification of the coding sequence enhances plant
expression of insect control protein genes. P Natl Acad Sci USA.

[1465] Gaspar P., Oliveira J. L., Frommlet J., Santos M. A., Moura G. (2012). EuGene: maximizing synthetic gene
design for heterologous expression. Bioinformatics.

[1466] Chin J. X., Chung B. K., Lee D. Y. (2014). Codon Optimization
OnLine (COOL): a web-based multi-objective optimization platform for
synthetic gene design. Bioinformatics.

[1467] Guimaraes J. C., Rocha M., Arkin A. P., Cambray G. (2014). D-Tailor:
automated analysis and design of DNA sequences. Bioinformatics.

[1468] Gisby M. F., Mellors P., Madesis P., Ellin M., Laverty H., O'Kane S., Ferguson M. W., Day A. (2011). A synthetic
gene increases TGFbeta3 accumulation by 75-fold in tobacco chloroplasts
enabling rapid purification and folding into a biologically active
molecule. Plant Biotechnol J.

[1469] Kwon K. C., Chan H. T., Leon I. R., Williams-Carrier R., Barkan A., Daniell H. (2016). Codon Optimization
to Enhance Expression
Yields Insights into Chloroplast Translation. Plant Physiol.

[1470] Jackson M. A., Sternes P. R., Mudge S. R., Graham M. W., Birch R. G. (2014). Design rules for efficient transgene expression in
plants. Plant Biotechnol J.

[1471] Dahiyat B. I., Mayo S. L. (1997). De novo protein
design: fully automated
sequence selection. Science.

[1472] Kuhlman B., Dantas G., Ireton G. C., Varani G., Stoddard B. L., Baker D. (2003). Design of a novel globular
protein
fold with atomic-level accuracy. Science.

[1473] Kim D. E., Chivian D., Baker D. (2004). Protein structure prediction
and analysis using the Robetta server. Nucleic
Acids Res.

[1474] Li D., Ma Y., Zhou Y., Gou J., Zhong Y., Zhao L., Han L., Ovchinnikov S., Ma L., Huang S. (2019). A structural and data-driven approach to engineering
a plant cytochrome P450 enzyme. Sci China Life
Sci.

[1475] Ennist N. M., Wang S., Kennedy M. A., Curti M., Sutherland G. A., Vasilev C., Redler R. L., Maffeis V., Shareef S., Sica A. V. (2024). De novo design of proteins
housing excitonically coupled chlorophyll special pairs. Nat Chem Biol.

[1476] Dahiyat B. I., Mayo S. L. (1996). Protein design automation. Protein Sci.

[1477] Chen B., Lee H. L., Heng Y. C., Chua N., Teo W. S., Choi W. J., Leong S. S. J., Foo J. L., Chang M. W. (2018). Synthetic biology toolkits and applications in Saccharomyces
cerevisiae. Biotechnology Advances.

[1478] Poolman M. G., Miguet L., Sweetlove L. J., Fell D. A. (2009). A genome-scale metabolic
model of Arabidopsis and some
of its properties. Plant Physiol.

[1479] de Oliveira Dal'Molin C. G., Quek L. E., Palfreyman R. W., Brumbley S. M., Nielsen L. K. (2010). AraGEM,
a genome-scale reconstruction
of the primary metabolic network in Arabidopsis. Plant Physiol.

[1480] Amirzakaria J. Z., Marashi S. A., Malboobi M. A., Lohrasebi T., Forouzan E. (2022). Critical assessment of genome-scale metabolic models
of Arabidopsis thaliana. Mol Omics.

[1481] Seaver S. M., Bradbury L. M., Frelin O., Zarecki R., Ruppin E., Hanson A. D., Henry C. S. (2015). Improved
evidence-based
genome-scale metabolic models for maize leaf, embryo, and endosperm. Front Plant Sci.

[1482] Simons M., Saha R., Amiour N., Kumar A., Guillard L., Clement G., Miquel M., Li Z. N., Mouille G., Lea P. J. (2014). Assessing the Metabolic
Impact of Nitrogen Availability Using a Compartmentalized Maize Leaf
Genome-Scale Model. Plant Physiology.

[1483] Bogart E., Myers C. R. (2016). Multiscale Metabolic Modeling of
C4 Plants: Connecting Nonlinear Genome-Scale Models to Leaf-Scale
Metabolism in Developing Maize Leaves. PLoS
One.

[1484] Saha, R. ; Suthers, P. F. ; Maranas, C. D. Zea mays iRS1563: A Comprehensive Genome-Scale Metabolic Reconstruction of Maize Metabolism. Plos One 2011, 6 (7), e21784. 10.1371/journal.pone.0021784 21755001 PMC3131064

[1485] Dal'Molin C. G., Quek L. E., Palfreyman R. W., Brumbley S. M., Nielsen L. K. (2010). C4GEM, a genome-scale metabolic model
to study C4 plant metabolism. Plant Physiol.

[1486] Grafahrend-Belau E., Schreiber F., Koschutzki D., Junker B. H. (2009). Flux balance analysis
of barley seeds: a computational
approach to study systemic properties of central metabolism. Plant Physiol.

[1487] Lakshmanan M., Zhang Z., Mohanty B., Kwon J. Y., Choi H. Y., Nam H. J., Kim D. I., Lee D. Y. (2013). Elucidating
rice cell metabolism under flooding and drought stresses using flux-based
modeling and analysis. Plant Physiol.

[1488] Poolman M. G., Kundu S., Shaw R., Fell D. A. (2013). Responses
to Light Intensity in a Genome-Scale Model of Rice Metabolism. Plant Physiology.

[1489] Pilalis E., Chatziioannou A., Thomasset B., Kolisis F. (2011). An in silico compartmentalized metabolic
model of Brassica
napus enables the systemic study of regulatory aspects of plant central
metabolism. Biotechnol Bioeng.

[1490] Hay J., Schwender J. (2011). Metabolic network reconstruction and flux variability
analysis of storage synthesis in developing oilseed rape (Brassica
napus L.) embryos. Plant J.

[1491] Hay J., Schwender J. (2011). Computational
analysis of storage synthesis in developing
Brassica napus L. (oilseed rape) embryos: flux variability analysis
in relation to (1)(3)C metabolic flux analysis. Plant J.

[1492] Yuan H., Cheung C. Y., Poolman M. G., Hilbers P. A., van Riel N. A. (2016). A genome-scale metabolic network
reconstruction of
tomato (Solanum lycopersicum L.) and its application to photorespiratory
metabolism. Plant J.

[1493] Thiele I., Palsson B. O. (2010). A protocol for generating
a high-quality
genome-scale metabolic reconstruction. Nat Protoc.

[1494] Zamani Amirzakaria J., Marashi S. A., Malboobi M. A., Lohrasebi T., Forouzan E. (2022). Critical assessment of genome-scale metabolic models
of Arabidopsis thaliana. Mol Omics.

[1495] Orth J. D., Thiele I., Palsson B. O. (2010). What is
flux balance
analysis?. Nat Biotechnol.

[1496] Heirendt L., Arreckx S., Pfau T., Mendoza S. N., Richelle A., Heinken A., Haraldsdottir H. S., Wachowiak J., Keating S. M., Vlasov V. (2019). Creation
and analysis of biochemical constraint-based models using the COBRA
Toolbox v.3.0. Nat Protoc.

[1497] Pharkya P., Maranas C. D. (2006). An optimization
framework for identifying
reaction activation/inhibition or elimination candidates for overproduction
in microbial systems. Metab Eng.

[1498] Pharkya P., Burgard A. P., Maranas C. D. (2004). OptStrain:
a computational
framework for redesign of microbial production systems. Genome Res.

[1499] Burgard A. P., Pharkya P., Maranas C. D. (2003). Optknock:
a bilevel
programming framework for identifying gene knockout strategies for
microbial strain optimization. Biotechnol Bioeng.

[1500] diCenzo G. C., Tesi M., Pfau T., Mengoni A., Fondi M. (2020). Genome-scale
metabolic reconstruction of the symbiosis between a
leguminous plant and a nitrogen-fixing bacterium. Nat Commun.

[1501] Basler, G. ; Kuken, A. ; Fernie, A. R. ; Nikoloski, Z. Photorespiratory Bypasses lead to increased growth in Arabidopsis thaliana: are Predictions consistent with experimental evidence?. Front Bioeng Biotech 2016, 4, 10.3389/fbioe.2016.00031. PMC4823303 27092301

[1502] Shameer S., Baghalian K., Cheung C. Y. M., Ratcliffe R. G., Sweetlove L. J. (2018). Computational
analysis of the productivity potential
of CAM. Nat Plants.

[1503] Terzer M., Stelling J. (2008). Large-scale computation
of elementary
flux modes with bit pattern trees. Bioinformatics.

[1504] von Kamp A., Schuster S. (2006). Metatool 5.0: fast and flexible elementary
modes analysis. Bioinformatics.

[1505] Rohwer J. M. (2012). Kinetic
modelling of plant metabolic pathways. J Exp
Bot.

[1506] Schwender J., Goffman F., Ohlrogge J. B., Shachar-Hill Y. (2004). Rubisco without
the Calvin cycle improves the carbon efficiency of developing green
seeds. Nature.

[1507] Chandran D., Bergmann F. T., Sauro H. M. (2009). TinkerCell:
modular
CAD tool for synthetic biology. J Biol Eng.

[1508] Beal J., Lu T., Weiss R. (2011). Automatic
compilation
from high-level biologically-oriented programming language to genetic
regulatory networks. PLoS One.

[1509] Marchisio M. A., Stelling J. (2011). Automatic design of
digital synthetic
gene circuits. PLoS Comput Biol.

[1510] Huynh L., Tsoukalas A., Koppe M., Tagkopoulos I. (2013). SBROME: a
scalable optimization and module matching framework for automated
biosystems design. ACS Synth Biol.

[1511] Davidsohn N., Beal J., Kiani S., Adler A., Yaman F., Li Y., Xie Z., Weiss R. (2015). Accurate predictions
of genetic circuit behavior from part characterization and modular
composition. ACS Synth Biol.

[1512] Dupuy L., Mackenzie J., Rudge T., Haseloff J. (2008). A system for
modelling cell-cell interactions during plant morphogenesis. Ann Bot.

[1513] Merks R. M., Guravage M., Inze D., Beemster G. T. (2011). VirtualLeaf:
an open-source framework for cell-based modeling of plant tissue growth
and development. Plant Physiol.

[1514] Drewry, D. T. ; Kumar, P. ; Long, S. ; Bernacchi, C. ; Liang, X. Z. ; Sivapalan, M. Ecohydrological responses of dense canopies to environmental variability: 2. Role of acclimation under elevated CO2. J Geophys Res-Biogeo 2010, 115, 10.1029/2010JG001340.

[1515] Lynch J. P., Nielsen K. L., Davis R. D., Jablokow A. G. (1997). SimRoot:
Modelling and visualization of root systems. Plant Soil.

[1516] Postma J. A., Kuppe C., Owen M. R., Mellor N., Griffiths M., Bennett M. J., Lynch J. P., Watt M. (2017). OPENSIMROOT:
widening the scope and application of root architectural models. New Phytologist.

[1517] Dal'Molin, C. G. D. ; Quek, L. E. ; Saa, P. A. ; Nielsen, L. K. A multi-tissue genome-scale metabolic modeling framework for the analysis of whole plant systems. Frontiers in Plant Science 2015, 6, 10.3389/fpls.2015.00004. PMC4302846 25657653

[1518] Thirumalaikumar V. P., Devkar V., Mehterov N., Ali S., Ozgur R., Turkan I., Mueller-Roeber B., Balazadeh S. (2018). NAC transcription factor JUNGBRUNNEN1 enhances drought
tolerance in tomato. Plant Biotechnol J.

[1519] Scheunemann, M. ; Brady, S. M. ; Nikoloski, Z. Integration of large-scale data for extraction of integrated Arabidopsis root cell-type specific models. Scientific Reports 2018, 8, 10.1038/s41598-018-26232-8. PMC5962614 29784955

[1520] Chew Y. H., Wenden B., Flis A., Mengin V., Taylor J., Davey C. L., Tindal C., Thomas H., Ougham H. J., de Reffye P. (2014). Multiscale digital Arabidopsis
predicts individual organ and whole-organism growth. P Natl Acad Sci USA.

[1521] Marshall-Colon, A. ; Long, S. P. ; Allen, D. K. ; Allen, G. ; Beard, D. A. ; Benes, B. ; von Caemmerer, S. ; Christensen, A. J. ; Cox, D. J. ; Hart, J. C. ; Crops In Silico: Generating Virtual Crops Using an Integrative and Multi-scale Modeling Platform. Frontiers in Plant Science 2017, 8, 10.3389/fpls.2017.00786. PMC5430029 28555150

[1522] Sartor R. C., Noshay J., Springer N. M., Briggs S. P. (2019). Identification
of the expressome by machine learning on omics data. Proc Natl Acad Sci U S A.

[1523] Liu Q., Liang Z., Feng D., Jiang S., Wang Y., Du Z., Li R., Hu G., Zhang P., Ma Y. (2021). Transcriptional landscape
of rice roots at the single-cell resolution. Mol Plant.

[1524] Mahood E. H., Kruse L. H., Moghe G. D. (2020). Machine learning:
A powerful tool for gene function prediction in plants. Appl Plant Sci.

[1525] Do, H. ; Than, K. ; Larmande, P. Evaluating Named-Entity Recognition Approaches in Plant Molecular Biology. In Multi-disciplinary Trends in Artificial Intelligence, Cham, 2018; Kaenampornpan, M. ; Malaka, R. ; Nguyen, D. D. ; Schwind, N. , Eds.; Springer International Publishing: pp 219-225.

[1526] Umarov, R. K. ; Solovyev, V. V. Recognition of prokaryotic and eukaryotic promoters using convolutional deep learning neural networks. Plos One 2017, 12 (2), e0171410. 10.1371/journal.pone.0171410 28158264 PMC5291440

[1527] Yang C., Yang L. S., Zhou M., Xie H. L., Zhang C. J., Wang M., Zhu H. Q. (2018). LncADeep:
an ab
initio lncRNA identification and functional annotation tool based
on deep learning. Bioinformatics.

[1528] Meng, J. ; Kang, Q. ; Chang, Z. ; Luan, Y. S. PlncRNA-HDeep: plant long noncoding RNA prediction using hybrid deep learning based on two encoding styles. Bmc Bioinformatics 2021, 22 (Suppl 3), 10.1186/s12859-020-03870-2. PMC8114701 33980138

[1529] Leung M. K. K., Xiong H. Y., Lee L. J., Frey B. J. (2014). Deep learning
of the tissue-regulated splicing code. Bioinformatics.

[1530] Kotopka B. J., Smolke C. D. (2020). Model-driven generation of artificial
yeast promoters. Nat Commun.

[1531] Cuperus J. T., Groves B., Kuchina A., Rosenberg A. B., Jojic N., Fields S., Seelig G. (2017). Deep learning
of the
regulatory grammar of yeast 5 untranslated regions from 500,000 random
sequences. Genome Res.

[1532] Ding W., Cheng J., Guo D., Mao L., Li J., Lu L., Zhang Y., Yang J., Jiang H. (2018). Engineering
the 5 UTR-Mediated Regulation of Protein Abundance in Yeast Using
Nucleotide Sequence Activity Relationships. ACS Synth Biol.

[1533] Sample P. J., Wang B., Reid D. W., Presnyak V., McFadyen I. J., Morris D. R., Seelig G. (2019). Human 5 UTR
design
and variant effect prediction from a massively parallel translation
assay. Nat Biotechnol.

[1534] DeChant C., Wiesner-Hanks T., Chen S., Stewart E. L., Yosinski J., Gore M. A., Nelson R. J., Lipson H. (2017). Automated
Identification of Northern Leaf Blight-Infected Maize Plants from
Field Imagery Using Deep Learning. Phytopathology.

[1535] Fuentes, A. ; Yoon, S. ; Kim, S. C. ; Park, D. S. A Robust Deep-Learning-Based Detector for Real-Time Tomato Plant Diseases and Pests Recognition. Sensors (Basel) 2017, 17 (9), 2022 10.3390/s17092022. 28869539 PMC5620500

[1536] Tran, T. T. ; Choi, J. W. ; Le, T. T. H. ; Kim, J. W. A Comparative Study of Deep CNN in Forecasting and Classifying the Macronutrient Deficiencies on Development of Tomato Plant. Appl Sci-Basel 2019, 9 (8), 1601. 10.3390/app9081601

[1537] Chen, Y. ; Lee, W. S. ; Gan, H. ; Peres, N. ; Fraisse, C. ; Zhang, Y. C. ; He, Y. Strawberry Yield Prediction Based on a Deep Neural Network Using High-Resolution Aerial Orthoimages. Remote Sensing 2019, 11 (13), 1584. 10.3390/rs11131584

[1538] Yang, W. ; Nigon, T. ; Hao, Z. Y. ; Paiao, G. D. ; Fernandez, F. G. ; Mulla, D. ; Yang, C. Estimation of corn yield based on hyperspectral imagery and convolutional neural network. Comput Electron Agr 2021, 184, 106092. 10.1016/j.compag.2021.106092

[1539] Namin, S. T. ; Esmaeilzadeh, M. ; Najafi, M. ; Brown, T. B. ; Borevitz, J. O. Deep phenotyping: deep learning for temporal phenotype/genotype classification. Plant Methods 2018, 14, 10.1186/s13007-018-0333-4. PMC6076396 30087695

[1540] Ubbens, J. R. ; Stavness, I. Deep Plant Phenomics: A Deep Learning Platform for Complex Plant Phenotyping Tasks. Frontiers in Plant Science 2017, 8, 10.3389/fpls.2017.01190. PMC5500639 28736569

[1541] Pound, M. P. ; Atkinson, J. A. ; Townsend, A. J. ; Wilson, M. H. ; Griffiths, M. ; Jackson, A. S. ; Bulat, A. ; Tzimiropoulos, G. ; Wells, D. M. ; Murchie, E. H. ; Deep machine learning provides state-of-the-art performance in image-based plant phenotyping. Gigascience 2017, 6 (10), 10.1093/gigascience/gix083. PMC5632296 29020747

[1542] Pound M. P., Atkinson J. A., Wells D. M., Pridmore T. P., French A. P. (2017). Deep Learning for Multi-task Plant Phenotyping. IEEE Int Conf Comp V.

[1543] Wang T., Rostamza M., Song Z. H., Wang L. J., McNickle G., Iyer-Pascuzzi A. S., Qiu Z. J., Jin J. (2019). SegRoot: A
high throughput segmentation method for root image analysis. Comput Electron Agr.

[1544] Yasrab, R. ; Atkinson, J. A. ; Wells, D. M. ; French, A. P. ; Pridmore, T. P. ; Pound, M. P. RootNav 2.0: Deep learning for automatic navigation of complex plant root architectures. Gigascience 2019, 8 (11), 10.1093/gigascience/giz123. PMC6839032 31702012

[1545] Alipanahi B., Delong A., Weirauch M. T., Frey B. J. (2015). Predicting
the sequence specificities of DNA- and RNA-binding proteins by deep
learning. Nat Biotechnol.

[1546] Uygun S., Azodi C. B., Shiu S. H. (2019). Cis-Regulatory
Code
for Predicting Plant Cell-Type Transcriptional Response to High Salinity(1)([OPEN]). Plant Physiology.

[1547] Gao X., Zhang J., Wei Z., Hakonarson H. (2018). DeepPolyA:
A Convolutional Neural Network Approach for Polyadenylation Site Prediction. Ieee Access.

[1548] Mejia-Guerra, M. K. ; Buckler, E. S. A k-mer grammar analysis to uncover maize regulatory architecture. BMC Plant Biol 2019, 19, 10.1186/s12870-019-1693-2. PMC6419808 30876396

[1549] Kelley D. R., Snoek J., Rinn J. L. (2016). Basset:
learning
the regulatory code of the accessible genome with deep convolutional
neural networks. Genome Res.

[1550] Wang M., Tai C., E W., Wei L. (2018). DeFine: deep convolutional
neural networks accurately quantify intensities of transcription factor-DNA
binding and facilitate evaluation of functional non-coding variants. Nucleic Acids Res.

[1551] Greenside P., Shimko T., Fordyce P., Kundaje A. (2018). Discovering
epistatic feature interactions from neural network models of regulatory
DNA sequences. Bioinformatics.

[1552] Zhou J., Theesfeld C. L., Yao K., Chen K. M., Wong A. K., Troyanskaya O. G. (2018). Deep learning sequence-based ab initio
prediction of variant effects on expression and disease risk. Nat Genet.

[1553] Zhou J., Troyanskaya O. G. (2015). Predicting
effects of noncoding variants
with deep learning-based sequence model. Nat
Methods.

[1554] Zhao H., Tu Z., Liu Y., Zong Z., Li J., Liu H., Xiong F., Zhan J., Hu X., Xie W. (2021). PlantDeepSEA,
a deep learning-based web service to predict the regulatory
effects of genomic variants in plants. Nucleic
Acids Res.

[1555] Huynh-Thu, V. A. ; Irrthum, A. ; Wehenkel, L. ; Geurts, P. Inferring regulatory networks from expression data using tree-based methods. PLoS One 2010, 5 (9), e12776 10.1371/journal.pone.0012776. 20927193 PMC2946910

[1556] Moore B. M., Wang P., Fan P., Lee A., Leong B., Lou Y. R., Schenck C. A., Sugimoto K., Last R., Lehti-Shiu M. D. (2020). Within- and cross-species
predictions of plant specialized metabolism genes using transfer learning. In Silico Plants.

[1557] Toubiana D., Puzis R., Wen L., Sikron N., Kurmanbayeva A., Soltabayeva A., Del Mar Rubio Wilhelmi M., Sade N., Fait A., Sagi M. (2019). Combined
network analysis and machine learning allows the prediction of metabolic
pathways from tomato metabolomics data. Commun
Biol.

[1558] Draper J., Mur L. A., Jenkins G., Ghosh-Biswas G. C., Bablak P., Hasterok R., Routledge A. P. (2001). Brachypodium
distachyon. A new model system for functional genomics in grasses. Plant Physiol.

[1559] International Barley Genome Sequencing C., Mayer K. F., Waugh R., Brown J. W., Schulman A., Langridge P., Platzer M., Fincher G. B., Muehlbauer G. J., Sato K. (2012). A physical, genetic and functional sequence assembly
of the barley genome. Nature.

[1560] Ling H. Q., Ma B., Shi X., Liu H., Dong L., Sun H., Cao Y., Gao Q., Zheng S., Li Y. (2018). Genome sequence of the
progenitor of wheat A subgenome Triticum urartu. Nature.

[1561] McCaw M. E., Wallace J. G., Albert P. S., Buckler E. S., Birchler J. A. (2016). Fast-Flowering Mini-Maize: Seed to Seed in 60 Days. Genetics.

[1562] Huang, P. ; Shyu, C. ; Coelho, C. P. ; Cao, Y. Y. ; Brutnell, T. P. Setaria viridis as a Model System to Advance Millet Genetics and Genomics. Frontiers in Plant Science 2016, 7, 10.3389/fpls.2016.01781. PMC5124564 27965689

[1563] Schaefer D. G., Zryd J. P. (1997). Efficient gene targeting
in the moss
Physcomitrella patens. Plant J.

[1564] Reski R., Bae H., Simonsen H. T. (2018). Physcomitrella patens,
a versatile synthetic biology chassis. Plant
Cell Rep.

[1565] Zhan, X. ; Zhang, Y. H. ; Chen, D. F. ; Simonsen, H. T. Metabolic engineering of the moss Physcomitrella patens to produce the sesquiterpenoids patchoulol and alpha/beta-santalene. Frontiers in Plant Science 2014, 5, 10.3389/fpls.2014.00636. PMC4235272 25477891

[1566] King B. C., Vavitsas K., Ikram N. K., Schroder J., Scharff L. B., Bassard J. E., Hamberger B., Jensen P. E., Simonsen H. T. (2016). In vivo assembly of DNA-fragments
in the moss, Physcomitrella patens. Sci Rep.

[1567] Khairul Ikram N. K. B., Beyraghdar Kashkooli A., Peramuna A. V., van der Krol A. R., Bouwmeester H., Simonsen H. T. (2017). Stable Production
of the Antimalarial Drug Artemisinin in the Moss Physcomitrella patens. Front Bioeng Biotechnol.

[1568] Banerjee A., Arnesen J. A., Moser D., Motsa B. B., Johnson S. R., Hamberger B. (2019). Engineering modular diterpene biosynthetic
pathways in Physcomitrella patens. Planta.

[1569] Bowman J. L., Kohchi T., Yamato K. T., Jenkins J., Shu S. Q., Ishizaki K., Yamaoka S., Nishihama R., Nakamura Y., Berger F. (2017). Insights into Land Plant
Evolution Garnered from the Marchantia polymorpha Genome. Cell.

[1570] Frangedakis E., Guzman-Chavez F., Rebmann M., Markel K., Yu Y., Perraki A., Tse S. W., Liu Y., Rever J., Sauret-Gueto S. (2021). Construction of DNA Tools for Hyperexpression
in Marchantia Chloroplasts. ACS Synth Biol.

[1571] Delmans M., Pollak B., Haseloff J. (2017). MarpoDB: An Open Registry
for Marchantia Polymorpha Genetic Parts. Plant
Cell Physiol.

[1572] Sauret-Gueto S., Frangedakis E., Silvestri L., Rebmann M., Tomaselli M., Markel K., Delmans M., West A., Patron N. J., Haseloff J. (2020). Systematic Tools for
Reprogramming Plant Gene Expression in a Simple Model, Marchantia
polymorpha. ACS Synth Biol.

[1573] Tse S. W., Annese D., Romani F., Guzman-Chavez F., Bonter I., Forestier E., Frangedakis E., Haseloff J. (2024). Optimizing Promoters and Subcellular
Localization for
Constitutive Transgene Expression in Marchantia polymorpha. Plant Cell Physiol.

[1574] Yu Z., Boehm C. R., Hibberd J. M., Abell C., Haseloff J., Burgess S. J., Reyna-Llorens I. (2018). Droplet-based
microfluidic analysis
and screening of single plant cells. PLoS One.

[1575] Hellwig S., Drossard J., Twyman R. M., Fischer R. (2004). Plant cell
cultures for the production of recombinant proteins. Nat Biotechnol.

[1576] Brandizzi, F. ; Irons, S. ; Kearns, A. ; Hawes, C. BY-2 cells: culture and transformation for live cell imaging. Curr Protoc Cell Biol 2003, 19, 10.1002/0471143030.cb0107s19. 18228413

[1577] Shaaltiel Y., Bartfeld D., Hashmueli S., Baum G., Brill-Almon E., Galili G., Dym O., Boldin-Adamsky S. A., Silman I., Sussman J. L. (2007). Production
of glucocerebrosidase with terminal mannose glycans for enzyme replacement
therapy of Gaucher's disease using a plant cell system. Plant Biotechnol J.

[1578] Kim T. G., Baek M. Y., Lee E. K., Kwon T. H., Yang M. S. (2008). Expression of human growth hormone
in transgenic rice
cell suspension culture. Plant Cell Rep.

[1579] Lu C. H., Engelmann N. J., Lila M. A., Erdman J. W. (2008). Optimization of lycopene extraction from tomato cell
suspension culture by response surface methodology. J Agric Food Chem.

[1580] Dalkin K., Edwards R., Edington B., Dixon R. A. (1990). Stress
Responses in Alfalfa (Medicago sativa L.): I. Induction of Phenylpropanoid
Biosynthesis and Hydrolytic Enzymes in Elicitor-Treated Cell Suspension
Cultures. Plant Physiol.

[1581] Smith M. L., Mason H. S., Shuler M. L. (2002). Hepatitis
B surface
antigen (HBsAg) expression in plant cell culture: Kinetics of antigen
accumulation in batch culture and its intracellular form. Biotechnol Bioeng.

[1582] Sajid Z. A., Aftab F. (2016). An efficient method
for the establishment
of cell suspension cultures in potato (Solanum tuberosum L.). Pak. J. Bot.

[1583] Rasche S., Herwartz D., Schuster F., Jablonka N., Weber A., Fischer R., Schillberg S. (2016). More for less:
Improving the biomass yield of a pear cell suspension culture by design
of experiments. Sci Rep.

[1584] Konczak-Islam I., Okuno S., Yoshimoto M., Yamakawa O. (2003). Composition of phenolics
and anthocyanins in a sweet
potato cell suspension culture. Biochemical
Engineering Journal.

[1585] Iantcheva A., Revalska M., Zehirov G., Vassileva V. (2014). Agrobacterium-mediated
transformation of Medicago truncatula cell suspension culture provides
a system for functional analysis. In Vitro Cellular
& Developmental Biology-Plant.

[1586] Rademacher T., Sack M., Blessing D., Fischer R., Holland T., Buyel J. (2019). Plant cell packs: a
scalable platform
for recombinant protein production and metabolic engineering. Plant Biotechnol J.

[1587] Wu T., Kerbler S. M., Fernie A. R., Zhang Y. (2021). Plant cell cultures
as heterologous bio-factories for secondary metabolite production. Plant Commun.

[1588] Santos R. B., Abranches R., Fischer R., Sack M., Holland T. (2016). Putting the Spotlight Back on Plant Suspension Cultures. Front Plant Sci.

[1589] Ochoa-Villarreal M., Howat S., Hong S., Jang M. O., Jin Y. W., Lee E. K., Loake G. J. (2016). Plant cell culture
strategies for the production of natural products. BMB Rep.

[1590] Pistelli L., Giovannini A., Ruffoni B., Bertoli A., Pistelli L. (2010). Hairy root cultures for secondary metabolites production. Bio-farms for nutraceuticals: functional food and safety control
by biosensors.

[1591] Irigoyen S., Ramasamy M., Pant S., Niraula P., Bedre R., Gurung M., Rossi D., Laughlin C., Gorman Z., Achor D. (2020). Plant hairy roots enable
high throughput identification of antimicrobials against Candidatus
Liberibacter spp. Nat Commun.

[1592] Kai G., Xu H., Zhou C., Liao P., Xiao J., Luo X., You L., Zhang L. (2011). Metabolic engineering tanshinone
biosynthetic pathway in Salvia miltiorrhiza hairy root cultures. Metab Eng.

[1593] Sarkar M. A. R., Watanabe S., Suzuki A., Hashimoto F., Anai T. (2019). Identification of novel MYB transcription factors involved in the
isoflavone biosynthetic pathway by using the combination screening
system with agroinfiltration and hairy root transformation. Plant Biotechnology.

[1594] Li X., Patena W., Fauser F., Jinkerson R. E., Saroussi S., Meyer M. T., Ivanova N., Robertson J. M., Yue R., Zhang R. (2019). A genome-wide
algal mutant library and functional
screen identifies genes required for eukaryotic photosynthesis. Nat Genet.

[1595] Fauser F., Vilarrasa-Blasi J., Onishi M., Ramundo S., Patena W., Millican M., Osaki J., Philp C., Nemeth M., Salome P. A. (2022). Systematic characterization
of gene function in the photosynthetic alga Chlamydomonas reinhardtii. Nat Genet.

[1596] Lauersen K. J., Wichmann J., Baier T., Kampranis S. C., Pateraki I., Moller B. L., Kruse O. (2018). Phototrophic production
of heterologous diterpenoids and a hydroxy-functionalized derivative
from Chlamydomonas reinhardtii. Metab Eng.

[1597] Jackson H. O., Taunt H. N., Mordaka P. M., Smith A. G., Purton S. (2021). The Algal Chloroplast as a Testbed
for Synthetic Biology
Designs Aimed at Radically Rewiring Plant Metabolism. Front Plant Sci.

[1598] Specht E. A., Mayfield S. P. (2013). Synthetic oligonucleotide libraries
reveal novel regulatory elements in Chlamydomonas chloroplast mRNAs. ACS Synth Biol.

[1599] Rasala B. A., Muto M., Sullivan J., Mayfield S. P. (2011). Improved
heterologous protein expression in the chloroplast of Chlamydomonas
reinhardtii through promoter and 5′ untranslated region optimization. Plant biotechnology journal.

[1600] Tiller N., Bock R. (2014). The translational apparatus
of plastids
and its role in plant development. Mol Plant.

[1601] Dlugosz, E. M. ; Lenaghan, S. C. ; Stewart, C. N., Jr . A Robotic Platform for High-throughput Protoplast Isolation and Transformation. J Vis Exp 2016, (115), 10.3791/54300. PMC5092064 27768035

[1602] Lenaghan S. C., Neal Stewart C. (2019). An Automated
Protoplast Transformation System. Methods Mol
Biol.

[1603] Lin C. S., Hsu C. T., Yuan Y. H., Zheng P. X., Wu F. H., Cheng Q. W., Wu Y. L., Wu T. L., Lin S., Yue J. J. (2022). DNA-free CRISPR-Cas9 gene editing of wild tetraploid
tomato Solanum peruvianum using protoplast regeneration. Plant Physiol.

[1604] Sheen J. (2001). Signal transduction in maize and
Arabidopsis mesophyll protoplasts. Plant Physiol.

[1605] Wang S., Li E., Porth I., Chen J. G., Mansfield S. D., Douglas C. J. (2014). Regulation of secondary cell wall
biosynthesis by poplar R2R3 MYB transcription factor PtrMYB152 in
Arabidopsis. Sci Rep.

[1606] Wang X. D., Nolan K. E., Irwanto R. R., Sheahan M. B., Rose R. J. (2011). Ontogeny
of embryogenic callus in Medicago truncatula:
the fate of the pluripotent and totipotent stem cells. Ann Bot.

[1607] Otvos K., Pasternak T. P., Miskolczi P., Domoki M., Dorjgotov D., Szucs A., Bottka S., Dudits D., Feher A. (2005). Nitric oxide
is required for, and
promotes auxin-mediated activation of, cell division and embryogenic
cell formation but does not influence cell cycle progression in alfalfa
cell cultures. Plant J.

[1608] Pasternak, T. ; Lystvan, K. ; Betekhtin, A. ; Hasterok, R. From Single Cell to Plants: Mesophyll Protoplasts as a Versatile System for Investigating Plant Cell Reprogramming. Int J Mol Sci 2020, 21 (12), 4195 10.3390/ijms21124195 32545519 PMC7348876

[1609] Dai, X. ; Zhang, S. ; Liu, S. ; Qi, H. ; Duan, X. ; Han, Z. ; Wang, J. Functional Characterization and Phenotyping of Protoplasts on a Microfluidics-Based Flow Cytometry. Biosensors (Basel) 2022, 12 (9), 688 10.3390/bios12090688. 36140072 PMC9496511

[1610] Gonzalez-Garcia, M. P. ; Bustillo-Avendano, E. ; Sanchez-Corrionero, A. ; Del Pozo, J. C. ; Moreno-Risueno, M. A. Fluorescence-Activated Cell Sorting Using the D-Root Device and Optimization for Scarce and/or Non-Accessible Root Cell Populations. Plants (Basel) 2020, 9 (4), 499 10.3390/plants9040499. 32295129 PMC7238278

[1611] Perez, J. G. ; Stark, J. C. ; Jewett, M. C. Cell-Free Synthetic Biology: Engineering Beyond the Cell. Cold Spring Harb Perspect Biol 2016, 8 (12), a023853 10.1101/cshperspect.a023853. 27742731 PMC5131772

[1612] Silverman A. D., Karim A. S., Jewett M. C. (2020). Cell-free gene expression:
an expanded repertoire of applications. Nat
Rev Genet.

[1613] Shimizu Y., Inoue A., Tomari Y., Suzuki T., Yokogawa T., Nishikawa K., Ueda T. (2001). Cell-free translation
reconstituted with purified components. Nat
Biotechnol.

[1614] Sharpes C. E., McManus J. B., Blum S. M., Mgboji G. E., Lux M. W. (2021). Assessment
of Colorimetric Reporter Enzymes in the
PURE System. ACS Synth Biol.

[1615] Garamella J., Marshall R., Rustad M., Noireaux V. (2016). The All E.
coli TX-TL Toolbox 2.0: A Platform for Cell-Free Synthetic Biology. ACS Synth Biol.

[1616] Casini A., Chang F. Y., Eluere R., King A. M., Young E. M., Dudley Q. M., Karim A., Pratt K., Bristol C., Forget A. (2018). A Pressure
Test to Make
10 Molecules in 90 Days: External Evaluation of Methods to Engineer
Biology. J Am Chem Soc.

[1617] Feng J., Yang C., Zhao Z., Xu J., Li J., Li P. (2021). Application of Cell-Free Protein
Synthesis System for
the Biosynthesis of l-Theanine. ACS Synth Biol.

[1618] Miguez A. M., Zhang Y., Piorino F., Styczynski M. P. (2021). Metabolic
Dynamics in Escherichia coli-Based Cell-Free Systems. ACS Synth Biol.

[1619] Rasor B. J., Vogeli B., Jewett M. C., Karim A. S. (2022). Cell-Free
Protein Synthesis for High-Throughput Biosynthetic Pathway Prototyping. Methods Mol Biol.

[1620] Karim A. S., Dudley Q. M., Juminaga A., Yuan Y., Crowe S. A., Heggestad J. T., Garg S., Abdalla T., Grubbe W. S., Rasor B. J. (2020). In vitro prototyping
and rapid optimization of biosynthetic enzymes for cell design. Nat Chem Biol.

[1621] Kopniczky M. B., Canavan C., McClymont D. W., Crone M. A., Suckling L., Goetzmann B., Siciliano V., MacDonald J. T., Jensen K., Freemont P. S. (2020). Cell-Free
Protein Synthesis as a Prototyping Platform for Mammalian Synthetic
Biology. ACS Synth Biol.

[1622] Sierra A. M. R., Arold S. T., Grunberg R. (2022). Efficient
multi-gene
expression in cell-free droplet microreactors. PLoS One.

[1623] Ayoubi-Joshaghani M. H., Dianat-Moghadam H., Seidi K., Jahanban-Esfahalan A., Zare P., Jahanban-Esfahlan R. (2020). Cell-free
protein synthesis: The
transition from batch reactions to minimal cells and microfluidic
devices. Biotechnol Bioeng.

[1624] Gan R., Cabezas M. D., Pan M., Zhang H., Hu G., Clark L. G., Jewett M. C., Nicol R. (2022). High-Throughput Regulatory
Part Prototyping and Analysis by Cell-Free Protein Synthesis and Droplet
Microfluidics. ACS Synth Biol.

[1625] Romantseva E., Alperovich N., Ross D., Lund S. P., Strychalski E. A. (2022). Effects
of DNA template preparation on variability
in cell-free protein production. Synth Biol
(Oxf).

[1626] Dudley Q. M., Cai Y. M., Kallam K., Debreyne H., Carrasco
Lopez J. A., Patron N. J. (2021). Biofoundry-assisted expression and
characterization of plant proteins. Synth Biol
(Oxf).

[1627] Ogawa A., Takamatsu M. (2019). Mutation of the start codon to enhance
Cripavirus internal ribosome entry site-mediated translation in a
wheat germ extract. Bioorg Med Chem Lett.

[1628] Bottomley W., Whitfeld P. R. (1979). Cell-free transcription
and translation
of total spinach chloroplast DNA. Eur J Biochem.

[1629] Bard J., Bourque D. P., Hildebrand M., Zaitlin D. (1985). In vitro expression
of chloroplast genes in lysates
of higher plant chloroplasts. Proc Natl Acad
Sci U S A.

[1630] Orozco E. M., Mullet J. E., Chua N. H. (1985). An in vitro
system for accurate transcription initiation of chloroplast protein
genes. Nucleic Acids Res.

[1631] Yoshimoto K., Sakaiya M., Isono K., Kobayashi H. (2001). Comparison
of Strength of Endogenous and Exogenous Gene Promoters in Arabidopsis
Chloroplasts. Plant Biotechnology.

[1632] Inaba-Hasegawa K., Ohmura A., Nomura M., Sugiura M. (2021). Development
of an In Vitro Chloroplast Splicing System: Sequences Required for
Correct pre-mRNA Splicing. Plant Cell Physiol.

[1633] Yerramilli V. S., Kim K. H. (2018). Labeling RNAs in live cells using
malachite green aptamer scaffolds as fluorescent probes. ACS synthetic biology.

[1634] Cole S. D., Beabout K., Turner K. B., Smith Z. K., Funk V. L., Harbaugh S. V., Liem A. T., Roth P. A., Geier B. A., Emanuel P. A. (2019). Quantification
of Interlaboratory
Cell-Free Protein Synthesis Variability. ACS
Synth Biol.

[1635] Singh D., Dhar S., Yadav D. K. (2010). Effect of endophytic
bacterial antagonists against black rot disease of cauliflower caused
by Xanthomonas campestris pv. campestris. Indian
Phytopath.

[1636] Kakar K. U., Ren X. L., Nawaz Z., Cui Z. Q., Li B., Xie G. L., Hassan M. A., Ali E., Sun G. C. (2016). A consortium
of rhizobacterial strains and biochemical growth elicitors improve
cold and drought stress tolerance in rice (Oryza sativa L.). Plant Biol (Stuttg).

[1637] Ali B., Sabri A. N., Ljung K., Hasnain S. (2009). Auxin production by
plant associated bacteria: impact on endogenous IAA content and growth
of Triticum aestivum L. Lett Appl Microbiol.

[1638] Shahzad R., Waqas M., Khan A. L., Asaf S., Khan M. A., Kang S. M., Yun B. W., Lee I. J. (2016). Seed-borne
endophytic Bacillus amyloliquefaciens RWL-1 produces gibberellins
and regulates endogenous phytohormones of Oryza sativa. Plant Physiol Biochem.

[1639] Ravanbakhsh M., Sasidharan R., Voesenek L., Kowalchuk G. A., Jousset A. (2018). Microbial modulation of plant ethylene signaling: ecological
and evolutionary consequences. Microbiome.

[1640] Kessler J. D., Valentine D. L., Redmond M. C., Du M., Chan E. W., Mendes S. D., Quiroz E. W., Villanueva C. J., Shusta S. S., Werra L. M. (2011). A persistent oxygen
anomaly reveals the fate of spilled methane in the deep Gulf of Mexico. Science.

[1641] Ossowicki A., Tracanna V., Petrus M. L. C., van
Wezel G., Raaijmakers J. M., Medema M. H., Garbeva P. (2020). Microbial
and volatile profiling of soils suppressive to Fusarium culmorum of
wheat. Proc Biol Sci.

[1642] Carrión V. J., Perez-Jaramillo J., Cordovez V., Tracanna V., de Hollander M., Ruiz-Buck D., Mendes L. W., van Ijcken W. F. J., Gomez-Exposito R., Elsayed S. S. (2019). Pathogen-induced
activation of disease-suppressive functions in the endophytic root
microbiome. Science (New York, N.Y.).

[1643] Fierer N. (2017). Embracing
the unknown: disentangling the complexities
of the soil microbiome. Nat Rev Microbiol.

[1644] Li X. G., Jousset A., de Boer W., Carrion V. J., Zhang T. L., Wang X. X., Kuramae E. E. (2019). Legacy of land use
history determines reprogramming of plant physiology by soil microbiome. Isme J.

[1645] Banerjee S., Walder F., Buchi L., Meyer M., Held A. Y., Gattinger A., Keller T., Charles R., van der Heijden M. G. A. (2019). Agricultural intensification reduces
microbial network complexity and the abundance of keystone taxa in
roots. Isme J.

[1646] Bickel S., Or D. (2020). Soil bacterial diversity
mediated
by microscale aqueous-phase processes across biomes. Nat Commun.

[1647] Begum N., Qin C., Ahanger M. A., Raza S., Khan M. I., Ashraf M., Ahmed N., Zhang L. (2019). Role of Arbuscular
Mycorrhizal Fungi in Plant Growth Regulation: Implications in Abiotic
Stress Tolerance. Front Plant Sci.

[1648] Evans P. N., Boyd J. A., Leu A. O., Woodcroft B. J., Parks D. H., Hugenholtz P., Tyson G. W. (2019). An evolving view
of methane metabolism in the Archaea. Nat Rev
Microbiol.

[1649] Morgavi D. P., Forano E., Martin C., Newbold C. J. (2010). Microbial
ecosystem and methanogenesis in ruminants. Animal.

[1650] Schorn, S. ; Ahmerkamp, S. ; Bullock, E. ; Weber, M. ; Lott, C. ; Liebeke, M. ; Lavik, G. ; Kuypers, M. M. M. ; Graf, J. S. ; Milucka, J. Diverse methylotrophic methanogenic archaea cause high methane emissions from seagrass meadows. Proc Natl Acad Sci U S A 2022, 119 (9), 10.1073/pnas.2106628119. PMC8892325 35165204

[1651] Rudrappa T., Czymmek K. J., Pare P. W., Bais H. P. (2008). Root-secreted
malic acid recruits beneficial soil bacteria. Plant Physiol.

[1652] Tian B., Pei Y., Huang W., Ding J., Siemann E. (2021). Increasing flavonoid concentrations
in root exudates
enhance associations between arbuscular mycorrhizal fungi and an invasive
plant. Isme J.

[1653] Vilchez J. I., Yang Y., He D., Zi H., Peng L., Lv S., Kaushal R., Wang W., Huang W., Liu R. (2020). DNA demethylases are
required for myo-inositol-mediated mutualism between plants and beneficial
rhizobacteria. Nat Plants.

[1654] Lebeis S. L., Paredes S. H., Lundberg D. S., Breakfield N., Gehring J., McDonald M., Malfatti S., Glavina
del Rio T., Jones C. D., Tringe S. G. (2015). Salicylic
acid modulates colonization of the root microbiome by specific bacterial
taxa. Science.

[1655] Zhalnina K., Louie K. B., Hao Z., Mansoori N., da Rocha U. N., Shi S., Cho H., Karaoz U., Loque D., Bowen B. P. (2018). Dynamic
root exudate
chemistry and microbial substrate preferences drive patterns in rhizosphere
microbial community assembly. Nat Microbiol.

[1656] Yan Y., Kuramae E. E., de Hollander M., Klinkhamer P. G., van Veen J. A. (2017). Functional traits dominate the diversity-related selection
of bacterial communities in the rhizosphere. Isme J.

[1657] Lundberg D. S., Lebeis S. L., Paredes S. H., Yourstone S., Gehring J., Malfatti S., Tremblay J., Engelbrektson A., Kunin V., Rio T. G. d. (2012). Defining the core Arabidopsis
thaliana root microbiome. Nature.

[1658] Edwards J., Johnson C., Santos-Medellin C., Lurie E., Podishetty N. K., Bhatnagar S., Eisen J. A., Sundaresan V. (2015). Structure,
variation, and assembly
of the root-associated microbiomes of rice. Proc Natl Acad Sci U S A.

[1659] van Dam N. M., Bouwmeester H. J. (2016). Metabolomics
in the Rhizosphere:
Tapping into Belowground Chemical Communication. Trends Plant Sci.

[1660] Chaparro J. M., Badri D. V., Bakker M. G., Sugiyama A., Manter D. K., Vivanco J. M. (2013). Root exudation of phytochemicals
in Arabidopsis follows specific patterns that are developmentally
programmed and correlate with soil microbial functions. PloS one.

[1661] Ren, X. ; Murray, R. M. Role of interaction network topology in controlling microbial population in consortia. In 2018 IEEE Conference on Decision and Control (CDC), 2018; IEEE: pp 2691-2697.

[1662] Zegeye, E. K. ; Brislawn, C. J. ; Farris, Y. ; Fansler, S. J. ; Hofmockel, K. S. ; Jansson, J. K. ; Wright, A. T. ; Graham, E. B. ; Naylor, D. ; McClure, R. S. ; Selection, Succession, and Stabilization of Soil Microbial Consortia. mSystems 2019, 4 (4), 10.1128/mSystems.00055-19. PMC6517688 31098394

[1663] Chaudhary P., Xu M., Ahamad L., Chaudhary A., Kumar G., Adeleke B. S., Verma K. K., Hu D.-M., Širić I., Kumar P. (2023). Application of synthetic
consortia
for improvement of soil fertility, pollution remediation, and agricultural
productivity: a review. Agronomy.

[1664] Ren, X. ; Murray, R. M. Cooperation enhances robustness of coexistence in spatially structured consortia. In 2019 18th European Control Conference (ECC), 2019; IEEE: pp 2651-2656.

[1665] Ren, X. ; Baetica, A.-A. ; Swaminathan, A. ; Murray, R. M. Population regulation in microbial consortia using dual feedback control. In 2017 IEEE 56th annual conference on decision and control (CDC), 2017; IEEE: pp 5341-5347.

[1666] Zhang, T. ; Zhang, H. Microbial Consortia Are Needed to Degrade Soil Pollutants. Microorganisms 2022, 10 (2), 261 10.3390/microorganisms10020261. 35208716 PMC8874626

[1667] Ceroni F., Algar R., Stan G. B., Ellis T. (2015). Quantifying
cellular capacity identifies gene expression designs with reduced
burden. Nat Methods.

[1668] Diaz-Zorita M., Fernandez-Canigia M.
V. (2009). Field performance of
a liquid formulation of Azospirillum brasilense on dryland wheat productivity. Eur J Soil Biol.

[1669] Dobbelaere S., Croonenborghs A., Thys A., Ptacek D., Vanderleyden J., Dutto P., Labandera-Gonzalez C., Caballero-Mellado J., Aguirre J. F., Kapulnik Y. (2001). Responses
of agronomically important crops to inoculation with Azospirillum. Aust J Plant Physiol.

[1670] O'Hanlon K. (2019). Plant Growth-Promoting Bacteria Field
Trials in Europe. Endophytes for a Growing World.

[1671] Weinzierl, R. ; Henn, T. ; Koehler, P. ; Tucker, C. Microbial insecticides; Cooperative Extension Service, University of Illinois at Urbana-Champaign, 1989.

[1672] Kumar S., Hasty J. (2023). Stability, robustness,
and containment:
preparing synthetic biology for real-world deployment. Curr Opin Biotechnol.

[1673] Wright O., Stan G. B., Ellis T. (2013). Building-in biosafety
for synthetic biology. Microbiology (Reading).

[1674] Santos, M. S. ; Nogueira, M. A. ; Hungria, M. Microbial inoculants: reviewing the past, discussing the present and previewing an outstanding future for the use of beneficial bacteria in agriculture. Amb Express 2019, 9 (1), 10.1186/s13568-019-0932-0. PMC6925611 31865554

[1675] Sammauria R., Kumawat S., Kumawat P., Singh J., Jatwa T. K. (2020). Microbial
inoculants: potential tool for sustainability
of agricultural production systems. Arch Microbiol.

[1676] Alori E. T., Babalola O. O. (2018). Microbial Inoculants for Improving
Crop Quality and Human Health in Africa. Front
Microbiol.

[1677] Glover, L. A. Environmental and regulatory aspects of using genetically-modified microorganisms in the field. In Molecular Biology in Crop Protection; Marshall, G. , Walters, D. Eds.; Springer Netherlands, 1994; pp 263-274.

[1678] Wesseler J., Kleter G., Meulenbroek M., Purnhagen K. P. (2023). EU regulation of genetically modified microorganisms
in light of new policy developments: Possible implications for EU
bioeconomy investments. Applied Economic Perspectives
and Policy.

[1679] Amenumey S. E., Capel P. D. (2014). Fertilizer consumption and energy
input for 16 crops in the United States. Natural
resources research.

[1680] Woods J., Williams A., Hughes J. K., Black M., Murphy R. (2010). Energy and the food system. Philos
Trans R Soc Lond B Biol Sci.

[1681] Cordell D., White S. (2011). Peak phosphorus: clarifying
the key
issues of a vigorous debate about long-term phosphorus security. Sustainability.

[1682] Oosterhuis D. M., Loka D. A., Raper T. B. (2013). Potassium
and stress
alleviation: Physiological functions and management of cotton. Journal of plant nutrition and soil science.

[1683] Sharma, S. B. ; Sayyed, R. Z. ; Trivedi, M. H. ; Gobi, T. A. Phosphate solubilizing microbes: sustainable approach for managing phosphorus deficiency in agricultural soils. Springerplus 2013, 2, 10.1186/2193-1801-2-587. PMC4320215 25674415

[1684] Alori E. T., Glick B. R., Babalola O. O. (2017). Microbial phosphorus
solubilization and its potential for use in sustainable agriculture. Frontiers in Microbiology.

[1685] Shulse, C. N. ; Chovatia, M. ; Agosto, C. ; Wang, G. ; Hamilton, M. ; Deutsch, S. ; Yoshikuni, Y. ; Blow, M. J. Engineered Root Bacteria Release Plant-Available Phosphate from Phytate. Appl Environ Microb 2019, 85 (18), 10.1128/AEM.01210-19. PMC6715853 31285192

[1686] Radzki W., Gutierrez Manero F. J., Algar E., Lucas Garcia J. A., Garcia-Villaraco A., Ramos Solano B. (2013). Bacterial siderophores efficiently
provide iron to iron-starved tomato plants in hydroponics culture. Antonie Van Leeuwenhoek.

[1687] Rungin S., Indananda C., Suttiviriya P., Kruasuwan W., Jaemsaeng R., Thamchaipenet A. (2012). Plant growth
enhancing effects by a siderophore-producing endophytic streptomycete
isolated from a Thai jasmine rice plant (Oryza sativa L. cv. KDML105). Antonie Van Leeuwenhoek.

[1688] Lemare M., Puja H., David S. R., Mathieu S., Ihiawakrim D., Geoffroy V. A., Rigouin C. (2022). Engineering
siderophore
production in Pseudomonas to improve asbestos weathering. Microb Biotechnol.

[1689] Krithika S., Balachandar D. (2016). Expression
of Zinc Transporter Genes
in Rice as Influenced by Zinc-Solubilizing Enterobacter cloacae Strain
ZSB14. Front Plant Sci.

[1690] Sharma S. K., Sharma M. P., Ramesh A., Joshi O. P. (2012). Characterization
of zinc-solubilizing Bacillus isolates and their potential to influence
zinc assimilation in soybean seeds. J Microbiol
Biotechnol.

[1691] Teale W. D., Paponov I. A., Palme K. (2006). Auxin in action: signalling,
transport and the control of plant growth and development. Nature Reviews Molecular Cell Biology.

[1692] Calvo P., Ormeno-Orrillo E., Martinez-Romero E., Zuniga D. (2010). Characterization of Bacillus isolates
of potato rhizosphere
from andean soils of Peru and their potential PGPR characteristics. Braz J Microbiol.

[1693] Duca D. R., Rose D. R., Glick B. R. (2018). Indole
acetic acid
overproduction transformants of the rhizobacterium Pseudomonas sp
UW4. Anton Leeuw Int J G.

[1694] Zúñiga A., Fuente F. D. L., Federici F., Lionne C., Bônnet J., De Lorenzo V., González B. (2018). An Engineered
Device for Indoleacetic Acid Production under Quorum Sensing Signals
Enables Cupriavidus pinatubonensis JMP134 to Stimulate Plant Growth. ACS Synth. Biol..

[1695] Iqbal, N. ; Khan, N. A. ; Ferrante, A. ; Trivellini, A. ; Francini, A. ; Khan, M. I. R. Ethylene Role in Plant Growth, Development and Senescence: Interaction with Other Phytohormones. Frontiers in Plant Science 2017, 8, 10.3389/fpls.2017.00475. PMC5378820 28421102

[1696] Glick B. R., Todorovic B., Czarny J., Cheng Z. Y., Duan J., McConkey B. (2007). Promotion of plant growth by bacterial
ACC deaminase. Crit Rev Plant Sci.

[1697] van Loon L. C., Geraats B. P. J., Linthorst H. J. M. (2006). Ethylene
as a modulator of disease resistance in plants. Trends in Plant Science.

[1698] Guinel, F. C. Ethylene, a Hormone at the Center-Stage of Nodulation. Frontiers in Plant Science 2015, 6, 10.3389/fpls.2015.01121. PMC4714629 26834752

[1699] Glick B. R. (2014). Bacteria with ACC deaminase can promote
plant growth
and help to feed the world. Microbiol Res.

[1700] Tavares M. J., Nascimento F. X., Glick B. R., Rossi M. J. (2018). The expression
of an exogenous ACC deaminase by the endophyte Serratia grimesii BXF1
promotes the early nodulation and growth of common bean. Letters in Applied Microbiology.

[1701] Fernandez-Llamosas, H. ; Ibero, J. ; Thijs, S. ; Imperato, V. ; Vangronsveld, J. ; Diaz, E. ; Carmona, M. Enhancing the Rice Seedlings Growth Promotion Abilities of Azoarcussp. CIB by Heterologous Expression of ACC Deaminase to Improve Performance of Plants Exposed to Cadmium Stress. Microorganisms 2020, 8 (9), 1453. 10.3390/microorganisms8091453 32971998 PMC7564240

[1702] Pantelides I. S., Tjamos S. E., Pappa S., Kargakis M., Paplomatas E. J. (2013). The ethylene receptor ETR1 is required
for Fusarium
oxysporum pathogenicity. Plant Pathol.

[1703] Di X. T., Gomila J., Takken F. L. W. (2017). Involvement of
salicylic acid, ethylene and jasmonic acid signalling pathways in
the susceptibility of tomato to Fusarium oxysporum. Mol Plant Pathol.

[1704] Liu Y., Zhu A., Tan H., Cao L., Zhang R. (2019). Engineering
banana endosphere microbiome to improve Fusarium wilt resistance in
banana. Microbiome.

[1705] Gong T., Xu X., Dang Y., Kong A., Wu Y., Liang P., Wang S., Yu H., Xu P., Yang C. (2018). An engineered Pseudomonas putida
can simultaneously degrade organophosphates,
pyrethroids and carbamates. Sci Total Environ.

[1706] Martinez I., Mohamed M. E. S., Rozas D., Garcia J. L., Diaz E. (2016). Engineering synthetic bacterial consortia
for enhanced desulfurization
and revalorization of oil sulfur compounds. Metab. Eng..

[1707] Adams B. L. (2016). The Next
Generation of Synthetic Biology Chassis: Moving
Synthetic Biology from the Laboratory to the Field. ACS Synth Biol.

[1708] Liu, S. F. ; Hao, H. T. ; Lu, X. ; Zhao, X. ; Wang, Y. ; Zhang, Y. B. ; Xie, Z. K. ; Wang, R. Y. Transcriptome profiling of genes involved in induced systemic salt tolerance conferred by Bacillus amyloliquefaciens FZB42 in Arabidopsis thaliana. Scientific Reports 2017, 7, 10.1038/s41598-017-11308-8. PMC5597682 28904348

[1709] Bharti, N. ; Pandey, S. S. ; Barnawal, D. ; Patel, V. K. ; Kalra, A. Plant growth promoting rhizobacteria Dietzia natronolimnaea modulates the expression of stress responsive genes providing protection of wheat from salinity stress. Scientific Reports 2016, 6, 10.1038/srep34768. PMC5052518 27708387

[1710] Abd El-Daim, I. A. ; Bejai, S. ; Meijer, J. Bacillus velezensis 5113 Induced Metabolic and Molecular Reprogramming during Abiotic Stress Tolerance in Wheat. Scientific Reports 2019, 9, 10.1038/s41598-019-52567-x. PMC6841942 31704956

[1711] Zhang H., Kim M. S., Sun Y., Dowd S. E., Shi H. Z., Pare P. W. (2008). Soil bacteria confer
plant salt tolerance
by tissue-specific regulation of the sodium transporter HKT1. Mol Plant Microbe In.

[1712] Sandhya V., Z A.
S. K., Grover M., Reddy G., Venkateswarlu B. (2009). Alleviation of drought stress effects
in sunflower
seedlings by the exopolysaccharides producing Pseudomonas putida strain
GAP-P45. Biol Fert Soils.

[1713] Ashraf M., Hasnain S., Berge O., Mahmood T. (2004). Inoculating
wheat seedlings with exopolysaccharide-producing bacteria restricts
sodium uptake and stimulates plant growth under salt stress. Biol Fert Soils.

[1714] Lindow, S. ; Panopoulos, N. Field tests of recombinant ice-Pseudomonas syringae for biological frost control in potato. Proceedings of the First International Conference on Release of Genetically Engineered Microorganisms; Sussman, M., Collins, C. H., Skinner, F. A., Eds.; Academic Press, 1988; pp 121-138.

[1715] Environmental Release - Ice Nucleation - Advanced Genetics Sciences. Biotechnology Law Report 1988, 7 (2), 111-112. 10.1089/blr.1988.7.111.

[1716] Hazel, W. J. Pseudomonas syringae. Final progress report on 1987 field test and amended Experimental Use Permit application from Steven Lindow of the University of California. US EPA Office of Pesticides and Toxic Substances, 1988.

[1717] Crawford M. (1987). California Field-Test
Goes Forward. Science.

[1718] Vallad G. E., Goodman R. M. (2004). Systemic acquired
resistance and
induced systemic resistance in conventional agriculture. Crop Sci.

[1719] Maurhofer M., Reimmann C., Schmidli-Sacherer P., Heeb S., Haas D., Defago G. (1998). Salicylic acid biosynthetic
genes expressed in Pseudomonas fluorescens strain P3 improve the induction
of systemic resistance in tobacco against tobacco necrosis virus. Phytopathology.

[1720] Sanahuja G., Banakar R., Twyman R. M., Capell T., Christou P. (2011). Bacillus thuringiensis: a century
of research, development
and commercial applications. Plant Biotechnol
J.

[1721] Nielsen-LeRoux C., Gaudriault S., Ramarao N., Lereclus D., Givaudan A. (2012). How the insect pathogen
bacteria Bacillus thuringiensis
and Xenorhabdus/Photorhabdus occupy their hosts. Current Opinion in Microbiology.

[1722] Baum J. A., Kakefuda M., GawronBurke C. (1996). Engineering
Bacillus thuringiensis bioinsecticides with an indigenous site-specific
recombination system. Appl Environ Microb.

[1723] Anastassiadou, M. ; Arena, M. ; Auteri, D. ; Brancato, A. ; Bura, L. ; Cabrera, L. C. ; Chaideftou, E. ; Chiusolo, A. ; Crivellente, F. ; De Lentdecker, C. ; Peer review of the pesticide risk assessment of the active substance Bacillus thuringiensis ssp. aizawai strain ABTS-1857. Efsa J 2020, 18 (10), 10.2903/j.efsa.2020.6294. PMC7596667 33163112

[1724] Anastassiadou, M. ; Arena, M. ; Auteri, D. ; Brancato, A. ; Bura, L. ; Cabrera, L. C. ; Chaideftou, E. ; Chiusolo, A. ; Crivellente, F. ; De Lentdecker, C. ; Peer review of the pesticide risk assessment of the active substance Bacillus thuringiensis subsp. kurstaki strain EG2348. Efsa J 2021, 19 (4), 10.2903/j.efsa.2021.6495 PMC8033632 33859734

[1725] Peng Q., Yu Q., Song F. (2019). Expression
of cry genes
in Bacillus thuringiensis biotechnology. Appl
Microbiol Biotechnol.

[1726] Audtho M., Valaitis A. P., Alzate O., Dean D. H. (1999). Production
of chymotrypsin-resistant Bacillus thuringiensis Cry2Aa1 delta-endotoxin
by protein engineering. Appl Environ Microbiol.

[1727] Rajamohan F., Alzate O., Cotrill J. A., Curtiss A., Dean D. H. (1996). Protein
engineering of Bacillus thuringiensis δ-endotoxin:
Mutations at domain II of CryIAb enhance receptor affinity and toxicity
toward gypsy moth larvae. Proceedings of the
National Academy of Sciences.

[1728] Muralimohan, N. ; Rathinam, M. ; Kasturi, K. ; Sreevathsa, R. Protein Engineering Of Bt Genes cry1Ab And cry1Ba For The Development Of Chimeric Genes cryAbabba, cryBabaab And cryAbbaab Via Domain Swapping. Journal of Advanced Zoology 2024, 45 (3), 10.53555/jaz.v45i3.4137.

[1729] Abdullah M. A. F., Alzate O., Mohammad M., McNall R. J., Adang M. J., Dean D. H. (2003). Introduction of Culex toxicity into
Bacillus thuringiensis Cry4Ba by protein engineering. Applied and environmental microbiology.

[1730] Qiao J.-Q., Wu H.-J., Huo R., Gao X.-W., Borriss R. (2014). Stimulation of plant growth and biocontrol
by Bacillus
amyloliquefaciens subsp. plantarum FZB42 engineered for improved action. Chem. Biol. Technol. Agric..

[1731] Wang S. A., Wu H. J., Zhan J. A., Xia Y. F., Gao S. F., Wang W. D., Xue P. Q., Gao X. W. (2011). The role
of synergistic action and molecular mechanism in the effect of genetically
engineered strain Bacillus subtilis OKBHF in enhancing tomato growth
and Cucumber mosaic virus resistance. Biocontrol.

[1732] Shivaraj S. M., Vats S., Bhat J. A., Dhakte P., Goyal V., Khatri P., Kumawat S., Singh A., Prasad M., Sonah H. (2020). Nitric oxide and hydrogen
sulfide crosstalk during heavy metal stress in plants. Physiol Plantarum.

[1733] Li R., Wu H., Ding J., Fu W., Gan L., Li Y. (2017). Mercury pollution in vegetables,
grains and soils from areas surrounding
coal-fired power plants. Sci Rep.

[1734] Pazirandeh M., Wells B. M., Ryan R. L. (1998). Development
of bacterium-based
heavy metal biosorbents: Enhanced uptake of cadmium and mercury by
Escherichia coli expressing a metal binding motif. Appl Environ Microb.

[1735] Nguyen T. T., Lee H. R., Hong S. H., Jang J. R., Choe W. S., Yoo I. K. (2013). Selective lead adsorption
by recombinant
Escherichia coli displaying a lead-binding peptide. Appl Biochem Biotechnol.

[1736] Ravikumar S., Yoo I.-k., Lee S. Y., Hong S. H. (2011). Construction
of copper removing bacteria through the integration of two-component
system and cell surface display. Applied biochemistry
and biotechnology.

[1737] Wei W., Liu X. Z., Sun P. Q., Wang X., Zhu H., Hong M., Mao Z. W., Zhao J. (2014). Simple Whole-Cell Biodetection
and Bioremediation of Heavy Metals Based on an Engineered Lead-Specific
Operon. Environ. Sci. Technol..

[1738] Yin K., Lv M., Wang Q. N., Wu Y. X., Liao C. Y., Zhang W. W., Chen L. X. (2016). Simultaneous bioremediation and biodetection
of mercury ion through surface display of carboxylesterase E2 from
Pseudomonas aeruginosa PA1. Water Res.

[1739] Sauge-Merle S., Cuine S., Carrier P., Lecomte-Pradines C., Luu D. T., Peltier G. (2003). Enhanced toxic metal
accumulation
in engineered bacterial cells expressing Arabidopsis thaliana phytochelatin
synthase. Appl Environ Microb.

[1740] Kang S. H., Singh S., Kim J. Y., Lee W., Mulchandani A., Chen W. (2007). Bacteria metabolically engineered
for enhanced phytochelatin production and cadmium accumulation. Appl Environ Microb.

[1741] Duprey, A. ; Chansavang, V. ; Fremion, F. ; Gonthier, C. ; Louis, Y. ; Lejeune, P. ; Springer, F. ; Desjardin, V. ; Rodrigue, A. ; Dorel, C. “NiCo Buster”: engineering E-coli for fast and efficient capture of cobalt and nickel. Journal of Biological Engineering 2014, 8, 10.1186/1754-1611-8-19. PMC4124493 25104972

[1742] Chen J., Sun G. X., Wang X. X., de Lorenzo V., Rosen B. P., Zhu Y. G. (2014). Volatilization of Arsenic from Polluted
Soil by Pseudomonas putida Engineered for Expression of the arsM Arsenic(III)
S-Adenosine Methyltransferase Gene. Environ.
Sci. Technol..

[1743] Dvorak P., Nikel P. I., Damborsky J., de Lorenzo V. (2017). Bioremediation
3.0: Engineering pollutant-removing
bacteria in the times of systemic biology. Biotechnol
Adv.

[1744] Villacieros M., Whelan C., Mackova M., Molgaard J., Sanchez-Contreras M., Lloret J., Aguirre
de Carcer D., Oruezabal R. I., Bolanos L., Macek T. (2005). Polychlorinated
biphenyl rhizoremediation by Pseudomonas fluorescens F113 derivatives,
using a Sinorhizobium meliloti nod system to drive bph gene expression. Appl Environ Microbiol.

[1745] Zuo Z., Gong T., Che Y., Liu R., Xu P., Jiang H., Qiao C., Song C., Yang C. (2015). Engineering
Pseudomonas putida KT2440 for simultaneous degradation of organophosphates
and pyrethroids and its application in bioremediation of soil. Biodegradation.

[1746] Kurumbang N. P., Dvorak P., Bendl J., Brezovsky J., Prokop Z., Damborsky J. (2014). Computer-assisted
engineering of
the synthetic pathway for biodegradation of a toxic persistent pollutant. ACS Synth Biol.

[1747] Wittich R. M., Wolff P. (2007). Growth of the genetically
engineered
strain Cupriavidus necator RW112 with chlorobenzoates and technical
chlorobiphenyls. Microbiology (Reading).

[1748] Wittebole X., De Roock S., Opal S. M. (2014). A historical overview
of bacteriophage therapy as an alternative to antibiotics for the
treatment of bacterial pathogens. Virulence.

[1749] Mallmann W. L., Hemstreet C. (1924). Isolation of an inhibitory substance
from plants. J Agric Res.

[1750] Buttimer C., McAuliffe O., Ross R. P., Hill C., O'Mahony J., Coffey A. (2017). Bacteriophages and Bacterial Plant
Diseases. Front Microbiol.

[1751] Holtappels D., Fortuna K., Lavigne R., Wagemans J. (2021). The future
of phage biocontrol in integrated plant protection for sustainable
crop production. Curr Opin Biotechnol.

[1752] Huang, Y. ; Wang, W. ; Zhang, Z. ; Gu, Y. ; Huang, A. ; Wang, J. ; Hao, H. Phage Products for Fighting Antimicrobial Resistance. Microorganisms 2022, 10 (7), 1324 10.3390/microorganisms10071324. 35889048 PMC9324367

[1753] Adriaenssens E. M., Van Vaerenbergh J., Vandenheuvel D., Dunon V., Ceyssens P. J., De Proft M., Kropinski A. M., Noben J. P., Maes M., Lavigne R. (2012). T4-related
bacteriophage
LIMEstone isolates for the control of soft rot on potato caused by
'Dickeya solani'. PLoS One.

[1754] Buttimer, C. ; McAuliffe, O. ; Ross, R. P. ; Hill, C. ; O’Mahony, J. ; Coffey, A. Bacteriophages and Bacterial Plant Diseases. Front. Microbiol. 2017, 8, 10.3389/fmicb.2017.00034. PMC5247434 28163700

[1755] Iriarte F. B., Obradovic A., Wernsing M. H., Jackson L. E., Balogh B., Hong J. A., Momol M. T., Jones J. B., Vallad G. E. (2012). Soil-based
systemic delivery and phyllosphere in vivo
propagation of bacteriophages: Two possible strategies for improving
bacteriophage persistence for plant disease control. Bacteriophage.

[1756] Rahimi-Midani, A. ; Choi, T. J. Transport of Phage in Melon Plants and Inhibition of Progression of Bacterial Fruit Blotch. Viruses 2020, 12 (4), 477 10.3390/v12040477. 32340158 PMC7232510

[1757] Parcey, M. ; Gayder, S. ; Castle, A. J. ; Svircev, A. M. Molecular Profile of Phage Infection: A Novel Approach for the Characterization of Erwinia Phages through qPCR. Int J Mol Sci 2020, 21 (2), 553 10.3390/ijms21020553. 31952282 PMC7014438

[1758] Lemire S., Yehl K. M., Lu T. K. (2018). Phage-Based
Applications
in Synthetic Biology. Annu Rev Virol.

[1759] Schwarz C., Mathieu J., Laverde Gomez J. A., Yu P., Alvarez P. J. J. (2022). Renaissance
for Phage-Based Bacterial
Control. Environ Sci Technol.

[1760] Edgar R., Friedman N., Molshanski-Mor S., Qimron U. (2012). Reversing bacterial resistance to antibiotics by phage-mediated
delivery of dominant sensitive genes. Appl Environ
Microbiol.

[1761] Park J. Y., Moon B. Y., Park J. W., Thornton J. A., Park Y. H., Seo K. S. (2017). Genetic engineering of a temperate
phage-based delivery system for CRISPR/Cas9 antimicrobials against
Staphylococcus aureus. Sci Rep.

[1762] Born, Y. ; Fieseler, L. ; Thony, V. ; Leimer, N. ; Duffy, B. ; Loessner, M. J. Engineering of Bacteriophages Y2::dpoL1-C and Y2::luxAB for Efficient Control and Rapid Detection of the Fire Blight Pathogen, Erwinia amylovora. Appl Environ Microbiol 2017, 83 (12), 10.1128/AEM.00341-17. PMC5452800 28389547

[1763] Goldfarb T., Sberro H., Weinstock E., Cohen O., Doron S., Charpak-Amikam Y., Afik S., Ofir G., Sorek R. (2015). BREX is a novel phage
resistance system widespread in microbial genomes. Embo J.

[1764] Ofir G., Melamed S., Sberro H., Mukamel Z., Silverman S., Yaakov G., Doron S., Sorek R. (2018). DISARM is
a widespread bacterial defence system with broad anti-phage activities. Nat Microbiol.

[1765] Barrangou R., Fremaux C., Deveau H., Richards M., Boyaval P., Moineau S., Romero D. A., Horvath P. (2007). CRISPR provides
acquired resistance against viruses in prokaryotes. Science.

[1766] Mojica F. J., Diez-Villasenor C., Garcia-Martinez J., Soria E. (2005). Intervening sequences of regularly
spaced prokaryotic repeats derive
from foreign genetic elements. J Mol Evol.

[1767] Doron, S. ; Melamed, S. ; Ofir, G. ; Leavitt, A. ; Lopatina, A. ; Keren, M. ; Amitai, G. ; Sorek, R. Systematic discovery of antiphage defense systems in the microbial pangenome. Science 2018, 359 (6379), 10.1126/science.aar4120. PMC6387622 29371424

[1768] Nayfach S., Paez-Espino D., Call L., Low S. J., Sberro H., Ivanova N. N., Proal A. D., Fischbach M. A., Bhatt A. S., Hugenholtz P. (2021). Metagenomic compendium
of 189,680 DNA viruses from the human gut microbiome. Nat Microbiol.

[1769] Pires D. P., Cleto S., Sillankorva S., Azeredo J., Lu T. K. (2016). Genetically Engineered Phages: a
Review of Advances over the Last Decade. Microbiol
Mol Biol Rev.

[1770] Yuan X., Fan L., Jin H., Wu Q., Ding Y. (2025). Phage engineering using synthetic biology and artificial intelligence
to enhance phage applications in food industry. Current Opinion in Food Science.

[1771] Doud M. B., Robertson J. M., Strathdee S. A. (2025). Optimizing
phage therapy with artificial intelligence: a perspective. Front Cell Infect Microbiol.

[1772] Shao B., Yan J. (2024). A long-context language model for
deciphering and generating bacteriophage genomes. Nat Commun.

[1773] Koskella B., Meaden S. (2013). Understanding bacteriophage specificity
in natural microbial communities. Viruses.

[1774] Mills S., Ross R. P., Hill C. (2017). Bacteriocins
and bacteriophage;
a narrow-minded approach to food and gut microbiology. Fems Microbiol Rev.

[1775] Ando H., Lemire S., Pires D. P., Lu T. K. (2015). Engineering
Modular Viral Scaffolds for Targeted Bacterial Population Editing. Cell Syst.

[1776] Yehl K., Lemire S., Yang A. C., Ando H., Mimee M., Torres M. T., de la Fuente-Nunez C., Lu T. K. (2019). Engineering Phage Host-Range and Suppressing Bacterial Resistance
through Phage Tail Fiber Mutagenesis. Cell.

[1777] Kilcher S., Studer P., Muessner C., Klumpp J., Loessner M. J. (2018). Cross-genus
rebooting of custom-made, synthetic bacteriophage
genomes in L-form bacteria. Proc Natl Acad Sci
U S A.

[1778] Jones J. B., Jackson L. E., Balogh B., Obradovic A., Iriarte F. B., Momol M. T. (2007). Bacteriophages for plant disease
control. Annu Rev Phytopathol.

[1779] Iriarte F. B., Balogh B., Momol M. T., Smith L. M., Wilson M., Jones J. B. (2007). Factors affecting
survival of bacteriophage
on tomato leaf surfaces. Appl Environ Microbiol.

[1780] Oechslin, F. Resistance Development to Bacteriophages Occurring during Bacteriophage Therapy. Viruses 2018, 10 (7), 351 10.3390/v10070351. 29966329 PMC6070868

[1781] Qiao J., Qiao X., Sun Y., Mindich L. (2010). Role of host
protein glutaredoxin 3 in the control of transcription during bacteriophage
Phi2954 infection. Proc Natl Acad Sci U S A.

[1782] Olanrewaju O. S., Ayangbenro A. S., Glick B. R., Babalola O. O. (2019). Plant health:
feedback effect of root exudates-rhizobiome interactions. Appl Microbiol Biot.

[1783] Sasse J., Martinoia E., Northen T. (2018). Feed Your Friends:
Do Plant Exudates Shape the Root Microbiome?. Trends in Plant Science.

[1784] Chaparro J. M., Sheflin A. M., Manter D. K., Vivanco J. M. (2012). Manipulating
the soil microbiome to increase soil health and plant fertility. Biol Fert Soils.

[1785] Zhang J., Liu Y. X., Zhang N., Hu B., Jin T., Xu H., Qin Y., Yan P., Zhang X., Guo X. (2019). NRT1.1B is associated
with root microbiota composition
and nitrogen use in field-grown rice. Nat Biotechnol.

[1786] Castrillo G., Teixeira P. J. P. L., Paredes S. H., Law T. F., de Lorenzo L., Feltcher M. E., Finkel O. M., Breakfield N. W., Mieczkowski P., Jones C. D., Paz-Ares J., Dangl J. L. (2017). Root microbiota drive direct integration of phosphate stress and
immunity. Nature Publishing Group.

[1787] Escobar M. A., Dandekar A. M. (2003). Agrobacterium tumefaciens as an agent
of disease. Trends in Plant Science.

[1788] Sheng J. S., Citovsky V. (1996). Agrobacterium plant
cell DNA transport:
Have virulence proteins, will travel. Plant
Cell.

[1789] Mansouri H., Petit A., Oger P., Dessaux Y. (2002). Engineered
rhizosphere: The trophic bias generated by opine-producing plants
is independent of the opine type, the soil origin, and the plant species. Appl Environ Microb.

[1790] Mondy S., Beury-cirou A. L. A., Libanga C. (2014). An increasing opine
carbon bias in artificial exudation systems and genetically modified
plant rhizospheres leads to an increasing reshaping of bacterial populations. Molecular Ecology.

[1791] Oger P. M., Mansouri H., Nesme X., Dessaux Y. (2004). Engineering
root exudation of lotus toward the production of two novel carbon
compounds leads to the selection of distinct microbial populations
in the rhizosphere. Microb Ecol.

[1792] Guyon P., Petit A., Tempe J., Dessaux Y. (1993). Transformed
Plants Producing Opines Specifically Promote Growth of Opine-Degrading
Agrobacteria. Molecular Plant-Microbe Interactions.

[1793] Savka M. A., Farrand S. K. (1992). Mannityl Opine Accumulation and Exudation
by Transgenic Tobacco. Plant Physiology.

[1794] Wilson M., Savka M. A., Hwang I., Farrand S. K., Lindow S. E. (1995). Altered
Epiphytic Colonization of Mannityl Opine-Producing
Transgenic Tobacco Plants by a Mannityl Opine-Catabolizing Strain
of Pseudomonas-Syringae. Appl Environ Microb.

[1795] Oger P., Petit A., Dessaux Y. (1997). Genetically engineered
plants producing opines alter their biological environment. Nature Biotechnology.

[1796] Ryu M.-H., Zhang J., Toth T., Khokhani D., Geddes B. A., Mus F., Garcia-Costas A., Peters J. W., Poole P. S., Ané J.-M. (2020). Control of nitrogen fixation in bacteria that associate with cereals. Nature Microbiology.

[1797] Yan D. W., Tajima H., Cline L. C., Fong R. Y., Ottaviani J. I., Shapiro H. Y., Blumwald E. (2022). Genetic modification
of flavone biosynthesis in rice enhances biofilm formation of soil
diazotrophic bacteria and biological nitrogen fixation. Plant Biotechnol J.

[1798] Waters C. M., Bassler B. L. (2005). Quorum sensing: cell-to-cell communication
in bacteria. Annu Rev Cell Dev Biol.

[1799] Boo, A. ; Amaro, R. L. ; Stan, G. B. Quorum sensing in synthetic biology: A review. Curr Opin Syst Biol 2021, 28, 100378. 10.1016/j.coisb.2021.100378

[1800] Liang, X. L. ; Wagner, R. E. ; Li, B. X. ; Zhang, N. ; Radosevich, M. Quorum Sensing Signals Alterin vitroSoil Virus Abundance and Bacterial Community Composition. Frontiers in Microbiology 2020, 11, 10.3389/fmicb.2020.01287. PMC7298970 32587586

[1801] Schikora A., Schenk S. T., Hartmann A. (2016). Beneficial
effects
of bacteria-plant communication based on quorum sensing molecules
of the N-acyl homoserine lactone group. Plant
Molecular Biology.

[1802] Venturi V., Keel C. (2016). Signaling in the Rhizosphere. Trends Plant Sci.

[1803] Von Bodman S. B., Bauer W. D., Coplin D. L. (2003). Quorum
sensing in
plant-pathogenic bacteria. Annu Rev Phytopathol.

[1804] Rinaudi-Marron L. V., Gonzalez J. E. (2015). Regulation of Symbiotically
Important
Functions by Quorum Sensing in the Sinorhizobium meliloti-Alfalfa
Interaction. Biological Nitrogen Fixation, Vols
1 and 2.

[1805] Shahni Y. S., Singh K. P., Pandey S., Panigrahi C. K., Das N., Panotra N., Sharavanan P., Mishra S. (2025). Role of Quorum Sensing
Molecules in Plant-microbe Interaction for Sustainable Agriculture:
A Review. Journal of Advances in Biology &
Biotechnology.

[1806] Fray R. G., Throup J. P., Daykin M., Wallace A., Williams P., Stewart G. S. A. B., Grierson D. (1999). Plants genetically
modified to produce N-acylhomoserine lactones communicate with bacteria. Nature Biotechnology.

[1807] Yan D., Tajima H., Cline L. C., Fong R. Y., Ottaviani J. I., Shapiro H. Y., Blumwald E. (2022). Genetic modification
of flavone biosynthesis
in rice enhances biofilm formation of soil diazotrophic bacteria and
biological nitrogen fixation. Plant Biotechnol
J.

[1808] Fray R. G., Throup J. P., Daykin M., Wallace A., Williams P., Stewart G. S., Grierson D. (1999). Plants genetically
modified to produce N-acylhomoserine lactones communicate with bacteria. Nat Biotechnol.

[1809] Wrubel R. P., Krimsky S., Anderson M. D. (1997). Regulatory
Oversight
of Genetically Engineered Microorganisms: Has Regulation Inhibited
Innovation?. Environ Manage.

[1810] Hanlon P., Sewalt V. (2021). GEMs: genetically engineered
microorganisms
and the regulatory oversight of their uses in modern food production. Crit Rev Food Sci Nutr.

[1811] Hirsch P. R. (2005). Release of transgenic bacterial inoculants
- rhizobia
as a case study. Plant Soil.

[1812] Schiemann, J. The Biosafety Results of Field Tests of Genetically Modified Plants and Microorganisms. 1998.

[1813] Wozniak, C. A. ; McClung, G. ; Gagliardi, J. ; Segal, M. ; Matthews, K. Regulation of Genetically Engineered Microorganisms Under FIFRA, FFDCA and TSCA. Regulation of Agricultural Biotechnology: The United States and Canada 2012, 57. 10.1007/978-94-007-2156-2_4.

[1814] Geddes B. A., Ryu M.-H., Mus F., Garcia Costas A., Peters J. W., Voigt C. A., Poole P. (2015). Use of plant colonizing
bacteria as chassis for transfer of N2-fixation to cereals. Current Opinion in Biotechnology.

[1815] Mus F., Crook M. B., Garcia K., Garcia Costas A., Geddes B. A., Kouri E. D., Paramasivan P., Ryu M.-H., Oldroyd G. E. D., Poole P. S. (2016). Symbiotic
Nitrogen Fixation and the Challenges to Its Extension to Nonlegumes. Appl Environ Microb.

[1816] Geddes B. A., Paramasivan P., Joffrin A., Thompson A. L., Christensen K., Jorrin B., Brett P., Conway S. J., Oldroyd G. E. D., Poole P. S. (2019). Engineering transkingdom signalling
in plants to control gene expression in rhizosphere bacteria. Nature Communications.

[1817] Haskett, T. L. ; Paramasivan, P. ; Mendes, M. D. ; Green, P. ; Geddes, B. A. ; Knights, H. E. ; Jorrin, B. ; Ryu, M. H. ; Brett, P. ; Voigt, C. A. ; Engineered plant control of associative nitrogen fixation. P Natl Acad Sci USA 2022, 119 (16), 10.1073/pnas.2117465119. PMC9169844 35412890

[1818] Helinski D. R. (2022). A Brief
History of Plasmids. EcoSal Plus.

[1819] Bower D. M., Prather K. L. (2009). Engineering of bacterial
strains
and vectors for the production of plasmid DNA. Appl Microbiol Biotechnol.

[1820] Lutz R., Bujard H. (1997). Independent and tight
regulation
of transcriptional units in Escherichia coli via the LacR/O, the TetR/O
and AraC/I1-I2 regulatory elements. Nucleic
Acids Res.

[1821] Lovett M. A., Katz L., Helinski D. R. (1974). Unidirectional replication
of plasmid ColE1 DNA. Nature.

[1822] Jain A., Srivastava P. (2013). Broad host
range plasmids. FEMS Microbiology Letters.

[1823] Martínez-García, E. ; Benedetti, I. ; Hueso, A. ; De Lorenzo, V. Mining Environmental Plasmids for Synthetic Biology Parts and Devices. Microbiology Spectrum 2015, 3 (1), 10.1128/microbiolspec.PLAS-0033-2014. 26104565

[1824] Weinstein M., Roberts R. C., Helinski D. R. (1992). A region
of the
broad-host-range plasmid RK2 causes stable in planta inheritance of
plasmids in Rhizobium meliloti cells isolated from alfalfa root nodules. J Bacteriol.

[1825] Roberts R. C., Burioni R., Helinski D. R. (1990). Genetic
characterization
of the stabilizing functions of a region of broad-host-range plasmid
RK2. J Bacteriol.

[1826] Roberts R. C., Helinski D. R. (1992). Definition of a
minimal plasmid stabilization
system from the broad-host-range plasmid RK2. J Bacteriol.

[1827] Gruber S., Hagen J., Schwab H., Koefinger P. (2014). Versatile
and stable vectors for efficient gene expression in Ralstonia eutropha
H16. J Biotechnol.

[1828] Geddes B. A., Mendoza-Suarez M. A., Poole P. S. (2019). A Bacterial Expression
Vector Archive (BEVA) for Flexible Modular Assembly of Golden Gate-Compatible
Vectors. Front Microbiol.

[1829] Antoine R., Locht C. (1992). Isolation and molecular characterization
of a novel broad-host-range plasmid from Bordetella bronchiseptica
with sequence similarities to plasmids from gram-positive organisms. Mol Microbiol.

[1830] Katashkina J. I., Kuvaeva T. M., Andreeva I. G., Skorokhodova A. Y., Biryukova I. V., Tokmakova I. L., Golubeva L. I., Mashko S. V. (2007). Construction
of stably maintained non-mobilizable derivatives of RSF1010 lacking
all known elements essential for mobilization. BMC Biotechnol.

[1831] Sato M., Sakai F. (1992). Cell survival and plasmid
stability
in *Pseudomonas syringae* containing RSF1010 and its
derivative plasmids in soil and media. Japanese
Journal of Phytopathology.

[1832] Van Elsas J. D., Trevors J. T., Van Overbeek L. S., Starodub M. E. (1989). Survival of Pseudomonas-Fluorescens Containing Plasmids
Rp4 or Prk2501 and Plasmid Stability after Introduction into 2 Soils
of Different Texture. Can J Microbiol.

[1833] Brooks A.
C., Hwang L. C. (2017). Reconstitutions
of plasmid partition
systems and their mechanisms. Plasmid.

[1834] Schumacher M. A. (2012). Bacterial plasmid partition machinery:
a minimalist
approach to survival. Curr Opin Struct Biol.

[1835] Kim J. Y., Kang H. A., Ryu D. D. (1993). Effects of the par
locus on the growth rate and structural stability of recombinant cells. Biotechnol Prog.

[1836] Manen D., Goebel T., Caro L. (1990). The par region
of pSC101
affects plasmid copy number as well as stability. Mol Microbiol.

[1837] Skogman G., Nilsson J., Gustafsson P. (1983). The use of
a partition locus to increase stability of tryptophan-operon-bearing
plasmids in Escherichia coli. Gene.

[1838] Meacock P. A., Cohen S. N. (1980). Partitioning of
bacterial plasmids
during cell division: a cis-acting locus that accomplishes stable
plasmid inheritance. Cell.

[1839] Kroll J., Klinter S., Schneider C., Voss I., Steinbuchel A. (2010). Plasmid addiction systems: perspectives
and applications in biotechnology. Microb Biotechnol.

[1840] Yang S., Kang Z., Cao W., Du G., Chen J. (2016). Construction
of a novel, stable, food-grade expression system by
engineering the endogenous toxin-antitoxin system in Bacillus subtilis. J Biotechnol.

[1841] Easter C. L., Schwab H., Helinski D. R. (1998). Role of the parCBA
operon of the broad-host-range plasmid RK2 in stable plasmid maintenance. J Bacteriol.

[1842] Wright O., Delmans M., Stan G. B., Ellis T. (2015). GeneGuard:
A modular plasmid system designed for biosafety. ACS Synth Biol.

[1843] Dong W. R., Xiang L. X., Shao J. Z. (2010). Novel antibiotic-free
plasmid selection system based on complementation of host auxotrophy
in the NAD de novo synthesis pathway. Appl Environ
Microbiol.

[1844] Vidal L., Pinsach J., Striedner G., Caminal G., Ferrer P. (2008). Development of an antibiotic-free
plasmid selection system based on glycine auxotrophy for recombinant
protein overproduction in Escherichia coli. J Biotechnol.

[1845] Velur Selvamani R. S., Telaar M., Friehs K., Flaschel E. (2014). Antibiotic-free
segregational plasmid stabilization in Escherichia coli owing to the
knockout of triosephosphate isomerase (tpiA). Microb Cell Fact.

[1846] Hagg P., de Pohl J. W., Abdulkarim F., Isaksson L. A. (2004). A host/plasmid system that is not dependent on antibiotics
and antibiotic resistance genes for stable plasmid maintenance in
Escherichia coli. J Biotechnol.

[1847] Kang C. W., Lim H. G., Yang J., Noh M. H., Seo S. W., Jung G. Y. (2018). Synthetic auxotrophs
for stable and
tunable maintenance of plasmid copy number. Metab Eng.

[1848] Sandberg T. E., Salazar M. J., Weng L. L., Palsson B. O., Feist A. M. (2019). The emergence
of adaptive laboratory evolution as an
efficient tool for biological discovery and industrial biotechnology. Metab Eng.

[1849] Lim H. G., Eng T., Banerjee D., Alarcon G., Lau A. K., Park M. R., Simmons B. A., Palsson B. O., Singer S. W., Mukhopadhyay A. (2021). Generation of Pseudomonas
putida KT2440 Strains with Efficient Utilization of Xylose and Galactose
via Adaptive Laboratory Evolution. Acs Sustain
Chem Eng.

[1850] Lin Y., Alstrup M., Pang J. K. Y., Maroti G., Er-Rafik M., Tourasse N., Okstad O. A., Kovacs A. T. (2021). Adaptation
of Bacillus
thuringiensis to Plant Colonization Affects Differentiation and Toxicity. mSystems.

[1851] Blake C., Nordgaard M., Maroti G., Kovacs A. T. (2021). Diversification
of Bacillus subtilis during experimental evolution on Arabidopsis
thaliana and the complementarity in root colonization of evolved subpopulations. Environ Microbiol.

[1852] Li E., Zhang H., Jiang H., Pieterse C. M. J., Jousset A., Bakker P., de Jonge R. (2021). Experimental-Evolution-Driven
Identification
of Arabidopsis Rhizosphere Competence Genes in Pseudomonas protegens. mBio.

[1853] Raman S., Rogers J. K., Taylor N. D., Church G. M. (2014). Evolution-guided
optimization of biosynthetic pathways. Proc
Natl Acad Sci U S A.

[1854] Luan G., Cai Z., Li Y., Ma Y. (2013). Genome replication
engineering assisted continuous evolution (GREACE) to improve microbial
tolerance for biofuels production. Biotechnol
Biofuels.

[1855] Badran A. H., Liu D. R. (2015). Development of potent in vivo mutagenesis
plasmids with broad mutational spectra. Nat
Commun.

[1856] Camps M., Naukkarinen J., Johnson B. P., Loeb L. A. (2003). Targeted
gene evolution in Escherichia coli using a highly error-prone DNA
polymerase I. Proc Natl Acad Sci U S A.

[1857] Ravikumar A., Arzumanyan G. A., Obadi M. K. A., Javanpour A. A., Liu C. C. (2018). Scalable, Continuous Evolution of Genes at Mutation
Rates above Genomic Error Thresholds. Cell.

[1858] Wang H. H., Isaacs F. J., Carr P. A., Sun Z. Z., Xu G., Forest C. R., Church G. M. (2009). Programming cells by multiplex genome
engineering and accelerated evolution. Nature.

[1859] Halperin S. O., Tou C. J., Wong E. B., Modavi C., Schaffer D. V., Dueber J. E. (2018). CRISPR-guided DNA polymerases enable
diversification of all nucleotides in a tunable window. Nature.

[1860] Tou C. J., Schaffer D. V., Dueber J. E. (2020). Targeted
Diversification
in the S. cerevisiae Genome with CRISPR-Guided DNA Polymerase I. ACS Synth Biol.

[1861] Finney-Manchester S.
P., Maheshri N. (2013). Harnessing
mutagenic homologous recombination
for targeted mutagenesis in vivo by TaGTEAM. Nucleic Acids Res.

[1862] Mengiste A. A., Wilson R. H., Weissman R. F., Papa L. J., Hendel S. J., Moore C. L., Butty V. L., Shoulders M. D. (2023). Expanded
MutaT7 toolkit efficiently and simultaneously
accesses all possible transition mutations in bacteria. Nucleic Acids Res.

[1863] Cravens A., Jamil O. K., Kong D., Sockolosky J. T., Smolke C. D. (2021). Polymerase-guided base editing enables
in vivo mutagenesis
and rapid protein engineering. Nat Commun.

[1864] Alvarez B., Mencia M., de Lorenzo V., Fernandez L. A. (2020). In vivo diversification of target genomic sites using
processive base deaminase fusions blocked by dCas9. Nat Commun.

[1865] Long M., Xu M., Qiao Z., Ma Z., Osire T., Yang T., Zhang X., Shao M., Rao Z. (2020). Directed Evolution of Ornithine Cyclodeaminase Using an EvolvR-Based
Growth-Coupling Strategy for Efficient Biosynthesis of l-Proline. ACS Synth Biol.

[1866] Mengiste A. A., McDonald J. L., Nguyen
Tran M. T., Plank A. V., Wilson R. H., Butty V. L., Shoulders M. D. (2024). MutaT7GDE:
A Single Chimera for the Targeted, Balanced, Efficient, and Processive
Installation of All Possible Transition Mutations In Vivo. ACS Synthetic Biology.

[1867] Moore C. L., Papa L. J., Shoulders M. D. (2018). A Processive
Protein Chimera Introduces Mutations across Defined DNA Regions In
Vivo. J Am Chem Soc.

[1868] Chen H., Liu S., Padula S., Lesman D., Griswold K., Lin A., Zhao T., Marshall J. L., Chen F. (2020). Efficient, continuous mutagenesis
in human cells using a pseudo-random
DNA editor. Nat Biotechnol.

[1869] Aune T. E., Aachmann F. L. (2010). Methodologies to
increase the transformation
efficiencies and the range of bacteria that can be transformed. Appl Microbiol Biotechnol.

[1870] Corts, A. D. ; Thomason, L. C. ; Gill, R. T. ; Gralnick, J. A. A new recombineering system for precise genome-editing in Shewanella oneidensis strain MR-1 using single-stranded oligonucleotides. Scientific Reports 2019, 9, 10.1038/s41598-018-37025-4. PMC6328582 30631105

[1871] Walker M. W. G., Klompe S. E., Zhang D. J., Sternberg S. H. (2023). Novel molecular
requirements for CRISPR RNA-guided transposition. Nucleic Acids Res.

[1872] Ferri L., Gori A., Biondi E. G., Mengoni A., Bazzicalupo M. (2010). Plasmid electroporation of Sinorhizobium
strains: The
role of the restriction gene hsdR in type strain Rm1021. Plasmid.

[1873] Thomas C. M., Nielsen K. M. (2005). Mechanisms of, and barriers to, horizontal
gene transfer between bacteria. Nat Rev Microbiol.

[1874] Fischer-Fantuzzi L., Di Girolamo M. (1961). Triparental matings in Escherichia
coli. Genetics.

[1875] Stabb E. V., Ruby E. G. (2002). RP4-based plasmids
for conjugation
between Escherichia coli and members of the Vibrionaceae. Methods Enzymol.

[1876] Brophy J. A. N., Triassi A. J., Adams B. L., Renberg R. L., Stratis-Cullum D. N., Grossman A. D., Voigt C. A. (2018). Engineered integrative
and conjugative elements for efficient and inducible DNA transfer
to undomesticated bacteria. Nature Microbiology.

[1877] Meng D. C., Mukhitov N., Neitzey D., Lucht M., Schaak D. D., Voigt C. A. (2021). Rapid and simultaneous
screening
of pathway designs and chassis organisms, applied to engineered living
materials. Metab. Eng..

[1878] Meng D., Mukhitov N., Neitzey D., Lucht M., Schaak D. D., Voigt C. A. (2021). Rapid and simultaneous
screening
of pathway designs and chassis organisms, applied to engineered living
materials. Metab Eng.

[1879] Chemla Y., Dorfan Y., Yannai A., Meng D., Cao P., Glaven S., Gordon D. B., Elbaz J., Voigt C. A. (2022). Parallel
engineering of environmental bacteria and performance over years under
jungle-simulated conditions. PLoS One.

[1880] Brophy J. A. N., Triassi A. J., Adams B. L., Renberg R. L., Stratis-Cullum D. N., Grossman A. D., Voigt C. A. (2018). Engineered integrative
and conjugative elements for efficient and inducible DNA transfer
to undomesticated bacteria. Nat Microbiol.

[1881] Wu S., Tian P., Tan T. (2022). Genomic landscapes of bacterial transposons
and their applications in strain improvement. Appl Microbiol Biotechnol.

[1882] Wei X. X., Shi Z. Y., Li Z. J., Cai L., Wu Q., Chen G. Q. (2010). A mini-Mu transposon-based method
for multiple DNA
fragment integration into bacterial genomes. Appl Microbiol Biotechnol.

[1883] Choi K. H., Gaynor J. B., White K. G., Lopez C., Bosio C. M., Karkhoff-Schweizer R.
A. R., Schweizer H. P. (2005). A Tn7-based
broad-range bacterial cloning and expression system. Nature Methods.

[1884] Wang G., Zhao Z., Ke J., Engel Y., Shi Y. M., Robinson D., Bingol K., Zhang Z., Bowen B., Louie K. (2019). CRAGE
enables rapid
activation of biosynthetic gene clusters in undomesticated bacteria. Nat Microbiol.

[1885] Matsumoto A., Schluter T., Melkonian K., Takeda A., Nakagami H., Mine A. (2022). A versatile Tn7 transposon-based
bioluminescence tagging tool for quantitative and spatial detection
of bacteria in plants. Plant Commun.

[1886] St-Pierre F., Cui L., Priest D. G., Endy D., Dodd I. B., Shearwin K. E. (2013). One-step cloning
and chromosomal
integration of DNA. ACS Synth Biol.

[1887] Govindarajulu M., Epstein L., Wroblewski T., Michelmore R. W. (2015). Host-induced gene silencing inhibits the biotrophic
pathogen causing downy mildew of lettuce. Plant
Biotechnol J.

[1888] Fogg P. C., Colloms S., Rosser S., Stark M., Smith M. C. (2014). New applications
for phage integrases. J Mol Biol.

[1889] Bosse J. T., Li Y., Leanse L. G., Zhou L., Chaudhuri R. R., Peters S. E., Wang J., Maglennon G. A., Holden M. T. G., Maskell D. J. (2021). Rationally designed
mariner vectors for functional genomic analysis of Actinobacillus
pleuropneumoniae and other Pasteurellaceae species by transposon-directed
insertion-site sequencing (TraDIS). Anim Dis.

[1890] Monson R., Smith D. S., Matilla M. A., Roberts K., Richardson E., Drew A., Williamson N., Ramsay J., Welch M., Salmond G. P. (2015). A Plasmid-Transposon
Hybrid Mutagenesis System Effective in a Broad Range of Enterobacteria. Front Microbiol.

[1891] Nilsson M., Christiansen N., Hoiby N., Twetman S., Givskov M., Tolker-Nielsen T. (2014). A mariner transposon vector adapted
for mutagenesis in oral streptococci. Microbiologyopen.

[1892] Haskett T. L., Karunakaran R., Bueno Batista M., Dixon R., Poole P. S. (2022). Control of nitrogen
fixation and
ammonia excretion in Azorhizobium caulinodans. PLoS Genet.

[1893] Filsinger G. T., Wannier T. M., Pedersen F. B., Lutz I. D., Zhang J., Stork D. A., Debnath A., Gozzi K., Kuchwara H., Volf V. (2021). Characterizing the portability
of phage-encoded homologous recombination proteins. Nat Chem Biol.

[1894] Zhang X. Z., Yan X., Cui Z. L., Hong Q., Li S. P. (2006). mazF, a novel counter-selectable
marker for unmarked chromosomal
manipulation in Bacillus subtilis. Nucleic Acids
Res.

[1895] Jeong D. E., Park S. H., Pan J. G., Kim E. J., Choi S. K. (2015). Genome
engineering using a synthetic gene circuit in
Bacillus subtilis. Nucleic Acids Res.

[1896] Ledermann R., Strebel S., Kampik C., Fischer H. M. (2016). Versatile
Vectors for Efficient Mutagenesis of Bradyrhizobium diazoefficiens
and Other Alphaproteobacteria. Appl Environ
Microbiol.

[1897] Eberhart L. J., Knutson C. M., Barney B. M. (2016). A methodology for
markerless genetic modifications in Azotobacter vinelandii. J Appl Microbiol.

[1898] Suarez G. A., Dugan K. R., Renda B. A., Leonard S. P., Gangavarapu L. S., Barrick J. E. (2020). Rapid and assured
genetic engineering
methods applied to Acinetobacter baylyi ADP1 genome streamlining. Nucleic Acids Res.

[1899] Nyerges A., Csorgo B., Nagy I., Balint B., Bihari P., Lazar V., Apjok G., Umenhoffer K., Bogos B., Posfai G. (2016). A highly
precise and
portable genome engineering method allows comparison of mutational
effects across bacterial species. Proc Natl
Acad Sci U S A.

[1900] Sun Z., Deng A., Hu T., Wu J., Sun Q., Bai H., Zhang G., Wen T. (2015). A high-efficiency
recombineering
system with PCR-based ssDNA in Bacillus subtilis mediated by the native
phage recombinase GP35. Appl Microbiol Biotechnol.

[1901] Wannier T. M., Nyerges A., Kuchwara H. M., Czikkely M., Balogh D., Filsinger G. T., Borders N. C., Gregg C. J., Lajoie M. J., Rios X. (2020). Improved bacterial recombineering
by parallelized protein discovery. Proc Natl
Acad Sci U S A.

[1902] Aparicio T., Jensen S. I., Nielsen A. T., de Lorenzo V., Martinez-Garcia E. (2016). The Ssr protein (T1E_1405) from Pseudomonas
putida
DOT-T1E enables oligonucleotide-based recombineering in platform strain
P. putida EM42. Biotechnol J.

[1903] Ricaurte D. E., Martinez-Garcia E., Nyerges A., Pal C., de Lorenzo V., Aparicio T. (2018). A standardized workflow for surveying
recombinases expands bacterial genome-editing capabilities. Microb Biotechnol.

[1904] Yin J., Zhu H., Xia L., Ding X., Hoffmann T., Hoffmann M., Bian X., Muller R., Fu J., Stewart A. F. (2015). A new
recombineering system for Photorhabdus
and Xenorhabdus. Nucleic Acids Res.

[1905] Jiang W., Bikard D., Cox D., Zhang F., Marraffini L. A. (2013). RNA-guided editing of bacterial genomes
using CRISPR-Cas
systems. Nature Biotechnology.

[1906] Bassalo M. C., Garst A. D., Halweg-Edwards A. L., Grau W. C., Domaille D. W., Mutalik V. K., Arkin A. P., Gill R. T. (2016). Rapid and Efficient One-Step Metabolic Pathway Integration
in E. coli. ACS Synth Biol.

[1907] Ronda C., Pedersen L. E., Sommer M. O., Nielsen A. T. (2016). CRMAGE:
CRISPR Optimized MAGE Recombineering. Sci Rep.

[1908] Qi L. S., Larson M. H., Gilbert L. A., Doudna J. A., Weissman J. S., Arkin A. P., Lim W. A. (2013). Repurposing
CRISPR
as an RNA-guided platform for sequence-specific control of gene expression. Cell.

[1909] Pyne M. E., Moo-Young M., Chung D. A., Chou C. P. (2015). Coupling
the CRISPR/Cas9 System with Lambda Red Recombineering Enables Simplified
Chromosomal Gene Replacement in Escherichia coli. Appl Environ Microbiol.

[1910] Altenbuchner J. (2016). Editing of the Bacillus subtilis
Genome by the CRISPR-Cas9
System. Appl Environ Microbiol.

[1911] So Y., Park S. Y., Park E. H., Park S. H., Kim E. J., Pan J. G., Choi S. K. (2017). A Highly Efficient CRISPR-Cas9-Mediated
Large Genomic Deletion in Bacillus subtilis. Front Microbiol.

[1912] Wang X., Lv S., Liu T., Wei J., Qu S., Lu Y., Zhang J., Oo S., Zhang B., Pan X. (2020). CRISPR/Cas9 genome editing
shows the important role
of AZC_2928 gene in nitrogen-fixing bacteria of plants. Funct Integr Genomics.

[1913] Cobb R. E., Wang Y., Zhao H. (2015). High-efficiency
multiplex
genome editing of Streptomyces species using an engineered CRISPR/Cas
system. ACS Synth Biol.

[1914] Tong Y., Charusanti P., Zhang L., Weber T., Lee S. Y. (2015). CRISPR-Cas9 Based
Engineering of Actinomycetal Genomes. ACS Synth
Biol.

[1915] Jiang D., Zhang D., Li S., Liang Y., Zhang Q., Qin X., Gao J., Qiu J. L. (2022). Highly
efficient genome editing in Xanthomonas oryzae pv. oryzae through
repurposing the endogenous type I-C CRISPR-Cas system. Mol Plant Pathol.

[1916] Xiong B., Li Z., Liu L., Zhao D., Zhang X., Bi C. (2018). Genome editing of Ralstonia
eutropha
using an electroporation-based CRISPR-Cas9 technique. Biotechnol Biofuels.

[1917] Aparicio T., de Lorenzo V., Martinez-Garcia E. (2018). CRISPR/Cas9-Based
Counterselection Boosts Recombineering Efficiency in Pseudomonas putida. Biotechnol J.

[1918] Vento J. M., Crook N., Beisel C. L. (2019). Barriers
to genome
editing with CRISPR in bacteria. J Ind Microbiol
Biotechnol.

[1919] Strecker, J. ; Ladha, A. ; Makarova, K. S. ; Koonin, E. V. ; Zhang, F. Response to Comment on “RNA-guided DNA insertion with CRISPR-associated transposases”. Science 2020, 368 (6495), 10.1126/science.abb2920. 32499411

[1920] Tou C. J., Orr B., Kleinstiver B. P. (2023). Precise
cut-and-paste DNA insertion using engineered type V-K CRISPR-associated
transposases. Nat Biotechnol.

[1921] Vo P. L. H., Ronda C., Klompe S. E., Chen E. E., Acree C., Wang H. H., Sternberg S. H. (2021). CRISPR
RNA-guided integrases for high-efficiency, multiplexed bacterial genome
engineering. Nature Biotechnology.

[1922] Rubin B. E., Diamond S., Cress B. F., Crits-Christoph A., Lou Y. C., Borges A. L., Shivram H., He C., Xu M., Zhou Z. (2022). Species- and site-specific
genome editing in
complex bacterial communities. Nat Microbiol.

[1923] Chen W., Zhang Y., Zhang Y., Pi Y., Gu T., Song L., Wang Y., Ji Q. (2018). CRISPR/Cas9-based Genome
Editing in Pseudomonas aeruginosa and Cytidine Deaminase-Mediated
Base Editing in Pseudomonas Species. iScience.

[1924] Shen X., Wang Z., Huang X., Hu H., Wang W., Zhang X. (2017). Developing genome-reduced Pseudomonas
chlororaphis strains for the production of secondary metabolites. BMC Genomics.

[1925] Wang Y., Liu Y., Zheng P., Sun J., Wang M. (2021). Microbial Base Editing: A Powerful Emerging Technology for Microbial
Genome Engineering. Trends Biotechnol.

[1926] Rees H. A., Liu D. R. (2018). Base editing: precision chemistry
on the genome and transcriptome of living cells. Nat Rev Genet.

[1927] Molla K. A., Yang Y. (2019). CRISPR/Cas-Mediated
Base Editing:
Technical Considerations and Practical Applications. Trends Biotechnol.

[1928] Tong Y., Whitford C. M., Robertsen H. L., Blin K., Jorgensen T. S., Klitgaard A. K., Gren T., Jiang X., Weber T., Lee S. Y. (2019). Highly
efficient DSB-free base editing for streptomycetes with CRISPR-BEST. Proc Natl Acad Sci U S A.

[1929] Wang, Y. ; Wang, S. ; Chen, W. ; Song, L. ; Zhang, Y. ; Shen, Z. ; Yu, F. ; Li, M. ; Ji, Q. CRISPR-Cas9 and CRISPR-Assisted Cytidine Deaminase Enable Precise and Efficient Genome Editing in Klebsiella pneumoniae. Appl Environ Microbiol 2018, 84 (23), 10.1128/AEM.01834-18. PMC6238054 30217854

[1930] Bonde M. T., Kosuri S., Genee H. J., Sarup-Lytzen K., Church G. M., Sommer M. O., Wang H. H. (2015). Direct mutagenesis
of thousands of genomic targets using microarray-derived oligonucleotides. ACS Synth Biol.

[1931] Singh V., Braddick D. (2015). Recent advances and
versatility of
MAGE towards industrial applications. Syst Synth
Biol.

[1932] Aparicio T., Nyerges A., Martinez-Garcia E., de Lorenzo V. (2020). High-Efficiency Multi-site Genomic Editing of Pseudomonas
putida through Thermoinducible ssDNA Recombineering. iScience.

[1933] Asin-Garcia E., Martin-Pascual M., Garcia-Morales L., van Kranenburg R., Martins Dos Santos V.
A. P. (2021). ReScribe: An Unrestrained
Tool Combining Multiplex Recombineering and Minimal-PAM ScCas9 for
Genome Recoding Pseudomonas putida. ACS Synth
Biol.

[1934] Isaacs F. J., Carr P. A., Wang H. H., Lajoie M. J., Sterling B., Kraal L., Tolonen A. C., Gianoulis T. A., Goodman D. B., Reppas N. B. (2011). Precise manipulation
of chromosomes in vivo enables genome-wide codon replacement. Science.

[1935] Summerer D., Chen S., Wu N., Deiters A., Chin J. W., Schultz P. G. (2006). A genetically encoded
fluorescent
amino acid. Proc Natl Acad Sci U S A.

[1936] Rogers J. M., Suga H. (2015). Discovering functional, non-proteinogenic
amino acid containing, peptides using genetic code reprogramming. Org Biomol Chem.

[1937] Saleh A. M., Wilding K. M., Calve S., Bundy B. C., Kinzer-Ursem T. L. (2019). Non-canonical amino acid labeling
in proteomics and
biotechnology. J Biol Eng.

[1938] Lee J., Schwarz K. J., Kim D. S., Moore J. S., Jewett M. C. (2020). Ribosome-mediated
polymerization of long chain carbon and cyclic amino acids into peptides
in vitro. Nat Commun.

[1939] Lee J., Torres R., Kim D. S., Byrom M., Ellington A. D., Jewett M. C. (2020). Ribosomal incorporation
of cyclic beta-amino acids
into peptides using in vitro translation. Chem
Commun (Camb).

[1940] Goto Y., Suga H. (2009). Translation initiation with initiator
tRNA charged with exotic peptides. J Am Chem
Soc.

[1941] Leisle L., Valiyaveetil F., Mehl R. A., Ahern C. A. (2015). Incorporation
of Non-Canonical Amino Acids. Adv Exp Med Biol.

[1942] Perez J. G., Carlson E. D., Weisser O., Kofman C., Seki K., Des Soye B. J., Karim A. S., Jewett M. C. (2022). Improving
genomically recoded Escherichia coli to produce proteins containing
non-canonical amino acids. Biotechnol J.

[1943] He J., Van Treeck B., Nguyen H. B., Melancon C. E. (2016). Development of an Unnatural Amino Acid Incorporation System in the
Actinobacterial Natural Product Producer Streptomyces venezuelae ATCC
15439. ACS Synth Biol.

[1944] Stork D. A., Squyres G. R., Kuru E., Gromek K. A., Rittichier J., Jog A., Burton B. M., Church G. M., Garner E. C., Kunjapur A. M. (2021). Designing efficient
genetic code
expansion in Bacillus subtilis to gain biological insights. Nat Commun.

[1945] Zheng H., Lin S., Chen P. R. (2020). Genetically encoded
protein labeling and crosslinking in living Pseudomonas aeruginosa. Bioorg Med Chem.

[1946] Xu C., Zou Q., Tian J., Li M., Xing B., Gong J., Wang J., Huo Y. X., Guo S. (2023). Simplified
Construction of Engineered Bacillus subtilis Host for Improved Expression
of Proteins Harboring Noncanonical Amino Acids. ACS Synth Biol.

[1947] He X., Gao T., Chen Y., Liu K., Guo J., Niu W. (2022). Genetic Code Expansion in Pseudomonas putida KT2440. ACS Synth Biol.

[1948] Ma N. J., Moonan D. W., Isaacs F. J. (2014). Precise
manipulation
of bacterial chromosomes by conjugative assembly genome engineering. Nat Protoc.

[1949] Wang K., Fredens J., Brunner S. F., Kim S. H., Chia T., Chin J. W. (2016). Defining synonymous
codon compression
schemes by genome recoding. Nature.

[1950] Robertson W. E., Funke L. F. H., de la Torre D., Fredens J., Wang K., Chin J. W. (2021). Creating custom
synthetic genomes in Escherichia coli with REXER and GENESIS. Nat Protoc.

[1951] Zhang W., Mitchell L. A., Bader J. S., Boeke J. D. (2020). Synthetic
Genomes. Annu Rev Biochem.

[1952] Venetz J. E., Del Medico L., Wolfle A., Schachle P., Bucher Y., Appert D., Tschan F., Flores-Tinoco C. E., van Kooten M., Guennoun R. (2019). Chemical synthesis rewriting
of a bacterial genome to achieve design flexibility and biological
functionality. Proc Natl Acad Sci U S A.

[1953] Itaya M., Tsuge K., Koizumi M., Fujita K. (2005). Combining
two genomes in one cell: stable cloning of the Synechocystis PCC6803
genome in the Bacillus subtilis 168 genome. Proc Natl Acad Sci U S A.

[1954] Itaya M., Fujita K., Kuroki A., Tsuge K. (2008). Bottom-up
genome assembly using the Bacillus subtilis genome vector. Nat Methods.

[1955] Xu X., Meier F., Blount B. A., Pretorius I. S., Ellis T., Paulsen I. T., Williams T. C. (2023). Trimming the genomic
fat: minimising and re-functionalising genomes using synthetic biology. Nat Commun.

[1956] Li J., Mau R. L., Dijkstra P., Koch B. J., Schwartz E., Liu X. A., Morrissey E. M., Blazewicz S. J., Pett-Ridge J., Stone B. W. (2019). Predictive
genomic traits
for bacterial growth in culture versus actual growth in soil. ISME J.

[1957] Baba T., Ara T., Hasegawa M., Takai Y., Okumura Y., Baba M., Datsenko K. A., Tomita M., Wanner B. L., Mori H. (2006). Construction of Escherichia
coli
K-12 in-frame, single-gene knockout mutants: the Keio collection. Mol Syst Biol.

[1958] Iwadate Y., Honda H., Sato H., Hashimoto M., Kato J. (2011). Oxidative stress sensitivity of engineered Escherichia coli cells
with a reduced genome. FEMS Microbiol Lett.

[1959] Martinez-Garcia E., Nikel P. I., Aparicio T., de Lorenzo V. (2014). Pseudomonas
2.0: genetic upgrading of P. putida KT2440 as an enhanced host for
heterologous gene expression. Microb Cell Fact.

[1960] Lieder S., Nikel P. I., de Lorenzo V., Takors R. (2015). Genome reduction boosts heterologous gene expression
in Pseudomonas putida. Microb Cell Fact.

[1961] Fan X., Zhang Y., Zhao F., Liu Y., Zhao Y., Wang S., Liu R., Yang C. (2020). Genome reduction
enhances
production of polyhydroxyalkanoate and alginate oligosaccharide in
Pseudomonas mendocina. Int J Biol Macromol.

[1962] Suarez R. A., Stulke J., van Dijl J. M. (2019). Less Is
More: Toward
a Genome-Reduced Bacillus Cell Factory for “Difficult Proteins”. ACS Synth. Biol..

[1963] Suarez, R. A. ; Antelo-Varela, M. ; Maass, S. ; Neef, J. ; Becher, D. ; van Dijl, J. M. Redirected Stress Responses in a Genome-Minimized 'midiBacillus' Strain with Enhanced Capacity for Protein Secretion. Msystems 2021, 6 (6), 10.1128/mSystems.00655-21. PMC8670375 34904864

[1964] Choe D., Cho S., Kim S. C., Cho B. K. (2016). Minimal
genome: Worthwhile or worthless efforts toward being smaller?. Biotechnol J.

[1965] Moger-Reischer R. Z., Glass J. I., Wise K. S., Sun L., Bittencourt D. M. C., Lehmkuhl B. K., Schoolmaster D. R., Lynch M., Lennon J. T. (2023). Evolution of a minimal
cell. Nature.

[1966] Choe D., Lee J. H., Yoo M., Hwang S., Sung B. H., Cho S., Palsson B., Kim S. C., Cho B. K. (2019). Adaptive laboratory evolution of
a genome-reduced Escherichia
coli. Nat Commun.

[1967] Bayer C. N., Rennig M., Ehrmann A. K., Norholm M. H. H. (2021). A standardized
genome architecture for bacterial synthetic biology (SEGA). Nat Commun.

[1968] Bourgeois L., Pyne M. E., Martin V. J. J. (2018). A Highly Characterized
Synthetic Landing Pad System for Precise Multicopy Gene Integration
in Yeast. ACS Synth Biol.

[1969] Bryant J. A., Sellars L. E., Busby S. J., Lee D. J. (2014). Chromosome
position effects on gene expression in Escherichia coli K-12. Nucleic Acids Res.

[1970] Lato D. F., Golding G. B. (2020). Spatial Patterns
of Gene Expression
in Bacterial Genomes. J Mol Evol.

[1971] Englaender J. A., Jones J. A., Cress B. F., Kuhlman T. E., Linhardt R. J., Koffas M. A. G. (2017). Effect of Genomic
Integration Location
on Heterologous Protein Expression and Metabolic Engineering in E.
coli. ACS Synth Biol.

[1972] Sauer C., Syvertsson S., Bohorquez L. C., Cruz R., Harwood C. R., van Rij T., Hamoen L. W. (2016). Effect
of Genome Position on Heterologous Gene Expression in Bacillus subtilis:
An Unbiased Analysis. ACS Synth Biol.

[1973] Biggs B. W., Bedore S. R., Arvay E., Huang S., Subramanian H., McIntyre E. A., Duscent-Maitland C. V., Neidle E. L., Tyo K. E. J. (2020). Development
of a genetic toolset
for the highly engineerable and metabolically versatile Acinetobacter
baylyi ADP1. Nucleic Acids Res.

[1974] Jeong D. E., So Y., Park S. Y., Park S. H., Choi S. K. (2018). Random knock-in
expression system for high yield production
of heterologous protein in Bacillus subtilis. J Biotechnol.

[1975] Taketani M., Zhang J., Zhang S., Triassi A. J., Huang Y. J., Griffith L. G., Voigt C. A. (2020). Genetic circuit
design automation for the gut resident species Bacteroides thetaiotaomicron. Nat Biotechnol.

[1976] Bernhards C. B., Liem A. T., Berk K. L., Roth P. A., Gibbons H. S., Lux M. W. (2022). Putative Phenotypically
Neutral Genomic
Insertion Points in Prokaryotes. ACS Synth Biol.

[1977] Hasnain A. (2019). A data-driven
method for quantifying the impact of
a genetic circuit on its host. BioCAS.

[1978] Zobel S., Benedetti I., Eisenbach L., De Lorenzo V., Wierckx N., Blank L. M. (2015). Tn7-Based Device
for Calibrated Heterologous Gene Expression in Pseudomonas putida. ACS Synth. Biol..

[1979] Ke J., Zhao Z., Coates C. R., Hadjithomas M., Kuftin A., Louie K., Weller D., Thomashow L., Mouncey N. J., Northen T. R. (2022). Development
of platforms
for functional characterization and production of phenazines using
a multi-chassis approach via CRAGE. Metab Eng.

[1980] Zhao Z., Cheng J. F., Yoshikuni Y. (2022). Chromosomal
integration of complex DNA constructs using CRAGE and CRAGE-Duet systems. STAR Protoc.

[1981] Wang B., Zhao Z., Jabusch L. K., Chiniquy D. M., Ono K., Conway J. M., Zhang Z., Wang G., Robinson D., Cheng J. F. (2020). CRAGE-Duet Facilitates Modular Assembly of
Biological Systems for Studying Plant-Microbe Interactions. ACS Synth Biol.

[1982] Mutalik V. K., Guimaraes J. C., Cambray G., Mai Q. A., Christoffersen M. J., Martin L., Yu A., Lam C., Rodriguez C., Bennett G. (2013). Quantitative estimation
of activity and quality for collections of functional genetic elements. Nature Methods.

[1983] Jack B. R., Leonard S. P., Mishler D. M., Renda B. A., Leon D., Suarez G. A., Barrick J. E. (2015). Predicting
the Genetic
Stability of Engineered DNA Sequences with the EFM Calculator. ACS Synth. Biol..

[1984] Kang D. H., Ko S. C., Heo Y. B., Lee H. J., Woo H. M. (2022). RoboMoClo: A Robotics-Assisted Modular
Cloning Framework
for Multiple Gene Assembly in Biofoundry. ACS
Synth Biol.

[1985] Chao R., Mishra S., Si T., Zhao H. (2017). Engineering
biological systems using automated biofoundries. Metab Eng.

[1986] Hillson N., Caddick M., Cai Y., Carrasco J. A., Chang M. W., Curach N. C., Bell D. J., Le Feuvre R., Friedman D. C., Fu X. (2019). Building a global alliance
of biofoundries. Nat Commun.

[1987] Baker D., Church G., Collins J., Endy D., Jacobson J., Keasling J., Modrich P., Smolke C., Weiss R. (2006). Engineering life: building a fab
for biology. Sci Am.

[1988] Espah Borujeni A., Zhang J., Doosthosseini H., Nielsen A. A. K., Voigt C. A. (2020). Genetic circuit characterization
by inferring RNA polymerase movement and ribosome usage. Nat Commun.

[1989] Gorochowski, T. E. ; Borujeni, A. E. ; Park, Y. ; Nielsen, A. A. K. ; Zhang, J. ; Der, B. S. ; Gordon, D. B. ; Voigt, C. A. Genetic circuit characterization and debugging using RNA-seq. Molecular Systems Biology 2017, 13, 1-16 10.15252/msb.20167461. PMC5731345 29122925

[1990] Borujeni, A. E. ; Zhang, J. ; Doosthosseini, H. ; Nielsen, A. A. K. ; Voigt, C. A. Genetic circuit characterization by inferring RNA polymerase movement and ribosome usage. Nature Communications 2020, 11 (1), 10.1038/s41467-020-18630-2. PMC7536230 33020480

[1991] Iyer S., Park B. R., Kim M. (2016). Absolute quantitative
measurement of transcriptional kinetic parameters in vivo. Nucleic Acids Res.

[1992] Friedman L. J., Gelles J. (2012). Mechanism of transcription
initiation
at an activator-dependent promoter defined by single-molecule observation. Cell.

[1993] Rhodius V. A., Mutalik V. K., Gross C. A. (2012). Predicting the strength
of UP-elements and full-length E. coli sigmaE promoters. Nucleic Acids Res.

[1994] Davis J. H., Rubin A. J., Sauer R. T. (2011). Design,
construction
and characterization of a set of insulated bacterial promoters. Nucleic Acids Res.

[1995] Wang W., Li Y., Wang Y., Shi C., Li C., Li Q., Linhardt R. J. (2018). Bacteriophage T7
transcription system:
an enabling tool in synthetic biology. Biotechnol
Adv.

[1996] Meyer A. J., Ellefson J. W., Ellington A. D. (2015). Directed
Evolution of a Panel of Orthogonal T7 RNA Polymerase Variants for
in Vivo or in Vitro Synthetic Circuitry. ACS
Synth Biol.

[1997] Jones J. A., Vernacchio V. R., Lachance D. M., Lebovich M., Fu L., Shirke A. N., Schultz V. L., Cress B., Linhardt R. J., Koffas M. A. (2015). ePathOptimize: A Combinatorial Approach for Transcriptional
Balancing of Metabolic Pathways. Sci Rep.

[1998] Komura R., Aoki W., Motone K., Satomura A., Ueda M. (2018). High-throughput evaluation of T7
promoter variants using biased randomization
and DNA barcoding. PLoS One.

[1999] Lammens, E. M. ; Feyaerts, N. ; Kerremans, A. ; Boon, M. ; Lavigne, R. Assessing the Orthogonality of Phage-Encoded RNA Polymerases for Tailored Synthetic Biology Applications in Pseudomonas Species. Int J Mol Sci 2023, 24 (8), 7175 10.3390/ijms24087175. 37108338 PMC10138996

[2000] Zhao H., Zhang H. M., Chen X., Li T., Wu Q., Ouyang Q., Chen G. Q. (2017). Novel T7-like expression
systems
used for Halomonas. Metab Eng.

[2001] Conrad T., Plumbom I., Alcobendas M., Vidal R., Sauer S. (2020). Maximizing transcription of nucleic
acids with efficient T7 promoters. Commun Biol.

[2002] Senda N., Enomoto T., Kihara K., Yamashiro N., Takagi N., Kiga D., Nishida H. (2022). Development
of an expression-tunable
multiple protein synthesis system in cell-free reactions using T7-promoter-variant
series. Synth Biol (Oxf).

[2003] Kushwaha M., Salis H. M. (2015). A portable expression
resource for
engineering cross-species genetic circuits and pathways. Nat Commun.

[2004] Liang X., Li C., Wang W., Li Q. (2018). Integrating
T7 RNA Polymerase and Its Cognate Transcriptional Units for a Host-Independent
and Stable Expression System in Single Plasmid. ACS Synth Biol.

[2005] Ingolia N. T., Ghaemmaghami S., Newman J. R., Weissman J. S. (2009). Genome-wide
analysis in vivo of translation with nucleotide resolution using ribosome
profiling. Science.

[2006] Brar G. A., Weissman J. S. (2015). Ribosome profiling
reveals the what,
when, where and how of protein synthesis. Nat
Rev Mol Cell Biol.

[2007] Guiziou S., Sauveplane V., Chang H. J., Clerté C., Declerck N., Jules M., Bonnet J. (2016). A part toolbox to tune
genetic expression in Bacillus subtilis. Nucleic
Acids Research.

[2008] Zhang B., Zhou N., Liu Y. M., Liu C., Lou C. B., Jiang C. Y., Liu S. J. (2015). Ribosome binding
site libraries and pathway modules for shikimic acid synthesis with
Corynebacterium glutamicum. Microb Cell Fact.

[2009] Tian T., Salis H. M. (2015). A predictive biophysical
model of
translational coupling to coordinate and control protein expression
in bacterial operons. Nucleic Acids Res.

[2010] Na D., Lee D. (2010). RBSDesigner: software for designing synthetic ribosome
binding sites that yields a desired level of protein expression. Bioinformatics.

[2011] Espah Borujeni A., Channarasappa A. S., Salis H. M. (2014). Translation rate
is controlled by coupled trade-offs between site accessibility, selective
RNA unfolding and sliding at upstream standby sites. Nucleic Acids Res.

[2012] Renda, A. ; Poly, S. ; Lai, Y. J. ; Pannuri, A. ; Yakhnin, H. ; Potts, A. H. ; Bevilacqua, P. C. ; Romeo, T. ; Babitzke, P. CsrA-Mediated Translational Activation of ymdA Expression in Escherichia coli. mBio 2020, 11 (5), 10.1128/mBio.00849-20. PMC7492729 32934077

[2013] Morita T., Mochizuki Y., Aiba H. (2006). Translational repression
is sufficient for gene silencing by bacterial small noncoding RNAs
in the absence of mRNA destruction. Proc Natl
Acad Sci U S A.

[2014] Wang H., Li J., Jewett M. C. (2018). Development
of a
Pseudomonas putida cell-free protein synthesis platform for rapid
screening of gene regulatory elements. Synth
Biol (Oxf).

[2015] Volkenborn K., Kuschmierz L., Benz N., Lenz P., Knapp A., Jaeger K. E. (2020). The length of ribosomal binding site
spacer sequence controls the production yield for intracellular and
secreted proteins by Bacillus subtilis. Microb
Cell Fact.

[2016] Immethun C. M., Kathol M., Changa T., Saha R. (2022). Synthetic
Biology Tool Development Advances Predictable Gene Expression in the
Metabolically Versatile Soil Bacterium Rhodopseudomonas palustris. Front Bioeng Biotechnol.

[2017] Xu K., Tong Y., Li Y., Tao J., Rao S., Li J., Zhou J., Liu S. (2022). Autoinduction Expression Modules
for Regulating Gene Expression in Bacillus subtilis. ACS Synth Biol.

[2018] Goodman D. B., Church G. M., Kosuri S. (2013). Causes and
effects
of N-terminal codon bias in bacterial genes. Science.

[2019] Clifton K. P., Jones E. M., Paudel S., Marken J. P., Monette C. E., Halleran A. D., Epp L., Saha M. S. (2018). The genetic
insulator RiboJ increases expression of insulated genes. J Biol Eng.

[2020] Bartoli V., Meaker G. A., Di Bernardo M., Gorochowski T. E. (2020). Tunable genetic devices through simultaneous control
of transcription and translation. Nature Communications.

[2021] Bai C., Zhang Y., Zhao X., Hu Y., Xiang S., Miao J., Lou C., Zhang L. (2015). Exploiting
a precise
design of universal synthetic modular regulatory elements to unlock
the microbial natural products in Streptomyces. Proc Natl Acad Sci U S A.

[2022] Neves D., Vos S., Blank L. M., Ebert B. E. (2020). Pseudomonas
mRNA 2.0: Boosting Gene Expression Through Enhanced mRNA Stability
and Translational Efficiency. Front Bioeng Biotechnol.

[2023] de Hoon M. J., Makita Y., Nakai K., Miyano S. (2005). Prediction
of transcriptional terminators in Bacillus subtilis and related species. PLoS Comput Biol.

[2024] Hudson A. J., Wieden H. J. (2019). Rapid generation
of sequence-diverse
terminator libraries and their parameterization using quantitative
Term-Seq. Synth Biol (Oxf).

[2025] Schuster L. A., Reisch C. R. (2021). A plasmid toolbox
for controlled
gene expression across the Proteobacteria. Nucleic
Acids Res.

[2026] MacLellan S. R., MacLean A. M., Finan T. M. (2006). Promoter prediction
in the rhizobia. Microbiology (Reading).

[2027] Ryu M. H., Zhang J., Toth T., Khokhani D., Geddes B. A., Mus F., Garcia-Costas A., Peters J. W., Poole P. S., Ane J. M. (2020). Control
of nitrogen fixation in bacteria that associate with cereals. Nat Microbiol.

[2028] Venkataraman M., Ynigez-Gutierrez A., Infante V., MacIntyre A., Fernandes-Junior P. I., Ane J. M., Pfleger B. (2023). Synthetic Biology Toolbox
for Nitrogen-Fixing Soil Microbes. ACS Synth
Biol.

[2029] Kulakowski, S. ; Rivier, A. ; Kuo, R. ; Mengel, S. ; Eng, T. Development of modular expression across phylogenetically distinct diazotrophs. J Ind Microbiol Biotechnol 2024, 51, 10.1093/jimb/kuae033. PMC11537724 39257030

[2030] Liao Y., Huang L., Wang B., Zhou F., Pan L. (2015). The global transcriptional landscape of Bacillus amyloliquefaciens
XH7 and high-throughput screening of strong promoters based on RNA-seq
data. Gene.

[2031] Han L., Cui W., Suo F., Miao S., Hao W., Chen Q., Guo J., Liu Z., Zhou L., Zhou Z. (2019). Development of a novel strategy for
robust synthetic bacterial promoters
based on a stepwise evolution targeting the spacer region of the core
promoter in Bacillus subtilis. Microb Cell Fact.

[2032] Liu D., Mao Z., Guo J., Wei L., Ma H., Tang Y., Chen T., Wang Z., Zhao X. (2018). Construction,
Model-Based Analysis, and Characterization of a Promoter Library for
Fine-Tuned Gene Expression in Bacillus subtilis. ACS Synth Biol.

[2033] Song Y., Nikoloff J. M., Fu G., Chen J., Li Q., Xie N., Zheng P., Sun J., Zhang D. (2016). Promoter Screening
from Bacillus subtilis in Various Conditions Hunting for Synthetic
Biology and Industrial Applications. PLoS One.

[2035] Popp P.
F., Dotzler M., Radeck J., Bartels J., Mascher T. (2017). The Bacillus BioBrick
Box 2.0: expanding the genetic
toolbox for the standardized work with Bacillus subtilis. Sci Rep.

[2036] Yang S., Du G., Chen J., Kang Z. (2017). Characterization
and application of endogenous phase-dependent promoters in Bacillus
subtilis. Appl Microbiol Biotechnol.

[2037] Radeck J., Kraft K., Bartels J., Cikovic T., Durr F., Emenegger J., Kelterborn S., Sauer C., Fritz G., Gebhard S. (2013). The Bacillus
BioBrick Box: generation and evaluation of essential genetic building
blocks for standardized work with Bacillus subtilis. J Biol Eng.

[2038] Falkenberg K. B., Mol V., de la Maza Larrea A. S., Pogrebnyakov I., Norholm M. H. H., Nielsen A. T., Jensen S. I. (2021). The ProUSER2.0
Toolbox: Genetic Parts and Highly Customizable Plasmids for Synthetic
Biology in Bacillus subtilis. ACS Synth Biol.

[2039] Gonzalez L. M., Mukhitov N., Voigt C. A. (2020). Resilient living
materials built by printing bacterial spores. Nat Chem Biol.

[2040] Wang J., Ai X., Mei H., Fu Y., Chen B., Yu Z., He J. (2013). High-throughput identification
of promoters and screening of highly active promoter-5'-UTR DNA
region
with different characteristics from Bacillus thuringiensis. PLoS One.

[2041] Tang Q., Lu T., Liu S. J. (2018). Developing a Synthetic
Biology Toolkit for Comamonas testosteroni, an Emerging Cellular Chassis
for Bioremediation. ACS Synth Biol.

[2042] Li H., Liao J. C. (2015). A synthetic anhydrotetracycline-controllable
gene expression
system in Ralstonia eutropha H16. ACS Synth
Biol.

[2043] Bi C., Su P., Muller J., Yeh Y. C., Chhabra S. R., Beller H. R., Singer S. W., Hillson N. J. (2013). Development of a
broad-host synthetic biology toolbox for Ralstonia eutropha and its
application to engineering hydrocarbon biofuel production. Microb Cell Fact.

[2044] Jiang X., Zhu C., Lin J., Li J., Fu S., Gong H. (2016). Vector promoters
used in Klebsiella pneumoniae. Biotechnol Appl
Biochem.

[2045] Elmore J. R., Furches A., Wolff G. N., Gorday K., Guss A. M. (2017). Development
of a high efficiency integration system
and promoter library for rapid modification of Pseudomonas putida
KT2440. Metab Eng Commun.

[2046] Zobel S., Benedetti I., Eisenbach L., de Lorenzo V., Wierckx N., Blank L. M. (2015). Tn7-Based
Device
for Calibrated Heterologous Gene Expression in Pseudomonas putida. ACS Synth Biol.

[2047] Calero P., Jensen S. I., Nielsen A. T. (2016). Broad-Host-Range
ProUSER Vectors Enable Fast Characterization of Inducible Promoters
and Optimization of p-Coumaric Acid Production in Pseudomonas putida
KT2440. ACS Synth Biol.

[2048] Gauttam R., Mukhopadhyay A., Simmons B. A., Singer S. W. (2021). Development
of dual-inducible duet-expression vectors for tunable gene expression
control and CRISPR interference-based gene repression in Pseudomonas
putida KT2440. Microb Biotechnol.

[2049] Sathesh-Prabu C., Tiwari R., Kim D., Lee S. K. (2021). Inducible
and tunable gene expression systems for Pseudomonas putida KT2440. Sci Rep.

[2050] Cook T. B., Rand J. M., Nurani W., Courtney D. K., Liu S. A., Pfleger B. F. (2018). Genetic tools for reliable gene expression
and recombineering in Pseudomonas putida. J
Ind Microbiol Biotechnol.

[2051] Siegl T., Tokovenko B., Myronovskyi M., Luzhetskyy A. (2013). Design, construction and characterisation
of a synthetic
promoter library for fine-tuned gene expression in actinomycetes. Metab Eng.

[2052] Cao Y., Song M., Li F., Li C., Lin X., Chen Y., Chen Y., Xu J., Ding Q., Song H. (2019). A Synthetic Plasmid Toolkit for Shewanella oneidensis MR-1. Front Microbiol.

[2053] Luo Y., Zhang L., Barton K. W., Zhao H. (2015). Systematic Identification
of a Panel of Strong Constitutive Promoters from Streptomyces albus. ACS Synth Biol.

[2054] Ji C. H., Kim J. P., Kang H. S. (2018). Library
of Synthetic
Streptomyces Regulatory Sequences for Use in Promoter Engineering
of Natural Product Biosynthetic Gene Clusters. ACS Synth Biol.

[2055] Yi J. S., Kim M. W., Kim M., Jeong Y., Kim E. J., Cho B. K., Kim B. G. (2017). A Novel
Approach
for Gene Expression Optimization through Native Promoter and 5'
UTR
Combinations Based on RNA-seq, Ribo-seq, and TSS-seq of Streptomyces
coelicolor. ACS Synth Biol.

[2056] Seghezzi N., Amar P., Koebmann B., Jensen P. R., Virolle M. J. (2011). The construction of a library of
synthetic promoters
revealed some specific features of strong Streptomyces promoters. Appl Microbiol Biotechnol.

[2057] Horbal L., Siegl T., Luzhetskyy A. (2018). A set of synthetic
versatile genetic control elements for the efficient expression of
genes in Actinobacteria. Sci Rep.

[2058] Phelan R. M., Sachs D., Petkiewicz S. J., Barajas J. F., Blake-Hedges J. M., Thompson M. G., Reider
Apel A., Rasor B. J., Katz L., Keasling J. D. (2017). Development of Next
Generation Synthetic Biology Tools for Use in Streptomyces venezuelae. ACS Synth Biol.

[2059] Hu Z., Li H., Weng Y., Li P., Zhang C., Xiao D. (2020). Improve the production of D-limonene
by regulating the mevalonate
pathway of Saccharomyces cerevisiae during alcoholic beverage fermentation. J Ind Microbiol Biotechnol.

[2060] Chen H., Li M., Liu C., Zhang H., Xian M., Liu H. (2018). Enhancement of the
catalytic activity
of Isopentenyl diphosphate isomerase (IDI) from Saccharomyces cerevisiae
through random and site-directed mutagenesis. Microb Cell Fact.

[2061] Coelho P. S., Brustad E. M., Kannan A., Arnold F. H. (2013). Olefin
cyclopropanation via carbene transfer catalyzed by engineered cytochrome
P450 enzymes. Science.

[2062] Wrublewski D. T. (2019). Analysis for Science Librarians of
the 2018 Nobel Prize
in Chemistry: Directed Evolution of Enzymes and Phage Display of Peptides
and Antibodies. Science & Technology Libraries.

[2063] Iqbal M., Marman M., Arintya F., Broms K., Clark T., Srigiriraju L. (2023). Mating disruption technology: An
innovative tool for managing yellow stem borer (Scirpophaga incertulas
Walker) of rice in Indonesia: Teknologi gangguan kawin: Inovasi untuk
pengendalian penggerek batang kuning (Scirpophaga incertulas Walker)
pada padi di Indonesia. Jurnal Entomologi Indonesia.

[2064] Fischbach M. A., Lai J. R., Roche E. D., Walsh C. T., Liu D. R. (2007). Directed
evolution can rapidly improve the activity
of chimeric assembly-line enzymes. Proceedings
of the National Academy of Sciences.

[2065] Torres M. J., Brandan C. P., Sabaté D. C., Petroselli G., Erra-Balsells R., Audisio M. C. (2017). Biological activity
of the lipopeptide-producing Bacillus amyloliquefaciens PGPBacCA1
on common bean Phaseolus vulgaris L. pathogens. Biological Control.

[2066] Hao W., Cui W., Suo F., Han L., Cheng Z., Zhou Z. (2022). Construction and application of an
efficient dual-base editing platform
for Bacillus subtilis evolution employing programmable base conversion. Chem Sci.

[2067] Naranjo H. D., Rat A., De Zutter N., De Ridder E., Lebbe L., Audenaert K., Willems A. (2023). Uncovering Genomic Features and Biosynthetic Gene Clusters
in Endophytic Bacteria from Roots of the Medicinal Plant Alkanna tinctoria
Tausch as a Strategy To Identify Novel Biocontrol Bacteria. Microbiol Spectr.

[2068] Gao J. L., Sun P., Sun Y. C., Xue J., Wang G., Wang L. W., Du Y., Zhang X., Sun J. G. (2021). Caulobacter endophyticus sp. nov., an endophytic bacterium
harboring three lasso peptide biosynthetic gene clusters and producing
indoleacetic acid isolated from maize root. Antonie Van Leeuwenhoek.

[2069] Zboralski A., Biessy A., Filion M. (2023). Genome exploration
and ecological competence are key to developing effective Pseudomonas-based
biocontrol inoculants. Canadian Journal of Plant
Pathology.

[2070] Liu D., Yan R., Fu Y., Wang X., Zhang J., Xiang W. (2019). Antifungal, Plant Growth-Promoting,
and Genomic Properties of an
Endophytic Actinobacterium Streptomyces sp. NEAU-S7GS2. Front Microbiol.

[2071] Shih, S. Y. ; Huang, Y. S. ; Chou, K. R. ; Wu, H. Y. ; Tsai, H. Isolation and genome characterization of Paenibacillus polymyxa 188, a potential biocontrol agent against fungi. J Appl Microbiol 2024, 135 (4), 10.1093/jambio/lxae075. 38509027

[2072] Nonthakaew N., Panbangred W., Songnuan W., Intra B. (2022). Plant growth-promoting
properties of Streptomyces spp. isolates and their impact on mung
bean plantlets' rhizosphere microbiome. Front
Microbiol.

[2073] Smanski M. J., Zhou H., Claesen J., Shen B., Fischbach M. A., Voigt C. A. (2016). Synthetic biology
to access and expand
nature's chemical diversity. Nat Rev Microbiol.

[2074] Fischbach M., Voigt C. A. (2010). Prokaryotic gene clusters: a rich
toolbox for synthetic biology. Biotechnol J.

[2075] Li Y. F., Tsai K. J. S., Harvey C. J. B., Li J. J., Ary B. E., Berlew E. E., Boehman B. L., Findley D. M., Friant A. G., Gardner C. A. (2016). Comprehensive curation
and analysis of fungal biosynthetic gene clusters of published natural
products. Fungal Genet Biol.

[2076] Robey, M. T. ; Caesar, L. K. ; Drott, M. T. ; Keller, N. P. ; Kelleher, N. L. An interpreted atlas of biosynthetic gene clusters from 1,000 fungal genomes. Proc Natl Acad Sci U S A 2021, 118 (19), 10.1073/pnas.2020230118. PMC8126772 33941694

[2077] Nutzmann H. W., Osbourn A. (2014). Gene clustering in
plant specialized
metabolism. Curr Opin Biotechnol.

[2078] Kautsar S. A., Suarez Duran H. G., Blin K., Osbourn A., Medema M. H. (2017). plantiSMASH: automated
identification, annotation and
expression analysis of plant biosynthetic gene clusters. Nucleic Acids Res.

[2079] Kirst H. A. (2010). The spinosyn family of insecticides:
realizing the
potential of natural products research. J Antibiot
(Tokyo).

[2080] Ramachanderan R., Schaefer B. (2020). Spinosyn insecticides. ChemTexts.

[2081] He H., Tang J., Chen J., Hu J., Zhu Z., Liu Y., Shuai L., Cao L., Liu Z., Xia Z. (2021). Flaviolin-Like Gene Cluster Deletion Optimized
the Butenyl-Spinosyn
Biosynthesis Route in Saccharopolyspora pogona. ACS Synth Biol.

[2082] Sheehan L. S., Lill R. E., Wilkinson B., Sheridan R. M., Vousden W. A., Kaja A. L., Crouse G. D., Gifford J., Graupner P. R., Karr L. (2006). Engineering
of the spinosyn PKS: directing starter unit incorporation. J Nat Prod.

[2083] Xue C., Duan Y., Zhao F., Lu W. (2013). Stepwise increase of
spinosad production in Saccharopolyspora spinosa by metabolic engineering. Biochemical Engineering Journal.

[2084] Bridget A. F., Nguyen C. T., Magar R. T., Sohng J. K. (2023). Increasing
production of spinosad in Saccharopolyspora spinosa by metabolic engineering. Biotechnol Appl Biochem.

[2085] Tao H., Zhang Y., Deng Z., Liu T. (2019). Strategies for Enhancing
the Yield of the Potent Insecticide Spinosad in Actinomycetes. Biotechnol J.

[2086] Wang X., Zhang C., Wang M., Lu W. (2014). Genome-scale
metabolic network reconstruction of Saccharopolyspora spinosa for
spinosad production improvement. Microb Cell
Fact.

[2087] Proschak A., Zhou Q., Schoner T., Thanwisai A., Kresovic D., Dowling A., ffrench-Constant R., Proschak E., Bode H. B. (2014). Biosynthesis of the insecticidal
xenocyloins in Xenorhabdus bovienii. Chembiochem.

[2088] Meesil W., Muangpat P., Sitthisak S., Rattanarojpong T., Chantratita N., Machado R. A. R., Shi Y. M., Bode H. B., Vitta A., Thanwisai A. (2023). Genome mining
reveals novel biosynthetic gene clusters in entomopathogenic bacteria. Sci Rep.

[2089] Ganley J. G., Pandey A., Sylvester K., Lu K. Y., Toro-Moreno M., Rutschlin S., Bradford J. M., Champion C. J., Bottcher T., Xu J. (2020). A Systematic Analysis of Mosquito-Microbiome Biosynthetic Gene Clusters
Reveals Antimalarial Siderophores that Reduce Mosquito Reproduction
Capacity. Cell Chem Biol.

[2090] Shi Y. M., Hirschmann M., Shi Y. N., Ahmed S., Abebew D., Tobias N. J., Grun P., Crames J. J., Poschel L., Kuttenlochner W. (2022). Global analysis of biosynthetic
gene clusters reveals conserved and unique natural products in entomopathogenic
nematode-symbiotic bacteria. Nat Chem.

[2091] Takahashi E., Kimura T., Nakamura K., Arahira M., Iida M. (1995). Phosphonothrixin,
a novel herbicidal antibiotic produced by Saccharothrix
sp. ST-888. I. Taxonomy, fermentation, isolation and biological properties. J Antibiot (Tokyo).

[2092] Lin J., Nishiyama M., Kuzuyama T. (2015). Identification of the biosynthetic
gene cluster for the herbicide phosphonothrixin in Saccharothrix sp.
ST-888. J Antibiot (Tokyo).

[2093] Shi Y. M., Brachmann A. O., Westphalen M. A., Neubacher N., Tobias N. J., Bode H. B. (2019). Dual phenazine
gene
clusters enable diversification during biosynthesis. Nat Chem Biol.

[2094] Chin-A-Woeng T. F. C., Bloemberg G. V., Lugtenberg B. J. J. (2003). Phenazines
and their role in biocontrol by Pseudomonas bacteria. New Phytol.

[2095] He Y., Suzuki S., Aono T., Oyaizu H. (2004). Importance of 2,4-DAPG
in the biological control of brown patch by Pseudomonas fluorescens
HP72 and newly identified genes involved in 2,4-DAPG biosynthesis. Soil Science and Plant Nutrition.

[2096] Zhang Z., Zhang L., Zhang L., Chu H., Zhou J., Ju F. (2024). Diversity and distribution of biosynthetic
gene clusters in agricultural soil microbiomes. mSystems.

[2097] Santana-Pereira A. L. R., Sandoval-Powers M., Monsma S., Zhou J., Santos S. R., Mead D. A., Liles M. R. (2020). Discovery of Novel Biosynthetic Gene Cluster Diversity
From a Soil Metagenomic Library. Front Microbiol.

[2098] Dror, B. ; Wang, Z. ; Brady, S. F. ; Jurkevitch, E. ; Cytryn, E. Elucidating the Diversity and Potential Function of Nonribosomal Peptide and Polyketide Biosynthetic Gene Clusters in the Root Microbiome. mSystems 2020, 5 (6), 10.1128/mSystems.00866-20. PMC7762793 33361322

[2099] Blin K., Shaw S., Kloosterman A. M., Charlop-Powers Z., van Wezel G. P., Medema M. H., Weber T. (2021). antiSMASH
6.0: improving cluster detection and comparison capabilities. Nucleic Acids Res.

[2100] Medema M. H., Blin K., Cimermancic P., de Jager V., Zakrzewski P., Fischbach M. A., Weber T., Takano E., Breitling R. (2011). antiSMASH:
rapid identification, annotation and analysis of secondary metabolite
biosynthesis gene clusters in bacterial and fungal genome sequences. Nucleic Acids Res.

[2101] Blin K., Medema M. H., Kottmann R., Lee S. Y., Weber T. (2017). The antiSMASH database, a comprehensive
database of microbial secondary
metabolite biosynthetic gene clusters. Nucleic
Acids Res.

[2102] Blin K., Shaw S., Kautsar S. A., Medema M. H., Weber T. (2021). The antiSMASH
database version 3: increased taxonomic coverage and
new query features for modular enzymes. Nucleic
Acids Res.

[2103] Terlouw B. R., Blin K., Navarro-Munoz J. C., Avalon N. E., Chevrette M. G., Egbert S., Lee S., Meijer D., Recchia M. J. J., Reitz Z. L. (2023). MIBiG
3.0: a community-driven effort to annotate experimentally validated
biosynthetic gene clusters. Nucleic Acids Res.

[2104] van Santen J. A., Poynton E. F., Iskakova D., McMann E., Alsup T. A., Clark T. N., Fergusson C. H., Fewer D. P., Hughes A. H., McCadden C. A. (2022). The
Natural Products Atlas 2.0: a database of microbially-derived natural
products. Nucleic Acids Res.

[2105] Bibb M. J., Janssen G. R., Ward J. M. (1985). Cloning
and analysis
of the promoter region of the erythromycin resistance gene (ermE)
of Streptomyces erythraeus. Gene.

[2106] Labes G., Bibb M., Wohlleben W. (1997). Isolation
and characterization of a strong promoter element from the Streptomyces
ghanaensis phage I19 using the gentamicin resistance gene (aacC1)
of Tn 1696 as reporter. Microbiology (Reading).

[2107] Wang W., Li X., Wang J., Xiang S., Feng X., Yang K. (2013). An engineered
strong promoter for
streptomycetes. Appl Environ Microbiol.

[2108] Kang H. S., Charlop-Powers Z., Brady S. F. (2016). Multiplexed CRISPR/Cas9-
and TAR-Mediated Promoter Engineering of Natural Product Biosynthetic
Gene Clusters in Yeast. ACS Synth Biol.

[2109] Murakami T., Holt T. G., Thompson C. J. (1989). Thiostrepton-induced
gene expression in Streptomyces lividans. J
Bacteriol.

[2110] Rodriguez-Garcia A., Combes P., Perez-Redondo R., Smith M. C., Smith M. C. (2005). Natural and synthetic tetracycline-inducible
promoters for use in the antibiotic-producing bacteria Streptomyces. Nucleic Acids Res.

[2111] Horbal L., Fedorenko V., Luzhetskyy A. (2014). Novel and
tightly regulated resorcinol and cumate-inducible expression systems
for Streptomyces and other actinobacteria. Appl
Microbiol Biotechnol.

[2112] Horbal L., Luzhetskyy A. (2016). Dual control
system - A novel scaffolding
architecture of an inducible regulatory device for the precise regulation
of gene expression. Metab Eng.

[2113] Lussier F. X., Denis F., Shareck F. (2010). Adaptation
of the highly
productive T7 expression system to Streptomyces lividans. Appl Environ Microbiol.

[2114] Sidda J. D., Song L., Poon V., Al-Bassam M., Lazos O., Buttner M. J., Challis G. L., Corre C. (2014). Discovery
of a family of γ-aminobutyrate ureas via rational derepression
of a silent bacterial gene cluster. Chemical
Science.

[2115] O'Rourke S., Wietzorrek A., Fowler K., Corre C., Challis G. L., Chater K. F. (2009). Extracellular signalling, translational
control, two repressors and an activator all contribute to the regulation
of methylenomycin production in Streptomyces coelicolor. Molecular microbiology.

[2116] Bunet R., Song L., Mendes M. V., Corre C., Hotel L., Rouhier N., Framboisier X., Leblond P., Challis G. L., Aigle B. (2011). Characterization and
manipulation of the pathway-specific late regulator AlpW reveals Streptomyces
ambofaciens as a new producer of kinamycins. Journal of Bacteriology.

[2117] Yoo Y. J., Hwang J. Y., Shin H. L., Cui H., Lee J., Yoon Y. J. (2015). Characterization of negative regulatory
genes for the
biosynthesis of rapamycin in Streptomyces rapamycinicus and its application
for improved production. J Ind Microbiol Biotechnol.

[2118] Booth T. J., Kalaitzis J. A., Vuong D., Crombie A., Lacey E., Piggott A. M., Wilkinson B. (2020). Production
of novel pladienolide analogues through native expression of a pathway-specific
activator. Chem Sci.

[2119] Komatsu M., Uchiyama T., Omura S., Cane D. E., Ikeda H. (2010). Genome-minimized Streptomyces host
for the heterologous expression
of secondary metabolism. Proc Natl Acad Sci
U S A.

[2120] Liu W., Zhang Q., Guo J., Chen Z., Li J., Wen Y. (2015). Increasing Avermectin Production in Streptomyces avermitilis by Manipulating
the Expression of a Novel TetR-Family Regulator and Its Target Gene
Product. Appl Environ Microbiol.

[2121] Laureti L., Song L., Huang S., Corre C., Leblond P., Challis G. L., Aigle B. (2011). Identification of a
bioactive 51-membered macrolide complex by activation of a silent
polyketide synthase in Streptomyces ambofaciens. Proc Natl Acad Sci U S A.

[2122] Hur Y. A., Choi S. S., Sherman D. H., Kim E. S. (2008). Identification
of TmcN as a pathway-specific positive regulator of tautomycetin biosynthesis
in Streptomyces sp. CK4412. Microbiology (Reading).

[2123] Ingram C., Brawner M., Youngman P., Westpheling J. (1989). xylE functions
as an efficient reporter gene in Streptomyces spp.: use for the study
of galP1, a catabolite-controlled promoter. Journal of Bacteriology.

[2124] Guo F., Xiang S., Li L., Wang B., Rajasarkka J., Grondahl-Yli-Hannuksela K., Ai G., Metsa-Ketela M., Yang K. (2015). Targeted activation of silent natural
product biosynthesis pathways
by reporter-guided mutant selection. Metab Eng.

[2125] Nah H. J., Woo M. W., Choi S. S., Kim E. S. (2015). Precise
cloning and tandem integration of large polyketide biosynthetic gene
cluster using Streptomyces artificial chromosome system. Microb Cell Fact.

[2126] Li L., Zheng G., Chen J., Ge M., Jiang W., Lu Y. (2017). Multiplexed site-specific genome engineering for overproducing bioactive
secondary metabolites in actinomycetes. Metab
Eng.

[2127] Manderscheid N., Bilyk B., Busche T., Kalinowski J., Paululat T., Bechthold A., Petzke L., Luzhetskyy A. (2016). An influence
of the copy number of biosynthetic gene clusters on the production
level of antibiotics in a heterologous host. J Biotechnol.

[2128] Ke J., Robinson D., Wu Z. Y., Kuftin A., Louie K., Kosina S., Northen T., Cheng J. F., Yoshikuni Y. (2022). CRAGE-CRISPR
facilitates rapid activation of secondary metabolite biosynthetic
gene clusters in bacteria. Cell Chem Biol.

[2129] Ameruoso A., Villegas Kcam M. C., Cohen K. P., Chappell J. (2022). Activating
natural product synthesis using CRISPR interference and activation
systems in Streptomyces. Nucleic Acids Res.

[2130] Bloch, S. ; Temme, K. ; Tamsir, A. ; Higgins, D. ; Davis-Richardson, A. ; Clark, R. ; Gottlieb, S. Guided microbial remodeling, a platform for the rational improvement of microbial species for agriculture. HK40052404A, 2022.

[2131] Hmelo L.
R., Borlee B. R., Almblad H., Love M. E., Randall T. E., Tseng B. S., Lin C., Irie Y., Storek K. M., Yang J. J. (2015). Precision-engineering
the Pseudomonas aeruginosa genome with two-step allelic exchange. Nat Protoc.

[2132] Temme, K. ; Tamsir, A. Methods for multipart, modular and scarless assembly of dna molecules. WO2014011800A1, 2014.

[2133] Bloch S.
E., Ryu M. H., Ozaydin B., Broglie R. (2020). Harnessing
atmospheric nitrogen for cereal crop production. Curr Opin Biotechnol.

[2134] Wang X., Zhou H., Chen H., Jing X., Zheng W., Li R., Sun T., Liu J., Fu J., Huo L. (2018). Discovery of recombinases enables genome mining
of cryptic biosynthetic gene clusters in Burkholderiales species. Proc Natl Acad Sci U S A.

[2135] Tan G. Y., Deng K., Liu X., Tao H., Chang Y., Chen J., Chen K., Sheng Z., Deng Z., Liu T. (2017). Heterologous Biosynthesis of Spinosad:
An Omics-Guided Large Polyketide Synthase Gene Cluster Reconstitution
in Streptomyces. ACS Synth Biol.

[2136] Petrova Y. D., Zhao J., Webster G., Mullins A. J., Williams K., Alswat A. S., Challis G. L., Bailey A. M., Mahenthiralingam E. (2022). Cloning and expression of Burkholderia
polyyne biosynthetic
gene clusters in Paraburkholderia hosts provides a strategy for biopesticide
development. Microb Biotechnol.

[2137] Loeschcke A., Markert A., Wilhelm S., Wirtz A., Rosenau F., Jaeger K. E., Drepper T. (2013). TREX: a universal tool
for the transfer and expression of biosynthetic pathways in bacteria. ACS Synth Biol.

[2138] Gomez-Escribano J. P., Bibb M. J. (2011). Engineering Streptomyces
coelicolor
for heterologous expression of secondary metabolite gene clusters. Microb Biotechnol.

[2139] Myronovskyi M., Rosenkranzer B., Nadmid S., Pujic P., Normand P., Luzhetskyy A. (2018). Generation
of a cluster-free Streptomyces
albus chassis strains for improved heterologous expression of secondary
metabolite clusters. Metab Eng.

[2140] Li L., Maclntyre L. W., Brady S. F. (2021). Refactoring biosynthetic gene clusters
for heterologous production of microbial natural products. Curr Opin Biotechnol.

[2141] Kodumal S. J., Patel K. G., Reid R., Menzella H. G., Welch M., Santi D. V. (2004). Total synthesis of long DNA sequences:
synthesis of a contiguous 32-kb polyketide synthase gene cluster. Proc Natl Acad Sci U S A.

[2142] Chan L. Y., Kosuri S., Endy D. (2005). Refactoring
bacteriophage
T7. Mol Syst Biol.

[2143] Shao Z., Rao G., Li C., Abil Z., Luo Y., Zhao H. (2013). Refactoring
the silent spectinabilin gene cluster using
a plug-and-play scaffold. ACS Synth Biol.

[2144] Ren H., Biswas S., Ho S., Van Der Donk W. A., Zhao H. (2018). Rapid discovery of glycocins through pathway refactoring in Escherichia
coli. ACS chemical biology.

[2145] Menzella H. G., Reisinger S. J., Welch M., Kealey J. T., Kennedy J., Reid R., Tran C. Q., Santi D. V. (2006). Redesign,
synthesis and functional expression of the 6-deoxyerythronolide B
polyketide synthase gene cluster. J Ind Microbiol
Biotechnol.

[2146] Ghosh D., Kohli A. G., Moser F., Endy D., Belcher A. M. (2012). Refactored M13 bacteriophage as a platform for tumor
cell imaging and drug delivery. ACS Synth Biol.

[2147] Jaschke P. R., Lieberman E. K., Rodriguez J., Sierra A., Endy D. (2012). A fully decompressed
synthetic bacteriophage
oX174 genome assembled and archived in yeast. Virology.

[2148] Song C., Luan J., Cui Q., Duan Q., Li Z., Gao Y., Li R., Li A., Shen Y., Li Y. (2019). Enhanced Heterologous Spinosad Production from a 79-kb
Synthetic Multioperon Assembly. ACS Synth Biol.

[2149] Sánchez-Pascuala A., de Lorenzo V., Nikel P. I. (2017). Refactoring the Embden-Meyerhof-Parnas
pathway as a
whole of portable GlucoBricks for implantation of glycolytic modules
in Gram-negative bacteria. ACS synthetic biology.

[2150] Thompson M. G., Kirkpatrick L. D., Geiselman G. M., Waldburger L. M., Pearson A. N., Szarzanowicz M., Vuu K. M., Markel K., Hummel N. F., Suazo D. D. (2023). Genetically
refactored Agrobacterium-mediated transformation. bioRxiv.

[2151] Hillier S. G., Lathe R. (2019). Terpenes, hormones
and life: isoprene
rule revisited. J Endocrinol.

[2152] Inda M. E., Lu T. K. (2020). Microbes as Biosensors. Annu Rev Microbiol.

[2153] Wan X., Saltepe B., Yu L., Wang B. (2021). Programming living
sensors for environment, health and biomanufacturing. Microb Biotechnol.

[2154] Lazar, J. T. ; Tabor, J. J. Bacterial two-component systems as sensors for synthetic biology applications. Curr Opin Syst Biol 2021, 28, 100398. 10.1016/j.coisb.2021.100398. 34917859 PMC8670732

[2155] Salis H., Tamsir A., Voigt C. (2009). Engineering bacterial
signals and sensors. Contrib Microbiol.

[2156] Haskett T. L., Paramasivan P., Mendes M. D., Green P., Geddes B. A., Knights H. E., Jorrin B., Ryu M. H., Brett P., Voigt C. A. (2022). Engineered plant control
of associative nitrogen fixation. Proc Natl
Acad Sci U S A.

[2157] Skjoedt M. L., Snoek T., Kildegaard K. R., Arsovska D., Eichenberger M., Goedecke T. J., Rajkumar A. S., Zhang J., Kristensen M., Lehka B. J. (2016). Engineering
prokaryotic transcriptional activators as metabolite biosensors in
yeast. Nat Chem Biol.

[2158] Pini F., East A. K., Appia-Ayme C., Tomek J., Karunakaran R., Mendoza-Suarez M., Edwards A., Terpolilli J. J., Roworth J., Downie J. A. (2017). Bacterial Biosensors for in Vivo Spatiotemporal Mapping of Root Secretion. Plant Physiol.

[2159] Fernandez M., Morel B., Ramos J. L., Krell T. (2016). Paralogous
Regulators ArsR1 and ArsR2 of Pseudomonas putida KT2440 as a Basis
for Arsenic Biosensor Development. Appl Environ
Microbiol.

[2160] Gonzalez L. M., Mukhitov N., Voigt C. A. (2020). Resilient living
materials built by printing bacterial spores. Nat Chem Biol.

[2161] Del Valle I., Fulk E. M., Kalvapalle P., Silberg J. J., Masiello C. A., Stadler L. B. (2021). Translating New
Synthetic Biology Advances for Biosensing Into the Earth and Environmental
Sciences. Front Microbiol.

[2162] Xiong L. L., Garrett M. A., Buss M. T., Kornfield J. A., Shapiro M. G. (2022). Tunable Temperature-Sensitive Transcriptional Activation
Based on Lambda Repressor. ACS Synth Biol.

[2163] Fernandez-Rodriguez J., Moser F., Song M., Voigt C. A. (2017). Engineering
RGB color vision into Escherichia coli. Nat
Chem Biol.

[2164] Choi, J. ; Ahn, J. ; Bae, J. ; Koh, M. Recent Synthetic Biology Approaches for Temperature- and Light-Controlled Gene Expression in Bacterial Hosts. Molecules 2022, 27 (20), 6798. 10.3390/molecules27206798. 36296389 PMC9611254

[2165] Sen S., Apurva D., Satija R., Siegal D., Murray R. M. (2017). Design
of a Toolbox of RNA Thermometers. ACS Synth
Biol.

[2166] Levskaya A., Chevalier A. A., Tabor J. J., Simpson Z. B., Lavery L. A., Levy M., Davidson E. A., Scouras A., Ellington A. D., Marcotte E. M. (2005). Synthetic biology: engineering
Escherichia coli to see light. Nature.

[2167] Pham H. L., Wong A., Chua N., Teo W. S., Yew W. S., Chang M. W. (2017). Engineering a riboswitch-based genetic
platform for the self-directed evolution of acid-tolerant phenotypes. Nat Commun.

[2168] Brink K. R., Hunt M. G., Mu A. M., Groszman K., Hoang K. V., Lorch K. P., Pogostin B. H., Gunn J. S., Tabor J. J. (2023). An E. coli display method for characterization of peptide-sensor
kinase interactions. Nat Chem Biol.

[2169] Tran P., Prindle A. (2021). Synthetic biology in
biofilms: Tools,
challenges, and opportunities. Biotechnol Prog.

[2170] Moser F., Espah Borujeni A., Ghodasara A. N., Cameron E., Park Y., Voigt C. A. (2018). Dynamic control
of endogenous metabolism with combinatorial logic circuits. Mol Syst Biol.

[2171] Sengupta A., Pakrasi H. B., Wangikar P. P. (2018). Recent
advances
in synthetic biology of cyanobacteria. Appl
Microbiol Biotechnol.

[2172] Immethun C. M., Ng K. M., DeLorenzo D. M., Waldron-Feinstein B., Lee Y. C., Moon T. S. (2016). Oxygen-Responsive
Genetic Circuits Constructed in Synechocystis sp PCC 6803. Biotechnol Bioeng.

[2173] Kasey C. M., Zerrad M., Li Y., Cropp T. A., Williams G. J. (2018). Development of Transcription Factor-Based
Designer
Macrolide Biosensors for Metabolic Engineering and Synthetic Biology. ACS Synth Biol.

[2174] Wendisch V. F. (2014). Microbial production of amino acids
and derived chemicals:
synthetic biology approaches to strain development. Curr Opin Biotechnol.

[2175] Jang S., Jang S., Im D. K., Kang T. J., Oh M. K., Jung G. Y. (2019). Artificial Caprolactam-Specific Riboswitch
as an Intracellular Metabolite Sensor. ACS Synth
Biol.

[2176] Hicks M., Bachmann T. T., Wang B. (2020). Synthetic Biology Enables
Programmable Cell-Based Biosensors. Chemphyschem.

[2177] Gerstel U., Romling U. (2003). The csgD promoter,
a control unit
for biofilm formation in Salmonella typhimurium. Res Microbiol.

[2178] El-Samad H., Kurata H., Doyle J. C., Gross C. A., Khammash M. (2005). Surviving heat shock: control strategies
for robustness
and performance. Proc Natl Acad Sci U S A.

[2179] Rhodius V. A., Segall-Shapiro T. H., Sharon B. D., Ghodasara A., Orlova E., Tabakh H., Burkhardt D. H., Clancy K., Peterson T. C., Gross C. A. (2013). Design
of orthogonal genetic switches based on a crosstalk map of sigmas,
anti-sigmas, and promoters. Mol Syst Biol.

[2180] Hanko, E. K. R. ; Paiva, A. C. ; Jonczyk, M. ; Abbott, M. ; Minton, N. P. ; Malys, N. A genome-wide approach for identification and characterisation of metabolite-inducible systems. Nature Communications 2020, 11 (1), 10.1038/s41467-020-14941-6. PMC7057948 32139676

[2181] d'Oelsnitz S., Love J. D., Ellington A. D., Ross D. (2024). Ligify: Automated Genome Mining for Ligand-Inducible Transcription
Factors. ACS Synth Biol.

[2182] Xi C., Diao J., Moon T. S. (2023). Advances
in ligand-specific biosensing
for structurally similar molecules. Cell Syst.

[2183] d'Oelsnitz S., Diaz D. J., Kim W., Acosta D. J., Dangerfield T. L., Schechter M. W., Minus M. B., Howard J. R., Do H., Loy J. M. (2024). Biosensor and machine learning-aided engineering
of an amaryllidaceae enzyme. Nat Commun.

[2184] d'Oelsnitz S., Kim W., Burkholder N. T., Javanmardi K., Thyer R., Zhang Y., Alper H. S., Ellington A. D. (2022). Using fungible biosensors to evolve improved alkaloid
biosyntheses. Nat Chem Biol.

[2185] d'Oelsnitz S., Love J. D., Diaz D. J., Ellington A. D. (2022). GroovDB:
A Database of Ligand-Inducible Transcription Factors. ACS Synth Biol.

[2186] Laub M. T., Biondi E. G., Skerker J. M. (2007). Phosphotransfer
profiling: Systematic mapping of two-component signal transduction
pathways and phosphorelays. Method Enzymol.

[2187] Moser F., Broers N. J., Hartmans S., Tamsir A., Kerkman R., Roubos J. A., Bovenberg R., Voigt C. A. (2012). Genetic circuit performance under conditions relevant
for industrial bioreactors. ACS Synth Biol.

[2188] Del Vecchio D. (2015). Modularity,
context-dependence, and insulation in engineered
biological circuits. Trends Biotechnol.

[2189] Del Vecchio D., Dy A. J., Qian Y. (2016). Control theory meets
synthetic biology. Journal of The Royal Society
Interface.

[2190] Segall-Shapiro T. H., Meyer A. J., Ellington A. D., Sontag E. D., Voigt C. a. (2014). A 'resource allocator'
for transcription
based on a highly fragmented T7 RNA polymerase. Molecular Systems Biology.

[2191] Meng F., Ellis T. (2020). The second decade of
synthetic biology:
2010-2020. Nat Commun.

[2192] De Gelder L., Ponciano J. M., Joyce P., Top E. M. (2007). Stability
of a promiscuous plasmid in different hosts: no guarantee for a long-term
relationship. Microbiol-Sgm.

[2193] Meyer A. J., Segall-Shapiro T. H., Glassey E., Zhang J., Voigt C. A. (2019). Escherichia coli
“Marionette” strains
with 12 highly optimized small-molecule sensors. Nat Chem Biol.

[2194] San Millan, A. ; MacLean, R. C. Fitness costs of plasmids: a limit to plasmid transmission. Microbiology Spectrum 2017, 5 (5), mtbp-0016-2017 10.1128/microbiolspec.MTBP-0016-2017. PMC11687550 28944751

[2195] Keigwin, R. P. Reregistration eligibility decision for coppers. 2009.

[2196] Tamm, L. ; Thuerig, B. ; Apostolov, S. ; Blogg, H. ; Borgo, E. ; Corneo, P. E. ; Fittje, S. ; de Palma, M. ; Donko, A. ; Experton, C. ; Use of copper-based fungicides in organic agriculture in twelve European countries. Agronomy 2022, 12 (3), 673. 10.3390/agronomy12030673

[2197] Kang Y., Lee W., Kim S., Jang G., Kim B. G., Yoon Y. (2018). Enhancing the copper-sensing capability
of Escherichia coli-based whole-cell bioreporters by genetic engineering. Appl Microbiol Biotechnol.

[2198] Donley N. (2019). The USA lags behind other agricultural
nations in banning
harmful pesticides. Environ Health.

[2199] Chen J., Sun S., Li C. Z., Zhu Y. G., Rosen B. P. (2014). Biosensor for Organoarsenical Herbicides
and Growth
Promoters. Environ. Sci. Technol..

[2200] Yuan Y., Miao J. (2024). Agrochemical control of gene expression
using evolved split RNA polymerase. II. PeerJ.

[2201] Landry B. P., Palanki R., Dyulgyarov N., Hartsough L. A., Tabor J. J. (2018). Phosphatase activity tunes two-component
system sensor detection threshold. Nature Communications.

[2202] Schmidl S. R., Ekness F., Sofjan K., Daeffler K. N. M., Brink K. R., Landry B. P., Gerhardt K. P., Dyulgyarov N., Sheth R. U., Tabor J. J. (2019). Rewiring bacterial two-component
systems by modular DNA-binding domain swapping. Nat Chem Biol.

[2203] Menz, J. ; Range, T. ; Trini, J. ; Ludewig, U. ; Neuhauser, B. Molecular basis of differential nitrogen use efficiencies and nitrogen source preferences in contrasting Arabidopsis accessions. Scientific Reports 2018, 8, 10.1038/s41598-018-21684-4. PMC5820274 29463824

[2204] Ravikumar S., David Y., Park S. J., Choi J. I. (2018). A Chimeric
Two-Component Regulatory System-Based Escherichia coli Biosensor Engineered
to Detect Glutamate. Appl Biochem Biotech.

[2205] Binder, S. ; Schendzielorz, G. ; Stabler, N. ; Krumbach, K. ; Hoffmann, K. ; Bott, M. ; Eggeling, L. A high-throughput approach to identify genomic variants of bacterial metabolite producers at the single-cell level. Genome Biology 2012, 13 (5), 10.1186/gb-2012-13-5-r40. PMC3446293 22640862

[2206] Ellefson J. W., Ledbetter M. P., Ellington A. D. (2018). Directed
evolution of a synthetic phylogeny of programmable Trp repressors. Nat Chem Biol.

[2207] Mahr R., Gatgens C., Gatgens J., Polen T., Kalinowski J., Frunzke J. (2015). Biosensor-driven adaptive laboratory
evolution of L-valine production in Corynebacterium glutamicum. Metab. Eng..

[2208] Rothstein D. E. (2009). Soil amino-acid availability across a temperate-forest
fertility gradient. Biogeochemistry.

[2209] Crombez H., Motte H., Beeckman T. (2019). Tackling Plant
Phosphate
Starvation by the Roots. Dev Cell.

[2210] Santos-Beneit, F. The Pho regulon: a huge regulatory network in bacteria. Frontiers in Microbiology 2015, 6, ARTN 402. 10.3389/fmicb.2015.00402 PMC4415409 25983732

[2211] Dollard M.-A., Billard P. (2003). Whole-cell bacterial sensors for
the monitoring of phosphate bioavailability. Journal of Microbiological Methods.

[2212] Cardemil C. V., Smulski D. R., LaRossa R. A., Vollmer A. C. (2010). Bioluminescent
Escherichia coli Strains for the Quantitative Detection of Phosphate
and Ammonia in Coastal and Suburban Watersheds. DNA and Cell Biology.

[2213] Gillor O., Hadas O., Post A. F., Belkin S. (2010). Phosphorus
and nitrogen in a monomictic freshwater lake: employing cyanobacterial
bioreporters to gain new insights into nutrient bioavailability. Freshwater Biol.

[2214] Olmez T. T., Kehribar E. S., Isilak M. E., Lu T. K., Seker U. O. S. (2019). Synthetic Genetic Circuits for Self-Actuated
Cellular
Nanomaterial Fabrication Devices. ACS Synth.
Biol..

[2215] Morrissey J., Guerinot M. L. (2009). Iron Uptake and Transport in Plants:
The Good, the Bad, and the lonome. Chem Rev.

[2216] Choi, J. ; Ryu, S. Regulation of Iron Uptake by Fine-Tuning the Iron Responsiveness of the Iron Sensor Fur. Appl Environ Microbiol 2019, 85 (9), 10.1128/AEM.03026-18. PMC6495754 30824449

[2217] Brocklehurst K. R., Hobman J. L., Lawley B., Blank L., Marshall S. J., Brown N. L., Morby A. P. (1999). ZntR is
a Zn(II)-responsive
MerR-like transcriptional regulator of zntA in Escherichia coli. Molecular Microbiology.

[2218] Bondarenko O., Rolova T., Kahru A., Ivask A. (2008). Bioavailability
of Cd, Zn and Hg in Soil to Nine Recombinant Luminescent Metal Sensor
Bacteria. Sensors-Basel.

[2219] Hirooka K., Edahiro T., Kimura K., Fujita Y. (2012). Direct and
indirect regulation of the ycnKJI operon involved in copper uptake
through two transcriptional repressors, YcnK and CsoR, in Bacillus
subtilis. J Bacteriol.

[2220] Diaz-Mireles E., Wexler M., Todd J. D., Bellini D., Johnston A. W. B., Sawers R. G. (2005). The manganese-responsive
repressor
Mur of Rhizobium leguminosarum is a member of the Fur-superfamily
that recognizes an unusual operator sequence. Microbiology (Reading).

[2221] Chao T. C., Becker A., Buhrmester J., Puhler A., Weidner S. (2004). The Sinorhizobium meliloti fur gene
regulates, with dependence on Mn(II), transcription of the sitABCD
operon, encoding a metal-type transporter. J
Bacteriol.

[2222] Que Q., Helmann J. D. (2000). Manganese homeostasis
in Bacillus subtilis is regulated
by MntR, a bifunctional regulator related to the diphtheria toxin
repressor family of proteins. Mol Microbiol.

[2223] Balan A., Santacruz C. P., Moutran A., Ferreira R. C., Medrano F. J., Perez C. A., Ramos C. H., Ferreira L. C. (2006). The molybdate-binding
protein (ModA) of the plant pathogen Xanthomonas axonopodis pv. citri. Protein Expr Purif.

[2224] Delgado M. J., Tresierra-Ayala A., Talbi C., Bedmar E. J. (2006). Functional
characterization of the Bradyrhizobium japonicum modA and modB genes
involved in molybdenum transport. Microbiology
(Reading).

[2225] Rech S., Deppenmeier U., Gunsalus R. P. (1995). Regulation of the
molybdate transport operon, modABCD, of Escherichia coli in response
to molybdate availability. J Bacteriol.

[2226] Bolanos L., Brewin N. J., Bonilla I. (1996). Effects of Boron on
Rhizobium-Legume Cell-Surface Interactions and Nodule Development. Plant Physiol.

[2227] Papenfort K., Bassler B. L. (2016). Quorum sensing signal-response
systems
in Gram-negative bacteria. Nat Rev Microbiol.

[2228] Roessler M., Muller V. (2002). Chloride, a new environmental signal
molecule involved in gene regulation in a moderately halophilic bacterium,
Halobacillus halophilus. J Bacteriol.

[2229] Vetrivel, A. ; Ilango, S. ; Ariyamuthu, R. ; Devarajan, G. ; Nithya, T. Innovative Approaches in Microbial Bioremediation: Harnessing Synthetic Biology for Environmental Pollutants Removal. In Sustainable Environmental Remediation: Avenues in Nano and Biotechnology; Springer, 2025; pp 387-407.

[2230] Siegfried K., Endes C., Bhuiyan A. F. M. K., Kuppardt A., Mattusch J., van der
Meer J. R., Chatzinotas A., Harms H. (2012). Field Testing of Arsenic
in Groundwater
Samples of Bangladesh Using a Test Kit Based on Lyophilized Bioreporter
Bacteria. Environ. Sci. Technol..

[2231] Wan X., Volpetti F., Petrova E., French C., Maerkl S. J., Wang B. (2019). Cascaded amplifying circuits enable ultrasensitive cellular sensors
for toxic metals. Nat Chem Biol.

[2232] Fernandez M., Morel B., Ramos J. L., Krell T. (2016). Paralogous
Regulators ArsR1 and ArsR2 of Pseudomonas putida KT2440 as a Basis
for Arsenic Biosensor Development. Appl Environ
Microb.

[2233] Cerminati S., Soncini F. C., Checa S. K. (2015). A sensitive whole-cell
biosensor for the simultaneous detection of a broad-spectrum of toxic
heavy metal ions. Chem Commun.

[2234] Cayron J., Prudent E., Escoffier C., Gueguen E., Mandrand-Berthelot M.
A., Pignol D., Garcia D., Rodrigue A. (2017). Pushing the limits of nickel detection
to nanomolar range using a set of engineered bioluminescent Escherichia
coli. Environ Sci Pollut R.

[2235] Branco, R. ; Cristóvão, A. ; Morais, P. V. Highly Sensitive, Highly Specific Whole-Cell Bioreporters for the Detection of Chromate in Environmental Samples. PLoS ONE 2013, 8 (1), e54005 10.1371/journal.pone.0054005. 23326558 PMC3543429

[2236] Hurdebise Q., Tarayre C., Fischer C., Colinet G., Hiligsmann S., Delvigne F. (2015). Determination of Zinc, Cadmium and
Lead Bioavailability in Contaminated Soils at the Single-Cell Level
by a Combination of Whole-Cell Biosensors and Flow Cytometry. Sensors-Basel.

[2237] Bereza-Malcolm, L. ; Aracic, S. ; Franks, A. E. Development and Application of a Synthetically-Derived Lead Biosensor Construct for Use in Gram-Negative Bacteria. Sensors (Basel) 2016, 16 (12), 2174 10.3390/s16122174. 27999352 PMC5191153

[2238] He W., Hu Z.-H., Yuan S., Zhong W.-H., Mei Y.-Z., Dai C.-C. (2018). Bacterial Bioreporter-Based Mercury and Phenanthrene
Assessment in Yangtze River Delta Soils of China. Journal of Environmental Quality.

[2239] Ravikumar S., Ganesh I., Yoo I.-k., Hong S. H. (2012). Construction
of a bacterial biosensor for zinc and copper and its application to
the development of multifunctional heavy metal adsorption bacteria. Process Biochemistry.

[2240] Wei H., Ze-Ling S., Le-Le C., Wen-hui Z., Chuan-Chao D. (2014). Specific detection
of bioavailable phenanthrene and mercury by bacterium reporters in
the red soil. Int. J. Environ. Sci. Technol..

[2241] Gupta S., Saxena M., Saini N., Mahmooduzzafar, Kumar R., Kumar A. (2012). An Effective
Strategy for a Whole-Cell Biosensor Based on Putative Effector Interaction
Site of the Regulatory DmpR Protein. PLOS ONE.

[2242] Wise A. A., Kuske C. R. (2000). Generation of novel
bacterial regulatory
proteins that detect priority pollutant phenols. Appl Environ Microb.

[2243] Chong H., Ching C. B. (2016). Development of Colorimetric-Based
Whole-Cell Biosensor for Organophosphorus Compounds by Engineering
Transcription Regulator DmpR. ACS Synth. Biol..

[2244] Jha R. K., Kern T. L., Kim Y., Tesar C., Jedrzejczak R., Joachimiak A., Strauss C. E. (2016). A microbial sensor
for organophosphate hydrolysis exploiting an engineered specificity
switch in a transcription factor. Nucleic Acids
Res.

[2245] Keith L., Telliard W. (1979). ES&T Special Report: Priority
pollutants: I-a perspective view. Environ. Sci.
Technol..

[2246] Ray S., Panjikar S., Anand R. (2018). Design of Protein-Based Biosensors
for Selective Detection of Benzene Groups of Pollutants. Acs Sensors.

[2247] Sevilla E., Yuste L., Moreno R., Rojo F. (2017). Differential
expression of the three Alcanivorax borkumensis SK2 genes coding for
the P450 cytochromes involved in the assimilation of hydrocarbons. Environ Microbiol Rep.

[2248] Jaspers M. C., Meier C., Zehnder A. J., Harms H., van der Meer J. R. (2001). Measuring mass transfer processes
of octane with the
help of an alkSalkB::gfp-tagged Escherichia coli. Environ Microbiol.

[2249] Reed B., Blazeck J., Alper H. (2012). Evolution
of an alkane-inducible
biosensor for increased responsiveness to short-chain alkanes. J Biotechnol.

[2250] Kumari R., Tecon R., Beggah S., Rutler R., Arey J. S., van der Meer J. R. (2011). Development
of bioreporter assays
for the detection of bioavailability of long-chain alkanes based on
the marine bacterium Alcanivorax borkumensis strain SK2. Environ Microbiol.

[2251] Zhang D., He Y., Wang Y., Wang H., Wu L., Aries E., Huang W. E. (2012). Whole-cell
bacterial bioreporter
for actively searching and sensing of alkanes and oil spills. Microb Biotechnol.

[2252] Wu W., Zhang L., Yao L., Tan X., Liu X., Lu X. (2015). Genetically assembled fluorescent
biosensor for in situ detection
of bio-synthesized alkanes. Sci Rep.

[2253] Bahls M. O., Platz L., Morgado G., Schmidt G. W., Panke S. (2022). Directed evolution of biofuel-responsive
biosensors for automated
optimization of branched-chain alcohol biosynthesis. Metab Eng.

[2254] Ratajczak A., Geissdorfer W., Hillen W. (1998). Alkane hydroxylase
from Acinetobacter sp. strain ADP1 is encoded by alkM and belongs
to a new family of bacterial integral-membrane hydrocarbon hydroxylases. Appl Environ Microbiol.

[2255] Dietrich J. A., Shis D. L., Alikhani A., Keasling J. D. (2013). Transcription
Factor-Based Screens and Synthetic Selections for Microbial Small-Molecule
Biosynthesis. ACS Synth. Biol..

[2256] Cary T. J., Rylott E. L., Zhang L., Routsong R. M., Palazzo A. J., Strand S. E., Bruce N. C. (2021). Field trial
demonstrating
phytoremediation of the military explosive RDX by XplA/XplB-expressing
switchgrass. Nat Biotechnol.

[2257] Garmendia J., de las Heras A., Galvao T. C., de Lorenzo V. (2008). Tracing explosives
in soil with transcriptional regulators of Pseudomonas putida evolved
for responding to nitrotoluenes. Microb Biotechnol.

[2258] Behzadian F., Barjeste H., Hosseinkhani S., Zarei A. R. (2011). Construction and
characterization of Escherichia coli
whole-cell biosensors for toluene and related compounds. Curr Microbiol.

[2259] Davidson M. E., Harbaugh S. V., Chushak Y. G., Stone M. O., Kelley-Loughnane N. (2013). Development of a 2,4-dinitrotoluene-responsive
synthetic
riboswitch in E. coli cells. Acs Chem Biol.

[2260] Espah Borujeni A., Mishler D. M., Wang J., Huso W., Salis H. M. (2016). Automated
physics-based design of synthetic riboswitches
from diverse RNA aptamers. Nucleic Acids Res.

[2261] Lewis T. A., Newcombe D. A., Crawford R. L. (2004). Bioremediation of
soils contaminated with explosives. J Environ
Manage.

[2262] Yagur-Kroll S., Lalush C., Rosen R., Bachar N., Moskovitz Y., Belkin S. (2014). Escherichia coli bioreporters for
the detection of 2,4-dinitrotoluene and 2,4,6-trinitrotoluene. Appl Microbiol Biotechnol.

[2263] Yagur-Kroll S., Amiel E., Rosen R., Belkin S. (2015). Detection
of 2,4-dinitrotoluene and 2,4,6-trinitrotoluene by an Escherichia
coli bioreporter: performance enhancement by directed evolution. Appl Microbiol Biotechnol.

[2264] Belkin S., Yagur-Kroll S., Kabessa Y., Korouma V., Septon T., Anati Y., Zohar-Perez C., Rabinovitz Z., Nussinovitch A., Agranat A. J. (2017). Remote detection
of buried landmines using a bacterial sensor. Nature biotechnology.

[2265] Essington E. A., Vezeau G. E., Cetnar D. P., Grandinette E., Bell T. H., Salis H. M. (2024). An autonomous microbial
sensor enables
long-term detection of TNT explosive in natural soil. Nat Commun.

[2266] Pini F., East A. K., Appia-Ayme C., Tomek J., Karunakaran R., Mendoza-Suárez M., Edwards A., Terpolilli J. J., Roworth J., Downie J. A. (2017). Bacterial Biosensors for in Vivo Spatiotemporal Mapping of Root Secretion. Plant Physiology.

[2267] Mattiuzzo M., Bertani I., Ferluga S., Cabrio L., Bigirimana J., Guarnaccia C., Pongor S., Maraite H., Venturi V. (2011). The plant pathogen
Pseudomonas fuscovaginae contains
two conserved quorum sensing systems involved in virulence and negatively
regulated by RsaL and the novel regulator RsaM. Environmental Microbiology.

[2268] Scott S. R., Hasty J. (2016). Quorum Sensing Communication
Modules
for Microbial Consortia. ACS Synth Biol.

[2269] Wu Y., Wang C. W., Wang D., Wei N. (2021). A Whole-Cell Biosensor
for Point-of-Care Detection of Waterborne Bacterial Pathogens. ACS Synth. Biol..

[2270] Cheng Y. Y., Chen Z., Cao X., Ross T. D., Falbel T. G., Burton B. M., Venturelli O. S. (2023). Programming
bacteria for multiplexed DNA detection. Nat
Commun.

[2271] Cooper R. M., Wright J. A., Ng J. Q., Goyne J. M., Suzuki N., Lee Y. K., Ichinose M., Radford G., Ryan F. J., Kumar S. (2023). Engineered bacteria
detect tumor DNA. Science.

[2272] Wei H. L., Zhang L. Q. (2006). Quorum-sensing system
influences
root colonization and biological control ability in Pseudomonas fluorescens
2P24. Antonie Van Leeuwenhoek.

[2273] Xiong Q., Liu D., Zhang H., Dong X., Zhang G., Liu Y., Zhang R. (2020). Quorum sensing
signal
autoinducer-2 promotes root colonization of Bacillus velezensis SQR9
by affecting biofilm formation and motility. Appl Microbiol Biotechnol.

[2274] Cai W., Ou F., Staehelin C., Dai W. (2020). Bradyrhizobium sp.
strain ORS278 promotes rice growth and its quorum sensing system is
required for optimal root colonization. Environ
Microbiol Rep.

[2275] Koutsoudis M. D., Tsaltas D., Minogue T. D., von Bodman S. B. (2006). Quorum-sensing
regulation governs bacterial adhesion, biofilm development, and host
colonization in Pantoea stewartii subspecies stewartii. Proc Natl Acad Sci U S A.

[2276] Mensi I., Daugrois J. H., Pieretti I., Gargani D., Fleites L. A., Noell J., Bonnot F., Gabriel D. W., Rott P. (2016). Surface polysaccharides and quorum
sensing are involved in the attachment
and survival of Xanthomonas albilineans on sugarcane leaves. Mol Plant Pathol.

[2277] Perez-Velazquez J., Schlicht R., Dulla G., Hense B. A., Kuttler C., Lindow S. E. (2012). Stochastic modeling
of Pseudomonas
syringae growth in the phyllosphere. Math Biosci.

[2278] Choudhary S., Schmidt-Dannert C. (2010). Applications of quorum sensing in
biotechnology. Appl Microbiol Biotechnol.

[2279] Helman Y., Chernin L. (2015). Silencing the mob: disrupting quorum
sensing as a means to fight plant disease. Mol
Plant Pathol.

[2280] Dessaux, Y. ; Faure, D. Quorum Sensing and Quorum Quenching in Agrobacterium: A “Go/No Go System”?. Genes (Basel) 2018, 9 (4), 210. 10.3390/genes9040210 29659511 PMC5924552

[2281] van
Gestel J., Bareia T., Tenennbaum B., Dal Co A., Guler P., Aframian N., Puyesky S., Grinberg I., D'Souza G. G., Erez Z. (2021). Short-range
quorum sensing controls horizontal gene transfer at micron scale in
bacterial communities. Nat Commun.

[2282] DeAngelis K. M., Lindow S. E., Firestone M. K. (2008). Bacterial
quorum sensing and nitrogen cycling in rhizosphere soil. FEMS Microbiol Ecol.

[2283] Cha C., Gao P., Chen Y. C., Shaw P. D., Farrand S. K. (1998). Production
of acyl-homoserine lactone quorum-sensing signals by gram-negative
plant-associated bacteria. Mol Plant Microbe
Interact.

[2284] Yan H., Liu C., Yu W., Zhu X., Chen B. (2023). The aggregate
distribution of Pseudomonas aeruginosa on biochar facilitates quorum
sensing and biofilm formation. Sci Total Environ.

[2285] You L., Cox R. S., Weiss R., Arnold F. H. (2004). Programmed
population control by cell-cell communication and regulated killing. Nature.

[2286] Balagadde F. K., You L., Hansen C. L., Arnold F. H., Quake S. R. (2005). Long-term monitoring of bacteria
undergoing programmed
population control in a microchemostat. Science.

[2287] Dinh C. V., Prather K. L. J. (2019). Development of an autonomous and
bifunctional quorum-sensing circuit for metabolic flux control in
engineered Escherichia coli. Proc Natl Acad
Sci U S A.

[2288] Horswill A. R., Stoodley P., Stewart P. S., Parsek M. R. (2007). The effect
of the chemical, biological, and physical environment on quorum sensing
in structured microbial communities. Anal Bioanal
Chem.

[2289] Zuniga A., Fuente F., Federici F., Lionne C., Bonnet J., de Lorenzo V., Gonzalez B. (2018). An Engineered Device
for Indoleacetic Acid Production under Quorum Sensing Signals Enables
Cupriavidus pinatubonensis JMP134 To Stimulate Plant Growth. ACS Synth Biol.

[2290] Bareia T., Pollak S., Eldar A. (2018). Self-sensing
in Bacillus
subtilis quorum-sensing systems. Nat Microbiol.

[2291] Cui S., Lv X., Wu Y., Li J., Du G., Ledesma-Amaro R., Liu L. (2019). Engineering a Bifunctional Phr60-Rap60-Spo0A
Quorum-Sensing Molecular Switch for Dynamic Fine-Tuning of Menaquinone-7
Synthesis in Bacillus subtilis. ACS Synth Biol.

[2292] Vaiana C. A., Kim H., Cottet J., Oai K., Ge Z., Conforti K., King A. M., Meyer A. J., Chen H., Voigt C. A. (2022). Characterizing chemical signaling between engineered
“microbial sentinels” in porous microplates. Mol Syst Biol.

[2293] Du P., Zhao H., Zhang H., Wang R., Huang J., Tian Y., Luo X., Luo X., Wang M., Xiang Y. (2020). De novo design of an
intercellular signaling toolbox
for multi-channel cell-cell communication and biological computation. Nat Commun.

[2294] Scott S. R., Din M. O., Bittihn P., Xiong L., Tsimring L. S., Hasty J. (2017). A stabilized microbial ecosystem
of self-limiting bacteria using synthetic quorum-regulated lysis. Nat Microbiol.

[2295] Kylilis, N. ; Tuza, Z. A. ; Stan, G. B. ; Polizzi, K. M. Tools for engineering coordinated system behaviour in synthetic microbial consortia. Nature Communications 2018, 9, ARTN 2677. 10.1038/s41467-018-05046-2 PMC6041260 29992956

[2296] Scott S. R., Hasty J. (2016). Quorum Sensing Communication Modules
for Microbial Consortia. ACS Synth. Biol..

[2297] Gao X., Cheng H. Y., Del Valle I., Liu S., Masiello C. A., Silberg J. J. (2016). Charcoal Disrupts Soil Microbial Communication through
a Combination of Signal Sorption and Hydrolysis. ACS Omega.

[2298] Masiello C. A., Chen Y., Gao X., Liu S., Cheng H. Y., Bennett M. R., Rudgers J. A., Wagner D. S., Zygourakis K., Silberg J. J. (2013). Biochar and microbial signaling:
production conditions determine effects on microbial communication. Environ Sci Technol.

[2299] Sheng H., Wang F., Gu C., Stedtfeld R., Bian Y., Liu G., Wu W., Jiang X. (2018). Sorption characteristics
of N-acyl homserine lactones as signal molecules in natural soils
based on the analysis of kinetics and isotherms. RSC Adv.

[2300] Miller M. B., Bassler B. L. (2001). Quorum sensing in bacteria. Annu
Rev Microbiol.

[2301] Jenul, C. ; Horswill, A. R. Regulation of Staphylococcus aureus Virulence. Microbiol Spectr 2019, 7 (2), 10.1128/microbiolspec.GPP3-0031-2018. PMC6452892 30953424

[2302] Marchand N., Collins C. H. (2016). Synthetic Quorum
Sensing and Cell-Cell
Communication in Gram-Positive Bacillus megaterium. ACS Synth Biol.

[2303] Marchand N., Collins C. H. (2013). Peptide-based communication
system
enables Escherichia coli to Bacillus megaterium interspecies signaling. Biotechnol Bioeng.

[2304] Bais H. P., Weir T. L., Perry L. G., Gilroy S., Vivanco J. M. (2006). The role of root exudates in rhizosphere
interactions
with plants and other organisms. Annu Rev Plant
Biol.

[2305] Carvalhais L. C., Dennis P. G., Fedoseyenko D., Hajirezaei M. R., Borriss R., Von Wirén N. (2011). Root exudation
of sugars, amino acids, and organic acids by maize as affected by
nitrogen, phosphorus, potassium, and iron deficiency. Journal of Plant Nutrition and Soil Science.

[2306] Harbort C. J., Hashimoto M., Inoue H., Niu Y. L., Guan R., Rombola A. D., Kopriva S., Voges M. J. E. E. E., Sattely E. S., Garrido-Oter R. (2020). Root-Secreted Coumarins
and the Microbiota Interact to Improve Iron Nutrition in Arabidopsis. Cell Host Microbe.

[2307] Miller W. G., Brandl M. T., Quinones B., Lindow S. E. (2001). Biological
sensor for sucrose availability: Relative sensitivities of various
reporter genes. Appl Environ Microb.

[2308] Leveau J. H., Lindow S. E. (2001). Appetite of an epiphyte: quantitative
monitoring of bacterial sugar consumption in the phyllosphere. Proc Natl Acad Sci U S A.

[2309] Jaeger C. H., Lindow S. E., Miller W., Clark E., Firestone M. K. (1999). Mapping
of sugar and amino acid availability
in soil around roots with bacterial sensors of sucrose and tryptophan. Appl Environ Microbiol.

[2310] Bringhurst R. M., Cardon Z. G., Gage D. J. (2001). Galactosides
in
the rhizosphere: Utilization by sinorhizobium meliloti and development
of a biosensor. P Natl Acad Sci USA.

[2311] Tonti-Filippini J., Nevill P. G., Dixon K., Small I. (2017). What can we
do with 1000 plastid genomes?. Plant J.

[2312] Horbal L., Fedorenko V., Luzhetskyy A. (2014). Novel and
tightly regulated resorcinol and cumate-inducible expression systems
for Streptomyces and other actinobacteria. Appl
Microbiol Biot.

[2313] de los Santos E. L., Meyerowitz J. T., Mayo S. L., Murray R. M. (2016). Engineering
Transcriptional Regulator Effector Specificity Using Computational
Design and In Vitro Rapid Prototyping: Developing a Vanillin Sensor. ACS Synth Biol.

[2314] Siedler S., Khatri N. K., Zsohar A., Kjaerbolling I., Vog M., Hammar P., Nielsen C. F., Marienhagen J., Sommer M. O. A., Joensson H. N. (2017). Development of a
Bacterial Biosensor
for Rapid Screening of Yeast p-Coumaric Acid Production. ACS Synth. Biol..

[2315] Jiang T., Li C. Y., Yan Y. J. (2021). Optimization
of
a p-Coumaric Acid Biosensor System for Versatile Dynamic Performance. ACS Synth. Biol..

[2316] Machado, L. F. M. ; Currin, A. ; Dixon, N. Directed evolution of the PcaV allosteric transcription factor to generate a biosensor for aromatic aldehydes. Journal of Biological Engineering 2019, 13 (1), ARTN 91. 10.1186/s13036-019-0214-z PMC6882365 31798685

[2317] Ganesh I., Maruthamuthu M. K., Hong S. H. (2016). Engineering a chimeric
malate two-component system by introducing a positive feedback loop
in Escherichia coli. Korean J Chem Eng.

[2318] Ganesh I., Ravikumar S., Lee S. H., Park S. J., Hong S. H. (2013). Engineered
fumarate sensing Escherichia coli based
on novel chimeric two-component system. Journal
of Biotechnology.

[2319] Thompson M. G., Costello Z., Hummel N. F. C., Cruz-Morales P., Blake-Hedges J. M., Krishna R. N., Skyrud W., Pearson A. N., Incha M. R., Shih P. M. (2019). Robust Characterization
of Two Distinct Glutarate Sensing Transcription Factors of Pseudomonas
putida L-Lysine Metabolism. ACS Synth. Biol..

[2320] Dimas R. P., Jiang X. L., de la Paz J. A., Morcos F., Chan C. T. Y. (2019). Engineering
repressors with coevolutionary
cues facilitates toggle switches with a master reset. Nucleic Acids Research.

[2321] Taylor N. D., Garruss A. S., Moretti R., Chan S., Arbing M. A., Cascio D., Rogers J. K., Isaacs F. J., Kosuri S., Baker D. (2016). Engineering
an allosteric
transcription factor to respond to new ligands. Nat Methods.

[2322] Rogers J. K., Guzman C. D., Taylor N. D., Raman S., Anderson K., Church G. M. (2015). Synthetic biosensors for precise
gene control and real-time monitoring of metabolites. Nucleic Acids Research.

[2323] Siedler S., Stahlhut S. G., Malla S., Maury J., Neves A. R. (2014). Novel biosensors based on flavonoid-responsive
transcriptional
regulators introduced into Escherichia coli. Metab. Eng..

[2324] Sun H. H., Zhao H. M., Ang E. L. (2020). A New Biosensor
for Stilbenes and a Cannabinoid Enabled by Genome Mining of a Transcriptional
Regulator. ACS Synth. Biol..

[2325] Ding P. T., Ding Y. L. (2020). Stories of Salicylic
Acid: A Plant
Defense Hormone. Trends in Plant Science.

[2326] Bloch S. E., Clark R., Gottlieb S. S., Wood L. K., Shah N., Mak S.-M., Lorigan J. G., Johnson J., Davis-Richardson A. G., Williams L. (2020). Biological nitrogen
fixation in maize: optimizing nitrogenase expression in a root-associated
diazotroph. Journal of Experimental Botany.

[2327] McGenity T. J., Crombie A. T., Murrell J. C. (2018). Microbial
cycling
of isoprene, the most abundantly produced biological volatile organic
compound on Earth. Isme J.

[2328] Fini, A. ; Brunetti, C. ; Loreto, F. ; Centritto, M. ; Ferrini, F. ; Tattini, M. Isoprene Responses and Functions in Plants Challenged by Environmental Pressures Associated to Climate Change. Frontiers in Plant Science 2017, 8, ARTN 1281. 10.3389/fpls.2017.01281 PMC5526906 28798754

[2329] Kim S. K., Kim S. H., Subhadra B., Woo S. G., Rha E., Kim S. W., Kim H., Lee D. H., Lee S. G. (2018). A Genetically
Encoded Biosensor for Monitoring Isoprene Production in Engineered
Escherichia coli. ACS Synth. Biol..

[2330] McClune C. J., Alvarez-Buylla A., Voigt C. A., Laub M. T. (2019). Engineering
orthogonal signalling pathways reveals the sparse occupancy of sequence
space. Nature.

[2331] Abdel-Ghany S. E., Day I., Heuberger A. L., Broeckling C. D., Reddy A. S. (2016). Production of Phloroglucinol, a Platform
Chemical, in Arabidopsis using a Bacterial Gene. Sci Rep.

[2332] Mondy S., Lenglet A., Beury-Cirou A., Libanga C., Ratet P., Faure D., Dessaux Y. (2014). An increasing
opine carbon bias in artificial exudation systems and genetically
modified plant rhizospheres leads to an increasing reshaping of bacterial
populations. Mol Ecol.

[2333] Savka M. A., Farrand S. K. (1997). Modification of
rhizobacterial populations
by engineering bacterium utilization of a novel plant-produced resource. Nat Biotechnol.

[2334] Levin I., Liu M., Voigt C. A., Coley C. W. (2022). Merging
enzymatic and synthetic chemistry with computational synthesis planning. Nat Commun.

[2335] Saltepe B., Kehribar E. S., Su Yirmibesoglu S. S., Safak Seker U. O. (2018). Cellular Biosensors with Engineered Genetic Circuits. ACS Sens.

[2336] Karig D., Weiss R. (2005). Signal-amplifying genetic circuit
enables in vivo observation of weak promoter activation in the Rhl
quorum sensing system. Biotechnol Bioeng.

[2337] Gao C., Xu P., Ye C., Chen X., Liu L. (2019). Genetic Circuit-Assisted
Smart Microbial Engineering. Trends Microbiol.

[2338] Tan S. I., Ng I. S. (2020). New Insight into Plasmid-Driven T7
RNA Polymerase in Escherichia coli and Use as a Genetic Amplifier
for a Biosensor. ACS Synth. Biol..

[2339] Wang B., Barahona M., Buck M. (2014). Engineering
modular
and tunable genetic amplifiers for scaling transcriptional signals
in cascaded gene networks. Nucleic Acids Res.

[2340] Yokobayashi Y., Weiss R., Arnold F. H. (2002). Directed evolution
of a genetic circuit. Proc Natl Acad Sci U S
A.

[2341] Knight T. F., Sussman G. J. (1998). Cellular gate technology. Spr S Disc Math.

[2342] Entus R., Aufderheide B., Sauro H. M. (2007). Design and implementation
of three incoherent feed-forward motif based biological concentration
sensors. Syst Synth Biol.

[2343] Bandyopadhyay S., Pavlika V., Bracewell D. G., Nesbeth D. N. (2023). A Biological OR(XNOR) Logic Gate Couples Carbon Source
and Transgene Expression Switching in a Komagataella phaffii (Pichia
pastoris) Strain Co-producing Process-Enhancing Lipase and a Virus-like
Particle (VLP) Vaccine. ACS Synth Biol.

[2344] Moon T. S., Lou C., Tamsir A., Stanton B. C., Voigt C. A. (2012). Genetic programs
constructed from layered logic gates
in single cells. Nature.

[2345] Vaidyanathan P., Der B. S., Bhatia S., Roehner N., Silva R., Voigt C. A., Densmore D. (2015). A Framework
for Genetic
Logic Synthesis. P Ieee.

[2346] Xiang Y., Dalchau N., Wang B. (2018). Scaling up
genetic
circuit design for cellular computing: advances and prospects. Nat Comput.

[2347] Wang B., Buck M. (2012). Customizing cell signaling using
engineered genetic logic circuits. Trends Microbiol.

[2348] Wang B., Kitney R. I., Joly N., Buck M. (2011). Engineering
modular and orthogonal genetic logic gates for robust digital-like
synthetic biology. Nat Commun.

[2349] Anderson J. C., Voigt C. A., Arkin A. P. (2007). Environmental
signal
integration by a modular AND gate. Mol Syst
Biol.

[2350] Wang B., Barahona M., Buck M. (2013). A modular
cell-based
biosensor using engineered genetic logic circuits to detect and integrate
multiple environmental signals. Biosens Bioelectron.

[2351] Li X., Daniel R. (2022). Synthetic nonlinear computation for genetic circuit
design. Curr Opin Biotechnol.

[2352] Roquet N., Lu T. K. (2014). Digital and analog gene circuits
for biotechnology. Biotechnol J.

[2353] Purcell O., Lu T. K. (2014). Synthetic analog
and digital circuits
for cellular computation and memory. Curr Opin
Biotechnol.

[2354] Basu S., Mehreja R., Thiberge S., Chen M. T., Weiss R. (2004). Spatiotemporal
control of gene expression with pulse-generating networks. Proc Natl Acad Sci U S A.

[2355] Danino T., Mondragon-Palomino O., Tsimring L., Hasty J. (2010). A synchronized
quorum of genetic clocks. Nature.

[2356] Potvin-Trottier L., Lord N. D., Vinnicombe G., Paulsson J. (2016). Synchronous long-term
oscillations in a synthetic gene
circuit. Nature.

[2357] Frei T., Khammash M. (2021). Adaptive circuits in
synthetic biology. Current Opinion in Systems
Biology.

[2358] Bartoli V., di Bernardo M., Gorochowski T. E. (2020). Self-adaptive
biosystems through tunable genetic parts and circuits. Current Opinion in Systems Biology.

[2359] Steel, H. ; Lillacci, G. ; Khammash, M. ; Papachristodoulou, A. Challenges at the interface of control engineering and synthetic biology. In 2017 IEEE 56th Annual Conference on Decision and Control (CDC), 12-15 Dec. 2017, 2017; pp 1014-1023 10.1109/CDC.2017.8263791.

[2360] Yang L., Nielsen A. A., Fernandez-Rodriguez J., McClune C. J., Laub M. T., Lu T. K., Voigt C. A. (2014). Permanent
genetic memory with >1-byte capacity. Nat
Methods.

[2361] Siuti P., Yazbek J., Lu T. K. (2013). Synthetic circuits
integrating logic and memory in living cells. Nat Biotechnol.

[2362] Yehl K., Lu T. (2017). Scaling Computation and Memory in
Living Cells. Curr Opin Biomed Eng.

[2363] Bonnet J., Subsoontorn P., Endy D. (2012). Rewritable digital
data storage in live cells via engineered control of recombination
directionality. P Natl Acad Sci USA.

[2364] Padmakumar J. P., Sun J. J., Cho W., Zhou Y., Krenz C., Han W. Z., Densmore D., Sontag E. D., Voigt C. A. (2025). Partitioning
of a 2-bit hash function across 66 communicating
cells. Nat Chem Biol.

[2365] Shin J., Zhang S., Der B. S., Nielsen A. A., Voigt C. A. (2020). Programming Escherichia coli to function as a digital
display. Molecular systems biology.

[2366] Sexton J. T., Tabor J. J. (2020). Multiplexing cell-cell
communication. Mol Syst Biol.

[2367] Shin J., Zhang S., Der B. S., Nielsen A. A., Voigt C. A. (2020). Programming Escherichia coli to function
as a digital
display. Mol Syst Biol.

[2368] Sarkar K., Chakraborty S., Bonnerjee D., Bagh S. (2021). Distributed Computing with Engineered
Bacteria and Its Application
in Solving Chemically Generated 2 x 2 Maze Problems. ACS Synth Biol.

[2369] Al-Radhawi M. A., Tran A. P., Ernst E. A., Chen T., Voigt C. A., Sontag E. D. (2020). Distributed Implementation
of Boolean
Functions by Transcriptional Synthetic Circuits. ACS Synth Biol.

[2370] Chen T., Ali Al-Radhawi M., Voigt C. A., Sontag E. D. (2021). A synthetic
distributed genetic multi-bit counter. iScience.

[2371] Subsoontorn P., Endy D. (2012). Design and Analysis
of Genetically
Encoded Counters. Procedia Computer Science.

[2372] Clancy K., Voigt C. A. (2010). Programming cells:
towards an automated
'Genetic Compiler'. Curr Opin Biotechnol.

[2373] Dasika M. S., Maranas C. D. (2008). OptCircuit: an optimization based
method for computational design of genetic circuits. BMC Syst Biol.

[2374] Hooshangi S., Thiberge S., Weiss R. (2005). Ultrasensitivity
and
noise propagation in a synthetic transcriptional cascade. Proc Natl Acad Sci U S A.

[2375] Sprinzak D., Elowitz M. B. (2005). Reconstruction of
genetic circuits. Nature.

[2376] Thattai M., van Oudenaarden A. (2002). Attenuation
of noise in ultrasensitive
signaling cascades. Biophys J.

[2377] Castillo-Hair S. M., Fujita M., Igoshin O. A., Tabor J. J. (2019). An Engineered
B. subtilis Inducible Promoter System with over 10 000-Fold Dynamic
Range. ACS Synth Biol.

[2378] Shen-Orr S. S., Milo R., Mangan S., Alon U. (2002). Network motifs
in the transcriptional regulation network of Escherichia coli. Nat Genet.

[2379] Milo R., Shen-Orr S., Itzkovitz S., Kashtan N., Chklovskii D., Alon U. (2002). Network motifs: simple
building blocks of complex networks. Science.

[2380] Mangan S., Zaslaver A., Alon U. (2003). The coherent feedforward
loop serves as a sign-sensitive delay element in transcription networks. J Mol Biol.

[2381] Xiong K., Lancaster A. K., Siegal M. L., Masel J. (2019). Feed-forward
regulation adaptively evolves via dynamics rather than topology when
there is intrinsic noise. Nat Commun.

[2382] Alon U. (2007). Network motifs: theory and experimental
approaches. Nat Rev Genet.

[2383] Jones R. D., Qian Y., Siciliano V., DiAndreth B., Huh J., Weiss R., Del Vecchio D. (2020). An endoribonuclease-based
feedforward controller for decoupling resource-limited genetic modules
in mammalian cells. Nat Commun.

[2384] Goentoro L., Shoval O., Kirschner M. W., Alon U. (2009). The incoherent feedforward loop can provide fold-change detection
in gene regulation. Mol Cell.

[2385] Frei, T. ; Khammash, M. Adaptive circuits in synthetic biology. Curr Opin Syst Biol 2021, 28, 100399. 10.1016/j.coisb.2021.100399

[2386] Barajas C., Huang H. H., Gibson J., Sandoval L., Del Vecchio D. (2022). Feedforward growth rate control mitigates
gene activation
burden. Nat Commun.

[2387] Barone F., Dorr F., Marasco L. E., Mildiner S., Patop I. L., Sosa S., Vattino L. G., Vignale F. A., Altszyler E., Basanta B. (2017). Design
and evaluation
of an incoherent feed-forward loop for an arsenic biosensor based
on standard iGEM parts. Synth Biol (Oxf).

[2388] Pieters P. A., Nathalia B. L., van der
Linden A. J., Yin P., Kim J., Huck W. T. S., de Greef T. F. A. (2021). Cell-Free Characterization
of Coherent Feed-Forward Loop-Based Synthetic Genetic Circuits. ACS Synth Biol.

[2389] Alon, U. An Introduction to Systems Biology: Design Principles of Biological Circuits; Taylor & Francis, 2006.

[2390] Mitrophanov A. Y., Groisman E. A. (2008). Positive feedback
in cellular control
systems. Bioessays.

[2391] Becskei A., Seraphin B., Serrano L. (2001). Positive feedback
in
eukaryotic gene networks: cell differentiation by graded to binary
response conversion. EMBO J.

[2392] Higo A., Isu A., Fukaya Y., Hisabori T. (2017). Designing
Synthetic Flexible Gene Regulation Networks Using RNA Devices in Cyanobacteria. ACS Synth. Biol..

[2393] Marciano D. C., Lua R. C., Katsonis P., Amin S. R., Herman C., Lichtarge O. (2014). Negative feedback
in genetic circuits
confers evolutionary resilience and capacitance. Cell Rep.

[2394] Simpson M. L., Cox C. D., Sayler G. S. (2003). Frequency domain
analysis of noise in autoregulated gene circuits. Proc Natl Acad Sci U S A.

[2395] Becskei A., Serrano L. (2000). Engineering stability
in gene networks
by autoregulation. Nature.

[2396] Wall M. E., Hlavacek W. S., Savageau M. A. (2003). Design
principles
for regulator gene expression in a repressible gene circuit. J Mol Biol.

[2397] Andrews, L. B. ; Nielsen, A. A. K. ; Voigt, C. A. Cellular checkpoint control using programmable sequential logic. Science 2018, 361 (6408), 10.1126/science.aap8987. 30237327

[2398] Ivask, A. ; Rolova, T. ; Kahru, A. A suite of recombinant luminescent bacterial strains for the quantification of bioavailable heavy metals and toxicity testing. Bmc Biotechnology 2009, 9, 41. 10.1186/1472-6750-9-41 19426479 PMC2685376

[2399] Wang B. J., Barahona M., Buck M. (2013). A modular
cell-based
biosensor using engineered genetic logic circuits to detect and integrate
multiple environmental signals. Biosens Bioelectron.

[2400] Pierce F. J., Nowak P. (1999). Aspects of precision agriculture. Advances
in agronomy.

[2401] Sishodia R. P., Ray R. L., Singh S. K. (2020). Applications of
remote sensing in precision agriculture: A review. Remote sensing.

[2402] Rigoulot S. B., Schimel T. M., Lee J. H., Sears R. G., Brabazon H., Layton J. S., Li L., Meier K. A., Poindexter M. R., Schmid M. J. (2021). Imaging of multiple
fluorescent proteins in canopies enables synthetic biology in plants. Plant Biotechnology Journal.

[2403] Shpigel E., Shemer B., Elad T., Belkin S. (2022). Remote Bio-Detection
of Buried Landmines by Luminescent Microbial Sensors. Engineering Proceedings.

[2404] Mitiouchkina T., Mishin A. S., Somermeyer L. G., Markina N. M., Chepurnyh T. V., Guglya E. B., Karataeva T. A., Palkina K. A., Shakhova E. S., Fakhranurova L. I. (2020). Plants
with genetically encoded autoluminescence. Nature
Biotechnology.

[2405] Shakhova E. S., Karataeva T. A., Markina N. M., Mitiouchkina T., Palkina K. A., Perfilov M. M., Wood M. G., Hoang T. T., Hall M. P., Fakhranurova L. I. (2024). An improved pathway for autonomous
bioluminescence imaging in eukaryotes. Nature
Methods.

[2406] Beddow J. M., Pardey P. G., Chai Y., Hurley T. M., Kriticos D. J., Braun H.-J., Park R. F., Cuddy W. S., Yonow T. (2015). Research investment implications of shifts in the global geography
of wheat stripe rust. Nature Plants.

[2407] Cross, J. F. ; Cobo, N. ; Drewry, D. T. Non-invasive diagnosis of wheat stripe rust progression using hyperspectral reflectance. Frontiers in Plant Science 2024, 15, 10.3389/fpls.2024.1429879. PMC11422131 39323538

[2408] Shaw, A. K. ; Medford, J. ; Antunes, M. ; McCormick, W. S. ; Wicker, D. Hyperspectral exploitation with plant sentinels. In Chemical and Biological Sensing VIII; SPIE: 2007; Vol. 6554, pp 223-234.

[2409] Chemla, Y. ; Levin, I. ; Fan, Y. ; Johnson, A. A. ; Coley, C. W. ; Voigt, C. A. Hyperspectral reporters for long-distance and wide-area detection of gene expression in living bacteria. Nat Biotechnol 2025, 10.1038/s41587-025-02622-y. 40216953

[2410] Cheng H. Y., Masiello C. A., Del Valle I., Gao X., Bennett G. N., Silberg J. J. (2018). Ratiometric Gas Reporting: A Nondisruptive
Approach To Monitor Gene Expression in Soils. ACS Synth Biol.

[2411] Belkin S., Yagur-Kroll S., Kabessa Y., Korouma V., Septon T., Anati Y., Zohar-Perez C., Rabinovitz Z., Nussinovitch A., Agranat A. J. (2017). Remote detection
of buried landmines using a bacterial sensor. Nat Biotechnol.

[2412] Ye Z. X., Li S., Hennigan J. N., Lebeau J., Moreb E. A., Wolf J., Lynch M. D. (2021). Two-stage dynamic
deregulation of metabolism improves process robustness & scalability
in engineered E. coli. Metab. Eng..

[2413] Lynch M. D. (2021). The bioprocess TEA calculator: An
online technoeconomic
analysis tool to evaluate the commercial competitiveness of potential
bioprocesses. Metab. Eng..

[2414] Mimee M., Tucker A. C., Voigt C. A., Lu T. K. (2016). Programming
a Human Commensal Bacterium, Bacteroides thetaiotaomicron, to Sense
and Respond to Stimuli in the Murine Gut Microbiota (vo 1, pg 62,
2015). Cell Systems.

[2415] Larson M. H., Gilbert L. A., Wang X., Lim W. A., Weissman J. S., Qi L. S. (2013). CRISPR interference
(CRISPRi) for
sequence-specific control of gene expression. Nat Protoc.

[2416] Larson M. H., Gilbert L. A., Wang X. W., Lim W. A., Weissman J. S., Qi L. S. (2013). CRISPR interference (CRISPRi) for
sequence-specific control of gene expression. Nature Protocols.

[2417] Farasat, I. ; Salis, H. M. A Biophysical Model of CRISPR/Cas9 Activity for Rational Design of Genome Editing and Gene Regulation. Plos Computational Biology 2016, 12 (1), e1004724. 10.1371/journal.pcbi.1004724 26824432 PMC4732943

[2418] Peters J. M., Colavin A., Shi H., Czarny T. L., Larson M. H., Wong S., Hawkins J. S., Lu C. H. S., Koo B. M., Marta E. (2016). A Comprehensive, CRISPR-based
Functional Analysis of Essential Genes in Bacteria. Cell.

[2419] Peters J. M., Koo B. M., Patino R., Heussler G. E., Hearne C. C., Qu J., Inclan Y. F., Hawkins J. S., Lu C. H. S., Silvis M. R. (2019). Enabling genetic analysis
of diverse bacteria with Mobile-CRISPRi. Nat
Microbiol.

[2420] Li, L. ; Wei, K. ; Zheng, G. ; Liu, X. ; Chen, S. ; Jiang, W. ; Lu, Y. CRISPR-Cpf1-Assisted Multiplex Genome Editing and Transcriptional Repression in Streptomyces. Appl Environ Microbiol 2018, 84 (18), 10.1128/AEM.00827-18. PMC6121969 29980561

[2421] Kiattisewee C., Dong C., Fontana J., Sugianto W., Peralta-Yahya P., Carothers J. M., Zalatan J. G. (2021). Portable bacterial
CRISPR transcriptional activation enables metabolic engineering in
Pseudomonas putida. Metab. Eng..

[2422] Tan, S. Z. ; Reisch, C. R. ; Prather, K. L. J. A Robust CRISPR Interference Gene Repression System in Pseudomonas. J Bacteriol 2018, 200 (7), 10.1128/JB.00575-17. PMC5847647 29311279

[2423] Batianis C., Kozaeva E., Damalas S. G., Martin-Pascual M., Volke D. C., Nikel P. I., Martins
Dos Santos V. A. P. (2020). An expanded
CRISPRi toolbox for tunable control of gene expression in Pseudomonas
putida. Microb Biotechnol.

[2424] Luo J., Efimova E., Volke D. C., Santala V., Santala S. (2022). Engineering
cell morphology by CRISPR interference in Acinetobacter baylyi ADP1. Microb Biotechnol.

[2425] Cho S., Choe D., Lee E., Kim S. C., Palsson B., Cho B. K. (2018). High-Level dCas9 Expression Induces Abnormal Cell Morphology
in Escherichia coli. ACS Synth Biol.

[2426] Jones D. L., Leroy P., Unoson C., Fange D., Curic V., Lawson M. J., Elf J. (2017). Kinetics of
dCas9 target
search in Escherichia coli. Science.

[2427] Boch J., Scholze H., Schornack S., Landgraf A., Hahn S., Kay S., Lahaye T., Nickstadt A., Bonas U. (2009). Breaking the code of
DNA binding
specificity of TAL-type III effectors. Science.

[2428] Hurt J. A., Thibodeau S. A., Hirsh A. S., Pabo C. O., Joung J. K. (2003). Highly
specific zinc finger proteins obtained by directed
domain shuffling and cell-based selection. Proc
Natl Acad Sci U S A.

[2429] Durai S., Bosley A., Abulencia A. B., Chandrasegaran S., Ostermeier M. (2006). A bacterial one-hybrid selection
system for interrogating zinc finger-DNA interactions. Comb Chem High Throughput Screen.

[2430] Copeland M. F., Politz M. C., Johnson C. B., Markley A. L., Pfleger B. F. (2016). A transcription activator-like effector
(TALE) induction
system mediated by proteolysis. Nat Chem Biol.

[2431] Politz M. C., Copeland M. F., Pfleger B. F. (2013). Artificial repressors
for controlling gene expression in bacteria. Chem Commun (Camb).

[2432] Na D., Yoo S. M., Chung H., Park H., Park J. H., Lee S. Y. (2013). Metabolic engineering
of Escherichia coli using synthetic
small regulatory RNAs. Nature Biotechnology.

[2433] Noh M., Yoo S. M., Kim W. J., Lee S. Y. (2017). Gene Expression
Knockdown by Modulating Synthetic Small RNA Expression in Escherichia
coli. Cell Systems.

[2434] Lahiry A., Stimple S. D., Wood D. W., Lease R. A. (2017). Retargeting
a Dual-Acting sRNA for Multiple mRNA Transcript Regulation. ACS Synth Biol.

[2435] Sung M., Yoo S., Jun R., Lee J., Lee S., Na D. (2016). Optimization of phage lambda promoter
strength for
synthetic small regulatory RNA-based metabolic engineering. Biotechnol Bioproc E.

[2436] Ghodasara A., Voigt C. A. (2017). Balancing gene expression
without
library construction via a reusable sRNA pool. Nucleic Acids Res.

[2437] Apura P., Saramago M., Peregrina A., Viegas S. C., Carvalho S. M., Saraiva L. M., Arraiano C. M., Domingues S. (2020). Tailor-made sRNAs: a plasmid tool to control the expression
of target mRNAs in Pseudomonas putida. Plasmid.

[2438] Yang S., Wang Y., Wei C., Liu Q., Jin X., Du G., Chen J., Kang Z. (2018). A new sRNA-mediated
posttranscriptional regulation system for Bacillus subtilis. Biotechnol Bioeng.

[2439] Gao W., He Y., Zhang F., Zhao F., Huang C., Zhang Y., Zhao Q., Wang S., Yang C. (2019). Metabolic
engineering of Bacillus amyloliquefaciens LL3 for enhanced poly-gamma-glutamic
acid synthesis. Microb Biotechnol.

[2440] Uguru G. C., Mondhe M., Goh S., Hesketh A., Bibb M. J., Good L., Stach J. E. (2013). Synthetic
RNA Silencing
of Actinorhodin Biosynthesis in Streptomyces coelicolor A3(2). PLoS One.

[2441] Gur E., Sauer R. T. (2008). Evolution of the ssrA degradation
tag in Mycoplasma:
specificity switch to a different protease. Proc Natl Acad Sci U S A.

[2442] Cameron D. E., Collins J. J. (2014). Tunable protein
degradation in bacteria. Nat Biotechnol.

[2443] Taxis C., Stier G., Spadaccini R., Knop M. (2009). Efficient protein depletion by genetically controlled deprotection
of a dormant N-degron. Mol Syst Biol.

[2444] Fernandez-Rodriguez J., Voigt C. A. (2016). Post-translational
control of genetic
circuits using Potyvirus proteases. Nucleic
Acids Res.

[2445] Gao X., Yeom J., Groisman E. A. (2019). The expanded specificity and physiological
role of a widespread N-degron recognin. Proc
Natl Acad Sci U S A.

[2446] Li L., Mu L., Wang X., Yu J., Hu R., Li Z. (2017). A novel expression vector for the
secretion of abaecin in Bacillus
subtilis. Braz J Microbiol.

[2447] Nguyen, J. ; Lara-Gutierrez, J. ; Stocker, R. Environmental fluctuations and their effects on microbial communities, populations and individuals. Fems Microbiol Rev 2021, 45 (4), 10.1093/femsre/fuaa068. PMC8371271 33338228

[2448] Fierer N. (2017). Embracing the unknown: disentangling the complexities
of the soil microbiome. Nat Rev Microbiol.

[2449] Turner T. R., James E. K., Poole P. S. (2013). The plant microbiome. Genome Biol.

[2450] Jansson J. K., Hofmockel K. S. (2020). Soil microbiomes and climate change. Nat Rev Microbiol.

[2451] Kim J., Darlington A., Salvador M., Utrilla J., Jimenez J. I. (2020). Trade-offs
between gene expression, growth and phenotypic diversity in microbial
populations. Curr Opin Biotechnol.

[2452] Kafri M., Metzl-Raz E., Jona G., Barkai N. (2016). The Cost of
Protein Production. Cell Rep.

[2453] Klumpp S., Zhang Z., Hwa T. (2009). Growth rate-dependent
global effects on gene expression in bacteria. Cell.

[2454] Klumpp S., Hwa T. (2014). Bacterial growth: global effects
on gene expression, growth feedback and proteome partition. Curr Opin Biotechnol.

[2455] Zhang R., Li J., Melendez-Alvarez J., Chen X., Sochor P., Goetz H., Zhang Q., Ding T., Wang X., Tian X. J. (2020). Topology-dependent
interference of synthetic gene circuit function by growth feedback. Nat Chem Biol.

[2456] Nikolados E. M., Weisse A. Y., Ceroni F., Oyarzun D. A. (2019). Growth
Defects and Loss-of-Function in Synthetic Gene Circuits. ACS Synth Biol.

[2457] Smith, D. R. ; Chapman, M. R. Economical evolution: microbes reduce the synthetic cost of extracellular proteins. mBio 2010, 1 (3), 10.1128/mBio.00131-10. PMC2932507 20824102

[2458] Akashi H., Gojobori T. (2002). Metabolic efficiency
and amino acid
composition in the proteomes of Escherichia coli and Bacillus subtilis. Proc Natl Acad Sci U S A.

[2459] Peters M., Heinaru E., Talpsep E., Wand H., Stottmeister U., Heinaru A., Nurk A. (1997). Acquisition
of a deliberately
introduced phenol degradation operon, pheBA, by different indigenous
Pseudomonas species. Appl Environ Microbiol.

[2460] Kivisaar M. A., Habicht J. K., Heinaru A. L. (1989). Degradation of phenol
and m-toluate in Pseudomonas sp. strain EST1001 and its Pseudomonas
putida transconjugants is determined by a multiplasmid system. J Bacteriol.

[2461] Heinaru E., Truu J., Stottmeister U., Heinaru A. (2000). Three types of phenol and p-cresol catabolism in phenol-
and p-cresol-degrading bacteria isolated from river water continuously
polluted with phenolic compounds. FEMS Microbiol
Ecol.

[2462] Kivisaar M., Horak R., Kasak L., Heinaru A., Habicht J. (1990). Selection of independent plasmids
determining phenol
degradation in Pseudomonas putida and the cloning and expression of
genes encoding phenol monooxygenase and catechol 1,2-dioxygenase. Plasmid.

[2463] Carrera J., Rodrigo G., Singh V., Kirov B., Jaramillo A. (2011). Empirical model and in vivo characterization of the
bacterial response to synthetic gene expression show that ribosome
allocation limits growth rate. Biotechnol J.

[2464] McBride C. D., Del Vecchio D. (2021). Predicting Composition of Genetic
Circuits with Resource Competition: Demand and Sensitivity. ACS Synth Biol.

[2465] Weisse A. Y., Oyarzun D. A., Danos V., Swain P. S. (2015). Mechanistic
links between cellular trade-offs, gene expression, and growth. Proc Natl Acad Sci U S A.

[2466] Ceroni F., Algar R., Stan G. B., Ellis T. (2015). Quantifying
cellular capacity identifies gene expression designs with reduced
burden. Nature Methods.

[2467] Marr A. G. (1991). Growth rate of Escherichia coli. Microbiol Rev.

[2468] Kostinski S., Reuveni S. (2020). Ribosome Composition
Maximizes Cellular
Growth Rates in E. coli. Phys Rev Lett.

[2469] Tabor J. J., Bayer T. S., Simpson Z. B., Levy M., Ellington A. D. (2008). Engineering stochasticity in gene
expression. Mol Biosyst.

[2470] Gupta A., Reizman I. M., Reisch C. R., Prather K. L. (2017). Dynamic
regulation of metabolic flux in engineered bacteria using a pathway-independent
quorum-sensing circuit. Nat Biotechnol.

[2471] Ceroni F., Boo A., Furini S., Gorochowski T. E., Borkowski O., Ladak Y. N., Awan A. R., Gilbert C., Stan G. B., Ellis T. (2018). Burden-driven feedback
control of
gene expression. Nature Methods.

[2472] Tian T., Burrage K. (2004). Bistability and switching
in the
lysis/lysogeny genetic regulatory network of bacteriophage lambda. J Theor Biol.

[2473] Huang H. H., Qian Y., Del Vecchio D. (2018). A quasi-integral
controller for adaptation of genetic modules to variable ribosome
demand. Nat Commun.

[2474] Darlington A. P. S., Kim J., Jimenez J. I., Bates D. G. (2018). Dynamic
allocation of orthogonal ribosomes facilitates uncoupling of co-expressed
genes. Nat Commun.

[2475] Guan Y., Chen X., Shao B., Ji X., Xiang Y., Jiang G., Xu L., Lin Z., Ouyang Q., Lou C. (2022). Mitigating Host Burden of Genetic
Circuits by Engineering Autonegatively Regulated Parts and Improving
Functional Prediction. ACS Synth Biol.

[2476] Pitera D. J., Paddon C. J., Newman J. D., Keasling J. D. (2007). Balancing
a heterologous mevalonate pathway for improved isoprenoid production
in Escherichia coli. Metab Eng.

[2477] Nowroozi F. F., Baidoo E. E., Ermakov S., Redding-Johanson A. M., Batth T. S., Petzold C. J., Keasling J. D. (2014). Metabolic
pathway
optimization using ribosome binding site variants and combinatorial
gene assembly. Appl Microbiol Biotechnol.

[2478] Franden M. A., Jayakody L. N., Li W. J., Wagner N. J., Cleveland N. S., Michener W. E., Hauer B., Blank L. M., Wierckx N., Klebensberger J. (2018). Engineering Pseudomonas
putida KT2440 for efficient ethylene glycol utilization. Metab Eng.

[2479] Liu D., Zhang F. (2018). Metabolic Feedback Circuits Provide
Rapid Control of
Metabolite Dynamics. ACS Synth Biol.

[2480] Dahl R. H., Zhang F., Alonso-Gutierrez J., Baidoo E., Batth T. S., Redding-Johanson A. M., Petzold C. J., Mukhopadhyay A., Lee T. S., Adams P. D. (2013). Engineering dynamic pathway regulation using stress-response promoters. Nat Biotechnol.

[2481] Zhang F., Carothers J. M., Keasling J. D. (2012). Design of a dynamic
sensor-regulator system for production of chemicals and fuels derived
from fatty acids. Nat Biotechnol.

[2482] Niu T., Liu Y., Li J., Koffas M., Du G., Alper H. S., Liu L. (2018). Engineering
a Glucosamine-6-phosphate
Responsive glmS Ribozyme Switch Enables Dynamic Control of Metabolic
Flux in Bacillus subtilis for Overproduction of N-Acetylglucosamine. ACS Synth Biol.

[2483] Pfeiffer T., Schuster S., Bonhoeffer S. (2001). Cooperation
and competition in the evolution of ATP-producing pathways. Science.

[2484] Lipson D. A. (2015). The complex relationship between
microbial growth rate
and yield and its implications for ecosystem processes. Front Microbiol.

[2485] Nunan N., Schmidt H., Raynaud X. (2020). The ecology of heterogeneity:
soil bacterial communities and C dynamics. Philos
Trans R Soc Lond B Biol Sci.

[2486] Gyorfy Z., Draskovits G., Vernyik V., Blattner F. F., Gaal T., Posfai G. (2015). Engineered ribosomal RNA operon copy-number
variants of E. coli reveal the evolutionary trade-offs shaping rRNA
operon number. Nucleic Acids Res.

[2487] Izard J., Gomez Balderas C. D., Ropers D., Lacour S., Song X., Yang Y., Lindner A. B., Geiselmann J., de Jong H. (2015). A synthetic growth switch based on controlled expression
of RNA polymerase. Mol Syst Biol.

[2488] Zambrano M. M., Siegele D. A., Almiron M., Tormo A., Kolter R. (1993). Microbial Competition - Escherichia-Coli
Mutants That
Take over Stationary Phase Cultures. Science.

[2489] Zinser E. R., Kolter R. (2000). Prolonged stationary-phase incubation
selects for lrp mutations in Escherichia coli K-12. Journal of Bacteriology.

[2490] Utrilla J., O'Brien E. J., Chen K., McCloskey D., Cheung J., Wang H., Armenta-Medina D., Feist A. M., Palsson B. O. (2016). Global Rebalancing
of Cellular Resources
by Pleiotropic Point Mutations Illustrates a Multi-scale Mechanism
of Adaptive Evolution. Cell Systems.

[2491] Zinser E. R., Kolter R. (1999). Mutations enhancing
amino acid catabolism
confer a growth advantage in stationary phase. J Bacteriol.

[2492] Finkel S. E. (2006). Long-term survival during stationary
phase: evolution
and the GASP phenotype. Nat Rev Microbiol.

[2493] Kim J., Oliveros J. C., Nikel P. I., de Lorenzo V., Silva-Rocha R. (2013). Transcriptomic fingerprinting of Pseudomonas putida
under alternative physiological regimes. Environ
Microbiol Rep.

[2494] Faucher, C. ; Mazana, V. ; Kardacz, M. ; Parthuisot, N. ; Ferdy, J. B. ; Duneau, D. Step-Specific Adaptation and Trade-Off over the Course of an Infection by GASP Mutation Small Colony Variants. Mbio 2021, 12 (1), ARTN e01399-20. 10.1128/mBio.01399-20 PMC7845629 33436427

[2495] Hershberg, R. Mutation-The Engine of Evolution: Studying Mutation and Its Role in the Evolution of Bacteria. CSH Perspect Biol 2015, 7 (9), a018077. 10.1101/cshperspect.a018077 PMC4563715 26330518

[2496] Rosenberg S. M. (2001). Evolving responsively: Adaptive mutation. Nature Reviews Genetics.

[2497] Perfeito L., Fernandes L., Mota C., Gordo I. (2007). Adaptive mutations
in bacteria: High rate and small effects. Science.

[2498] Denamur E., Matic I. (2006). Evolution of mutation rates in bacteria. Molecular
Microbiology.

[2499] Dekel E., Alon U. (2005). Optimality and evolutionary
tuning
of the expression level of a protein. Nature.

[2500] Watt V. M., Ingles C. J., Urdea M. S., Rutter W. J. (1985). Homology
requirements for recombination in Escherichia coli. Proc Natl Acad Sci U S A.

[2501] Khasanov F. K., Zvingila D. J., Zainullin A. A., Prozorov A. A., Bashkirov V. I. (1992). Homologous recombination between
plasmid and chromosomal DNA in Bacillus subtilis requires approximately
70 bp of homology. Mol Gen Genet.

[2502] Elena C., Ravasi P., Castelli M. E., Peiru S., Menzella H. G. (2014). Expression
of codon optimized genes in microbial systems:
current industrial applications and perspectives. Front Microbiol.

[2503] Webster G. R., Teh A. Y., Ma J. K. (2017). Synthetic
gene design-The
rationale for codon optimization and implications for molecular pharming
in plants. Biotechnol Bioeng.

[2504] Siguier P., Perochon J., Lestrade L., Mahillon J., Chandler M. (2006). ISfinder: the reference centre for
bacterial insertion
sequences. Nucleic Acids Res.

[2505] Geng P., Leonard S. P., Mishler D. M., Barrick J. E. (2019). Synthetic
Genome Defenses against Selfish DNA Elements Stabilize Engineered
Bacteria against Evolutionary Failure. ACS Synth.
Biol..

[2506] Shen, X. M. ; Wang, Z. ; Huang, X. Q. ; Hu, H. B. ; Wang, W. ; Zhang, X. H. Developing genome-reduced Pseudomonas chlororaphis strains for the production of secondary metabolites. Bmc Genomics 2017, 18, ARTN 715. 10.1186/s12864-017-4127-2 PMC5594592 28893188

[2507] Liang, P. X. ; Zhang, Y. T. ; Xu, B. ; Zhao, Y. X. ; Liu, X. S. ; Gao, W. X. ; Ma, T. ; Yang, C. ; Wang, S. F. ; Liu, R. H. Deletion of genomic islands in the Pseudomonas putida KT2440 genome can create an optimal chassis for synthetic biology applications. Microbial Cell Factories 2020, 19 (1), ARTN 70. 10.1186/s12934-020-01329-w PMC7081699 32188438

[2508] Lieder, S. ; Nikel, P. I. ; de Lorenzo, V. ; Takors, R. Genome reduction boosts heterologous gene expression in Pseudomonas putida. Microbial Cell Factories 2015, 14, ARTN 23. 10.1186/s12934-015-0207-7 PMC4352270 25890048

[2509] Fan X., Zhang Y. T., Zhao F. J., Liu Y. J., Zhao Y. X., Wang S. F., Liu R. H., Yang C. (2020). Genome reduction enhances
production of polyhydroxyalkanoate and alginate oligosaccharide in
Pseudomonas mendocina. International Journal
of Biological Macromolecules.

[2510] Suarez, G. A. ; Renda, B. A. ; Dasgupta, A. ; Barrick, J. E. Reduced Mutation Rate and Increased Transformability of Transposon-Free Acinetobacter baylyi ADP1-ISx. Appl Environ Microbiol 2017, 83 (17), 10.1128/AEM.01025-17. PMC5561288 28667117

[2511] Wynands B., Otto M., Runge N., Preckel S., Polen T., Blank L. M., Wierckx N. (2019). Streamlined Pseudomonas
taiwanensis VLB120 Chassis Strains with Improved Bioprocess Features. ACS Synth Biol.

[2512] Blazejewski T., Ho H. I., Wang H. H. (2019). Synthetic
sequence
entanglement augments stability and containment of genetic information
in cells. Science.

[2513] Nicholson W. L., Fajardo-Cavazos P., Rebeil R., Slieman T. A., Riesenman P. J., Law J. F., Xue Y. (2002). Bacterial endospores
and their significance in stress resistance. Antonie Van Leeuwenhoek.

[2514] Setlow P. (2007). I will survive: DNA protection in
bacterial spores. Trends Microbiol.

[2515] Zhang F., Gao D., Lin J., Zhu M., Zhuang Z., Duan Y., Zhu X. (2020). Construction of Inducible
Genetic Switch for the Global Regulator WblA To Sustain Both Overproduction
of Tiancimycins and On-Demand Sporulation in Streptomyces sp. CB03234. ACS Synth Biol.

[2516] Qian J., Lu Z. X., Mancuso C. P., Jhuang H. Y., Del Carmen Barajas-Ornelas R., Boswell S. A., Ramirez-Guadiana F. H., Jones V., Sonti A., Sedlack K. (2020). Barcoded
microbial system for high-resolution object provenance. Science.

[2517] Ma X., Ashnest J. R. (2020). Tracing the Origins
of Agricultural Products with Barcoded
Microbial Spores. Molecular Plant.

[2518] Thomas C. M., Nielsen K. M. (2005). Mechanisms of, and
barriers to, horizontal
gene transfer between bacteria. Nat Rev Microbiol.

[2519] Brito I. L. (2021). Examining
horizontal gene transfer in microbial communities. Nat Rev Microbiol.

[2520] Van Elsas J. D., Turner S., Bailey M. J. (2003). Horizontal
gene
transfer in the phytosphere. New Phytol.

[2521] Paget E., Simonet P. (1994). On the Track of Natural Transformation
in Soil. FEMS Microbiol Ecol.

[2522] Khanna M., Stotzky G. (1992). Transformation of Bacillus-Subtilis
by DNA Bound on Montmorillonite and Effect of Dnase on the Transforming
Ability of Bound DNA. Appl Environ Microb.

[2523] Graham J. B., Istock C. A. (1978). Genetic exchange in Bacillus subtilis
in soil. Mol Gen Genet.

[2524] Chamier B., Lorenz M. G., Wackernagel W. (1993). Natural Transformation
of Acinetobacter-Calcoaceticus by Plasmid DNA Adsorbed on Sand and
Groundwater Aquifer Material. Appl Environ Microb.

[2525] Nielsen K. M., vanWeerelt M. D. M., Berg T. N., Bones A. M., Hagler A. N., vanElsas J. D. (1997). Natural transformation and availability
of transforming DNA to Acinetobacter calcoaceticus in soil microcosms. Appl Environ Microb.

[2526] Gallori E., Bazzicalupo M., Dalcanto L., Fani R., Nannipieri P., Vettori C., Stotzky G. (1994). Transformation of Bacillus-Subtilis
by DNA-Bound on Clay in Nonsterile Soil. FEMS
Microbiol Ecol.

[2527] Klumper U., Riber L., Dechesne A., Sannazzarro A., Hansen L. H., Sorensen S. J., Smets B. F. (2015). Broad host
range
plasmids can invade an unexpectedly diverse fraction of a soil bacterial
community. Isme J.

[2528] Geisenberger O., Ammendola A., Christensen B. B., Molin S., Schleifer K. H., Eberl L. (1999). Monitoring the conjugal
transfer of plasmid RP4 in activated sludge and in situ identification
of the transconjugants. Fems Microbiology Letters.

[2529] Lilley A. K., Fry J. C., Day M. J., Bailey M. J. (1994). In-Situ
Transfer of an Exogenously Isolated Plasmid between Pseudomonas Spp
in Sugar-Beet Rhizosphere. Microbiol-Uk.

[2530] Musovic S., Oregaard G., Kroer N., Sorensen S. J. (2006). Cultivation-independent
examination of horizontal transfer and host range of an IncP-1 plasmid
among gram-positive and gram-negative bacteria indigenous to the barley
rhizosphere. Appl Environ Microb.

[2531] Sengelov G., Kowalchuk G. A., Sorensen S. J. (2000). Influence of fungal-bacterial
interactions on bacterial conjugation in the residuesphere. FEMS Microbiol Ecol.

[2532] Kenzaka T., Tani K., Nasu M. (2010). High-frequency
phage-mediated
gene transfer in freshwater environments determined at single-cell
level. Isme J.

[2533] Tang X., Yu P. F., Tang L., Zhou M., Fan C. Z., Lu Y., Mathieu J., Xiong W. P., Alvarez P. J. (2019). Bacteriophages from Arsenic-Resistant
Bacteria Transduced
Resistance Genes, which Changed Arsenic Speciation and Increased Soil
Toxicity. Environ Sci Tech Let.

[2534] Humphrey, S. ; Fillol-Salom, A. ; Quiles-Puchalt, N. ; Ibarra-Chavez, R. ; Haag, A. F. ; Chen, J. ; Penades, J. R. Bacterial chromosomal mobility via lateral transduction exceeds that of classical mobile genetic elements. Nature Communications 2021, 12 (1), ARTN 6509. 10.1038/s41467-021-26004-5 PMC8575950 34750368

[2535] Keen, E. C. ; Bliskovsky, V. V. ; Malagon, F. ; Baker, J. D. ; Prince, J. S. ; Klaus, J. S. ; Adhya, S. L. Novel “Superspreader” Bacteriophages Promote Horizontal Gene Transfer by Transformation. Mbio 2017, 8 (1), ARTN e02115-16. 10.1128/mBio.02115-16 PMC5241400 28096488

[2536] Elken E., Heinaru E., Joesaar M., Heinaru A. (2020). Formation
of new PHE plasmids in pseudomonads in a phenol-polluted environment. Plasmid.

[2537] Kay E., Vogel T. M., Bertolla F., Nalin R., Simonet P. (2002). In situ transfer
of antibiotic resistance genes from transgenic (transplastomic) tobacco
plants to bacteria. Appl Environ Microb.

[2538] Cheng H.-Y., Masiello C. A., Bennett G. N., Silberg J. J. (2016). Volatile
Gas Production by Methyl Halide Transferase: An In Situ Reporter Of
Microbial Gene Expression In Soil. Environ.
Sci. Technol..

[2539] Xiao Y., Ng S., Nam K. H., Ke A. (2017). How type II
CRISPR-Cas establish immunity through Cas1-Cas2-mediated spacer integration. Nature.

[2540] Munck C., Sheth R. U., Freedberg D. E., Wang H. H. (2020). Recording mobile DNA in the gut microbiota using an
Escherichia coli CRISPR-Cas spacer acquisition platform. Nat Commun.

[2541] Murakami H., Sano K., Motomura K., Kuroda A., Hirota R. (2023). Assessment of horizontal gene transfer-mediated destabilization
of Synechococcus elongatus PCC 7942 biocontainment system. J Biosci Bioeng.

[2542] Getino, M. ; de la Cruz, F. Natural and Artificial Strategies To Control the Conjugative Transmission of Plasmids. Microbiol Spectr 2018, 6 (1), 10.1128/microbiolspec.MTBP-0015-2016. PMC11633558 29327679

[2543] Audette G. F., Manchak J., Beatty P., Klimke W. A., Frost L. S. (2007). Entry exclusion in F-like plasmids
requires intact
TraG in the donor that recognizes its cognate TraS in the recipient. Microbiology (Reading).

[2544] Marrero J., Waldor M. K. (2005). Interactions between
inner membrane
proteins in donor and recipient cells limit conjugal DNA transfer. Dev Cell.

[2545] Garcillan-Barcia M. P., de la Cruz F. (2008). Why is entry
exclusion an essential
feature of conjugative plasmids?. Plasmid.

[2546] Humbert, M. ; Huguet, K. T. ; Coulombe, F. ; Burrus, V. Entry Exclusion of Conjugative Plasmids of the IncA, IncC, and Related Untyped Incompatibility Groups. J Bacteriol 2019, 201 (10), 10.1128/JB.00731-18. PMC6482922 30858294

[2547] Garcillan-Barcia M. P., Jurado P., Gonzalez-Perez B., Moncalian G., Fernandez L. A., de la Cruz F. (2007). Conjugative
transfer can be inhibited by blocking relaxase activity within recipient
cells with intrabodies. Mol Microbiol.

[2548] Hayashi N., Lai Y., Fuerte-Stone J., Mimee M., Lu T. K. (2024). Cas9-assisted biological
containment
of a genetically engineered human commensal bacterium and genetic
elements. Nat Commun.

[2549] Caliando B. J., Voigt C. A. (2015). Targeted DNA degradation
using a
CRISPR device stably carried in the host genome. Nat Commun.

[2550] Nyerges A., Vinke S., Flynn R., Owen S. V., Rand E. A., Budnik B., Keen E., Narasimhan K., Marchand J. A., Baas-Thomas M. (2023). A swapped genetic code
prevents viral infections and gene transfer. Nature.

[2551] Zurcher J. F., Robertson W. E., Kappes T., Petris G., Elliott T. S., Salmond G. P. C., Chin J. W. (2022). Refactored genetic
codes enable bidirectional genetic isolation. Science.

[2552] Rackham O., Chin J. W. (2005). A network of orthogonal ribosome
x mRNA pairs. Nat Chem Biol.

[2553] Jia B., Qi H., Li B. Z., Pan S., Liu D., Liu H., Cai Y., Yuan Y. J. (2017). Orthogonal
Ribosome Biofirewall. ACS Synth. Biol..

[2554] An W., Chin J. W. (2009). Synthesis of orthogonal transcription-translation networks. Proc Natl Acad Sci U S A.

[2555] Motomura K., Sano K., Watanabe S., Kanbara A., Gamal Nasser A. H., Ikeda T., Ishida T., Funabashi H., Kuroda A., Hirota R. (2018). Synthetic Phosphorus
Metabolic Pathway
for Biosafety and Contamination Management of Cyanobacterial Cultivation. ACS Synth Biol.

[2556] Koeris M. (2015). Life in Our Phage World A Centennial
Field Guide to
the Earth's Most Diverse Inhabitants. Science.

[2557] Williamson K. E., Fuhrmann J. J., Wommack K. E., Radosevich M. (2017). Viruses in
Soil Ecosystems: An Unknown Quantity Within an Unexplored Territory. Annu Rev Virol.

[2558] Kuzyakov Y., Mason-Jones K. (2018). Viruses in
soil: Nano-scale undead
drivers of microbial life, biogeochemical turnover, and ecosystem
functions. Soil Biology & Biochemistry.

[2559] Ashelford K. E., Day M. J., Fry J. C. (2003). Elevated
abundance
of bacteriophage infecting bacteria in soil. Appl Environ Microbiol.

[2560] Braga L. P. P., Spor A., Kot W., Breuil M. C., Hansen L. H., Setubal J. C., Philippot L. (2020). Impact of
phages on soil bacterial communities and nitrogen availability under
different assembly scenarios. Microbiome.

[2561] Huang D., Yu P., Ye M., Schwarz C., Jiang X., Alvarez P. J. J. (2021). Enhanced mutualistic
symbiosis between
soil phages and bacteria with elevated chromium-induced environmental
stress. Microbiome.

[2562] Ghosh D., Roy K., Williamson K. E., White D. C., Wommack K. E., Sublette K. L., Radosevich M. (2008). Prevalence
of lysogeny among soil bacteria and presence of 16S rRNA and trzN
genes in viral-community DNA. Appl Environ Microbiol.

[2563] Keel C., Ucurum Z., Michaux P., Adrian M., Haas D. (2002). Deleterious
impact of a virulent bacteriophage on survival and biocontrol
activity of Pseudomonas fluorescens strain CHAO in natural soil. Mol Plant Microbe Interact.

[2564] Tzipilevich E., Benfey P. N. (2021). Phage-Resistant
Bacteria Reveal a
Role for Potassium in Root Colonization. mBio.

[2565] Labrie S. J., Samson J. E., Moineau S. (2010). Bacteriophage resistance
mechanisms. Nat Rev Microbiol.

[2566] Hampton H. G., Watson B. N. J., Fineran P. C. (2020). The arms
race between
bacteria and their phage foes. Nature.

[2567] Hille, F. ; Charpentier, E. CRISPR-Cas: biology, mechanisms and relevance. Philos Trans R Soc Lond B Biol Sci 2016, 371 (1707), 20150496 10.1098/rstb.2015.0496. 27672148 PMC5052741

[2568] Hynes, A. P. ; Labrie, S. J. ; Moineau, S. Programming Native CRISPR Arrays for the Generation of Targeted Immunity. mBio 2016, 7 (3), 10.1128/mBio.00202-16. PMC4959665 27143383

[2569] Halter M. C., Zahn J. A. (2018). Characterization
of a novel lytic
bacteriophage from an industrial Escherichia coli fermentation process
and elimination of virulence using a heterologous CRISPR-Cas9 system. J Ind Microbiol Biotechnol.

[2570] Jakutyte-Giraitiene L., Gasiunas G. (2016). Design of a CRISPR-Cas
system to
increase resistance of Bacillus subtilis to bacteriophage SPP1. J Ind Microbiol Biotechnol.

[2571] Dupuis M. E., Villion M., Magadan A. H., Moineau S. (2013). CRISPR-Cas
and restriction-modification systems are compatible and increase phage
resistance. Nat Commun.

[2572] Pourcel C., Touchon M., Villeriot N., Vernadet J. P., Couvin D., Toffano-Nioche C., Vergnaud G. (2019). CRISPRCasdb a successor of CRISPRdb
containing CRISPR
arrays and cas genes from complete genome sequences, and tools to
download and query lists of repeats and spacers. Nucleic Acids Res.

[2573] Bradde S., Nourmohammad A., Goyal S., Balasubramanian V. (2020). The size of
the immune repertoire of bacteria. Proc Natl
Acad Sci U S A.

[2574] Robertson W. E., Funke L. F. H., de
la Torre D., Fredens J., Elliott T. S., Spinck M., Christova Y., Cervettini D., Boge F. L., Liu K. C. (2021). Sense
codon reassignment enables viral resistance and encoded polymer synthesis. Science.

[2575] Rovner A. J., Haimovich A. D., Katz S. R., Li Z., Grome M. W., Gassaway B. M., Amiram M., Patel J. R., Gallagher R. R., Rinehart J. (2015). Recoded organisms engineered
to depend on synthetic amino acids. Nature.

[2576] Mandell D. J., Lajoie M. J., Mee M. T., Takeuchi R., Kuznetsov G., Norville J. E., Gregg C. J., Stoddard B. L., Church G. M. (2015). Biocontainment
of genetically modified organisms by
synthetic protein design. Nature.

[2577] Ostrov N., Nyerges A., Chiappino-Pepe A., Rudolph A., Baas-Thomas M., Church G. M. (2020). Synthetic genomes
with altered genetic codes. Current Opinion
in Systems Biology.

[2578] Ostrov N., Landon M., Guell M., Kuznetsov G., Teramoto J., Cervantes N., Zhou M., Singh K., Napolitano M. G., Moosburner M. (2016). Design, synthesis, and
testing toward a 57-codon genome. Science.

[2579] Moe-Behrens G. H., Davis R., Haynes K. A. (2013). Preparing synthetic
biology for the world. Front Microbiol.

[2580] Cameron D. E., Bashor C. J., Collins J. J. (2014). A brief
history
of synthetic biology. Nat Rev Microbiol.

[2581] Isabella V. M., Ha B. N., Castillo M. J., Lubkowicz D. J., Rowe S. E., Millet Y. A., Anderson C. L., Li N., Fisher A. B., West K. A. (2018). Development of a synthetic
live bacterial therapeutic for the human metabolic disease phenylketonuria. Nat Biotechnol.

[2582] Huang Y., Lin X., Yu S., Chen R., Chen W. (2022). Intestinal Engineered Probiotics
as Living Therapeutics: Chassis
Selection, Colonization Enhancement, Gene Circuit Design, and Biocontainment. ACS Synth Biol.

[2583] Gonzalez-Morales S. I., Pacheco-Gutierrez N.
B., Ramirez-Rodriguez C. A., Brito-Bello A. A., Estrella-Hernandez P., Herrera-Estrella L., Lopez-Arredondo D. L. (2020). Metabolic engineering of phosphite metabolism in Synechococcus
elongatus PCC 7942 as an effective measure to control biological contaminants
in outdoor raceway ponds. Biotechnol Biofuels.

[2584] Whitford C. M., Dymek S., Kerkhoff D., Marz C., Schmidt O., Edich M., Droste J., Pucker B., Ruckert C., Kalinowski J. (2018). Auxotrophy
to Xeno-DNA: an exploration
of combinatorial mechanisms for a high-fidelity biosafety system for
synthetic biology applications. J Biol Eng.

[2585] Halvorsen T. M., Ricci D. P., Park D. M., Jiao Y., Yung M. C. (2022). Comparison of Kill Switch Toxins
in Plant-Beneficial
Pseudomonas fluorescens Reveals Drivers of Lethality, Stability, and
Escape. ACS Synth Biol.

[2586] Stirling F., Bitzan L., O'Keefe S., Redfield E., Oliver J. W. K., Way J., Silver P. A. (2017). Rational
Design
of Evolutionarily Stable Microbial Kill Switches. Mol Cell.

[2587] Piraner D. I., Abedi M. H., Moser B. A., Lee-Gosselin A., Shapiro M. G. (2017). Tunable thermal bioswitches for in vivo control of
microbial therapeutics. Nat Chem Biol.

[2588] Stirling F., Naydich A., Bramante J., Barocio R., Certo M., Wellington H., Redfield E., O'Keefe S., Gao S., Cusolito A. (2020). Synthetic Cassettes for pH-Mediated Sensing,
Counting, and Containment. Cell Rep.

[2589] Ishikawa M., Kojima T., Hori K. (2021). Development of a Biocontained
Toluene-Degrading Bacterium for Environmental Protection. Microbiol Spectr.

[2590] Rottinghaus A. G., Ferreiro A., Fishbein S. R. S., Dantas G., Moon T. S. (2022). Genetically stable CRISPR-based kill
switches for engineered
microbes. Nat Commun.

[2591] Chan C. T., Lee J. W., Cameron D. E., Bashor C. J., Collins J. J. (2016). 'Deadman' and 'Passcode'
microbial kill switches for
bacterial containment. Nat Chem Biol.

[2592] Gallagher R. R., Patel J. R., Interiano A. L., Rovner A. J., Isaacs F. J. (2015). Multilayered
genetic safeguards limit
growth of microorganisms to defined environments. Nucleic Acids Research.

[2593] Xuan W., Schultz P. G. (2017). A Strategy for Creating
Organisms
Dependent on Noncanonical Amino Acids. Angew
Chem Int Ed Engl.

[2594] Kong W., Wanda S. Y., Zhang X., Bollen W., Tinge S. A., Roland K. L., Curtiss R. (2008). Regulated programmed lysis of recombinant Salmonella
in host tissues
to release protective antigens and confer biological containment. Proc Natl Acad Sci U S A.

[2595] Kim D., Lee J. W. (2020). Genetic Biocontainment
Systems for the Safe Use of
Engineered Microorganisms. Biotechnol Bioproc
E.

[2596] Kong W., Brovold M., Koeneman B. A., Clark-Curtiss J., Curtiss R. (2012). Turning self-destructing Salmonella
into a universal DNA vaccine delivery platform. Proc Natl Acad Sci U S A.

[2597] Xu X., Dinesen C., Pioppi A., Kovacs A. T., Lozano-Andrade C. N. (2025). Composing
a microbial symphony: synthetic communities for promoting plant growth. Trends Microbiol.

[2598] Trivedi P., Leach J. E., Tringe S. G., Sa T., Singh B. K. (2020). Plant-microbiome interactions: from community assembly
to plant health. Nat Rev Microbiol.

[2599] Kehe J., Kulesa A., Ortiz A., Ackerman C. M., Thakku S. G., Sellers D., Kuehn S., Gore J., Friedman J., Blainey P. C. (2019). Massively parallel screening of synthetic
microbial communities. Proc Natl Acad Sci U
S A.

[2600] Niu B., Paulson J. N., Zheng X., Kolter R. (2017). Simplified and representative
bacterial community of maize roots. Proc Natl
Acad Sci U S A.

[2601] Wang C.-J., Yang W., Wang C., Gu C., Niu D.-D., Liu H.-X., Wang Y.-P., Guo J.-H. (2012). Induction
of Drought Tolerance in Cucumber Plants by a Consortium of Three Plant
Growth-Promoting Rhizobacterium Strains. PLOS
ONE.

[2602] Yang W., Zheng L., Liu H.-X., Wang K.-B., Yu Y.-Y., Luo Y.-M., Guo J.-H. (2014). Evaluation of the
effectiveness of a consortium of three plant-growth promoting rhizobacteria
for biocontrol of cotton Verticillium wilt. Biocontrol Science and Technology.

[2603] Zhang, L.-N. ; Wang, D.-C. ; Hu, Q. ; Dai, X.-Q. ; Xie, Y.-S. ; Li, Q. ; Liu, H.-M. ; Guo, J.-H. Consortium of Plant Growth-Promoting Rhizobacteria Strains Suppresses Sweet Pepper Disease by Altering the Rhizosphere Microbiota. Frontiers in Microbiology 2019, 10, 10.3389/fmicb.2019.01668. PMC6664061 31396185

[2604] Zhang, J. ; Ahmed, W. ; Dai, Z. ; Zhou, X. ; He, Z. ; Wei, L. ; Ji, G. Microbial Consortia: An Engineering Tool to Suppress Clubroot of Chinese Cabbage by Changing the Rhizosphere Bacterial Community Composition. Biology 2022, 11, 918. 10.3390/biology11060918 35741438 PMC9219690

[2605] Shayanthan A., Ordoñez P. A.
C., Oresnik I. J. (2022). The role
of synthetic microbial communities (SynCom) in sustainable agriculture. Frontiers in Agronomy.

[2606] Yuan J., Zhao K., Tan X., Xue R., Zeng Y., Ratti C., Trivedi P. (2023). Perspective on the
development of synthetic microbial community (SynCom) biosensors. Trends Biotechnol.

[2607] Delgado-Baquerizo M., Singh B. K., Liu Y. R., Saez-Sandino T., Coleine C., Munoz-Rojas M., Bastida F., Trivedi P. (2025). Integrating
ecological and evolutionary frameworks for SynCom success. New Phytol.

[2608] Yadav A., Chen M., Acharya S. M., Yang Y., Zhao T. Z., Chakraborty R. (2024). A stable 15-member
bacterial SynCom
promotes Brachypodium growth under drought stress. bioRxiv.

[2609] Li Y., Li R., Liu R., Shi J., Qiu X., Lei J., Zhao X., Wang C., Ge M., Xu H. (2025). A simplified SynCom based on core-helper strain
interactions enhances
symbiotic nitrogen fixation in soybean. J Integr
Plant Biol.

[2610] Hernandez-Garcia J. A., Bernal J. S., Antony-Babu S., Villa-Tanaca L., Hernandez-Rodriguez C., De-la-Vega-Camarillo E. (2025). Teosinte-derived
SynCom and precision biofertilization modulate the maize microbiome,
enhancing growth, yield, and soil functionality in a Mexican field. Front Microbiol.

[2611] Zhang, W. Strategic Engineering of Synthetic Microbial Communities (SynComs) for Optimizing Plant Health and Yield in Agriculture. Molecular Microbiology Research 2024, 14, 10.5376/mmr.2024.14.0014.

[2612] Hao, X. ; Gu, Y. ; Zhang, H. ; Wang, X. ; Liu, X. ; Chen, C. ; Wang, C. ; Zhang, X. ; Liu, X. ; Shen, X. Synthetic Microbial Community Promotes Bacterial Communities Leading to Soil Multifunctionality in Desertified Land. Microorganisms 2024, 12 (6), 1117 10.3390/microorganisms12061117. 38930499 PMC11205429

[2613] Arnault, G. ; Marais, C. ; Preveaux, A. ; Briand, M. ; Poisson, A. S. ; Sarniguet, A. ; Barret, M. ; Simonin, M. Seedling microbiota engineering using bacterial synthetic community inoculation on seeds. FEMS Microbiol Ecol 2024, 100 (4), 10.1093/femsec/fiae027. PMC10977042 38503562

[2614] Flores-Núñez V. M., Camarena-Pozos D. A., Chávez-González J. D., Alcalde-Vázquez R., Vázquez-Sánchez M. N., Hernández-Melgar A. G., Xool-Tamayo J., Moreno-Ulloa A., Martínez L. P. P. (2023). Synthetic
communities increase microbial diversity and productivity of Agave
tequilana plants in the field. Phytobiomes Journal.

[2615] Gastelum G., Gomez-Gil B., Olmedo-Alvarez G., Rocha J. (2025). Harnessing emergent
properties of microbial consortia for Agriculture:
Assembly of the Xilonen SynCom. Biofilm.

[2616] Lee Y., Ko Y. M., Kwak Y. S. (2025). Genetic
and Nutritional Dynamics
of SynCom in Suppressing Apple Fire Blight. Plant Pathol J.

[2617] Albright M. B. N., Louca S., Winkler D. E., Feeser K. L., Haig S.-J., Whiteson K. L., Emerson J. B., Dunbar J. (2022). Solutions
in microbiome engineering: prioritizing barriers to organism establishment. The ISME Journal.

[2618] Mee M. T., Collins J. J., Church G. M., Wang H. H. (2014). Syntrophic
exchange in synthetic microbial communities. Proc Natl Acad Sci U S A.

[2619] Losoi P. S., Santala V. P., Santala S. M. (2019). Enhanced
Population
Control in a Synthetic Bacterial Consortium by Interconnected Carbon
Cross-Feeding. ACS Synth Biol.

[2620] LaSarre B., McCully A. L., Lennon J. T., McKinlay J. B. (2017). Microbial
mutualism dynamics governed by dose-dependent toxicity of cross-fed
nutrients. ISME J.

[2621] Kerner A., Park J., Williams A., Lin X. N. (2012). A programmable
Escherichia coli consortium via tunable symbiosis. PLoS One.

[2622] Kong W., Meldgin D. R., Collins J. J., Lu T. (2018). Designing
microbial consortia with defined social interactions. Nature Chemical Biology.

[2623] Balagadde F. K., Song H., Ozaki J., Collins C. H., Barnet M., Arnold F. H., Quake S. R., You L. (2008). A synthetic
Escherichia coli predator-prey ecosystem. Mol
Syst Biol.

[2624] Shepherd E. S., DeLoache W. C., Pruss K. M., Whitaker W. R., Sonnenburg J. L. (2018). An exclusive metabolic niche enables
strain engraftment
in the gut microbiota. Nature.

[2625] Wassenaar T. M., Beimfohr C., Geske T., Zimmermann K. (2014). Voluntarily
exposure to a single, high dose of probiotic Escherichia coli results
in prolonged colonisation. Benef Microbes.

[2626] Mondel M., Schroeder B. O., Zimmermann K., Huber H., Nuding S., Beisner J., Fellermann K., Stange E. F., Wehkamp J. (2009). Probiotic
E. coli treatment mediates
antimicrobial human beta-defensin synthesis and fecal excretion in
humans. Mucosal Immunol.

[2627] Aparicio, T. ; Silbert, J. ; Cepeda, S. ; de Lorenzo, V. Propagation of recombinant genes through complex microbiomes with synthetic mini-RP4 plasmid vectors. BioDesign Research 2022, 2022, 9850305 10.34133/2022/9850305. 37850127 PMC10521647

[2628] Ronda C., Chen S. P., Cabral V., Yaung S. J., Wang H. H. (2019). Metagenomic engineering of the mammalian
gut microbiome
in situ. Nat Methods.

[2629] Taghavi S., Barac T., Greenberg B., Borremans B., Vangronsveld J., van der Lelie D. (2005). Horizontal
Gene Transfer to Endogenous Endophytic Bacteria from Poplar Improves
Phytoremediation of Toluene. Applied and Environmental
Microbiology.

[2630] French K. E., Zhou Z., Terry N. (2020). Horizontal
'gene drives'
harness indigenous bacteria for bioremediation. Sci Rep.

[2631] Hsu B. B., Plant I. N., Lyon L., Anastassacos F. M., Way J. C., Silver P. A. (2020). In situ reprogramming of gut bacteria
by oral delivery. Nat Commun.

[2632] Carbonell P., Jervis A. J., Robinson C. J., Yan C., Dunstan M., Swainston N., Vinaixa M., Hollywood K. A., Currin A., Rattray N. J. W. (2018). An automated Design-Build-Test-Learn
pipeline for enhanced microbial production of fine chemicals. Commun Biol.

[2633] Radivojevic T., Costello Z., Workman K., Garcia Martin H. (2020). A machine
learning Automated Recommendation Tool for synthetic biology. Nat Commun.

[2634] Holowko M. B., Frow E. K., Reid J. C., Rourke M., Vickers C. E. (2021). Building a biofoundry. Synth
Biol (Oxf).

[2635] Zhang J., Chen Y., Fu L., Guo E., Wang B., Dai L., Si T. (2021). Accelerating strain
engineering in biofuel research via build and test automation of synthetic
biology. Curr Opin Biotechnol.

[2636] Clarke L. J., Kitney R. I. (2016). Synthetic biology
in the UK - An
outline of plans and progress. Synth Syst Biotechnol.

[2637] Zhao G. (2023). Biofoundry
and its industrial application. Synthetic Biology
Journal.

[2638] Zhu, Y. ; Kong, Z. ; Wu, J. ; Liu, W. ; Han, Y. ; Yin, M. ; Xu, H. ; Hsieh, C.-Y. ; Hou, T. Generative AI for Controllable Protein Sequence Design: A Survey. arXiv 2024, 2402.10516. 10.48550/arXiv.2402.10516 PMC13332047 42399444

[2639] Nguyen E., Poli M., Durrant M. G., Kang B., Katrekar D., Li D. B., Bartie L. J., Thomas A. W., King S. H., Brixi G. (2024). Sequence
modeling and
design from molecular to genome scale with Evo. Science.

[2640] Geneious. https://www.geneious.com/ (accessed 12/11/2024).

[2641] Benchling. https://www.benchling.com (accessed 12/11/2024).

[2642] snapgene. www.snapgene.com (accessed 12/11/2024).

[2643] Hillson N. J., Rosengarten R. D., Keasling J. D. (2012). j5 DNA assembly
design automation software. ACS Synth Biol.

[2644] McLaughlin J. A., Myers C. J., Zundel Z., Mısırlı G., Zhang M., Ofiteru I. D., Goñi-Moreno A., Wipat A. (2018). SynBioHub: A Standards-Enabled Design Repository for Synthetic Biology. ACS Synthetic Biology.

[2645] Hoops S., Sahle S., Gauges R., Lee C., Pahle J., Simus N., Singhal M., Xu L., Mendes P., Kummer U. (2006). COPASI-a COmplex PAthway SImulator. Bioinformatics.

[2646] Park J. S., Kim J. R. (2019). Non-compartmental
data analysis using
SimBiology and MATLAB. Transl Clin Pharmacol.

[2647] Funahashi A., Jouraku A., Matsuoka Y., Kitano H. (2007). Integration
of CellDesigner and SABIO-RK. In Silico Biol.

[2648] Vilanova C., Porcar M. (2014). iGEM 2.0-refoundations
for engineering
biology. Nat Biotechnol.

[2649] Smolke C. D. (2009). Building outside of the box: iGEM
and the BioBricks
Foundation. Nat Biotechnol.

[2650] Registry of Standard Biological Parts http://parts.igem.org/ (accessed 12/11/2024).

[2651] Pedreira T., Elfmann C., Stülke J. (2022). The current
state of SubtiWiki, the database for the model organism Bacillus subtilis. Nucleic Acids Research.

[2652] Ishii T., Yoshida K.-i., Terai G., Fujita Y., Nakai K. (2001). DBTBS: a database of Bacillus subtilis
promoters and transcription
factors. Nucleic Acids Research.

[2653] Winsor G. L., Lo R., Sui S. J. H., Ung K. S. E., Huang S., Cheng D., Ching W.-K. H., Hancock R. E. W., Brinkman F. S. L. (2005). Pseudomonas aeruginosa
Genome Database
and PseudoCAP: facilitating community-based, continually updated,
genome annotation. Nucleic Acids Research.

[2654] Zheng W., Tan T. K., Paterson I. C., Mutha N. V. R., Siow C. C., Tan S. Y., Old L. A., Jakubovics N. S., Choo S. W. (2016). StreptoBase: An Oral Streptococcus mitis Group Genomic
Resource and Analysis Platform. PLOS ONE.

[2655] Hucka M., Finney A., Sauro H. M., Bolouri H., Doyle J. C., Kitano H., Arkin A. P., Bornstein B. J., Bray D., Cornish-Bowden A. (2003). The systems biology
markup language (SBML): a medium for representation and exchange of
biochemical network models. Bioinformatics.

[2656] Keating S. M., Waltemath D., Konig M., Zhang F., Drager A., Chaouiya C., Bergmann F. T., Finney A., Gillespie C. S., Helikar T. (2020). SBML Level 3: an extensible
format for the exchange and reuse of biological models. Mol Syst Biol.

[2657] Roehner N., Myers C. J. (2014). A methodology to
annotate systems
biology markup language models with the synthetic biology open language. ACS Synth Biol.

[2658] Terry L., Earl J., Thayer S., Bridge S., Myers C. J. (2021). SBOLCanvas: A Visual Editor for Genetic
Designs. ACS Synth Biol.

[2659] Clark C. J., Scott-Brown J., Gorochowski T. E. (2021). paraSBOLv:
a foundation for standard-compliant genetic design visualization tools. Synth Biol (Oxf).

[2660] Quinn J. Y., Cox R. S., Adler A., Beal J., Bhatia S., Cai Y., Chen J., Clancy K., Galdzicki M., Hillson N. J. (2015). SBOL
Visual: A Graphical Language for Genetic Designs. PLoS Biol.

[2661] Misirli G., Beal J., Gorochowski T. E., Stan G. B., Wipat A., Myers C. J. (2020). SBOL Visual 2 Ontology. ACS Synth Biol.

[2662] Di Salvo M., Pinatel E., Tala A., Fondi M., Peano C., Alifano P. (2018). G4PromFinder: an algorithm
for predicting
transcription promoters in GC-rich bacterial genomes based on AT-rich
elements and G-quadruplex motifs. BMC Bioinformatics.

[2663] Shahmuradov I. A., Mohamad Razali R., Bougouffa S., Radovanovic A., Bajic V. B. (2017). bTSSfinder: a novel
tool for the
prediction of promoters in cyanobacteria and Escherichia coli. Bioinformatics.

[2664] Coelho R. V., Dall'Alba G., de Avila E. S. S., Echeverrigaray S., Delamare A. P. L. (2020). Toward Algorithms
for Automation of Postgenomic Data
Analyses: Bacillus subtilis Promoter Prediction with Artificial Neural
Network. OMICS.

[2665] Coppens L., Lavigne R. (2020). SAPPHIRE: a neural
network based
classifier for sigma70 promoter prediction in Pseudomonas. BMC Bioinformatics.

[2666] Kingsford C. L., Ayanbule K., Salzberg S. L. (2007). Rapid, accurate,
computational discovery of Rho-independent transcription terminators
illuminates their relationship to DNA uptake. Genome Biol.

[2667] Naville M., Ghuillot-Gaudeffroy A., Marchais A., Gautheret D. (2011). ARNold: a
web tool for the prediction of Rho-independent transcription terminators. RNA Biol.

[2668] Gardner P. P., Barquist L., Bateman A., Nawrocki E. P., Weinberg Z. (2011). RNIE: genome-wide prediction of bacterial intrinsic
terminators. Nucleic Acids Res.

[2669] Lammens E. M., Putzeys L., Boon M., Lavigne R. (2023). Sourcing Phage-Encoded
Terminators Using ONT-cappable-seq for SynBio Applications in Pseudomonas. ACS Synth Biol.

[2670] Turner, D. ; Ackermann, H. W. ; Kropinski, A. M. ; Lavigne, R. ; Sutton, J. M. ; Reynolds, D. M. Comparative Analysis of 37 Acinetobacter Bacteriophages. Viruses 2018, 10 (1), 5 10.3390/v10010005. PMC5795418 29295549

[2671] Salis H.
M., Mirsky E. A., Voigt C. A. (2009). Automated design
of synthetic ribosome binding sites to control protein expression. Nature Biotechnology.

[2672] Moore S. J., MacDonald J. T., Wienecke S., Ishwarbhai A., Tsipa A., Aw R., Kylilis N., Bell D. J., McClymont D. W., Jensen K. (2018). Rapid acquisition and
model-based analysis of cell-free transcription-translation reactions
from nonmodel bacteria. Proc Natl Acad Sci U
S A.

[2673] Hossain A., Lopez E., Halper S. M., Cetnar D. P., Reis A. C., Strickland D., Klavins E., Salis H. M. (2020). Automated
design of thousands of nonrepetitive parts for engineering stable
genetic systems. Nature Biotechnology.

[2674] Cetnar D. P., Salis H. M. (2021). Systematic Quantification of Sequence
and Structural Determinants Controlling mRNA stability in Bacterial
Operons. ACS Synthetic Biology.

[2675] Athey J., Alexaki A., Osipova E., Rostovtsev A., Santana-Quintero L. V., Katneni U., Simonyan V., Kimchi-Sarfaty C. (2017). A new and
updated resource for codon usage tables. BMC
Bioinformatics.

[2676] Atum. https://www.atum.bio/resources/tools/gene-designer (accessed 12/11/2024.

[2677] JCAT. http://www.jcat.de (accessed 12/11/2024.

[2678] GeneArt. https://www.thermofisher.com/us/en/home/life-science/cloning/gene-synthesis/geneart-gene-synthesis.html (accessed 12/11/2024).

[2679] Salvachua D., Rydzak T., Auwae R., De Capite A., Black B. A., Bouvier J. T., Cleveland N. S., Elmore J. R., Furches A., Huenemann J. D. (2020). Metabolic engineering of Pseudomonas putida for increased polyhydroxyalkanoate
production from lignin. Microb Biotechnol.

[2680] Liu S., Wang M., Du G., Chen J. (2016). Improving the active
expression of transglutaminase in Streptomyces lividans by promoter
engineering and codon optimization. BMC Biotechnology.

[2681] Liu G., Zhang Y., Zhang T. (2020). Computational
approaches for effective
CRISPR guide RNA design and evaluation. Comput
Struct Biotechnol J.

[2682] Labun K., Montague T. G., Krause M., Torres Cleuren Y. N., Tjeldnes H., Valen E. (2019). CHOPCHOP v3: expanding the CRISPR
web toolbox beyond genome editing. Nucleic Acids
Res.

[2683] Moreno-Mateos M. A., Vejnar C. E., Beaudoin J. D., Fernandez J. P., Mis E. K., Khokha M. K., Giraldez A. J. (2015). CRISPRscan: designing
highly efficient sgRNAs for CRISPR-Cas9 targeting in vivo. Nat Methods.

[2684] Chuai G. H., Wang Q. L., Liu Q. (2017). In Silico
Meets In
Vivo: Towards Computational CRISPR-Based sgRNA Design. Trends Biotechnol.

[2685] Heigwer F., Zhan T., Breinig M., Winter J., Brugemann D., Leible S., Boutros M. (2016). CRISPR library
designer
(CLD): software for multispecies design of single guide RNA libraries. Genome Biol.

[2686] Liu H., Wei Z., Dominguez A., Li Y., Wang X., Qi L. S. (2015). CRISPR-ERA: a comprehensive design
tool for CRISPR-mediated gene
editing, repression and activation. Bioinformatics.

[2687] Nyerges Á., Csörgő B., Nagy I., Bálint B., Bihari P., Lázár V., Apjok G., Umenhoffer K., Bogos B., Pósfai G. (2016). A highly precise and
portable genome engineering method allows comparison
of mutational effects across bacterial species. Proceedings of the National Academy of Sciences.

[2688] Wannier T. M., Nyerges A., Kuchwara H. M., Czikkely M., Balogh D., Filsinger G. T., Borders N. C., Gregg C. J., Lajoie M. J., Rios X. (2020). Improved bacterial recombineering
by parallelized protein discovery. Proceedings
of the National Academy of Sciences.

[2689] Bonde M. T., Klausen M. S., Anderson M. V., Wallin A. I., Wang H. H., Sommer M. O. (2014). MODEST: a web-based
design tool for
oligonucleotide-mediated genome engineering and recombineering. Nucleic Acids Res.

[2690] Bordbar A., Monk J. M., King Z. A., Palsson B. O. (2014). Constraint-based
models predict metabolic and associated cellular functions. Nat Rev Genet.

[2691] Volk M. J., Tran V. G., Tan S. I., Mishra S., Fatma Z., Boob A., Li H., Xue P., Martin T. A., Zhao H. (2023). Metabolic Engineering: Methodologies
and Applications. Chem Rev.

[2692] Gu C., Kim G. B., Kim W. J., Kim H. U., Lee S. Y. (2019). Current
status and applications of genome-scale metabolic models. Genome Biol.

[2693] Norsigian C. J., Pusarla N., McConn J. L., Yurkovich J. T., Drager A., Palsson B. O., King Z. (2019). BiGG Models 2020: multi-strain
genome-scale models and expansion across the phylogenetic tree. Nucleic Acids Res.

[2694] Seaver S. M. D., Liu F., Zhang Q., Jeffryes J., Faria J. P., Edirisinghe J. N., Mundy M., Chia N., Noor E., Beber M. E. (2021). The ModelSEED Biochemistry
Database for the integration of metabolic annotations and the reconstruction,
comparison and analysis of metabolic models for plants, fungi and
microbes. Nucleic Acids Res.

[2695] Dias O., Rocha M., Ferreira E. C., Rocha I. (2015). Reconstructing
genome-scale metabolic models with merlin. Nucleic
Acids Res.

[2696] Agren R., Liu L., Shoaie S., Vongsangnak W., Nookaew I., Nielsen J. (2013). The RAVEN
toolbox and its use for
generating a genome-scale metabolic model for Penicillium chrysogenum. PLoS Comput Biol.

[2697] Karp P. D., Paley S., Romero P. (2002). The Pathway
Tools software. Bioinformatics.

[2698] Swainston N., Smallbone K., Mendes P., Kell D., Paton N. (2011). The SuBliMinaL
Toolbox: automating steps in the reconstruction of
metabolic networks. J Integr Bioinform.

[2699] Machado D., Andrejev S., Tramontano M., Patil K. R. (2018). Fast automated reconstruction of genome-scale metabolic
models for microbial species and communities. Nucleic Acids Res.

[2700] Henry C. S., DeJongh M., Best A. A., Frybarger P. M., Linsay B., Stevens R. L. (2010). High-throughput
generation, optimization
and analysis of genome-scale metabolic models. Nat Biotechnol.

[2701] Lieven C., Beber M. E., Olivier B. G., Bergmann F. T., Ataman M., Babaei P., Bartell J. A., Blank L. M., Chauhan S., Correia K. (2020). MEMOTE for standardized
genome-scale metabolic model testing. Nat Biotechnol.

[2702] Castillo-Alfonso, F. ; Quintana-Menendez, A. ; Vigueras-Ramirez, G. ; Sales-Cruz, A. M. ; Rosales-Colunga, L. M. ; Olivares-Hernandez, R. Analysis of the Propionate Metabolism in Bacillus subtilis during 3-Indolacetic Production. Microorganisms 2022, 10 (12), 2352. 10.3390/microorganisms10122352. 36557605 PMC9782769

[2703] Yang L., Cluett W. R., Mahadevan R. (2011). EMILiO: a
fast algorithm for genome-scale strain design. Metab Eng.

[2704] Kim J., Reed J. L., Maravelias C. T. (2011). Large-scale
bi-level strain design
approaches and mixed-integer programming solution techniques. PLoS One.

[2705] Chowdhury A., Zomorrodi A. R., Maranas C. D. (2014). k-OptForce: integrating
kinetics with flux balance analysis for strain design. PLoS Comput Biol.

[2706] Nogales J., Palsson B. O., Thiele I. (2008). A genome-scale
metabolic
reconstruction of Pseudomonas putida KT2440: iJN746 as a cell factory. BMC Syst Biol.

[2707] Saifuddin M., Bhatnagar J. M., Segre D., Finzi A. C. (2019). Microbial
carbon use efficiency predicted from genome-scale metabolic models. Nat Commun.

[2708] Schulte C. C. M., Ramachandran V. K., Papachristodoulou A., Poole P. S. (2022). Genome-Scale Metabolic Modelling
of Lifestyle Changes
in Rhizobium leguminosarum. mSystems.

[2709] Hatzimanikatis V., Li C., Ionita J. A., Henry C. S., Jankowski M. D., Broadbelt L. J. (2005). Exploring
the diversity of complex
metabolic networks. Bioinformatics.

[2710] Rodrigo G., Carrera J., Prather K. J., Jaramillo A. (2008). DESHARKY:
automatic design of metabolic pathways for optimal cell growth. Bioinformatics.

[2711] Campodonico M. A., Andrews B. A., Asenjo J. A., Palsson B. O., Feist A. M. (2014). Generation of an atlas for commodity
chemical production
in Escherichia coli and a novel pathway prediction algorithm, GEM-Path. Metab Eng.

[2712] Carbonell P., Parutto P., Baudier C., Junot C., Faulon J. L. (2014). Retropath: automated pipeline for embedded metabolic
circuits. ACS Synth Biol.

[2713] Finnigan W., Hepworth L. J., Flitsch S. L., Turner N. J. (2021). RetroBioCat
as a computer-aided synthesis planning tool for biocatalytic reactions
and cascades. Nat Catal.

[2714] Oh Y. K., Palsson B. O., Park S. M., Schilling C. H., Mahadevan R. (2007). Genome-scale reconstruction of metabolic
network in
Bacillus subtilis based on high-throughput phenotyping and gene essentiality
data. J Biol Chem.

[2715] Henry C. S., Zinner J. F., Cohoon M. P., Stevens R. L. (2009). iBsu1103:
a new genome-scale metabolic model of Bacillus subtilis based on SEED
annotations. Genome Biol.

[2716] Hao T., Han B., Ma H., Fu J., Wang H., Wang Z., Tang B., Chen T., Zhao X. (2013). In silico
metabolic engineering of Bacillus subtilis for improved production
of riboflavin, Egl-237, (R,R)-2,3-butanediol and isobutanol. Mol Biosyst.

[2717] Kocabas P., Calik P., Calik G., Ozdamar T. H. (2009). Microarray
Studies in Bacillus subtilis. Biotechnol J.

[2718] Blazquez, B. ; San Leon, D. ; Rojas, A. ; Tortajada, M. ; Nogales, J. New Insights on Metabolic Features of Bacillus subtilis Based on Multistrain Genome-Scale Metabolic Modeling. Int J Mol Sci 2023, 24 (8), 7091 10.3390/ijms24087091. 37108252 PMC10138676

[2719] Aminian-Dehkordi J., Mousavi S. M., Jafari A., Mijakovic I., Marashi S. A. (2019). Manually curated genome-scale reconstruction of the
metabolic network of Bacillus megaterium DSM319. Sci Rep.

[2720] Zou W., Zhou M., Liu L., Chen J. (2013). Reconstruction and
analysis of the industrial strain Bacillus megaterium WSH002 genome-scale
in silico metabolic model. J Biotechnol.

[2721] Guo J., Zhang H., Wang C., Chang J. W., Chen L. L. (2016). Construction
and analysis of a genome-scale metabolic network for Bacillus licheniformis
WX-02. Res Microbiol.

[2722] Puchalka J., Oberhardt M. A., Godinho M., Bielecka A., Regenhardt D., Timmis K. N., Papin J. A., Martins
dos Santos V. A. (2008). Genome-scale reconstruction and analysis of the Pseudomonas
putida KT2440 metabolic network facilitates applications in biotechnology. PLoS Comput Biol.

[2723] Sohn S. B., Kim T. Y., Park J. M., Lee S. Y. (2010). In silico
genome-scale metabolic analysis of Pseudomonas putida KT2440 for polyhydroxyalkanoate
synthesis, degradation of aromatics and anaerobic survival. Biotechnol J.

[2724] Yuan Q., Huang T., Li P., Hao T., Li F., Ma H., Wang Z., Zhao X., Chen T., Goryanin I. (2017). Pathway-Consensus Approach to Metabolic Network Reconstruction
for Pseudomonas putida KT2440 by Systematic Comparison of Published
Models. PLoS One.

[2725] Nogales J., Mueller J., Gudmundsson S., Canalejo F. J., Duque E., Monk J., Feist A. M., Ramos J. L., Niu W., Palsson B. O. (2020). High-quality genome-scale
metabolic modelling of Pseudomonas putida highlights its broad metabolic
capabilities. Environ Microbiol.

[2726] Huang X., Lin Y. H. (2020). Reconstruction and
analysis of a
three-compartment genome-scale metabolic model for Pseudomonas fluorescens. Biotechnol Appl Biochem.

[2727] Babaei P., Marashi S. A., Asad S. (2015). Genome-scale
reconstruction
of the metabolic network in Pseudomonas stutzeri A1501. Mol Biosyst.

[2728] Oberhardt M. A., Puchalka J., Fryer K. E., Martins
dos Santos V. A., Papin J. A. (2008). Genome-scale metabolic network analysis
of the opportunistic pathogen Pseudomonas aeruginosa PAO1. J Bacteriol.

[2729] Ofaim S., Zarecki R., Porob S., Gat D., Lahav T., Kashi Y., Aly R., Eizenberg H., Ronen Z., Freilich S. (2020). Genome-scale reconstruction of Paenarthrobacter
aurescens TC1 metabolic model towards the study of atrazine bioremediation. Sci Rep.

[2730] Resendis-Antonio O., Reed J. L., Encarnacion S., Collado-Vides J., Palsson B. O. (2007). Metabolic reconstruction and modeling
of nitrogen fixation in Rhizobium etli. PLoS
Comput Biol.

[2731] Resendis-Antonio O., Hernandez M., Salazar E., Contreras S., Batallar G. M., Mora Y., Encarnacion S. (2011). Systems biology
of bacterial nitrogen fixation: high-throughput technology and its
integrative description with constraint-based modeling. BMC Syst Biol.

[2732] Zhao H., Li M., Fang K., Chen W., Wang J. (2012). In silico insights into the symbiotic
nitrogen fixation in Sinorhizobium
meliloti via metabolic reconstruction. PLoS
One.

[2733] Yang Y., Hu X. P., Ma B. G. (2017). Construction and
simulation of the Bradyrhizobium diazoefficiens USDA110 metabolic
network: a comparison between free-living and symbiotic states. Mol Biosyst.

[2734] Xu N., Yang Q., Yang X., Wang M., Guo M. (2021). Reconstruction
and analysis of a genome-scale metabolic model for Agrobacterium tumefaciens. Mol Plant Pathol.

[2735] Thomas G. H., Zucker J., Macdonald S. J., Sorokin A., Goryanin I., Douglas A. E. (2009). A fragile metabolic
network adapted for cooperation in the symbiotic bacterium Buchnera
aphidicola. BMC Syst Biol.

[2736] Liao Y. C., Huang T. W., Chen F. C., Charusanti P., Hong J. S., Chang H. Y., Tsai S. F., Palsson B. O., Hsiung C. A. (2011). An experimentally validated genome-scale metabolic
reconstruction of Klebsiella pneumoniae MGH 78578, iYL1228. J Bacteriol.

[2737] Park J. M., Song H., Lee H. J., Seung D. (2013). Genome-scale
reconstruction and in silico analysis of Klebsiella oxytoca for 2,3-butanediol
production. Microb Cell Fact.

[2738] Peyraud R., Cottret L., Marmiesse L., Gouzy J., Genin S. (2016). A Resource Allocation Trade-Off between
Virulence and Proliferation Drives Metabolic Versatility in the Plant
Pathogen Ralstonia solanacearum. PLoS Pathog.

[2739] Klanchui A., Khannapho C., Phodee A., Cheevadhanarak S., Meechai A. (2012). iAK692: a genome-scale metabolic model of Spirulina
platensis C1. BMC Syst Biol.

[2740] Koduru L., Kim H. Y., Lakshmanan M., Mohanty B., Lee Y. Q., Lee C. H., Lee D. Y. (2020). Genome-scale
metabolic reconstruction and in silico analysis of the rice leaf blight
pathogen, Xanthomonas oryzae. Mol Plant Pathol.

[2741] Schatschneider S., Persicke M., Watt S. A., Hublik G., Puhler A., Niehaus K., Vorholter F. J. (2013). Establishment,
in silico analysis, and experimental verification of a large-scale
metabolic network of the xanthan producing Xanthomonas campestris
pv. campestris strain B100. J Biotechnol.

[2742] Botero D., Monk J., Rodriguez Cubillos M.
J., Rodriguez
Cubillos A., Restrepo M., Bernal-Galeano V., Reyes A., Gonzalez Barrios A., Palsson B. O., Restrepo S. (2020). Genome-Scale Metabolic Model of Xanthomonas phaseoli pv. manihotis:
An Approach to Elucidate Pathogenicity at the Metabolic Level. Front Genet.

[2743] Hassani M. A., Duran P., Hacquard S. (2018). Microbial
interactions
within the plant holobiont. Microbiome.

[2744] Tisue, S. ; Wilensky, U. Netlogo: A simple environment for modeling complexity. In International conference on complex systems; 2004; Citeseer: Vol. 21, pp 16-21.

[2745] Holcombe M., Adra S., Bicak M., Chin S., Coakley S., Graham A. I., Green J., Greenough C., Jackson D., Kiran M. (2012). Modelling complex biological
systems using an agent-based approach. Integrative
Biology.

[2746] North M. J., Collier N. T., Ozik J., Tatara E. R., Macal C. M., Bragen M., Sydelko P. (2013). Complex adaptive systems
modeling with Repast Simphony. Complex adaptive
systems modeling.

[2747] Banitz T., Gras A., Ginovart M. (2015). Individual-based modeling
of soil organic matter in NetLogo: Transparent, user-friendly, and
open. Environmental Modelling & Software.

[2748] Rubio Fernandez D., Barreto-Hernández E. (2022). Simulating
Microbial
Functional Diversity Dynamics in Agricultural Soils: An Individual
Based Modeling Approach. Advances in Bioscience
and Biotechnology.

[2749] Gorochowski T. E. (2016). Agent-based modelling in synthetic
biology. Essays Biochem.

[2750] Kreft J.-U., Picioreanu C., Wimpenny J. W. T., van
Loosdrecht M. C. M. (2001). Individual-based modelling of biofilms. Microbiology.

[2751] Kreft J.-U., Booth G., Wimpenny J. W. T. (1998). BacSim,
a simulator
for individual-based modelling of bacterial colony growth. Microbiology.

[2752] Gorochowski T. E., Matyjaszkiewicz A., Todd T., Oak N., Kowalska K., Reid S., Tsaneva-Atanasova K. T., Savery N. J., Grierson C. S., di Bernardo M. (2012). BSim: An Agent-Based
Tool for Modeling Bacterial Populations in Systems and Synthetic Biology. PLOS ONE.

[2753] Matyjaszkiewicz A., Fiore G., Annunziata F., Grierson C. S., Savery N. J., Marucci L., di Bernardo M. (2017). BSim 2.0:
An Advanced Agent-Based Cell Simulator. ACS
Synthetic Biology.

[2754] Jang S. S., Oishi K. T., Egbert R. G., Klavins E. (2012). Specification
and Simulation of Synthetic Multicelled Behaviors. ACS Synthetic Biology.

[2755] Gutiérrez M., Gregorio-Godoy P., Pérez del Pulgar G., Muñoz L. E., Sáez S., Rodríguez-Patón A. (2017). A New Improved
and Extended Version of the Multicell Bacterial Simulator gro. ACS Synthetic Biology.

[2756] Wei G., Bogdan P., Marculescu R. (2013). Efficient
Modeling and Simulation
of Bacteria-Based Nanonetworks with BNSim. IEEE
Journal on Selected Areas in Communications.

[2757] Muci A. L., Jorquera M. A., Ávila Á. I., Rengel Z., Crowley D. E., de la Luz Mora M. (2012). A combination
of cellular automata and agent-based models for simulating the root
surface colonization by bacteria. Ecological
Modelling.

[2758] Bauer E., Thiele I. (2018). From Network Analysis to Functional
Metabolic Modeling of the Human Gut Microbiota. mSystems.

[2759] Heinken A., Basile A., Thiele I. (2021). Advances in constraint-based
modelling of microbial communities. Current
Opinion in Systems Biology.

[2760] Pfau T., Christian N., Masakapalli S. K., Sweetlove L. J., Poolman M. G., Ebenhöh O. (2018). The intertwined
metabolism during symbiotic nitrogen fixation elucidated by metabolic
modelling. Scientific Reports.

[2761] Gomes de Oliveira Dal'Molin, C. ; Quek, L.-E. ; Saa, P. A. ; Nielsen, L. K. A multi-tissue genome-scale metabolic modeling framework for the analysis of whole plant systems. Frontiers in Plant Science 2015, 6, 10.3389/fpls.2015.00004. PMC4302846 25657653

[2762] diCenzo G. C., Tesi M., Pfau T., Mengoni A., Fondi M. (2020). Genome-scale
metabolic reconstruction of the symbiosis between a
leguminous plant and a nitrogen-fixing bacterium. Nature Communications.

[2763] Xu X., Zarecki R., Medina S., Ofaim S., Liu X., Chen C., Hu S., Brom D., Gat D., Porob S. (2019). Modeling microbial communities from atrazine contaminated
soils promotes the development of biostimulation solutions. The ISME Journal.

[2764] Zhuang K., Ma E., Lovley D. R., Mahadevan R. (2012). The design
of long-term effective uranium bioremediation strategy using a community
metabolic model. Biotechnology and Bioengineering.

[2765] Islam M. M., Le T., Daggumati S. R., Saha R. (2020). Investigation of microbial community
interactions between Lake Washington
methanotrophs using genome-scale metabolic modeling. PeerJ.

[2766] Tobalina L., Bargiela R., Pey J., Herbst F.-A., Lores I., Rojo D., Barbas C., Peláez A. I., Sánchez J., von Bergen M. (2015). Context-specific metabolic
network reconstruction of a naphthalene-degrading bacterial community
guided by metaproteomic data. Bioinformatics.

[2767] Karkaria B. D., Fedorec A. J. H., Barnes C. P. (2021). Automated design
of synthetic microbial communities. Nat Commun.

[2768] Maheshwari, D. K. ; Das, A. ; Dheeman, S. ; Pandey, P. An Overall Insight Into the Attributes, Interactions, and Future Applications of “Microbial Consortium” for Plant Growth Promotion with Contemporary Approaches. In Sustainable Agrobiology: Design and Development of Microbial Consortia; Maheshwari, D. K. , Dheeman, S. Eds.; Springer Nature Singapore, 2023; pp 3-22.

[2769] Harfouche A. L., Jacobson D. A., Kainer D., Romero J. C., Harfouche A. H., Scarascia Mugnozza G., Moshelion M., Tuskan G. A., Keurentjes J. J. B., Altman A. (2019). Accelerating Climate
Resilient Plant Breeding by Applying Next-Generation Artificial Intelligence. Trends in Biotechnology.

[2770] Zhou L., Zhang C., Liu F., Qiu Z., He Y. (2019). Application of Deep Learning in Food: A Review. Comprehensive Reviews in Food Science and Food Safety.

[2771] Kamilaris A., Prenafeta-Boldú F. X. (2018). Deep learning in
agriculture: A survey. Computers and Electronics
in Agriculture.

[2772] Song P., Wang J., Guo X., Yang W., Zhao C. (2021). High-throughput phenotyping: Breaking through the bottleneck in future
crop breeding. The Crop Journal.

[2773] Vorholt J. A., Vogel C., Carlström C. I., Müller D. B. (2017). Establishing Causality: Opportunities of Synthetic
Communities for Plant Microbiome Research. Cell
Host & Microbe.

[2774] Finkel O. M., Castrillo G., Herrera Paredes S., Salas González I., Dangl J. L. (2017). Understanding and
exploiting plant beneficial microbes. Current
Opinion in Plant Biology.

[2775] de Souza, R. S. C. ; Armanhi, J. S. L. ; Arruda, P. From Microbiome to Traits: Designing Synthetic Microbial Communities for Improved Crop Resiliency. Frontiers in Plant Science 2020, 11, 10.3389/fpls.2020.01179. PMC7484511 32983187

[2776] Zhao, L. ; Walkowiak, S. ; Fernando, W. G. Artificial Intelligence: A Promising Tool in Exploring the Phytomicrobiome in Managing Disease and Promoting Plant Health. Plants 2023 12, 10.3390/plants12091852. PMC10180744 37176910

[2777] Herrera Paredes S., Gao T., Law T. F., Finkel O. M., Mucyn T., Teixeira P. J. P. L., Salas González I., Feltcher M. E., Powers M. J., Shank E. A. (2018). Design
of synthetic bacterial communities for predictable plant phenotypes. PLOS Biology.

[2778] Doedel, E. Auto: a program for the automatic bifurcation analysis of autonomous systems. Congressus Numerantium 1981, 30.

[2779] O'Brien E. L., Itallie E. V., Bennett M. R. (2012). Modeling
synthetic
gene oscillators. Math Biosci.

[2780] Qian Y., Huang H. H., Jimenez J. I., Del Vecchio D. (2017). Resource Competition
Shapes the Response of Genetic Circuits. ACS
Synth Biol.

[2781] Hu C. Y., Varner J. D., Lucks J. B. (2015). Generating Effective
Models and Parameters for RNA Genetic Circuits. ACS Synth Biol.

[2782] Tas H., Grozinger L., Goni-Moreno A., de Lorenzo V. (2021). Automated
design and implementation of a NOR gate in Pseudomonas putida. Synth Biol (Oxf).

[2783] Wu Y., Chen T., Liu Y., Tian R., Lv X., Li J., Du G., Chen J., Ledesma-Amaro R., Liu L. (2020). Design of a programmable biosensor-CRISPRi genetic circuits for dynamic
and autonomous dual-control of metabolic flux in Bacillus subtilis. Nucleic Acids Res.

[2784] Misirli G., Nguyen T., McLaughlin J. A., Vaidyanathan P., Jones T. S., Densmore D., Myers C., Wipat A. (2019). A Computational Workflow for the Automated Generation of Models of
Genetic Designs. ACS Synth Biol.

[2785] Lux M. W., Bramlett B. W., Ball D. A., Peccoud J. (2012). Genetic design
automation: engineering fantasy or scientific renewal?. Trends Biotechnol.

[2786] Buecherl L., Myers C. J. (2022). Engineering genetic
circuits: advancements
in genetic design automation tools and standards for synthetic biology. Curr Opin Microbiol.

[2787] Appleton, E. ; Madsen, C. ; Roehner, N. ; Densmore, D. Design Automation in Synthetic Biology. Cold Spring Harb Perspect Biol 2017, 9 (4), a023978 10.1101/cshperspect.a023978. 28246188 PMC5378053

[2788] HamediRad M., Chao R., Weisberg S., Lian J., Sinha S., Zhao H. (2019). Towards a fully automated algorithm
driven platform for biosystems design. Nat Commun.

[2789] Asmiov Labs. https://blog.asimov.com/blog-post/asimov-labs (accessed 12/11/24).

[2790] Serber, Z. ; Dean, E. J. ; Manchester, S. ; Gora, K. ; Flashman, M. ; Shellman, E. ; Kimball, A. ; Szyjka, S. ; Frewen, B. ; Treynor, T. Automated system for HTP genomic engineering. US20200291392A1, 2020.

[2791] Wang L., Zang X., Zhou J. (2022). Synthetic biology:
A powerful booster for future agriculture. Advanced
Agrochem.

[2792] Liu Y., Su A., Li J., Ledesma-Amaro R., Xu P., Du G., Liu L. (2020). Towards next-generation model microorganism
chassis for biomanufacturing. Appl Microbiol
Biotechnol.

[2793] Chao R., Liang J., Tasan I., Si T., Ju L., Zhao H. (2017). Fully Automated One-Step Synthesis of Single-Transcript
TALEN Pairs Using a Biological Foundry. ACS
Synth Biol.

[2794] Dunstan M. S., Robinson C. J., Jervis A. J., Yan C., Carbonell P., Hollywood K. A., Currin A., Swainston N., Feuvre R. L., Micklefield J. (2020). Engineering Escherichia
coli towards de novo production of gatekeeper (2S)-flavanones: naringenin,
pinocembrin, eriodictyol and homoeriodictyol. Synth Biol (Oxf).

[2795] Zournas A., Incha M. R., Radivojevic T., Blay V., Martí J. M., Costello Z., Schimdt M., Chung T., Thompson M. G., Pearson A. (2025). Machine learning-led
semi-automated medium optimization reveals salt as key for flaviolin
production in Pseudomonas putida. Communications
Biology.

[2796] Silverman A. D., Akova U., Alam K. K., Jewett M. C., Lucks J. B. (2020). Design
and Optimization of a Cell-Free Atrazine Biosensor. ACS Synth Biol.

[2797] Jung J. K., Alam K. K., Verosloff M. S., Capdevila D. A., Desmau M., Clauer P. R., Lee J. W., Nguyen P. Q., Pasten P. A., Matiasek S. J. (2020). Cell-free
biosensors for rapid detection of water contaminants. Nat Biotechnol.

[2798] Thavarajah W., Silverman A. D., Verosloff M. S., Kelley-Loughnane N., Jewett M. C., Lucks J. B. (2020). Point-of-Use
Detection
of Environmental Fluoride via a Cell-Free Riboswitch-Based Biosensor. ACS Synth Biol.

[2799] Karim A. S., Jewett M. C. (2016). A cell-free framework
for rapid biosynthetic
pathway prototyping and enzyme discovery. Metab
Eng.

[2800] Boles K. S., Kannan K., Gill J., Felderman M., Gouvis H., Hubby B., Kamrud K. I., Venter J. C., Gibson D. G. (2017). Digital-to-biological converter for on-demand production
of biologics. Nat Biotechnol.

[2801] Zhang Y., Minagawa Y., Kizoe H., Miyazaki K., Iino R., Ueno H., Tabata K. V., Shimane Y., Noji H. (2019). Accurate high-throughput screening
based on digital protein synthesis
in a massively parallel femtoliter droplet array. Sci Adv.

[2802] Chappell J., Jensen K., Freemont P. S. (2013). Validation of an
entirely in vitro approach for rapid prototyping of DNA regulatory
elements for synthetic biology. Nucleic Acids
Res.

[2803] Kelwick R., Webb A. J., MacDonald J. T., Freemont P. S. (2016). Development of a
Bacillus subtilis cell-free transcription-translation
system for prototyping regulatory elements. Metab Eng.

[2804] Yim S. S., Johns N. I., Park J., Gomes A. L., McBee R. M., Richardson M., Ronda C., Chen S. P., Garenne D., Noireaux V. (2019). Multiplex transcriptional
characterizations across diverse bacterial species using cell-free
systems. Mol Syst Biol.

[2805] Yang, C. ; Yang, M. ; Zhao, W. ; Ding, Y. ; Wang, Y. ; Li, J. Establishing a Klebsiella pneumoniae-Based Cell-Free Protein Synthesis System. Molecules 2022, 27 (15), 4684 10.3390/molecules27154684. 35897861 PMC9330377

[2806] Li J., Wang H., Kwon Y. C., Jewett M. C. (2017). Establishing a high
yielding streptomyces-based cell-free protein synthesis system. Biotechnol Bioeng.

[2807] Noireaux V., Bar-Ziv R., Libchaber A. (2003). Principles
of cell-free genetic circuit assembly. Proc
Natl Acad Sci U S A.

[2808] Takahashi M. K., Chappell J., Hayes C. A., Sun Z. Z., Kim J., Singhal V., Spring K. J., Al-Khabouri S., Fall C. P., Noireaux V. (2015). Rapidly
characterizing
the fast dynamics of RNA genetic circuitry with cell-free transcription-translation
(TX-TL) systems. ACS Synth Biol.

[2809] Hu C. Y., Takahashi M. K., Zhang Y., Lucks J. B. (2018). Engineering
a Functional Small RNA Negative Autoregulation Network with Model-Guided
Design. ACS Synth Biol.

[2810] Westbrook A., Tang X., Marshall R., Maxwell C. S., Chappell J., Agrawal D. K., Dunlop M. J., Noireaux V., Beisel C. L., Lucks J. (2019). Distinct
timescales
of RNA regulators enable the construction of a genetic pulse generator. Biotechnol Bioeng.

[2811] Jewett M. C., Swartz J. R. (2004). Mimicking the Escherichia
coli cytoplasmic
environment activates long-lived and efficient cell-free protein synthesis. Biotechnol Bioeng.

[2812] Jewett M. C., Calhoun K. A., Voloshin A., Wuu J. J., Swartz J. R. (2008). An integrated cell-free metabolic
platform for protein
production and synthetic biology. Mol Syst Biol.

[2813] Moore S. J., Lai H. E., Chee S. M., Toh M., Coode S., Chengan K., Capel P., Corre C., de Los Santos E. L., Freemont P. S. (2021). A Streptomyces venezuelae Cell-Free
Toolkit for Synthetic Biology. ACS Synth Biol.

[2814] Kightlinger W., Duncker K. E., Ramesh A., Thames A. H., Natarajan A., Stark J. C., Yang A., Lin L., Mrksich M., DeLisa M. P. (2019). A cell-free biosynthesis
platform for modular construction of protein glycosylation pathways. Nat Commun.

[2815] Faulon J. L., Faure L. (2021). In silico, in vitro, and in vivo
machine learning in synthetic biology and metabolic engineering. Curr Opin Chem Biol.

[2816] Presnell K. V., Alper H. S. (2019). Systems Metabolic Engineering Meets
Machine Learning: A New Era for Data-Driven Metabolic Engineering. Biotechnol J.

[2817] Rai K., Wang Y., O’Connell R. W., Patel A. B., Bashor C. J. (2024). Using machine
learning to enhance and accelerate synthetic biology. Current Opinion in Biomedical Engineering.

[2818] Kong D., Qian J., Gao C., Wang Y., Shi T., Ye C. (2025). Machine Learning Empowering Microbial Cell Factory:
A Comprehensive Review. Appl Biochem Biotechnol.

[2819] Yan Z., Chu W., Sheng Y., Tang K., Wang S., Liu Y., Wong W. F. (2024). Integrating
Deep Learning and Synthetic Biology: A
Co-Design Approach for Enhancing Gene Expression via N-Terminal Coding
Sequences. ACS Synth Biol.

[2820] Albornoz R. V., Oyarzun D., Burgess K. (2024). Optimisation
of surfactin
yield in Bacillus using data-efficient active learning and high-throughput
mass spectrometry. Comput Struct Biotechnol
J.

[2821] Carruthers, D. N. ; Kinnunen, P. C. ; Li, Y. ; Chen, Y. ; Gin, J. W. ; Yunus, I. S. ; Galliard, W. R. ; Tan, S. ; Adams, P. D. ; Singh, A. K. Automation and machine learning drive rapid optimization of isoprenol production in Pseudomonas putida. 2025. 10.1038/s41467-025-66304-8 PMC12748988 41390487

[2822] Alori, E. T. ; Emmanuel, O. C. ; Glick, B. R. ; Babalola, O. O. Plant-archaea relationships: a potential means to improve crop production in arid and semi-arid regions. World J Microb Biot 2020, 36 (9), ARTN 133. 10.1007/s11274-020-02910-6 32772189

[2823] Leininger S., Urich T., Schloter M., Schwark L., Qi J., Nicol G. W., Prosser J. I., Schuster S. C., Schleper C. (2006). Archaea predominate
among ammonia-oxidizing prokaryotes in soils. Nature.

[2824] Ouyang Y., Norton J. M., Stark J. M. (2017). Ammonium availability
and temperature control contributions of ammonia oxidizing bacteria
and archaea to nitrification in an agricultural soil. Soil Biology & Biochemistry.

[2825] Yadav, A. N. ; Sharma, D. ; Gulati, S. ; Singh, S. ; Dey, R. ; Pal, K. K. ; Kaushik, R. ; Saxena, A. K. Haloarchaea Endowed with Phosphorus Solubilization Attribute Implicated in Phosphorus Cycle. Scientific Reports 2015, 5, ARTN 12293. 10.1038/srep12293 PMC4516986 26216440

[2826] Cavicchioli R., Curmi P. M., Saunders N., Thomas T. (2003). Pathogenic
archaea: do they exist?. Bioessays.

[2827] Thevasundaram K., Gallagher J. J., Cherng F., Chang M. C. Y. (2022). Engineering
nonphotosynthetic carbon fixation for production of bioplastics by
methanogenic archaea. Proc Natl Acad Sci U S
A.

[2828] Lyu Z., Jain R., Smith P., Fetchko T., Yan Y., Whitman W. B. (2016). Engineering the Autotroph Methanococcus maripaludis
for Geraniol Production. ACS Synth Biol.

[2829] Zuo, Z. Q. ; Xue, Q. ; Zhou, J. ; Zhao, D. H. ; Han, J. ; Xiang, H. Engineering Haloferax mediterranei as an Efficient Platform for High Level Production of Lycopene. Frontiers in Microbiology 2018, 9, ARTN 2893. 10.3389/fmicb.2018.02893 PMC6282799 30555438

[2830] Born J., Weitzel K., Suess B., Pfeifer F. (2021). A Synthetic
Riboswitch to Regulate Haloarchaeal Gene Expression. Front Microbiol.

[2831] Erkel C., Kube M., Reinhardt R., Liesack W. (2006). Genome of Rice Cluster
I archaea-the key methane producers
in the rice rhizosphere. Science.

[2832] Tapio I., Snelling T. J., Strozzi F., Wallace R. J. (2017). The ruminal
microbiome associated with methane emissions from ruminant livestock. J Anim Sci Biotechnol.

[2833] Jiang Y., Qian H., Wang L., Feng J., Huang S., Hungate B. A., van Kessel C., Horwath W. R., Zhang X., Qin X. (2019). Limited
potential of harvest index improvement to reduce methane emissions
from rice paddies. Glob Chang Biol.

[2834] Pitta D. W., Indugu N., Melgar A., Hristov A., Challa K., Vecchiarelli B., Hennessy M., Narayan K., Duval S., Kindermann M. (2022). The effect of 3-nitrooxypropanol,
a potent methane inhibitor, on ruminal microbial gene expression profiles
in dairy cows. Microbiome.

[2835] Hristov A. N., Oh J., Giallongo F., Frederick T. W., Harper M. T., Weeks H. L., Branco A. F., Moate P. J., Deighton M. H., Williams S. R. (2015). An
inhibitor persistently decreased enteric methane emission from dairy
cows with no negative effect on milk production. Proc Natl Acad Sci U S A.

[2836] White R. H. (1987). Indole-3-Acetic-Acid and 2-(Indol-3-Ylmethyl)Indol-3-Yl
Acetic-Acid in the Thermophilic Archaebacterium Sulfolobus-Acidocaldarius. Journal of Bacteriology.

[2837] Taffner, J. ; Erlacher, A. ; Bragina, A. ; Berg, C. ; Moissl-Eichinger, C. ; Berg, G. What Is the Role of Archaea in Plants? New Insights from the Vegetation of Alpine Bogs. mSphere 2018, 3 (3), ARTN e00122-18. 10.1128/mSphere.00122-18 PMC5956146 29743201

[2838] Song G. C., Im H., Jung J., Lee S., Jung M. Y., Rhee S. K., Ryu C. M. (2019). Plant growth-promoting
archaea trigger induced systemic resistance in Arabidopsis thaliana
against Pectobacterium carotovorum and Pseudomonas syringae. Environmental Microbiology.

[2839] Farkas J. A., Picking J. W., Santangelo T. J. (2013). Genetic
techniques for the archaea. Annu Rev Genet.

[2840] Dodsworth J. A., Li L., Wei S., Hedlund B. P., Leigh J. A., de Figueiredo P. (2010). Interdomain
conjugal transfer of
DNA from bacteria to archaea. Appl Environ Microbiol.

[2841] Farkas J. A., Picking J. W., Santangelo T. J. (2013). Genetic
Techniques for the Archaea. Annual Review of
Genetics, Vol 47.

[2842] Maezato Y., Dana K., Blum P. (2011). Engineering
thermoacidophilic
archaea using linear DNA recombination. Methods
Mol Biol.

[2843] Pritchett M. A., Zhang J. K., Metcalf W. W. (2004). Development of a
markerless genetic exchange method for Methanosarcina acetivorans
C2A and its use in construction of new genetic tools for methanogenic
archaea. Appl Environ Microbiol.

[2844] Suzuki S., Kurosawa N. (2017). Development of the Multiple Gene
Knockout System with One-Step PCR in Thermoacidophilic Crenarchaeon
Sulfolobus acidocaldarius. Archaea.

[2845] Sato T., Fukui T., Atomi H., Imanaka T. (2003). Targeted gene
disruption by homologous recombination in the hyperthermophilic archaeon
Thermococcus kodakaraensis KOD1. J Bacteriol.

[2846] Bao J., de Dios Mateos E., Scheller S. (2022). Efficient CRISPR/Cas12a-Based
Genome-Editing Toolbox for Metabolic Engineering in Methanococcus
maripaludis. ACS Synth Biol.

[2847] Li J., Zhang L., Xu Q., Zhang W., Li Z., Chen L., Dong X. (2022). CRISPR-Cas9
Toolkit for Genome Editing
in an Autotrophic CO(2)-Fixing Methanogenic Archaeon. Microbiol Spectr.

[2848] Nayak D. D., Metcalf W. W. (2017). Cas9-mediated genome
editing in the
methanogenic archaeon Methanosarcina acetivorans. Proc Natl Acad Sci U S A.

[2849] Nayak D. D., Metcalf W. W. (2017). Cas9-mediated genome
editing in the
methanogenic archaeon Methanosarcina acetivorans. Proceedings of the National Academy of Sciences.

[2850] Nazem-Bokaee H., Gopalakrishnan S., Ferry J. G., Wood T. K., Maranas C. D. (2016). Assessing methanotrophy
and carbon fixation for biofuel
production by Methanosarcina acetivorans. Microb
Cell Fact.

[2851] Benedict M. N., Gonnerman M. C., Metcalf W. W., Price N. D. (2012). Genome-scale
metabolic reconstruction and hypothesis testing in the methanogenic
archaeon Methanosarcina acetivorans C2A. J Bacteriol.

[2852] Satish Kumar V., Ferry J. G., Maranas C. D. (2011). Metabolic reconstruction
of the archaeon methanogen Methanosarcina Acetivorans. BMC Syst Biol.

[2853] Gehring A. M., Walker J. E., Santangelo T. J. (2016). Transcription
Regulation in Archaea. J Bacteriol.

[2854] Gregor D., Pfeifer F. (2005). In vivo analyses of constitutive
and regulated promoters in halophilic archaea. Microbiology (Reading).

[2855] Koide, T. ; Reiss, D. J. ; Bare, J. C. ; Pang, W. L. ; Facciotti, M. T. ; Schmid, A. K. ; Pan, M. ; Marzolf, B. ; Van, P. T. ; Lo, F. Y. ; Prevalence of transcription promoters within archaeal operons and coding sequences. Molecular Systems Biology 2009, 5, ARTN 285. 10.1038/msb.2009.42 PMC2710873 19536208

[2856] Benelli D., Londei P. (2011). Translation initiation in Archaea:
conserved and domain-specific features. Biochem
Soc Trans.

[2857] Santangelo T. J., Cubonova L., Skinner K. M., Reeve J. N. (2009). Archaeal
intrinsic transcription termination in vivo. J Bacteriol.

[2858] Karr E. A. (2014). Transcription Regulation in the Third Domain. Adv Appl Microbiol.

[2859] Sybers D., Joka Bernauw A., El Masri D., Ramadan
Maklad H., Charlier D., De Mey M., Bervoets I., Peeters E. (2022). Engineering transcriptional regulation in Escherichia
coli using an archaeal TetR-family transcription factor. Gene.

[2860] Perez-Rueda E., Janga S. C. (2010). Identification and
genomic analysis
of transcription factors in archaeal genomes exemplifies their functional
architecture and evolutionary origin. Mol Biol
Evol.

[2861] Lemmens L., Maklad H. R., Bervoets I., Peeters E. (2019). Transcription
Regulators in Archaea: Homologies and Differences with Bacterial Regulators. J Mol Biol.

[2862] Guss A. M., Rother M., Zhang J. K., Kulkarni G., Metcalf W. W. (2008). New methods for tightly regulated
gene expression and
highly efficient chromosomal integration of cloned genes for Methanosarcina
species. Archaea.

[2863] Yang H., Lipscomb G. L., Keese A. M., Schut G. J., Thomm M., Adams M. W., Wang B. C., Scott R. A. (2010). SurR regulates
hydrogen production in Pyrococcus furiosus by a sulfur-dependent redox
switch. Mol Microbiol.

[2864] Akinyemi T. S., Shao N., Lyu Z., Drake I. J., Liu Y., Whitman W. B. (2021). Tuning Gene Expression
by Phosphate in the Methanogenic
Archaeon Methanococcus maripaludis. ACS Synth
Biol.

[2865] Lie T. J., Wood G. E., Leigh J. A. (2005). Regulation of nif
expression in Methanococcus maripaludis: roles of the euryarchaeal
repressor NrpR, 2-oxoglutarate, and two operators. J Biol Chem.

[2866] Peng N., Ao X., Liang Y. X., She Q. (2011). Archaeal promoter
architecture and mechanism of gene activation. Biochem Soc Trans.

[2867] Lee S. J., Surma M., Hausner W., Thomm M., Boos W. (2008). The role of
TrmB and TrmB-like transcriptional regulators for sugar
transport and metabolism in the hyperthermophilic archaeon Pyrococcus
furiosus. Arch Microbiol.

[2868] Johnsen U., Sutter J. M., Schulz A. C., Tastensen J. B., Schonheit P. (2015). XacR - a novel transcriptional regulator
of D-xylose
and L-arabinose catabolism in the haloarchaeon Haloferax volcanii. Environmental Microbiology.

[2869] Wagner M., Wagner A., Ma X., Kort J. C., Ghosh A., Rauch B., Siebers B., Albers S. V. (2014). Investigation
of the malE promoter and MalR, a positive regulator of the maltose
regulon, for an improved expression system in Sulfolobus acidocaldarius. Appl Environ Microbiol.

[2870] Di Fiore A., Fiorentino G., Vitale R. M., Ronca R., Amodeo P., Pedone C., Bartolucci S., De Simone G. (2009). Structural analysis of BldR from
Sulfolobus solfataricus
provides insights into the molecular basis of transcriptional activation
in Archaea by MarR family proteins. J Mol Biol.

[2871] Reichlen M. J., Vepachedu V. R., Murakami K. S., Ferry J. G. (2012). MreA functions
in the global regulation of methanogenic pathways in Methanosarcina
acetivorans. mBio.

[2872] Bose A., Kulkarni G., Metcalf W. W. (2009). Regulation
of putative
methyl-sulphide methyltransferases in Methanosarcina acetivorans C2A. Mol Microbiol.

[2873] Brinkman A. B., Bell S. D., Lebbink R. J., de Vos W. M., van der Oost J. (2002). The Sulfolobus solfataricus Lrp-like
protein LysM regulates
lysine biosynthesis in response to lysine availability. J Biol Chem.

[2874] Vassart A., Van Wolferen M., Orell A., Hong Y., Peeters E., Albers S. V., Charlier D. (2013). Sa-Lrp from Sulfolobus
acidocaldarius is a versatile, glutamine-responsive, and architectural
transcriptional regulator. Microbiologyopen.

[2875] Schwaiger R., Schwarz C., Furtwangler K., Tarasov V., Wende A., Oesterhelt D. (2010). Transcriptional
control by two leucine-responsive regulatory proteins in Halobacterium
salinarum R1. BMC Mol Biol.

[2876] Hochheimer A., Hedderich R., Thauer R. K. (1998). The formylmethanofuran
dehydrogenase isoenzymes in Methanobacterium wolfei and Methanobacterium
thermoautotrophicum: induction of the molybdenum isoenzyme by molybdate
and constitutive synthesis of the tungsten isoenzyme. Arch Microbiol.

[2877] Wang G., Kennedy S. P., Fasiludeen S., Rensing C., DasSarma S. (2004). Arsenic resistance in Halobacterium
sp. strain NRC-1 examined by using an improved gene knockout system. J Bacteriol.

[2878] Schelert J., Drozda M., Dixit V., Dillman A., Blum P. (2006). Regulation of mercury resistance
in the crenarchaeote Sulfolobus
solfataricus. J Bacteriol.

[2879] Chivers P. T., Tahirov T. H. (2005). Structure of Pyrococcus
horikoshii
NikR: nickel sensing and implications for the regulation of DNA recognition. J Mol Biol.

[2880] Speed, M. C. ; Burkhart, B. W. ; Picking, J. W. ; Santangelo, T. J. An Archaeal Fluoride-Responsive Riboswitch Provides an Inducible Expression System for Hyperthermophiles. Appl Environ Microbiol 2018, 84 (7), 10.1128/AEM.02306-17. PMC5861836 29352088

[2881] Zink, I. A. ; Wimmer, E. ; Schleper, C. Heavily Armed Ancestors: CRISPR Immunity and Applications in Archaea with a Comparative Analysis of CRISPR Types in Sulfolobales. Biomolecules 2020, 10 (11), 1523 10.3390/biom10111523. 33172134 PMC7694759

[2882] Stachler A. E., Schwarz T. S., Schreiber S., Marchfelder A. (2020). CRISPRi as an efficient tool for gene repression in
archaea. Methods.

[2883] Dhamad, A. E. ; Lessner, D. J. A CRISPRi-dCas9 System for Archaea and Its Use To Examine Gene Function during Nitrogen Fixation by Methanosarcina acetivorans. Appl Environ Microbiol 2020, 86 (21), 10.1128/AEM.01402-20. PMC7580536 32826220

[2884] Zebec Z., Manica A., Zhang J., White M. F., Schleper C. (2014). CRISPR-mediated targeted mRNA degradation
in the archaeon
Sulfolobus solfataricus. Nucleic Acids Res.

[2885] Pozo M. J., Zabalgogeazcoa I., Vazquez de Aldana B.
R., Martinez-Medina A. (2021). Untapping
the potential of plant mycobiomes for applications
in agriculture. Curr Opin Plant Biol.

[2886] Carroll G. (1988). Fungal Endophytes in Stems and Leaves
- from Latent
Pathogen to Mutualistic Symbiont. Ecology.

[2887] Huang W.
Y., Cai Y. Z., Surveswaran S., Hyde K. D., Corke H., Sun M. (2009). Molecular
phylogenetic
identification of endophytic fungi isolated from three Artemisia species. Fungal Divers.

[2888] Sun X., Guo L. D., Hyde K. D. (2011). Community composition of endophytic
fungi in Acer truncatum and their role in decomposition. Fungal Divers.

[2889] Clay K., Shearin Z. R. C., Bourke K. A., Bickford W. A., Kowalski K. P. (2016). Diversity of fungal endophytes in
non-native Phragmites
australis in the Great Lakes. Biol Invasions.

[2890] Backman P. A., Sikora R. A. (2008). Endophytes: An emerging tool for
biological control. Biol Control.

[2891] Frey S. D. (2019). Mycorrhizal Fungi as Mediators of
Soil Organic Matter
Dynamics. Annu Rev Ecol Evol S.

[2892] Etesami H., Jeong B. R., Glick B. R. (2021). Contribution
of
Arbuscular Mycorrhizal Fungi, Phosphate-Solubilizing Bacteria, and
Silicon to P Uptake by Plant. Front Plant Sci.

[2893] Ceballos I., Ruiz M., Fernandez C., Pena R., Rodriguez A., Sanders I. R. (2013). The in vitro mass-produced
model mycorrhizal fungus, Rhizophagus irregularis, significantly increases
yields of the globally important food security crop cassava. PLoS One.

[2894] Oono R., Lefevre E., Simha A., Lutzoni F. (2015). A comparison
of the community diversity of foliar fungal endophytes between seedling
and adult loblolly pines (Pinus taeda). Fungal
Biol.

[2895] Veresoglou S. D., Rillig M. C. (2012). Suppression of fungal
and nematode
plant pathogens through arbuscular mycorrhizal fungi. Biol Lett.

[2896] Huang Y. L., Kuang Z. Y., Wang W. F., Cao L. X. (2016). Exploring
potential bacterial and fungal biocontrol agents transmitted from
seeds to sprouts of wheat. Biol Control.

[2897] Zhang X., Zhou Y. Y., Li Y., Fu X. C., Wang Q. (2017). Screening and characterization of
endophytic Bacillus for biocontrol
of grapevine downy mildew. Crop Prot.

[2898] Verma M., Brar S. K., Tyagi R. D., Surampalli R. Y., Valero J. R. (2007). Antagonistic fungi, Trichoderma spp.:
Panoply of biological
control. Biochem Eng J.

[2899] Gardiner D. M., Rusu A., Barrett L., Hunter G. C., Kazan K. (2020). Can natural gene drives be part of
future fungal pathogen control
strategies in plants?. New Phytologist.

[2900] Yun H. G., Kim D. J., Gwak W. S., Shin T. Y., Woo S. D. (2017). Entomopathogenic
Fungi as Dual Control Agents against
Both the Pest Myzus persicae and Phytopathogen Botrytis cinerea. Mycobiology.

[2901] Jaber L. R., Ownley B. H. (2018). Can we use entomopathogenic fungi
as endophytes for dual biological control of insect pests and plant
pathogens?. Biol Control.

[2902] Siddiqui Z. A., Mahmood I. (1996). Biological control
of plant parasitic
nematodes by fungi: A review. Bioresource Technol.

[2903] Iwanicki N. S. A., Pereira A. A., Botelho A., Rezende J. M., Moral R. A., Zucchi M. I., Delalibera Junior I. (2019). Monitoring
of the field application of Metarhizium anisopliae in Brazil revealed
high molecular diversity of Metarhizium spp in insects, soil and sugarcane
roots. Sci Rep.

[2904] Parra J. R. P. (2014). Biological Control in Brazil: An overview. Sci Agr.

[2905] Duran P., Thiergart T., Garrido-Oter R., Agler M., Kemen E., Schulze-Lefert P., Hacquard S. (2018). Microbial Interkingdom Interactions in Roots Promote
Arabidopsis Survival. Cell.

[2906] Jiang X., Zerfass C., Feng S., Eichmann R., Asally M., Schafer P., Soyer O. S. (2018). Impact
of spatial
organization on a novel auxotrophic interaction among soil microbes. ISME J.

[2907] Deveau A., Brule C., Palin B., Champmartin D., Rubini P., Garbaye J., Sarniguet A., Frey-Klett P. (2010). Role of fungal trehalose and bacterial thiamine in
the improved survival and growth of the ectomycorrhizal fungus Laccaria
bicolor S238N and the helper bacterium Pseudomonas fluorescens BBc6R8. Environ Microbiol Rep.

[2908] Rajendran G., Mistry S., Desai A. J., Archana G. (2007). Functional
expression of Escherichia coli fhuA gene in Rhizobium spp. of Cajanus
cajan provides growth advantage in presence of Fe3+: ferrichrome as
iron source. Arch Microbiol.

[2909] Roth M. G., Chilvers M. I. (2019). A protoplast generation
and transformation
method for soybean sudden death syndrome causal agents Fusarium virguliforme
and F. brasiliense. Fungal Biol Biotechnol.

[2910] Li D., Tang Y., Lin J., Cai W. (2017). Methods for genetic
transformation of filamentous fungi. Microb
Cell Fact.

[2911] Fincham J. R. (1989). Transformation
in fungi. Microbiol
Rev.

[2912] Shi L., Chen D., Xu C., Ren A., Yu H., Zhao M. (2017). Highly-efficient liposome-mediated transformation system
for the
basidiomycetous fungus Flammulina velutipes. J Gen Appl Microbiol.

[2913] Rehman L., Su X., Guo H., Qi X., Cheng H. (2016). Protoplast transformation as a potential platform for
exploring gene
function in Verticillium dahliae. BMC Biotechnol.

[2914] de Groot M. J., Bundock P., Hooykaas P. J., Beijersbergen A. G. (1998). Agrobacterium
tumefaciens-mediated transformation of filamentous fungi. Nat Biotechnol.

[2915] Michielse C. B., Hooykaas P. J., van den
Hondel C. A., Ram A. F. (2008). Agrobacterium-mediated transformation
of the filamentous
fungus Aspergillus awamori. Nat Protoc.

[2916] Lichius, A. ; Ruiz, D. M. ; Zeilinger, S. Genetic Transformation of Filamentous Fungi: Achievements and Challenges. In Grand Challenges in Fungal Biotechnology; Nevalainen, H. Ed.; Springer International Publishing, 2020; pp 123-164.

[2917] Fierro F., Kosalkova K., Gutierrez S., Martin J. F. (1996). Autonomously replicating
plasmids carrying the AMA1
region in Penicillium chrysogenum. Curr Genet.

[2918] Aleksenko A., Clutterbuck A. J. (1997). Autonomous plasmid replication in
Aspergillus nidulans: AMA1 and MATE elements. Fungal Genet Biol.

[2919] Gems D., Johnstone I. L., Clutterbuck A. J. (1991). An autonomously
replicating plasmid transforms Aspergillus nidulans at high frequency. Gene.

[2920] Lubertozzi D., Keasling J. D. (2009). Developing Aspergillus as a host
for heterologous expression. Biotechnol Adv.

[2921] Song R., Zhai Q., Sun L., Huang E., Zhang Y., Zhu Y., Guo Q., Tian Y., Zhao B., Lu H. (2019). CRISPR/Cas9
genome editing technology
in filamentous fungi: progress and perspective. Appl Microbiol Biotechnol.

[2922] Wang Q., Coleman J. J. (2019). Progress and Challenges:
Development
and Implementation of CRISPR/Cas9 Technology in Filamentous Fungi. Comput Struct Biotechnol J.

[2923] Woodcraft C., Chooi Y. H., Roux I. (2023). The expanding
CRISPR
toolbox for natural product discovery and engineering in filamentous
fungi. Nat Prod Rep.

[2924] Jarczynska Z. D., Rendsvig J. K. H., Pagels N., Viana V. R., Nodvig C. S., Kirchner F. H., Strucko T., Nielsen M. L., Mortensen U. H. (2021). DIVERSIFY: A Fungal Multispecies
Gene Expression Platform. ACS Synth Biol.

[2925] Huang L., Dong H., Zheng J., Wang B., Pan L. (2019). Highly efficient
single base editing in Aspergillus niger with CRISPR/Cas9
cytidine deaminase fusion. Microbiol Res.

[2926] Arazoe T., Ogawa T., Miyoshi K., Yamato T., Ohsato S., Sakuma T., Yamamoto T., Arie T., Kuwata S. (2015). Tailor-made TALEN system for highly
efficient targeted
gene replacement in the rice blast fungus. Biotechnol
Bioeng.

[2927] Snoek I. S., van der Krogt Z. A., Touw H., Kerkman R., Pronk J. T., Bovenberg R. A., van den Berg M. A., Daran J. M. (2009). Construction of
an hdfA Penicillium chrysogenum strain
impaired in non-homologous end-joining and analysis of its potential
for functional analysis studies. Fungal Genet
Biol.

[2928] Helber N., Requena N. (2008). Expression of the fluorescence markers
DsRed and GFP fused to a nuclear localization signal in the arbuscular
mycorrhizal fungus Glomus intraradices. New
Phytol.

[2929] Mattern D. J., Valiante V., Horn F., Petzke L., Brakhage A. A. (2017). Rewiring of the Austinoid Biosynthetic
Pathway in Filamentous
Fungi. ACS Chem Biol.

[2930] Wang C., St Leger R. J. (2007). A scorpion neurotoxin
increases the
potency of a fungal insecticide. Nat Biotechnol.

[2931] Tseng M.
N., Chung P. C., Tzean S. S. (2011). Enhancing the stress
tolerance and virulence of an entomopathogen by metabolic engineering
of dihydroxynaphthalene melanin biosynthesis genes. Appl Environ Microbiol.

[2932] Rosikiewicz P., Bonvin J., Sanders I. R. (2017). Cost-efficient
production
of in vitro Rhizophagus irregularis. Mycorrhiza.

[2933] Kameoka H., Tsutsui I., Saito K., Kikuchi Y., Handa Y., Ezawa T., Hayashi H., Kawaguchi M., Akiyama K. (2019). Stimulation of asymbiotic sporulation in arbuscular
mycorrhizal fungi by fatty acids. Nat Microbiol.

[2934] van den Berg M. A., Maruthachalam K. (2015). Genetic Transformation Systems in
Fungi, Vol 2. Genetic Transformation Systems
in Fungi.

[2935] Forbes P. J., Millam S., Hooker J. E., Harrier L. A. (1998). Transformation
of the arbuscular mycorrhiza Gigaspora rosea by particle bombardment. Mycol Res.

[2936] Miyauchi S., Kiss E., Kuo A., Drula E., Kohler A., Sanchez-Garcia M., Morin E., Andreopoulos B., Barry K. W., Bonito G. (2020). Large-scale genome sequencing
of mycorrhizal fungi provides insights into the early evolution of
symbiotic traits. Nat Commun.

[2937] Mozsik L., Pohl C., Meyer V., Bovenberg R. A. L., Nygard Y., Driessen A. J. M. (2021). Modular Synthetic
Biology Toolkit
for Filamentous Fungi. ACS Synth Biol.

[2938] Moreno-Gimenez E., Gandia M., Saez Z., Manzanares P., Yenush L., Orzaez D., Marcos J. F., Garrigues S. (2023). FungalBraid
2.0: expanding the synthetic biology toolbox for the biotechnological
exploitation of filamentous fungi. Front Bioeng
Biotechnol.

[2940] Tanaka M., Tokuoka M., Gomi K. (2014). Effects of
codon optimization
on the mRNA levels of heterologous genes in filamentous fungi. Appl Microbiol Biotechnol.

[2941] Unkles S. E., Valiante V., Mattern D. J., Brakhage A. A. (2014). Synthetic
biology tools for bioprospecting of natural products in eukaryotes. Chem Biol.

[2942] St Leger R., Joshi L., Bidochka M. J., Roberts D. W. (1996). Construction
of an improved mycoinsecticide overexpressing a toxic protease. Proc Natl Acad Sci U S A.

[2943] Hu G., St Leger J. (2002). Field studies using
a recombinant mycoinsecticide (Metarhizium
anisopliae) reveal that it is rhizosphere competent. Appl Environ Microb.

[2944] Liu Y. J., Liu J., Ying S. H., Liu S. S., Feng M. G. (2013). A fungal insecticide engineered for
fast per os killing
of caterpillars has high field efficacy and safety in full-season
control of cabbage insect pests. Appl Environ
Microbiol.

[2945] Wang Z. L., Ying S. H., Feng M. G. (2013). Recognition of a
core fragment of Beauveria bassiana hydrophobin gene promoter (P hyd1)
and its special use in improving fungal biocontrol potential. Microb Biotechnol.

[2946] Shang Y., Duan Z., Huang W., Gao Q., Wang C. (2012). Improving UV resistance and virulence of Beauveria
bassiana by genetic
engineering with an exogenous tyrosinase gene. J Invertebr Pathol.

[2947] Xie X. Q., Wang J., Huang B. F., Ying S. H., Feng M. G. (2010). A new manganese superoxide dismutase identified from
Beauveria bassiana enhances virulence and stress tolerance when overexpressed
in the fungal pathogen. Appl Microbiol Biotechnol.

[2948] de Crecy E., Jaronski S., Lyons B., Lyons T. J., Keyhani N. O. (2009). Directed
evolution of a filamentous fungus for thermotolerance. BMC Biotechnol.

[2949] Han J. O., Naeger N. L., Hopkins B. K., Sumerlin D., Stamets P. E., Carris L. M., Sheppard W. S. (2021). Directed evolution
of Metarhizium fungus improves its biocontrol efficacy against Varroa
mites in honey bee colonies. Sci Rep.

[2950] Blackwell B. A., Gilliam J. T., Savard M. E., David
Miller J., Duvick J. P. (1999). Oxidative deamination of hydrolyzed
fumonisin B1 (AP1) by cultures of Exophiala spinifera. Natural Toxins.

[2951] Jo C., Zhang J., Tam J. M., Church G. M., Khalil A. S., Segre D., Tang T. C. (2023). Unlocking
the magic in mycelium:
Using synthetic biology to optimize filamentous fungi for biomanufacturing
and sustainability. Mater Today Bio.

[2952] Meyer V. (2008). Genetic engineering of filamentous
fungi—progress, obstacles
and future trends. Biotechnology advances.

[2953] Meyer V. (2021). Metabolic
engineering of filamentous fungi. Metabolic
engineering: concepts and applications.

[2954] Cary, J. W. ; Chang, P. K. ; Bhatnagar, D. Clustered metabolic pathway genes in filamentous fungi. In Applied Mycology and Biotechnology; Khachatourians, G. G. , Arora, D. K. Eds.; Vol. 1; Elsevier, 2001; pp 165-198.

[2955] Alberti F., Foster G. D., Bailey A. M. (2017). Natural products
from filamentous fungi and production by heterologous expression. Appl Microbiol Biotechnol.

[2956] Almeida H., Palys S., Tsang A., Diallo A. B. (2020). TOUCAN:
a framework for fungal biosynthetic gene cluster discovery. NAR Genom Bioinform.

[2957] Clevenger K. D., Bok J. W., Ye R., Miley G. P., Verdan M. H., Velk T., Chen C., Yang K., Robey M. T., Gao P. (2017). A scalable platform
to identify fungal secondary metabolites and their gene clusters. Nat Chem Biol.

[2958] Kan E., Katsuyama Y., Maruyama J. I., Tamano K., Koyama Y., Ohnishi Y. (2019). Production
of the plant polyketide curcumin in Aspergillus
oryzae: strengthening malonyl-CoA supply for yield improvement. Biosci Biotechnol Biochem.

[2959] Kan E., Katsuyama Y., Maruyama J. I., Tamano K., Koyama Y., Ohnishi Y. (2020). Efficient
heterologous production of atrochrysone carboxylic
acid-related polyketides in an Aspergillus oryzae host with enhanced
malonyl-coenzyme A supply. J Gen Appl Microbiol.

[2960] Roux I., Woodcraft C., Hu J., Wolters R., Gilchrist C. L. M., Chooi Y. H. (2020). CRISPR-Mediated Activation of Biosynthetic
Gene Clusters for Bioactive Molecule Discovery in Filamentous Fungi. ACS Synth Biol.

[2961] Mozsik L., Hoekzema M., de Kok N. A. W., Bovenberg R. A. L., Nygard Y., Driessen A. J. (2021). M. CRISPR-based
transcriptional activation
tool for silent genes in filamentous fungi. Sci Rep.

[2962] Feist A. M., Scholten J. C., Palsson B. O., Brockman F. J., Ideker T. (2006). Modeling methanogenesis
with a genome-scale metabolic
reconstruction of Methanosarcina barkeri. Mol
Syst Biol.

[2963] Andersen M. R., Nielsen M. L., Nielsen J. (2008). Metabolic model integration
of the bibliome, genome, metabolome and reactome of Aspergillus niger. Mol Syst Biol.

[2964] Lu H., Cao W., Ouyang L., Xia J., Huang M., Chu J., Zhuang Y., Zhang S., Noorman H. (2017). Comprehensive reconstruction
and in silico analysis of Aspergillus niger genome-scale metabolic
network model that accounts for 1210 ORFs. Biotechnol
Bioeng.

[2965] Vongsangnak W., Olsen P., Hansen K., Krogsgaard S., Nielsen J. (2008). Improved annotation through genome-scale metabolic
modeling of Aspergillus oryzae. BMC Genomics.

[2966] Liu J., Gao Q., Xu N., Liu L. (2013). Genome-scale reconstruction
and in silico analysis of Aspergillus terreus metabolism. Mol Biosyst.

[2967] Vongsangnak W., Raethong N., Mujchariyakul W., Nguyen N. N., Leong H. W., Laoteng K. (2017). Genome-scale metabolic
network of Cordyceps militaris useful for comparative analysis of
entomopathogenic fungi. Gene.

[2968] Dreyfuss J. M., Zucker J. D., Hood H. M., Ocasio L. R., Sachs M. S., Galagan J. E. (2013). Reconstruction and
validation of
a genome-scale metabolic model for the filamentous fungus Neurospora
crassa using FARM. PLoS Comput Biol.

[2969] Schramm, M. ; Friedrich, S. ; Schmidtke, K. U. ; Kiebist, J. ; Panzer, P. ; Kellner, H. ; Ullrich, R. ; Hofrichter, M. ; Scheibner, K. Cell-Free Protein Synthesis with Fungal Lysates for the Rapid Production of Unspecific Peroxygenases. Antioxidants (Basel) 2022, 11 (2), 284 10.3390/antiox11020284. 35204167 PMC8868270

[2970] Walter R. M., Zemella A., Schramm M., Kiebist J., Kubick S. (2022). Vesicle-based cell-free synthesis
of short and long
unspecific peroxygenases. Front Bioeng Biotechnol.

[2971] Wu C., Wei J., Lin P. J., Tu L., Deutsch C., Johnson A. E., Sachs M. S. (2012). Arginine changes
the conformation
of the arginine attenuator peptide relative to the ribosome tunnel. J Mol Biol.

[2972] Sachs M. S., Wang Z., Gaba A., Fang P., Belk J., Ganesan R., Amrani N., Jacobson A. (2002). Toeprint analysis
of the positioning of translation apparatus components at initiation
and termination codons of fungal mRNAs. Methods.

[2973] Kluge J., Terfehr D., Kuck U. (2018). Inducible promoters
and functional genomic approaches for the genetic engineering of filamentous
fungi. Appl Microbiol Biotechnol.

[2974] Boel E., Hansen M. T., Hjort I., Hoegh I., Fiil N. P. (1984). Two different
types of intervening sequences in the
glucoamylase gene from Aspergillus niger. EMBO
J.

[2975] Waring R. B., May G. S., Morris N. R. (1989). Characterization
of an inducible expression system in Aspergillus nidulans using alcA
and tubulin-coding genes. Gene.

[2976] Geng A., Zou G., Yan X., Wang Q., Zhang J., Liu F., Zhu B., Zhou Z. (2012). Expression
and characterization of a novel metagenome-derived cellulase Exo2b
and its application to improve cellulase activity in Trichoderma reesei. Appl Microbiol Biotechnol.

[2977] Tada S., Gomi K., Kitamoto K., Takahashi K., Tamura G., Hara S. (1991). Construction of a fusion
gene comprising
the Taka-amylase A promoter and the Escherichia coli beta-glucuronidase
gene and analysis of its expression in Aspergillus oryzae. Mol Gen Genet.

[2978] Antunes M. S., Hodges T. K., Carpita N. C. (2016). A benzoate-activated
promoter from Aspergillus niger and regulation of its activity. Appl Microbiol Biotechnol.

[2979] Flipphi M. J., Visser J., van der
Veen P., de Graaff L. H. (1994). Arabinase gene expression in Aspergillus niger: indications
for coordinated regulation. Microbiology (Reading).

[2980] Janus D., Hortschansky P., Kuck U. (2008). Identification of a
minimal cre1 promoter sequence promoting glucose-dependent gene expression
in the beta-lactam producer Acremonium chrysogenum. Curr Genet.

[2981] Gouka R. J., Hessing J. G., Punt P. J., Stam H., Musters W., Van den Hondel C. A. (1996). An expression system based on the
promoter region of the Aspergillus awamori 1,4-beta-endoxylanase A
gene. Appl Microbiol Biotechnol.

[2982] Bischof R. H., Horejs J., Metz B., Gamauf C., Kubicek C. P., Seiboth B. (2015). L-Methionine repressible
promoters
for tuneable gene expression in Trichoderma reesei. Microb Cell Fact.

[2983] Ishida H., Hata Y., Kawato A., Abe Y., Kashiwagi Y. (2004). Isolation
of a novel promoter for efficient protein
production in Aspergillus oryzae. Biosci Biotechnol
Biochem.

[2984] Shoji J. Y., Maruyama J., Arioka M., Kitamoto K. (2005). Development
of Aspergillus oryzae thiA promoter as a tool for molecular biological
studies. FEMS Microbiol Lett.

[2985] Lamb T. M., Vickery J., Bell-Pedersen D. (2013). Regulation
of gene expression in Neurospora crassa with a copper responsive promoter. G3 (Bethesda).

[2986] Yin, X. ; Shin, H. D. ; Li, J. ; Du, G. ; Liu, L. ; Chen, J. Pgas, a Low-pH-Induced Promoter, as a Tool for Dynamic Control of Gene Expression for Metabolic Engineering of Aspergillus niger. Appl Environ Microbiol 2017, 83 (6), 10.1128/AEM.03222-16. PMC5335535 28087530

[2987] Muller C., Hjort C. M., Hansen K., Nielsen J. (2002). Altering the
expression of two chitin synthase genes differentially affects the
growth and morphology of Aspergillus oryzae. Microbiology (Reading).

[2988] Lee J., Son H., Lee S., Park A. R., Lee Y. W. (2010). Development
of a conditional gene expression system using a zearalenone-inducible
promoter for the ascomycete fungus Gibberella zeae. Appl Environ Microbiol.

[2989] Rantasalo A., Landowski C. P., Kuivanen J., Korppoo A., Reuter L., Koivistoinen O., Valkonen M., Penttila M., Jantti J., Mojzita D. (2018). A universal
gene expression system
for fungi. Nucleic Acids Res.

[2990] Pachlinger R., Mitterbauer R., Adam G., Strauss J. (2005). Metabolically
independent and accurately adjustable Aspergillus sp. expression system. Appl Environ Microbiol.

[2991] Vogt K., Bhabhra R., Rhodes J. C., Askew D. S. (2005). Doxycycline-regulated
gene expression in the opportunistic fungal pathogen Aspergillus fumigatus. BMC Microbiol.

[2992] Wanka F., Cairns T., Boecker S., Berens C., Happel A., Zheng X., Sun J., Krappmann S., Meyer V. (2016). Tet-on, or Tet-off, that is the question:
Advanced conditional gene
expression in Aspergillus. Fungal Genet Biol.

[2993] Baldin C., Kuhbacher A., Merschak P., Wagener J., Gsaller F. (2022). Modular Inducible Multigene
Expression System for Filamentous
Fungi. Microbiol Spectr.

[2994] Wang W., Shi X. Y., Wei D. Z. (2014). Light-mediated
control
of gene expression in filamentous fungus Trichoderma reesei. J Microbiol Methods.

[2995] Wang Q., Cobine P. A., Coleman J. J. (2018). Efficient genome
editing in Fusarium oxysporum based on CRISPR/Cas9 ribonucleoprotein
complexes. Fungal Genet Biol.

[2996] Fang Y., Tyler B. M. (2016). Efficient disruption
and replacement
of an effector gene in the oomycete Phytophthora sojae using CRISPR/Cas9. Mol Plant Pathol.

[2997] Matsu-Ura T., Dovzhenok A. A., Coradetti S. T., Subramanian K. R., Meyer D. R., Kwon J. J., Kim C., Salomonis N., Glass N. L., Lim S. (2018). Synthetic
Gene Network with Positive Feedback Loop Amplifies Cellulase Gene
Expression in Neurospora crassa. ACS Synth Biol.

[2998] Redden H., Morse N., Alper H. S. (2015). The synthetic biology
toolbox for tuning gene expression in yeast. FEMS Yeast Res.

[2999] Nasr M. A., Timmins L. R., Martin V. J. J., Kwan D. H. (2022). A Versatile
Transcription Factor Biosensor System Responsive to Multiple Aromatic
and Indole Inducers. ACS Synth Biol.

[3000] D'Ambrosio V., Pramanik S., Goroncy K., Jakočiu̅nas T., Schönauer D., Davari M. D., Schwaneberg U., Keasling J. D., Jensen M. K. (2020). Directed evolution of VanR biosensor
specificity in yeast. Biotechnology notes.

[3001] Skjoedt M. L., Snoek T., Kildegaard K. R., Arsovska D., Eichenberger M., Goedecke T. J., Rajkumar A. S., Zhang J., Kristensen M., Lehka B. J. (2016). Engineering prokaryotic
transcriptional activators as metabolite biosensors in yeast. Nature Chemical Biology.

[3002] Teo W. S., Chang M. W. (2015). Bacterial XylRs
and synthetic promoters
function as genetically encoded xylose biosensors in Saccharomyces
cerevisiae. Biotechnology journal.

[3003] Moser F., Horwitz A., Chen J., Lim W., Voigt C. A. (2013). Genetic sensor for strong methylating compounds. ACS Synth Biol.

[3004] Park J. H., Bassalo M. C., Lin G. M., Chen Y., Doosthosseini H., Schmitz J., Roubos J. A., Voigt C. A. (2023). Design
of Four Small-Molecule-Inducible Systems in the Yeast Chromosome,
Applied to Optimize Terpene Biosynthesis. ACS
Synth Biol.

[3005] Sanford A., Kiriakov S., Khalil A. S. (2022). A Toolkit for Precise,
Multigene Control in Saccharomyces cerevisiae. ACS Synth Biol.

[3006] Gnügge R., Dharmarajan L., Lang M., Stelling J. r. (2016). An orthogonal
permease-inducer-repressor feedback loop shows bistability. ACS synthetic biology.

[3007] Ryo S., Ishii J., Matsuno T., Nakamura Y., Matsubara D., Tominaga M., Kondo A. (2017). Positive feedback
genetic circuit
incorporating a constitutively active mutant Gal3 into yeast GAL induction
system. ACS Synthetic Biology.

[3008] Rantasalo A., Kuivanen J., Penttila M., Jantti J., Mojzita D. (2018). Synthetic Toolkit for Complex Genetic
Circuit Engineering
in Saccharomyces cerevisiae. ACS Synth Biol.

[3009] Gander M. W., Vrana J. D., Voje W. E., Carothers J. M., Klavins E. (2017). Digital logic circuits in yeast with CRISPR-dCas9 NOR
gates. Nat Commun.

[3010] Roman, E. ; Coman, I. ; Prieto, D. ; Alonso-Monge, R. ; Pla, J. Implementation of a CRISPR-Based System for Gene Regulation in Candida albicans. mSphere 2019, 4 (1), 10.1128/mSphere.00001-19. PMC6374588 30760608

[3011] Brochado A. R., Matos C., Møller B. L., Hansen J., Mortensen U. H., Patil K. R. (2010). Improved vanillin
production in baker's yeast through in silico design. Microbial Cell Factories.

[3012] Cardenas J., Da Silva N. A. (2014). Metabolic engineering of Saccharomyces
cerevisiae for the production of triacetic acid lactone. Metabolic engineering.

[3013] Cai P., Gao J., Zhou Y. (2019). CRISPR-mediated genome
editing in
non-conventional yeasts for biotechnological applications. Microb Cell Fact.

[3014] Wang L., Lin J., Zhang T., Xu K., Ren C., Zhang Z. (2013). Simultaneous screening and validation of effective
zinc finger nucleases in yeast. PLoS One.

[3015] Fatma Z., Tan S. I., Boob A. G., Zhao H. (2023). A landing
pad system for multicopy gene integration in Issatchenkia orientalis. Metab Eng.

[3016] Barbieri E. M., Muir P., Akhuetie-Oni B. O., Yellman C. M., Isaacs F. J. (2017). Precise editing at DNA replication
forks enables multiplex genome engineering in eukaryotes. Cell.

[3017] Leiter É., Gáll T., Csernoch L., Pócsi I. (2017). Biofungicide
utilizations of antifungal proteins of filamentous ascomycetes: current
and foreseeable future developments. BioControl.

[3018] Jones R. W., Prusky D. (2002). Expression of an antifungal
peptide
in Saccharomyces: a new approach for biological control of the postharvest
disease caused by Colletotrichum coccodes. Phytopathology.

[3019] Janisiewicz W., Pereira I. B., Almeida M., Roberts D., Wisniewski M., Kurtenbach E. (2008). Improved biocontrol of fruit decay
fungi with Pichia pastoris recombinant strains expressing Psd1 antifungal
peptide. Postharvest biology and technology.

[3020] Murphy K. A., Tabuloc C. A., Cervantes K. R., Chiu J. C. (2016). Ingestion of genetically
modified yeast symbiont reduces
fitness of an insect pest via RNA interference. Sci Rep.

[3021] Hernández-Fernández M., Cordero-Bueso G., Ruiz-Muñoz M., Cantoral J. M. (2021). Culturable Yeasts
as Biofertilizers
and Biopesticides for a Sustainable Agriculture: A Comprehensive Review. Plants.

[3022] Kildegaard K. R., Arnesen J. A., Adiego-Perez B., Rago D., Kristensen M., Klitgaard A. K., Hansen E. H., Hansen J., Borodina I. (2021). Tailored biosynthesis
of gibberellin plant hormones in yeast. Metab
Eng.

[3023] Chaisupa P., Rahman M. M., Hildreth S. B., Moseley S., Gatling C., Bryant M. R., Helm R. F., Wright R. C. (2024). Genetically
Encoded, Noise-Tolerant, Auxin Biosensors in Yeast. ACS Synthetic Biology.

[3024] Hesham A. E.-L. (2011). Molecular genetic identification
of yeast strains isolated
from egyptian soils for solubilization of inorganic phosphates and
growth promotion of corn plants. J Microbiol
Biotechnol.

[3025] Mukherjee S., Sen S. K. (2015). Exploration of novel rhizospheric
yeast isolate as fertilizing soil inoculant for improvement of maize
cultivation. J Sci Food Agric.

[3026] Petkova, M. ; Petrova, S. ; Spasova-Apostolova, V. ; Naydenov, M. Tobacco Plant Growth-Promoting and Antifungal Activities of Three Endophytic Yeast Strains. Plants (Basel) 2022, 11 (6), 751 10.3390/plants11060751. 35336632 PMC8953121

[3027] Sun G. L., Reynolds E. E., Belcher A. M. (2019). Designing
yeast
as plant-like hyperaccumulators for heavy metals. Nature Communications.

[3028] Sun G. L., Reynolds E. E., Belcher A. M. (2020). Using yeast
to sustainably
remediate and extract heavy metals from waste waters. Nature Sustainability.

[3029] Beltran-Flores E., Pla-Ferriol M., Martinez-Alonso M., Gaju N., Blanquez P., Sarra M. (2022). Fungal bioremediation
of agricultural wastewater in a long-term treatment: biomass stabilization
by immobilization strategy. J Hazard Mater.

[3030] Lu Y. C., Luo F., Pu Z. J., Zhang S., Huang M. T., Yang H. (2016). Enhanced detoxification
and degradation
of herbicide atrazine by a group of O-methyltransferases in rice. Chemosphere.

[3031] Cushman J. C., Bohnert H. J. (2000). Genomic approaches
to plant stress
tolerance. Curr Opin Plant Biol.

[3032] Bhatnagar-Mathur P., Vadez V., Sharma K. K. (2008). Transgenic
approaches
for abiotic stress tolerance in plants: retrospect and prospects. Plant Cell Rep.

[3033] Wu X. F., Wang C. L., Xie E. B., Gao Y., Fan Y. L., Liu P. Q., Zhao K. J. (2009). Molecular cloning
and characterization of the promoter for the multiple stress-inducible
gene BjCHI1 from Brassica juncea. Planta.

[3034] Tiwari V., Patel M. K., Chaturvedi A. K., Mishra A., Jha B. (2016). Functional
Characterization of the
Tau Class Glutathione-S-Transferases Gene (SbGSTU) Promoter of Salicornia
brachiata under Salinity and Osmotic Stress. PLoS One.

[3035] Dóczi R., Csanaki C., Bánfalvi Z. (2002). Expression
and promoter activity of the desiccation-specific Solanum tuberosum
gene, StDS2. Plant, Cell & Environment.

[3036] Kumar S., Asif M. H., Chakrabarty D., Tripathi R. D., Dubey R. S., Trivedi P. K. (2015). Comprehensive analysis
of regulatory elements of the promoters of rice sulfate transporter
gene family and functional characterization of OsSul1;1 promoter under
different metal stress. Plant Signal Behav.

[3037] Yi N., Oh S. J., Kim Y. S., Jang H. J., Park S. H., Jeong J. S., Song S. I., Choi Y. D., Kim J. K. (2011). Analysis
of the Wsi 18, a stress-inducible promoter that is active in the whole
grain of transgenic rice. Transgenic Res.

[3038] Selvaraj M. G., Jan A., Ishizaki T., Valencia M., Dedicova B., Maruyama K., Ogata T., Todaka D., Yamaguchi-Shinozaki K., Nakashima K. (2020). Expression of the CCCH-tandem
zinc finger protein gene OsTZF5 under a stress-inducible promoter
mitigates the effect of drought stress on rice grain yield under field
conditions. Plant Biotechnol J.

[3039] Jeong H. J., Jung K. H. (2015). Rice tissue-specific promoters and
condition-dependent promoters for effective translational application. J Integr Plant Biol.

[3040] Liu J., Wang F., Yu G., Zhang X., Jia C., Qin J., Pan H. (2015). Functional
Analysis of the Maize C-Repeat/DRE Motif-Binding
Transcription Factor CBF3 Promoter in Response to Abiotic Stress. Int J Mol Sci.

[3041] Wu Y., Zhou H., Que Y. X., Chen R. K., Zhang M. Q. (2008). Cloning
and identification of promoter Prd29A and its application in sugarcane
drought resistance. Sugar Tech.

[3042] Hou L., Chen L., Wang J., Xu D., Dai L., Zhang H., Zhao Y. (2012). Construction of Stress
Responsive
Synthetic Promoters and Analysis of Their Activity in Transgenic Arabidopsis
thaliana. Plant Molecular Biology Reporter.

[3043] Sun Q., Gao F., Zhao L., Li K., Zhang J. (2010). Identification
of a new 130 bp cis-acting element in the TsVP1 promoter involved
in the salt stress response from Thellungiella halophila. BMC Plant Biol.

[3044] Grichko V. P., Glick B. R. (2001). Flooding tolerance of transgenic
tomato plants expressing the bacterial enzyme ACC deaminase controlledby
the 35S, rolD or PRB-1b promoter. Plant Physiology
and Biochemistry.

[3045] Xu W., Yu Y., Ding J., Hua Z., Wang Y. (2010). Characterization
of a novel stilbene synthase promoter involved in pathogen- and stress-inducible
expression from Chinese wild Vitis pseudoreticulata. Planta.

[3046] Havlova M., Dobrev P. I., Motyka V., Storchova H., Libus J., Dobra J., Malbeck J., Gaudinova A., Vankova R. (2008). The role of cytokinins in responses to water deficit
in tobacco plants over-expressing trans-zeatin O-glucosyltransferase
gene under 35S or SAG12 promoters. Plant Cell
Environ.

[3047] Kirch H. H., van Berkel J., Glaczinski H., Salamini F., Gebhardt C. (1997). Structural organization,
expression
and promoter activity of a cold-stress-inducible gene of potato (Solanum
tuberosum L.). Plant Mol Biol.

[3048] Belintani N. G., Guerzoni J. T. S., Moreira R. M. P., Vieira L. G. E. (2012). Improving
low-temperature tolerance in sugarcane by expressing the ipt gene
under a cold inducible promoter. Biologia Plantarum.

[3049] Elliott K. A., Shirsat A. H. (1998). Promoter regions of the extA extensin
gene from Brassica napus control activation in response to wounding
and tensile stress. Plant Mol Biol.

[3050] Yi N., Kim Y. S., Jeong M.-H., Oh S.-J., Jeong J. S., Park S.-H., Jung H., Choi Y. D., Kim J.-K. (2010). Functional
analysis of six drought-inducible promoters in transgenic rice plants
throughout all stages of plant growth. Planta.

[3051] Díaz-Martín J., Almoguera C. n., Prieto-Dapena P., Espinosa J. M., Jordano J. (2005). Functional
Interaction
between Two Transcription Factors Involved in the Developmental Regulation
of a Small Heat Stress Protein Gene Promoter. Plant Physiology.

[3052] Chen G., Hu J., Lian J., Zhang Y., Zhu L., Zeng D., Guo L., Yu L., Xu G., Qian Q. (2019). Functional characterization of OsHAK1
promoter in response to osmotic/drought
stress by deletion analysis in transgenic rice. Plant Growth Regulation.

[3053] Agarwal P. K., Shukla P. S., Gupta K., Jha B. (2013). Bioengineering
for salinity tolerance in plants: state of the art. Mol Biotechnol.

[3054] Yang S., Vanderbeld B., Wan J., Huang Y. (2010). Narrowing
down the targets: towards successful genetic engineering of drought-tolerant
crops. Mol Plant.

[3055] Yamaguchi T., Blumwald E. (2005). Developing salt-tolerant
crop plants:
challenges and opportunities. Trends Plant Sci.

[3056] Roy S. J., Negrao S., Tester M. (2014). Salt resistant crop
plants. Curr Opin Biotechnol.

[3057] Fang Y., Xiong L. (2015). General mechanisms
of drought response
and their application in drought resistance improvement in plants. Cell Mol Life Sci.

[3058] Tester M., Bacic A. (2005). Abiotic stress tolerance
in grasses.
From model plants to crop plants. Plant Physiol.

[3059] Thomashow M. F. (2001). So what's
new in the field of plant cold acclimation?
Lots!. Plant Physiol.

[3060] von Koskull-Doring P., Scharf K. D., Nover L. (2007). The diversity
of plant
heat stress transcription factors. Trends Plant
Sci.

[3061] Chen W. J., Zhu T. (2004). Networks of transcription factors
with roles in environmental stress response. Trends Plant Sci.

[3062] Maruyama K., Todaka D., Mizoi J., Yoshida T., Kidokoro S., Matsukura S., Takasaki H., Sakurai T., Yamamoto Y. Y., Yoshiwara K. (2012). Identification of cis-acting
promoter elements in cold- and dehydration-induced transcriptional
pathways in Arabidopsis, rice, and soybean. DNA Res.

[3063] Patro S., Kumar D., Ranjan R., Maiti I. B., Dey N. (2012). The development of efficient plant promoters for transgene expression
employing plant virus promoters. Mol Plant.

[3064] Zahur M., Maqbool A., Irfan M., Barozai M. Y., Qaiser U., Rashid B., Husnain T., Riazuddin S. (2009). Functional
analysis of cotton small heat shock protein promoter region in response
to abiotic stresses in tobacco using Agrobacterium-mediated transient
assay. Mol Biol Rep.

[3065] Perales L., Penarrubia L., Cornejo M. J. (2008). Induction of a polyubiquitin
gene promoter by dehydration stresses in transformed rice cells. J Plant Physiol.

[3066] Xu, Z. ; Wang, M. ; Guo, Z. ; Zhu, X. ; Xia, Z. Identification of a 119-bp Promoter of the Maize Sulfite Oxidase Gene (ZmSO) That Confers High-Level Gene Expression and ABA or Drought Inducibility in Transgenic Plants. Int J Mol Sci 2019, 20 (13), 3326 10.3390/ijms20133326. 31284569 PMC6651508

[3067] Liu H., Zhu K., Tan C., Zhang J., Zhou J., Jin L., Ma G., Zou Q. (2019). Identification and characterization
of PsDREB2 promoter involved in tissue-specific expression and abiotic
stress response from Paeonia suffruticosa. PeerJ.

[3068] Nakashima K., Ito Y., Yamaguchi-Shinozaki K. (2009). Transcriptional
regulatory networks in response to abiotic stresses in Arabidopsis
and grasses. Plant Physiol.

[3069] Shinozaki K., Yamaguchi-Shinozaki K. (2006). Gene networks
involved in drought
stress response and tolerance. J Exp Bot.

[3070] Huang G. T., Ma S. L., Bai L. P., Zhang L., Ma H., Jia P., Liu J., Zhong M., Guo Z. F. (2012). Signal
transduction during cold, salt, and drought stresses in plants. Mol Biol Rep.

[3071] Wang W., Vinocur B., Altman A. (2003). Plant responses to
drought, salinity and extreme temperatures: towards genetic engineering
for stress tolerance. Planta.

[3072] Valliyodan B., Nguyen H. T. (2006). Understanding regulatory
networks
and engineering for enhanced drought tolerance in plants. Curr Opin Plant Biol.

[3073] Lohani N., Singh M. B., Bhalla P. L. (2022). Biological
Parts
for Engineering Abiotic Stress Tolerance in Plants. Biodes Res.

[3074] Wani S. H., Kumar V., Khare T., Guddimalli R., Parveda M., Solymosi K., Suprasanna P., Kavi Kishor P. B. (2020). Engineering salinity tolerance in plants: progress
and prospects. Planta.

[3075] Zhang H., Zhu J., Gong Z., Zhu J. K. (2022). Abiotic
stress responses in plants. Nat Rev Genet.

[3076] Jahed K. R., Saini A. K., Sherif S. M. (2023). Coping with the
cold: unveiling cryoprotectants, molecular signaling pathways, and
strategies for cold stress resilience. Front
Plant Sci.

[3077] Teige M., Scheikl E., Eulgem T., Dóczi R., Ichimura K., Shinozaki K., Dangl J. L., Hirt H. (2004). The MKK2 pathway
mediates cold and salt stress signaling in Arabidopsis. Molecular Cell.

[3078] Qin L. X., Li Y., Li D. D., Xu W. L., Zheng Y., Li X. B. (2014). Arabidopsis drought-induced
protein
Di19-3 participates in plant response to drought and high salinity
stresses. Plant Mol Biol.

[3079] Belogurov G. A., Turkina M. V., Penttinen A., Huopalahti S., Baykov A. A., Lahti R. (2002). H+-pyrophosphatase
of Rhodospirillum rubrum. High yield expression in Escherichia coli
and identification of the Cys residues responsible for inactivation
my mersalyl. J Biol Chem.

[3080] Ono K., Hibino T., Kohinata T., Suzuki S., Tanaka Y., Nakamura T., Takabe T., Takabe T. (2001). Overexpression of DnaK
from a halotolerant cyanobacterium Aphanothece halophytica enhances
the high-temperatue tolerance of tobacco during germination and early
growth. Plant Sci.

[3081] Waltz E. (2014). Beating the heat. Nat Biotechnol.

[3082] Castiglioni P., Warner D., Bensen R. J., Anstrom D. C., Harrison J., Stoecker M., Abad M., Kumar G., Salvador S., D'Ordine R. (2008). Bacterial RNA chaperones
confer abiotic stress tolerance in plants and improved grain yield
in maize under water-limited conditions. Plant
Physiol.

[3083] Gupta B. K., Sahoo K. K., Ghosh A., Tripathi A. K., Anwar K., Das P., Singh A. K., Pareek A., Sopory S. K., Singla-Pareek S. L. (2018). Manipulation of glyoxalase pathway
confers tolerance to multiple stresses in rice. Plant Cell Environ.

[3084] Yeo E. T., Kwon H. B., Han S. E., Lee J. T., Ryu J. C., Byu M. O. (2000). Genetic engineering
of drought resistant
potato plants by introduction of the trehalose-6-phosphate synthase
(TPS1) gene from Saccharomyces cerevisiae. Mol
Cells.

[3085] Nunez-Munoz L., Vargas-Hernandez B., Hinojosa-Moya J., Ruiz-Medrano R., Xoconostle-Cazares B. (2021). Plant drought tolerance provided
through genome editing of the trehalase gene. Plant Signal Behav.

[3086] Garg A. K., Kim J. K., Owens T. G., Ranwala A. P., Choi Y. D., Kochian L. V., Wu R. J. (2002). Trehalose accumulation
in rice plants confers high tolerance levels to different abiotic
stresses. Proc Natl Acad Sci U S A.

[3087] Pilon-Smits E., Ebskamp M., Paul M. J., Jeuken M., Weisbeek P. J., Smeekens S. (1995). Improved Performance of Transgenic
Fructan-Accumulating Tobacco under Drought Stress. Plant Physiol.

[3088] Kawakami A., Sato Y., Yoshida M. (2008). Genetic engineering
of rice capable of synthesizing fructans and enhancing chilling tolerance. J Exp Bot.

[3089] Chen T. H., Murata N. (2002). Enhancement of tolerance of abiotic
stress by metabolic engineering of betaines and other compatible solutes. Curr Opin Plant Biol.

[3090] Hong Z., Lakkineni K., Zhang Z., Verma D. P. (2000). Removal
of feedback inhibition of delta(1)-pyrroline-5-carboxylate synthetase
results in increased proline accumulation and protection of plants
from osmotic stress. Plant Physiol.

[3091] Kretzschmar T., Pelayo M. A., Trijatmiko K. R., Gabunada L. F., Alam R., Jimenez R., Mendioro M. S., Slamet-Loedin I. H., Sreenivasulu N., Bailey-Serres J. (2015). A trehalose-6-phosphate
phosphatase enhances anaerobic germination
tolerance in rice. Nat Plants.

[3092] Fernandez O., Bethencourt L., Quero A., Sangwan R. S., Clement C. (2010). Trehalose and plant
stress responses: friend or foe?. Trends Plant
Sci.

[3093] Li H. W., Zang B. S., Deng X. W., Wang X. P. (2011). Overexpression
of the trehalose-6-phosphate synthase gene OsTPS1 enhances abiotic
stress tolerance in rice. Planta.

[3094] Kahraman M., Sevim G., Bor M. (2019). The role of proline,
glycinebetaine, and trehalose in stress-responsive gene expression. Osmoprotectant-mediated abiotic stress tolerance in plants:
recent advances and future perspectives.

[3095] Sawahel W. A., Hassan A. H. (2002). Generation of transgenic wheat plants
producing high levels of the osmoprotectant proline. Biotechnology Letters.

[3096] Annunziata M. G., Ciarmiello L. F., Woodrow P., Dell'Aversana E., Carillo P. (2019). Spatial and Temporal
Profile of Glycine Betaine Accumulation
in Plants Under Abiotic Stresses. Front Plant
Sci.

[3097] Quan R., Shang M., Zhang H., Zhao Y., Zhang J. (2004). Improved chilling tolerance by transformation
with betA gene for
the enhancement of glycinebetaine synthesis in maize. Plant Science.

[3098] Sakamoto A., Alia, Murata N. (1998). Metabolic engineering
of rice leading
to biosynthesis of glycinebetaine and tolerance to salt and cold. Plant Mol Biol.

[3099] Im Y. J., Ji M., Lee A., Killens R., Grunden A. M., Boss W. F. (2009). Expression of Pyrococcus
furiosus
superoxide reductase in Arabidopsis enhances heat tolerance. Plant Physiol.

[3100] Oki H., Kim S., Nakanishi H., Takahashi M., Yamaguchi H., Mori S., Nishizawa N. K. (2004). Directed
evolution of yeast ferric reductase to produce plants with tolerance
to iron deficiency in alkaline soils. Soil science
and plant nutrition.

[3101] Mora A., Earle E. (2001). Resistance to Alternaria
brassicicola
in transgenic broccoli expressing a Trichoderma harzianum endochitinase
gene. Molecular breeding.

[3102] Balasubramanian V., Vashisht D., Cletus J., Sakthivel N. (2012). Plant β-1,
3-glucanases: their biological functions and transgenic expression
against phytopathogenic fungi. Biotechnology
letters.

[3103] Lorito M., Woo S. L., Fernandez I. G., Colucci G., Harman G. E., Pintor-Toro J. A., Filippone E., Muccifora S., Lawrence C. B., Zoina A. (1998). Genes from
mycoparasitic fungi as a source for improving plant resistance to
fungal pathogens. Proceedings of the National
Academy of Sciences.

[3104] Girhepuje P., Shinde G. (2011). Transgenic tomato plants
expressing
a wheat endochitinase gene demonstrate enhanced resistance to Fusarium
oxysporum f. sp. lycopersici. Plant Cell, Tissue
and Organ Culture (PCTOC).

[3105] Distefano G., La Malfa S., Vitale A., Lorito M., Deng Z., Gentile A. (2008). Defence-related gene expression in
transgenic lemon plants producing an antimicrobial Trichoderma harzianum
endochitinase during fungal infection. Transgenic
research.

[3106] Mishra M., Jalil S. U., Mishra R. K., Kumari S., Pandey B. K. (2016). In vitro
screening of guava plantlets transformed with
endochitinase gene against Fusarium oxysporum f. sp. psidii. Czech Journal of Genetics and Plant Breeding.

[3107] Sher Khan R., Iqbal A., Malak R., Shehryar K., Attia S., Ahmed T., Ali Khan M., Arif M., Mii M. (2019). Plant defensins: types, mechanism
of action and prospects of genetic
engineering for enhanced disease resistance in plants. 3 Biotech.

[3108] Zhu Y. J., Agbayani R., Moore P. H. (2007). Ectopic
expression
of Dahlia merckii defensin DmAMP1 improves papaya resistance to Phytophthora
palmivora by reducing pathogen vigor. Planta.

[3109] Seo H. H., Park S., Park S., Oh B. J., Back K., Han O., Kim J. I., Kim Y. S. (2014). Overexpression
of a defensin enhances resistance to a fruit-specific anthracnose
fungus in pepper. PLoS One.

[3110] Abel P. P., Nelson R. S., De B., Hoffmann N., Rogers S. G., Fraley R. T., Beachy R. N. (1986). Delay of
disease
development in transgenic plants that express the tobacco mosaic virus
coat protein gene. Science.

[3111] Khalid A., Zhang Q., Yasir M., Li F. (2017). Small RNA
Based Genetic Engineering for Plant Viral Resistance: Application
in Crop Protection. Front Microbiol.

[3112] van Esse H. P., Reuber T. L., van der
Does D. (2020). Genetic modification
to improve disease resistance in crops. New
Phytol.

[3113] Azad M. A., Amin L., Sidik N. M. (2014). Gene technology
for papaya ringspot virus disease management. ScientificWorldJournal.

[3114] Fitch M. M. M., Manshardt R. M., Gonsalves D., Slightom J. L., Sanford J. C. (1992). Virus Resistant
Papaya Plants Derived
from Tissues Bombarded with the Coat Protein Gene of Papaya Ringspot
Virus. Bio/Technology.

[3115] Escobar M. A., Civerolo E. L., Summerfelt K. R., Dandekar A. M. (2001). RNAi-mediated oncogene silencing confers resistance
to crown gall tumorigenesis. Proc Natl Acad
Sci U S A.

[3116] Koch A., Kumar N., Weber L., Keller H., Imani J., Kogel K. H. (2013). Host-induced gene silencing of cytochrome
P450 lanosterol C14alpha-demethylase-encoding genes confers strong
resistance to Fusarium species. Proc Natl Acad
Sci U S A.

[3117] Nitnavare R. B., Bhattacharya J., Singh S., Kour A., Hawkesford M. J., Arora N. (2021). Next Generation dsRNA-Based Insect
Control: Success So Far and Challenges. Front
Plant Sci.

[3118] Zhang J., Khan S. A., Heckel D. G., Bock R. (2017). Next-Generation
Insect-Resistant Plants: RNAi-Mediated Crop Protection. Trends Biotechnol.

[3119] Khajuria C., Ivashuta S., Wiggins E., Flagel L., Moar W., Pleau M., Miller K., Zhang Y., Ramaseshadri P., Jiang C. (2018). Development
and characterization
of the first dsRNA-resistant insect population from western corn rootworm,
Diabrotica virgifera virgifera LeConte. PLoS
One.

[3120] Mao Y. B., Cai W. J., Wang J. W., Hong G. J., Tao X. Y., Wang L. J., Huang Y. P., Chen X. Y. (2007). Silencing
a cotton bollworm P450 monooxygenase gene by plant-mediated RNAi impairs
larval tolerance of gossypol. Nat Biotechnol.

[3121] Ji X., Zhang H., Zhang Y., Wang Y., Gao C. (2015). Establishing
a CRISPR-Cas-like immune system conferring DNA virus resistance in
plants. Nat Plants.

[3122] Baltes, N. J. ; Hummel, A. W. ; Konecna, E. ; Cegan, R. ; Bruns, A. N. ; Bisaro, D. M. ; Voytas, D. F. Conferring resistance to geminiviruses with the CRISPR-Cas prokaryotic immune system. Nat Plants 2015, 1 (10), 10.1038/nplants.2015.145. PMC8612103 34824864

[3123] Yan S., Ren B., Zeng B., Shen J. (2020). Improving RNAi efficiency
for pest control in crop species. Biotechniques.

[3124] Numan M., Bashir S., Khan Y., Mumtaz R., Shinwari Z. K., Khan A. L., Khan A., Ahmed A.-H. (2018). Plant growth
promoting bacteria as an alternative strategy for salt tolerance in
plants: a review. Microbiological Research.

[3125] Egamberdieva D. (2009). Alleviation of salt stress by plant
growth regulators
and IAA producing bacteria in wheat. Acta Physiologiae
Plantarum.

[3126] Nautiyal C. S., Srivastava S., Chauhan P. S., Seem K., Mishra A., Sopory S. K. (2013). Plant growth-promoting bacteria Bacillus
amyloliquefaciens NBRISN13 modulates gene expression profile of leaf
and rhizosphere community in rice during salt stress. Plant Physiology and Biochemistry.

[3127] Morsy M. R., Oswald J., He J., Tang Y., Roossinck M. J. (2010). Teasing apart a three-way symbiosis:
transcriptome
analyses of Curvularia protuberata in response to viral infection
and heat stress. Biochem Biophys Res Commun.

[3128] Marquez L. M., Redman R. S., Rodriguez R. J., Roossinck M. J. (2007). A virus
in a fungus in a plant: three-way symbiosis
required for thermal tolerance. Science.

[3129] Kerr, A. ; Bullard, G. Biocontrol of Crown Gall by Rhizobium rhizogenes: Challenges in Biopesticide Commercialisation. Agronomy-Basel 2020, 10 (8), 1126. 10.3390/agronomy10081126

[3130] Kerr, A. ; Jones, D. A. ; Clare, B. G. ; Ryder, M. H. ; Farrand, S. K. Agrobacterium radiobacter K84 carrying transfer-deficient pAgK84 plasmids. US5354684A, 1988.

[3131] Timms-Wilson T. M., Ellis R. J., Renwick A., Rhodes D. J., Mavrodi D. V., Weller D. M., Thomashow L. S., Bailey M. J. (2000). Chromosomal Insertion of Phenazine-1-Carboxylic
Acid
Biosynthetic Pathway Enhances Efficacy of Damping-off Disease Control
by Pseudomonas fluorescens. MPMI.

[3132] Huang Z., Bonsall R. F., Mavrodi D. V., Weller D. M., Thomashow L. S. (2004). Transformation
of Pseudomonas fluorescens with genes
for biosynthesis of phenazine-1-carboxylic acid improves biocontrol
of rhizoctonia root rot and in situ antibiotic production. FEMS Microbiol Ecol.

[3133] Thomashow L. S., Weller D. M. (1988). Role of a phenazine
antibiotic from
Pseudomonas fluorescens in biological control of Gaeumannomyces graminis
var. tritici. J Bacteriol.

[3134] Pierson L. S., Thomashow L. S. (1992). Cloning
and heterologous expression of the phenazine biosynthetic locus from
Pseudomonas aureofaciens 30-84. Mol Plant Microbe
Interact.

[3135] Bankhead S. B., Landa B. B., Lutton E., Weller D. M., Gardener B. B. M. (2004). Minimal
changes in rhizobacterial population structure
following root colonization by wild type and transgenic biocontrol
strains. FEMS Microbiol Ecol.

[3136] Patel J. K., Archana G. (2018). Engineered production
of 2,4-diacetylphloroglucinol
in the diazotrophic endophytic bacterium Pseudomonas sp WS5 and its
beneficial effect in multiple plant-pathogen systems. Appl Soil Ecol.

[3137] Zhang J. B., Mavrodi D. V., Yang M. M., Thomashow L. S., Mavrodi O. V., Kelton J., Weller D. M. (2020). Pseudomonas synxantha
2-79 Transformed with Pyrrolnitrin Biosynthesis Genes Has Improved
Biocontrol Activity Against Soilborne Pathogens of Wheat and Canola. Phytopathology.

[3138] Leclere V., Bechet M., Adam A., Guez J. S., Wathelet B., Ongena M., Thonart P., Gancel F., Chollet-Imbert M., Jacques P. (2005). Mycosubtilin overproduction
by Bacillus
subtilis BBG100 enhances the organism's antagonistic and biocontrol
activities. Appl Environ Microb.

[3139] Zhang L., Ruan L., Hu C., Wu H., Chen S., Yu Z., Sun M. (2007). Fusion of the genes
for AHL-lactonase and S-layer protein in Bacillus thuringiensis increases
its ability to inhibit soft rot caused by Erwinia carotovora. Appl Microbiol Biotechnol.

[3140] Li Q., Ni H., Meng S., He Y., Yu Z., Li L. (2011). Suppressing Erwinia carotovora pathogenicity
by projecting N-acyl
homoserine lactonase onto the surface of Pseudomonas putida cells. J Microbiol Biotechnol.

[3141] Ma M., Du H., Sun T., An S., Yang G., Wang D. (2019). Characteristics of archaea and bacteria
in rice rhizosphere along
a mercury gradient. Sci Total Environ.

[3142] Song G. C., Im H., Jung J., Lee S., Jung M. Y., Rhee S. K., Ryu C. M. (2019). Plant growth-promoting
archaea trigger induced systemic resistance in Arabidopsis thaliana
against Pectobacterium carotovorum and Pseudomonas syringae. Environ Microbiol.

[3143] Park S.-Y., Peterson F. C., Mosquna A., Yao J., Volkman B. F., Cutler S. R. (2015). Agrochemical control of plant water
use using engineered abscisic acid receptors. Nature.

[3144] Bittner A., Ciesla A., Gruden K., Lukan T., Mahmud S., Teige M., Vothknecht U. C., Wurzinger B. (2022). Organelles and phytohormones: a network of interactions
in plant stress responses. J Exp Bot.

[3145] Hossain Z., Nouri M. Z., Komatsu S. (2012). Plant cell organelle
proteomics in response to abiotic stress. J
Proteome Res.

[3146] Dopp I. J., Yang X., Mackenzie S. A. (2021). A new take
on organelle-mediated stress sensing in plants. New Phytol.

[3147] Zhou X., Wang G., Sutoh K., Zhu J. K., Zhang W. (2008). Identification
of cold-inducible microRNAs in plants by transcriptome
analysis. Biochim Biophys Acta.

[3148] Fang P., Hu Y., Xia W., Wu X., Sun T., Pandey A. K., Ning K., Zhu C., Xu P. (2023). Transcriptome
Dynamics of Common Bean Roots Exposed to Various Heavy Metals Reveal
Valuable Target Genes and Promoters for Genetic Engineering. J Agric Food Chem.

[3149] Seki M., Narusaka M., Abe H., Kasuga M., Yamaguchi-Shinozaki K., Carninci P., Hayashizaki Y., Shinozaki K. (2001). Monitoring the expression pattern of 1300 Arabidopsis
genes under drought and cold stresses by using a full-length cDNA
microarray. Plant Cell.

[3150] Danquah A., de Zelicourt A., Colcombet J., Hirt H. (2014). The role of ABA and MAPK signaling
pathways in plant abiotic stress
responses. Biotechnol Adv.

[3151] Sah S. K., Reddy K. R., Li J. (2016). Abscisic Acid
and Abiotic
Stress Tolerance in Crop Plants. Front Plant
Sci.

[3152] Wasilewska A., Vlad F., Sirichandra C., Redko Y., Jammes F., Valon C., Frei dit
Frey N., Leung J. (2008). An update on abscisic acid signaling in plants and
more. Mol Plant.

[3153] Nakashima K., Yamaguchi-Shinozaki K. (2013). ABA signaling
in stress-response
and seed development. Plant Cell Rep.

[3154] Mur L. A., Prats E., Pierre S., Hall M. A., Hebelstrup K. H. (2013). Integrating
nitric oxide into salicylic acid and jasmonic
acid/ ethylene plant defense pathways. Front
Plant Sci.

[3155] Caarls L., Pieterse C. M., Van Wees S. C. (2015). How salicylic
acid
takes transcriptional control over jasmonic acid signaling. Front Plant Sci.

[3156] Liu L., Sonbol F.-M., Huot B., Gu Y., Withers J., Mwimba M., Yao J., He S. Y., Dong X. (2016). Salicylic
acid receptors activate jasmonic acid signalling through a non-canonical
pathway to promote effector-triggered immunity. Nature Communications.

[3157] An C., Mou Z. (2011). Salicylic acid and its function in plant immunity F. Journal of integrative plant biology.

[3158] Campos M. L., Kang J. H., Howe G. A. (2014). Jasmonate-triggered
plant immunity. J Chem Ecol.

[3159] Morgan P. W., Drew M. C. (1997). Ethylene and plant
responses to stress. Physiologia Plantarum.

[3160] Baharudin N. F., Osman N. I. (2023). Plant development, stress responses,
and secondary metabolism under ethylene regulation. Plant Stress.

[3161] Jibran R., A Hunter D., P Dijkwel P. (2013). Hormonal regulation
of leaf senescence through integration of developmental and stress
signals. Plant Molecular Biology.

[3162] Barry C. S., Giovannoni J. J. (2007). Ethylene
and fruit ripening. Journal of Plant Growth
Regulation.

[3163] Hartman S., Sasidharan R., Voesenek L. (2021). The role of ethylene
in metabolic acclimations to low oxygen. New
Phytol.

[3164] Torres M. A., Jones J. D., Dangl J. L. (2006). Reactive oxygen
species signaling in response to pathogens. Plant Physiol.

[3165] Khan M., Ali S., Al Azzawi T. N. I., Saqib S., Ullah F., Ayaz A., Zaman W. (2023). The key roles
of ROS and RNS as a signaling molecule in plant-microbe interactions. Antioxidants.

[3166] Das K., Roychoudhury A. (2014). Reactive oxygen species (ROS) and response of antioxidants
as ROS-scavengers during environmental stress in plants. Frontiers in Environmental Science.

[3167] Hasanuzzaman, M. ; Bhuyan, M. ; Zulfiqar, F. ; Raza, A. ; Mohsin, S. M. ; Mahmud, J. A. ; Fujita, M. ; Fotopoulos, V. Reactive Oxygen Species and Antioxidant Defense in Plants under Abiotic Stress: Revisiting the Crucial Role of a Universal Defense Regulator. Antioxidants (Basel) 2020, 9 (8), 681 10.3390/antiox9080681. 32751256 PMC7465626

[3168] Sachdev, S. ; Ansari, S. A. ; Ansari, M. I. ; Fujita, M. ; Hasanuzzaman, M. Abiotic Stress and Reactive Oxygen Species: Generation, Signaling, and Defense Mechanisms. Antioxidants (Basel) 2021, 10 (2), 277 10.3390/antiox10020277. 33670123 PMC7916865

[3169] Dumanovic J., Nepovimova E., Natic M., Kuca K., Jacevic V. (2021). The Significance of
Reactive Oxygen Species and Antioxidant
Defense System in Plants: A Concise Overview. Front Plant Sci.

[3170] Yuan, P. ; Yang, T. ; Poovaiah, B. W. Calcium Signaling-Mediated Plant Response to Cold Stress. Int J Mol Sci 2018, 19 (12), 3896 10.3390/ijms19123896. 30563125 PMC6320992

[3171] Hong-Bo S., Li-Ye C., Ming-An S. (2008). Calcium as
a versatile
plant signal transducer under soil water stress. BioEssays.

[3172] Zhang L., Du L., Poovaiah B. W. (2014). Calcium signaling
and biotic defense responses in plants. Plant
Signal Behav.

[3173] Aldon, D. ; Mbengue, M. ; Mazars, C. ; Galaud, J. P. Calcium Signalling in Plant Biotic Interactions. Int J Mol Sci 2018, 19 (3), 665 10.3390/ijms19030665. 29495448 PMC5877526

[3174] Song W.-Y., Zhang Z.-B., Shao H.-B., Guo X.-L., Cao H.-X., Zhao H.-B., Fu Z.-Y., Hu X.-J. (2008). Relationship
between calcium decoding elements and plant abiotic-stress resistance. International Journal of Biological Sciences.

[3175] Kunkel B. N., Brooks D. M. (2002). Cross talk between
signaling pathways
in pathogen defense. Current opinion in plant
biology.

[3176] Koornneef A., Pieterse C. M. (2008). Cross talk in defense signaling. Plant physiology.

[3177] Chinnusamy V., Schumaker K., Zhu J. K. (2004). Molecular genetic
perspectives on cross-talk and specificity in abiotic stress signalling
in plants. Journal of Experimental Botany.

[3178] Nakashima K., Yamaguchi-Shinozaki K., Shinozaki K. (2014). The transcriptional
regulatory network in the drought response and its crosstalk in abiotic
stress responses including drought, cold, and heat. Front Plant Sci.

[3179] Walther D., Brunnemann R., Selbig J. (2007). The regulatory code
for transcriptional response diversity and its relation to genome
structural properties in A. thaliana. PLoS Genet.

[3180] Ali S., Kim W. C. (2019). A Fruitful Decade
Using Synthetic Promoters in the
Improvement of Transgenic Plants. Front Plant
Sci.

[3181] Dey N., Sarkar S., Acharya S., Maiti I. B. (2015). Synthetic promoters
in planta. Planta.

[3182] Fethe M. H., Liu W., Burris J. N., Millwood R. J., Mazarei M., Rudis M. R., Yeaman D. G., Dubosquielle M., Stewart C. N. (2014). The performance
of pathogenic bacterial
phytosensing transgenic tobacco in the field. Plant Biotechnol J.

[3183] Persad-Russell R., Mazarei M., Schimel T. M., Howe L., Schmid M. J., Kakeshpour T., Barnes C. N., Brabazon H., Seaberry E. M., Reuter D. N. (2022). Specific bacterial pathogen phytosensing
is enabled by a synthetic promoter-transcription factor system in
potato. Frontiers in Plant Science.

[3184] Liu W., Mazarei M., Rudis M. R., Fethe M. H., Peng Y., Millwood R. J., Schoene G., Burris J. N., Stewart C. N. (2013). Bacterial pathogen
phytosensing in transgenic tobacco
and Arabidopsis plants. Plant Biotechnol J.

[3185] Boni R., Chauhan H., Hensel G., Roulin A., Sucher J., Kumlehn J., Brunner S., Krattinger S. G., Keller B. (2018). Pathogen-inducible Ta-Lr34res expression in heterologous
barley confers disease resistance without negative pleiotropic effects. Plant biotechnology journal.

[3186] Maeda S., Goto S., Inoue H., Suwazono H., Takatsuji H., Mori M. (2024). Improvement of Broad-Spectrum
Disease-Resistant
Rice by the Overexpression of BSR1 via a Moderate-Strength Constitutive
Promoter and a Pathogen-Inducible Promoter. Plants.

[3187] Liu M., Shi Z., Zhang X., Wang M., Zhang L., Zheng K., Liu J., Hu X., Di C., Qian Q. (2019). Inducible overexpression
of Ideal Plant Architecture1 improves both
yield and disease resistance in rice. Nature
plants.

[3188] Coutos-Thevenot P., Poinssot B., Bonomelli A., Yean H., Breda C., Buffard D., Esnault R., Hain R., Boulay M. (2001). In vitro tolerance to Botrytis cinerea
of grapevine 41B rootstock in transgenic plants expressing the stilbene
synthase Vst1 gene under the control of a pathogen-inducible PR 10
promoter. J Exp Bot.

[3189] Cai M., Qiu D., Yuan T., Ding X., Li H., Duan L., Xu C., Li X., Wang S. (2008). Identification
of novel pathogen-responsive cis-elements and their binding proteins
in the promoter of OsWRKY13, a gene regulating rice disease resistance. Plant, cell & environment.

[3190] Zou X., Song E., Peng A., He Y., Xu L., Lei T., Yao L., Chen S. (2014). Activation
of three pathogen-inducible
promoters in transgenic citrus (Citrus sinensis Osbeck) after Xanthomonas
axonopodis pv. citri infection and wounding. Plant Cell, Tissue and Organ Culture (PCTOC).

[3191] Malnoy M., Reynoird J., Borejsza-Wysocka E., Aldwinckle H. (2006). Activation of the pathogen-inducible Gst1 promoter
of potato after elicitation by Venturia inaequalis and Erwinia amylovora
in transgenic apple (Malus× domestica). Transgenic research.

[3192] Pühringer H., Moll D., Hoffmann-Sommergruber K., Watillon B., Katinger H., da Câmara Machado M. L. (2000). The promoter
of an apple Ypr10 gene, encoding the major allergen Mal d 1, is stress-and
pathogen-inducible. Plant Science.

[3193] Manners J. M., Penninckx I. A., Vermaere K., Kazan K., Brown R. L., Morgan A., Maclean D. J., Curtis M. D., Cammue B. P., Broekaert W. F. (1998). The promoter
of the plant defensin
gene PDF1. 2 from Arabidopsis is systemically activated by fungal
pathogens and responds to methyl jasmonate but not to salicylic acid. Plant molecular biology.

[3194] Baruah I., Baldodiya G. M., Sahu J., Baruah G. (2020). Dissecting
the role of promoters of pathogen-sensitive genes in plant defense. Current Genomics.

[3195] Wu J., Reca I. B., Spinelli F., Lironi D., De Lorenzo G., Poltronieri P., Cervone F., Joosten M. H., Ferrari S., Brutus A. (2019). An EFR-Cf-9
chimera confers enhanced resistance to
bacterial pathogens by SOBIR1-and BAK1-dependent recognition of elf18. Molecular plant pathology.

[3196] Albert M., Jehle A. K., Mueller K., Eisele C., Lipschis M., Felix G. (2010). Arabidopsis thaliana
pattern recognition
receptors for bacterial elongation factor Tu and flagellin can be
combined to form functional chimeric receptors. Journal of Biological Chemistry.

[3197] Romer P., Recht S., Lahaye T. (2009). A single plant
resistance
gene promoter engineered to recognize multiple TAL effectors from
disparate pathogens. Proc Natl Acad Sci U S
A.

[3198] Streubel J., Baum H., Grau J., Stuttman J., Boch J. (2017). Dissection of TALE-dependent gene activation reveals that they induce
transcription cooperatively and in both orientations. PloS one.

[3199] de Lange O., Schreiber T., Schandry N., Radeck J., Braun K. H., Koszinowski J., Heuer H., Strauss A., Lahaye T. (2013). Breaking the DNA-binding
code of Ralstonia solanacearum
TAL effectors provides new possibilities to generate plant resistance
genes against bacterial wilt disease. New Phytol.

[3200] Boch J., Bonas U. (2010). Xanthomonas AvrBs3 family-type III
effectors: discovery and function. Annual review
of phytopathology.

[3201] Römer P., Recht S., Strauß T., Elsaesser J., Schornack S., Boch J., Wang S., Lahaye T. (2010). Promoter elements of rice susceptibility genes are
bound and activated by specific TAL effectors from the bacterial blight
pathogen, Xanthomonas oryzae pv. oryzae. New
Phytologist.

[3202] Mazarei M., Teplova I., Hajimorad M. R., Stewart C. N. (2008). Pathogen phytosensing: plants to
report plant pathogens. Sensors.

[3203] Huang Z., Peng S., Li H., Zeng F. (2017). Transcriptional
properties of eight synthetic pathogen-inducible promoters in transgenic
Arabidopsis thaliana. Biologia plantarum.

[3204] Fahad S., Nie L., Chen Y., Wu C., Xiong D., Saud S., Hongyan L., Cui K., Huang J. (2015). Crop plant hormones
and environmental stress. Sustainable Agriculture
Reviews: Volume 15.

[3205] Verma V., Ravindran P., Kumar P. P. (2016). Plant hormone-mediated
regulation of stress responses. BMC Plant Biol.

[3206] Osakabe Y., Watanabe T., Sugano S. S., Ueta R., Ishihara R., Shinozaki K., Osakabe K. (2016). Optimization of CRISPR/Cas9
genome editing to modify abiotic stress responses in plants. Sci Rep.

[3207] Geddes B. A., Paramasivan P., Joffrin A., Thompson A. L., Christensen K., Jorrin B., Brett P., Conway S. J., Oldroyd G. E. D., Poole P. S. (2019). Engineering transkingdom signalling
in plants to control gene expression in rhizosphere bacteria. Nat Commun.

[3208] Ge, H. ; Li, X. ; Chen, S. ; Zhang, M. ; Liu, Z. ; Wang, J. ; Li, X. ; Yang, Y. The Expression of CARK1 or RCAR11 Driven by Synthetic Promoters Increases Drought Tolerance in Arabidopsis thaliana. Int J Mol Sci 2018, 19 (7), 1945 10.3390/ijms19071945. 29970817 PMC6073707

[3209] Wang D., Yang Z., Feng M., Yang W., Qu R., Nie S. (2024). The overexpression of SlBRI1 driven by Atrd29A promoter-transgenic
plants improves the chilling stress tolerance of tomato. Planta.

[3210] Pino M. T., Skinner J. S., Park E. J., Jeknić Z., Hayes P. M., Thomashow M. F., Chen T. H. (2007). Use of a stress
inducible promoter to drive ectopic AtCBF expression improves potato
freezing tolerance while minimizing negative effects on tuber yield. Plant Biotechnology Journal.

[3211] Qiu W., Liu M., Qiao G., Jiang J., Xie L., Zhuo R. (2012). An isopentyl transferase
gene driven by the stress-inducible rd29A
promoter improves salinity stress tolerance in transgenic tobacco. Plant molecular biology reporter.

[3212] Kovalchuk N., Jia W., Eini O., Morran S., Pyvovarenko T., Fletcher S., Bazanova N., Harris J., Beck-Oldach K., Shavrukov Y. (2013). Optimization of TaDREB3
gene expression in transgenic barley using cold-inducible promoters. Plant Biotechnol J.

[3213] Jayaraman K., Sevanthi A. M., Sivakumar S., Viswanathan C., Mohapatra T., Mandal P. K. (2021). Stress-inducible
expression of chalcone isomerase2 gene improves accumulation of flavonoids
and imparts enhanced abiotic stress tolerance to rice. Environmental and Experimental Botany.

[3214] Hinchee, M. ; Zhang, C. ; Chang, S. ; Cunningham, M. ; Hammond, W. ; Nehra, N. Biotech Eucalyptus can sustainably address society’s need for wood: the example of freeze tolerant Eucalyptus in the southeastern US. In BMC Proceedings; 2011; Springer: Vol. 5, p I24.

[3215] Hinchee M., Rottmann W., Mullinax L., Zhang C., Chang S., Cunningham M., Pearson L., Nehra N. (2011). Short-rotation
woody crops for bioenergy and biofuels applications. Biofuels: Global impact on renewable energy, production agriculture,
and technological advancements.

[3216] Su J., Wu R. (2004). Stress-inducible synthesis of proline in transgenic
rice confers faster growth under stress conditions than that with
constitutive synthesis. Plant Science.

[3217] Kim J. M., To T. K., Matsui A., Tanoi K., Kobayashi N. I., Matsuda F., Habu Y., Ogawa D., Sakamoto T., Matsunaga S. (2017). Acetate-mediated novel
survival strategy against drought in plants. Nat Plants.

[3218] Rasheed S., Bashir K., Kim J. M., Ando M., Tanaka M., Seki M. (2018). The modulation of acetic
acid pathway
genes in Arabidopsis improves survival under drought stress. Sci Rep.

[3219] Kasuga M., Liu Q., Miura S., Yamaguchi-Shinozaki K., Shinozaki K. (1999). Improving plant drought, salt, and freezing tolerance
by gene transfer of a single stress-inducible transcription factor. Nat Biotechnol.

[3220] Brophy J. A. N. (2022). Toward synthetic plant development. Plant Physiol.

[3221] Zhang H., Zhao Y., Zhu J. K. (2020). Thriving
under Stress:
How Plants Balance Growth and the Stress Response. Dev Cell.

[3222] Rivero R. M., Kojima M., Gepstein A., Sakakibara H., Mittler R., Gepstein S., Blumwald E. (2007). Delayed leaf senescence
induces extreme drought tolerance in a flowering plant. Proc Natl Acad Sci U S A.

[3223] Brophy J. A. N., LaRue T., Dinneny J. R. (2018). Understanding
and
engineering plant form. Semin Cell Dev Biol.

[3224] Kim I.-J., Lee J., Han J.-A., Kim C.-S., Hur Y. (2011). Citrus Lea promoter confers fruit-preferential
and stressinducible
gene expression in Arabidopsis. Canadian journal
of plant science.

[3225] Alok A., Kaur J., Tiwari S. (2020). Functional characterization
of wheat myo-inositol oxygenase promoter under different abiotic stress
conditions in Arabidopsis thaliana. Biotechnol
Lett.

[3226] Yamaguchi-Shinozaki K., Shinozaki K. (1993). Characterization
of the expression
of a desiccation-responsive rd29 gene of Arabidopsis thaliana and
analysis of its promoter in transgenic plants. Mol Gen Genet.

[3227] Msanne J., Lin J., Stone J. M., Awada T. (2011). Characterization
of abiotic stress-responsive Arabidopsis thaliana RD29A and RD29B
genes and evaluation of transgenes. Planta.

[3228] Walley J. W., Coughlan S., Hudson M. E., Covington M. F., Kaspi R., Banu G., Harmer S. L., Dehesh K. (2007). Mechanical
stress induces biotic and abiotic stress responses via a novel cis-element. PLoS Genet.

[3229] Santos E., Remy S., Thiry E., Windelinckx S., Swennen R., Sagi L. (2009). Characterization and isolation of
a T-DNA tagged banana promoter active during in vitro culture and
low temperature stress. BMC Plant Biol.

[3230] Yang Y., Al-Baidhani H. H. J., Harris J., Riboni M., Li Y., Mazonka I., Bazanova N., Chirkova L., Sarfraz
Hussain S., Hrmova M. (2020). DREB/CBF expression
in wheat and barley using the stress-inducible promoters of HD-Zip
I genes: impact on plant development, stress tolerance and yield. Plant Biotechnol J.

[3231] Tao Y., Wang F., Jia D., Li J., Zhang Y., Jia C., Wang D., Pan H. (2015). Cloning and
Functional Analysis of
the Promoter of a Stress-inducible Gene (ZmRXO1) in Maize. Plant Molecular Biology Reporter.

[3232] Lin C. H., Chen C. Y. (2017). The pathogen-inducible
promoter of
defense-related LsGRP1 gene from Lilium functioning in phylogenetically
distinct species of plants. Plant Sci.

[3233] Zhu W., Zhang D., Lu X., Zhang L., Yu Z., Lv H., Zhang H. (2014). Characterisation
of an SKn-type Dehydrin Promoter from
Wheat and Its Responsiveness to Various Abiotic and Biotic Stresses. Plant Molecular Biology Reporter.

[3234] Dang H. Q., Tran N. Q., Tuteja R., Tuteja N. (2011). Promoter of
a salinity and cold stress-induced MCM6 DNA helicase from pea. Plant Signal Behav.

[3235] Yang Y., Tagaloguin P., Chaffin T. A., Shao Y., Mazarei M., Millwood R. J., Stewart C. N. (2024). Drought stress-inducible synthetic
promoters designed for poplar
are functional in rice. Plant Cell Rep.

[3236] Yang Y., Lee J. H., Poindexter M. R., Shao Y., Liu W., Lenaghan S. C., Ahkami A. H., Blumwald E., Stewart C. N. (2021). Rational design
and testing of abiotic stress-inducible synthetic promoters from poplar
cis-regulatory elements. Plant Biotechnol J.

[3237] Delatorre C. A., Cohen Y., Liu L., Peleg Z., Blumwald E. (2012). The regulation
of the SARK promoter activity by hormones
and environmental signals. Plant Sci.

[3238] Decima Oneto C., Otegui M. E., Baroli I., Beznec A., Faccio P., Bossio E., Blumwald E., Lewi D. (2016). Water deficit
stress tolerance in maize conferred by expression of an isopentenyltransferase
(IPT) gene driven by a stress- and maturation-induced promoter. J Biotechnol.

[3239] Bihmidine S., Lin J., Stone J. M., Awada T., Specht J. E., Clemente T. E. (2013). Activity of the Arabidopsis RD29A
and RD29B promoter elements in soybean under water stress. Planta.

[3240] Yamaguchi-Shinozaki K., Shinozaki K. (1994). A novel cis-acting
element in an
Arabidopsis gene is involved in responsiveness to drought, low-temperature,
or high-salt stress. The Plant Cell.

[3241] Estrada-Melo A. C., Chao, Reid M. S., Jiang C. Z. (2015). Overexpression
of
an ABA biosynthesis gene using a stress-inducible promoter enhances
drought resistance in petunia. Hortic Res.

[3242] Yang Y., Chaffin T. A., Shao Y., Balasubramanian V. K., Markillie M., Mitchell H., Rubio-Wilhelmi M. M., Ahkami A. H., Blumwald E., Neal Stewart C. (2024). Novel synthetic inducible promoters controlling gene
expression during water-deficit stress with green tissue specificity
in transgenic poplar. Plant Biotechnol J.

[3243] Rai M., He C., Wu R. (2009). Comparative functional analysis of
three abiotic stress-inducible promoters in transgenic rice. Transgenic Research.

[3244] Ono A., Izawa T., Chua N. H., Shimamoto K. (1996). The rab16B
Promoter of Rice Contains Two Distinct Abscisic Acid-Responsive Elements. Plant Physiology.

[3245] Lee S.-C., Kim S.-H., Kim S.-R. (2013). Drought
inducible
OsDhn1 promoter is activated by OsDREB1A and OsDREB1D. Journal of Plant Biology.

[3246] Su J., Shen Q., David Ho T. H., Wu R. (1998). Dehydration-stress-regulated
transgene expression in stably transformed rice plants. Plant Physiol.

[3247] Raikwar S., Srivastava V. K., Gill S. S., Tuteja R., Tuteja N. (2015). Emerging Importance
of Helicases in Plant Stress Tolerance:
Characterization of Oryza sativa Repair Helicase XPB2 Promoter and
Its Functional Validation in Tobacco under Multiple Stresses. Front Plant Sci.

[3248] Saidi Y., Finka A., Chakhporanian M., Zryd J. P., Schaefer D. G., Goloubinoff P. (2005). Controlled
expression of recombinant proteins in Physcomitrella patens by a conditional
heat-shock promoter: a tool for plant research and biotechnology. Plant Mol Biol.

[3249] Rerksiri W., Zhang X., Xiong H., Chen X. (2013). Expression
and promoter analysis of six heat stress-inducible genes in rice. ScientificWorldJournal.

[3250] Yi S. Y., Sun A. Q., Sun Y., Yang J. Y., Zhao C. M., Liu J. (2006). Differential regulation of Lehsp23.8
in tomato plants: Analysis of a multiple stress-inducible promoter. Plant Sci.

[3251] Dong C. J., Wang Y., Yu S. S., Liu J. Y. (2010). Characterization
of a novel rice metallothionein gene promoter: its tissue specificity
and heavy metal responsiveness. J Integr Plant
Biol.

[3252] Zahur M., Maqbool A., Ifran M., Barozai M. Y., Rashid B., Riazuddin S., Husnain T. (2009). [Isolation and functional
analysis of cotton universal stress protein promoter in response to
phytohormones and abiotic stresses]. Mol Biol
(Mosk).

[3253] Feng, Z. ; Liu, N. ; Zhang, G. ; Bu, Y. ; Wang, B. ; Gong, Y. Promoter of Vegetable Soybean GmTIP1;6 Responds to Diverse Abiotic Stresses and Hormone Signals in Transgenic Arabidopsis. Int J Mol Sci 2022, 23 (20), 12684 10.3390/ijms232012684. 36293538 PMC9604487

[3254] Espasandin F. D., Collavino M. M., Luna C. V., Paz R. C., Tarragó J. R., Ruiz O. A., Mroginski L. A., Sansberro P. A. (2010). Agrobacterium tumefaciens-mediated transformation of
Lotus tenuis and regeneration of transgenic lines. Plant Cell, Tissue and Organ Culture (PCTOC).

[3255] Yang Y., Shao Y., Chaffin T. A., Lee J. H., Poindexter M. R., Ahkami A. H., Blumwald E., Stewart C. N. (2022). Performance of abiotic stress-inducible synthetic promoters
in genetically engineered hybrid poplar (Populus tremula x Populus
alba). Front Plant Sci.

[3256] Ganguly M., Roychoudhury A., Sarkar S. N., Sengupta D. N., Datta S. K., Datta K. (2011). Inducibility of three salinity/abscisic
acid-regulated promoters in transgenic rice with gusA reporter gene. Plant Cell Rep.

[3257] Suekawa M., Fujikawa Y., Inada S., Murano A., Esaka M. (2016). Gene expression and promoter analysis
of a novel tomato aldo-keto
reductase in response to environmental stresses. J Plant Physiol.

[3258] Ogita N., Okushima Y., Tokizawa M., Yamamoto Y. Y., Tanaka M., Seki M., Makita Y., Matsui M., Okamoto-Yoshiyama K., Sakamoto T. (2018). Identifying the target
genes of SUPPRESSOR OF GAMMA RESPONSE 1, a master transcription factor
controlling DNA damage response in Arabidopsis. Plant J.

[3259] Sears R. G., Rigoulot S. B., Occhialini A., Morgan B., Kakeshpour T., Brabazon H., Barnes C. N., Seaberry E. M., Jacobs B., Brown C. (2023). Engineered
gamma radiation phytosensors for environmental monitoring. Plant Biotechnol J.

[3260] Liu Q., Zhang Q., Burton R. A., Shirley N. J., Atwell B. J. (2010). Expression
of vacuolar H+-pyrophosphatase (OVP3) is under control of an anoxia-inducible
promoter in rice. Plant Molecular Biology.

[3261] Wang H., Liu C., Ma P. a., Lu C., Li K., Wang W. (2018). Functional
characterization of cytosolic pyruvate phosphate
dikinase gene (MecyPPDK) and promoter (MecyPPDKP) of cassava in response
to abiotic stress in transgenic tobacco. Crop
science.

[3262] Liu J.-J., Ekramoddoullah A. K.
M., Piggott N., Zamani A. (2005). Molecular cloning of a pathogen/wound-inducible PR10
promoter from Pinus monticola and characterization in transgenic Arabidopsis
plants. Planta.

[3263] Sasaki K., Yuichi O., Hiraga S., Gotoh Y., Seo S., Mitsuhara I., Ito H., Matsui H., Ohashi Y. (2007). Characterization
of two rice peroxidase promoters that respond to blast fungus-infection. Molecular Genetics and Genomics.

[3264] Chakravarthi M., Syamaladevi D. P., Harunipriya P., Augustine S. M., Subramonian N. (2016). A novel PR10
promoter from Erianthus
arundinaceus directs high constitutive transgene expression and is
enhanced upon wounding in heterologous plant systems. Molecular Biology Reports.

[3265] Mhiri C., Morel J.-B., Vernhettes S., Casacuberta J. M., Lucas H., Grandbastien M.-A. (1997). The promoter
of the tobacco Tnt1 retrotransposon is induced by wounding and by
abiotic stress. Plant Molecular Biology.

[3266] de Moura S. M., Freitas E. O., Ribeiro T. P., Paes-de-Melo B., Arraes F. B. M., Macedo L. L. P., Paixao J. F. R., Lourenco-Tessutti I. T., Artico S., da Cunha Valenca D. (2022). Discovery and functional
characterization of novel cotton promoters with potential application
to pest control. Plant Cell Rep.

[3267] Liu W., Mazarei M., Peng Y., Fethe M. H., Rudis M. R., Lin J., Millwood R. J., Arelli P. R., Stewart C. N. (2014). Computational discovery of soybean promoter cis-regulatory elements
for the construction of soybean cyst nematode-inducible synthetic
promoters. Plant Biotechnol J.

[3268] Hong J. K., Lee S. C., Hwang B. K. (2005). Activation of pepper
basic PR-1 gene promoter during defense signaling to pathogen, abiotic
and environmental stresses. Gene.

[3269] Samac D. A., Shah D. M. (1991). Developmental and
Pathogen-Induced
Activation of the Arabidopsis Acidic Chitinase Promoter. Plant Cell.

[3270] Zhang, S. A chemical reaction revolutionized farming 100 years ago. now it needs to go. Wired Magazine 2016.

[3271] Smil, V. Enriching the earth: Fritz Haber, Carl Bosch, and the transformation of world food production; MIT press, 2004.

[3272] Van Deynze, A. ; Zamora, P. ; Delaux, P. M. ; Heitmann, C. ; Jayaraman, D. ; Rajasekar, S. ; Graham, D. ; Maeda, J. ; Gibson, D. ; Schwartz, K. D. ; Nitrogen fixation in a landrace of maize is supported by a mucilage-associated diazotrophic microbiota. Plos Biol 2018, 16 (8), e2006352. 10.1371/journal.pbio.2006352 30086128 PMC6080747

[3273] Simmonds, J. Community matters: A history of biological nitrogen fixation and nodulation research, 1965 to 1995; Rensselaer Polytechnic Institute, 2007.

[3274] Jin H., Kim S. S., Venkateshalu S., Lee J., Lee K., Jin K. (2023). Electrochemical Nitrogen Fixation
for Green Ammonia: Recent Progress
and Challenges. Adv Sci (Weinh).

[3275] Bosworth A. H., Williams M. K., Albrecht K. A., Kwiatkowski R., Beynon J., Hankinson T. R., Ronson C. W., Cannon F., Wacek T. J., Triplett E. W. (1994). Alfalfa
Yield Response to Inoculation
with Recombinant Strains of Rhizobium-Meliloti with an Extra Copy
of Dctabd and/or Modified Nifa Expression. Appl
Environ Microb.

[3276] Scupham A. J., Bosworth A. H., Ellis W. R., Wacek T. J., Albrecht K. A., Triplett E. W. (1996). Inoculation with
Sinorhizobium meliloti
RMBPC-2 increases alfalfa yield compared with inoculation with a nonengineered
wild-type strain. Appl Environ Microb.

[3277] Ronson C. W., Bosworth A., Genova M., Gudbrandsen S., Hankinson T., Kwiatkowski R., Ratcliffe H., Robie C., Sweeney P., Szeto W. (1990). Field
Release of Genetically-Engineered Rhizobium-Meliloti and Bradyrhizobium-Japonicum
Strains. Nitrogen Fixation : Achievements and
Objectives.

[3278] Vidaver, A. K. ; Tolin, S. ; Post, A. The Status, Promise and Potential Perils of Commercially Available Genetically Modified Microorganisms in Agriculture and the Environment. Papers in Plant Pathology 2012, 260, 95. 10.1007/978-94-007-2156-2_5

[3279] Sinorhizobium meliloti strain RMBPC-2. EPA, Ed.; 1998; Vol. 40.

[3280] Wen A., Havens K. L., Bloch S. E., Shah N., Higgins D. A., Davis-Richardson A. G., Sharon J., Rezaei F., Mohiti-Asli M., Johnson A. (2021). Enabling Biological Nitrogen Fixation for Cereal Crops
in Fertilized Fields. ACS Synth Biol.

[3281] Temme, K. ; Shah, N. ; Ozaydin, B. ; Bloch, S. ; Tamsir, A. ; Fulop, D. Consortia of microorganisms for spatial and temporal delivery of nitrogen. EP4090642A4, 2023.

[3282] Martinez-Feria R., Simmonds M. B., Ozaydin B., Lewis S., Schwartz A., Pluchino A., McKellar M., Gottlieb S. S., Kayatsky T., Vital R. (2024). Genetic
remodeling of
soil diazotrophs enables partial replacement of synthetic nitrogen
fertilizer with biological nitrogen fixation in maize. Sci Rep.

[3283] Liu C., Sakimoto K. K., Colon B. C., Silver P. A., Nocera D. G. (2017). Ambient
nitrogen reduction cycle using a hybrid inorganic-biological system. Proc Natl Acad Sci U S A.

[3284] Ambrosio R., Ortiz-Marquez J. C. F., Curatti L. (2017). Metabolic engineering
of a diazotrophic bacterium improves ammonium release and biofertilization
of plants and microalgae. Metab Eng.

[3285] Jiang P., Ninfa A. J. (2009). Reconstitution of
Escherichia coli
glutamine synthetase adenylyltransferase from N-terminal and C-terminal
fragments of the enzyme. Biochemistry.

[3286] Schnabel T., Sattely E. (2021). Engineering Posttranslational Regulation
of Glutamine Synthetase for Controllable Ammonia Production in the
Plant Symbiont Azospirillum brasilense. Appl
Environ Microbiol.

[3287] Schnabel T., Sattely E. (2021). Improved Stability
of Engineered
Ammonia Production in the Plant-Symbiont Azospirillum brasilense. ACS Synth Biol.

[3288] Mowafy, A. M. ; El-ftouh, E. A. A. ; Sdiek, M. Y. ; Abdelshafi, S. A. ; Sallam, A. A. ; Agha, M. S. ; Abou Zeid, W. R. Nitrogen-Fixing ArchaeaArchaea and Sustainable Agriculture. In Nitrogen Fixing Bacteria: Sustainable Growth of Non-legumes; Maheshwari, D. K. , Dobhal, R. , Dheeman, S. Eds.; Springer Nature Singapore, 2022; pp 115-126.

[3289] Bae, H. S. ; Morrison, E. ; Chanton, J. P. ; Ogram, A. Methanogens Are Major Contributors to Nitrogen Fixation in Soils of the Florida Everglades. Appl Environ Microbiol 2018, 84 (7), 10.1128/AEM.02222-17. PMC5861825 29374038

[3290] Naitam M. G., Kaushik R. (2021). Archaea: An Agro-Ecological
Perspective. Curr Microbiol.

[3291] Bosworth A. H., Williams M. K., Albrecht K. A., Kwiatkowski R., Beynon J., Hankinson T. R., Ronson C. W., Cannon F., Wacek T. J., Triplett E. W. (1994). Alfalfa
yield response to inoculation
with recombinant strains of Rhizobium meliloti with an extra copy
of dctABD and/or modified nifA expression. Applied
and Environmental Microbiology.

[3292] Reisinger, M. ; Sanders, E. ; Broglie, R. ; Temme, K. Agricultural compositions comprising remodeled nitrogen fixing microbes. WO2020006064A3, 2024.

[3293] Geddes B.
A., Ryu M. H., Mus F., Garcia Costas A., Peters J. W., Voigt C. A., Poole P. (2015). Use of plant colonizing
bacteria as chassis for transfer of N(2)-fixation to cereals. Curr Opin Biotechnol.

[3294] Dixon R. A., Postgate J. R. (1972). Genetic transfer of nitrogen fixation
from Klebsiella pneumoniae to Escherichia coli. Nature.

[3295] Fox A. R., Soto G., Valverde C., Russo D., Lagares A., Zorreguieta Á., Alleva K., Pascuan C., Frare R., Mercado-Blanco J. (2016). Major cereal crops benefit
from biological nitrogen fixation when inoculated with the nitrogen-fixing
bacterium Pseudomonas protegens Pf-5 X940. Environmental
Microbiology.

[3296] Setten L., Soto G., Mozzicafreddo M., Fox A. R., Lisi C., Cuccioloni M., Angeletti M., Pagano E., Díaz-Paleo A., Ayub N. D. (2013). Engineering Pseudomonas protegens Pf-5 for Nitrogen
Fixation and its Application to Improve Plant Growth under Nitrogen-Deficient
Conditions. PLOS ONE.

[3297] Jing X., Cui Q., Li X., Yin J., Ravichandran V., Pan D., Fu J., Tu Q., Wang H., Bian X. (2020). Engineering Pseudomonas
protegens Pf-5 to improve its antifungal activity and nitrogen fixation. Microb Biotechnol.

[3298] Buren S., Young E. M., Sweeny E. A., Lopez-Torrejon G., Veldhuizen M., Voigt C. A., Rubio L. M. (2017). Formation
of Nitrogenase
NifDK Tetramers in the Mitochondria of Saccharomyces cerevisiae. ACS Synth Biol.

[3299] Woodruff L. B. A., Gorochowski T. E., Roehner N., Mikkelsen T. S., Densmore D., Gordon D. B., Nicol R., Voigt C. A. (2017). Registry
in a tube: multiplexed pools of retrievable parts for genetic design
space exploration. Nucleic Acids Res.

[3300] Paschen A., Drepper T., Masepohl B., Klipp W. (2001). Rhodobacter
capsulatus nifA mutants mediating nif gene expression in the presence
of ammonium. FEMS Microbiol Lett.

[3301] Rey F. E., Heiniger E. K., Harwood C. S. (2007). Redirection of metabolism
for biological hydrogen production. Appl Environ
Microbiol.

[3302] Oger P., Petit A., Dessaux Y. (1997). Genetically engineered
plants producing opines alter their biological environment. Nat Biotechnol.

[3303] Bressan M., Roncato M. A., Bellvert F., Comte G., Haichar F. Z., Achouak W., Berge O. (2009). Exogenous
glucosinolate
produced by Arabidopsis thaliana has an impact on microbes in the
rhizosphere and plant roots. ISME J.

[3304] Badri D. V., Quintana N., El Kassis E. G., Kim H. K., Choi Y. H., Sugiyama A., Verpoorte R., Martinoia E., Manter D. K., Vivanco J. M. (2009). An ABC transporter
mutation alters root exudation of phytochemicals that provoke an overhaul
of natural soil microbiota. Plant Physiol.

[3305] Singh V. S., Dubey B. K., Rai S., Singh S. P., Tripathi A. K. (2022). Engineering
D-glucose utilization in Azospirillum brasilense
Sp7 promotes rice root colonization. Appl Microbiol
Biotechnol.

[3306] Glick B. R. (2012). Plant growth-promoting bacteria: mechanisms and applications. Scientifica (Cairo).

[3307] Sasse J., Martinoia E., Northen T. (2018). Feed Your Friends:
Do Plant Exudates Shape the Root Microbiome?. Trends Plant Sci.

[3308] Mendes R., Kruijt M., de Bruijn I., Dekkers E., van der Voort M., Schneider J. H., Piceno Y. M., DeSantis T. Z., Andersen G. L., Bakker P. A. (2011). Deciphering the rhizosphere microbiome for disease-suppressive bacteria. Science.

[3309] Jhu M.-Y., Oldroyd G. E. (2023). Dancing to a different
tune, can
we switch from chemical to biological nitrogen fixation for sustainable
food security?. PLoS Biology.

[3310] Guo K., Yang J., Yu N., Luo L., Wang E. (2023). Biological
nitrogen fixation in cereal crops: Progress, strategies, and perspectives. Plant Commun.

[3311] Huisman R., Geurts R. (2020). A Roadmap toward Engineered Nitrogen-Fixing
Nodule Symbiosis. Plant Commun.

[3312] Banba M., Gutjahr C., Miyao A., Hirochika H., Paszkowski U., Kouchi H., Imaizumi-Anraku H. (2008). Divergence
of evolutionary ways among common sym genes: CASTOR and CCaMK show
functional conservation between two symbiosis systems and constitute
the root of a common signaling pathway. Plant
Cell Physiol.

[3313] Rogers C., Oldroyd G. E. (2014). Synthetic biology approaches to engineering
the nitrogen symbiosis in cereals. J Exp Bot.

[3314] Altuzar-Molina A., Lozano L., Ortiz-Berrocal M., Ramirez M., Martinez L., de Lourdes Velazquez-Hernandez M., Dhar-Ray S., Silvente S., Mariano N., Shishkova S., Hernandez G., Reddy P. M. (2020). Expression of the Legume-Specific
Nod Factor Receptor Proteins Alters Developmental and Immune Responses
in Rice. Plant Molecular Biology Reporter.

[3315] He J., Zhang C., Dai H., Liu H., Zhang X., Yang J., Chen X., Zhu Y., Wang D., Qi X. (2019). A LysM Receptor Heteromer
Mediates Perception of Arbuscular
Mycorrhizal Symbiotic Signal in Rice. Mol Plant.

[3316] Untergasser A., Bijl G. J., Liu W., Bisseling T., Schaart J. G., Geurts R. (2012). One-step Agrobacterium mediated transformation
of eight genes essential for rhizobium symbiotic signaling using the
novel binary vector system pHUGE. PLoS One.

[3317] Haskett T. L., Geddes B. A., Paramasivan P., Green P., Chitnavis S., Mendes M. D., Jorrin B., Knights H. E., Bastholm T. R., Ramsay J. P. (2023). Rhizopine
biosensors for plant-dependent control of bacterial gene expression. Environ Microbiol.

[3318] Murphy P. J., Wexler W., Grzemski W., Rao J. P., Gordon D. (1995). Rhizopines—Their role in symbiosis
and competition. Soil Biology and Biochemistry.

[3319] Russell C. (1975). Biologists
draft genetic research guidelines. Bioscience.

[3320] Larrea-Alvarez, M. ; Purton, S. The Chloroplast of Chlamydomonas reinhardtii as a Testbed for Engineering Nitrogen Fixation into Plants. Int J Mol Sci 2021, 22 (16), 8806 10.3390/ijms22168806 . 34445505 PMC8395883

[3321] Jiang X., Coroian D., Barahona E., Echavarri-Erasun C., Castellanos-Rueda R., Eseverri A., Aznar-Moreno J. A., Buren S., Rubio L. M. (2022). Functional Nitrogenase Cofactor Maturase
NifB in Mitochondria and Chloroplasts of Nicotiana benthamiana. mBio.

[3322] Eseverri A., Lopez-Torrejon G., Jiang X., Buren S., Rubio L. M., Caro E. (2020). Use of synthetic biology tools to
optimize the production of active nitrogenase Fe protein in chloroplasts
of tobacco leaf cells. Plant Biotechnol J.

[3323] Lopez-Torrejon G., Jimenez-Vicente E., Buesa J. M., Hernandez J. A., Verma H. K., Rubio L. M. (2016). Expression of a functional oxygen-labile
nitrogenase component in the mitochondrial matrix of aerobically grown
yeast. Nat Commun.

[3324] Buren S., Pratt K., Jiang X., Guo Y., Jimenez-Vicente E., Echavarri-Erasun C., Dean D. R., Saaem I., Gordon D. B., Voigt C. A. (2019). Biosynthesis of the
nitrogenase active-site cofactor precursor NifB-co in Saccharomyces
cerevisiae. Proc Natl Acad Sci U S A.

[3325] Yang J., Xiang N., Liu Y., Guo C., Li C., Li H., Cai S., Dixon R., Wang Y. P. (2023). Organelle-dependent
polyprotein designs enable stoichiometric expression of nitrogen fixation
components targeted to mitochondria. Proc Natl
Acad Sci U S A.

[3326] Baysal C., Buren S., He W., Jiang X., Capell T., Rubio L. M., Christou P. (2022). Functional
expression
of the nitrogenase Fe protein in transgenic rice. Commun Biol.

[3327] He W., Buren S., Baysal C., Jiang X., Capell T., Christou P., Rubio L. M. (2022). Nitrogenase Cofactor Maturase NifB
Isolated from Transgenic Rice is Active in FeMo-co Synthesis. ACS Synth Biol.

[3328] Sherbo R. S., Silver P. A., Nocera D. G. (2022). Riboflavin
synthesis
from gaseous nitrogen and carbon dioxide by a hybrid inorganic-biological
system. Proc Natl Acad Sci U S A.

[3329] Llorente, B. ; Williams, T. C. ; Goold, H. D. The Multiplanetary Future of Plant Synthetic Biology. Genes (Basel) 2018, 9 (7), 348 10.3390/genes9070348. 29996548 PMC6071031

[3330] Verseux C. N., Paulino-Lima I. G., Baqué M., Billi D., Rothschild L. J. (2016). Synthetic
biology for space exploration:
promises and societal implications. Ambivalences
of creating life: Societal and philosophical dimensions of synthetic
biology.

[3331] Coleman M. A., Goold H. D. (2019). Harnessing synthetic biology for
kelp forest conservation(1). J Phycol.

[3332] Mitchell C. A. (2022). History
of controlled environment horticulture: Indoor
farming and its key technologies. HortScience.

[3333] Zhang, L. ; Yang, X. ; Li, T. ; Gan, R. ; Wang, Z. ; Peng, J. ; Hu, J. ; Guo, J. ; Zhang, Y. ; Li, Q. ; Plant factory technology lights up urban horticulture in the post-coronavirus world. Hortic Res 2022, 10.1093/hr/uhac018. PMC9016861 35184181

[3334] Bacilio M., Moreno M., Lopez-Aguilar D. R., Bashan Y. (2017). Scaling from the growth
chamber to the greenhouse to
the field: Demonstration of diminishing effects of mitigation of salinity
in peppers inoculated with plant growth-promoting bacterium and humic
acids. Applied Soil Ecology.

[3335] Reisman H. B. (1993). Problems in scale-up of biotechnology
production processes. Crit Rev Biotechnol.

[3336] Formenti L. R., Norregaard A., Bolic A., Hernandez D. Q., Hagemann T., Heins A. L., Larsson H., Mears L., Mauricio-Iglesias M., Kruhne U. (2014). Challenges in industrial
fermentation technology research. Biotechnol
J.

[3337] Yu K., Rinklebe J. (2011). Advancement in soil microcosm apparatus for biogeochemical
research. Ecological Engineering.

[3338] Tourna M., Freitag T. E., Nicol G. W., Prosser J. I. (2008). Growth,
activity and temperature responses of ammonia-oxidizing archaea and
bacteria in soil microcosms. Environ Microbiol.

[3339] Trevors J. T., Van Elsas J., Van Overbeek L., Starodub M. (1990). Transport of a genetically
engineered Pseudomonas fluorescens
strain through a soil microcosm. Applied and
environmental microbiology.

[3340] Sjogren R. E. (1994). Prolonged survival of an environmental
Escherchia coli
in laboratory soil microcosms. Water, Air, and
Soil Pollution.

[3341] Van
Elsas J., Trevors J., Van Overbeek L. (1991). Influence
of soil properties on the vertical movement of genetically-marked
Pseudomonas fluorescens through large soil microcosms. Biology and Fertility of soils.

[3342] Gao J., Sasse J., Lewald K. M., Zhalnina K., Cornmesser L. T., Duncombe T. A., Yoshikuni Y., Vogel J. P., Firestone M. K., Northen T. R. (2018). Ecosystem fabrication
(EcoFAB) protocols for the construction
of laboratory ecosystems designed to study plant-microbe interactions. JoVE (Journal of Visualized Experiments).

[3343] Sasse J., Kant J., Cole B. J., Klein A. P., Arsova B., Schlaepfer P., Gao J., Lewald K., Zhalnina K., Kosina S. (2019). Multilab EcoFAB study
shows highly
reproducible physiology and depletion of soil metabolites by a model
grass. New Phytologist.

[3344] Zhang X., Cai X. (2011). Climate change impacts
on global
agricultural land availability. Environmental
Research Letters.

[3345] Tilman D. (1999). Global environmental impacts of agricultural expansion:
the need for sustainable and efficient practices. Proc Natl Acad Sci U S A.

[3346] Food and Agriculture Organization of the United Nations Land use in agriculture by the numbers. 2020. https://www.fao.org/sustainability/news/detail/en/c/1274219/ (accessed 12/11/2024).

[3347] Mbow, C. ; Rosenzweig, C. E. ; Barioni, L. G. ; Benton, T. G. ; Herrero, M. ; Krishnapillai, M. ; Ruane, A. C. ; Liwenga, E. ; Pradhan, P. ; Rivera-Ferre, M. G. Food security; IPCC, 2020. 10.1038/s43016-020-0031-z 37128000

[3348] Lynch J., Cain M., Frame D., Pierrehumbert R. (2021). Agriculture's
contribution to climate change and role in mitigation is distinct
from predominantly fossil CO2-emitting sectors. Frontiers in sustainable food systems.

[3349] Sajid M., Stone S. R., Kaur P. (2021). Transforming traditional
nutrition paradigms with synthetic biology driven microbial production
platforms. Current Research in Biotechnology.

[3350] Lv X., Wu Y., Gong M., Deng J., Gu Y., Liu Y., Li J., Du G., Ledesma-Amaro R., Liu L. (2021). Synthetic biology for
future food: research progress and future directions. Future Foods.

[3351] Shi S., Wang Z., Shen L., Xiao H. (2022). Synthetic biology:
a new frontier in food production. Trends Biotechnol.

[3352] Arun K., Anoopkumar A., Sindhu R., Binod P., Aneesh E. M., Madhavan A., Awasthi M. K. (2023). Synthetic biology
for sustainable food ingredients production: recent trends. Systems Microbiology and Biomanufacturing.

[3353] Shankar, S. ; Hoyt, M. A. Expression constructs and methods of genetically engineering methylotrophic yeast. WO2016183163A1, 2018.

[3354] Tyagi A., Kumar A., Aparna S., Mallappa R. H., Grover S., Batish V. K. (2016). Synthetic biology:
applications in
the food sector. Critical Reviews in Food Science
and Nutrition.

[3355] Zhang C., Liyang S., Jianwu D. (2022). Cultured meat from
biomaterials: challenges and prospects. Synthetic
Biology Journal.

[3356] Antonovsky N., Gleizer S., Noor E., Zohar Y., Herz E., Barenholz U., Zelcbuch L., Amram S., Wides A., Tepper N. (2016). Sugar Synthesis from
CO2 in Escherichia coli. Cell.

[3357] Gleizer S., Ben-Nissan R., Bar-On Y. M., Antonovsky N., Noor E., Zohar Y., Jona G., Krieger E., Shamshoum M., Bar-Even A. (2019). Conversion of Escherichia
coli to Generate All Biomass Carbon from CO(2). Cell.

[3358] Gassler T., Sauer M., Gasser B., Egermeier M., Troyer C., Causon T., Hann S., Mattanovich D., Steiger M. G. (2020). The industrial yeast Pichia pastoris is converted from
a heterotroph into an autotroph capable of growth on CO2. Nature Biotechnology.

[3359] Takefuji Y. (2021). Sustainable protein alternatives. Trends in Food Science & Technology.

[3360] Turrell C. (2023). From air to your plate: tech startups
making food from
atmospheric CO(2). Nat Biotechnol.

[3361] Stokstad E. (2023). Unmixed blessing. Science.

[3362] Xu P., Bhan N., Koffas M. A. (2013). Engineering
plant metabolism into
microbes: from systems biology to synthetic biology. Current opinion in biotechnology.

[3363] Galanie S., Thodey K., Trenchard I. J., Filsinger Interrante M., Smolke C. D. (2015). Complete biosynthesis of opioids
in yeast. Science.

[3364] Jamil O. K., Cravens A., Payne J. T., Kim C. Y., Smolke C. D. (2022). Biosynthesis of tetrahydropapaverine
and semisynthesis
of papaverine in yeast. Proc Natl Acad Sci U
S A.

[3365] Srinivasan P., Smolke C. D. (2020). Biosynthesis of medicinal tropane
alkaloids in yeast. Nature.

[3366] Paddon C. J., Westfall P. J., Pitera D. J., Benjamin K., Fisher K., McPhee D., Leavell M. D., Tai A., Main A., Eng D. (2013). High-level semi-synthetic
production of the potent antimalarial artemisinin. Nature.

[3367] Ajikumar P. K., Xiao W. H., Tyo K. E., Wang Y., Simeon F., Leonard E., Mucha O., Phon T. H., Pfeifer B., Stephanopoulos G. (2010). Isoprenoid
pathway optimization for
Taxol precursor overproduction in Escherichia coli. Science.

[3368] Lin G.-M., Voigt C. A. (2023). Design of a redox-proficient
Escherichia
coli for screening terpenoids and modifying cytochrome P450s. Nature Catalysis.

[3369] Ma Y., Zu Y., Huang S., Stephanopoulos G. (2023). Engineering
a universal and efficient platform for terpenoid synthesis in yeast. Proc Natl Acad Sci U S A.

[3370] Hansen E. H., Moller B. L., Kock G. R., Bunner C. M., Kristensen C., Jensen O. R., Okkels F. T., Olsen C. E., Motawia M. S., Hansen J. (2009). De novo biosynthesis
of vanillin
in fission yeast (Schizosaccharomyces pombe) and baker's yeast
(Saccharomyces
cerevisiae). Appl Environ Microbiol.

[3371] Tan H., Chen X., Liang N., Chen R., Chen J., Hu C., Li Q., Li Q., Pei W., Xiao W. (2019). Transcriptome analysis reveals novel enzymes for apo-carotenoid biosynthesis
in saffron and allows construction of a pathway for crocetin synthesis
in yeast. J Exp Bot.

[3372] Olsson K., Carlsen S., Semmler A., Simon E., Mikkelsen M. D., Moller B. L. (2016). Microbial production
of next-generation
stevia sweeteners. Microb Cell Fact.

[3373] Ford C. Z., Sayler G. S., Burlage R. S. (1999). Containment
of a
genetically engineered microorganism during a field bioremediation
application. Appl Microbiol Biotechnol.

[3374] Eapen S., D'Souza S. F. (2005). Prospects of genetic engineering
of plants for phytoremediation of toxic metals. Biotechnol Adv.

[3375] Anderson C., Brooks R., Chiarucci A., LaCoste C., Leblanc M., Robinson B., Simcock R., Stewart R. (1999). Phytomining for nickel, thallium and gold. Journal of geochemical exploration.

[3376] Kikis C., Thalassinos G., Antoniadis V. (2024). Soil phytomining:
recent developments—a review. Soil Systems.

[3377] Chaney R. L., Baker A. J., Morel J. L. (2021). The long
road to
developing agromining/phytomining. Agromining:
farming for metals: extracting unconventional resources using plants.

[3378] Gebru, K. A. ; Kidanemariam, T. G. ; Gebretinsae, H. K. Bio-cement production using microbially induced calcite precipitation (MICP) method: A review. Chemical Engineering Science 2021, 238, 116610. 10.1016/j.ces.2021.116610

[3379] Cumbers, J. Can We Redesign The Modern City With Synthetic Biology? Could We Grow Our Houses Instead Of Building Them? In Forbes, 2019.

